# Supplementary material for: Nitrogen cost minimization is promoted by structural changes in the transcriptome of N-deprived Prochlorococcus cells
Source: ISME J. 2017 Jun 6;11(10):2267–78. doi: 10.1038/ismej.2017.88 (PMC5607370; doi:10.1038/ismej.2017.88)
Supplement: Supplementary Material Guide [file ismej201788x1.docx]

Supplemental Material Guide

Figures:

Figure 1: Transcriptional mapping for the *Prochlorococcus* Med4 *urt* Operon.

Figure 2: Antisense Transcriptional Mapping for *Prochlorococcus* Med4 PMM1552 Gene.

Figure 3: Antisense transcriptional mapping for the *Prochlorococcus* Med4 gene PMM1312.

Figure 4: Transcriptional Mapping for *Prochlorococcus* Med4 Unknown Peak.

Tables:

Table 1: Raw Sequence Counts and Mean Sequence Counts per Read for each treatment.

Table 2: Correlation of expression values at the various sampling times between two datasets -- this study and Tolonen et al. (2006).

Tables 3-7 Differentially expressed transcripts at the various sampling times

Table 3: Gene Expression For Genes with Top 50% Expression Values during 3 Hours Post Starvation.

Table 4: Gene Expression For Genes with Top 50% Expression Values during 12 Hours Post Starvation.

Table 5: Gene Expression For Genes with Top 50% Expression Values during 24 Hours Post Starvation.

Table 6: Transcripts Described During 12 and 24 Hours Post Starvation.

Table 7: High Light Inducible Protein 12 Hour Fold Changes.

Tables 8-11 TSSAR identified transcriptional start sites after 12 and 24 hours of N deprivation.

Table 8: N-Replete Transcriptional Start Sites at 12 Hours Post Starvation Identified by TSSAR.

Table 9: N-Deprived Transcriptional Start Sites at 12 Hours Post Starvation Identified by TSSAR.

Table 10: N-Replete Transcriptional Start Sites at 24 Hours Post Starvation Identified by TSSAR.

Table 11: N-Deprived Transcriptional Start Sites at 24 Hours Post Starvation Identified by TSSAR.

Tables 12-13 Transcriptional start site comparisons of primary and internal sites and comparison to Voigt et al., 2014

Table 12: Internal Start Site Comparison of This Study to Voigt et al., (2014). Internal TSSs identified by TSSAR were directly compared to Voigt et al. to determine presence or absence in their study. Genes in bold did not have an internal TSS in their study.

Table 13: Ratio of internal to primary start site utilization based on read mappings and TSSAR. All results are for TSSAR identified internal start sites with a p value of 0. ID column matches to results found in Tables S8-S9.

Tables 14-15 Protein threading results and possible translational start sites

Table 14: Sequence IDs and protein threading results from transcripts with significant internal transcriptional starts sites in MED4 under N-limitation.

Table 15: Relative number of pyrimidine-rich regions and Possible NtcA binding sites.

Tables 16-19 Final gene expression comparisons and operon prediction

Table 16: Autocorrelation of Expression Values for Three Datasets -- This study all data, This study top 50% expression values and Tolonen et al. (2006).

Table 17: Transcripts Described During 3 Hours Post Starvation.

Table 18: Ribosomal Transcripts with Significant Regulation 12 and 24 Hours Post Starvation.

Table 19: Predicted Operon Organization by Rockhopper.
