# Supplementary material for: Nitrogen cost minimization is promoted by structural changes in the transcriptome of N-deprived Prochlorococcus cells
Source: ISME J. 2017 Jun 6;11(10):2267–78. doi: 10.1038/ismej.2017.88 (PMC5607370; doi:10.1038/ismej.2017.88)
Supplement: Supplementary Methods [file ismej201788x2.docx]

**Supplemental Methods**

**Differential Expression**

For the differential expression measurements calculated by Rockhopper (McClure *et al.*, 2013), the following was completed. First the reference species was chosen as *Prochlorococcus marinus* subsp. pastoris str. CCMP1986. Next the parameter settings were changed to make the minimum seed length 15% of the read length, and to allow mismatches up to 15% of the read of the read length. Following this, replicates were imported for both the control and experimental treatments, and the program was run comparing the two treatments against each other. The resulting “transcripts” file contains the count data, expression values and p-values for each of the genes in Prochlorococcus.

**Transcriptional Start Site Identification**

Raw Sequencing Reads were aligned to the Prochlorococcus MED4 reference genome using the segemehl (Hoffmann *et al.*, 2009; 2014). First an index file was made using the downloaded *Prochlorococcus* MED4 fna (genome) file. Next the primary transcriptome files from each treatment were aligned to the reference genome using the command:

segemehl.x -i Pro.idx -d Pro.fna -q T0.fastq -t 60 -o T0_TSS.sam

The resulting sam file was converted to a sorted and indexed bam file using the following commands with samtools (Li *et al.*, 2009):

samtools view –bS TSS.sam > TSS.bam

samtools sort TSS.bam TSS_sorted

samtools index TSS_sorted.bam

These resulting bam files were uploaded into TSSAR (Amman *et al.*, 2014). First the NCBI ID of NC_005072 was added to produce the correct genome size for *Prochlorococcus* MED4. The window size was set to 200 as this is the average gene length in *Prochlorococcus* MED4. The 5’ RACE-seq data was uploaded into the plus library, while the non-primary TSS sequencing data was uploaded into the minus library for both 12 hours post N starvation and 24 hours post N starvation. The data was then processed by TSSAR. The resulting files were subject to TSSAR parameters of a p-value ≤ 1x10^-10^, a noise threshold of 4 and a merge range of 5. The resulting predicted TSS classification file was then downloaded.

For hand curation, reads were uploaded into the IGV program (Robinson *et al.*, 2011; Thorvaldsdóttir *et al.*, 2013). A genome file was created using the Prochlorococcus MED4 fna and gbk file. Once this genome file was loaded, the sorted bam files from each treatment and time point could directly be loaded into IGV. The results from TSSAR were compared to the actual read mappings for the control and experimental treatments at both 12 and 24 hours post N starvation. To be as robust as possible during the hand curation, each predicted TSS by TSSAR was compared to the actual read mappings and only the strongest and most abundant TSSs were kept, with any predicted start site with less than 100 reads removed. A very rare occasion would occur where TSSAR would miss a primary TSS, in that case, the location of the TSS was marked and the difference was estimated based on the amount of reads present in the 5’ RACE-seq sample compared to the non primary TSS sample.

**Supplemental Results and Discussion**

**Dataset Comparison To Previous Work**

Fold changes calculated from TEX-treated RNA were compared to those observed in Tolonen et al. 2006 (Tolonen *et al.*, 2006). Pearson correlation coefficients were calculated comparing time points within (Supplementary Table S16) and across datasets (Supplementary Table S2). During 3 hours post starvation, our results were not correlated with Tolonen. This may be due to the low read mapping of the 3 hour sequences, with raw counts mapping on average 30% less than the raw counts from 12 and 24 hours post starvation (Supplementary Table S1). However, beginning 12 hours post starvation and continuing through 24 hours post starvation our data was positively correlated with Tolonen, with Pearson correlation coefficients of 0.589 and 0.584 respectively (Supplementary Table S2). When using the top 50% of the expression values from each time point, the Pearson correlation coefficients increased to 0.647 and 0.723 (Supplementary Table S2).

Time point comparisons within each dataset displayed a strong positive correlation between 12 and 24 hours for our dataset and for Tolonen. Our dataset produced a Pearson correlation coefficient of 0.892 for the top 50% of the expression values, while Tolonen’s dataset had a coefficient of 0.772 (Supplementary Table S16). Tolonen also displayed a positive correlation between the 3-hour dataset and the 12/24 hour dataset. This was not evident in our dataset and may again be due to the lower read mapping of our 3-hour sequences.

**Transcriptional Responses to N Starvation**

*3 Hours Post Starvation*

Transcriptional changes begin to occur within 3 hours of N starvation (Supplementary Table S3). Many of the transcripts showing large increases in abundance during the 3 hour time point dropped down to almost control levels during 12 and 24 hours post N starvation (Supplementary Table S3). To gain a further understanding of the transcriptional response, transcripts were grouped by functional category using the CyanoBase annotations (Nakao *et al.*, 2010). The two largest categories contained hypothetical proteins and proteins with a function listed as "Other". Analysis of the "Other" category identified several transcripts which function to produce ammonia for the cell (Supplementary Tables S3 & S17), perhaps providing an alternative N source at the inception of N starvation (Bryant *et al.*, 1994). One of which is cyanate lyase (*cynS*), which catalyzes the decomposition of cyanate (NCO^-^) into carbon dioxide (CO_2_) and ammonia (NH_3_), and is only found in certain strains of cyanobacteria (García-Fernández *et al.*, 2004; Kamennaya and Post, 2011). Abundance decreases significantly between 3 hours post starvation and 12 hours post starvation, however, it is still increased over control levels (Supplementary Table S17). CynS transcription relies on the *P. marinus* *ntcA* gene (Harano *et al.*, 1997). NtcA is only slightly enriched during 3 and 12 hours post starvation (Supplementary Table S17). At 24 hours the gene was significantly enriched with a log_2_ fold change of 2.17 ± 1.20. Interestingly, we didn't observe any differential gene expression changes in the N regulatory protein P-II. This regulatory protein plays an important role in N metabolism especially under N stress (Forchhammer, 2004). Our data indicate that it began to show increased abundance 12 and 24 hours post starvation, however, the results were not significant. Acetylornithine aminotransferase -- a gene important in the N side-chain containing amino acid synthesis of Arg and Lys -- is a leaderless mRNA with complex dynamics. The gene decreased in abundance at 3 hours post-starvation and then highly enriched again at 12 and 24 hours post starvation. The ribosomal gene *rpsB* has a 5' UTR ~ 89 bp in length- the gene showed progressively decreased abundance over the course of N starvation

The protein serine hydroxymethyltransferase (SHMT), could provide an alternative source of N. SHMT converts two molecules of glycine to one molecule of serine, ammonia, and carbon dioxide (Herrero and Flores, 2008). The gene was enriched at 3 hours post starvation (log_2_ fold change of 2.05 ± 0.685, p-value =0.027) (Supplementary Table S17). Lipoic acid synthase also increased in abundance during 3 hours post starvation (Supplementary Table S17). Interestingly, lipoic acid was first characterized as a necessary growth factor for bacterial survival, and later as a member of the vitamin B family (Snell *et al.*, 1937; Reed *et al.*, 1951). More recently, it was identified as a powerful antioxidant in humans, as well as having a role in the prevention of complications from certain diseases (Packer *et al.*, 1995; 2001). It showed a similar pattern of regulation to a nickel-containing superoxide dismutase transcript from our dataset (Supplementary Table S17), thus it could possibly be functioning in a similar antioxidant role in *Prochlorococcus* Med4.

The major ammonium transport gene, *amt1*, was significantly enriched at 3 hours post starvation with a log_2_ fold change of 3.67 ± 0.28 (Supplementary Table S17). Amt1 is activated during N starvation (Vázquez-Bermúdez *et al.*, 2002; Tolonen *et al.*, 2006). Expression peaks at 3 hours and slightly declines as N starvation persists although it’s still significantly enriched. Other transporters, such as the urea transporter and cyanate transporter, have roles in uptake and assimilation of other N sources. Transcripts mapping to the *urtAB* genes, which are a part of the urea transporter operon were enriched, but not until 12 hours post starvation (Supplementary Table S17).

Carbon fixation, photosystem II and photosystem I were other functional categories containing enriched transcripts within 3 hours post starvation (Supplementary Table S3). Several transcripts mapped to core and peripheral proteins within both photosystem I and II. Furthermore both subunits of RuBisCO were found to be enriched within 3 hours post starvation (Supplementary Tables S3 & S17).

Of the 28 transcripts which showed a log_2_ fold change ≤ -2, many mapped to hypothetical proteins (Supplementary Table S3). One of the non-hypothetical proteins with a large decrease in abundance was the high light 20 (*hli20*) gene (Supplementary Table S17). In contrast to other high light proteins, which were hypothesized to be quickly enriched under high light and other stressful conditions in order to protect photosystem I and II (Havaux *et al.*, 2003; Tolonen *et al.*, 2006) (Supplementary Table S7), this protein demonstrated decreased abundance throughout the entire experiment versus the control. It may have an alternative function compared to other high light proteins in our dataset. Another transcript that demonstrated large decreases in abundance within 3 hours post starvation was the DNA repair protein *ruvB* (Supplementary Tables S3 & S17). Though, this gene is highly enriched 12 hours post starvation, along with other repair proteins (Supplementary Table S6).

*12 and 24 Hours Post Starvation*

Following 12 hours post N starvation, large changes were occurring within the cell (Supplementary Tables S4-S6). The majority (63 out of 84) of transcripts that were enriched 12 hours post starvation were also enriched 24 hours post starvation (Supplementary Tables S4-S7, S18). We observed 15 high light inducible proteins that were all enriched with an average log_2_ fold change of 3.94 and an average p-value of 1.14x10^-5^ (Supplementary Table S7). These transcripts were some of the most differentially expressed proteins at 12 hours post starvation, coherent with previous findings (Tolonen *et al.*, 2006). These genes increase their abundance in response to environmental stressors, such as nutrient, or light stress (Salem and van Waasbergen, 2004; Tolonen *et al.*, 2006), and possibly work to protects the cells by stabilizing the photosystems during stress (Wang *et al.*, 2008).

Sequencing results suggest that after 12 hours post starvation, nitrogen assimilation genes were significantly enriched as a general nitrogen starvation response. In addition to the cyanate, urea transporter, and urease subunits above, an accessory urease protein, *ureG* increased in abundance. Both urease subunits along with the accessory gene transcript demonstrated this same pattern of transcription organization during 24 hours post starvation, and as such were also enriched for 24 hours post starvation. Transcripts encoding proteins that function in degradation of proteins, peptides, and glycopeptides were highly enriched, including the Clp protease proteolytic subunit and chymotrypsin serine protease (Supplementary Table S6). These proteases are known to degrade misfolded and damaged proteins in cyanobacteria, (Clarke, 1999) -- N stress is likely causing the accumulation damaged proteins that need to be recycled or destroyed. This increased turnover of processed and damaged proteins may allow for the replenishment of the amino acid pool, which is likely to be constrained during nitrogen starvation.

DNA damage is possibly occurring at 12 hours post starvation; three important DNA repair transcripts increased their abundances (Supplementary Table S6). The first was a ssDNA binding protein transcript, hereafter identified as SSB. SSB's bind with high affinity to ssDNA, and play a role in the induction of the bacterial SOS damage response (Meyer and Laine, 1990). The two other SOS genes were *recA* and *lexA*. The *recA* gene was enriched during 12 and 24 hours post starvation (Supplementary Tables S4 & S5). The *lexA* gene decreases in expression between 3 and 12 hours post starvation (Supplementary Table S4). In addition to the SOS proteins, the Holliday junction DNA helicase *ruvB* transcript also increased in abundance. Cyanobacterial ruv genes participate in strand exchange during homologous recombination, as a way to repair DNA damage (Domain *et al.*, 2004). The continued enrichment of both *recA* and *ruvB* genes also suggests that DNA damage is possibly also occurring after 24 hours of N starvation.

The transcript encoding the photosystem II D1 protein was highly expressed and increased in abundance during 12 hours post starvation. The D1 protein is part of a heterodimer, which along with the D2 protein, make up the main reaction center for photosystem II (Rutherford and Faller, 2003). The D1 protein has a very high light dependent turnover rate (Mattoo *et al.*, 1984). We hypothesize that PSII is being damaged, and D1 protein turnover cannot be maintained due to inefficient protein synthesis during N starvation. This is similarly reflected in decreases in the maximum quantum yield for PSII.

Many transcripts with significantly decreased abundance 12 and 24 hours post starvation group into the categories of photosynthesis, carbon fixation, or protein synthesis (Supplementary Tables S6 & S18). Both chains of RuBisCO were decreased, although only the large chain was considered significant. This was expected as RuBisCO is the most abundant protein, and thus a large sink for N. Seven genes from photosystem II and seven genes from photosystem I also displayed decreased abundance. Furthermore, 24 ribosomal proteins were decreased. Importantly, although photosynthetic mechanisms display severe decreases in abundance, it is likely that a basal photosynthetic level is being maintained for long term survival and essential protein synthesis, as is seen in a related cyanobacteria (Sauer *et al.*, 2001).

**Operon structure under N starvation**

We examined whether the operon structure of N assimilatory genes changed in response to N limitation. Rockhopper computational analysis (McClure *et al.*, 2013) identified 367 predicted multi-gene operons, ranging in size from 247 to 10,649 bp (Supplementary Table S19). Some of the most highly expressed operons under N-starvation conditions were those containing urease subunits and putative urea transporters. Urease genes allow for cyanobacteria to utilize urea as a source of N for the cell. In *Prochlorococcus,* the urease enzyme consists of three subunits (*ureA*, *ureB*, *ureC*) and four accessory proteins (*ureD*, *ureE*, *ureF*, *ureG*) (Palinska *et al.*, 2000). The urea transporter genes (*urtA-E*) were also organized in the same way.

**Antisense RNA Abundance**

In contrast to the differential utilization of internal TSSs between N-replete and N-depleted samples, we observed that the number of antisense TSSs was approximately equal in both the N-replete and N-starved samples (Table 2). If antisense RNA regulators are as effective as protein regulators in Prochlorococcus, then antisense RNA regulators could further reduce the cellular N requirement. Antisense RNAs can modulate gene expression through a variety of mechanisms that either increase or decrease RNA stability, promote RNA degradation, attract transcription initiation proteins or alter translation processes (Georg and Hess, 2011; Pelechano and Steinmetz, 2013).

Genes within antisense TSSs don’t always correlate with notable differences in gene expression, however. Several of the genes with antisense TSSs were previously identified as constitutively expressed in *Prochlorococcus* MED4 after 12 hours of N stress (Tolonen *et al.*, 2006), consistent with our observations in this study. There were a few examples of transcripts where gene expression may have been altered by antisense RNAs though. Transcripts encoded by PMM1312 – a putative nuclease with roles in DNA replication and repair (Marchler-Bauer *et al.*, 2015) – were expressed from an antisense TSS to a much greater relative level in the N-starved cells compared to the N-replete cells (Fig. S3). Perhaps surprisingly, this change correlated with increased relative expression of PMM1312 in N-starved samples. Furthermore, after 24 hours of N starvation, these antisense TSSs were still evident in the N-replete cells and less prevalent in the N-starved cells, with gene expression still elevated in the N-starved cells (Supplementary Tables S10-S11). In contrast, a similar increase in an antisense TSS was concomitant with a decrease in the gene expression in N-starved cells in the gene PMM1552. This gene, which codes for a small ribosomal subunit protein, demonstrated an increase in the antisense TSS near the beginning of the gene, possibly resulting in the decrease observed in gene expression (Fig. S2). The results after 24 hours N starvation produced a similar result for this gene. Together, these results support the role of RNAs in transcriptional regulation and as a broad cellular response to N conservation.

**Orphan TSSs**

In addition to the internal and antisense TSSs found throughout the MED4 genome, we discovered several orphan TSSs. Often, these TSSs were found in between coding regions, and several times the reads were in the opposite direction of the surrounding genes. It is possible that many code for hypothetical proteins similar to those found in other species or strains of *Prochlorococcus*, such as the unknown peak between PMM0416 and PMM0417 (nucleotide position = 395864) with high identity but low query coverage (Fig. S4). Others could possibly code for sRNAs or other non-coding RNAs such as the peaks between PMM0659 and PMM0660 (nucleotide positions = 627700-628300) Given that the annotation of *Prochlorococcus* MED4 is ever changing, these were likely missed in the original annotation and some may have been identified in newer non-RefSeq verified annotations.

**sRNA verification**

sRNAs in *Prochlorococcus* were previously identified computationally (Axmann *et al.*, 2005), and using microarray expression data (Steglich *et al.*, 2008). Our analysis uncovered TSSs occurring between genes, and we hypothesized that these may be regulatory sRNAs. Of those that were tested by Rfam (Burge *et al.*, 2012), only two had significant hits to previously identified RNAs. One ncRNA, identified as Yfr6, is located between genes PMM0659 and PMM0660. Yfr6 decreased its expression under nitrogen depletion, correlating to expression changes we observed in our cells. We hypothesize that Yfr6 acted as a riboswitch, due to the sequence similarity of its 5’ UTR and the downstream-peptide RNA motif (Weinberg *et al.*, 2010). Furthermore, its resemblance to *glnA* and its high abundance in our N-starved cells (top 10% of counts) likely implies a large influence on regulation gene expression during N starvation (Axmann *et al.*, 2005; Weinberg *et al.*, 2010). The second ncRNA, identified as Yfr7, is located between the genes PMM0683 and PMM0684. Similar to Yfr6, it was highly abundant in our N-starved cells with very large counts, although counts were only increased over the control 12 hours post starvation. We hypothesized based on its similarity to a RNA in the Rfam database that this ncRNA to be an orthologue of γ-proteobacterial 6S RNA, which may repress σ^70^-dependent promoters under nutrient limitation (Trotochaud and Wassarman, 2004; Axmann *et al.*, 2005; Barrick *et al.*, 2005).

**TSS Comparison to Previous Research**

Internal TSSs observed after 12 hours of N starvation were compared to Voigt et al. 2014 to identify any differences during N-starvation (Voigt *et al.*, 2014). After 12 hours of N starvation, there were 220 genes in which we both identified an internal TSS. In contrast, there were 251 genes in which our study found an internal TSS that was not previously identified by Voigt et al (Supplementary Table S12). Voigt et al also found internal TSSs within 373 genes, which were not observed in our study. These differences between data sets are most likely due to a high number of low abundance TSSs, which are sampled differently based on methodological differences and different library preparations for sequencing on separate instruments, in this case 454 vs. Illumina (Thomason *et al.*, 2015; Wade, 2015). Differences in experimental conditions also could play a role in these observed differences.

**References:**

Amman F, Wolfinger MT, Lorenz R, Hofacker IL, Stadler PF, Findeiß S. (2014). TSSAR: TSS annotation regime for dRNA-seq data. *BMC Bioinformatics* **15**: 89.

Axmann IM, Kensche P, Vogel J, Kohl S, Herzel H, Hess WR. (2005). Identification of cyanobacterial non-coding RNAs by comparative genome analysis. *Genome Biol* **6**: R73–R73.

Barrick JE, Sudarsan N, Weinberg Z, Ruzzo WL, Breaker RR. (2005). 6S RNA is a widespread regulator of eubacterial RNA polymerase that resembles an open promoter. *RNA* **11**: 774–784.

Bryant DA, Flores E, Herrero A. (1994). Assimilatory Nitrogen Metabolism and Its Regulation. In: Bryant DA (ed) Advances in Photosynthesis and Respiration Vol. 1. *The Molecular Biology of Cyanobacteria (Advances in Photosynthesis and Respiration)*. Springer Netherlands: Dordrecht, pp 487–517.

Burge SW, Daub J, Eberhardt R, Tate J, Barquist L, Nawrocki EP, *et al.* (2012). Rfam 11.0: 10 years of RNA families. *Nucleic Acids Res* **41**: D226–D232.

Clarke AK. (1999). ATP-dependent Clp Proteases in Photosynthetic Organisms---A Cut Above the Rest! *Ann Bot* **83**: 593–599.

Domain F, Houot L, Chauvat F, Cassier-Chauvat C. (2004). Function and regulation of the cyanobacterial genes lexA, recA and ruvB: LexA is critical to the survival of cells facing inorganic carbon starvation. *Mol Microbiol* **53**: 65–80.

Forchhammer K. (2004). Global carbon/nitrogen control by PII signal transduction in cyanobacteria: from signals to targets. *FEMS Microbiol Rev* **28**: 319–333.

García-Fernández JM, de Marsac NT, Diez J. (2004). Streamlined Regulation and Gene Loss as Adaptive Mechanisms in *Prochlorococcus* for Optimized Nitrogen Utilization in Oligotrophic Environments. *Microbiol Mol Biol Rev* **68**: 630–638.

Georg J, Hess WR. (2011). cis-antisense RNA, another level of gene regulation in bacteria. *Microbiol Mol Biol Rev* **75**: 286–300.

Harano Y, Suzuki I, Maeda S, Kaneko T, Tabata S, Omata T. (1997). Identification and nitrogen regulation of the cyanase gene from the cyanobacteria *Synechocystis* sp. strain PCC 6803 and *Synechococcus* sp. strain PCC 7942. *J Bacteriol* **179**: 5744–5750.

Havaux M, Guedeney G, He Q, Grossman AR. (2003). Elimination of high-light-inducible polypeptides related to eukaryotic chlorophyll a/b-binding proteins results in aberrant photoacclimation in *Synechocystis* PCC6803. *BBA-Bioenergetics* **1557**: 21–33.

Herrero A, Flores E (eds). (2008). The Cyanobacteria: Molecular Biology, Genomics and Evolution. Caister Academic Press.

Hoffmann S, Hoffmann S, Otto C, Otto C, Kurtz S, Kurtz S, *et al.* (2009). Fast Mapping of Short Sequences with Mismatches, Insertions and Deletions Using Index Structures Searls DB (ed). *PLoS Comput Biol* **5**: e1000502.

Hoffmann S, Otto C, Doose G, Tanzer A, Langenberger D, Christ S, *et al.* (2014). A multi-split mapping algorithm for circular RNA, splicing, trans-splicing and fusion detection. *Genome Biol* **15**: 1–11.

Kamennaya NA, Post AF. (2011). Characterization of Cyanate Metabolism in Marine *Synechococcus* and *Prochlorococcus* spp. *Appl Environ Microbiol* **77**: 291–301.

Li H, Handsaker B, Wysoker A, Fennell T, Ruan J, Homer N, *et al.* (2009). The Sequence Alignment/Map format and SAMtools. *Bioinformatics* **25**: 2078–2079.

Marchler-Bauer A, Derbyshire MK, Gonzales NR, Lu S, Chitsaz F, Geer LY, *et al.* (2015). CDD: NCBI's conserved domain database. *Nucleic Acids Res* **43**: D222–D226.

Mattoo AK, Hoffman-Falk H, Marder JB, Edelman M. (1984). Regulation of protein metabolism: Coupling of photosynthetic electron transport to in vivo degradation of the rapidly metabolized 32-kilodalton protein of the chloroplast membranes. *Proc Natl Acad Sci USA* **81**: 1380–1384.

McClure R, Balasubramanian D, Sun Y, Bobrovskyy M, Sumby P, Genco CA, *et al.* (2013). Computational analysis of bacterial RNA-Seq data. *Nucleic Acids Res* **41**: e140–e140.

Meyer RR, Laine PS. (1990). The single-stranded DNA-binding protein of *Escherichia coli*. *Microbiol Rev* **54**: 342–380.

Nakao M, Okamoto S, Kohara M, Fujishiro T, Fujisawa T, Sato S, *et al.* (2010). CyanoBase: the cyanobacteria genome database update 2010. *Nucleic Acids Res* **38**: D379–D381.

Packer L, Kraemer K, Rimbach G. (2001). Molecular aspects of lipoic acid in the prevention of diabetes complications. *Nutrition* **17**: 888–895.

Packer L, Witt EH, Tritschler HJ. (1995). Alpha-lipoic acid as a biological antioxidant. *Free Radical Biol Med* **19**: 227–250.

Palinska KA, Jahns T, Rippka R, Tandeau de Marsac N. (2000). *Prochlorococcus marinus* strain PCC 9511, a picoplanktonic cyanobacterium, synthesizes the smallest urease. *Microbiology* **146**: 3099–3107.

Pelechano V, Steinmetz LM. (2013). Gene regulation by antisense transcription. *Nat Rev Genet* **14**: 880–893.

Reed LJ, DeBusk BG, Gunsalus IC, Hornberger CS. (1951). Crystalline α-Lipoic Acid: A Catalytic Agent Associated with Pyruvate Dehydrogenase. *Science* **114**: 93–94.

Robinson JT, Thorvaldsdottir H, Winckler W, Guttman M, Lander ES, Getz G, *et al.* (2011). Integrative genomics viewer. *Nat Biotech* **29**: 24–26.

Rutherford AW, Faller P. (2003). Photosystem II: evolutionary perspectives. *Philos Trans R So Lond, B, Biol Sci* **358**: 245–253.

Salem K, van Waasbergen LG. (2004). Light Control of hliA Transcription and Transcript Stability in the Cyanobacterium *Synechococcus elongatus* Strain PCC 7942. *J Bacteriol* **186**: 1729–1736.

Sauer J, Schreiber U, Schmid R, Völker U, Forchhammer K. (2001). Nitrogen Starvation-Induced Chlorosis in *Synechococcus* PCC 7942. Low-Level Photosynthesis As a Mechanism of Long-Term Survival. *Plant Physiol* **126**: 233–243.

Snell EE, Strong FM, Peterson WH. (1937). Growth factors for bacteria VI: Fractionation and properties of an accessory factor for lactic acid bacteria. *Biochem J* **31**: 1789–1799.

Steglich C, Futschik ME, Futschik ME, Lindell D, Lindell D, Voss B, *et al.* (2008). The Challenge of Regulation in a Minimal Photoautotroph: Non-Coding RNAs in *Prochlorococcus* Matic I (ed). *PLoS Genet* **4**: e1000173.

Thomason MK, Bischler T, Eisenbart SK, Förstner KU, Zhang A, Herbig A, *et al.* (2015). Global transcriptional start site mapping using differential RNA sequencing reveals novel antisense RNAs in Escherichia coli. *J Bacteriol* **197**: 18–28.

Thorvaldsdóttir H, Robinson JT, Mesirov JP. (2013). Integrative Genomics Viewer (IGV): high-performance genomics data visualization and exploration. *Brief Bioinform* **14**: 178–192.

Tolonen AC, Tolonen AC, Aach J, Aach J, Lindell D, Lindell D, *et al.* (2006). Global gene expression of *Prochlorococcus* ecotypes in response to changes in nitrogen availability. *Mol Syst Biol* **2**: 53.

Trotochaud AE, Wassarman KM. (2004). 6S RNA Function Enhances Long-Term Cell Survival. *J Bacteriol* **186**: 4978–4985.

Vázquez-Bermúdez MF, Paz-Yepes J, Herrero A, Flores E. (2002). The NtcA-activated amt1 gene encodes a permease required for uptake of low concentrations of ammonium in the cyanobacterium *Synechococcus* sp. PCC 7942. *Microbiology* **148**: 861–869.

Voigt K, Sharma CM, Mitschke J, Lambrecht SJ, Voß B, Hess WR, *et al.* (2014). Comparative transcriptomics of two environmentally relevant cyanobacteria reveals unexpected transcriptome diversity. *ISME J* **8**: 2056–2068.

Wade JT. (2015). Where to begin? Mapping transcription start sites genome-wide in Escherichia coli. *J Bacteriol* **197**: 4–6.

Wang Q, Jantaro S, Lu B, Majeed W, Bailey M, He Q. (2008). The High Light-Inducible Polypeptides Stabilize Trimeric Photosystem I Complex under High Light Conditions in *Synechocystis* PCC 6803. *Plant Physiol* **147**: 1239–1250.

Weinberg Z, Wang JX, Bogue J, Yang J, Corbino K, Moy RH, *et al.* (2010). Comparative genomics reveals 104 candidate structured RNAs from bacteria, archaea, and their metagenomes. *Genome Biol* **11**: R31–R31.
