# Supplementary material for: Nitrogen cost minimization is promoted by structural changes in the transcriptome of N-deprived Prochlorococcus cells
Source: ISME J. 2017 Jun 6;11(10):2267–78. doi: 10.1038/ismej.2017.88 (PMC5607370; doi:10.1038/ismej.2017.88)
Supplement: Supplementary Figures [file ismej201788x3.docx]

**Figure S1. Transcriptional mapping for the *Prochlorococcus* Med4 *urt* Operon.** Transcriptomic read coverage is indicated for the *urt* operon. The first black bar represents the *urtA* and *urtB* genes, while second black bar represents *urtC-E*, the remaining genes in the operon.

**Figure S2. Antisense Transcriptional Mapping for *Prochlorococcus* Med4 PMM1552 Gene.** Transcriptomic read coverage is indicated for the PM1552 gene. Experimental (black) and control reads (grey) were compared at 12 hours post N deprivation. The gene is coded on the reverse (lagging) strand, with the coverage map on top representing forward reads, and the coverage on the bottom representing reverse reads. Since this gene is coded on the reverse strand, forward reads are considered antisense. This panel represents the full length of the gene. The x-axis is the distance from the beginning of the gene and proceeds from right to left.

**Figure S3. Antisense transcriptional mapping for the *Prochlorococcus* MED4 gene PMM1312.** Transcriptomic read coverage is indicated for the PM1312 gene. Experimental (black) and control reads (grey) were compared at 12 hours post N deprivation. The gene is coded on the sense strand, with the coverage map on top representing sense reads, and the coverage on the bottom representing antisense reads. This panel represents the full length of the gene. The x-axis is the distance from the beginning of the gene.

**Figure S4. Transcriptional Mapping for *Prochlorococcus* Med4 Unknown Peak.** Transcriptomic read coverage is indicated for an unknown peak between the PMM416 and PMM417 genes. Experimental (red) and control (black) reads were compared at 12 hours post N deprivation. Each panel represents the full length of the gene. The x-axis represents the position in the genome for these genes and unknown peak.
