# Supplementary figures and images for: Nitrogen cost minimization is promoted by structural changes in the transcriptome of N-deprived Prochlorococcus cells
Source: ISME J. 2017 Jun 6;11(10):2267–78. doi: 10.1038/ismej.2017.88 (PMC5607370; doi:10.1038/ismej.2017.88)

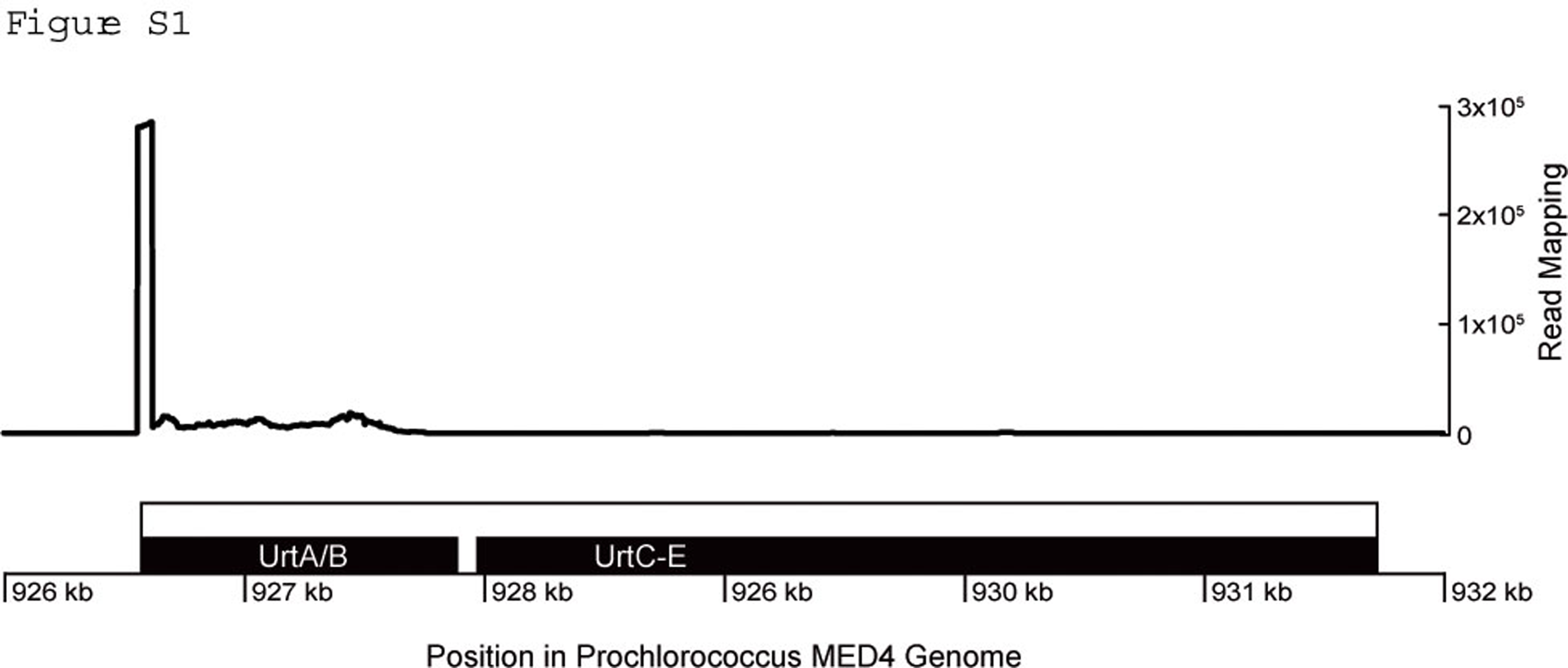

Supplement: Supplementary Figures S1 [file ismej201788x4.tif]

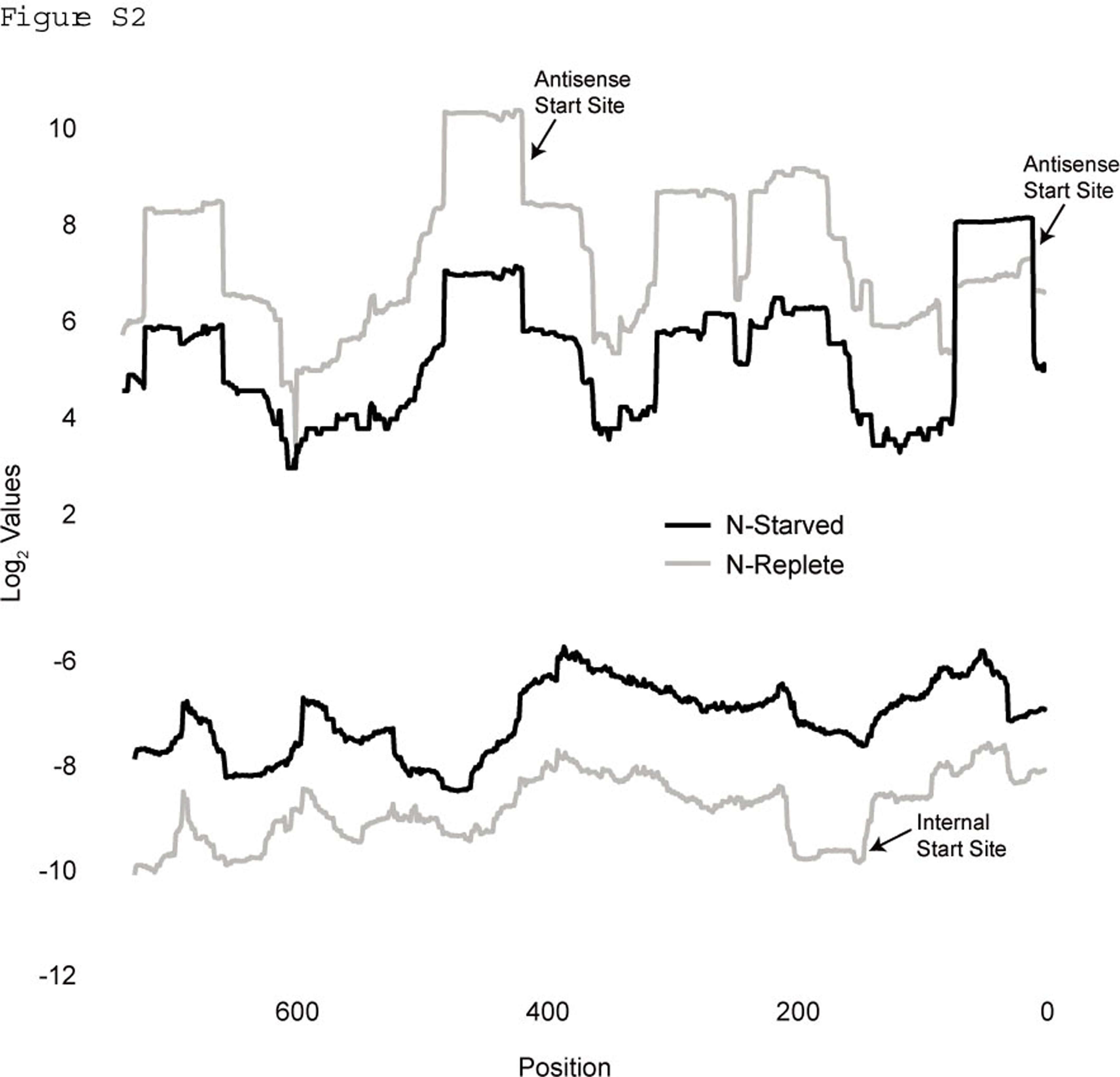

Supplement: Supplementary Figures S2 [file ismej201788x5.tif]

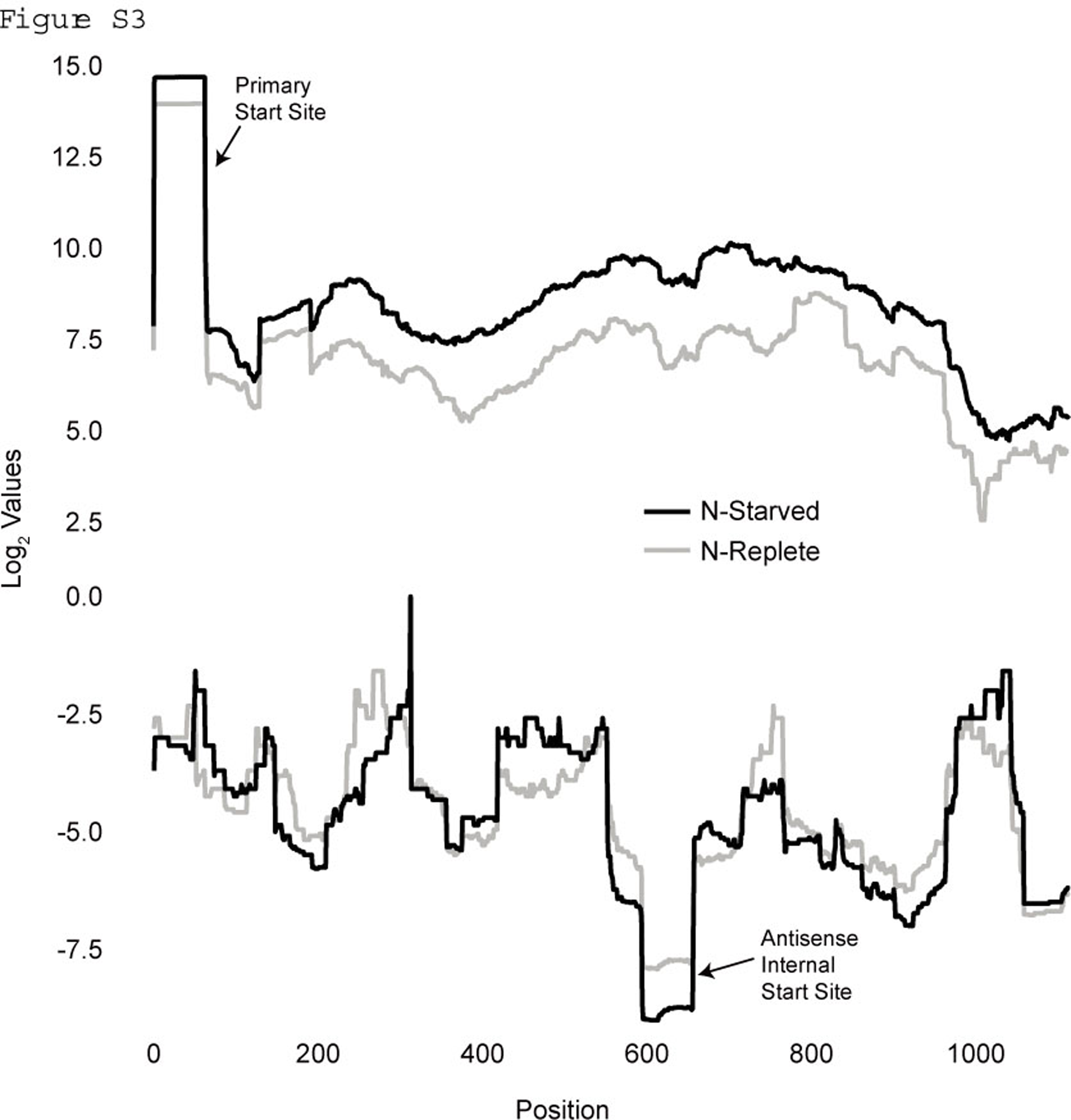

Supplement: Supplementary Figures S3 [file ismej201788x6.tif]

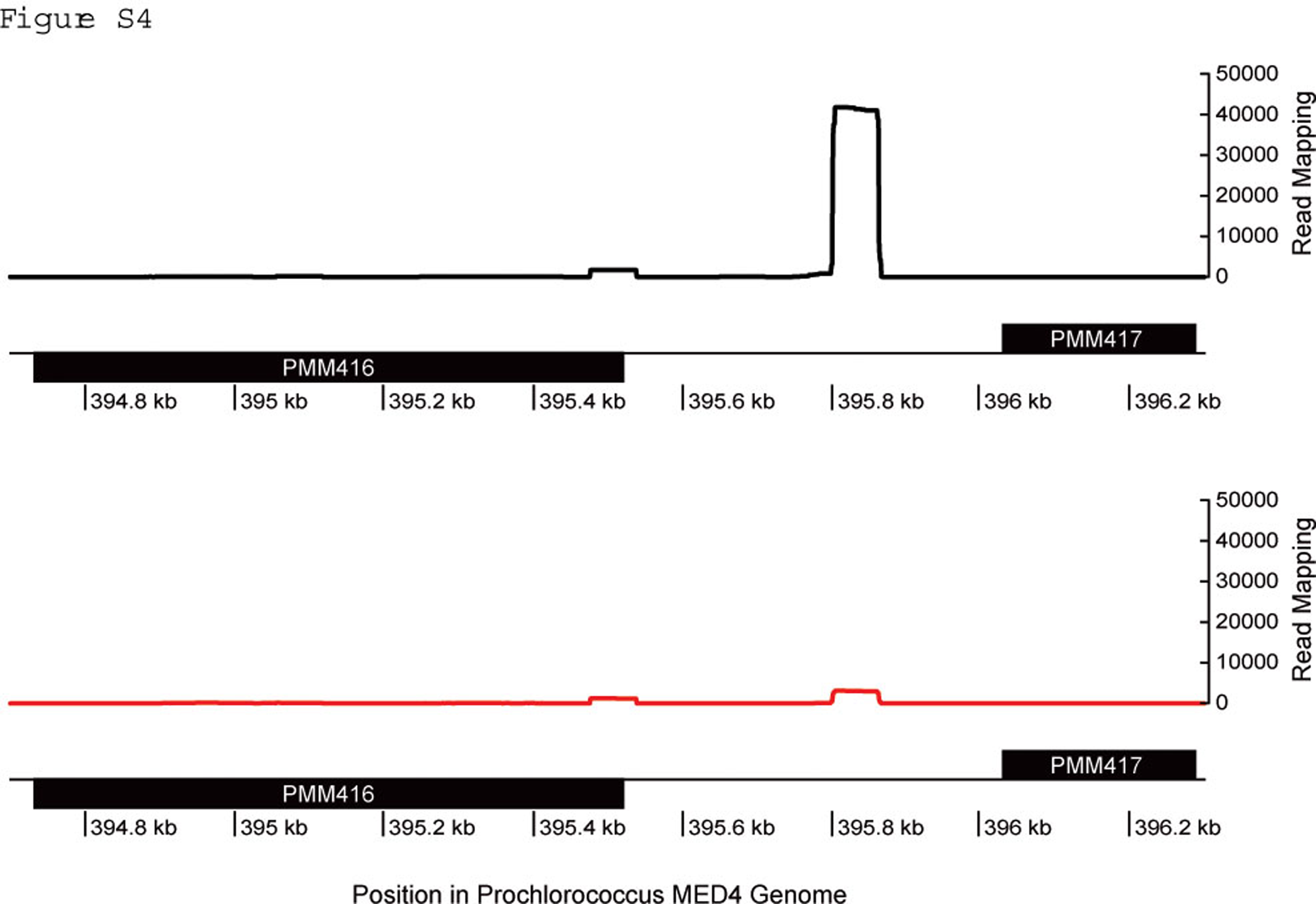

Supplement: Supplementary Figures S4 [file ismej201788x7.tif]
