# Supplementary material for: Nitrogen cost minimization is promoted by structural changes in the transcriptome of N-deprived Prochlorococcus cells
Source: ISME J. 2017 Jun 6;11(10):2267–78. doi: 10.1038/ismej.2017.88 (PMC5607370; doi:10.1038/ismej.2017.88)
Supplement: Supplementary Table 1 [file ismej201788x8.pdf]

Table S1: Raw Sequence Counts and Mean Sequence Counts per Read

Time (hrs)	Treatment	Original Sequences	Average Raw Counts
0	Control	35531407	18071
0	Control	26906967	13166
0	Experimental	27298646	13755
0	Experimental	21399153	10406
3	Control	19799879	6903
3	Control	13485385	7221
3	Experimental	20069084	10131
3	Experimental	14978176	9651
12	Control	19731389	9963
12	Control	24588668	12342
12	Experimental	27804712	13699
12	Experimental	34846401	17186
24	Control	24719832	11746
24	Control	24060503	11173
24	Experimental	23942075	12505
24	Experimental	33349917	16182
