# Supplementary material for: Nitrogen cost minimization is promoted by structural changes in the transcriptome of N-deprived Prochlorococcus cells
Source: ISME J. 2017 Jun 6;11(10):2267–78. doi: 10.1038/ismej.2017.88 (PMC5607370; doi:10.1038/ismej.2017.88)
Supplement: Supplementary Table 2 [file ismej201788x9.pdf]

Table S2. Correlation of expression values between two datasets -- this study and Tolonen et al. (2006).

		3 Hours Post Starvation (This Study)	12 Hours Post Starvation (This Study)	24 Hours Post Starvation (This Study)
All Data	3 Hours Post Starvation (Tolonen)	0.007	0.318	0.329
	12 Hours Post Starvation (Tolonen)	0.028	0.553	0.538
	24 Hours Post Starvation (Tolonen)	0.016	0.589	0.584
Top 50% Values	3 Hours Post Starvation (Tolonen)	-0.004	0.377	0.408
	12 Hours Post Starvation (Tolonen)	-0.004	0.647	0.638
	24 Hours Post Starvation (Tolonen)	-0.008	0.690	0.723
