# Supplementary material for: Nitrogen cost minimization is promoted by structural changes in the transcriptome of N-deprived Prochlorococcus cells
Source: ISME J. 2017 Jun 6;11(10):2267–78. doi: 10.1038/ismej.2017.88 (PMC5607370; doi:10.1038/ismej.2017.88)
Supplement: Supplementary Table 5 [file ismej201788x12.pdf]

Table S5. Gene Expression For Genes with Top 50% Expression Values during 24 Hours Post Starvation

Name	Number	log2 Fold Change	Standard Deviation	Standard Error	p-value	Category	Definition
PMM0001	2	0.87	1.35	0.96	0.49	DNA replication, recombination, and repair	DNA polymerase III, beta chain
PMM0008	2	-0.11	0.05	0.03	0.62	Conserved hypothetical protein	conserved hypothetical protein
PMM0013	2	-1.40	0.93	0.65	0.27	Fatty acid, phospholipid and sterol metabolism	RNA-binding region RNP-1 (RNA recognition motif)
PMM0015	2	0.03	0.48	0.34	0.93	Conserved hypothetical protein	Domain of unknown function DUF25
PMM0016	2	-0.88	1.81	1.28	0.08	Chaperones	Heat shock protein GrpE
PMM0017	2	1.37	0.02	0.01	0.11	Chaperones	DnaJ protein
PMM0020	2	0.87	0.05	0.03	0.46	Conserved hypothetical protein	conserved hypothetical protein
PMM0023	2	-0.62	1.18	0.84	0.28	CO2 fixation	Glyceraldehyde 3-phosphate dehydrogenase(NADP+)(phosphorylating)
PMM0025	2	-0.59	0.21	0.15	0.40	Protein modification and translation factors	Cyclophilin-type peptidyl-prolyl cis-trans isomerase
PMM0026	2	-1.32	0.39	0.28	0.03	Protein modification and translation factors	Elongation factor P (EF-P)
PMM0027	2	1.21	0.07	0.05	0.19	Fatty acid, phospholipid and sterol metabolism	Biotin / Lipoyl attachment:Acetyl-CoA biotin carboxyl carrier...
PMM0030	2	4.93	0.95	0.67	0.00	Protein modification and translation factors	possible Transcription factor TFIID (or TATA-b
PMM0031	2	1.13	0.35	0.25	0.33	DNA replication, recombination, and repair	HNH endonuclease:HNH nuclease
PMM0032	2	1.07	0.68	0.48	0.46	Protein and peptide secretion	possible Bacterial type II secretion system pr
PMM0033	2	1.11	0.46	0.33	1.00	Conserved hypothetical protein	conserved hypothetical protein
PMM0034	2	-0.22	0.17	0.12	0.57	Conserved hypothetical protein	conserved hypothetical protein
PMM0035	2	1.83	0.09	0.06	0.02	Hydrogenase	soluble hydrogenase small subunit
PMM0037	2	1.56	0.33	0.24	0.10	Purine ribonucleotide biosynthesis	Glutamine amidotransferase class-I:GMP synthase
PMM0043	2	-0.25	0.69	0.48	0.53	Other	flavoprotein
PMM0046	2	-2.59	0.10	0.07	0.00	Interconversions and salvage of nucleosides and nucleotides	Nucleoside diphosphate kinase
PMM0048	2	-0.01	0.46	0.33	0.66	Aminoacyl tRNA synthetases and tRNA modification	Glutamyl-tRNA (Gln) amidotransferase subunit B
PMM0051	2	1.13	1.34	0.95	0.19	Conserved hypothetical protein	conserved hypothetical protein
PMM0055	2	0.36	0.11	0.08	1.00	Conserved hypothetical protein	conserved hypothetical protein
PMM0056	2	-0.44	1.67	1.18	0.41	Conserved hypothetical protein	conserved hypothetical protein
PMM0058	2	2.89	0.30	0.21	0.00	Conserved hypothetical protein	conserved hypothetical protein
PMM0059	2	0.92	1.09	0.77	0.56	Conserved hypothetical protein	conserved hypothetical protein
PMM0060	2	-0.44	0.14	0.10	0.45	Fatty acid, phospholipid and sterol metabolism	acetyl-CoA carboxylase, biotin carboxylase subunit
PMM0061	2	-0.58	1.63	1.15	0.22	Other	YGGT family, conserved hypothetical integral membrane protein
PMM0062	2	-2.24	0.44	0.31	0.00	Photosystem II	photosystem II PsbX protein
PMM0063	2	1.85	0.17	0.12	0.19	Conserved hypothetical protein	conserved hypothetical protein
PMM0064	2	0.42	0.90	0.63	0.71	Adaptations and atypical conditions	possible high light inducible protein
PMM0068	2	1.42	1.24	0.88	0.24	Regulatory functions	putative formylmethionine deformylase
PMM0073	2	0.01	0.61	0.43	0.63	Transport and binding proteins	ABC transporter, membrane component
PMM0075	2	2.41	1.68	1.19	0.00	Conserved hypothetical protein	conserved hypothetical protein
PMM0078	2	-0.79	0.32	0.22	0.23	Other	possible 4'-phosphopantetheinyl transferase family protein
PMM0079	2	-2.29	0.49	0.35	0.00	Transport and binding proteins	putative bacterioferritin comigratory (BCP) protein
PMM0081	2	-0.55	1.18	0.83	0.38	Serine family / Sulfur assimilation	Phosphoadenosine phosphosulfate reductase
PMM0082	2	1.31	0.23	0.17	0.15	Regulatory functions	putative NADH dehydrogenase, transport associated
PMM0083	2	-0.68	1.16	0.82	0.26	Regulatory functions	putative sodium/sulfate transporter, DASS family
PMM0085	2	0.23	0.19	0.14	1.00	Regulatory functions	putative potassium channel, VIC family
PMM0086	2	0.22	0.13	0.09	1.00	Conserved hypothetical protein	Conserved hypothetical protein
PMM0087	2	3.49	1.13	0.80	0.00	Conserved hypothetical protein	conserved hypothetical protein
PMM0088	2	1.45	0.66	0.47	0.15	Conserved hypothetical protein	conserved hypothetical protein
PMM0091	2	0.64	0.24	0.17	1.00	Conserved hypothetical protein	conserved hypothetical protein
PMM0092	2	2.56	0.44	0.31	0.00	Conserved hypothetical protein	conserved hypothetical protein
PMM0093	2	-0.41	0.61	0.43	0.82	Adaptations and atypical conditions	possible high light inducible protein
PMM0095	2	-0.52	1.28	0.90	0.34	Other	similar to serum resistance locus BrkB
PMM0099	2	0.85	0.02	0.02	0.38	Conserved hypothetical protein	conserved hypothetical protein
PMM0101	2	1.35	0.34	0.24	0.14	Conserved hypothetical protein	conserved hypothetical protein
PMM0103	2	-0.37	1.21	0.86	0.71	Conserved hypothetical protein	conserved hypothetical protein
PMM0105	2	1.50	0.83	0.59	0.12	Riboflavin	RibD/ribG C-terminal domain
PMM0106	2	0.75	0.60	0.42	0.91	Other	6-pyruvoyl tetrahydropterin synthase
PMM0111	2	0.72	0.32	0.22	0.47	Conserved hypothetical protein	conserved hypothetical protein
PMM0114	2	1.13	0.16	0.12	0.23	Conserved hypothetical protein	conserved hypothetical protein
PMM0115	2	1.17	0.19	0.13	0.41	Carotenoid	zeta-carotene desaturase
PMM0116	2	0.73	0.08	0.05	0.92	Conserved hypothetical protein	conserved hypothetical protein
PMM0117	2	2.49	0.50	0.35	0.00	Conserved hypothetical protein	conserved hypothetical protein
PMM0120	2	1.65	0.06	0.04	0.05	Cell division	putative cell division inhibitor
PMM0121	2	0.91	1.38	0.98	0.59	Conserved hypothetical protein	conserved hypothetical protein
PMM0123	2	1.40	0.32	0.22	0.14	Serine family / Sulfur assimilation	O-acetylserine (thiol)-lyase A
PMM0124	2	-0.10	0.70	0.50	0.55	Conserved hypothetical protein	conserved hypothetical protein in cyanobacteria
PMM0125	2	-0.53	0.49	0.35	0.40	Transport and binding proteins	possible ABC transporter, ATP-binding component
PMM0126	2	0.06	0.02	0.02	0.77	Other	possible Herpesvirus UL6 like
PMM0128	2	0.39	0.80	0.57	1.00	Regulatory functions	two-component response regulator
PMM0132	2	-1.14	0.82	0.58	0.18	Other	cyanobacterial conserved hypothetical
PMM0133	2	2.06	0.28	0.20	0.00	Radiation sensitivity	putative DNA repair protein RadA
PMM0134	2	-0.75	1.11	0.79	0.31	Regulatory functions	two-component response regulator
PMM0136	2	-1.67	0.95	0.67	0.02	Fatty acid, phospholipid and sterol metabolism	3-oxoacyl-[acyl-carrier-protein] synthase III
PMM0137	2	-0.48	0.43	0.31	0.48	Fatty acid, phospholipid and sterol metabolism	Malonyl coenzyme A-acyl carrier protein transacylase
PMM0138	2	0.47	0.10	0.07	0.87	Fatty acid, phospholipid and sterol metabolism	putative 1-acyl-sn-glycerol-3-phosphate acyltransferase
PMM0139	2	-1.04	0.34	0.24	0.22	Conserved hypothetical protein	conserved hypothetical protein
PMM0140	2	1.28	2.88	2.04	1.00	Regulatory functions	putative Ycf34
PMM0142	2	-1.75	1.28	0.90	0.00	Fatty acid, phospholipid and sterol metabolism	RNA-binding region RNP-1 (RNA recognition motif)
PMM0143	2	1.05	0.30	0.21	0.46	Carotenoid	Squalene and phytoene synthases
PMM0144	2	2.34	0.62	0.44	0.00	Carotenoid	phytoene desaturase
PMM0145	2	1.97	0.05	0.03	0.01	Conserved hypothetical protein	conserved hypothetical protein
PMM0146	2	2.10	0.49	0.34	0.01	Conserved hypothetical protein	conserved hypothetical protein
PMM0147	2	-0.34	0.00	0.00	0.50	Regulatory functions	putative Rubisco transcriptional regulator
PMM0149	2	1.39	0.32	0.22	0.21	NADH dehydrogenase	putative NADH Dehydrogenase (complex I) subunit (chain 5)
PMM0150	2	1.39	0.04	0.03	0.20	NADH dehydrogenase	putative NADH dehydrogenase subunit (chain 4)
PMM0153	2	-0.81	0.63	0.45	0.22	Conserved hypothetical protein	conserved hypothetical protein
PMM0154	2	-1.11	0.55	0.39	0.13	Regulatory functions	Bacterial regulatory protein, LuxR family
PMM0159	2	0.38	0.09	0.06	0.75	NADH dehydrogenase	putative NADH Dehydrogenase subunit
PMM0160	2	3.11	0.90	0.63	0.00	NADH dehydrogenase	putative respiratory-chain NADH dehydrogenase subunit
PMM0163	2	-2.03	0.28	0.20	0.00	Conserved hypothetical protein	conserved hypothetical protein
PMM0164	2	1.36	0.16	0.12	0.20	Aromatic amino acid family	Tryptophan synthase, beta chain:Pyridoxal-5'-phosphate-depend...
PMM0166	2	-2.62	1.18	0.84	0.00	Serine family / Sulfur assimilation	Adenylylsulfate kinase
PMM0172	2	1.45	0.82	0.58	0.07	NADH dehydrogenase	putative NADH dehydrogenase subunit
PMM0179	2	1.33	1.71	1.21	0.30	Other	Glutaredoxin
PMM0180	2	0.37	0.05	0.04	1.00	Protein modification and translation factors	peptide chain release factor RF-2
PMM0184	2	1.10	0.25	0.18	0.20	Aromatic amino acid family	para-aminobenzoate synthase component II
PMM0195	2	0.12	0.34	0.24	0.71	Other	Phosphoglycerate kinase
PMM0200	2	-1.01	0.22	0.16	0.34	Degradation of RNA	possible ribonuclease HI
PMM0201	2	-2.30	0.06	0.04	0.00	Ribosomal proteins	50S ribosomal protein L7/L12
PMM0202	2	-2.46	0.35	0.25	0.00	Ribosomal proteins	50S ribosomal protein L10
PMM0203	2	-1.98	0.45	0.32	0.00	Ribosomal proteins	50S ribosomal protein L1
PMM0204	2	-1.41	0.33	0.23	0.00	Ribosomal proteins	50S ribosomal protein L11
PMM0205	2	1.66	0.82	0.58	0.12	RNA synthesis, modification, and DNA transcription	transcription antitermination protein, NusG
PMM0206	2	-1.37	1.04	0.74	0.03	Protein and peptide secretion	putative preprotein translocase, SecE subunit
PMM0208	2	-0.15	0.15	0.11	0.60	Other	Enolase
PMM0209	2	-0.68	0.32	0.23	0.38	Other	possible kinase
PMM0211	2	-0.50	0.57	0.40	0.47	Other	FAD-dependent pyridine nucleotide-disulphide oxidoreductase
PMM0212	2	3.18	1.65	1.17	0.00	Conserved hypothetical protein	conserved hypothetical protein
PMM0213	2	1.40	1.07	0.75	0.19	Transport and binding proteins	putative sodium-dependent bicarbonate transporter
PMM0214	2	-2.54	0.92	0.65	0.00	Regulatory functions	putative sulfate transporter
PMM0216	2	-1.52	0.09	0.06	0.22	Adaptations and atypical conditions	Glyoxalase/Bleomycin resistance protein/Dioxygenase superfamily
PMM0218	2	-0.05	0.56	0.40	0.50	Other	GTP1/OBG family
PMM0219	2	-0.37	1.21	0.86	0.57	Conserved hypothetical protein	conserved hypothetical
PMM0220	2	1.67	0.70	0.49	1.00	Other	No Cyanobase Name
PMM0223	2	2.47	0.37	0.26	0.00	Photosystem II	Photosystem II PsbA protein (D1)
PMM0224	2	0.43	0.95	0.67	1.00	Aromatic amino acid family	Chorismate synthase
PMM0226	2	0.18	0.13	0.09	0.77	Cell division	cell division protein FtsH2
PMM0228	2	-0.73	0.83	0.58	0.14	Photosystem II	Photosystem II manganese-stabilizing protein
PMM0231	2	-2.76	0.40	0.28	0.00	Conserved hypothetical protein	conserved hypothetical protein
PMM0235	2	-0.52	1.04	0.73	0.72	Aminoacyl tRNA synthetases and tRNA modification	Glutamyl-tRNA(Gln) amidotransferase subunit C
PMM0237	2	-0.21	0.06	0.04	0.75	Conserved hypothetical protein	conserved hypothetical protein

PMM0239	2	-1.69	0.52	0.36	0.00	Conserved hypothetical protein	conserved hypothetical protein
PMM0242	2	1.14	0.66	0.46	0.20	Thiamine	thioredoxin-like protein TxlA
PMM0243	2	0.16	0.59	0.42	1.00	Pyrimidine ribonucleotide biosynthesis	possible Thy1 protein homolog
PMM0244	2	0.06	0.22	0.15	0.60	Purine ribonucleotide biosynthesis	dCTP Deaminase
PMM0245	2	1.58	0.40	0.28	0.04	Cobalamin, heme, phycobilin and porphyrin	cob(I)alamin adenosyltransferase
PMM0246	2	2.17	1.70	1.20	0.00	Transport and binding proteins	Global nitrogen regulatory protein, CRP family of transcriptional regulators
PMM0251	2	-2.79	0.26	0.18	0.00	Photosystem II	Photosystem II PsbH protein
PMM0252	2	2.28	0.23	0.16	0.00	Photosystem II	Photosystem II reaction centre N protein (psbN)
PMM0253	2	0.28	0.54	0.38	1.00	Photosystem II	photosystem II reaction center PsbI protein
PMM0254	2	-0.13	0.11	0.08	0.61	Conserved hypothetical protein	conserved hypothetical protein
PMM0258	2	1.81	0.56	0.39	0.02	Other	Serine hydroxymethyltransferase (SHMT)
PMM0259	2	0.28	0.69	0.49	1.00	Conserved hypothetical protein	conserved hypothetical protein
PMM0260	2	1.27	2.31	1.64	0.40	Conserved hypothetical protein	conserved hypothetical protein
PMM0263	2	2.26	0.23	0.16	1.00	Transport and binding proteins	Ammonium transporter family
PMM0264	2	-1.32	0.69	0.49	0.09	Murein sacculus and peptidoglycan	LytB protein homolog
PMM0265	2	-1.03	0.56	0.40	0.19	Conserved hypothetical protein	conserved hypothetical protein
PMM0267	2	-1.67	0.16	0.12	0.06	Other	probable esterase
PMM0268	2	0.06	0.12	0.09	0.88	Conserved hypothetical protein	conserved hypothetical protein
PMM0272	2	-4.18	1.09	0.77	0.00	Photosystem II	Photosystem II protein PsbK
PMM0273	2	-1.10	0.50	0.35	0.14	Other	probable oxidoreductase
PMM0275	2	-1.17	0.96	0.68	0.04	Pyrimidine ribonucleotide biosynthesis	Orotate phosphoribosyltransferase
PMM0278	2	0.03	0.10	0.07	0.91	Other	Phosphotransferase superclass
PMM0279	2	-0.79	1.38	0.98	0.19	Conserved hypothetical protein	conserved hypothetical protein
PMM0282	2	0.41	1.50	1.06	0.70	Fatty acid, phospholipid and sterol metabolism	enoyl-[acyl-carrier-protein] reductase
PMM0284	2	-1.39	0.24	0.17	0.05	Regulatory functions	putative pleiotropic regulatory protein
PMM0286	2	-1.23	0.26	0.18	0.13	Other	NUDIX hydrolase
PMM0289	2	1.24	0.13	0.10	0.21	Transport and binding proteins	possible ABC transporter
PMM0290	2	1.16	0.33	0.23	0.33	Transport and binding proteins	possible ABC transporter, ATP binding component
PMM0293	2	0.82	0.24	0.17	0.50	NADH dehydrogenase	putative respiratory-chain NADH dehydrogenase subunit
PMM0294	2	1.32	0.75	0.53	0.27	NADH dehydrogenase	putative NADH Dehydrogenase (complex I) subunit (chain 3)
PMM0295	2	-0.84	0.43	0.30	0.21	Other	probable rubredoxin
PMM0296	2	-1.56	0.42	0.30	0.01	Conserved hypothetical protein	conserved hypothetical protein
PMM0297	2	-0.52	0.26	0.18	0.86	Photosystem II	Cytochrome b559 alpha-subunit
PMM0298	2	-0.69	0.26	0.19	0.87	Photosystem II	Cytochrome b559 beta-subunit
PMM0299	2	-1.15	0.23	0.16	0.06	Photosystem II	photosystem II PsbL protein
PMM0300	2	-1.16	0.01	0.01	0.16	Photosystem II	photosytem II PsbJ protein
PMM0301	2	1.82	0.08	0.06	0.04	Other	5'-methylthioadenosine phosphorylase
PMM0305	2	-2.06	1.49	1.05	0.03	Phycobilisome	Phycobilisome protein
PMM0306	2	-3.80	1.16	0.82	0.00	Phycobilisome	phycoerythrin linker protein CpeS homolog
PMM0307	2	-3.44	0.68	0.48	0.00	Conserved hypothetical protein	hypothetical
PMM0308	2	0.95	0.70	0.49	0.51	Conserved hypothetical protein	conserved hypothetical protein
PMM0309	2	0.04	0.54	0.38	1.00	Other	possible Pollen allergen
PMM0311	2	-1.65	0.74	0.53	0.01	Aspartate family	S-adenosylmethionine synthetase
PMM0312	2	-1.75	0.43	0.31	0.00	Ribosomal proteins	30S ribosomal protein S1, homolog A
PMM0313	2	-0.59	0.47	0.33	0.55	Conserved hypothetical protein	conserved hypothetical protein
PMM0314	2	-1.53	0.40	0.28	0.02	Photosystem II	Photosystem II PsbT protein
PMM0315	2	-1.10	0.36	0.26	0.95	Photosystem II	Photosystem II PsbB protein (CP47)
PMM0316	2	1.19	0.76	0.53	0.37	Soluble electron carriers	possible ferredoxin
PMM0317	2	-1.93	0.78	0.55	0.00	Photosystem II	possible Photosystem II reaction center M protein (PsbM)
PMM0318	2	-1.55	1.18	0.84	0.00	Cobalamin, heme, phycobilin and porphyrin	putative protein methyltransferase
PMM0321	2	-0.27	0.33	0.23	0.51	Cell division	putative septum site-determining protein MinD
PMM0324	2	-0.54	1.55	1.09	0.41	Detoxification	PDZ domain (also known as DHR or GLGF):Tail specific protease...
PMM0325	2	-0.82	0.58	0.41	1.00	Cytochrome b6/f complex	Cytochrome b6
PMM0326	2	0.37	0.11	0.08	1.00	Cytochrome b6/f complex	PetD protein (subunit IV of the Cytochrome b6f complex)
PMM0327	2	0.11	0.05	0.04	0.72	Other	putative neutral invertase-like protein
PMM0328	2	-1.81	0.46	0.33	0.00	DNA replication, recombination, and repair	Formamidopyrimidine-DNA glycolase (FAPY-DNA glycolase)
PMM0329	2	-2.13	0.33	0.23	0.00	Photosystem I	Photosystem I PsaE protein (subunit IV)
PMM0333	2	1.01	0.96	0.68	0.34	Other	GCN5-related N-acetyltransferase
PMM0334	2	2.84	1.79	1.27	0.00	Conserved hypothetical protein	conserved hypothetical protein
PMM0336	2	1.33	0.38	0.27	0.27	Conserved hypothetical protein	conserved hypothetical
PMM0337	2	8.45	0.19	0.14	0.00	Conserved hypothetical protein	conserved hypothetical protein
PMM0338	2	0.55	0.29	0.20	0.89	Conserved hypothetical protein	conserved hypothetical
PMM0339	2	0.41	0.70	0.50	1.00	Carotenoid	Bacterial-type phytoene dehydrogenase
PMM0341	2	1.30	0.16	0.11	0.19	Conserved hypothetical protein	conserved hypothetical
PMM0342	2	1.21	0.05	0.04	0.36	Other	possible Helper component proteinase
PMM0343	2	0.98	1.21	0.85	0.68	Other	mttA/Hcf106 family
PMM0345	2	0.19	0.03	0.02	0.80	Transport and binding proteins	putative bacterioferritin comigratory protein
PMM0346	2	-6.53	2.46	1.74	0.00	Conserved hypothetical protein	conserved hypothetical protein
PMM0347	2	0.09	0.22	0.16	0.58	Conserved hypothetical protein	conserved hypothetical
PMM0348	2	-0.77	1.08	0.77	0.97	Other	possible Spectrin repeat
PMM0350	2	-1.37	1.82	1.29	0.57	Regulatory functions	possible TIR domain
PMM0351	2	1.24	1.76	1.24	0.33	Cobalamin, heme, phycobilin and porphyrin	possible Small, acid-soluble spore proteins, a
PMM0356	2	-2.27	0.86	0.61	0.00	Fatty acid, phospholipid and sterol metabolism	Alpha/beta hydrolase fold:Esterase/lipase/thioesterase family...
PMM0363	2	1.66	0.48	0.34	0.04	Regulatory functions	possible MarR family
PMM0364	2	3.47	0.67	0.47	0.00	Other	possible Malic enzyme
PMM0365	2	4.11	0.31	0.22	0.00	Other	possible DsrE-like protein
PMM0366	2	1.49	0.35	0.25	0.10	Transport and binding proteins	Type-1 copper (blue) domain
PMM0367	2	1.12	1.06	0.75	0.61	Conserved hypothetical protein	conserved hypothetical protein
PMM0368	2	2.58	0.42	0.30	0.00	Conserved hypothetical protein	conserved hypothetical protein
PMM0370	2	3.84	0.29	0.21	0.00	Transport and binding proteins	putative cyanate ABC transporter, substrate binding protein
PMM0371	2	2.65	0.70	0.50	0.00	Transport and binding proteins	putative cyanate ABC transporter
PMM0373	2	-0.37	0.47	0.33	0.64	Other	Cyanate lyase
PMM0374	2	2.23	0.50	0.35	0.00	Other	mttA/Hcf106 family
PMM0377	2	0.90	0.55	0.39	0.37	Conserved hypothetical protein	hypothetical
PMM0378	2	1.51	0.69	0.49	0.97	Conserved hypothetical protein	conserved hypothetical protein
PMM0379	2	-0.32	1.28	0.90	0.48	Conserved hypothetical protein	hypothetical
PMM0383	2	2.20	0.59	0.42	0.01	Transport and binding proteins	probable periplasmic protein
PMM0388	2	1.84	1.26	0.89	0.02	Chemotaxis	putative similar to tRNA-(MS[2]IO[6]A)-hydroxylase
PMM0395	2	1.96	0.35	0.25	0.08	Conserved hypothetical protein	conserved hypothetical protein
PMM0400	2	-1.52	1.82	1.29	0.16	Adaptations and atypical conditions	light repressed protein A homolog
PMM0403	2	-0.16	1.03	0.73	0.43	Conserved hypothetical protein	conserved hypothetical protein
PMM0405	2	-1.43	0.12	0.09	0.03	Transport and binding proteins	Dihydrolipoamide acetyltransferase component (E2) of pyruvate de
PMM0407	2	0.06	0.54	0.38	0.84	Serine family / Sulfur assimilation	O-acetylserine (thiol)-lyase A
PMM0410	2	-1.68	0.68	0.48	0.00	Ribosomal proteins	30S ribosomal protein S4
PMM0411	2	0.80	0.67	0.48	0.60	Conserved hypothetical protein	conserved hypothetical protein
PMM0412	2	1.77	0.55	0.39	0.03	Conserved hypothetical protein	conserved hypothetical protein
PMM0416	2	-3.02	0.73	0.51	0.00	RNA synthesis, modification, and DNA transcription	SAM (and some other nucleotide) binding motif:Generic methyl-...
PMM0417	2	-1.53	0.04	0.03	0.07	Conserved hypothetical protein	hypothetical
PMM0418	2	-0.21	1.49	1.06	1.00	Other	NifU-like protein
PMM0428	2	-1.57	0.55	0.39	0.02	Cobalamin, heme, phycobilin and porphyrin	chlorophyll synthase 33 kD subunit
PMM0429	2	-3.39	0.09	0.06	0.00	Conserved hypothetical protein	conserved hypothetical protein
PMM0435	2	-0.53	0.31	0.22	0.31	NADH dehydrogenase	putative NADH dehydrogenase (complex I) subunit (chain 2)
PMM0436	2	0.67	0.91	0.64	0.92	DNA replication, recombination, and repair	Prokaryotic DNA topoisomerase
PMM0441	2	1.26	0.09	0.06	0.19	Other	Aldo/keto reductase family
PMM0443	2	1.15	0.04	0.03	0.20	Conserved hypothetical protein	conserved hypothetical protein
PMM0445	2	0.95	0.38	0.27	1.00	Respiratory terminal oxidases	Cytochrome c oxidase, subunit I
PMM0446	2	2.06	0.47	0.33	0.01	Respiratory terminal oxidases	putative cytochrome c oxidase, subunit 2
PMM0447	2	3.76	0.63	0.45	0.00	Conserved hypothetical protein	conserved hypothetical protein
PMM0448	2	0.16	0.38	0.27	0.99	Cobalamin, heme, phycobilin and porphyrin	putative protoheme IX farnesyltransferase
PMM0451	2	0.97	0.68	0.48	0.21	Other	possible Arenavirus glycoprotein
PMM0452	2	-2.30	0.86	0.61	0.00	Chaperones	GroEL2 protein (Chaperonin cpn60 2)
PMM0453	2	-4.63	1.51	1.07	0.00	Fatty acid, phospholipid and sterol metabolism	3-oxoacyl-[acyl-carrier protein] reductase
PMM0461	2	-0.34	0.09	0.06	0.50	Cytochrome b6/f complex	Cytochrome f
PMM0462	2	-1.36	0.04	0.03	0.03	Cytochrome b6/f complex	Rieske iron-sulfur protein
PMM0465	2	-0.92	1.16	0.82	0.20	Conserved hypothetical protein	hypothetical
PMM0468	2	-2.93	0.32	0.23	0.00	Photosystem I	Photosystem I PsaJ protein (subunit IX)
PMM0469	2	-3.27	0.30	0.21	0.00	Photosystem I	Photosystem I PsaF protein (subunit III)
PMM0470	2	-0.75	0.57	0.40	0.27	Other	probable o-sialoglycoprotein endopeptidase

PMM0471	2	-2.22	1.78	1.26	0.00	Adaptations and atypical conditions	possible high light inducible protein
PMM0472	2	0.88	0.81	0.58	0.85	Transport and binding proteins	putative Na+/H+ antiporter, CPA1 family
PMM0473	2	-0.85	0.77	0.54	0.36	Aminoacyl tRNA synthetases and tRNA modification	Glutamyl-tRNA synthetase
PMM0474	2	-0.68	0.71	0.50	0.95	Conserved hypothetical protein	Conserved hypothetical protein
PMM0475	2	-1.32	0.44	0.31	0.09	Ribosomal proteins	Ribosomal protein L19
PMM0476	2	0.32	0.34	0.24	1.00	Conserved hypothetical protein	conserved hypothetical protein
PMM0477	2	-0.74	0.17	0.12	0.87	Protein modification and translation factors	putative methionine aminopeptidase
PMM0478	2	#NAME?	NA	NA	0.00	Conserved hypothetical protein	conserved hypothetical protein
PMM0479	2	-0.75	0.52	0.36	0.31	Conserved hypothetical protein	conserved hypothetical protein
PMM0480	2	1.61	0.06	0.05	0.04	Conserved hypothetical protein	conserved hypothetical protein
PMM0481	2	-1.18	0.18	0.13	0.14	Conserved hypothetical protein	conserved hypothetical protein
PMM0482	2	0.78	0.53	0.37	1.00	Other	Band 7 protein
PMM0483	2	-0.08	0.22	0.16	0.76	Cobalamin, heme, phycobilin and porphyrin	glutamate-1-semialdehyde 2,1-aminomutase
PMM0485	2	0.96	1.36	0.96	0.94	Conserved hypothetical protein	conserved hypothetical protein
PMM0491	2	1.95	0.25	0.18	0.04	Other	4a-hydroxytetrahydrobiopterin dehydratase (PCD)
PMM0492	2	-1.14	0.48	0.34	0.15	Conserved hypothetical protein	conserved hypothetical protein
PMM0493	2	-1.04	1.47	1.04	0.03	Other	Carboxypeptidase Taq (M32) metallopeptidase
PMM0494	2	-1.69	1.94	1.37	0.03	Phosphorus compounds	putative inorganic pyrophosphatase
PMM0495	2	0.78	0.48	0.34	0.60	Cobalamin, heme, phycobilin and porphyrin	Porphobilinogen deaminase
PMM0496	2	0.80	0.96	0.68	0.43	RNA synthesis, modification, and DNA transcription	Putative principal RNA polymerase sigma factor
PMM0500	2	-0.67	0.54	0.38	0.28	Conserved hypothetical protein	conserved hypothetical protein
PMM0501	2	#NAME?	NA	NA	0.01	Conserved hypothetical protein	conserved hypothetical protein
PMM0502	2	1.25	0.06	0.05	0.20	Conserved hypothetical protein	conserved hypothetical protein
PMM0503	2	-0.54	1.07	0.76	0.47	Cobalamin, heme, phycobilin and porphyrin	possible precorrin-6X reductase
PMM0506	2	-0.48	1.16	0.82	0.40	Purine ribonucleotide biosynthesis	Adenylosuccinate synthetase
PMM0507	2	-0.16	0.33	0.23	0.65	Photosystem II	possible Photosystem II reaction center Psb27 protein
PMM0508	2	0.14	1.00	0.71	0.84	Aminoacyl tRNA synthetases and tRNA modification	Prolyl-tRNA synthetase
PMM0510	2	-2.09	0.20	0.14	0.00	Transposon-related functions	possible Reverse transcriptase (RNA-dependent
PMM0511	2	-2.11	0.08	0.06	0.00	Other	Inorganic pyrophosphatase
PMM0515	2	-1.81	1.54	1.09	0.00	Cobalamin, heme, phycobilin and porphyrin	possible alpha-ribazole-5'-P phosphatase
PMM0519	2	1.22	0.66	0.47	0.32	Other	Transaldolase
PMM0520	2	-0.38	0.14	0.10	0.48	Other	NAD binding site
PMM0522	2	-0.15	0.07	0.05	0.64	Pyrimidine ribonucleotide biosynthesis	uridylate kinase
PMM0525	2	-0.58	0.30	0.21	0.37	Other	Ferrochelatase
PMM0526	2	0.48	0.04	0.03	1.00	Branched chain family	Acetolactate synthase large subunit
PMM0530	2	-0.37	0.14	0.10	0.37	Ribosomal proteins	30S ribosomal protein S1 homolog B, putative Nbp1
PMM0532	2	0.14	0.39	0.27	0.98	Conserved hypothetical protein	conserved hypothetical protein
PMM0533	2	-0.77	0.62	0.44	0.28	Conserved hypothetical protein	conserved hypothetical protein
PMM0534	2	-1.42	0.31	0.22	0.05	Fatty acid, phospholipid and sterol metabolism	acetyl-CoA carboxylase, alpha subunit
PMM0536	2	-1.19	0.26	0.18	0.10	Folic acid	putative GTP cyclohydrolase I
PMM0537	2	-0.39	0.97	0.68	0.40	Aromatic amino acid family	phosphoribosylanthranilate isomerase
PMM0540	2	-3.57	1.58	1.11	0.00	Photosystem I	possible photosystem I reaction centre subunit XII (PsaM)
PMM0541	2	-0.75	0.50	0.35	0.27	Conserved hypothetical protein	conserved hypothetical protein
PMM0543	2	0.07	0.31	0.22	0.75	Cobalamin, heme, phycobilin and porphyrin	Protochlorophyllide reductase iron-sulfur ATP-binding protein
PMM0544	2	0.25	0.41	0.29	0.69	Cobalamin, heme, phycobilin and porphyrin	Light-independent protochlorophyllide reductase subunit B
PMM0545	2	-1.10	0.09	0.06	0.19	Cobalamin, heme, phycobilin and porphyrin	Light-independent protochlorophyllide reductase subunit N
PMM0546	2	1.78	0.34	0.24	0.02	Conserved hypothetical protein	conserved hypothetical
PMM0548	2	2.96	0.73	0.52	0.00	Other	HAM1 family protein
PMM0549	2	-3.04	0.99	0.70	0.00	CO2 fixation	carboxysome shell protein CsoS1
PMM0550	2	-1.28	0.23	0.16	0.11	CO2 fixation	Ribulose bisphosphate carboxylase, large chain
PMM0551	2	-1.24	0.02	0.01	0.09	CO2 fixation	Ribulose bisphosphate carboxylase, small chain
PMM0552	2	-2.02	0.19	0.13	0.00	CO2 fixation	carboxysome shell protein CsoS2
PMM0554	2	-0.05	0.55	0.39	0.65	CO2 fixation	putative carboxysome peptide A
PMM0555	2	0.20	1.31	0.93	0.81	CO2 fixation	putative carboxysome peptide B
PMM0556	2	-0.17	1.25	0.88	0.76	Conserved hypothetical protein	conserved hypothetical protein
PMM0557	2	-1.28	1.11	0.78	0.01	Conserved hypothetical protein	conserved hypothetical
PMM0558	2	-0.04	0.35	0.25	1.00	Conserved hypothetical protein	conserved hypothetical protein
PMM0560	2	-1.09	0.22	0.15	0.05	Other	possible ATP phosphoribosyltransferase
PMM0561	2	-0.77	1.15	0.81	0.27	Transport and binding proteins	putative multidrug efflux ABC transporter
PMM0564	2	-1.12	0.78	0.55	0.16	Conserved hypothetical protein	conserved hypothetical protein
PMM0565	2	0.93	0.56	0.40	0.41	DNA replication, recombination, and repair	chromosomal replication initiator protein DnaA
PMM0570	2	1.46	0.65	0.46	0.20	NADH dehydrogenase	NADH dehydrogenase subunit NdhL (ndhL)
PMM0571	2	3.88	2.48	1.76	0.00	Conserved hypothetical protein	conserved hypothetical protein
PMM0573	2	-0.78	0.86	0.61	0.25	Conserved hypothetical protein	conserved hypothetical protein
PMM0574	2	-0.64	0.73	0.51	0.48	Conserved hypothetical protein	conserved hypothetical protein
PMM0577	2	-0.48	0.75	0.53	0.85	RNA synthesis, modification, and DNA transcription	Putative type II alternative sigma factor, sigma70 family
PMM0579	2	1.12	0.05	0.03	0.48	Conserved hypothetical protein	conserved hypothetical protein
PMM0580	2	-1.05	0.24	0.17	0.15	Degradation of proteins, peptides, and glycopeptides	ATP-dependent Clp protease, Hsp 100, ATP-binding subunit ClpB
PMM0581	2	-1.01	0.90	0.64	1.00	Soluble electron carriers	plastocyanin
PMM0583	2	-4.21	0.95	0.67	0.00	Cobalamin, heme, phycobilin and porphyrin	Uroporphyrinogen decarboxylase (URO-D)
PMM0586	2	-0.78	0.26	0.18	0.30	Conserved hypothetical protein	conserved hypothetical
PMM0593	2	0.50	0.01	0.01	0.86	Other	Peptidase family M3
PMM0594	2	0.12	0.50	0.35	0.58	NADH dehydrogenase	putative NADH Dehydrogenase (complex I) subunit (chain 4)
PMM0595	2	0.40	0.84	0.60	1.00	Aspartate family	Homoserine kinase:GHMP kinases putative ATP-binding domain
PMM0599	2	-3.64	0.97	0.69	0.00	Conserved hypothetical protein	conserved hypothetical protein
PMM0601	2	-0.78	0.77	0.54	0.33	Transport and binding proteins	ABC transporter, substrate binding protein, possibly Mn
PMM0603	2	-1.02	0.55	0.39	0.22	Transport and binding proteins	ABC transporter component, possibly Mn transport
PMM0605	2	-3.91	0.16	0.11	0.00	Conserved hypothetical protein	conserved hypothetical protein
PMM0609	2	0.10	0.26	0.18	1.00	Polysaccharides and glycoproteins	Putative ADPglucose--glucosyltransferase (GlgA)
PMM0613	2	-0.40	0.23	0.16	0.48	Aromatic amino acid family	EPSP synthase (3-phosphoshikimate 1-carboxyvinyltransferase)
PMM0614	2	-0.73	0.20	0.14	0.34	Conserved hypothetical protein	conserved hypothetical protein
PMM0615	2	0.39	1.02	0.72	0.91	Other	Possible nitrilase
PMM0618	2	1.33	0.51	0.36	0.37	Carotenoid	polyprenyl synthetase; solanesyl diphosphate synthase (sds)
PMM0619	2	1.08	0.04	0.03	0.40	Pyruvate and acetyl-CoA metabolism	acetyl-coenzyme A synthetase
PMM0622	2	-0.39	0.64	0.45	0.52	Conserved hypothetical protein	conserved hypothetical protein
PMM0626	2	2.49	0.31	0.22	0.00	Conserved hypothetical protein	hypothetical
PMM0627	2	-1.55	0.29	0.21	1.00	Photosystem II	light-harvesting complex protein
PMM0628	2	0.93	0.10	0.07	0.37	Transport and binding proteins	possible sodium:solute symporter, ESS family
PMM0629	2	-0.64	0.85	0.60	0.48	Conserved hypothetical protein	conserved hypothetical protein
PMM0633	2	-1.55	1.10	0.78	0.00	Carotenoid	putative lycopene epsilon cyclase
PMM0637	2	0.63	0.35	0.25	0.39	Regulatory functions	Ferric uptake regulator family
PMM0638	2	-0.30	0.18	0.13	0.54	Conserved hypothetical protein	conserved hypothetical protein
PMM0641	2	-0.80	0.52	0.37	0.36	Conserved hypothetical protein	conserved hypothetical protein
PMM0642	2	-0.10	0.93	0.66	0.62	Serine family / Sulfur assimilation	putative O-Acetyl homoserine sulfhydrylase
PMM0647	2	0.73	0.55	0.39	0.51	Conserved hypothetical protein	conserved hypothetical protein
PMM0649	2	-0.87	1.11	0.79	0.36	Other	Pentapeptide repeats
PMM0651	2	2.56	0.28	0.20	0.00	Regulatory functions	possible VHS domain
PMM0652	2	-0.54	0.43	0.30	0.48	RNA synthesis, modification, and DNA transcription	possible 5'-3' exonuclease, C-terminal SAM fol
PMM0658	2	-0.91	0.95	0.67	0.36	Aminoacyl tRNA synthetases and tRNA modification	putative pseudouridylate synthase specific to ribosomal small subunit
PMM0659	2	0.39	0.82	0.58	1.00	DNA replication, recombination, and repair	NAD-dependent DNA ligase N-terminus
PMM0660	2	-1.54	0.49	0.34	0.05	Degradation of RNA	possible RNA recognition motif. (a.k.a. RRM, R
PMM0661	2	1.48	0.09	0.06	0.16	Purine ribonucleotide biosynthesis	ribonucleotide reductase (Class II)
PMM0664	2	0.18	0.18	0.13	1.00	Conserved hypothetical protein	conserved hypothetical protein
PMM0665	2	1.36	0.26	0.19	0.11	Other	Hsp33 protein
PMM0667	2	0.98	1.32	0.94	0.50	Conserved hypothetical protein	conserved hypothetical protein
PMM0674	2	-0.34	0.51	0.36	0.55	Aspartate family	Aminotransferases class-I
PMM0681	2	2.30	0.22	0.16	0.00	Conserved hypothetical protein	conserved hypothetical protein
PMM0683	2	0.86	0.63	0.45	0.37	Purine ribonucleotide biosynthesis	phosphoribosylaminoimidazole carboxylase
PMM0684	2	3.38	0.04	0.03	0.00	Regulatory functions	possible Zinc finger, C2H2 type
PMM0685	2	3.58	0.55	0.39	0.00	Conserved hypothetical protein	hypothetical protein
PMM0687	2	3.13	0.68	0.48	0.00	Conserved hypothetical protein	conserved hypothetical
PMM0688	2	0.67	0.16	0.11	0.48	Protein modification and translation factors	possible Elongation factor Tu domain 2
PMM0689	2	3.44	0.77	0.54	0.00	Adaptations and atypical conditions	possible high light inducible protein
PMM0690	2	4.37	0.58	0.41	0.00	Adaptations and atypical conditions	possible high light inducible protein
PMM0691	2	-1.54	1.35	0.96	0.01	Conserved hypothetical protein	conserved hypothetical protein
PMM0692	2	-2.43	0.50	0.35	0.00	Regulatory functions	possible DDT domain
PMM0693	2	1.17	1.59	1.12	0.25	Other	possible Hepatitis C virus envelope glycoprote
PMM0697	2	1.08	2.01	1.42	0.47	DNA replication, recombination, and repair	possible D12 class N6 adenine-specific DNA met

PMM0698	2	2.19	1.55	1.09	0.00	Chaperones	possible DnaJ central domain (4 repeats)
PMM0699	2	-2.12	1.79	1.26	0.01	Conserved hypothetical protein	conserved hypothetical
PMM0700	2	-0.39	0.51	0.36	0.60	Conserved hypothetical protein	conserved hypothetical protein
PMM0703	2	0.61	0.68	0.48	0.80	Conserved hypothetical protein	conserved hypothetical protein
PMM0704	2	1.87	0.28	0.20	0.01	Regulatory functions	putative potassium channel, VIC family
PMM0705	2	0.59	0.34	0.24	0.63	Regulatory functions	two-component response regulator, phosphate
PMM0707	2	1.29	0.56	0.40	0.13	Other	possible Lipoprotein
PMM0708	2	1.52	0.21	0.15	0.13	Regulatory functions	putative secreted protein
PMM0709	2	0.48	0.22	0.16	1.00	Membranes, lipoproteins and porins	possible porin
PMM0710	2	-4.00	0.03	0.02	0.00	Transport and binding proteins	ABC transporter, substrate binding protein, phosphate
PMM0714	2	1.61	1.51	1.07	0.27	Regulatory functions	Bacterial regulatory proteins, ArsR family
PMM0717	2	-0.56	0.59	0.41	0.43	Conserved hypothetical protein	conserved hypothetical protein
PMM0719	2	0.72	0.16	0.11	0.56	Conserved hypothetical protein	hypothetical
PMM0722	2	-0.08	2.95	2.08	0.97	Conserved hypothetical protein	hypothetical
PMM0725	2	0.52	0.01	0.01	1.00	Transport and binding proteins	putative phosphate ABC transporter, ATP binding subunit
PMM0726	2	3.59	1.24	0.88	0.00	Conserved hypothetical protein	hypothetical
PMM0732	2	-0.35	0.58	0.41	0.53	Other	possible Major surface glycoprotein
PMM0736	2	-0.48	1.09	0.77	0.50	Other	possible Alpha-2-macroglobulin family N-termin
PMM0739	2	1.04	0.21	0.15	0.33	Conserved hypothetical protein	conserved hypothetical protein
PMM0740	2	-0.11	0.15	0.10	0.48	Cytochrome b6/f complex	Cytochrome b6-f complex subunit VIII
PMM0741	2	-0.07	0.72	0.51	0.63	Conserved hypothetical protein	conserved hypothetical protein
PMM0742	2	1.24	0.62	0.44	0.38	Degradation of proteins, peptides, and glycopeptides	Clp protease subunit
PMM0743	2	-0.83	0.16	0.11	0.28	Cell division	FtsH ATP-dependent protease homolog
PMM0744	2	0.21	3.01	2.13	0.65	Conserved hypothetical protein	conserved hypothetical protein
PMM0747	2	-2.43	0.58	0.41	0.00	Cobalamin, heme, phycobilin and porphyrin	ferredoxin-dependent biliverdin reductase
PMM0751	2	-2.80	1.09	0.77	0.00	Conserved hypothetical protein	conserved hypothetical protein
PMM0753	2	-1.08	0.33	0.24	0.21	Ribosomal proteins	30S ribosomal protein S2
PMM0754	2	-1.27	0.86	0.61	0.03	Protein modification and translation factors	putative Elongation factor Ts
PMM0758	2	0.16	0.93	0.66	1.00	Serine family / Sulfur assimilation	Ferredoxin-sulfite reductase
PMM0760	2	-0.97	0.16	0.11	0.18	Cobalamin, heme, phycobilin and porphyrin	Aromatic-ring hydroxylase (flavoprotein monooxygenase)
PMM0762	2	-0.48	0.01	0.01	0.46	Aromatic amino acid family	tyrosine binding protein
PMM0766	2	-2.40	1.46	1.04	0.00	Other	Ribulose-phosphate 3-epimerase
PMM0767	2	-1.48	0.36	0.25	0.01	Surface polysaccharides, lipopolysaccharides and antigens	Fructose-1,6-bisphosphatase/sedoheptulose-1,7-bis phosphatase
PMM0769	2	-1.95	0.20	0.14	0.00	Other	ADP-glucose pyrophosphorylase
PMM0770	2	1.60	0.51	0.36	0.11	Pentose phosphate pathway	6-phosphogluconate dehydrogenase
PMM0772	2	0.56	1.20	0.85	0.69	Conserved hypothetical protein	conserved hypothetical protein
PMM0774	2	1.22	0.41	0.29	0.28	Branched chain family	Dihydroxy-acid dehydratase
PMM0775	2	0.14	0.97	0.68	0.95	Conserved hypothetical protein	conserved hypothetical
PMM0777	2	0.36	0.18	0.12	1.00	Conserved hypothetical protein	conserved hypothetical protein
PMM0779	2	-0.80	3.07	2.17	0.35	Conserved hypothetical protein	conserved hypothetical protein
PMM0781	2	-1.53	0.68	0.48	0.00	Fatty acid, phospholipid and sterol metabolism	Fructose-bisphosphate/sedoheptulose-1,7-bisphosph ate aldolase
PMM0784	2	-0.74	1.17	0.82	0.37	Fatty acid, phospholipid and sterol metabolism	acetyl-CoA carboxylase, beta subunit
PMM0785	2	-4.13	1.35	0.96	0.00	CO2 fixation	phosphoribulokinase
PMM0790	2	0.24	0.03	0.02	1.00	Conserved hypothetical protein	conserved hypothetical protein
PMM0794	2	-0.25	0.46	0.33	0.55	Conserved hypothetical protein	conserved hypothetical protein
PMM0797	2	-2.70	1.56	1.11	0.00	Nucleoproteins	possible mRNA binding protein
PMM0799	2	-2.96	1.34	0.95	0.00	Conserved hypothetical protein	conserved hypothetical protein
PMM0800	2	-1.86	0.09	0.07	0.03	Conserved hypothetical protein	conserved hypothetical protein
PMM0801	2	-2.19	2.14	1.51	0.00	Conserved hypothetical protein	conserved hypothetical protein
PMM0802	2	1.29	0.70	0.49	0.24	DNA replication, recombination, and repair	putative endonuclease
PMM0804	2	1.40	0.49	0.34	1.00	Other	ferritin
PMM0806	2	1.62	1.11	0.78	0.00	Regulatory functions	Bacterial regulatory proteins, Crp family
PMM0810	2	1.63	0.53	0.38	0.07	Conserved hypothetical protein	hypothetical
PMM0812	2	2.40	0.26	0.19	0.00	Conserved hypothetical protein	hypothetical
PMM0814	2	-1.09	0.80	0.57	0.18	Other	possible Cytochrome oxidase c subunit VIb
PMM0815	2	3.08	0.52	0.37	0.00	Adaptations and atypical conditions	possible high light inducible protein
PMM0816	2	4.01	0.53	0.37	0.00	Adaptations and atypical conditions	possible high light inducible protein
PMM0817	2	3.66	0.29	0.21	0.00	Adaptations and atypical conditions	possible high light inducible protein
PMM0818	2	4.26	0.12	0.08	0.00	Adaptations and atypical conditions	possible high light inducible protein
PMM0819	2	3.96	0.08	0.05	0.00	Conserved hypothetical protein	hypothetical
PMM0820	2	-1.60	0.10	0.07	0.11	Aromatic amino acid family	possible EPSP synthase (3-phosphoshikimate 1-c
PMM0821	2	0.56	0.07	0.05	0.79	Conserved hypothetical protein	conserved hypothetical protein
PMM0824	2	-0.23	0.33	0.23	0.53	Conserved hypothetical protein	conserved hypothetical protein
PMM0828	2	0.79	0.54	0.38	0.55	Other	S4 domain
PMM0829	2	0.32	0.10	0.07	1.00	Other	Triosephosphate isomerase
PMM0835	2	-4.13	0.91	0.64	0.00	Conserved hypothetical protein	conserved hypothetical protein
PMM0844	2	-1.49	0.43	0.31	0.02	Adaptations and atypical conditions	phytochrome-regulated gene
PMM0845	2	-0.06	0.82	0.58	0.53	Conserved hypothetical protein	conserved hypothetical protein
PMM0846	2	-0.25	0.47	0.33	0.50	Other	possible Uncharacterized secreted proteins, Ya
PMM0847	2	-0.83	0.19	0.14	0.28	Drug and analog sensitivity	putative acetazolamide conferring resistance protein Zam
PMM0851	2	-2.07	0.86	0.61	0.00	Other	Putative CbbY homolog
PMM0852	2	2.40	0.07	0.05	0.00	Conserved hypothetical protein	conserved hypothetical protein
PMM0853	2	-1.36	1.18	0.83	0.01	Ribosomal proteins	50S ribosomal protein L32
PMM0856	2	-0.77	0.77	0.54	0.96	Detoxification	thioredoxin peroxidase
PMM0857	2	-3.70	0.89	0.63	0.00	Other	possible Influenza RNA-dependent RNA polymeras
PMM0858	2	2.80	0.33	0.23	0.00	Conserved hypothetical protein	hypothetical
PMM0861	2	-0.83	0.10	0.07	0.91	Transport and binding proteins	possible Virion host shutoff protein
PMM0863	2	-0.70	0.19	0.14	0.33	Cobalamin, heme, phycobilin and porphyrin	putative cobinamide kinase
PMM0864	2	-2.44	1.71	1.21	0.00	Other	possible Fusion glycoprotein F0.
PMM0867	2	-0.53	1.56	1.10	0.37	Aminoacyl tRNA synthetases and tRNA modification	Methionyl-tRNA synthetase
PMM0869	2	-2.64	0.80	0.57	0.00	Ribosomal proteins	30S Ribosomal protein S18
PMM0870	2	-3.22	0.17	0.12	0.00	Ribosomal proteins	50S Ribosomal protein L33
PMM0872	2	-0.38	0.17	0.12	0.84	Other	possible Carboxylesterase
PMM0876	2	-2.18	0.09	0.06	0.00	Conserved hypothetical protein	conserved hypothetical
PMM0878	2	0.13	0.05	0.03	0.84	Branched chain family	putative Branched-chain amino acid aminotransferase
PMM0881	2	-0.16	1.16	0.82	0.54	Conserved hypothetical protein	conserved hypothetical protein
PMM0883	2	0.49	0.68	0.48	0.89	Conserved hypothetical protein	conserved hypothetical protein
PMM0893	2	0.21	1.37	0.97	0.76	Riboflavin	possible GTP cyclohydrolase II / 3,4-dihydroxy-2-butanone 4-phosphate synthase
PMM0894	2	-1.60	0.13	0.09	0.00	Protein modification and translation factors	Cyclophilin-type peptidyl-prolyl cis-trans isomerase
PMM0895	2	-0.52	2.55	1.80	1.00	Conserved hypothetical protein	conserved hypothetical protein
PMM0896	2	1.14	0.42	0.30	0.17	Chaperones	DnaJ2 protein
PMM0897	2	0.62	0.06	0.04	1.00	Chaperones	Molecular chaperone DnaK, heat shock protein hsp70
PMM0898	2	-0.67	0.30	0.22	0.35	Soluble electron carriers	ferredoxin, petF-like protein
PMM0899	2	0.27	0.82	0.58	1.00	Regulatory functions	Possible myo-inositol-1(or 4)-monophosphatase
PMM0901	2	-1.84	0.68	0.48	0.00	Chaperones	heat shock protein HtpG
PMM0902	2	-0.84	0.32	0.23	0.21	Ribosomal proteins	50S ribosomal protein L28
PMM0906	2	-1.96	0.34	0.24	0.00	Photosystem I	Photosystem I PsaK protein (subunit X)
PMM0907	2	-1.06	0.12	0.08	0.17	Sugars	1-deoxy-D-xylulose 5-phosphate synthase
PMM0910	2	0.84	1.18	0.84	0.86	Conserved hypothetical protein	conserved hypothetical membrane protein
PMM0912	2	0.09	0.35	0.25	0.63	Other	Pyruvate kinase
PMM0913	2	0.51	0.69	0.49	1.00	Transport and binding proteins	possible ABC transporter
PMM0919	2	0.26	0.46	0.32	0.83	Branched chain family	serine:pyruvate/alanine:glyoxylate aminotransferase
PMM0920	2	2.45	0.57	0.41	0.00	Glutamate family / Nitrogen assimilation	Glutamine synthetase, glutamate--ammonia ligase
PMM0922	2	0.14	2.04	1.44	0.96	Conserved hypothetical protein	conserved hypothetical protein
PMM0923	2	-3.12	0.36	0.25	0.00	Conserved hypothetical protein	conserved hypothetical protein
PMM0926	2	-0.45	0.29	0.20	0.51	Photosystem II	possible Photosystem II reaction center Psb28 protein
PMM0930	2	-2.02	0.98	0.70	0.00	Other	Pyruvate dehydrogenase E1 beta subunit
PMM0934	2	-0.04	0.89	0.63	1.00	Conserved hypothetical protein	conserved hypothetical protein
PMM0936	2	2.30	2.05	1.45	0.03	DNA replication, recombination, and repair	putative SOS mutagenesis protein UmuD
PMM0941	2	-0.26	0.23	0.16	0.59	Other	possible cAMP phosphodiesterases class-II
PMM0942	2	-1.15	0.08	0.06	0.15	DNA replication, recombination, and repair	putative holliday junction DNA helicase RuvA
PMM0943	2	-1.77	0.30	0.21	0.00	Ribosomal proteins	30S Ribosomal protein S15
PMM0946	2	0.83	0.03	0.02	0.79	Aminoacyl tRNA synthetases and tRNA modification	Glutamyl-tRNA(Gln) amidotransferase A subunit
PMM0947	2	-0.10	3.08	2.18	0.35	Conserved hypothetical protein	conserved hypothetical protein
PMM0949	2	0.68	1.34	0.94	1.00	Conserved hypothetical protein	conserved hypothetical protein
PMM0950	2	0.02	1.15	0.81	0.48	Other	No Cyanobase Name
PMM0953	2	-1.61	0.50	0.36	0.15	Conserved hypothetical protein	conserved hypothetical protein
PMM0954	2	0.16	0.46	0.33	1.00	Transport and binding proteins	ABC transporter, multidrug efflux family

PMM0955	2	0.06	0.30	0.22	1.00	Protein modification and translation factors	Peptide methionine sulfoxide reductase
PMM0957	2	1.49	0.47	0.33	0.06	Regulatory functions	possible GRAM domain
PMM0958	2	1.11	0.47	0.33	0.21	Conserved hypothetical protein	conserved hypothetical
PMM0963	2	1.66	0.65	0.46	0.05	Nitrogen metabolism	Urease alpha subunit
PMM0964	2	2.33	0.14	0.10	0.00	Nitrogen metabolism	Urease beta subunit
PMM0965	2	3.08	0.72	0.51	0.00	Nitrogen metabolism	Urease gamma subunit
PMM0966	2	1.05	0.25	0.18	0.29	Nitrogen metabolism	urease accessory protein UreD
PMM0969	2	1.85	0.94	0.66	0.05	Nitrogen metabolism	urease accessory protein UreG
PMM0970	2	2.80	0.27	0.19	0.01	Transport and binding proteins	putative urea ABC transporter, substrate binding protein
PMM0971	2	1.39	0.38	0.27	0.08	Transport and binding proteins	putative urea ABC transporter
PMM0974	2	-0.46	0.93	0.66	0.94	Transport and binding proteins	Putative ATP-binding subunit of urea ABC transport system
PMM0975	2	-0.21	0.74	0.53	0.59	Conserved hypothetical protein	conserved hypothetical protein
PMM0982	2	-0.41	0.03	0.02	0.52	DNA replication, recombination, and repair	HNH endonuclease:HNH nuclease
PMM0983	2	-1.41	0.06	0.04	0.05	Fatty acid, phospholipid and sterol metabolism	possible ATP synthase protein 8
PMM0987	2	-3.56	0.79	0.56	0.00	Ribosomal proteins	30S Ribosomal protein S21
PMM0988	2	0.60	1.04	0.74	1.00	Regulatory functions	Helix-hairpin-helix DNA-binding motif class 1
PMM0993	2	-3.24	1.72	1.22	0.00	Conserved hypothetical protein	conserved hypothetical protein
PMM0996	2	2.19	0.81	0.57	0.01	Conserved hypothetical protein	conserved hypothetical protein
PMM0997	2	0.20	0.39	0.28	0.65	Conserved hypothetical protein	possible Protein of unknown function DUF67
PMM0998	2	1.58	0.61	0.43	0.03	Conserved hypothetical protein	conserved hypothetical protein
PMM0999	2	-1.94	0.67	0.47	0.02	Conserved hypothetical protein	hypothetical
PMM1003	2	-3.57	1.13	0.80	0.00	Photosystem II	possible Photosystem II reaction centre N prot
PMM1005	2	-1.37	0.30	0.21	0.06	Regulatory functions	possible Legume lectins alpha domain
PMM1008	2	-0.14	0.57	0.40	0.71	Conserved hypothetical protein	hypothetical
PMM1011	2	-3.04	2.02	1.43	0.00	Conserved hypothetical protein	hypothetical
PMM1015	2	4.10	2.14	1.52	0.00	Conserved hypothetical protein	conserved hypothetical protein
PMM1026	2	0.43	0.11	0.08	1.00	Conserved hypothetical protein	conserved hypothetical protein
PMM1028	2	#NAME?	NA	NA	0.00	Conserved hypothetical protein	conserved hypothetical
PMM102a	2	-0.90	0.64	0.46	0.16	Other	No Cyanobase Name
PMM1030	2	-1.22	0.40	0.29	0.06	Regulatory functions	Ferric uptake regulator family
PMM1031	2	3.74	0.47	0.33	0.00	Transport and binding proteins	ABC transporter, ATP binding domain, possibly Mn transport
PMM1032	2	3.48	0.24	0.17	0.00	Transport and binding proteins	ABC transporter, substrate binding protein, possibly Mn.
PMM1033	2	0.50	0.52	0.36	1.00	Protein modification and translation factors	Cobalamin synthesis protein/P47K
PMM1037	2	Inf	NA	NA	0.00	Conserved hypothetical protein	conserved hypothetical
PMM1039	2	-0.91	0.28	0.20	0.21	Conserved hypothetical protein	conserved hypothetical protein
PMM1042	2	-1.21	0.66	0.47	0.03	Conserved hypothetical protein	conserved hpothetical protein
PMM1045	2	0.83	0.83	0.59	0.47	Conserved hypothetical protein	conserved hypothetical
PMM1054	2	1.01	0.13	0.09	0.33	DNA replication, recombination, and repair	Crossover junction endodeoxyribonuclease RuvC
PMM1055	2	-1.54	0.50	0.36	0.04	Cobalamin, heme, phycobilin and porphyrin	Protoporphyrin IX Magnesium chelatase, ChlI subunit
PMM1058	2	-3.20	0.39	0.28	0.00	Cytochrome b6/f complex	Cytochrome b6/f complex, subunit V
PMM1060	2	0.75	0.60	0.43	1.00	Other	Glutamine amidotransferase class-I
PMM1061	2	0.17	0.54	0.38	0.80	Thiamine	Thioredoxin
PMM1063	2	1.26	1.01	0.71	0.60	DNA replication, recombination, and repair	DNA gyrase/topoisomerase IV, subunit A
PMM1066	2	0.51	0.84	0.60	1.00	Branched chain family	2-isopropylmalate synthase
PMM1067	2	-0.05	0.89	0.63	0.57	WD repeat proteins	possible Adenoviral fiber protein (repeat/shaf
PMM1069	2	-2.35	1.00	0.70	0.00	Folic acid	putuative bifunctional Methylenetetrahydrofolate dehydrogenase Methenyltetrahydrofolate/cyclohydrolase
PMM1074	2	1.48	0.11	0.08	0.15	Other	Glucose-6-phosphate dehydrogenase
PMM1075	2	1.16	0.09	0.06	0.68	Cytochrome b6/f complex	ferredoxin-NADP oxidoreductase (FNR)
PMM1077	2	0.85	0.55	0.39	0.94	Regulatory functions	two-component sensor histidine kinase
PMM1078	2	0.38	0.60	0.42	0.87	Conserved hypothetical protein	conserved hypothetical
PMM1079	2	-0.95	1.18	0.83	0.16	Regulatory functions	possible Villin headpiece domain
PMM1080	2	0.27	1.38	0.98	1.00	Other	Ribose-phosphate pyrophosphokinase
PMM1086	2	0.10	0.32	0.23	1.00	Fatty acid, phospholipid and sterol metabolism	Alpha/beta hydrolase fold:Esterase/lipase/thioesterase family...
PMM1088	2	0.40	0.40	0.28	0.84	Degradation of proteins, peptides, and glycopeptides	ClpC
PMM1090	2	0.21	0.47	0.34	0.80	Branched chain family	Diaminopimelate decarboxylase
PMM1091	2	0.61	1.45	1.02	0.65	Conserved hypothetical protein	conserved hypothetical protein
PMM1092	2	0.63	0.39	0.28	0.91	Other	Undecaprenyl pyrophosphate synthetase (UPPS)
PMM1098	2	0.17	0.83	0.59	0.78	Photosystem II	photosystem II oxygen evolving complex protein PsbP
PMM1107	2	0.10	0.26	0.19	0.81	Pyridoxine	Pyridoxal phosphate biosynthetic protein PdxJ
PMM1109	2	-2.65	0.02	0.02	0.00	Conserved hypothetical protein	conserved hypothetical protein
PMM1111	2	1.31	1.16	0.82	0.20	Other	Glutaredoxin-related protein
PMM1113	2	-0.74	0.61	0.43	0.31	Regulatory functions	two-component response regulator
PMM1116	2	-1.47	0.56	0.40	0.00	Cell division	NAD binding site:Glucose inhibited division protein A family
PMM1117	2	-1.86	0.30	0.21	0.00	Photosystem II	possible Photosystem II reaction center Y protein (PsbY)
PMM1118	2	1.52	0.20	0.14	0.36	Adaptations and atypical conditions	possible high light inducible protein
PMM1119	2	1.98	0.42	0.30	1.00	Membranes, lipoproteins and porins	possible porin
PMM1121	2	2.04	0.26	0.19	1.00	Membranes, lipoproteins and porins	possible porin
PMM1123	2	2.04	0.43	0.30	0.07	Hydrogenase	putative hydrogenase accessory protein
PMM1124	2	0.65	0.03	0.02	1.00	Other	possible Natural resistance-associated macroph
PMM1125	2	-3.35	0.70	0.50	0.00	Regulatory functions	possible Bacterial regulatory proteins, deoR f
PMM1128	2	1.68	0.25	0.18	0.03	Adaptations and atypical conditions	possible high light inducible protein
PMM1129	2	0.29	0.11	0.08	1.00	Regulatory functions	possible Notch (DSL) domain
PMM1131	2	0.34	0.06	0.04	1.00	Conserved hypothetical protein	conserved hypothetical protein
PMM1132	2	1.35	0.38	0.27	0.39	Conserved hypothetical protein	conserved hypothetical protein
PMM1133	2	0.33	0.82	0.58	1.00	Conserved hypothetical protein	conserved hypothetical protein
PMM1134	2	2.87	0.55	0.39	0.00	Other	possible Phosphatidylinositol-specific phospho
PMM1135	2	2.56	0.17	0.12	0.00	Adaptations and atypical conditions	possible high light inducible protein
PMM1138	2	0.62	0.85	0.60	0.98	Regulatory functions	probable GTP-binding protein
PMM1139	2	0.10	0.42	0.30	0.84	Drug and analog sensitivity	possible membrane fusion protein
PMM1145	2	0.02	0.43	0.31	0.89	Respiration	putative nicotinamide nucleotide transhydrogenase, subunit beta
PMM1146	2	2.09	1.55	1.09	0.04	Nicotinate and nicotinamide	putative nicotinamide nucleotide transhydrogenase, subunit alpha 2 (A2)
PMM1147	2	0.74	1.09	0.77	1.00	DNA replication, recombination, and repair	putative nicotinamide nucleotide transhydrogenase, subunit alpha 1 (A1)
PMM1148	2	-1.11	0.03	0.02	0.05	Protein modification and translation factors	possible EF-1 guanine nucleotide exchange doma
PMM1149	2	2.92	0.19	0.14	0.00	Conserved hypothetical protein	conserved hypothetical
PMM1150	2	2.30	0.19	0.13	0.00	Regulatory functions	putative thioredoxin reductase
PMM1151	2	3.28	0.16	0.11	0.00	Protein modification and translation factors	translation initiation factor IF-1
PMM1152	2	0.27	0.66	0.47	0.86	Photosystem II	putative chaperon-like protein for quinone binding in photosystem II
PMM1152a	2	0.46	1.06	0.75	0.85	Other	No Cyanobase Name
PMM1154	2	1.38	0.76	0.54	0.21	Branched chain family	Acetolactate synthase small subunit
PMM1156	2	-0.37	0.20	0.14	0.37	Photosystem I	photosystem I assembly related protein Ycf4
PMM1157	2	1.58	0.39	0.27	1.00	Photosystem II	Photosystem II PsbD protein (D2)
PMM1158	2	0.88	0.06	0.04	0.97	Photosystem II	Photosystem II PsbC protein (CP43)
PMM1165	2	0.69	0.40	0.28	0.69	Aminoacyl tRNA synthetases and tRNA modification	Glycyl-tRNA synthetase alpha subunit
PMM1167	2	-0.17	0.15	0.11	0.76	Other	Macrophage migration inhibitory factor family
PMM1168	2	0.84	0.78	0.55	0.37	Conserved hypothetical protein	conserved hypothetical protein
PMM1169	2	-0.07	1.00	0.71	0.50	Conserved hypothetical protein	hypothetical
PMM1170	2	-1.32	0.65	0.46	0.08	Conserved hypothetical protein	conserved hypothetical protein
PMM1171	2	-2.69	1.39	0.98	0.00	Soluble electron carriers	Flavodoxin
PMM1174	2	-1.27	1.71	1.21	0.12	Conserved hypothetical protein	hypothetical
PMM1176	2	-0.39	0.30	0.21	0.52	Regulatory functions	possible Helix-turn-helix protein, copG family
PMM1179	2	1.73	0.25	0.17	0.01	Regulatory functions	putative SMR family transporter, possible pecM homologue
PMM1180	2	-0.39	0.16	0.11	0.47	Degradation of proteins, peptides, and glycopeptides	signal peptide peptidase SppA (protease IV)
PMM1183	2	-2.22	1.26	0.89	0.00	Ribosomal proteins	50S ribosomal protein L34
PMM1184	2	-1.31	0.66	0.46	0.09	Degradation of RNA	Bacterial ribonuclease P protein component
PMM1185	2	-0.71	0.32	0.23	0.32	Conserved hypothetical protein	conserved hypothetical protein
PMM1186	2	0.61	0.18	0.13	0.93	Regulatory functions	Putative inner membrane protein; similar to 60 kDa inner membrane protein family
PMM1188	2	-0.49	0.47	0.33	0.42	Aminoacyl tRNA synthetases and tRNA modification	Seryl-tRNA synthetase
PMM1190	2	-0.80	0.59	0.41	0.18	Ribosomal proteins	30S Ribosomal protein S14
PMM1191	2	-1.33	0.42	0.30	0.09	RNA synthesis, modification, and DNA transcription	polyribonucleotide nucleotidyltransferase
PMM1192	2	-0.29	1.07	0.76	0.53	Other	CysQ protein homolog
PMM1204	2	2.54	1.39	0.98	0.00	Surface polysaccharides, lipopolysaccharides and antigens	glucose-1-phosphate cytidylyltransferase
PMM1205	2	-0.49	0.36	0.25	0.46	Sugars	NDP-hexose 3,4-dehydratase
PMM1229	2	-0.15	0.17	0.12	0.59	Respiration	Dehydrogenase, E1 component
PMM1234	2	0.33	0.18	0.13	1.00	Hydrogenase	Zinc-containing alcohol dehydrogenase superfamily
PMM1235	2	0.70	1.11	0.78	0.61	Transport and binding proteins	possible N-terminal fragment of transketolase
PMM1240	2	-4.39	2.28	1.61	0.00	Other	methyltransferase
PMM1241	2	0.85	0.15	0.11	0.36	Conserved hypothetical protein	hypothetical protein
PMM1244	2	0.73	0.80	0.57	0.60	Conserved hypothetical protein	hypothetical protein

PMM1245	2	0.39	0.89	0.63	1.00	Conserved hypothetical protein	conserved hypothetical protein
PMM1250	2	-0.12	0.30	0.22	0.58	Conserved hypothetical protein	conserved hypothetical protein
PMM1251	2	0.89	0.28	0.20	0.84	Other	Carbamoyltransferase
PMM1252	2	-0.79	0.67	0.47	0.22	Other	possible acetyltransferase
PMM1257	2	-1.08	0.79	0.56	0.15	Surface polysaccharides, lipopolysaccharides and antigens	possible dTDP-glucose 4,6-dehydratase
PMM1258	2	0.90	0.02	0.02	0.69	Pyridoxine	pyridoxal-phosphate-dependent aminotransferase
PMM1259	2	3.51	0.69	0.49	0.00	Pyridoxine	pyridoxal-phosphate-dependent aminotransferase
PMM1260	2	-0.41	0.90	0.64	0.48	Transport and binding proteins	Nucleoside-diphosphate-sugar epimerase
PMM1261	2	-0.22	0.72	0.51	0.53	Sugars	UDP-glucose 6-dehydrogenase
PMM1262	2	2.02	0.23	0.16	0.02	Regulatory functions	SOS function regulatory protein, LexA repressor
PMM1264	2	-0.56	0.99	0.70	0.22	Cell division	cell division protein FtsH3
PMM1267	2	-0.84	1.51	1.07	0.12	Conserved hypothetical protein	conserved hypothetical
PMM1269	2	-0.37	0.09	0.07	1.00	Transport and binding proteins	predicted sugar kinase
PMM1270	2	-0.52	0.33	0.24	0.46	Aminoacyl tRNA synthetases and tRNA modification	Phenylalanyl-tRNA synthetase alpha chain
PMM1272	2	1.96	1.72	1.22	0.14	Conserved hypothetical protein	conserved hypothetical protein
PMM1273	2	-0.05	0.41	0.29	0.60	Riboflavin	putative riboflavin kinase/FAD synthase
PMM1275	2	-1.23	1.18	0.84	0.43	Conserved hypothetical protein	DUF170
PMM1276	2	-2.96	0.47	0.33	0.00	Conserved hypothetical protein	conserved hypothetical protein
PMM1277	2	-0.03	0.49	0.35	0.61	Conserved hypothetical protein	conserved hypothetical protein
PMM1283	2	0.84	0.03	0.02	0.94	Conserved hypothetical protein	conserved hypothetical protein
PMM1284	2	0.21	0.54	0.38	1.00	Adaptations and atypical conditions	PhoH-like phosphate starvation-inducible protein
PMM1285	2	-3.29	1.21	0.85	0.00	Ribosomal proteins	30S Ribosomal protein S16
PMM1286	2	-0.26	0.66	0.46	0.55	Protein and peptide secretion	signal recognition particle protein (SRP54)
PMM1287	2	0.02	0.26	0.18	0.63	Conserved hypothetical protein	conserved hypothetical protein
PMM1288	2	-0.68	0.19	0.14	0.97	Other	Pyruvate dehydrogenase E1 alpha subunit
PMM1289	2	0.47	0.70	0.49	0.91	Transport and binding proteins	Type II alternative RNA polymerase sigma factor, sigma-70 family
PMM1293	2	0.15	0.58	0.41	0.61	Other	FKBP-type peptidyl-prolyl cis-trans isomerase (PPIase)
PMM1294	2	0.60	0.33	0.23	1.00	Hydrogenase	putative nickel-containing superoxide dismutase precursor (NISOD)
PMM1296	2	0.90	0.50	0.35	1.00	Other	marine cyanobacterial conserved hypothetical
PMM1298	2	-2.51	0.37	0.26	0.00	Regulatory functions	putative dihydrolipoamide dehydrogenase
PMM1299	2	-0.68	0.71	0.51	0.30	RNA synthesis, modification, and DNA transcription	tRNA/rRNA methyltransferase (SpoU)
PMM1300	2	0.83	0.16	0.11	0.82	Degradation of RNA	UDP-N-glucosamine 1-carboxyvinyltransferase
PMM1301	2	0.94	0.43	0.30	0.94	Glutamate family / Nitrogen assimilation	Aminotransferase class-III pyridoxal-phosphate:Acetylornithin...
PMM1304	2	0.86	0.69	0.49	0.54	Other	possible cytosine deaminase
PMM1305	2	0.55	0.81	0.57	1.00	Conserved hypothetical protein	conserved hypothetical protein
PMM1307	2	1.07	0.15	0.10	0.35	Conserved hypothetical protein	conserved hypothetical protein
PMM1309	2	0.73	0.39	0.28	1.00	Cell division	Cell division protein FtsZ:Tubulin/FtsZ family
PMM1310	2	1.42	0.02	0.01	0.18	Regulatory functions	putative Ketopantoate hydroxymethyltransferase
PMM1312	2	2.02	0.73	0.52	0.04	Conserved hypothetical protein	conserved hypothetical protein
PMM1313	2	1.71	0.18	0.13	0.07	Degradation of proteins, peptides, and glycopeptides	Clp protease proteolytic subunit
PMM1314	2	3.00	0.79	0.56	0.00	Degradation of proteins, peptides, and glycopeptides	Clp protease proteolytic subunit
PMM1315	2	-0.69	0.06	0.04	0.41	Branched chain family	Ketol-acid reductoisomerase
PMM1317	2	-1.63	0.36	0.25	0.03	Adaptations and atypical conditions	possible high light inducible protein
PMM1318	2	-1.94	0.36	0.25	0.00	Conserved hypothetical protein	conserved hypothetical
PMM1319	2	0.58	1.28	0.90	0.81	Drug and analog sensitivity	possible Beta-lactamase
PMM1321	2	1.98	0.29	0.21	0.28	Nucleoproteins	Bacterial histone-like DNA-binding protein
PMM1322	2	1.09	0.29	0.20	0.55	Other	Putative isoamylase
PMM1323	2	-0.31	0.29	0.21	0.55	Regulatory functions	putative GPH family sugar transporter
PMM1324	2	0.72	0.22	0.16	0.51	Transport and binding proteins	possible transporter, membrane component
PMM1325	2	0.38	0.43	0.30	1.00	Conserved hypothetical protein	conserved hypothetical protein
PMM1327	2	2.39	1.03	0.73	0.01	Conserved hypothetical protein	conserved hypothetical protein
PMM1330	2	0.11	1.70	1.20	1.00	Conserved hypothetical protein	conserved hypothetical protein
PMM1331	2	-0.46	0.56	0.40	0.48	Conserved hypothetical protein	conserved hypothetical protein
PMM1333	2	0.99	0.35	0.25	0.56	Protein modification and translation factors	Peptide methionine sulfoxide reductase
PMM1336	2	-1.34	0.26	0.18	0.09	Fatty acid, phospholipid and sterol metabolism	Putative (3R)-hydroxymyristoyl-[acyl carrier protein] dehydratase
PMM1339	2	-0.70	0.34	0.24	0.33	Purine ribonucleotide biosynthesis	SAICAR synthetase
PMM1340	2	0.52	0.02	0.01	0.86	Purine ribonucleotide biosynthesis	phosphoribosylglycinamide synthetase
PMM1342	2	0.24	0.34	0.24	1.00	Other	possible circadian clock protein KaiC
PMM1343	2	-1.03	1.02	0.72	0.19	Other	possible circadian oscillation regulator KaiB
PMM1344	2	-2.59	0.50	0.36	0.00	Ribosomal proteins	50S ribosomal protein L21
PMM1345	2	-2.84	0.51	0.36	0.00	Ribosomal proteins	50S ribosomal protein L27
PMM1346	2	-2.29	1.06	0.75	0.00	Conserved hypothetical protein	conserved hypothetical protein
PMM1349	2	0.86	0.56	0.39	0.88	Conserved hypothetical protein	conserved hypothetical protein
PMM1350	2	-3.97	1.45	1.02	0.00	Other	Pentapeptide repeats
PMM1351	2	2.52	0.15	0.11	0.00	Conserved hypothetical protein	conserved hypothetical protein
PMM1352	2	1.08	0.68	0.48	0.96	Soluble electron carriers	ferredoxin
PMM1354	2	0.26	0.18	0.13	0.82	Regulatory functions	putative D-3-phosphoglycerate dehydrogenase (PGDH)
PMM1355	2	1.03	0.74	0.52	0.17	Conserved hypothetical protein	conserved hypothetical protein
PMM1363	2	0.03	2.11	1.49	1.00	Conserved hypothetical protein	hypothetical
PMM1365	2	-0.28	2.50	1.77	0.23	Regulatory functions	possible MATH domain
PMM1368	2	0.26	0.13	0.09	1.00	Conserved hypothetical protein	conserved hypothetical protein
PMM1369	2	-2.66	0.08	0.06	0.00	Regulatory functions	GAF domain
PMM1372	2	-0.35	0.85	0.60	0.40	Conserved hypothetical protein	conserved hypothetical protein
PMM1375	2	-2.86	0.97	0.68	0.00	Conserved hypothetical protein	possible M protein repeat
PMM1376	2	-0.31	0.96	0.68	0.64	Nucleoproteins	RNA-binding protein RbpD
PMM1377	2	1.21	0.16	0.11	0.20	Other	possible dihydroflavonol-4-reductase (maize, petunia, tomato)...
PMM1382	2	1.11	0.56	0.40	0.27	Fatty acid, phospholipid and sterol metabolism	fatty acid desaturase, type 2
PMM1383	2	1.60	0.78	0.55	0.08	Conserved hypothetical protein	conserved hypothetical protein
PMM1384	2	3.00	0.06	0.04	0.00	Adaptations and atypical conditions	possible high light inducible protein
PMM1385	2	2.89	0.09	0.06	0.01	Adaptations and atypical conditions	possible high light inducible protein
PMM1387	2	1.02	1.86	1.32	0.43	Conserved hypothetical protein	hypothetical
PMM1388	2	1.93	1.72	1.21	0.01	Conserved hypothetical protein	conserved hypothetical protein
PMM1390	2	4.01	0.83	0.59	0.00	Adaptations and atypical conditions	possible high light inducible protein
PMM1391	2	0.04	0.25	0.18	1.00	Regulatory functions	possible Helix-turn-helix protein, copG family
PMM1392	2	1.40	1.70	1.21	0.33	Branched chain family	possible Heat-labile enterotoxin alpha chain
PMM1394	2	-0.25	1.00	0.71	0.61	Conserved hypothetical protein	hypothetical
PMM1395	2	2.15	0.77	0.55	0.00	Conserved hypothetical protein	hypothetical
PMM1396	2	3.09	0.41	0.29	0.00	Adaptations and atypical conditions	possible high light inducible protein
PMM1397	2	3.91	0.14	0.10	0.00	Adaptations and atypical conditions	possible high light inducible protein
PMM1398	2	3.56	0.15	0.11	0.00	Adaptations and atypical conditions	possible high light inducible protein
PMM1399	2	3.99	0.63	0.44	0.00	Adaptations and atypical conditions	possible high light inducible protein
PMM1400	2	-1.52	0.62	0.44	0.60	Other	possible Hemagglutinin-neuraminidase
PMM1401	2	-2.27	0.04	0.03	0.00	Conserved hypothetical protein	conserved hypothetical
PMM1402	2	-1.52	0.57	0.40	0.17	Conserved hypothetical protein	unnamed protein product
PMM1404	2	5.09	0.38	0.27	0.00	Adaptations and atypical conditions	possible high light inducible protein
PMM1405	2	0.65	0.69	0.48	0.66	Conserved hypothetical protein	hypothetical
PMM1407	2	0.90	0.26	0.18	0.31	Other	possible SRP19 protein
PMM1408	2	0.06	0.15	0.10	0.65	Conserved hypothetical protein	hypothetical
PMM1409	2	3.07	0.30	0.21	0.00	Other	possible Rubredoxin
PMM1412	2	2.86	1.28	0.91	0.00	Conserved hypothetical protein	conserved hypothetical protein
PMM1413	2	1.24	1.32	0.93	0.32	Conserved hypothetical protein	conserved hypothetical
PMM1416	2	0.43	0.10	0.07	0.88	Conserved hypothetical protein	conserved hypothetical
PMM1419	2	-0.79	0.83	0.58	0.31	Transport and binding proteins	possible ATP synthase B/B' CF(0)
PMM1422	2	1.16	0.22	0.15	0.22	Conserved hypothetical protein	conserved hypothetical protein
PMM1423	2	0.79	0.76	0.54	0.56	Conserved hypothetical protein	conserved hypothetical protein
PMM1424	2	0.43	0.28	0.20	1.00	Other	possible Uncharacterized protein family UPF003
PMM1427	2	3.23	0.33	0.23	0.00	Conserved hypothetical protein	conserved hypothetical
PMM1428	2	1.17	2.48	1.75	0.84	Conserved hypothetical protein	conserved hypothetical protein
PMM1429	2	1.50	0.59	0.42	0.10	Conserved hypothetical protein	conserved hypothetical protein
PMM1430	2	0.33	0.97	0.68	0.66	Conserved hypothetical protein	conserved hypothetical protein
PMM1431	2	-0.02	0.23	0.16	0.67	RNA synthesis, modification, and DNA transcription	putative DNA-directed RNA polymerase (omega chain)
PMM1434	2	0.78	0.30	0.21	1.00	Glycolysis	Phosphoglycerate mutase, co-factor-independent (iPGM)
PMM1435	2	1.03	0.08	0.06	0.33	Conserved hypothetical protein	conserved hypothetical protein
PMM1436	2	-1.86	0.30	0.21	0.07	Chaperones	GroEL protein (Chaperonin cpn60)
PMM1437	2	-2.01	0.22	0.15	0.03	Chaperones	GroES protein (Chaperonin cpn10)
PMM1438	2	-3.18	0.88	0.62	0.00	ATP synthase	ATP synthase beta subunit, central region:ATP synth...
PMM1440	2	-1.68	0.34	0.24	0.01	Conserved hypothetical protein	conserved hypothetical protein
PMM1441	2	-1.90	1.62	1.15	0.00	Conserved hypothetical protein	hypothetical

PMM1442	2	1.73	0.59	0.42	0.04	Regulatory functions	putative aminopeptidase P
PMM1443	2	0.46	0.56	0.39	0.93	Conserved hypothetical protein	Domain of unknown function DUF21
PMM1449	2	1.41	0.96	0.68	0.09	Soluble electron carriers	ferredoxin
PMM1451	2	-3.17	0.32	0.23	0.00	ATP synthase	ATP synthase alpha subunit, central region:ATP synth...
PMM1452	2	-4.69	0.60	0.43	0.00	ATP synthase	ATP synthase, delta (OSCP) subunit
PMM1453	2	-5.56	0.25	0.17	0.00	ATP synthase	ATP synthase B/B' CF(0)
PMM1454	2	-4.92	0.56	0.40	0.00	ATP synthase	ATP synthase B/B' CF(0)
PMM1455	2	-4.35	0.18	0.13	0.00	Membranes, lipoproteins and porins	Eubacterial and plasma membrane ATP synthase subunit C:ATP sy...
PMM1456	2	-2.29	0.44	0.31	0.00	ATP synthase	ATP synthase A subunit
PMM1457	2	-3.62	0.24	0.17	0.00	Conserved hypothetical protein	possible ATP synthase subunit 1
PMM1459	2	0.36	0.55	0.39	1.00	Regulatory functions	putative c-type cytochrome biogenesis protein CcdA
PMM1462	2	-0.10	0.44	0.31	1.00	Conserved hypothetical protein	conserved hypothetical protein
PMM1463	2	0.69	0.67	0.47	0.56	Other	Nitrogen regulatory protein P-II
PMM1477	2	2.42	0.98	0.70	0.00	Conserved hypothetical protein	conserved hypothetical
PMM1478	2	1.15	0.48	0.34	0.39	Conserved hypothetical protein	conserved hypothetical protein
PMM1479	2	0.98	0.45	0.32	1.00	Conserved hypothetical protein	conserved hypothetical protein
PMM1480	2	1.00	0.26	0.19	0.24	Conserved hypothetical protein	conserved hypothetical protein
PMM1482	2	-1.82	0.80	0.57	0.23	Adaptations and atypical conditions	possible high light inducible protein
PMM1483	2	-0.46	0.42	0.30	0.33	RNA synthesis, modification, and DNA transcription	RNA polymerase beta prime subunit
PMM1484	2	0.15	0.03	0.02	0.66	RNA synthesis, modification, and DNA transcription	RNA polymerase gamma subunit
PMM1485	2	0.21	0.49	0.35	0.88	RNA synthesis, modification, and DNA transcription	RNA polymerase beta subunit
PMM1487	2	-0.97	1.52	1.07	0.17	Ribosomal proteins	30s Ribosomal protein S20
PMM1489	2	0.42	0.56	0.40	1.00	Other	Ribose 5-phosphate isomerase
PMM1490	2	2.79	0.39	0.28	0.00	Degradation of proteins, peptides, and glycopeptides	Serine proteases, trypsin family:Chymotrypsin serine protease...
PMM1492	2	-0.07	0.21	0.15	0.51	RNA synthesis, modification, and DNA transcription	N utilization substance protein A
PMM1494	2	-0.02	0.89	0.63	0.72	Protein modification and translation factors	Translation initiation factor IF-2
PMM1495	2	-0.69	1.26	0.89	0.55	Conserved hypothetical protein	hypothetical
PMM1498	2	1.35	0.42	0.29	0.33	Conserved hypothetical protein	conserved hypothetical protein
PMM1499	2	0.96	0.07	0.05	0.29	Conserved hypothetical protein	conserved hypothetical protein
PMM1500	2	1.03	0.14	0.10	0.36	Regulatory functions	putative aminotransferase
PMM1501	2	-0.37	0.39	0.27	0.39	Other	S1 RNA binding domain:Ribonuclease E and G
PMM1504	2	-0.84	0.77	0.54	0.21	Aromatic amino acid family	Chorismate mutase-Prephenate dehydratase
PMM1506	2	-0.89	0.18	0.13	0.25	Regulatory functions	ATP-dependent protease La (LON) domain
PMM1507	2	-2.01	0.43	0.30	0.00	Ribosomal proteins	30S ribosomal protein S10
PMM1508	2	-0.17	0.39	0.28	0.78	Protein modification and translation factors	Elongation factor Tu
PMM1509	2	0.03	0.08	0.06	0.84	Protein modification and translation factors	Elongation factor G
PMM1510	2	-0.19	0.55	0.39	0.58	Ribosomal proteins	30S ribosomal protein S7
PMM1511	2	-3.29	0.10	0.07	0.00	Ribosomal proteins	30S ribosomal protein S12
PMM1512	2	0.94	0.35	0.25	0.40	Glutamate family / Nitrogen assimilation	Ferredoxin-dependent glutamate synthase, Fd-GOGAT
PMM1514	2	0.74	1.74	1.23	0.87	Other	lipoic acid synthetase
PMM1515	2	0.05	0.88	0.62	1.00	Other	Site-specific recombinase
PMM1519	2	-2.00	0.02	0.01	0.00	Photosystem I	Photosystem I PsaL protein (subunit XI)
PMM1520	2	-2.68	0.84	0.59	0.00	Photosystem I	photosystem I subunit VIII (PsaI)
PMM1523	2	-1.56	0.56	0.40	0.02	Photosystem I	Photosystem I PsaB protein
PMM1524	2	-0.59	0.20	0.14	0.94	Photosystem I	Photosystem I PsaA protein
PMM1528	2	0.17	0.46	0.33	1.00	DNA replication, recombination, and repair	HNH endonuclease family protein
PMM1530	2	-1.07	0.14	0.10	0.15	Ribosomal proteins	50S ribosomal protein L31
PMM1531	2	-0.87	0.40	0.28	1.00	Ribosomal proteins	30S ribosomal protein S9
PMM1532	2	-2.98	0.01	0.00	0.00	Ribosomal proteins	50S ribosomal protein L13
PMM1534	2	-1.33	0.27	0.19	0.02	Ribosomal proteins	50S ribosomal protein L17
PMM1535	2	-0.84	0.50	0.35	0.21	RNA synthesis, modification, and DNA transcription	Bacterial RNA polymerase, alpha chain
PMM1536	2	-3.01	0.15	0.11	0.00	Ribosomal proteins	30S ribosomal protein S11
PMM1537	2	-4.16	0.29	0.20	0.00	Ribosomal proteins	30S ribosomal protein S13
PMM1538	2	-4.17	0.82	0.58	0.00	Ribosomal proteins	50S Ribosomal protein L36
PMM1540	2	-0.70	0.18	0.13	0.36	Protein and peptide secretion	preprotein translocase SecY subunit
PMM1541	2	-3.24	0.81	0.57	0.00	Ribosomal proteins	50S ribosomal protein L15
PMM1542	2	-2.83	0.02	0.02	0.00	Ribosomal proteins	30S ribosomal protein S5
PMM1543	2	-0.99	0.77	0.55	0.27	Ribosomal proteins	50S ribosomal protein L18
PMM1544	2	-1.87	0.36	0.25	0.00	Ribosomal proteins	50S ribosomal protein L6
PMM1545	2	-1.00	0.42	0.30	0.21	Ribosomal proteins	30S ribosomal protein S8
PMM1546	2	-0.19	0.90	0.63	0.51	Ribosomal proteins	50S ribosomal protein L5
PMM1548	2	-0.36	0.43	0.30	0.69	Ribosomal proteins	50S Ribosomal protein L14
PMM1549	2	#NAME?	NA	NA	0.07	Ribosomal proteins	30S Ribosomal protein S17
PMM1550	2	-1.93	0.75	0.53	0.13	Ribosomal proteins	50S ribosomal protein L29
PMM1551	2	-1.02	0.04	0.03	0.19	Ribosomal proteins	50S ribosomal protein L16
PMM1552	2	-2.56	0.06	0.04	0.00	Ribosomal proteins	30S ribosomal protein S3
PMM1553	2	-2.42	0.76	0.54	0.00	Ribosomal proteins	50S ribosomal protein L22
PMM1554	2	-2.15	0.69	0.49	0.00	Ribosomal proteins	30S Ribosomal protein S19
PMM1555	2	-2.54	0.59	0.42	0.00	Ribosomal proteins	50S ribosomal protein L2
PMM1556	2	-2.11	0.03	0.02	0.00	Ribosomal proteins	50S ribosomal protein L23
PMM1557	2	-1.14	0.29	0.21	0.13	Ribosomal proteins	50S ribosomal protein L4
PMM1558	2	-4.05	1.61	1.14	0.00	Ribosomal proteins	50S ribosomal protein L3
PMM1559	2	0.68	0.44	0.31	0.65	Conserved hypothetical protein	conserved hypothetical protein
PMM1562	2	3.38	0.66	0.47	0.00	DNA replication, recombination, and repair	RecA bacterial DNA recombination protein
PMM1563	2	-1.20	0.71	0.50	1.00	Conserved hypothetical protein	conserved hypothetical protein
PMM1566	2	0.33	0.33	0.23	1.00	Conserved hypothetical protein	conserved hypothetical protein
PMM1568	2	0.07	0.10	0.07	0.56	Conserved hypothetical protein	conserved hypothetical protein
PMM1570	2	-1.16	0.23	0.16	0.16	Cobalamin, heme, phycobilin and porphyrin	ATP:corrinoid adenosyltransferase BtuR/CobO/CobP
PMM1571	2	1.08	0.95	0.67	0.51	Conserved hypothetical protein	conserved hypothetical protein
PMM1575	2	0.51	0.06	0.04	1.00	Pyruvate and acetyl-CoA metabolism	Phosphoenolpyruvate carboxylase
PMM1578	2	-1.59	0.46	0.32	0.03	Photosystem I	Photosystem I protein PsaD
PMM1581	2	0.63	0.05	0.04	0.77	Regulatory functions	MRP protein homolog
PMM1583	2	0.53	0.07	0.05	0.94	Conserved hypothetical protein	conserved hypothetical protein
PMM1585	2	-1.67	0.21	0.15	0.08	Conserved hypothetical protein	conserved hypothetical protein
PMM1588	2	1.70	0.27	0.19	0.08	Regulatory functions	possible Conserved carboxylase domain
PMM1594	2	0.62	0.21	0.15	1.00	Cobalamin, heme, phycobilin and porphyrin	Heme oxygenase
PMM1596	2	-0.25	0.71	0.50	0.52	Other	Isocitrate dehydrogenase
PMM1599	2	-0.17	0.08	0.06	0.61	Conserved hypothetical protein	conserved hypothetical protein
PMM1600	2	0.74	0.33	0.24	0.97	Transport and binding proteins	putative Na+/H+ antiporter, CPA2 family
PMM1601	2	2.14	0.09	0.06	0.01	Other	phosphorylase
PMM1602	2	1.45	0.33	0.23	0.19	Conserved hypothetical protein	conserved hypothetical protein
PMM1603	2	2.15	1.32	0.93	0.00	Regulatory functions	putative ribonuclease III
PMM1604	2	-1.43	0.91	0.64	0.02	Conserved hypothetical protein	conserved hypothetical protein
PMM1605	2	1.89	1.01	0.71	0.05	Protein modification and translation factors	possible 16S rRNA processing protein RimM
PMM1606	2	-0.50	0.40	0.28	0.36	Other	Glutamine--fructose-6-phosphate transaminase (isomerizing)
PMM1607	2	-2.05	0.82	0.58	0.00	Photosystem I	Photosystem I subunit PsaC
PMM1608	2	-3.46	0.83	0.59	0.00	Fatty acid, phospholipid and sterol metabolism	acyl carrier protein (ACP)
PMM1609	2	-3.09	0.47	0.33	0.00	Fatty acid, phospholipid and sterol metabolism	3-oxoacyl-[acyl-carrier-protein] synthase II
PMM1610	2	-1.86	0.70	0.49	0.00	Other	Transketolase
PMM1611	2	1.65	0.15	0.11	0.15	Thiamine	ThiC family
PMM1613	2	0.92	0.23	0.17	0.40	Other	Zinc metallopeptidase M20/M25/M40 family
PMM1615	2	2.15	1.06	0.75	0.01	DNA replication, recombination, and repair	Holliday junction DNA helicase RuvB
PMM1616	2	0.80	0.39	0.28	0.45	Other	tmRNA binding protein SmpB
PMM1617	2	-1.28	0.12	0.08	0.50	Conserved hypothetical protein	conserved hypothetical protein
PMM1619	2	-0.29	0.27	0.19	0.82	Regulatory functions	two-component response regulator
PMM1622	2	1.39	0.48	0.34	0.15	Murein sacculus and peptidoglycan	Rod shape determining protein
PMM1623	2	3.08	0.94	0.67	0.00	DNA replication, recombination, and repair	single-stranded DNA-binding protein
PMM1625	2	0.64	0.40	0.28	1.00	Amino acids and amines	putative adenosylhomocysteinase
PMM1626	2	2.25	0.32	0.23	0.00	Conserved hypothetical protein	conserved hypothetical protein
PMM1629	2	1.20	0.00	0.00	0.21	Transport and binding proteins	Type II alternative RNA polymerase sigma factor, sigma-70 family
PMM1630	2	-0.14	0.05	0.03	0.77	Transport and binding proteins	MgtE family, putative magnesium transport protein
PMM1634	2	1.25	0.85	0.60	0.28	DNA replication, recombination, and repair	DNA gyrase, subunit B
PMM1635	2	-0.23	0.67	0.47	0.50	Aminoacyl tRNA synthetases and tRNA modification	tRNA delta-2-isopentenylpyrophosphate (IPP) transferase
PMM1636	2	-0.60	0.58	0.41	0.89	Protein modification and translation factors	Translation Initiation factor 3
PMM1639	2	-0.17	0.42	0.29	0.51	Protein and peptide secretion	Preprotein translocase SecA subunit
PMM1640	2	0.23	1.20	0.85	1.00	Regulatory functions	putative acetyltransferase, GNAT family
PMM1642	2	0.15	0.24	0.17	1.00	Regulatory functions	possible transcription regulator
PMM1643	2	-1.75	0.85	0.60	0.00	Riboflavin	Putative 6,7-dimethyl-8-ribityllumazine synthase or riboflavin synthase beta chain

PMM1644	2	-3.40	1.11	0.78	0.00	Photosystem II	possible Photosystem II reaction center Z protein (PsbZ)
PMM1648	2	0.28	0.08	0.06	0.78	Branched chain family	Aspartate kinase
PMM1649	2	0.32	0.29	0.20	1.00	DNA replication, recombination, and repair	Excinuclease ABC subunit B (UvrB)
PMM1650	2	-1.45	0.12	0.09	0.03	Conserved hypothetical protein	conserved hypothetical protein
PMM1652	2	0.55	0.43	0.30	1.00	Conserved hypothetical protein	conserved hypothetical protein
PMM1653	2	0.37	0.21	0.15	0.88	Aspartate family	Dihydrodipicolinate synthetase
PMM1655	2	-1.37	1.55	1.09	0.01	Other	FKBP-type peptidyl-prolyl cis-trans isomerase (PPIase)
PMM1656	2	-0.28	2.12	1.50	0.20	Degradation of proteins, peptides, and glycopeptides	Clp protease proteolytic subunit
PMM1657	2	-0.82	0.34	0.24	0.15	Degradation of proteins, peptides, and glycopeptides	Clp protease ATP-binding subunit, ClpX
PMM1661	2	-2.81	1.16	0.82	0.00	Ribosomal proteins	50S ribosomal protein L35
PMM1662	2	-2.32	1.34	0.94	0.00	Ribosomal proteins	50S ribosomal protein L20
PMM1665	2	-1.72	0.13	0.09	0.00	Fatty acid, phospholipid and sterol metabolism	sulfolipid (UDP-sulfoquinovose) biosynthesis protein
PMM1667	2	0.69	0.39	0.28	0.71	Conserved hypothetical protein	conserved hypothetical protein
PMM1669	2	2.25	0.94	0.67	0.00	Regulatory functions	putative Glycine cleavage H-protein
PMM1671	2	2.53	0.94	0.66	0.00	Conserved hypothetical protein	conserved hypothetical protein
PMM1672	2	2.04	0.51	0.36	0.01	Fatty acid, phospholipid and sterol metabolism	Fatty acid desaturase, type 1
PMM1673	2	-0.32	0.70	0.49	0.53	Ribosomal proteins	50S ribosomal protein L9
PMM1676	2	0.94	1.22	0.86	0.73	Conserved hypothetical protein	conserved hypothetical protein
PMM1678	2	0.96	0.53	0.37	0.41	Conserved hypothetical protein	conserved hypothetical protein
PMM1680	2	3.09	0.94	0.66	0.00	Conserved hypothetical protein	conserved hypothetical protein
PMM1683	2	-1.16	0.28	0.20	0.10	Conserved hypothetical protein	conserved hypothetical
PMM1685	2	1.94	0.13	0.09	0.00	Regulatory functions	putative spermidine synthase
PMM1688	2	0.00	0.14	0.10	0.65	Aminoacyl tRNA synthetases and tRNA modification	Aspartyl-tRNA synthetase
PMM1689	2	1.44	0.24	0.17	0.15	Pyrimidine ribonucleotide biosynthesis	Glutamine amidotransferase class-I:CTP synthase
PMM1694	2	1.50	0.62	0.44	0.06	Cobalamin, heme, phycobilin and porphyrin	putative uroporphyrin-III C-methyltransferase
PMM1697	2	-0.69	0.48	0.34	0.37	Transport and binding proteins	Type II alternative RNA polymerase sigma factor, sigma-70 family
PMM1700	2	3.80	0.42	0.30	0.00	Other	Aconitate hydratase B
PMM1702	2	-0.68	0.07	0.05	0.25	Purine ribonucleotide biosynthesis	formyltetrahydrofolate deformylase
PMM1703	2	1.45	0.05	0.04	0.07	Transport and binding proteins	NAD binding site:D-amino acid oxidase
PMM1704	2	-1.72	0.74	0.52	0.13	Chaperones	Molecular chaperone DnaK2, heat shock protein hsp70-2
PMM1706	2	-2.20	2.23	1.58	0.00	Ribosomal proteins	30S ribosomal protein S6
PMM1707	2	0.62	0.76	0.54	0.88	Glutamate family / Nitrogen assimilation	Argininosuccinate synthase
PMM1708	2	0.12	0.65	0.46	0.79	Conserved hypothetical protein	conserved hypothetical protein
PMM1716	2	0.86	0.82	0.58	0.39	Aspartate family	No Cyanobase Name
PMM1717	2	-3.05	0.42	0.30	0.00	Other	No Cyanobase Name
PMM1718	2	-0.73	0.89	0.63	0.76	Other	No Cyanobase Name
