# Supplementary material for: Nitrogen cost minimization is promoted by structural changes in the transcriptome of N-deprived Prochlorococcus cells
Source: ISME J. 2017 Jun 6;11(10):2267–78. doi: 10.1038/ismej.2017.88 (PMC5607370; doi:10.1038/ismej.2017.88)
Supplement: Supplementary Table 6 [file ismej201788x13.pdf]

Table S6. Transcripts Described During 12 and 24 Hours Post Starvation

Name	Log2 Fold Change (3hr)	Standard Error (3hr)	p-value (3hr)	Log2 Fold Change (12hr)	Standard Error (12hr)	p-value (12hr)	Log2 Fold Change (24hr)	Standard Error (24hr)	p-value (24hr)	Category	MIT Annotation
PMM1314	1.82	0.47	3.89E-01	2.91	0.59	1.15E-09	3	0.56	1.44E-11	Degradation of proteins, peptides, and glycopeptides	clpP3 Clp protease proteolytic subunit
PMM1490	-1.38	0.38	3.05E-01	2.1	0.1	9.20E-03	2.79	0.28	1.64E-08	Degradation of proteins, peptides, and glycopeptides	Serine proteases, trypsin family:Chymotrypsinserine protease
PMM1623	0.7	0.08	1.00E+00	3.39	0.05	8.01E-17	3.08	0.67	6.63E-10	DNA replication, recombination, and repair	ssb single-stranded DNA-binding protein
PMM1562	-1.32	0.63	1.00E+00	2.54	0.16	1.79E-02	3.38	0.47	2.40E-15	DNA replication, recombination, and repair	recA RecA bacterial DNA recombination protein
PMM1615	-2.79	0.07	2.95E-07	2.44	0.1	2.30E-04	2.15	0.75	1.34E-02	DNA replication, recombination, and repair	ruvB Holliday junction DNA helicase RuvB
PMM1262	2.37	0.36	1.82E-02	1.33	0.36	6.37E-01	2.02	0.16	1.93E-02	Regulatory functions	SOS function regulatory protein, LexA repressor
PMM0965	-1.57	0.89	1.36E-01	2.6	0.25	5.45E-05	3.08	0.51	5.73E-12	N metabolism	ureA Urease gamma subunit
PMM0964	-1.82	0.06	1.01E-01	2.37	1.21	5.28E-03	2.33	0.1	7.76E-06	N metabolism	ureB Urease beta subunit
PMM0969	-1.85	0.72	1.04E-01	1.75	1.41	2.17E-02	1.85	0.66	4.57E-02	N metabolism	ureG urease accessory protein UreG
PMM0223	-1.77	0	1.00E+00	2.84	0.15	8.31E-02	2.47	0.26	6.00E-05	Photosystem II	Photosystem II PsbA protein (D1)
PMM0550	2.93	0.5	4.41E-05	-1.48	0.14	5.18E-03	-1.28	0.16	1.09E-01	CO2 fixation	rbcL, cbbL Ribulose bisphosphate carboxylase, large chain
PMM0551	1.86	1.22	9.56E-07	-1.09	0.45	2.50E-01	-1.24	0.01	8.99E-02	CO2 fixation	rbcS, cbbS Ribulose bisphosphate carboxylase, Small chain
PMM0468	0.95	0.37	1.00E+00	-3.46	1.3	1.10E-23	-2.93	0.23	1.83E-18	Photosystem I	Photosystem I PsaJ protein (subunit IX)
PMM0540	3.64	0.58	1.25E-23	-3.39	1.23	3.26E-05	-3.57	1.11	1.49E-25	Photosystem I	psaM possible photosystem I reaction centre subunitXII (PsaM)
PMM0329	0.92	0.17	1.00E+00	-2.52	0.09	4.20E-10	-2.13	0.23	1.63E-07	Photosystem I	psaE Photosystem I PsaE protein (subunit IV)
PMM0469	-0.05	0.47	1.00E+00	-2.4	0.4	1.30E-10	-3.27	0.21	8.01E-32	Photosystem I	psaF Photosystem I PsaF protein (subunit III)
PMM0272	-0.8	0.39	1.00E+00	-3.34	0.47	3.46E-36	-4.18	0.77	3.06E-153	Photosystem II	Photosystem II protein PsbK
PMM0251	-0.83	0.42	1.00E+00	-2.46	0.06	1.63E-09	-2.79	0.18	1.84E-17	Photosystem II	psbH Photosystem II PsbH protein
PMM1644	-0.24	0.51	1.00E+00	-2.09	0.92	1.84E-05	-3.4	0.78	1.56E-47	Photosystem II	psbZ possible Photosystem II reaction center Z protein(PsbZ)
