# Supplementary material for: Nitrogen cost minimization is promoted by structural changes in the transcriptome of N-deprived Prochlorococcus cells
Source: ISME J. 2017 Jun 6;11(10):2267–78. doi: 10.1038/ismej.2017.88 (PMC5607370; doi:10.1038/ismej.2017.88)
Supplement: Supplementary Table 7 [file ismej201788x14.pdf]

Table S7. High Light Inducible Protein 12 Hour Fold Changes

Name	Log2 Fold Change	Standard Error	p-value	Category	MIT Annotation
**PMM0690**	Inf	NA	0.00E+00	Adaptations	hli21 possible high light inducible protein
PMM0815	4.86	0.53	3.51E-163	Adaptations	hli19 possible high light inducible protein
PMM1404	4.57	0.14	3.15E-84	Adaptations	hli5 possible high light inducible protein
**PMM1390**	4.81	0.89	2.27E-93	Adaptations	hli10 possible high light inducible protein
PMM0816	4.64	1.32	2.55E-90	Adaptations	hli18 possible high light inducible protein
PMM1396	4.17	0.14	1.24E-19	Adaptations	hli9 possible high light inducible protein
**PMM0689**	4.28	1.32	4.68E-44	Adaptations	hli22 possible high light inducible protein
PMM1397	3.91	0.26	9.63E-59	Adaptations	hli8 possible high light inducible protein
PMM1385	3.72	0.41	6.12E-06	Adaptations	hli11 possible high light inducible protein
PMM1398	3.64	0.91	1.13E-20	Adaptations	hli7 possible high light inducible protein
PMM1384	3.47	0.22	3.19E-07	Adaptations	hli12 possible high light inducible protein
PMM1399	3.36	0.54	1.35E-18	Adaptations	hli6 possible high light inducible protein
PMM0817	3.14	0.44	3.74E-16	Adaptations	hli17 possible high light inducible protein
PMM0818	2.92	0.55	3.61E-08	Adaptations	hli16 possible high light inducible protein
PMM1135	2.58	0.48	1.64E-04	Adaptations	hli14 possible high light inducible protein

Note those rows that are in bold and labeled with an * were identified as differentially expressed in the Tolonen et. al 2006 paper
