# Supplementary material for: Nitrogen cost minimization is promoted by structural changes in the transcriptome of N-deprived Prochlorococcus cells
Source: ISME J. 2017 Jun 6;11(10):2267–78. doi: 10.1038/ismej.2017.88 (PMC5607370; doi:10.1038/ismej.2017.88)
Supplement: Supplementary Table 8 [file ismej201788x15.pdf]

Table S8. N-Replete Transcriptional Start Sites at 12 Hours Post Starvation Identified by TSSAR.

Position	Strand	ID	Score	Difference	p-Value	Positional (	Class	Comment
158	+	TSS_000004	1000	744	0	5	P	16nt upstream of gene PMM0001;
1089	+	TSS_000013	1000	100	0	9	IP	within gene(s) PMM0001; 244nt upstream of gene PMM0002;
1991	+	TSS_000015	1000	172	0	4	IP	within gene(s) PMM0002; 53nt upstream of gene PMM0003;
2084	+	TSS_000019	1000	2221	0	2	I	within gene(s) PMM0003;
2919	-	TSS_009685	1000	52	0	1	Ai	antisense to gene(s) PMM0003;
3197	+	TSS_000026	1000	138	0	5	I	within gene(s) PMM0003;
8361	-	TSS_009699	1000	614	0	0	P	33nt upstream of gene PMM0005;
9568	+	TSS_000040	1000	184	0	5	Ai	antisense to gene(s) PMM0007;
10218	-	TSS_009703	1000	460	0	0	I	within gene(s) PMM0007;
10339	+	TSS_000043	1000	1555	0	4	P	28nt upstream of gene PMM0008;
11776	+	TSS_000054	1000	162	0	4	P	17nt upstream of gene PMM0010;
12361	+	TSS_000061	1000	152	0	0	I	within gene(s) PMM0010;
14116	-	TSS_009714	1000	1451	0	2	Ai	antisense to gene(s) PMM0011;
14141	+	TSS_000072	1000	65	0	0	I	within gene(s) PMM0011;
14568	+	TSS_000073	1000	531	0	1	P	17nt upstream of gene PMM0012;
14769	-	TSS_009717	1000	133	0	0	Ai	antisense to gene(s) PMM0012;
14964	-	TSS_009718	1000	536	0	6	Ai	antisense to gene(s) PMM0012;
15347	+	TSS_000080	1000	83	0	0	I	within gene(s) PMM0012;
16016	+	TSS_000083	1000	27455	0	3	P	2nt upstream of gene PMM0013;
16255	-	TSS_009725	1000	176	0	1	Ai	antisense to gene(s) PMM0013;
17671	+	TSS_000101	1000	471	0	2	P	41nt upstream of gene PMM0015;
18267	+	TSS_000109	1000	1171	0	4	P	16nt upstream of gene PMM0016;
20649	+	TSS_000146	1000	44	0	2	I	within gene(s) PMM0019;
23179	-	TSS_009753	1000	110	0	0	I	within gene(s) PMM0022;
24122	+	TSS_000151	1000	1553	0	3	P	28nt upstream of gene PMM0023;
24575	-	TSS_009757	1000	103	0	0	Ai	antisense to gene(s) PMM0023;
24586	-	TSS_009758	1000	52	0	0	Ai	antisense to gene(s) PMM0023;
25810	-	TSS_009768	1000	51	0	0	I	within gene(s) PMM0024;
25886	-	TSS_009770	1000	1299	0	1	I	within gene(s) PMM0024;
26468	-	TSS_009772	1000	258	0	0	I	within gene(s) PMM0025;
27201	+	TSS_000176	1000	72	0	0	P	86nt upstream of gene PMM0026;
27273	+	TSS_000177	1000	2551	0	2	P	14nt upstream of gene PMM0026;
27367	+	TSS_000180	1000	999	0	0	I	within gene(s) PMM0026;
27478	-	TSS_009780	1000	399	0	1	Ai	235nt upstream of gene PMM0025; antisense to gene(s) PMM0026;
27552	+	TSS_000183	1000	93	0	5	I	within gene(s) PMM0026;
27755	+	TSS_000188	1000	784	0	0	IP	within gene(s) PMM0026; 92nt upstream of gene PMM0027;
30271	+	TSS_000196	1000	109	0	2	I	within gene(s) PMM0030;
30946	-	TSS_009795	1000	293	0	2	P	16nt upstream of gene PMM0031;
31393	-	TSS_009806	1000	361	0	2	I	within gene(s) PMM0032;
31536	-	TSS_009812	1000	8798	0	2	P	16nt upstream of gene PMM0032;
31640	+	TSS_000206	1000	307	0	1	Ai	antisense to gene(s) PMM0033;
31718	+	TSS_000208	1000	99	0	0	Ai	antisense to gene(s) PMM0033;
32109	-	TSS_009817	1000	90	0	1	P	18nt upstream of gene PMM0033;
32317	+	TSS_000281	1000	3951	0	4	P	18nt upstream of gene PMM0034;
32374	+	TSS_000209	1000	87	0	0	I	within gene(s) PMM0034;
33332	+	TSS_000214	1000	288	0	1	Ai	antisense to gene(s) PMM0035;
33713	-	TSS_009835	1000	2238	0	2	P	16nt upstream of gene PMM0035;
34926	+	TSS_000220	1000	446	0	0	I	within gene(s) PMM0037;
37053	-	TSS_009843	1000	140	0	0	Ai	antisense to gene(s) PMM0038;
37342	+	TSS_000234	1000	949	0	3	P	18nt upstream of gene PMM0039;
41761	-	TSS_009854	1000	99	0	0	I	within gene(s) PMM0042;
43346	+	TSS_000247	1000	245	0	0	Ai	antisense to gene(s) PMM0043;
44029	+	TSS_000249	1000	671	0	1	Ai	antisense to gene(s) PMM0043;
44220	-	TSS_009872	1000	1036	0	0	P	30nt upstream of gene PMM0043;
44838	-	TSS_009876	1000	300	0	2	Ai	antisense to gene(s) PMM0044;
47672	-	TSS_009885	1000	155	0	5	I	within gene(s) PMM0045;
47817	+	TSS_000268	1000	31	0	1	Ai	antisense to gene(s) PMM0045;
47824	+	TSS_000363	1000	4286	0	4	Ai	antisense to gene(s) PMM0045;
49002	+	TSS_000275	1000	2951	0	3	P	15nt upstream of gene PMM0046;
49816	-	TSS_009890	1000	79	0	0	I	within gene(s) PMM0047;
50616	+	TSS_000283	1000	577	0	0	P	56nt upstream of gene PMM0048;
52823	+	TSS_000305	1000	366	0	5	P	44nt upstream of gene PMM0050;
52948	-	TSS_009900	1000	252	0	1	PAi	186nt upstream of gene PMM0049; antisense to gene(s) PMM0050;
54377	-	TSS_009912	1000	510	0	36	I	within gene(s) PMM0051;
54638	+	TSS_000315	1000	107	0	0	Ai	antisense to gene(s) PMM0051;
55050	+	TSS_000316	1000	105	0	0	P	16nt upstream of gene PMM0052;
56405	+	TSS_000317	1000	127	0	1	O	-
56601	-	TSS_009928	1000	2687	0	0	O	-
58622	-	TSS_014433	1000	200	0	3	I	within gene(s) PMM0055;
58796	+	TSS_000323	1000	7021	0	1	P	207nt upstream of gene PMM0056;
59479	+	TSS_000328	1000	1068	0	1	P	17nt upstream of gene PMM0057;
59995	-	TSS_009950	1000	92	0	3	Ai	antisense to gene(s) PMM0057;
61132	+	TSS_000334	1000	67	0	2	I	within gene(s) PMM0057;
61620	-	TSS_009951	1000	71	0	3	Ai	antisense to gene(s) PMM0057;
63006	+	TSS_000337	1000	1431	0	1	IP	within gene(s) PMM0057; 126nt upstream of gene PMM0058;
63116	+	TSS_000339	1000	388	0	1	P	16nt upstream of gene PMM0058;
63461	+	TSS_000341	1000	131	0	1	I	within gene(s) PMM0058;
65570	-	TSS_009963	1000	153	0	3	P	121nt upstream of gene PMM0059;
65752	+	TSS_000347	1000	284	0	1	P	89nt upstream of gene PMM0060;

66139	+	TSS_000348	1000	39	0	0	I	within gene(s) PMM0060;
66151	+	TSS_000349	1000	141	0	0	I	within gene(s) PMM0060;
66155	-	TSS_009967	1000	21	0	0	Ai	antisense to gene(s) PMM0060;
66390	-	TSS_009969	1000	20	0	0	Ai	antisense to gene(s) PMM0060;
66518	+	TSS_000351	1000	178	0	2	I	within gene(s) PMM0060;
66815	+	TSS_000353	1000	102	0	0	I	within gene(s) PMM0060;
66972	-	TSS_009974	1000	99	0	2	Ai	antisense to gene(s) PMM0060;
67529	-	TSS_009976	1000	467	0	2	P	16nt upstream of gene PMM0061;
67586	+	TSS_000359	1000	3300	0	2	P	66nt upstream of gene PMM0062;
67893	+	TSS_000364	1000	547	0	6	I	within gene(s) PMM0063;
68237	+	TSS_000375	1000	208	0	3	I	within gene(s) PMM0063;
68394	-	TSS_009990	1000	94	0	0	Ai	antisense to gene(s) PMM0063;
68453	+	TSS_000389	1000	196	0	1	I	within gene(s) PMM0063;
69179	-	TSS_009999	1000	354	0	2	IP	within gene(s) PMM0065; 143nt upstream of gene PMM0064;
69361	-	TSS_010003	1000	117	0	0	I	within gene(s) PMM0065;
70887	-	TSS_010014	1000	75	0	0	I	within gene(s) PMM0065;
72400	-	TSS_010021	1000	168	0	0	P	32nt upstream of gene PMM0068;
72542	+	TSS_000416	1000	39	0	2	I	within gene(s) PMM0069;
73169	-	TSS_010023	1000	65	0	0	Ai	antisense to gene(s) PMM0069;
73910	-	TSS_010025	1000	129	0	0	Ai	antisense to gene(s) PMM0069;
74118	-	TSS_010027	1000	327	0	0	Ai	antisense to gene(s) PMM0069;
75013	+	TSS_000420	1000	70	0	0	Ai	antisense to gene(s) PMM0070;
78158	-	TSS_010048	1000	294	0	1	I	within gene(s) PMM0073;
78218	-	TSS_010050	1000	428	0	1	I	within gene(s) PMM0073;
78295	-	TSS_010052	1000	228	0	0	I	within gene(s) PMM0073;
78481	-	TSS_010056	1000	377	0	0	I	within gene(s) PMM0073;
79154	-	TSS_010069	1000	732	0	4	P	33nt upstream of gene PMM0073;
79823	+	TSS_000434	1000	69	0	0	P	22nt upstream of gene PMM0075;
80192	-	TSS_010074	1000	126	0	0	Ai	antisense to gene(s) PMM0075;
80289	+	TSS_000436	1000	106	0	0	I	within gene(s) PMM0075;
80352	+	TSS_000438	1000	127	0	3	I	within gene(s) PMM0075;
81157	+	TSS_000443	1000	77	0	0	P	14nt upstream of gene PMM0076;
82018	-	TSS_010082	1000	86	0	3	Ai	antisense to gene(s) PMM0076;
82229	-	TSS_010085	1000	198	0	0	Ai	antisense to gene(s) PMM0076;
83123	+	TSS_000448	1000	88	0	0	I	within gene(s) PMM0077;
86371	-	TSS_010094	1000	77	0	0	I	within gene(s) PMM0081;
88867	+	TSS_000462	1000	128	0	6	I	within gene(s) PMM0083;
89200	-	TSS_010103	1000	192	0	4	Ai	antisense to gene(s) PMM0083;
89701	-	TSS_010107	1000	70	0	0	Ai	antisense to gene(s) PMM0083;
90051	+	TSS_000469	1000	158	0	1	I	within gene(s) PMM0084;
91200	+	TSS_000478	1000	3458	0	7	IP	within gene(s) PMM0084; 60nt upstream of gene PMM0085;
91487	-	TSS_010116	1000	226	0	0	Ai	antisense to gene(s) PMM0085;
91506	+	TSS_000484	1000	99	0	0	I	within gene(s) PMM0085;
91711	-	TSS_010119	1000	600	0	1	Ai	antisense to gene(s) PMM0085;
92216	-	TSS_010120	1000	161	0	0	I	within gene(s) PMM0086;
92293	-	TSS_010121	1000	2236	0	1	P	20nt upstream of gene PMM0086;
92350	+	TSS_000490	1000	877	0	1	P	40nt upstream of gene PMM0087;
92747	+	TSS_000494	1000	1892	0	2	P	17nt upstream of gene PMM0088;
93143	-	TSS_010124	1000	624	0	2	O	-
94679	-	TSS_010125	1000	309	0	3	Ai	antisense to gene(s) PMM0089;
96428	+	TSS_000512	1000	261	0	2	P	24nt upstream of gene PMM0091;
97181	+	TSS_000526	1000	35340	0	10	P	19nt upstream of gene PMM0093;
98015	-	TSS_010136	1000	605	0	4	I	within gene(s) PMM0095;
98676	-	TSS_010143	1000	198	0	1	P	16nt upstream of gene PMM0095;
98732	+	TSS_000529	1000	15	7.5E-10	0	Ad	antisense to gene(s) PMM0096 (4nt downstream);
99177	+	TSS_000531	1000	59	0	0	Ai	antisense to gene(s) PMM0096;
101137	+	TSS_000534	1000	114	0	0	Ai	antisense to gene(s) PMM0098;
102827	+	TSS_000540	1000	71	0	3	I	within gene(s) PMM0099;
104291	+	TSS_000549	1000	72	0	0	I	within gene(s) PMM0100;
105240	-	TSS_010162	1000	95	0	0	I	within gene(s) PMM0101;
105437	-	TSS_010164	1000	316	0	3	I	within gene(s) PMM0101;
105586	-	TSS_010168	1000	62	0	3	I	within gene(s) PMM0101;
105798	-	TSS_010171	1000	82	0	2	I	within gene(s) PMM0101;
105946	-	TSS_010174	1000	618	0	2	P	30nt upstream of gene PMM0101;
106019	+	TSS_000552	1000	64	0	0	P	4nt upstream of gene PMM0102;
107782	-	TSS_010180	1000	94	0	1	I	within gene(s) PMM0103;
107836	-	TSS_010181	1000	148	0	0	I	within gene(s) PMM0103;
109596	+	TSS_000557	1000	56	0	0	Ai	antisense to gene(s) PMM0105;
109827	+	TSS_000558	1000	53	0	0	Ai	antisense to gene(s) PMM0105;
110281	+	TSS_000560	1000	1159	0	1	Ai	antisense to gene(s) PMM0106;
110766	-	TSS_010191	1000	15987	0	0	P	12nt upstream of gene PMM0106;
110958	+	TSS_000569	1000	52	0	0	I	within gene(s) PMM0107;
113037	+	TSS_000572	1000	68	0	0	P	46nt upstream of gene PMM0111;
114786	+	TSS_000576	1000	166	0	1	Ai	antisense to gene(s) PMM0114;
114981	-	TSS_010202	1000	91	0	0	IP	within gene(s) PMM0115; 35nt upstream of gene PMM0114;
115971	+	TSS_000583	1000	140	0	0	Ai	antisense to gene(s) PMM0115;
116412	-	TSS_010216	1000	52	0	7	P	9nt upstream of gene PMM0115;
116480	-	TSS_010218	1000	12000	0	0	P	20nt upstream of gene PMM0116;
118346	-	TSS_010221	1000	206	0	0	Ai	antisense to gene(s) PMM0118;
119698	-	TSS_010226	1000	202	0	2	P	16nt upstream of gene PMM0120;
119798	+	TSS_000598	1000	1770	0	2	P	28nt upstream of gene PMM0121;

119905	+	TSS_000600	1000	80	0	0	I	within gene(s) PMM0121;
120990	-	TSS_010232	1000	789	0	1	IP	within gene(s) PMM0123; 240nt upstream of gene PMM0122;
121450	-	TSS_010237	1000	124	0	0	I	within gene(s) PMM0123;
121764	-	TSS_010239	1000	130	0	0	P	29nt upstream of gene PMM0123;
122897	+	TSS_000608	1000	175	0	2	O	-
123251	+	TSS_000609	1000	910	0	1	P	68nt upstream of gene PMM0126;
123779	+	TSS_000612	1000	1002	0	0	I	within gene(s) PMM0126;
124013	-	TSS_010253	1000	75	0	1	Ai	antisense to gene(s) PMM0126;
125323	+	TSS_000621	1000	2270	0	1	P	35nt upstream of gene PMM0128;
125778	+	TSS_000632	1000	80	0	23	I	within gene(s) PMM0128;
126175	-	TSS_010263	1000	108	0	0	I	within gene(s) PMM0129;
129278	-	TSS_010273	1000	107	0	0	I	within gene(s) PMM0131;
130071	-	TSS_010276	1000	346	0	3	P	34nt upstream of gene PMM0131;
130794	+	TSS_000649	1000	3266	0	1	Ai	antisense to gene(s) PMM0133;
130945	+	TSS_000652	1000	143	0	0	Ai	antisense to gene(s) PMM0133;
132109	+	TSS_001026	1000	2564	0	3	P	39nt upstream of gene PMM0134;
132594	+	TSS_000675	1000	88	0	0	I	within gene(s) PMM0134;
132850	-	TSS_010283	1000	306	0	0	Ai	antisense to gene(s) PMM0134;
133237	+	TSS_000679	1000	79	0	12	I	within gene(s) PMM0135;
134228	-	TSS_010290	1000	1765	0	6	Ai	antisense to gene(s) PMM0135;
134332	+	TSS_000689	1000	592	0	1	P	17nt upstream of gene PMM0136;
135334	+	TSS_000693	1000	264	0	2	IP	within gene(s) PMM0136; 37nt upstream of gene PMM0137;
136633	+	TSS_000698	1000	89	0	0	I	within gene(s) PMM0138;
139049	+	TSS_000701	1000	304	0	0	P	49nt upstream of gene PMM0142;
139538	-	TSS_010300	1000	62	0	0	IAd	within gene(s) PMM0143; antisense to gene(s) PMM0142 (22nt downstream);
139888	+	TSS_000704	1000	891	0	1	Ai	antisense to gene(s) PMM0143;
140081	-	TSS_010302	1000	623	0	0	I	within gene(s) PMM0143;
140446	-	TSS_010303	1000	129	0	1	P	20nt upstream of gene PMM0143;
141905	-	TSS_010312	1000	534	0	0	P	38nt upstream of gene PMM0144;
141924	+	TSS_000711	1000	249	0	5	P	34nt upstream of gene PMM0145;
142730	-	TSS_010313	1000	52	0	1	Ai	antisense to gene(s) PMM0146;
143021	-	TSS_010320	1000	128	0	2	I	within gene(s) PMM0147;
143488	-	TSS_010323	1000	407	0	3	I	within gene(s) PMM0147;
143883	-	TSS_010331	1000	419	0	12	P	14nt upstream of gene PMM0147;
143931	+	TSS_000726	1000	304	0	3	P	23nt upstream of gene PMM0148;
144023	+	TSS_000727	1000	62	0	0	I	within gene(s) PMM0148;
144701	+	TSS_000732	1000	2018	0	2	P	15nt upstream of gene PMM0149;
144965	+	TSS_000739	1000	311	0	6	I	within gene(s) PMM0149;
145139	+	TSS_000744	1000	105	0	0	I	within gene(s) PMM0149;
145211	+	TSS_000745	1000	259	0	3	I	within gene(s) PMM0149;
145511	+	TSS_000754	1000	272	0	1	I	within gene(s) PMM0149;
145715	+	TSS_000765	1000	208	0	9	I	within gene(s) PMM0149;
146423	-	TSS_010346	1000	280	0	0	Ai	antisense to gene(s) PMM0149;
146611	+	TSS_000783	1000	170	0	1	IP	within gene(s) PMM0149; 202nt upstream of gene PMM0150;
146794	+	TSS_000785	1000	11580	0	2	P	19nt upstream of gene PMM0150;
147332	+	TSS_000792	1000	433	0	0	I	within gene(s) PMM0150;
147731	-	TSS_010351	1000	104	0	1	Ai	antisense to gene(s) PMM0150;
147807	+	TSS_000799	1000	264	0	0	I	within gene(s) PMM0150;
148260	-	TSS_010354	1000	76	0	0	Ai	antisense to gene(s) PMM0150;
148515	+	TSS_000807	1000	268	0	1	P	169nt upstream of gene PMM0151;
149427	+	TSS_000811	1000	423	0	1	P	20nt upstream of gene PMM0152;
150063	+	TSS_000814	1000	62	0	0	I	within gene(s) PMM0152;
150290	+	TSS_000819	1000	302	0	0	I	within gene(s) PMM0152;
150387	-	TSS_010360	1000	218	0	4	Ai	antisense to gene(s) PMM0152;
151109	-	TSS_010365	1000	100	0	4	I	within gene(s) PMM0153;
151121	+	TSS_000823	1000	92	0	0	Ai	antisense to gene(s) PMM0153;
151351	-	TSS_010369	1000	156	0	0	I	within gene(s) PMM0153;
151412	+	TSS_000826	1000	451	0	2	PAi	165nt upstream of gene PMM0154; antisense to gene(s) PMM0153;
151546	-	TSS_010370	1000	165	0	2	P	47nt upstream of gene PMM0153;
151556	+	TSS_000828	1000	1131	0	5	P	21nt upstream of gene PMM0154;
151821	-	TSS_010375	1000	53	0	0	Ai	antisense to gene(s) PMM0154;
152951	-	TSS_010378	1000	92	0	0	I	within gene(s) PMM0156;
153992	-	TSS_010384	1000	56	0	0	IP	within gene(s) PMM0158; 190nt upstream of gene PMM0157;
154617	-	TSS_010397	1000	3746	0	36	IP	within gene(s) PMM0159; 214nt upstream of gene PMM0158;
154964	-	TSS_010404	1000	477	0	0	I	within gene(s) PMM0159;
155061	-	TSS_010406	1000	296	0	5	P	18nt upstream of gene PMM0159;
155927	-	TSS_010409	1000	69	0	3	I	within gene(s) PMM0160;
156247	-	TSS_010412	1000	60	0	4	P	16nt upstream of gene PMM0160;
157478	-	TSS_010426	1000	360	0	0	P	26nt upstream of gene PMM0161;
160552	-	TSS_010442	1000	78	0	1	I	within gene(s) PMM0164;
160684	-	TSS_010443	1000	1278	0	5	P	18nt upstream of gene PMM0164;
161048	+	TSS_000847	1000	460	0	0	P	21nt upstream of gene PMM0166;
162263	-	TSS_010446	1000	478	0	2	P	61nt upstream of gene PMM0167;
162318	+	TSS_000854	1000	1230	0	2	P	21nt upstream of gene PMM0168;
163802	-	TSS_010454	1000	368	0	0	P	32nt upstream of gene PMM0169;
163830	+	TSS_000860	1000	317	0	0	I	within gene(s) PMM0170;
165910	+	TSS_000866	1000	16802	0	3	P	15nt upstream of gene PMM0172;
166112	-	TSS_010459	1000	94	0	2	PAi	226nt upstream of gene PMM0171; antisense to gene(s) PMM0172;
166240	+	TSS_000878	1000	58	0	3	I	within gene(s) PMM0172;
166516	+	TSS_000889	1000	143	0	7	I	within gene(s) PMM0172;
172122	-	TSS_010469	1000	142	0	0	Ai	antisense to gene(s) PMM0177;

173360 -	TSS_010474	1000	543	0	2	P	17nt upstream of gene PMM0179;
173404 +	TSS_000919	1000	1031	0	0	P	16nt upstream of gene PMM0180;
175816 +	TSS_000926	1000	65	0	0	I	within gene(s) PMM0184;
176333 +	TSS_000927	1000	51	0	0	P	11nt upstream of gene PMM0185;
176661 +	TSS_000929	1000	56	0	5	I	within gene(s) PMM0185;
178914 -	TSS_010487	1000	1090	0	2	I	within gene(s) PMM0187;
180315 +	TSS_000932	1000	251	0	0	Ai	antisense to gene(s) PMM0188;
180517 +	TSS_000934	1000	83	0	0	Ai	antisense to gene(s) PMM0188;
182433 -	TSS_010492	1000	529	0	2	PAi	92nt upstream of gene PMM0189; antisense to gene(s) PMM0190;
183419 -	TSS_010494	1000	176	0	0	I	within gene(s) PMM0191;
183523 +	TSS_000939	1000	73	0	0	Ai	antisense to gene(s) PMM0191;
185776 +	TSS_000945	1000	85	0	0	I	within gene(s) PMM0192;
187884 -	TSS_010503	1000	97	0	0	IP	within gene(s) PMM0194; 90nt upstream of gene PMM0193;
188529 +	TSS_000951	1000	53	0	0	Ai	antisense to gene(s) PMM0194;
188865 +	TSS_000952	1000	1101	0	1	P	18nt upstream of gene PMM0195;
189007 +	TSS_000955	1000	379	0	1	I	within gene(s) PMM0195;
189333 +	TSS_000960	1000	71	0	4	I	within gene(s) PMM0195;
193271 -	TSS_010512	1000	64	0	0	IP	within gene(s) PMM0199; 222nt upstream of gene PMM0198;
193821 +	TSS_000977	1000	78	0	0	Ai	antisense to gene(s) PMM0199;
195266 +	TSS_000983	1000	1073	0	0	Ai	antisense to gene(s) PMM0201;
195331 -	TSS_015322	1000	246	0	3	I	within gene(s) PMM0201;
195809 -	TSS_010551	1000	277	0	3	I	within gene(s) PMM0202;
196137 -	TSS_015338	1000	15681	0	5	P	169nt upstream of gene PMM0202;
196509 +	TSS_000993	1000	122	0	1	Ai	antisense to gene(s) PMM0203;
196639 -	TSS_010572	1000	234	0	2	I	within gene(s) PMM0203;
197001 -	TSS_010577	1000	1314	0	1	IP	within gene(s) PMM0204; 133nt upstream of gene PMM0203;
197448 -	TSS_010588	1000	778	0	3	IP	within gene(s) PMM0205; 88nt upstream of gene PMM0204;
197685 +	TSS_001000	1000	213	0	0	Ai	antisense to gene(s) PMM0205;
197771 -	TSS_010596	1000	190	0	0	I	within gene(s) PMM0205;
197876 -	TSS_010601	1000	142	0	5	I	within gene(s) PMM0205;
198069 -	TSS_010604	1000	602	0	4	P	34nt upstream of gene PMM0205;
198181 -	TSS_010608	1000	281	0	0	IP	within gene(s) PMM0206; 146nt upstream of gene PMM0205;
198382 -	TSS_010609	1000	541	0	0	P	33nt upstream of gene PMM0206;
199933 -	TSS_010614	1000	158	0	3	I	within gene(s) PMM0207;
201442 +	TSS_001008	1000	5140	0	2	P	14nt upstream of gene PMM0208;
203219 -	TSS_010628	1000	845	0	0	I	within gene(s) PMM0209;
203802 -	TSS_010630	1000	85	0	0	I	within gene(s) PMM0209;
204777 -	TSS_010634	1000	86	0	1	P	41nt upstream of gene PMM0210;
204910 +	TSS_001026	1000	2073	0	2	P	82nt upstream of gene PMM0211;
205032 +	TSS_001030	1000	94	0	0	I	within gene(s) PMM0211;
205343 +	TSS_001039	1000	66	0	6	I	within gene(s) PMM0211;
205563 -	TSS_010639	1000	186	0	6	Ai	antisense to gene(s) PMM0211;
205900 -	TSS_010646	1000	1325	0	1	Ai	antisense to gene(s) PMM0211;
206424 -	TSS_010649	1000	912	0	2	IP	within gene(s) PMM0213; 172nt upstream of gene PMM0212;
207728 -	TSS_010664	1000	217	0	6	I	within gene(s) PMM0214;
208529 -	TSS_010696	1000	78	0	9	I	within gene(s) PMM0214;
208811 -	TSS_010711	1000	116	0	1	I	within gene(s) PMM0214;
208944 -	TSS_010720	1000	10707	0	4	P	28nt upstream of gene PMM0214;
209113 +	TSS_001064	1000	922	0	1	P	31nt upstream of gene PMM0215;
210185 +	TSS_001070	1000	357	0	1	P	16nt upstream of gene PMM0216;
211988 -	TSS_010727	1000	1002	0	0	Ai	antisense to gene(s) PMM0217;
212046 +	TSS_001076	1000	72	0	1	I	within gene(s) PMM0217;
212115 +	TSS_001078	1000	84	0	1	I	within gene(s) PMM0217;
212245 +	TSS_001079	1000	64	0	0	I	within gene(s) PMM0217;
212936 -	TSS_010728	1000	113	0	0	Ai	antisense to gene(s) PMM0217;
213029 +	TSS_001083	1000	648	0	6	P	33nt upstream of gene PMM0218;
214116 +	TSS_001089	1000	3069	0	5	P	17nt upstream of gene PMM0219;
214675 -	TSS_010733	1000	3234	0	5	P	19nt upstream of gene PMM0220;
216078 +	TSS_001096	1000	128	0	0	I	within gene(s) PMM0222;
216861 +	TSS_001102	1000	16843	0	17	I	within gene(s) PMM0223;
216760 +	TSS_001698	1000		0	0	P	47nt upstream of gene PMM0223;
217169 -	TSS_010742	1000	2501	0	1	Ai	antisense to gene(s) PMM0223;
217978 +	TSS_001129	1000	2371	0	3	P	24nt upstream of gene PMM0224;
218599 +	TSS_001138	1000	202	0	10	I	within gene(s) PMM0224;
218695 +	TSS_001143	1000	201	0	1	I	within gene(s) PMM0224;
219844 +	TSS_001149	1000	666	0	1	Ai	antisense to gene(s) PMM0226;
220009 +	TSS_001153	1000	611	0	6	Ai	antisense to gene(s) PMM0226;
220578 -	TSS_010821	1000	593	0	18	I	within gene(s) PMM0226;
221070 -	TSS_010850	1000	169	0	2	I	within gene(s) PMM0226;
221154 -	TSS_010854	1000	589	0	3	I	within gene(s) PMM0226;
221632 -	TSS_010866	1000	5770	0	12	P	16nt upstream of gene PMM0226;
221701 +	TSS_001171	1000	122	0	0	Ai	antisense to gene(s) PMM0227;
221978 +	TSS_001174	1000	458	0	0	Ai	antisense to gene(s) PMM0227;
222262 -	TSS_010876	1000	74	0	0	I	within gene(s) PMM0227;
223299 -	TSS_010889	1000	225	0	28	I	within gene(s) PMM0228;
223578 -	TSS_010913	1000	494	0	7	I	within gene(s) PMM0228;
223706 -	TSS_010918	1000	866	0	0	I	within gene(s) PMM0228;
223847 -	TSS_010919	1000	1197	0	1	P	140nt upstream of gene PMM0228;
225576 +	TSS_001186	1000	1117	0	0	P	14nt upstream of gene PMM0231;
225783 +	TSS_001189	1000	220	0	0	P	26nt upstream of gene PMM0232;
226315 -	TSS_010924	1000	121	0	0	I	within gene(s) PMM0233;

227265	-	TSS_010927	1000	85	0	0	IP	within gene(s) PMM0234; 96nt upstream of gene PMM0233;
227959	+	TSS_001192	1000	1231	0	0	P	28nt upstream of gene PMM0235;
228417	+	TSS_001194	1000	95	0	0	O	-
228961	+	TSS_001199	1000	81	0	2	O	-
229314	-	TSS_010935	1000	128	0	0	O	-
229472	+	TSS_001204	1000	207	0	0	P	167nt upstream of gene PMM0237;
230878	-	TSS_010938	1000	434	0	3	Ai	antisense to gene(s) PMM0238;
232150	+	TSS_001222	1000	137	0	0	I	within gene(s) PMM0238;
232913	-	TSS_010944	1000	63	0	0	Ai	antisense to gene(s) PMM0238;
233913	-	TSS_010946	1000	106	0	2	P	20nt upstream of gene PMM0239;
236862	-	TSS_010951	1000	63	0	0	I	within gene(s) PMM0243;
237372	-	TSS_010954	1000	80	0	1	IP	within gene(s) PMM0244; 48nt upstream of gene PMM0243;
237523	-	TSS_010958	1000	1664	0	3	IP	within gene(s) PMM0244; 199nt upstream of gene PMM0243;
237737	-	TSS_010959	1000	345	0	0	I	within gene(s) PMM0244;
238520	-	TSS_010963	1000	460	0	1	P	20nt upstream of gene PMM0245;
238693	+	TSS_001232	1000	1260	0	3	P	12nt upstream of gene PMM0246;
239672	-	TSS_010966	1000	74	0	3	Ai	antisense to gene(s) PMM0247;
241176	-	TSS_010968	1000	95	0	0	IP	within gene(s) PMM0250; 35nt upstream of gene PMM0249;
242059	+	TSS_001240	1000	194	0	0	PAi	162nt upstream of gene PMM0252; antisense to gene(s) PMM0251;
242165	-	TSS_010971	1000	530	0	4	P	24nt upstream of gene PMM0251;
242185	+	TSS_001242	1000	1788	0	2	P	36nt upstream of gene PMM0252;
242483	+	TSS_001247	1000	1075	0	1	P	19nt upstream of gene PMM0253;
244978	+	TSS_001253	1000	160	0	5	Ai	antisense to gene(s) PMM0255;
245524	-	TSS_010978	1000	546	0	10	IP	within gene(s) PMM0256; 219nt upstream of gene PMM0255;
245888	-	TSS_010986	1000	79	0	2	I	within gene(s) PMM0256;
246248	+	TSS_001257	1000	4605	0	1	Ai	antisense to gene(s) PMM0256;
246731	-	TSS_010992	1000	2359	0	5	IP	within gene(s) PMM0257; 20nt upstream of gene PMM0256;
248216	-	TSS_010997	1000	99	0	0	IP	within gene(s) PMM0258; 190nt upstream of gene PMM0257;
249284	-	TSS_011022	1000	2346	0	4	P	18nt upstream of gene PMM0258;
249485	+	TSS_001272	1000	396	0	2	P	22nt upstream of gene PMM0259;
251358	+	TSS_001276	1000	187	0	3	Ai	antisense to gene(s) PMM0261;
252586	+	TSS_002398	1000	675283	0	8	P	46nt upstream of gene PMM0263;
253836	+	TSS_001391	1000	2712	0	1	I	within gene(s) PMM0263;
254003	-	TSS_011074	1000	7765	0	8	Ai	antisense to gene(s) PMM0263;
254183	+	TSS_001403	1000	591	0	5	I	within gene(s) PMM0264;
254564	-	TSS_011083	1000	126	0	1	Ai	antisense to gene(s) PMM0264;
255509	+	TSS_001406	1000	161	0	1	I	within gene(s) PMM0265;
256772	+	TSS_001443	1000	134	0	0	Ai	antisense to gene(s) PMM0266;
258297	+	TSS_001451	1000	100	0	1	Ai	antisense to gene(s) PMM0268;
258478	-	TSS_011103	1000	304	0	0	I	within gene(s) PMM0268;
258539	+	TSS_001454	1000	363	0	0	Ai	antisense to gene(s) PMM0268;
258613	-	TSS_011109	1000	85	0	3	P	9nt upstream of gene PMM0268;
260372	+	TSS_001460	1000	72	0	2	Ai	antisense to gene(s) PMM0270;
261052	-	TSS_011117	1000	68	0	0	Ai	antisense to gene(s) PMM0271;
261925	+	TSS_001470	1000	7899	0	4	P	23nt upstream of gene PMM0272;
263126	-	TSS_011124	1000	772	0	4	P	19nt upstream of gene PMM0273;
263543	+	TSS_001475	1000	448	0	0	Ai	antisense to gene(s) PMM0274;
264013	+	TSS_001476	1000	93	0	0	Ai	antisense to gene(s) PMM0274;
264561	-	TSS_011131	1000	229	0	0	P	135nt upstream of gene PMM0274;
264640	+	TSS_001478	1000	253	0	2	P	18nt upstream of gene PMM0275;
265763	+	TSS_001487	1000	167	0	1	I	within gene(s) PMM0276;
267539	+	TSS_001490	1000	95	0	3	P	28nt upstream of gene PMM0278;
268849	+	TSS_001492	1000	84	0	0	IP	within gene(s) PMM0278; 175nt upstream of gene PMM0279;
269162	+	TSS_001495	1000	345	0	1	I	within gene(s) PMM0279;
271937	-	TSS_011165	1000	474	0	0	IP	within gene(s) PMM0282; 173nt upstream of gene PMM0281;
272570	-	TSS_011176	1000	105	0	1	P	3nt upstream of gene PMM0282;
273322	+	TSS_001508	1000	364	0	3	I	within gene(s) PMM0284;
274872	-	TSS_011187	1000	332	0	0	I	within gene(s) PMM0285;
274917	-	TSS_011188	1000	141	0	0	I	within gene(s) PMM0285;
277117	+	TSS_001517	1000	529	0	0	P	54nt upstream of gene PMM0288;
280333	-	TSS_011208	1000	1997	0	2	IP	within gene(s) PMM0290; 149nt upstream of gene PMM0289;
281326	-	TSS_011214	1000	124	0	3	Ai	antisense to gene(s) PMM0291;
282038	-	TSS_011218	1000	118	0	4	Ai	antisense to gene(s) PMM0291;
283519	-	TSS_011230	1000	337	0	13	I	within gene(s) PMM0293;
283811	-	TSS_011244	1000	4203	0	0	IP	within gene(s) PMM0294; 52nt upstream of gene PMM0293;
283904	-	TSS_011248	1000	2807	0	2	IP	within gene(s) PMM0294; 145nt upstream of gene PMM0293;
284183	-	TSS_011252	1000	397	0	10	P	57nt upstream of gene PMM0294;
284302	-	TSS_011256	1000	6652	0	2	PAi	176nt upstream of gene PMM0294; antisense to gene(s) PMM0295;
284638	+	TSS_001556	1000	2702	0	2	I	within gene(s) PMM0296;
284851	+	TSS_001562	1000	442	0	7	I	within gene(s) PMM0296;
285370	+	TSS_001581	1000	155	0	5	I	within gene(s) PMM0296;
285545	-	TSS_011267	1000	176	0	1	Ai	antisense to gene(s) PMM0296;
285768	+	TSS_001585	1000	48936	0	2	P	9nt upstream of gene PMM0297;
286270	+	TSS_001643	1000	1398	0	30	IP	within gene(s) PMM0299; 48nt upstream of gene PMM0300;
286877	+	TSS_001653	1000	190	0	1	Ai	antisense to gene(s) PMM0301;
287552	-	TSS_011282	1000	959	0	0	PAi	83nt upstream of gene PMM0301; antisense to gene(s) PMM0302;
287702	-	TSS_011284	1000	576	0	4	PAi	233nt upstream of gene PMM0301; antisense to gene(s) PMM0302;
290102	+	TSS_001663	1000	332	0	3	Ai	antisense to gene(s) PMM0303;
290195	+	TSS_001664	1000	87	0	0	Ai	antisense to gene(s) PMM0303;
291235	-	TSS_011289	1000	111	0	0	I	within gene(s) PMM0304;
291317	-	TSS_011290	1000	170	0	0	I	within gene(s) PMM0304;

291416	-	TSS_011292	1000	98	0	2	I	within gene(s) PMM0304;
293682	+	TSS_001668	1000	951	0	3	P	28nt upstream of gene PMM0305;
294128	-	TSS_011301	1000	292	0	1	Ai	antisense to gene(s) PMM0305;
299042	-	TSS_011346	1000	6681	0	2	P	18nt upstream of gene PMM0311;
299836	-	TSS_011375	1000	166	0	0	I	within gene(s) PMM0312;
299950	-	TSS_011379	1000	203	0	3	I	within gene(s) PMM0312;
300325	-	TSS_011387	1000	6461	0	10	P	75nt upstream of gene PMM0312;
300858	-	TSS_011395	1000	4734	0	12	P	21nt upstream of gene PMM0313;
301297	-	TSS_011414	1000	659	0	22	I	within gene(s) PMM0315;
302182	-	TSS_011494	1000	3713	0	30	I	within gene(s) PMM0315;
302461	-	TSS_011536	1000	642	0	0	I	within gene(s) PMM0315;
302478	-	TSS_011538	1000	174	0	3	I	within gene(s) PMM0315;
303244	-	TSS_011541	1000	161	0	6	O	-
303256	+	TSS_001736	1000	4531	0	18	P	24nt upstream of gene PMM0317;
305168	-	TSS_011548	1000	65	0	0	O	-
305442	-	TSS_011552	1000	230	0	0	I	within gene(s) PMM0320;
305727	-	TSS_011557	1000	650	0	3	IP	within gene(s) PMM0321; 174nt upstream of gene PMM0320;
306386	-	TSS_011563	1000	1272	0	2	P	15nt upstream of gene PMM0321;
308758	+	TSS_001762	1000	10947	0	3	Ai	antisense to gene(s) PMM0324;
309008	+	TSS_001770	1000	428	0	0	Ai	antisense to gene(s) PMM0324;
309678	-	TSS_011589	1000	168	0	0	I	within gene(s) PMM0324;
309757	-	TSS_011590	1000	319	0	0	P	22nt upstream of gene PMM0324;
309786	+	TSS_002869	1000	28462	0	3	P	20nt upstream of gene PMM0325;
309929	-	TSS_011593	1000	9342	0	2	PAi	194nt upstream of gene PMM0324; antisense to gene(s) PMM0325;
310023	+	TSS_001792	1000	1007	0	0	I	within gene(s) PMM0325;
310271	+	TSS_001806	1000	539	0	6	IP	within gene(s) PMM0325; 234nt upstream of gene PMM0326;
310480	+	TSS_001811	1000	586	0	2	P	25nt upstream of gene PMM0326;
310880	+	TSS_001841	1000	192	0	12	I	within gene(s) PMM0326;
312446	-	TSS_011641	1000	1935	0	3	P	16nt upstream of gene PMM0327;
312554	+	TSS_001856	1000	4102	0	0	O	-
312728	+	TSS_001864	1000		0	1	O	-
312873	-	TSS_011643	1000	1112	0	1	O	-
313029	+	TSS_001866	1000		0	15	O	-
313374	-	TSS_011647	1000	131	0	1	O	-
313530	+	TSS_001906	1000		0	0	O	-
314529	-	TSS_011662	1000	4028	0	3	O	-
314959	+	TSS_001927	1000	1712	0	0	O	-
315080	+	TSS_001928	1000		0	12	O	-
315603	+	TSS_002008	1000		0	5	O	-
315856	+	TSS_002023	1000		0	2	O	-
316009	+	TSS_002038	1000		0	0	O	-
316065	-	TSS_011685	1000	216	0	5	O	-
316220	-	TSS_011688	1000	511	0	4	O	-
316340	-	TSS_011698	1000	260	0	4	O	-
318015	+	TSS_002127	1000		0	0	O	-
319130	+	TSS_002134	1000	1196	0	2	Ai	antisense to gene(s) PMM0329;
319267	-	TSS_011744	1000	282	0	0	P	0nt upstream of gene PMM0329;
320607	+	TSS_002137	1000	97	0	0	Ai	antisense to gene(s) PMM0331;
322362	+	TSS_002140	1000	185	0	0	P	0nt upstream of gene PMM0333;
323615	-	TSS_011757	1000	389	0	5	P	16nt upstream of gene PMM0334;
324251	-	TSS_011760	1000	14126	0	4	O	-
324470	-	TSS_011765	1000	3377	0	4	O	-
324585	+	TSS_002150	1000	178	0	6	P	17nt upstream of gene PMM0335;
324964	+	TSS_002154	1000	1073	0	1	I	within gene(s) PMM0335;
325557	-	TSS_011801	1000	34119	0	3	P	15nt upstream of gene PMM0336;
325983	+	TSS_002164	1000	199	0	2	O	-
326712	+	TSS_002166	1000	229	0	0	O	-
327570	-	TSS_011815	1000	5891	0	2	O	-
327681	+	TSS_002169	1000	484	0	1	Ai	antisense to gene(s) PMM0339;
327887	+	TSS_002171	1000	97	0	0	Ai	antisense to gene(s) PMM0339;
328681	-	TSS_011822	1000	307	0	6	I	within gene(s) PMM0339;
329348	-	TSS_011824	1000	257	0	0	P	157nt upstream of gene PMM0339;
330221	-	TSS_011826	1000	212	0	0	P	15nt upstream of gene PMM0341;
330306	+	TSS_002175	1000	1611	0	1	P	35nt upstream of gene PMM0342;
330978	-	TSS_011829	1000	446	0	1	O	-
332301	+	TSS_002182	1000	268	0	1	P	16nt upstream of gene PMM0345;
332821	+	TSS_002189	1000	2477	0	2	I	within gene(s) PMM0346;
333569	-	TSS_011839	1000	239	0	0	P	108nt upstream of gene PMM0347;
333733	+	TSS_002192	1000	143	0	0	Ai	antisense to gene(s) PMM0348;
339021	+	TSS_002200	1000	546	0	1	P	18nt upstream of gene PMM0355;
339316	-	TSS_011847	1000	296	0	0	Ai	antisense to gene(s) PMM0355;
339953	+	TSS_003938	1000	95434	0	2	O	-
340175	-	TSS_011851	1000	192	0	0	O	-
340326	+	TSS_002204	1000	438	0	2	I	within gene(s) PMM0356;
341457	-	TSS_011856	1000	135	0	1	O	-
343466	+	TSS_002212	1000	151	0	3	O	-
345398	+	TSS_002219	1000	160	0	3	P	22nt upstream of gene PMM0363;
346662	+	TSS_002220	1000	95	0	0	O	-
346829	+	TSS_002222	1000	176	0	3	O	-
347280	+	TSS_002225	1000	5099	0	3	P	16nt upstream of gene PMM0364;
348066	-	TSS_011864	1000	1151	0	1	P	21nt upstream of gene PMM0365;

348206	+	TSS_002232	1000	563	0	5	P	17nt upstream of gene PMM0366;
348397	+	TSS_002235	1000	341	0	1	I	within gene(s) PMM0366;
348935	+	TSS_002238	1000	359	0	1	P	33nt upstream of gene PMM0367;
349333	-	TSS_011870	1000	219	0	3	Ai	antisense to gene(s) PMM0367;
349634	-	TSS_011872	1000	165	0	0	I	within gene(s) PMM0368;
349709	-	TSS_011873	1000	682	0	1	P	18nt upstream of gene PMM0368;
350069	-	TSS_011876	1000	1063	0	1	O	-
350388	+	TSS_002240	1000	243	0	1	O	-
350523	-	TSS_017092	1000	63541	0	4	O	-
351169	-	TSS_017097	1000	48923	0	6	O	-
352103	-	TSS_011884	1000	480	0	2	O	-
352188	+	TSS_002246	1000	2373	0	2	P	7nt upstream of gene PMM0369;
352957	+	TSS_002248	1000	296	0	0	P	18nt upstream of gene PMM0370;
353529	-	TSS_011888	1000	366	0	0	Ai	antisense to gene(s) PMM0370;
353732	-	TSS_011889	1000	692	0	1	Ai	antisense to gene(s) PMM0370;
354451	+	TSS_002273	1000	145	0	9	IP	within gene(s) PMM0370; 240nt upstream of gene PMM0371;
355412	-	TSS_011898	1000	331	0	0	Ai	antisense to gene(s) PMM0371;
356060	-	TSS_011899	1000	209	0	0	Ai	antisense to gene(s) PMM0372;
358183	-	TSS_011906	1000	1234	0	2	P	16nt upstream of gene PMM0377;
358512	-	TSS_011910	1000	11510	0	2	P	22nt upstream of gene PMM0378;
358753	-	TSS_011913	1000	8779	0	2	O	-
359627	-	TSS_011915	1000	1013	0	0	O	-
359991	-	TSS_011917	1000	2314	0	3	P	20nt upstream of gene PMM0379;
361663	+	TSS_002289	1000	391	0	0	O	-
362873	-	TSS_011924	1000	1352	0	3	P	140nt upstream of gene PMM0383;
366442	-	TSS_011935	1000	128	0	7	O	-
366522	-	TSS_011940	1000	1689	0	2	O	-
367091	-	TSS_011943	1000	115	0	0	I	within gene(s) PMM0386;
367935	-	TSS_011945	1000	498	0	0	O	-
368274	+	TSS_002313	1000	941	0	2	O	-
368514	-	TSS_011949	1000	328	0	4	O	-
368596	+	TSS_002315	1000	87	0	5	P	17nt upstream of gene PMM0387;
370969	+	TSS_002323	1000	3064	0	2	P	24nt upstream of gene PMM0391;
373282	+	TSS_002330	1000	81	0	1	IP	within gene(s) PMM0392; 19nt upstream of gene PMM0393;
374270	+	TSS_002333	1000	825	0	0	P	97nt upstream of gene PMM0395;
376105	+	TSS_002366	1000	125	0	1	Ai	antisense to gene(s) PMM0397;
376934	-	TSS_011976	1000	84	0	0	P	15nt upstream of gene PMM0397;
377675	-	TSS_011978	1000	184	0	1	I	within gene(s) PMM0398;
379061	-	TSS_011982	1000	140	0	2	P	24nt upstream of gene PMM0400;
380710	-	TSS_011986	1000	122	0	0	Ai	antisense to gene(s) PMM0402;
381726	+	TSS_002375	1000	3754	0	2	P	14nt upstream of gene PMM0403;
383585	+	TSS_002386	1000	10865	0	4	P	85nt upstream of gene PMM0405;
384035	-	TSS_011991	1000	399	0	1	Ai	antisense to gene(s) PMM0405;
384162	+	TSS_002423	1000	139	0	3	I	within gene(s) PMM0405;
384519	+	TSS_002440	1000	436	0	3	I	within gene(s) PMM0405;
384720	+	TSS_002453	1000	370	0	0	I	within gene(s) PMM0405;
385716	+	TSS_002462	1000	484	0	0	I	within gene(s) PMM0406;
386603	+	TSS_002473	1000	1902	0	1	Ai	antisense to gene(s) PMM0407;
387042	-	TSS_012107	1000	862	0	15	I	within gene(s) PMM0407;
387181	-	TSS_012119	1000	424	0	3	P	24nt upstream of gene PMM0407;
390631	+	TSS_002497	1000	179	0	3	P	26nt upstream of gene PMM0411;
392813	+	TSS_002502	1000	403	0	2	P	15nt upstream of gene PMM0414;
395057	+	TSS_002504	1000	143	0	0	Ai	antisense to gene(s) PMM0416;
395539	-	TSS_012147	1000	1626	0	3	P	16nt upstream of gene PMM0416;
395864	-	TSS_017469	1000	20465	0	5	O	-
396587	+	TSS_002510	1000	72	0	0	P	42nt upstream of gene PMM0419;
398147	+	TSS_002515	1000	1393	0	2	P	32nt upstream of gene PMM0420;
398719	+	TSS_002519	1000	246	0	3	I	within gene(s) PMM0420;
401612	-	TSS_012156	1000	645	0	0	P	18nt upstream of gene PMM0422;
401747	+	TSS_002529	1000	7194	0	3	O	-
401864	-	TSS_012160	1000	125	0	1	O	-
402007	-	TSS_012161	1000	187	0	0	O	-
404138	+	TSS_002533	1000	141	0	0	I	within gene(s) PMM0425;
405550	+	TSS_002534	1000	158	0	0	I	within gene(s) PMM0426;
408258	-	TSS_012169	1000	138	0	1	IP	within gene(s) PMM0428; 237nt upstream of gene PMM0427;
409230	-	TSS_012179	1000	420	0	3	P	20nt upstream of gene PMM0429;
409244	+	TSS_002543	1000	111	0	0	P	26nt upstream of gene PMM0430;
409562	-	TSS_012181	1000	285	0	0	Ai	antisense to gene(s) PMM0430;
412935	+	TSS_002549	1000	210	0	2	Ai	antisense to gene(s) PMM0435;
413484	+	TSS_002552	1000	428	0	3	Ai	antisense to gene(s) PMM0435;
414359	-	TSS_012204	1000	1149	0	2	P	44nt upstream of gene PMM0435;
414382	+	TSS_002557	1000	683	0	2	P	102nt upstream of gene PMM0436;
417856	+	TSS_002587	1000	162	0	0	I	within gene(s) PMM0438;
419521	-	TSS_012215	1000	456	0	1	Ai	antisense to gene(s) PMM0440;
422795	-	TSS_012227	1000	734	0	6	P	16nt upstream of gene PMM0443;
424880	-	TSS_012273	1000	606	0	6	I	within gene(s) PMM0445;
425395	-	TSS_012287	1000	261	0	0	I	within gene(s) PMM0446;
426162	+	TSS_002616	1000	346	0	1	P	8nt upstream of gene PMM0447;
427279	+	TSS_002626	1000	124	0	2	I	within gene(s) PMM0448;
427561	+	TSS_002632	1000	123	0	3	I	within gene(s) PMM0448;
430126	+	TSS_002652	1000	298	0	5	I	within gene(s) PMM0451;

431182	-	TSS_012320	1000	189	0	9	I	within gene(s) PMM0452;
432210	-	TSS_012388	1000	345	0	0	I	within gene(s) PMM0452;
432317	-	TSS_012391	1000	3818	0	3	P	16nt upstream of gene PMM0452;
432436	+	TSS_002676	1000	193	0	0	O	-
433380	-	TSS_012398	1000	277	0	5	P	16nt upstream of gene PMM0453;
433437	+	TSS_002678	1000	681	0	0	P	21nt upstream of gene PMM0454;
433825	-	TSS_012402	1000	270	0	0	Ai	antisense to gene(s) PMM0454;
433897	+	TSS_002683	1000	195	0	2	I	within gene(s) PMM0454;
435917	-	TSS_012408	1000	365	0	0	P	19nt upstream of gene PMM0456;
437733	+	TSS_002696	1000	161	0	0	Ai	antisense to gene(s) PMM0458;
440050	-	TSS_012418	1000	127	0	9	IP	within gene(s) PMM0461; 245nt upstream of gene PMM0460;
440245	-	TSS_012428	1000	148	0	21	I	within gene(s) PMM0461;
440885	-	TSS_012474	1000	209	0	9	IP	within gene(s) PMM0462; 115nt upstream of gene PMM0461;
441185	-	TSS_012492	1000	197	0	2	I	within gene(s) PMM0462;
441340	-	TSS_012497	1000	1346	0	3	P	29nt upstream of gene PMM0462;
442840	-	TSS_012506	1000	3010	0	5	P	17nt upstream of gene PMM0465;
444471	+	TSS_002717	1000	147	0	0	PAi	157nt upstream of gene PMM0467; antisense to gene(s) PMM0466;
444597	-	TSS_012512	1000	148	0	1	P	44nt upstream of gene PMM0466;
445213	+	TSS_002721	1000	329	0	1	Ai	antisense to gene(s) PMM0468;
445961	-	TSS_012568	1000	42134	0	4	P	42nt upstream of gene PMM0469;
445980	+	TSS_002728	1000	326	0	1	P	14nt upstream of gene PMM0470;
446903	+	TSS_002738	1000	282	0	0	IP	within gene(s) PMM0470; 167nt upstream of gene PMM0471;
447162	-	TSS_012578	1000	84	0	1	Ai	antisense to gene(s) PMM0471;
447381	+	TSS_002740	1000	1595	0	4	P	29nt upstream of gene PMM0472;
447456	+	TSS_002741	1000	109	0	0	I	within gene(s) PMM0472;
450442	+	TSS_002748	1000	118	0	1	Ai	antisense to gene(s) PMM0474;
450629	-	TSS_012595	1000	2108	0	1	P	148nt upstream of gene PMM0474;
450716	-	TSS_017932	1000	8153	0	3	IP	within gene(s) PMM0475; 235nt upstream of gene PMM0474;
450872	+	TSS_002751	1000	241	0	2	Ai	antisense to gene(s) PMM0475;
450998	+	TSS_002752	1000	431	0	2	Ai	antisense to gene(s) PMM0475;
451134	-	TSS_017945	1000	7945	0	8	P	4nt upstream of gene PMM0475;
451442	+	TSS_002758	1000	466	0	4	PAi	101nt upstream of gene PMM0477; antisense to gene(s) PMM0476;
451913	+	TSS_002787	1000	1357	0	1	I	within gene(s) PMM0477;
452132	+	TSS_002801	1000	10970	0	2	I	within gene(s) PMM0477;
453136	-	TSS_012625	1000	219	0	6	P	44nt upstream of gene PMM0478;
453247	+	TSS_002806	1000	973	0	2	P	22nt upstream of gene PMM0479;
453732	-	TSS_012630	1000	280	0	0	Ai	antisense to gene(s) PMM0479;
454387	+	TSS_002819	1000	959	0	3	P	17nt upstream of gene PMM0480;
454968	+	TSS_002822	1000	184	0	4	P	16nt upstream of gene PMM0481;
455492	+	TSS_002823	1000	188	0	0	O	-
455674	-	TSS_012639	1000	138	0	6	O	-
456144	-	TSS_012645	1000	259	0	2	Ai	antisense to gene(s) PMM0482;
456226	+	TSS_002836	1000	161	0	18	I	within gene(s) PMM0482;
456319	+	TSS_002848	1000	203	0	12	I	within gene(s) PMM0482;
456568	+	TSS_002853	1000	265	0	1	I	within gene(s) PMM0482;
456940	+	TSS_002855	1000	330	0	3	Ai	antisense to gene(s) PMM0483;
457405	+	TSS_002861	1000	730	0	2	Ai	antisense to gene(s) PMM0483;
457551	+	TSS_002863	1000	155	0	1	Ai	antisense to gene(s) PMM0483;
457755	-	TSS_012661	1000	112	0	2	I	within gene(s) PMM0483;
457975	-	TSS_012667	1000	2080	0	5	P	36nt upstream of gene PMM0483;
459029	-	TSS_012678	1000	399	0	0	P	17nt upstream of gene PMM0484;
459043	+	TSS_002866	1000	155	0	0	P	42nt upstream of gene PMM0485;
460033	+	TSS_002869	1000	103	0	3	P	60nt upstream of gene PMM0487;
465379	-	TSS_012690	1000	112	0	0	IP	within gene(s) PMM0492; 125nt upstream of gene PMM0491;
465840	+	TSS_002882	1000	663	0	3	P	16nt upstream of gene PMM0493;
466192	+	TSS_002884	1000	1020	0	2	I	within gene(s) PMM0493;
466610	+	TSS_002887	1000	157	0	0	I	within gene(s) PMM0493;
467409	+	TSS_002890	1000	172	0	1	P	15nt upstream of gene PMM0494;
467553	+	TSS_002893	1000	517	0	2	I	within gene(s) PMM0494;
467736	+	TSS_002903	1000	162	0	3	I	within gene(s) PMM0494;
468497	-	TSS_012700	1000	779	0	2	I	within gene(s) PMM0495;
469000	-	TSS_012706	1000	421	0	0	P	32nt upstream of gene PMM0495;
470267	-	TSS_012736	1000	2387	0	2	P	14nt upstream of gene PMM0496;
474859	-	TSS_012746	1000	557	0	0	IP	within gene(s) PMM0500; 37nt upstream of gene PMM0499;
475324	-	TSS_012748	1000	438	0	4	I	within gene(s) PMM0500;
475534	-	TSS_012752	1000	269	0	0	PAi	170nt upstream of gene PMM0500; antisense to gene(s) PMM0501;
475685	+	TSS_002923	1000	962	0	10	Ai	antisense to gene(s) PMM0502;
475749	+	TSS_002929	1000	2416	0	4	Ai	antisense to gene(s) PMM0502;
476045	-	TSS_012758	1000	632	0	0	P	16nt upstream of gene PMM0502;
476529	+	TSS_002933	1000	128	0	0	I	within gene(s) PMM0503;
477868	+	TSS_002935	1000	111	0	4	Ai	antisense to gene(s) PMM0505;
479542	-	TSS_012772	1000	602	0	2	P	26nt upstream of gene PMM0506;
479651	-	TSS_012773	1000	152	0	0	IP	within gene(s) PMM0507; 135nt upstream of gene PMM0506;
480066	-	TSS_012777	1000	257	0	3	IP	within gene(s) PMM0508; 31nt upstream of gene PMM0507;
481882	-	TSS_012791	1000	609	0	1	P	18nt upstream of gene PMM0508;
482442	+	TSS_002949	1000	2447	0	2	P	36nt upstream of gene PMM0510;
483044	-	TSS_012794	1000	112	0	2	Ai	antisense to gene(s) PMM0511;
483869	+	TSS_002954	1000	397	0	3	I	within gene(s) PMM0513;
486912	-	TSS_012803	1000	901	0	5	P	21nt upstream of gene PMM0515;
487573	+	TSS_002964	1000	112	0	0	I	within gene(s) PMM0516;
490430	+	TSS_002967	1000	180	0	2	I	within gene(s) PMM0518;

490706 +	TSS_005262	1000	3641	0	0 P	20nt upstream of gene PMM0519;
491974 -	TSS_012815	1000	183	0	0 I	within gene(s) PMM0520;
493398 -	TSS_012821	1000	246	0	3 I	within gene(s) PMM0521;
493513 -	TSS_012824	1000	330	0	0 IP	within gene(s) PMM0522; 82nt upstream of gene PMM0521;
493630 +	TSS_003004	1000	312	0	3 Ai	antisense to gene(s) PMM0522;
493755 +	TSS_003006	1000	140	0	0 Ai	antisense to gene(s) PMM0522;
493996 -	TSS_012831	1000	162	0	3 I	within gene(s) PMM0522;
494177 -	TSS_018185	1000	874	0	0 P	13nt upstream of gene PMM0522;
494529 +	TSS_003010	1000	264	0	0 Ai	antisense to gene(s) PMM0523;
495001 -	TSS_012835	1000	664	0	8 P	12nt upstream of gene PMM0523;
496211 +	TSS_003015	1000	1955	0	3 P	44nt upstream of gene PMM0525;
497254 +	TSS_003020	1000	235	0	6 I	within gene(s) PMM0525;
497550 +	TSS_003023	1000	3501	0	2 P	15nt upstream of gene PMM0526;
498247 +	TSS_003041	1000	101	0	6 I	within gene(s) PMM0526;
498643 +	TSS_003048	1000	305	0	0 I	within gene(s) PMM0526;
498964 -	TSS_012850	1000	189	0	2 Ai	antisense to gene(s) PMM0526;
499089 +	TSS_003081	1000	183	0	30 I	within gene(s) PMM0526;
502682 -	TSS_012876	1000	1321	0	3 P	24nt upstream of gene PMM0530;
503609 +	TSS_003095	1000	177	0	3 P	22nt upstream of gene PMM0532;
503597 +	TSS_005527	1000	2652	0	1 P	34nt upstream of gene PMM0532;
504428 +	TSS_003118	1000	339	0	5 P	29nt upstream of gene PMM0533;
504905 +	TSS_003122	1000	86	0	4 I	within gene(s) PMM0533;
505257 -	TSS_012884	1000	167	0	0 Ai	antisense to gene(s) PMM0533;
505261 +	TSS_003125	1000	504	0	10 IP	within gene(s) PMM0533; 240nt upstream of gene PMM0534;
505757 +	TSS_003131	1000	110	0	0 I	within gene(s) PMM0534;
506152 +	TSS_003135	1000	244	0	0 I	within gene(s) PMM0534;
507310 +	TSS_003138	1000	694	0	1 P	62nt upstream of gene PMM0536;
508770 -	TSS_012890	1000	186	0	0 I	within gene(s) PMM0537;
510847 +	TSS_003160	1000	824	0	2 P	19nt upstream of gene PMM0540;
511407 +	TSS_003164	1000	182	0	0 P	58nt upstream of gene PMM0542;
512853 +	TSS_003171	1000	3986	0	2 Ai	antisense to gene(s) PMM0543;
513385 -	TSS_012942	1000	8922	0	5 P	24nt upstream of gene PMM0543;
513547 +	TSS_003178	1000	138	0	0 Ad	antisense to gene(s) PMM0544 (7nt downstream);
514356 -	TSS_013001	1000	774	0	12 I	within gene(s) PMM0544;
515091 -	TSS_013055	1000	668	0	16 I	within gene(s) PMM0544;
515379 -	TSS_013097	1000	428	0	34 IP	within gene(s) PMM0545; 246nt upstream of gene PMM0544;
516192 -	TSS_013197	1000	1600	0	5 I	within gene(s) PMM0545;
516933 -	TSS_018536	1000		0	13 P	15nt upstream of gene PMM0546;
518401 -	TSS_013222	1000	240	0	6 P	18nt upstream of gene PMM0548;
518681 +	TSS_005665	1000	90415	0	2 P	44nt upstream of gene PMM0549;
519993 +	TSS_003307	1000	1684	0	3 I	within gene(s) PMM0550;
520175 -	TSS_013242	1000	197	0	0 Ai	antisense to gene(s) PMM0550;
520224 +	TSS_003313	1000	774	0	1 I	within gene(s) PMM0550;
520384 -	TSS_013247	1000	626	0	0 Ai	antisense to gene(s) PMM0550;
521962 -	TSS_013260	1000	138	0	4 Ai	antisense to gene(s) PMM0552;
522659 +	TSS_003383	1000	235	0	6 I	within gene(s) PMM0552;
523106 -	TSS_013277	1000	249	0	11 Ai	antisense to gene(s) PMM0552;
524269 -	TSS_013293	1000	136	0	0 Ai	antisense to gene(s) PMM0553;
524632 -	TSS_013297	1000	3384	0	2 Ai	antisense to gene(s) PMM0553;
524738 +	TSS_003406	1000	279	0	0 IP	within gene(s) PMM0553; 124nt upstream of gene PMM0554;
524791 +	TSS_003407	1000	1399	0	0 IP	within gene(s) PMM0553; 71nt upstream of gene PMM0554;
525973 -	TSS_013307	1000	6781	0	4 P	18nt upstream of gene PMM0557;
526448 -	TSS_013314	1000	1398	0	2 P	15nt upstream of gene PMM0558;
527220 +	TSS_003425	1000	2993	0	1 P	22nt upstream of gene PMM0560;
528660 +	TSS_003433	1000	119	0	2 I	within gene(s) PMM0561;
529035 +	TSS_003439	1000	307	0	4 I	within gene(s) PMM0561;
529358 +	TSS_003440	1000	123	0	4 I	within gene(s) PMM0561;
531559 -	TSS_013327	1000	130	0	0 P	40nt upstream of gene PMM0564;
531701 +	TSS_003456	1000	220	0	4 P	15nt upstream of gene PMM0565;
532356 -	TSS_013330	1000	273	0	2 Ai	antisense to gene(s) PMM0565;
534371 +	TSS_003463	1000	223	0	7 P	6nt upstream of gene PMM0567;
538166 +	TSS_003475	1000	615	0	2 P	179nt upstream of gene PMM0570;
539865 +	TSS_003481	1000	360	0	0 Ad	antisense to gene(s) PMM0573 (15nt downstream);
539992 +	TSS_003483	1000	773	0	0 Ai	antisense to gene(s) PMM0573;
540221 -	TSS_013363	1000		0	12 P	97nt upstream of gene PMM0573;
540317 -	TSS_013367	1000	293	0	0 P	163nt upstream of gene PMM0573;
542147 -	TSS_013396	1000	2606	0	5 I	within gene(s) PMM0577;
543284 -	TSS_013410	1000	676	0	4 P	38nt upstream of gene PMM0578;
545216 +	TSS_005808	1000	4515	0	1 Ai	antisense to gene(s) PMM0580;
546045 -	TSS_013420	1000	2117	0	1 I	within gene(s) PMM0580;
546530 +	TSS_003515	1000	1038	0	2 Ai	antisense to gene(s) PMM0581;
546844 -	TSS_013445	1000		0	5 I	17nt upstream of gene PMM0581;
548961 -	TSS_013477	1000	4613	0	4 P	43nt upstream of gene PMM0583;
550115 +	TSS_003526	1000	163	0	0 Ai	antisense to gene(s) PMM0584;
551319 -	TSS_013495	1000	461	0	2 P	13nt upstream of gene PMM0584;
558189 +	TSS_003536	1000	102	0	1 P	13nt upstream of gene PMM0590;
558490 +	TSS_003542	1000	93	0	3 I	within gene(s) PMM0590;
561219 -	TSS_013519	1000	198	0	6 I	within gene(s) PMM0593;
562701 +	TSS_003548	1000	390	0	0 Ai	antisense to gene(s) PMM0594;
563864 -	TSS_013550	1000	189	0	2 I	within gene(s) PMM0594;
564355 +	TSS_003560	1000	217	0	1 Ai	antisense to gene(s) PMM0595;

565234 -	TSS_013565	1000	193	0	4	IP	within gene(s) PMM0596; 90nt upstream of gene PMM0595;
565420 -	TSS_013568	1000	992	0	1	I	within gene(s) PMM0596;
567864 +	TSS_003576	1000	150	0	5	Ai	antisense to gene(s) PMM0597;
569600 -	TSS_013585	1000	1176	0	8	P	20nt upstream of gene PMM0599;
569733 +	TSS_003585	1000	132	0	3	P	18nt upstream of gene PMM0600;
571704 +	TSS_003598	1000	119	0	2	I	within gene(s) PMM0601;
572225 +	TSS_003601	1000	99	0	4	I	within gene(s) PMM0602;
572734 +	TSS_003607	1000	131	0	0	IP	within gene(s) PMM0602; 61nt upstream of gene PMM0603;
573526 -	TSS_013605	1000	623	0	2	Ai	antisense to gene(s) PMM0603;
573992 +	TSS_003616	1000	126	0	0	I	within gene(s) PMM0604;
574977 +	TSS_003619	1000	163	0	1	Ai	antisense to gene(s) PMM0605;
575199 -	TSS_013612	1000	2955	0	1	P	16nt upstream of gene PMM0605;
577744 +	TSS_003626	1000	210	0	0	I	within gene(s) PMM0608;
578017 -	TSS_013621	1000	263	0	0	Ai	antisense to gene(s) PMM0608;
578318 +	TSS_003633	1000	1204	0	3	IP	within gene(s) PMM0608; 247nt upstream of gene PMM0609;
578964 -	TSS_013627	1000	176	0	0	Ai	antisense to gene(s) PMM0609;
579495 +	TSS_003641	1000	378	0	0	I	within gene(s) PMM0609;
582602 -	TSS_013632	1000	426	0	3	I	within gene(s) PMM0611;
582767 -	TSS_013635	1000	156	0	5	P	17nt upstream of gene PMM0611;
584058 -	TSS_013639	1000	252	0	2	I	within gene(s) PMM0613;
585123 +	TSS_003656	1000	2318	0	6	P	120nt upstream of gene PMM0614;
585737 +	TSS_003660	1000	876	0	1	IP	within gene(s) PMM0614; 160nt upstream of gene PMM0615;
586359 +	TSS_003669	1000	150	0	3	I	within gene(s) PMM0615;
587070 -	TSS_013647	1000	349	0	0	Ai	antisense to gene(s) PMM0616;
588622 +	TSS_003678	1000	1012	0	3	P	9nt upstream of gene PMM0618;
588874 +	TSS_003680	1000	204	0	1	I	within gene(s) PMM0618;
589671 +	TSS_003684	1000	188	0	0	P	29nt upstream of gene PMM0619;
590072 +	TSS_003690	1000	178	0	9	I	within gene(s) PMM0619;
590670 -	TSS_013656	1000	1307	0	3	Ai	antisense to gene(s) PMM0619;
593663 -	TSS_013670	1000	175	0	0	I	within gene(s) PMM0622;
594680 +	TSS_003754	1000	198	0	2	I	within gene(s) PMM0623;
595886 +	TSS_003756	1000	224	0	1	P	18nt upstream of gene PMM0626;
596067 -	TSS_013674	1000	170	0	0	Ai	antisense to gene(s) PMM0626;
596385 -	TSS_013676	1000	1525	0	1	O	-
600230 +	TSS_003768	1000	472	0	0	P	26nt upstream of gene PMM0631;
601604 -	TSS_013696	1000	133	0	0	IP	within gene(s) PMM0633; 102nt upstream of gene PMM0632;
602377 -	TSS_013698	1000	256	0	0	I	within gene(s) PMM0633;
602756 -	TSS_013699	1000	114	0	2	I	within gene(s) PMM0633;
605794 +	TSS_003774	1000	302	0	4	P	19nt upstream of gene PMM0637;
608933 -	TSS_013719	1000	123	0	0	Ai	antisense to gene(s) PMM0640;
609209 -	TSS_013720	1000	573	0	0	Ai	antisense to gene(s) PMM0640;
609497 +	TSS_003785	1000	142	0	0	I	within gene(s) PMM0640;
610210 +	TSS_003794	1000	5405	0	6	P	16nt upstream of gene PMM0641;
610941 +	TSS_003799	1000	9852	0	3	P	17nt upstream of gene PMM0642;
611411 +	TSS_003813	1000	530	0	18	I	within gene(s) PMM0642;
614001 -	TSS_013743	1000	91	0	1	I	within gene(s) PMM0644;
614082 -	TSS_013745	1000	234	0	1	I	within gene(s) PMM0644;
614781 -	TSS_013754	1000	556	0	2	P	15nt upstream of gene PMM0644;
617157 -	TSS_013761	1000	7728	0	7	Ai	antisense to gene(s) PMM0646;
618720 +	TSS_003887	1000	315	0	6	Ai	antisense to gene(s) PMM0648;
619528 -	TSS_013770	1000	118	0	2	P	21nt upstream of gene PMM0648;
619599 +	TSS_003889	1000	509	0	1	P	17nt upstream of gene PMM0649;
620989 -	TSS_013774	1000	3636	0	5	P	13nt upstream of gene PMM0651;
621700 -	TSS_013778	1000	548	0	0	P	156nt upstream of gene PMM0652;
624037 -	TSS_013788	1000	228	0	4	O	-
624976 +	TSS_003899	1000	272	0	4	I	within gene(s) PMM0658;
626301 +	TSS_003905	1000	769	0	1	I	within gene(s) PMM0659;
627516 +	TSS_003908	1000	609	0	0	O	-
627971 -	TSS_013796	1000	18199	0	3	O	-
628169 +	TSS_003910	1000	4276	0	2	O	-
628505 -	TSS_013812	1000	226	0	3	I	within gene(s) PMM0660;
628656 -	TSS_013813	1000	640	0	0	P	10nt upstream of gene PMM0660;
629618 -	TSS_013837	1000	197	0	0	I	within gene(s) PMM0661;
630363 -	TSS_013863	1000	135	0	9	I	within gene(s) PMM0661;
631116 -	TSS_013882	1000	2524	0	1	I	within gene(s) PMM0661;
631165 +	TSS_003929	1000	252	0	0	P	27nt upstream of gene PMM0662;
633583 +	TSS_003940	1000	660	0	1	P	23nt upstream of gene PMM0664;
635224 -	TSS_013892	1000	157	0	3	IP	within gene(s) PMM0666; 40nt upstream of gene PMM0665;
638295 +	TSS_003954	1000	202	0	1	Ai	antisense to gene(s) PMM0670;
638760 -	TSS_013903	1000	113	0	3	IP	within gene(s) PMM0671; 217nt upstream of gene PMM0670;
643136 +	TSS_003962	1000	312	0	2	P	21nt upstream of gene PMM0675;
643669 +	TSS_003964	1000	809	0	0	P	25nt upstream of gene PMM0676;
644591 +	TSS_003974	1000	91	0	3	I	within gene(s) PMM0676;
644914 -	TSS_013920	1000	575	0	0	Ad	antisense to gene(s) PMM0676 (4nt downstream);
647282 -	TSS_013927	1000	1871	0	2	P	69nt upstream of gene PMM0678;
648438 +	TSS_003986	1000	87	0	0	Ai	antisense to gene(s) PMM0680;
649133 +	TSS_003988	1000	115	0	0	I	within gene(s) PMM0681;
649461 +	TSS_003991	1000	99	0	6	I	within gene(s) PMM0681;
651413 +	TSS_003994	1000	149	0	0	I	within gene(s) PMM0683;
652626 +	TSS_003998	1000	2227	0	1	O	-
652926 +	TSS_004002	1000	20477	0	3	I	within gene(s) PMM0684;

653400	-	TSS_013936	1000	762	0	1	Ai	antisense to gene(s) PMM0685;
653980	-	TSS_013939	1000	38699	0	2	O	-
655217	-	TSS_013942	1000	123	0	0	O	-
655603	-	TSS_013943	1000	1369	0	2	I	within gene(s) PMM0687;
655934	-	TSS_013946	1000	1332	0	1	O	-
656679	-	TSS_013953	1000	495	0	0	P	87nt upstream of gene PMM0688;
657528	-	TSS_013961	1000	3994	0	4	O	-
657710	+	TSS_004013	1000	586	0	2	O	-
657998	+	TSS_004016	1000	215	0	3	O	-
659043	-	TSS_013967	1000	152	0	3	P	15nt upstream of gene PMM0690;
659754	+	TSS_004021	1000	1088	0	1	Ad	antisense to gene(s) PMM0691 (2nt downstream);
660094	-	TSS_013973	1000	362	0	6	P	133nt upstream of gene PMM0691;
660419	-	TSS_013976	1000	331	0	6	P	22nt upstream of gene PMM0692;
661103	-	TSS_013980	1000	161	0	2	I	within gene(s) PMM0693;
661646	+	TSS_004027	1000	145	0	0	I	within gene(s) PMM0694;
661983	+	TSS_004029	1000	165	0	2	O	-
663678	-	TSS_013985	1000	438	0	6	P	16nt upstream of gene PMM0697;
664058	-	TSS_013988	1000	105	0	0	I	within gene(s) PMM0698;
665165	+	TSS_004038	1000	2270	0	5	P	16nt upstream of gene PMM0699;
667292	+	TSS_004044	1000	178	0	2	P	14nt upstream of gene PMM0703;
668059	+	TSS_004048	1000	220	0	2	I	within gene(s) PMM0704;
668368	+	TSS_004049	1000	123	0	0	I	within gene(s) PMM0704;
669388	+	TSS_004050	1000	237	0	0	P	12nt upstream of gene PMM0705;
671615	-	TSS_013997	1000	138	0	0	I	within gene(s) PMM0707;
672523	-	TSS_014000	1000	93	0	0	I	within gene(s) PMM0708;
674151	+	TSS_004070	1000	487	0	0	Ai	antisense to gene(s) PMM0709;
674830	-	TSS_014015	1000	262	0	1	I	within gene(s) PMM0709;
674981	+	TSS_004075	1000	142	0	0	Ai	antisense to gene(s) PMM0709;
675828	+	TSS_004078	1000	17568	0	6	P	26nt upstream of gene PMM0710;
676283	+	TSS_004102	1000	145	0	1	I	within gene(s) PMM0710;
676791	-	TSS_014025	1000	377	0	1	Ai	antisense to gene(s) PMM0710;
678373	+	TSS_004118	1000	135	0	0	Ai	antisense to gene(s) PMM0712;
678437	+	TSS_004120	1000	317	0	0	Ai	antisense to gene(s) PMM0712;
680440	-	TSS_014034	1000	521	0	0	P	15nt upstream of gene PMM0713;
680511	-	TSS_014035	1000	225	0	0	PAi	86nt upstream of gene PMM0713; antisense to gene(s) PMM0714;
680855	-	TSS_014036	1000	116	0	0	Ai	antisense to gene(s) PMM0714;
682685	-	TSS_014037	1000	155	0	0	P	0nt upstream of gene PMM0717;
685615	-	TSS_014039	1000	1215	0	5	P	13nt upstream of gene PMM0722;
687667	+	TSS_004132	1000	703	0	0	IP	within gene(s) PMM0724; 61nt upstream of gene PMM0725;
688326	-	TSS_014046	1000	489	0	0	Ai	antisense to gene(s) PMM0725;
688353	+	TSS_004139	1000	406	0	0	I	within gene(s) PMM0725;
688629	+	TSS_004141	1000	169	0	0	O	-
688866	+	TSS_004143	1000	3889	0	2	O	-
689593	-	TSS_019603	1000	102	0	4	P	17nt upstream of gene PMM0726;
696382	-	TSS_014057	1000	195	0	0	P	18nt upstream of gene PMM0732;
698300	-	TSS_014059	1000	579	0	3	P	15nt upstream of gene PMM0734;
699080	+	TSS_004151	1000	170	0	7	O	-
702227	-	TSS_014073	1000	274	0	2	P	16nt upstream of gene PMM0739;
702332	+	TSS_004158	1000	2809	0	5	P	11nt upstream of gene PMM0740;
702516	-	TSS_014076	1000	560	0	0	O	-
703275	+	TSS_004162	1000	855	0	1	P	17nt upstream of gene PMM0742;
703511	+	TSS_004164	1000	106	0	1	I	within gene(s) PMM0742;
704459	-	TSS_014101	1000	307	0	0	I	within gene(s) PMM0743;
705461	-	TSS_014138	1000	224	0	0	I	within gene(s) PMM0743;
705815	-	TSS_014145	1000	741	0	1	P	23nt upstream of gene PMM0743;
706437	+	TSS_004187	1000	112	0	0	Ai	antisense to gene(s) PMM0745;
708310	+	TSS_004191	1000	1900	0	2	Ai	antisense to gene(s) PMM0746;
708607	-	TSS_014151	1000	682	0	1	P	14nt upstream of gene PMM0746;
710247	+	TSS_004196	1000	216	0	1	IP	within gene(s) PMM0748; 23nt upstream of gene PMM0749;
711092	+	TSS_004204	1000	166	0	0	I	within gene(s) PMM0749;
711431	-	TSS_014159	1000	110	0	0	Ai	antisense to gene(s) PMM0749;
711539	+	TSS_004206	1000	1916	0	1	I	within gene(s) PMM0750;
711829	-	TSS_014161	1000	1000	0	6	Ai	antisense to gene(s) PMM0750;
712163	+	TSS_004209	1000	1528	0	0	IP	within gene(s) PMM0750; 42nt upstream of gene PMM0751;
713700	+	TSS_004218	1000	246	0	5	I	within gene(s) PMM0753;
713922	+	TSS_004224	1000	258	0	0	I	within gene(s) PMM0753;
714097	+	TSS_004239	1000	1494	0	4	IP	within gene(s) PMM0753; 130nt upstream of gene PMM0754;
716611	+	TSS_004255	1000	164	0	1	I	within gene(s) PMM0756;
719060	+	TSS_004263	1000	126	0	2	I	within gene(s) PMM0757;
720208	+	TSS_004270	1000	204	0	1	Ai	antisense to gene(s) PMM0758;
720641	-	TSS_014207	1000	760	0	1	I	within gene(s) PMM0758;
721002	-	TSS_014217	1000		0	0	P	15nt upstream of gene PMM0758;
721555	-	TSS_014218	1000	143	0	0	Ai	antisense to gene(s) PMM0759;
723763	-	TSS_014249	1000	1162	0	24	I	within gene(s) PMM0760;
724438	-	TSS_014296	1000	265	0	18	I	within gene(s) PMM0760;
724578	-	TSS_014299	1000	5737	0	2	P	20nt upstream of gene PMM0760;
725488	+	TSS_004283	1000	384	0	4	P	25nt upstream of gene PMM0762;
728301	-	TSS_014311	1000	314	0	0	Ai	antisense to gene(s) PMM0764;
729981	-	TSS_014324	1000	235	0	1	I	within gene(s) PMM0766;
730261	-	TSS_014326	1000	767	0	2	P	15nt upstream of gene PMM0766;
730331	+	TSS_004332	1000	673	0	0	P	103nt upstream of gene PMM0767;

730435	+	TSS_004333	1000	1078	0	1	I	within gene(s) PMM0767;
730737	+	TSS_004340	1000	289	0	7	I	within gene(s) PMM0767;
730925	+	TSS_004346	1000	146	0	6	I	within gene(s) PMM0767;
731386	-	TSS_014336	1000	84	0	1	Ai	antisense to gene(s) PMM0767;
732872	+	TSS_004368	1000	2217	0	6	P	21nt upstream of gene PMM0769;
733226	-	TSS_014345	1000	247	0	0	Ai	antisense to gene(s) PMM0769;
734116	-	TSS_014356	1000	332	0	4	Ai	antisense to gene(s) PMM0769;
734269	+	TSS_004413	1000	2128	0	4	P	18nt upstream of gene PMM0770;
736446	+	TSS_004421	1000	162	0	5	P	15nt upstream of gene PMM0772;
737785	-	TSS_014366	1000	152	0	0	I	within gene(s) PMM0774;
738561	+	TSS_004430	1000	174	0	0	Ai	antisense to gene(s) PMM0774;
738854	-	TSS_014379	1000	94	0	6	I	within gene(s) PMM0774;
738964	-	TSS_014383	1000	241	0	1	I	within gene(s) PMM0774;
739095	-	TSS_014385	1000	1287	0	1	P	13nt upstream of gene PMM0774;
739410	-	TSS_014389	1000	1611	0	10	P	14nt upstream of gene PMM0775;
740068	+	TSS_004436	1000	586	0	2	P	38nt upstream of gene PMM0777;
741992	+	TSS_004444	1000	969	0	1	P	15nt upstream of gene PMM0779;
744091	-	TSS_014493	1000	12130	0	3	P	43nt upstream of gene PMM0781;
745876	+	TSS_004461	1000	117	0	1	Ai	antisense to gene(s) PMM0784;
746288	-	TSS_014510	1000	396	0	2	I	within gene(s) PMM0784;
746584	-	TSS_020211	1000	2641	0	5	P	18nt upstream of gene PMM0784;
747387	-	TSS_014538	1000	621	0	9	I	within gene(s) PMM0785;
747664	-	TSS_020215	1000	4971	0	2	P	15nt upstream of gene PMM0785;
749173	-	TSS_014552	1000	171	0	4	I	within gene(s) PMM0787;
752029	-	TSS_014566	1000	214	0	8	P	25nt upstream of gene PMM0790;
752047	+	TSS_004480	1000	1694	0	6	Ai	antisense to gene(s) PMM0791;
752129	+	TSS_004481	1000	168	0	0	Ai	antisense to gene(s) PMM0791;
755386	-	TSS_014570	1000	1279	0	3	IP	within gene(s) PMM0795; 114nt upstream of gene PMM0794;
757822	-	TSS_014579	1000	128	0	0	P	66nt upstream of gene PMM0796;
759405	+	TSS_004488	1000	99	0	2	P	16nt upstream of gene PMM0799;
760249	-	TSS_014587	1000	97	0	11	P	17nt upstream of gene PMM0800;
760930	-	TSS_014591	1000	187	0	1	P	14nt upstream of gene PMM0801;
762992	+	TSS_004493	1000	1567	0	0	P	16nt upstream of gene PMM0804;
763063	+	TSS_004496	1000	306	0	17	I	within gene(s) PMM0804;
764067	+	TSS_004528	1000	432	0	0	Ai	antisense to gene(s) PMM0806;
764468	-	TSS_014604	1000	209	0	3	P	26nt upstream of gene PMM0806;
768634	-	TSS_014612	1000	1000	0	1	P	16nt upstream of gene PMM0810;
768967	-	TSS_014614	1000	119	0	0	O	-
769201	-	TSS_014615	1000	98	0	0	O	-
771730	-	TSS_014625	1000	397	0	1	P	22nt upstream of gene PMM0814;
771982	+	TSS_004541	1000	135	0	5	O	-
772207	-	TSS_014626	1000	165	0	0	O	-
773820	-	TSS_014639	1000	545	0	1	P	20nt upstream of gene PMM0818;
774773	-	TSS_014642	1000	2534	0	3	P	18nt upstream of gene PMM0819;
775070	-	TSS_014646	1000	255	0	1	P	49nt upstream of gene PMM0820;
775810	-	TSS_014647	1000	153	0	0	O	-
777149	-	TSS_014653	1000	566	0	0	I	within gene(s) PMM0821;
778980	-	TSS_014664	1000	140	0	2	P	19nt upstream of gene PMM0824;
784311	+	TSS_004585	1000	129	0	0	PAi	76nt upstream of gene PMM0828; antisense to gene(s) PMM0827;
784601	+	TSS_004586	1000	324	0	0	P	14nt upstream of gene PMM0829;
784835	+	TSS_004587	1000	153	0	0	I	within gene(s) PMM0829;
787909	-	TSS_014765	1000	457	0	10	I	within gene(s) PMM0831;
789844	-	TSS_014847	1000	102	0	2	I	within gene(s) PMM0831;
790206	-	TSS_014848	1000		0	1	I	17nt upstream of gene PMM0831;
790790	+	TSS_004627	1000	108	0	5	I	within gene(s) PMM0832;
796520	+	TSS_004635	1000	392	0	1	Ai	antisense to gene(s) PMM0839;
800875	-	TSS_014874	1000	107	0	0	I	within gene(s) PMM0842;
803506	-	TSS_014887	1000	103	0	12	I	within gene(s) PMM0844;
804298	-	TSS_014919	1000	2803	0	2	P	14nt upstream of gene PMM0844;
807762	-	TSS_014931	1000	222	0	2	P	29nt upstream of gene PMM0847;
808842	+	TSS_004657	1000	7629	0	6	P	117nt upstream of gene PMM0851;
810234	-	TSS_014951	1000		0	0	P	16nt upstream of gene PMM0853;
811735	-	TSS_014957	1000	480	0	0	Ai	antisense to gene(s) PMM0854;
813572	-	TSS_014996	1000	7484	0	12	P	45nt upstream of gene PMM0856;
814241	-	TSS_015003	1000	105	0	0	P	14nt upstream of gene PMM0857;
814913	+	TSS_004676	1000	1940	0	2	O	-
815258	-	TSS_015011	1000	140	0	0	O	-
816976	+	TSS_004681	1000	1777	0	1	O	-
817333	+	TSS_004684	1000	1391	0	3	O	-
817753	+	TSS_004688	1000	92	0	3	O	-
818781	+	TSS_004689	1000	137	0	0	O	-
819403	-	TSS_015025	1000	194	0	2	O	-
820064	-	TSS_015029	1000	185	0	0	O	-
821702	-	TSS_015036	1000	107	0	2	P	16nt upstream of gene PMM0864;
824596	-	TSS_015053	1000	106	0	10	P	21nt upstream of gene PMM0867;
826236	-	TSS_015057	1000	537	0	7	IP	within gene(s) PMM0870; 156nt upstream of gene PMM0869;
826307	-	TSS_020771	1000	9271	0	6	P	24nt upstream of gene PMM0870;
827556	-	TSS_015062	1000	343	0	0	Ai	antisense to gene(s) PMM0871;
828957	+	TSS_007140	1000	17983	0	2	I	within gene(s) PMM0872;
831934	+	TSS_004724	1000	128	0	0	Ai	antisense to gene(s) PMM0875;
832333	+	TSS_004726	1000	533	0	3	P	14nt upstream of gene PMM0876;

832529	-	TSS_015072	1000	211	0	2	Ai	antisense to gene(s) PMM0876;
834878	-	TSS_015079	1000	1040	0	3	I	within gene(s) PMM0877;
835327	+	TSS_004733	1000	97	0	5	Ai	antisense to gene(s) PMM0877;
837146	-	TSS_015092	1000	693	0	2	P	38nt upstream of gene PMM0878;
840244	-	TSS_015098	1000	162	0	2	Ai	antisense to gene(s) PMM0879;
841241	-	TSS_015102	1000	166	0	0	I	within gene(s) PMM0880;
842149	-	TSS_015104	1000	202	0	0	I	within gene(s) PMM0881;
844697	-	TSS_015118	1000	4359	0	5	P	15nt upstream of gene PMM0883;
845195	-	TSS_015123	1000	105	0	0	Ai	antisense to gene(s) PMM0884;
855315	+	TSS_004770	1000	156	0	2	P	49nt upstream of gene PMM0893;
857485	-	TSS_015161	1000	475	0	6	I	within gene(s) PMM0894;
857549	-	TSS_015164	1000	8309	0	3	P	18nt upstream of gene PMM0894;
858686	-	TSS_015176	1000	450	0	0	I	within gene(s) PMM0896;
858957	-	TSS_015178	1000	568	0	1	IP	within gene(s) PMM0897; 21nt upstream of gene PMM0896;
859899	+	TSS_004788	1000	615	0	0	Ai	antisense to gene(s) PMM0897;
860406	-	TSS_015192	1000	2085	0	2	I	within gene(s) PMM0897;
860982	-	TSS_015200	1000	417	0	2	P	65nt upstream of gene PMM0897;
862187	+	TSS_004797	1000	150	0	6	I	within gene(s) PMM0899;
862487	-	TSS_015205	1000	179	0	0	Ai	antisense to gene(s) PMM0899;
863809	+	TSS_004803	1000	2300	0	2	P	23nt upstream of gene PMM0901;
863967	+	TSS_004806	1000	163	0	1	I	within gene(s) PMM0901;
864270	+	TSS_004816	1000	117	0	3	I	within gene(s) PMM0901;
865761	+	TSS_004829	1000	570	0	1	P	15nt upstream of gene PMM0902;
865990	-	TSS_015214	1000	89	0	0	Ai	antisense to gene(s) PMM0902;
867288	+	TSS_004837	1000	477	0	0	I	within gene(s) PMM0906;
867687	+	TSS_004841	1000	209	0	0	Ai	antisense to gene(s) PMM0907;
867831	+	TSS_004843	1000	126	0	0	Ai	antisense to gene(s) PMM0907;
868162	-	TSS_015227	1000	116	0	0	I	within gene(s) PMM0907;
869052	+	TSS_004851	1000	124	0	7	Ai	antisense to gene(s) PMM0907;
869447	-	TSS_015250	1000	10105	0	5	P	16nt upstream of gene PMM0907;
869708	+	TSS_004857	1000	243	0	2	I	within gene(s) PMM0908;
870149	+	TSS_004859	1000	175	0	0	I	within gene(s) PMM0908;
871670	+	TSS_004866	1000	168	0	1	P	15nt upstream of gene PMM0910;
872329	-	TSS_015264	1000	155	0	1	P	33nt upstream of gene PMM0911;
873612	+	TSS_004883	1000	437	0	2	I	within gene(s) PMM0912;
876780	+	TSS_004892	1000	97	0	0	I	within gene(s) PMM0916;
881167	-	TSS_015286	1000	1216	0	2	P	15nt upstream of gene PMM0919;
881352	+	TSS_004909	1000	7081	0	4	P	15nt upstream of gene PMM0920;
883958	+	TSS_004977	1000	126	0	3	P	15nt upstream of gene PMM0922;
885534	-	TSS_015310	1000	174	0	0	PAi	91nt upstream of gene PMM0924; antisense to gene(s) PMM0925;
886557	+	TSS_004989	1000	1539	0	7	P	14nt upstream of gene PMM0926;
889213	+	TSS_004994	1000	256	0	0	Ai	antisense to gene(s) PMM0929;
891056	+	TSS_005002	1000	107	0	3	Ai	antisense to gene(s) PMM0930;
891599	-	TSS_015341	1000	4600	0	1	P	21nt upstream of gene PMM0930;
893051	-	TSS_015345	1000	91	0	3	IP	within gene(s) PMM0933; 12nt upstream of gene PMM0932;
894754	+	TSS_005009	1000	124	0	0	P	45nt upstream of gene PMM0936;
899977	+	TSS_005022	1000	832	0	3	P	13nt upstream of gene PMM0941;
900053	+	TSS_005025	1000	303	0	0	I	within gene(s) PMM0941;
901047	+	TSS_005044	1000	32185	0	9	P	14nt upstream of gene PMM0943;
905299	-	TSS_015375	1000	1429	0	2	P	14nt upstream of gene PMM0945;
906542	-	TSS_015389	1000	280	0	0	I	within gene(s) PMM0946;
907099	-	TSS_015394	1000	1792	0	3	P	6nt upstream of gene PMM0947;
911843	-	TSS_015410	1000	93	0	3	P	14nt upstream of gene PMM0953;
913344	+	TSS_005068	1000	503	0	0	I	within gene(s) PMM0954;
913440	+	TSS_005069	1000	392	0	0	IP	within gene(s) PMM0954; 246nt upstream of gene PMM0955;
913823	+	TSS_005072	1000	260	0	3	I	within gene(s) PMM0955;
914442	+	TSS_005074	1000	302	0	1	O	-
914689	-	TSS_015414	1000	451	0	2	O	-
915624	+	TSS_005077	1000	205	0	2	P	2nt upstream of gene PMM0957;
916221	-	TSS_015420	1000	149	0	2	O	-
916530	-	TSS_015423	1000	532	0	6	P	16nt upstream of gene PMM0958;
918232	+	TSS_005082	1000	206	0	0	Ai	antisense to gene(s) PMM0960;
918396	-	TSS_015429	1000	224	0	0	IP	within gene(s) PMM0961; 47nt upstream of gene PMM0960;
919141	-	TSS_015430	1000	227	0	0	I	within gene(s) PMM0961;
920008	+	TSS_005090	1000	91	0	1	PAi	153nt upstream of gene PMM0962; antisense to gene(s) PMM0961;
920682	-	TSS_015432	1000	209	0	0	Ai	antisense to gene(s) PMM0962;
922689	+	TSS_005102	1000	261	0	2	Ai	antisense to gene(s) PMM0963;
923723	-	TSS_015443	1000	293	0	0	I	within gene(s) PMM0965;
923809	-	TSS_015445	1000	155	0	4	IP	within gene(s) PMM0966; 85nt upstream of gene PMM0965;
924547	-	TSS_015448	1000	348	0	0	I	within gene(s) PMM0966;
926558	+	TSS_005119	1000	20904	0	5	P	10nt upstream of gene PMM0970;
926646	+	TSS_005123	1000	122	0	1	I	within gene(s) PMM0970;
927259	+	TSS_005142	1000	250	0	0	I	within gene(s) PMM0970;
927406	+	TSS_005145	1000	186	0	1	I	within gene(s) PMM0970;
929465	-	TSS_015467	1000	123	0	1	Ai	antisense to gene(s) PMM0972;
930151	+	TSS_005157	1000	182	0	0	IP	within gene(s) PMM0972; 101nt upstream of gene PMM0973;
930208	-	TSS_015472	1000	571	0	4	Ai	antisense to gene(s) PMM0972;
931059	+	TSS_005159	1000	154	0	0	I	within gene(s) PMM0974;
933017	+	TSS_005163	1000	385	0	0	I	within gene(s) PMM0975;
936929	+	TSS_005169	1000	141	0	1	O	-
937963	+	TSS_005172	1000	1335	0	1	P	15nt upstream of gene PMM0982;

939028	-	TSS_015485	1000	355	0	0	P	17nt upstream of gene PMM0983;
939168	+	TSS_008103	1000	3048	0	2	O	-
941587	+	TSS_005179	1000	774	0	6	P	17nt upstream of gene PMM0987;
942377	-	TSS_015491	1000	525	0	0	P	126nt upstream of gene PMM0988;
945784	+	TSS_005187	1000	1372	0	3	P	20nt upstream of gene PMM0992;
946441	+	TSS_005190	1000	285	0	0	Ai	antisense to gene(s) PMM0993;
946927	-	TSS_015497	1000	1243	0	4	P	16nt upstream of gene PMM0993;
947802	+	TSS_005194	1000	98	0	0	O	-
948048	+	TSS_008123	1000		0	9	P	17nt upstream of gene PMM0996;
948628	+	TSS_008123	1000		0	1	O	-
949329	-	TSS_021293	1000	16289	0	1	O	-
950002	+	TSS_005199	1000	1210	0	1	P	15nt upstream of gene PMM0999;
950681	+	TSS_005200	1000	168	0	0	P	15nt upstream of gene PMM1001;
952881	-	TSS_015509	1000	85	0	1	Ai	antisense to gene(s) PMM1002;
956897	-	TSS_015517	1000	2433	0	5	P	17nt upstream of gene PMM1005;
958314	-	TSS_015596	1000	237	0	0	P	15nt upstream of gene PMM1007;
958766	-	TSS_015597	1000	211	0	0	P	26nt upstream of gene PMM1008;
958974	+	TSS_005251	1000	106	0	0	I	within gene(s) PMM1009;
960692	-	TSS_015599	1000	152	0	2	P	16nt upstream of gene PMM1011;
961818	+	TSS_005256	1000	362	0	3	P	14nt upstream of gene PMM1013;
963397	-	TSS_015603	1000	93	0	0	P	14nt upstream of gene PMM1015;
968005	+	TSS_005275	1000	250	0	5	I	within gene(s) PMM1022;
971676	-	TSS_015615	1000	150	0	0	P	4nt upstream of gene PMM1026;
972175	-	TSS_015616	1000	1054	0	2	O	-
972740	+	TSS_005279	1000	643	0	0	P	166nt upstream of gene PMM1028;
973076	-	TSS_015619	1000	333	0	1	Ai	antisense to gene(s) PMM1028;
973232	+	TSS_005282	1000	1040	0	0	O	-
974221	+	TSS_005287	1000	1174	0	1	P	27nt upstream of gene PMM1030;
975550	+	TSS_005290	1000	125	0	0	P	17nt upstream of gene PMM1032;
977043	+	TSS_008322	1000	2254	0	6	P	32nt upstream of gene PMM1033;
977685	-	TSS_015634	1000	145	0	0	Ai	antisense to gene(s) PMM1033;
979706	-	TSS_015644	1000	278	0	2	O	-
979901	+	TSS_015644	1000		0	1	O	-
981600	+	TSS_005321	1000	182	0	0	I	within gene(s) PMM1038;
983461	+	TSS_005326	1000	117	0	3	I	within gene(s) PMM1039;
984870	+	TSS_005332	1000	145	0	0	I	within gene(s) PMM1041;
985398	+	TSS_005333	1000	597	0	0	IP	within gene(s) PMM1041; 91nt upstream of gene PMM1042;
985522	+	TSS_005334	1000	588	0	0	I	within gene(s) PMM1042;
995402	+	TSS_005346	1000	191	0	0	Ad	antisense to gene(s) PMM1053 (3nt downstream);
996144	-	TSS_015671	1000	276	0	0	IP	within gene(s) PMM1054; 183nt upstream of gene PMM1053;
997566	-	TSS_015689	1000	223	0	1	P	29nt upstream of gene PMM1055;
998948	-	TSS_015693	1000	138	0	0	I	within gene(s) PMM1057;
998966	+	TSS_005355	1000	2498	0	2	P	23nt upstream of gene PMM1058;
1000663	-	TSS_015701	1000	909	0	3	P	16nt upstream of gene PMM1061;
1000796	-	TSS_015705	1000	19666	0	3	P	149nt upstream of gene PMM1061;
1000995	-	TSS_015707	1000	162	0	1	I	within gene(s) PMM1062;
1001106	+	TSS_005361	1000	584	0	1	Ai	antisense to gene(s) PMM1062;
1001916	-	TSS_015710	1000	134	0	0	I	within gene(s) PMM1062;
1002034	-	TSS_015713	1000	100	0	1	P	15nt upstream of gene PMM1062;
1002186	+	TSS_005367	1000	983	0	8	P	13nt upstream of gene PMM1063;
1004709	+	TSS_005390	1000	216	0	9	IP	within gene(s) PMM1063; 118nt upstream of gene PMM1064;
1005630	+	TSS_005399	1000	94	0	0	I	within gene(s) PMM1064;
1007850	+	TSS_008477	1000	9264	0	5	P	14nt upstream of gene PMM1066;
1008963	+	TSS_005417	1000	590	0	0	I	within gene(s) PMM1066;
1009566	+	TSS_005430	1000	212	0	1	P	29nt upstream of gene PMM1067;
1011515	+	TSS_005435	1000	159	0	0	P	31nt upstream of gene PMM1069;
1012301	+	TSS_005440	1000	339	0	0	IP	within gene(s) PMM1069; 179nt upstream of gene PMM1070;
1013805	-	TSS_015739	1000	290	0	0	Ai	antisense to gene(s) PMM1071;
1018128	-	TSS_015754	1000	492	0	0	P	18nt upstream of gene PMM1074;
1018655	-	TSS_015770	1000	776	0	6	I	within gene(s) PMM1075;
1019222	-	TSS_015819	1000	1837	0	6	I	within gene(s) PMM1075;
1019352	-	TSS_015821	1000	3069	0	2	P	16nt upstream of gene PMM1075;
1019487	+	TSS_008630	1000	1756	0	1	P	32nt upstream of gene PMM1076;
1020379	-	TSS_015827	1000	3429	0	2	I	within gene(s) PMM1077;
1020982	-	TSS_015832	1000	196	0	1	I	within gene(s) PMM1077;
1021282	-	TSS_015833	1000	449	0	0	I	within gene(s) PMM1077;
1022015	+	TSS_005488	1000	259	0	4	P	18nt upstream of gene PMM1079;
1022762	-	TSS_015837	1000	291	0	0	I	within gene(s) PMM1080;
1023134	+	TSS_005496	1000	143	0	6	Ai	antisense to gene(s) PMM1080;
1023493	-	TSS_015866	1000	3209	0	2	P	19nt upstream of gene PMM1080;
1023698	+	TSS_005501	1000	101	0	3	P	26nt upstream of gene PMM1081;
1024480	+	TSS_005505	1000	274	0	2	I	within gene(s) PMM1081;
1024757	-	TSS_015871	1000	214	0	0	Ai	antisense to gene(s) PMM1081;
1028961	+	TSS_005515	1000	113	0	0	IP	within gene(s) PMM1084; 27nt upstream of gene PMM1085;
1029223	-	TSS_015883	1000	179	0	0	Ai	antisense to gene(s) PMM1085;
1029801	-	TSS_015885	1000	333	0	0	Ai	antisense to gene(s) PMM1085;
1032155	+	TSS_005522	1000	550	0	1	Ai	antisense to gene(s) PMM1088;
1034423	-	TSS_016144	1000	313	0	30	I	within gene(s) PMM1088;
1034569	-	TSS_016157	1000	20516	0	2	P	17nt upstream of gene PMM1088;
1035252	+	TSS_005561	1000	774	0	0	P	14nt upstream of gene PMM1090;
1036076	+	TSS_005570	1000	415	0	7	I	within gene(s) PMM1090;

1037579 -	TSS_016170	1000	803	0	0	Ai	antisense to gene(s) PMM1092;
1038020 +	TSS_005588	1000	104	0	0	I	within gene(s) PMM1092;
1038329 +	TSS_005591	1000	104	0	1	IP	within gene(s) PMM1092; 44nt upstream of gene PMM1093;
1038879 -	TSS_016175	1000	222	0	0	Ai	antisense to gene(s) PMM1093;
1039004 +	TSS_005596	1000	164	0	2	I	within gene(s) PMM1093;
1041219 +	TSS_005604	1000	186	0	0	Ai	antisense to gene(s) PMM1096;
1041398 -	TSS_016180	1000	116	0	0	I	within gene(s) PMM1096;
1041770 +	TSS_005605	1000	1091	0	0	Ai	antisense to gene(s) PMM1097;
1042334 -	TSS_016181	1000	450	0	0	P	14nt upstream of gene PMM1097;
1042406 +	TSS_005610	1000	4569	0	2	P	20nt upstream of gene PMM1098;
1042908 -	TSS_016185	1000	92	0	0	Ai	antisense to gene(s) PMM1098;
1046932 -	TSS_016189	1000	303	0	0	P	81nt upstream of gene PMM1101;
1057074 -	TSS_016198	1000	3783	0	1	P	35nt upstream of gene PMM1107;
1058522 +	TSS_005650	1000	579	0	3	IP	within gene(s) PMM1110; 95nt upstream of gene PMM1111;
1059299 +	TSS_005659	1000	663	0	2	P	47nt upstream of gene PMM1113;
1059858 +	TSS_005663	1000	494	0	2	I	within gene(s) PMM1113;
1060619 +	TSS_005666	1000	135	0	2	Ai	antisense to gene(s) PMM1115;
1063397 +	TSS_005674	1000	103	0	1	Ai	antisense to gene(s) PMM1116;
1063713 +	TSS_005674	1000		0	1	P	21nt upstream of gene PMM1117;
1063850 +	TSS_005678	1000	299	0	4	O	-
1064482 +	TSS_005681	1000	3270	0	5	P	19nt upstream of gene PMM1118;
1065783 -	TSS_016237	1000	6247	0	15	I	within gene(s) PMM1119;
1066209 -	TSS_022609	1000		0	0	P	9nt upstream of gene PMM1119;
1068999 -	TSS_016277	1000	6247	0	15	I	within gene(s) PMM1121;
1069447 -	TSS_016325	1000	2388	0	2	P	28nt upstream of gene PMM1121;
1070354 -	TSS_016328	1000	137	0	1	O	-
1071430 +	TSS_005748	1000	111	0	2	O	-
1071927 -	TSS_016346	1000	224	0	1	I	within gene(s) PMM1123;
1072201 -	TSS_016370	1000	300	0	18	I	within gene(s) PMM1123;
1072469 -	TSS_005754	1000		0	0	P	94nt upstream of gene PMM1123;
1074287 -	TSS_016385	1000	430	0	0	I	within gene(s) PMM1124;
1076222 +	TSS_005763	1000	105	0	0	Ai	antisense to gene(s) PMM1126;
1078286 -	TSS_016390	1000	722	0	1	I	within gene(s) PMM1127;
1078419 +	TSS_005767	1000	105	0	0	Ai	antisense to gene(s) PMM1127;
1079462 -	TSS_016395	1000	2047	0	3	P	17nt upstream of gene PMM1128;
1080845 +	TSS_005772	1000	236	0	5	P	24nt upstream of gene PMM1131;
1082063 -	TSS_016402	1000	4318	0	3	O	-
1082218 +	TSS_005782	1000	2304	0	4	P	51nt upstream of gene PMM1132;
1083365 -	TSS_016408	1000	175	0	1	Ai	antisense to gene(s) PMM1132;
1083716 +	TSS_005789	1000	90	0	0	I	within gene(s) PMM1132;
1084946 -	TSS_016416	1000	432	0	2	O	-
1085043 +	TSS_005790	1000	1535	0	1	P	19nt upstream of gene PMM1134;
1085641 +	TSS_005794	1000	3234	0	4	P	15nt upstream of gene PMM1135;
1086451 -	TSS_016420	1000	117	0	0	O	-
1087615 +	TSS_005797	1000	649	0	1	O	-
1089084 -	TSS_016426	1000	111	0	6	P	38nt upstream of gene PMM1138;
1090775 +	TSS_005805	1000	346	0	0	I	within gene(s) PMM1140;
1094754 +	TSS_005813	1000	851	0	1	P	29nt upstream of gene PMM1142;
1095730 +	TSS_005822	1000	225	0	0	I	within gene(s) PMM1142;
1100340 -	TSS_016477	1000	2022	0	6	P	17nt upstream of gene PMM1147;
1100440 +	TSS_005841	1000	762	0	1	P	16nt upstream of gene PMM1148;
1101175 +	TSS_005889	1000	3190	0	2	P	15nt upstream of gene PMM1149;
1101469 +	TSS_005889	1000		0	0	P	8nt upstream of gene PMM1149;
1103158 -	TSS_016496	1000	3316	0	2	P	16nt upstream of gene PMM1151;
1103474 -	TSS_016498	1000	737	0	2	O	-
1104586 -	TSS_016507	1000	5481	0	1	IP	within gene(s) PMM1152a; 112nt upstream of gene PMM1152;
1105595 +	TSS_005935	1000	380	0	5	IP	within gene(s) PMM1153; 99nt upstream of gene PMM1154;
1107430 -	TSS_016510	1000	1742	0	4	P	2nt upstream of gene PMM1156;
1107587 +	TSS_023319	1000		0	0	P	26nt upstream of gene PMM1157;
1108620 +	TSS_006076	1000	21745	0	33	IP	within gene(s) PMM1157; 53nt upstream of gene PMM1158;
1108805 +	TSS_006100	1000	4926	0	20	I	within gene(s) PMM1158;
1114472 +	TSS_006214	1000	112	0	6	P	107nt upstream of gene PMM1163;
1115437 +	TSS_006219	1000	360	0	0	O	-
1118179 -	TSS_016578	1000	463	0	1	P	33nt upstream of gene PMM1165;
1118495 +	TSS_006222	1000	169	0	0	Ai	antisense to gene(s) PMM1166;
1120049 +	TSS_006228	1000	2252	0	1	P	15nt upstream of gene PMM1169;
1121159 +	TSS_006233	1000	363	0	0	Ai	antisense to gene(s) PMM1171;
1121580 -	TSS_016605	1000	5960	0	3	P	27nt upstream of gene PMM1171;
1122779 +	TSS_006237	1000	401	0	3	P	17nt upstream of gene PMM1174;
1123430 -	TSS_016610	1000	683	0	6	Ai	antisense to gene(s) PMM1175;
1124119 +	TSS_006244	1000	257	0	0	I	within gene(s) PMM1176;
1125577 +	TSS_006247	1000	99	0	0	Ai	antisense to gene(s) PMM1177;
1127018 +	TSS_006253	1000	515	0	0	Ai	antisense to gene(s) PMM1178;
1127626 +	TSS_006258	1000	2671	0	2	Ai	antisense to gene(s) PMM1179;
1127928 +	TSS_006261	1000	128	0	1	Ai	antisense to gene(s) PMM1179;
1128259 +	TSS_006263	1000	709	0	1	P	28nt upstream of gene PMM1180;
1128474 -	TSS_016631	1000	1880	0	3	PAi	241nt upstream of gene PMM1179; antisense to gene(s) PMM1180;
1128570 -	TSS_016634	1000	1164	0	1	Ai	antisense to gene(s) PMM1180;
1130146 +	TSS_006288	1000	20179	0	4	P	13nt upstream of gene PMM1183;
1130398 -	TSS_016644	1000	521	0	0	Ai	antisense to gene(s) PMM1184;
1130649 +	TSS_006293	1000	1861	0	0	IP	within gene(s) PMM1184; 44nt upstream of gene PMM1185;

1131150	+	TSS_006299	1000	5915	0	4	P	29nt upstream of gene PMM1186;
1131719	+	TSS_006311	1000	747	0	0	I	within gene(s) PMM1186;
1131989	+	TSS_006317	1000	113	0	0	I	within gene(s) PMM1186;
1133995	+	TSS_006326	1000	951	0	5	I	within gene(s) PMM1188;
1136341	+	TSS_006335	1000	159	0	0	P	18nt upstream of gene PMM1190;
1136852	+	TSS_006340	1000	201	0	1	P	0nt upstream of gene PMM1191;
1137382	-	TSS_016669	1000	1203	0	1	Ai	antisense to gene(s) PMM1191;
1138412	+	TSS_006439	1000	457	0	9	I	within gene(s) PMM1191;
1138845	-	TSS_016693	1000	765	0	1	Ai	antisense to gene(s) PMM1191;
1139830	+	TSS_006480	1000	457	0	0	PAi	95nt upstream of gene PMM1193; antisense to gene(s) PMM1192;
1147383	-	TSS_016707	1000	189	0	0	I	within gene(s) PMM1199;
1150601	-	TSS_016714	1000	100	0	0	I	within gene(s) PMM1202;
1152750	-	TSS_016722	1000	194	0	0	I	within gene(s) PMM1204;
1152998	-	TSS_016723	1000	432	0	1	IP	within gene(s) PMM1205; 88nt upstream of gene PMM1204;
1154177	-	TSS_016730	1000	159	0	4	P	20nt upstream of gene PMM1205;
1157220	+	TSS_006506	1000	560	0	0	Ai	antisense to gene(s) PMM1208;
1158156	-	TSS_016736	1000	110	0	0	P	14nt upstream of gene PMM1208;
1164607	-	TSS_016750	1000	116	0	2	Ai	antisense to gene(s) PMM1215;
1168569	+	TSS_006537	1000	105	0	0	P	18nt upstream of gene PMM1219;
1175722	+	TSS_006543	1000	103	0	0	I	within gene(s) PMM1225;
1181446	-	TSS_016766	1000	318	0	2	Ai	antisense to gene(s) PMM1229;
1183984	+	TSS_006551	1000	421	0	6	I	within gene(s) PMM1232;
1186173	+	TSS_006555	1000	418	0	14	I	within gene(s) PMM1234;
1186359	-	TSS_016777	1000	369	0	2	Ai	antisense to gene(s) PMM1234;
1186823	+	TSS_006563	1000	4175	0	2	I	within gene(s) PMM1234;
1187821	+	TSS_006570	1000	554	0	2	IP	within gene(s) PMM1235; 110nt upstream of gene PMM1236;
1192406	+	TSS_006581	1000	667	0	2	IP	within gene(s) PMM1239; 15nt upstream of gene PMM1240;
1201748	-	TSS_016798	1000	204	0	0	I	within gene(s) PMM1249;
1202453	-	TSS_016801	1000	129	0	1	I	within gene(s) PMM1250;
1203802	+	TSS_006595	1000	276	0	1	Ai	antisense to gene(s) PMM1251;
1204621	-	TSS_016812	1000	308	0	0	P	82nt upstream of gene PMM1251;
1206887	+	TSS_006600	1000	216	0	0	Ai	antisense to gene(s) PMM1254;
1209541	-	TSS_016827	1000	1434	0	1	I	within gene(s) PMM1256;
1211786	+	TSS_006609	1000	190	0	0	I	within gene(s) PMM1258;
1212300	-	TSS_016834	1000	136	0	0	Ai	antisense to gene(s) PMM1258;
1212532	+	TSS_006610	1000	98	0	0	IP	within gene(s) PMM1258; 148nt upstream of gene PMM1259;
1212721	-	TSS_016836	1000	108	0	0	Ai	antisense to gene(s) PMM1258 PMM1259;
1213317	+	TSS_006616	1000	525	0	4	I	within gene(s) PMM1259;
1214339	+	TSS_006618	1000	132	0	0	I	within gene(s) PMM1260;
1214724	+	TSS_006620	1000	862	0	0	IP	within gene(s) PMM1260; 140nt upstream of gene PMM1261;
1214815	+	TSS_006622	1000	169	0	0	IP	within gene(s) PMM1260; 49nt upstream of gene PMM1261;
1215208	+	TSS_006624	1000	97	0	2	I	within gene(s) PMM1261;
1215731	-	TSS_016842	1000	107	0	2	Ai	antisense to gene(s) PMM1261;
1216927	-	TSS_016852	1000	8641	0	0	P	26nt upstream of gene PMM1262;
1218465	-	TSS_016879	1000	326	0	1	I	within gene(s) PMM1264;
1218623	+	TSS_006654	1000	1169	0	1	Ai	antisense to gene(s) PMM1264;
1219165	-	TSS_016932	1000	459	0	12	I	within gene(s) PMM1264;
1219801	-	TSS_016977	1000	393	0	1	P	24nt upstream of gene PMM1264;
1219908	-	TSS_016979	1000	327	0	0	IP	within gene(s) PMM1265; 131nt upstream of gene PMM1264;
1220160	+	TSS_006668	1000	151	0	5	Ai	antisense to gene(s) PMM1265;
1221738	-	TSS_016988	1000	92	0	3	IP	within gene(s) PMM1267; 244nt upstream of gene PMM1266;
1223096	+	TSS_006679	1000	184	0	0	Ai	antisense to gene(s) PMM1269;
1223572	-	TSS_016991	1000	141	0	3	I	within gene(s) PMM1269;
1224017	-	TSS_016998	1000	6717	0	10	P	16nt upstream of gene PMM1269;
1224107	+	TSS_006686	1000	703	0	2	Ai	antisense to gene(s) PMM1270;
1224306	-	TSS_017006	1000	373	0	3	I	within gene(s) PMM1270;
1224483	+	TSS_006689	1000	1601	0	1	Ai	antisense to gene(s) PMM1270;
1225100	-	TSS_017022	1000	1375	0	2	P	20nt upstream of gene PMM1270;
1226399	+	TSS_006697	1000	2641	0	2	PAi	139nt upstream of gene PMM1273; antisense to gene(s) PMM1272;
1226493	-	TSS_017028	1000	7750	0	2	P	23nt upstream of gene PMM1272;
1228410	-	TSS_017034	1000	124	0	0	Ai	antisense to gene(s) PMM1274;
1228824	+	TSS_006706	1000	1238	0	3	P	13nt upstream of gene PMM1276;
1229316	+	TSS_006710	1000	905	0	6	Ai	antisense to gene(s) PMM1278;
1230808	-	TSS_017050	1000	309	0	2	I	within gene(s) PMM1280;
1231722	-	TSS_017056	1000	527	0	2	IP	within gene(s) PMM1281; 194nt upstream of gene PMM1280;
1233089	+	TSS_006729	1000	1427	0	1	Ai	antisense to gene(s) PMM1283;
1233818	-	TSS_017155	1000	42426	0	2	P	18nt upstream of gene PMM1283;
1235226	-	TSS_017168	1000	15372	0	4	P	16nt upstream of gene PMM1285;
1235758	+	TSS_006768	1000	145	0	2	Ai	antisense to gene(s) PMM1286;
1236777	-	TSS_017199	1000	7744	0	7	P	16nt upstream of gene PMM1286;
1238666	-	TSS_017213	1000	306	0	1	I	within gene(s) PMM1287;
1238943	-	TSS_024014	1000	1621	0	2	P	24nt upstream of gene PMM1287;
1239039	+	TSS_006778	1000	680	0	0	P	30nt upstream of gene PMM1288;
1239358	-	TSS_017216	1000	238	0	1	Ai	antisense to gene(s) PMM1288;
1239949	-	TSS_017222	1000	405	0	0	Ai	antisense to gene(s) PMM1288;
1240190	+	TSS_006812	1000	1009	0	1	Ai	antisense to gene(s) PMM1289;
1240340	+	TSS_006819	1000	169	0	2	Ai	antisense to gene(s) PMM1289;
1240490	-	TSS_017232	1000	700	0	0	I	within gene(s) PMM1289;
1240780	+	TSS_006826	1000	153	0	1	Ai	antisense to gene(s) PMM1289;
1240970	-	TSS_017248	1000	237	0	1	I	within gene(s) PMM1289;
1241067	-	TSS_017252	1000	10882	0	2	P	18nt upstream of gene PMM1289;

1241165	+	TSS_006832	1000	142	0	3	Ad	antisense to gene(s) PMM1290 (26nt downstream);
1241345	+	TSS_006835	1000	109	0	0	Ai	antisense to gene(s) PMM1290;
1242168	+	TSS_006837	1000	268	0	1	Ai	antisense to gene(s) PMM1290;
1242445	+	TSS_006839	1000	1278	0	2	Ai	antisense to gene(s) PMM1290;
1243570	-	TSS_017266	1000	139	0	0	Ai	antisense to gene(s) PMM1291;
1244009	+	TSS_006851	1000	229	0	0	Ai	antisense to gene(s) PMM1292;
1245509	+	TSS_006857	1000	2832	0	0	P	17nt upstream of gene PMM1293;
1245700	+	TSS_006865	1000	770	0	0	I	within gene(s) PMM1293;
1245796	+	TSS_006866	1000	1885	0	6	I	within gene(s) PMM1293;
1246156	+	TSS_009993	1000		0	0	I	17nt upstream of gene PMM1294;
1246440	-	TSS_017277	1000	121	0	0	Ai	antisense to gene(s) PMM1294;
1246482	+	TSS_006885	1000	120	0	1	IP	within gene(s) PMM1294; 248nt upstream of gene PMM1295;
1247681	-	TSS_017281	1000	235	0	0	P	20nt upstream of gene PMM1296;
1250130	-	TSS_017302	1000	2890	0	6	IP	within gene(s) PMM1299; 75nt upstream of gene PMM1298;
1250468	-	TSS_017308	1000	522	0	5	I	within gene(s) PMM1299;
1251088	+	TSS_006901	1000	225	0	0	P	15nt upstream of gene PMM1300;
1252018	+	TSS_006904	1000	109	0	0	I	within gene(s) PMM1300;
1252148	+	TSS_006906	1000	187	0	2	I	within gene(s) PMM1300;
1252727	-	TSS_017313	1000	489	0	0	O	-
1252970	+	TSS_006914	1000	1431	0	6	P	32nt upstream of gene PMM1301;
1253102	+	TSS_006915	1000	190	0	0	I	within gene(s) PMM1301;
1256224	+	TSS_006926	1000	137	0	3	Ai	antisense to gene(s) PMM1303;
1258142	-	TSS_017321	1000	224	0	12	P	16nt upstream of gene PMM1304;
1258258	+	TSS_006932	1000	758	0	6	P	151nt upstream of gene PMM1305;
1260324	+	TSS_006942	1000	91	0	3	I	within gene(s) PMM1306;
1260859	+	TSS_010105	1000		0	0	IP	within gene(s) PMM1306; 33nt upstream of gene PMM1307;
1261120	+	TSS_006947	1000	162	0	0	IP	within gene(s) PMM1307; 179nt upstream of gene PMM1308;
1262176	+	TSS_006950	1000	848	0	2	P	26nt upstream of gene PMM1309;
1263425	-	TSS_017349	1000	514	0	0	O	-
1263674	+	TSS_006980	1000	716	0	1	I	within gene(s) PMM1310;
1264789	-	TSS_017352	1000	106	0	0	I	within gene(s) PMM1311;
1264868	+	TSS_006988	1000	223	0	4	Ai	antisense to gene(s) PMM1311;
1265561	+	TSS_006991	1000	15215	0	4	I	within gene(s) PMM1312;
1266213	-	TSS_017356	1000	134	0	1	Ai	antisense to gene(s) PMM1312;
1266690	+	TSS_007010	1000	11753	0	2	P	20nt upstream of gene PMM1313;
1268104	+	TSS_007076	1000	3876	0	2	P	24nt upstream of gene PMM1315;
1270161	+	TSS_007111	1000	357	0	7	P	15nt upstream of gene PMM1317;
1270931	-	TSS_017384	1000	169	0	8	I	within gene(s) PMM1318;
1272067	-	TSS_017393	1000	97779	0	8	P	65nt upstream of gene PMM1321;
1273518	-	TSS_017400	1000	1726	0	3	Ai	antisense to gene(s) PMM1322;
1273698	+	TSS_007125	1000	153	0	0	I	within gene(s) PMM1322;
1274764	+	TSS_007134	1000	943	0	1	P	86nt upstream of gene PMM1323;
1275301	-	TSS_017407	1000	113	0	0	Ai	antisense to gene(s) PMM1323;
1275591	+	TSS_007141	1000	145	0	0	I	within gene(s) PMM1323;
1276011	-	TSS_017409	1000	598	0	2	Ai	antisense to gene(s) PMM1323;
1276210	+	TSS_007144	1000	234	0	0	I	within gene(s) PMM1324;
1276620	-	TSS_017414	1000	241	0	0	Ai	antisense to gene(s) PMM1324;
1276641	+	TSS_007147	1000	402	0	0	I	within gene(s) PMM1324;
1278318	+	TSS_007153	1000	390	0	0	IP	within gene(s) PMM1326; 106nt upstream of gene PMM1327;
1281106	-	TSS_017423	1000	297	0	0	IP	within gene(s) PMM1330; 132nt upstream of gene PMM1329;
1281349	-	TSS_017426	1000	167	0	0	P	30nt upstream of gene PMM1330;
1282915	+	TSS_007173	1000	144	0	1	I	within gene(s) PMM1332;
1284193	-	TSS_017436	1000	164	0	0	I	within gene(s) PMM1333;
1284754	+	TSS_007180	1000	141	0	1	Ai	antisense to gene(s) PMM1334;
1285667	-	TSS_017439	1000	173	0	3	I	within gene(s) PMM1335;
1286762	+	TSS_007193	1000	362	0	5	Ai	antisense to gene(s) PMM1337;
1286829	-	TSS_017449	1000	174	0	2	IP	within gene(s) PMM1337; 150nt upstream of gene PMM1336;
1287550	-	TSS_017452	1000	178	0	0	IP	within gene(s) PMM1338; 17nt upstream of gene PMM1337;
1287887	-	TSS_017460	1000	269	0	9	I	within gene(s) PMM1338;
1287977	-	TSS_017462	1000	170	0	2	I	within gene(s) PMM1338;
1288162	+	TSS_007201	1000	140	0	6	Ai	antisense to gene(s) PMM1338;
1288975	-	TSS_017473	1000	243	0	0	I	within gene(s) PMM1338;
1289984	-	TSS_017481	1000	151	0	2	I	within gene(s) PMM1339;
1290416	-	TSS_017484	1000	96	0	2	I	within gene(s) PMM1339;
1290486	+	TSS_007208	1000	88	0	0	P	90nt upstream of gene PMM1340;
1291245	-	TSS_017488	1000	139	0	0	Ai	antisense to gene(s) PMM1340;
1291318	-	TSS_017489	1000	248	0	0	Ai	antisense to gene(s) PMM1340;
1291478	+	TSS_007213	1000	1722	0	2	I	within gene(s) PMM1340;
1291709	-	TSS_017490	1000	119	0	0	Ai	antisense to gene(s) PMM1340;
1292169	-	TSS_017493	1000	153	0	2	Ai	antisense to gene(s) PMM1341;
1293363	+	TSS_007228	1000	104	0	0	I	within gene(s) PMM1341;
1293438	+	TSS_007231	1000	330	0	0	I	within gene(s) PMM1341;
1293828	+	TSS_007234	1000	209	0	2	I	within gene(s) PMM1341;
1295522	-	TSS_017510	1000	120	0	0	P	13nt upstream of gene PMM1342;
1295943	-	TSS_017513	1000	121	0	0	P	48nt upstream of gene PMM1343;
1296395	+	TSS_007250	1000	9025	0	7	IP	within gene(s) PMM1344; 96nt upstream of gene PMM1345;
1297508	-	TSS_017522	1000	444	0	6	P	89nt upstream of gene PMM1346;
1299151	+	TSS_007262	1000	408	0	0	Ai	antisense to gene(s) PMM1348;
1299756	+	TSS_007264	1000	160	0	2	P	9nt upstream of gene PMM1349;
1300005	+	TSS_007266	1000	175	0	5	I	within gene(s) PMM1349;
1300564	-	TSS_017530	1000	127	0	0	Ai	antisense to gene(s) PMM1349;

1300764	+	TSS_007274	1000	12468	0	2	I	within gene(s) PMM1350;
1302130	-	TSS_017551	1000	18291	0	2	P	33nt upstream of gene PMM1352;
1302279	-	TSS_017555	1000	215	0	4	IP	within gene(s) PMM1353; 182nt upstream of gene PMM1352;
1304163	+	TSS_007316	1000	741	0	0	Ai	antisense to gene(s) PMM1354;
1304765	-	TSS_017575	1000	1212	0	4	P	19nt upstream of gene PMM1354;
1307622	+	TSS_007327	1000	556	0	0	O	-
1309146	-	TSS_017584	1000	124	0	0	I	within gene(s) PMM1360;
1309784	+	TSS_007330	1000	127	0	2	P	56nt upstream of gene PMM1361;
1313108	+	TSS_007340	1000	1165	0	3	P	16nt upstream of gene PMM1365;
1315509	-	TSS_017597	1000	1479	0	7	P	15nt upstream of gene PMM1368;
1316344	-	TSS_017609	1000	926	0	5	P	10nt upstream of gene PMM1369;
1317860	+	TSS_007358	1000	97	0	0	O	-
1318282	-	TSS_017617	1000	111	0	0	O	-
1319591	+	TSS_007363	1000	6092	0	2	O	-
1319966	+	TSS_007369	1000	589	0	2	P	13nt upstream of gene PMM1372;
1321010	+	TSS_007374	1000	5699	0	2	O	-
1322462	-	TSS_017626	1000	627	0	0	O	-
1322954	-	TSS_017628	1000	263	0	0	P	15nt upstream of gene PMM1374;
1324248	+	TSS_007385	1000	665	0	0	P	19nt upstream of gene PMM1377;
1326668	-	TSS_017637	1000	15177	0	2	O	-
1328576	+	TSS_007394	1000	106	0	0	O	-
1328956	-	TSS_017642	1000	8070	0	2	O	-
1329106	+	TSS_007397	1000	569	0	3	O	-
1332432	+	TSS_007407	1000	3521	0	2	O	-
1332606	-	TSS_017652	1000	183	0	4	O	-
1332932	-	TSS_017653	1000	97	0	0	I	within gene(s) PMM1383;
1333562	-	TSS_017656	1000	128	0	1	P	188nt upstream of gene PMM1383;
1334143	+	TSS_007412	1000	201	0	0	O	-
1334592	+	TSS_007415	1000		0	5	P	21nt upstream of gene PMM1384;
1335359	+	TSS_007426	1000	26107	0	2	O	-
1336436	+	TSS_007433	1000	469	0	0	O	-
1338319	-	TSS_017666	1000	241	0	5	Ai	antisense to gene(s) PMM1386;
1338981	+	TSS_007441	1000	197	0	2	P	17nt upstream of gene PMM1387;
1339834	-	TSS_017670	1000	333	0	0	P	32nt upstream of gene PMM1388;
1341449	+	TSS_007446	1000	135	0	0	P	15nt upstream of gene PMM1391;
1343374	+	TSS_007449	1000	149	0	0	I	within gene(s) PMM1394;
1343856	-	TSS_017680	1000	4699	0	8	O	-
1344022	-	TSS_017683	1000	152	0	2	O	-
1345690	-	TSS_024748	1000	5034	0	5	P	16nt upstream of gene PMM1399;
1346497	-	TSS_017730	1000	388	0	0	I	within gene(s) PMM1400;
1346865	-	TSS_024786	1000	78431	0	3	P	239nt upstream of gene PMM1400;
1346985	+	TSS_007461	1000	2513	0	0	P	146nt upstream of gene PMM1401;
1347115	+	TSS_010871	1000	2891	0	2	P	16nt upstream of gene PMM1401;
1347808	+	TSS_007465	1000	223	0	2	P	21nt upstream of gene PMM1402;
1350386	-	TSS_017753	1000	7268	0	5	P	18nt upstream of gene PMM1404;
1351013	-	TSS_017759	1000	614	0	4	P	26nt upstream of gene PMM1405;
1352055	-	TSS_017763	1000	485	0	0	P	24nt upstream of gene PMM1408;
1352429	-	TSS_017766	1000	793	0	2	P	18nt upstream of gene PMM1409;
1353625	-	TSS_017772	1000	267	0	6	O	-
1353716	-	TSS_024834	1000	1367	0	1	O	-
1354656	+	TSS_007483	1000	812	0	1	P	26nt upstream of gene PMM1412;
1355165	+	TSS_007492	1000	1062	0	2	P	17nt upstream of gene PMM1413;
1355765	+	TSS_007495	1000	142	0	2	IP	within gene(s) PMM1414; 230nt upstream of gene PMM1415;
1356437	-	TSS_017783	1000	331	0	0	Ai	antisense to gene(s) PMM1415;
1356695	+	TSS_007499	1000	93	0	0	I	within gene(s) PMM1415;
1357783	+	TSS_007505	1000	132	0	0	I	within gene(s) PMM1416;
1358056	+	TSS_007507	1000	136	0	2	I	within gene(s) PMM1416;
1358652	+	TSS_007515	1000	929	0	0	I	within gene(s) PMM1416;
1358898	-	TSS_017795	1000	425	0	0	Ai	antisense to gene(s) PMM1416;
1359810	-	TSS_017801	1000	384	0	6	Ai	antisense to gene(s) PMM1416;
1359899	-	TSS_017809	1000	217	0	7	O	-
1360185	-	TSS_017814	1000	23873	0	3	O	-
1362875	+	TSS_007533	1000	252	0	0	Ai	antisense to gene(s) PMM1421 PMM1422;
1363264	-	TSS_017821	1000	262	0	7	I	within gene(s) PMM1422;
1363417	-	TSS_017822	1000	270	0	0	P	27nt upstream of gene PMM1422;
1364352	-	TSS_017830	1000	3808	0	1	P	202nt upstream of gene PMM1424;
1364483	+	TSS_007537	1000	222	0	0	O	-
1365171	+	TSS_007540	1000	126	0	4	P	15nt upstream of gene PMM1425;
1366150	-	TSS_017834	1000	278	0	2	Ai	antisense to gene(s) PMM1425;
1366222	-	TSS_017836	1000	250	0	0	Ai	antisense to gene(s) PMM1425;
1367108	+	TSS_011000	1000		0	0	P	18nt upstream of gene PMM1427;
1367587	+	TSS_007545	1000	290	0	2	P	92nt upstream of gene PMM1428;
1369350	-	TSS_017841	1000	139	0	0	P	19nt upstream of gene PMM1431;
1369959	+	TSS_007554	1000	151	0	1	I	within gene(s) PMM1432;
1371646	+	TSS_007561	1000	797	0	4	P	31nt upstream of gene PMM1434;
1372149	-	TSS_017853	1000	1481	0	1	Ai	antisense to gene(s) PMM1434;
1372986	+	TSS_007570	1000	131	0	0	I	within gene(s) PMM1434;
1373308	+	TSS_007573	1000	2240	0	4	P	17nt upstream of gene PMM1435;
1373532	+	TSS_007576	1000	457	0	1	I	within gene(s) PMM1435;
1373679	+	TSS_007579	1000	1293	0	0	Ai	antisense to gene(s) PMM1436;
1374806	-	TSS_018016	1000	1734	0	18	I	within gene(s) PMM1436;

1374887	+	TSS_007611	1000	312	0	0	Ai	antisense to gene(s) PMM1436;
1375257	+	TSS_007617	1000	2944	0	1	Ai	antisense to gene(s) PMM1436;
1375708	-	TSS_025122	1000		0	4	P	71nt upstream of gene PMM1437;
1375811	+	TSS_007625	1000	1176	0	1	P	21nt upstream of gene PMM1438;
1377943	+	TSS_007718	1000	171	0	1	Ai	antisense to gene(s) PMM1440;
1378269	-	TSS_025142	1000	12461	0	3	P	18nt upstream of gene PMM1440;
1378535	-	TSS_018171	1000	155	0	0	P	17nt upstream of gene PMM1441;
1378674	+	TSS_007728	1000	390	0	2	Ai	antisense to gene(s) PMM1442;
1379477	+	TSS_007734	1000	8941	0	9	Ai	antisense to gene(s) PMM1442;
1379556	+	TSS_007737	1000	852	0	0	Ai	antisense to gene(s) PMM1442;
1379949	+	TSS_007739	1000	135	0	0	P	27nt upstream of gene PMM1443;
1380019	+	TSS_007743	1000	377	0	6	I	within gene(s) PMM1443;
1380876	+	TSS_007754	1000	2604	0	2	IP	within gene(s) PMM1443; 80nt upstream of gene PMM1444;
1382749	+	TSS_007760	1000	148	0	0	IP	within gene(s) PMM1445; 31nt upstream of gene PMM1446;
1385792	+	TSS_007769	1000	751	0	3	Ai	antisense to gene(s) PMM1449;
1386095	+	TSS_007773	1000	242	0	1	Ai	antisense to gene(s) PMM1450;
1386700	+	TSS_007780	1000	433	0	0	Ai	antisense to gene(s) PMM1450;
1387132	-	TSS_018258	1000	114	0	6	IP	within gene(s) PMM1451; 89nt upstream of gene PMM1450;
1388669	-	TSS_018386	1000	4453	0	8	IP	within gene(s) PMM1452; 88nt upstream of gene PMM1451;
1388825	-	TSS_018394	1000	319	0	1	IP	within gene(s) PMM1452; 244nt upstream of gene PMM1451;
1389346	-	TSS_018403	1000	875	0	6	IP	within gene(s) PMM1453; 191nt upstream of gene PMM1452;
1390543	-	TSS_018403	1000		0	0	P	104nt upstream of gene PMM1455;
1391446	-	TSS_018450	1000	163	0	2	IP	within gene(s) PMM1457; 115nt upstream of gene PMM1456;
1391900	-	TSS_018457	1000	351	0	1	P	44nt upstream of gene PMM1457;
1392904	-	TSS_018459	1000	157	0	4	Ai	antisense to gene(s) PMM1458;
1393119	+	TSS_007826	1000	100	0	0	I	within gene(s) PMM1458;
1393186	+	TSS_007827	1000	573	0	0	I	within gene(s) PMM1458;
1393368	+	TSS_007830	1000	1989	0	2	P	69nt upstream of gene PMM1459;
1395953	+	TSS_007839	1000	1515	0	5	P	14nt upstream of gene PMM1462;
1396223	+	TSS_007842	1000	3623	0	4	P	14nt upstream of gene PMM1463;
1397666	+	TSS_007854	1000	2038	0	4	P	16nt upstream of gene PMM1465;
1398965	+	TSS_007876	1000	117	0	1	IP	within gene(s) PMM1465; 53nt upstream of gene PMM1466;
1401712	-	TSS_018480	1000	157	0	0	I	within gene(s) PMM1467;
1403187	-	TSS_018487	1000	1106	0	1	P	46nt upstream of gene PMM1467;
1410558	+	TSS_007896	1000	135	0	0	Ai	antisense to gene(s) PMM1476;
1411576	-	TSS_018507	1000	6619	0	3	P	17nt upstream of gene PMM1479;
1412048	+	TSS_007901	1000	274	0	0	Ai	antisense to gene(s) PMM1480;
1412609	-	TSS_018513	1000	633	0	5	P	161nt upstream of gene PMM1480;
1413912	+	TSS_007904	1000	445	0	0	O	-
1414968	-	TSS_018571	1000	556	0	10	I	within gene(s) PMM1483;
1416430	+	TSS_007945	1000	530	0	6	Ai	antisense to gene(s) PMM1483;
1416999	-	TSS_018730	1000	2145	0	5	I	within gene(s) PMM1483;
1417887	-	TSS_018792	1000	240	0	7	I	within gene(s) PMM1483;
1418150	+	TSS_007967	1000	1660	0	1	Ai	antisense to gene(s) PMM1484;
1418507	+	TSS_007976	1000	2063	0	1	Ai	antisense to gene(s) PMM1484;
1419267	-	TSS_018869	1000	658	0	5	I	within gene(s) PMM1484;
1419883	+	TSS_008002	1000	407	0	1	Ai	antisense to gene(s) PMM1484;
1420518	+	TSS_008005	1000	403	0	2	Ai	antisense to gene(s) PMM1485;
1422142	+	TSS_008029	1000	311	0	0	Ai	antisense to gene(s) PMM1485;
1422752	-	TSS_019004	1000	212	0	0	I	within gene(s) PMM1485;
1422856	-	TSS_019005	1000	314	0	0	I	within gene(s) PMM1485;
1423046	-	TSS_019006	1000	169	0	1	I	within gene(s) PMM1485;
1423524	-	TSS_019012	1000	717	0	5	P	211nt upstream of gene PMM1485;
1423961	+	TSS_008036	1000	252	0	0	Ai	antisense to gene(s) PMM1486;
1424666	-	TSS_019021	1000	359	0	6	P	20nt upstream of gene PMM1487;
1426785	-	TSS_019038	1000	619	0	3	P	38nt upstream of gene PMM1489;
1427278	+	TSS_008046	1000	271	0	2	Ai	antisense to gene(s) PMM1490;
1428507	+	TSS_008050	1000	754	0	0	IP	within gene(s) PMM1491; 98nt upstream of gene PMM1492;
1428986	+	TSS_008060	1000	161	0	0	I	within gene(s) PMM1492;
1429590	+	TSS_008075	1000	443	0	8	I	within gene(s) PMM1492;
1431142	+	TSS_008104	1000	715	0	7	I	within gene(s) PMM1494;
1431241	+	TSS_008110	1000	320	0	6	I	within gene(s) PMM1494;
1433023	+	TSS_008171	1000	1021	0	3	I	within gene(s) PMM1494;
1433296	+	TSS_008177	1000	358	0	0	I	within gene(s) PMM1494;
1435445	+	TSS_008191	1000	306	0	0	I	within gene(s) PMM1496;
1436999	+	TSS_008197	1000	2155	0	1	P	24nt upstream of gene PMM1498;
1437356	-	TSS_019089	1000	677	0	1	Ai	antisense to gene(s) PMM1498;
1439023	-	TSS_019098	1000	929	0	0	I	within gene(s) PMM1499;
1439399	+	TSS_008223	1000	177	0	0	Ai	antisense to gene(s) PMM1500;
1440282	-	TSS_019119	1000	1141	0	1	P	23nt upstream of gene PMM1500;
1440561	+	TSS_008230	1000	218	0	0	I	within gene(s) PMM1501;
1441843	+	TSS_011722	1000	9587	0	3	I	within gene(s) PMM1501;
1443305	+	TSS_008235	1000	1503	0	2	PAi	201nt upstream of gene PMM1504; antisense to gene(s) PMM1503;
1443593	-	TSS_019128	1000	784	0	1	PAi	140nt upstream of gene PMM1503; antisense to gene(s) PMM1504;
1444017	-	TSS_019131	1000	140	0	2	Ai	antisense to gene(s) PMM1504;
1446380	-	TSS_019144	1000	4070	0	7	P	60nt upstream of gene PMM1507;
1447058	+	TSS_008266	1000	1623	0	1	Ai	antisense to gene(s) PMM1508;
1447086	-	TSS_019202	1000	1842	0	0	I	within gene(s) PMM1508;
1447634	+	TSS_008277	1000	535	0	1	O	-
1448025	-	TSS_019253	1000	33651	0	6	I	within gene(s) PMM1509;
1449812	-	TSS_026256	1000	3354	0	5	P	64nt upstream of gene PMM1509;

1450427	-	TSS_019360	1000	1755	0	9	IP	within gene(s) PMM1511; 104nt upstream of gene PMM1510;
1450752	-	TSS_026278	1000	2146	0	5	P	25nt upstream of gene PMM1511;
1450891	+	TSS_011781	1000	261	0	5	P	31nt upstream of gene PMM1512;
1452589	-	TSS_019376	1000	128	0	1	Ai	antisense to gene(s) PMM1512;
1454166	+	TSS_008330	1000	143	0	2	I	within gene(s) PMM1512;
1454543	+	TSS_008337	1000	318	0	3	I	within gene(s) PMM1512;
1454745	+	TSS_008339	1000	126	0	1	I	within gene(s) PMM1512;
1456713	-	TSS_019390	1000	2980	0	4	P	20nt upstream of gene PMM1514;
1459395	-	TSS_019394	1000	117	0	0	Ai	antisense to gene(s) PMM1518;
1460363	-	TSS_019411	1000	14043	0	3	P	15nt upstream of gene PMM1520;
1462291	-	TSS_019457	1000	5199	0	13	I	within gene(s) PMM1523;
1462416	-	TSS_019467	1000	5017	0	36	I	within gene(s) PMM1523;
1464275	-	TSS_019709	1000	5123	0	10	IP	within gene(s) PMM1524; 142nt upstream of gene PMM1523;
1464704	-	TSS_019746	1000	3643	0	18	I	within gene(s) PMM1524;
1465661	-	TSS_019863	1000	5893	0	33	I	within gene(s) PMM1524;
1466574	-	TSS_026889	1000	158379	0	2	P	109nt upstream of gene PMM1524;
1468533	-	TSS_019956	1000	284	0	0	Ai	antisense to gene(s) PMM1525;
1468704	+	TSS_008460	1000	90	0	7	P	21nt upstream of gene PMM1526;
1469172	+	TSS_008462	1000	140	0	0	I	within gene(s) PMM1526;
1469928	-	TSS_019961	1000	144	0	0	I	within gene(s) PMM1527;
1470923	-	TSS_019964	1000	158	0	0	P	35nt upstream of gene PMM1527;
1470954	+	TSS_008468	1000	541	0	2	P	15nt upstream of gene PMM1528;
1471756	+	TSS_008472	1000	251	0	0	Ai	antisense to gene(s) PMM1529;
1473037	-	TSS_019982	1000	471	0	7	IP	within gene(s) PMM1531; 166nt upstream of gene PMM1530;
1473409	-	TSS_019991	1000	298	0	6	IP	within gene(s) PMM1532; 114nt upstream of gene PMM1531;
1473764	-	TSS_026938	1000	4813	0	4	P	28nt upstream of gene PMM1532;
1474126	-	TSS_020011	1000	206	0	0	I	within gene(s) PMM1533;
1474906	-	TSS_020017	1000	333	0	4	IP	within gene(s) PMM1534; 247nt upstream of gene PMM1533;
1475305	-	TSS_020030	1000	953	0	0	I	within gene(s) PMM1535;
1475507	-	TSS_020036	1000	263	0	0	I	within gene(s) PMM1535;
1476176	-	TSS_020052	1000	242	0	18	IP	within gene(s) PMM1536; 175nt upstream of gene PMM1535;
1476326	-	TSS_020063	1000	264	0	19	I	within gene(s) PMM1536;
1477037	-	TSS_027000	1000	65911	0	2	P	21nt upstream of gene PMM1538;
1478505	-	TSS_020121	1000	362	0	0	I	within gene(s) PMM1540;
1478791	+	TSS_008510	1000	169	0	0	Ai	antisense to gene(s) PMM1540;
1479684	+	TSS_008519	1000	894	0	0	Ai	antisense to gene(s) PMM1542;
1480030	-	TSS_020176	1000	93	0	0	I	within gene(s) PMM1542;
1480604	-	TSS_020214	1000	13713	0	12	IP	within gene(s) PMM1544; 178nt upstream of gene PMM1543;
1482034	-	TSS_020312	1000	536	0	1	IP	within gene(s) PMM1547; 93nt upstream of gene PMM1546;
1483975	+	TSS_008614	1000	924	0	1	Ai	antisense to gene(s) PMM1552;
1484971	-	TSS_020521	1000	974	0	10	IP	within gene(s) PMM1554; 134nt upstream of gene PMM1553;
1485370	-	TSS_020549	1000	2010	0	0	I	within gene(s) PMM1555;
1486430	-	TSS_020640	1000	988	0	7	IP	within gene(s) PMM1557; 94nt upstream of gene PMM1556;
1487844	-	TSS_027287	1000	2273	0	1	P	229nt upstream of gene PMM1558;
1487895	+	TSS_008689	1000	783	0	1	P	13nt upstream of gene PMM1559;
1489480	+	TSS_008697	1000	400	0	6	P	92nt upstream of gene PMM1561;
1490373	+	TSS_012048	1000	1656	0	2	P	85nt upstream of gene PMM1562;
1490650	+	TSS_008712	1000	550	0	10	I	within gene(s) PMM1562;
1490789	-	TSS_020725	1000	278	0	2	Ai	antisense to gene(s) PMM1562;
1491832	-	TSS_020737	1000	194	0	1	P	14nt upstream of gene PMM1563;
1493560	-	TSS_020738	1000	109	0	0	Ai	antisense to gene(s) PMM1565;
1494281	+	TSS_008753	1000	278	0	0	Ai	antisense to gene(s) PMM1566;
1494828	+	TSS_008756	1000	104	0	8	Ai	antisense to gene(s) PMM1566;
1495329	+	TSS_008757	1000	1079	0	2	Ai	antisense to gene(s) PMM1566;
1495758	-	TSS_020748	1000	1530	0	3	PAi	19nt upstream of gene PMM1566; antisense to gene(s) PMM1567;
1496620	+	TSS_008761	1000	585	0	0	IAd	within gene(s) PMM1567; antisense to gene(s) PMM1568 (11nt downstream);
1497016	-	TSS_020764	1000	11428	0	10	P	21nt upstream of gene PMM1568;
1497103	-	TSS_020768	1000	124	0	2	P	108nt upstream of gene PMM1568;
1499500	-	TSS_020778	1000	111	0	2	I	within gene(s) PMM1570;
1499641	-	TSS_020780	1000	304	0	0	P	35nt upstream of gene PMM1570;
1499652	+	TSS_008780	1000	188	0	11	P	65nt upstream of gene PMM1571;
1501198	+	TSS_008788	1000	135	0	0	Ai	antisense to gene(s) PMM1573;
1502320	-	TSS_020787	1000	125	0	0	I	within gene(s) PMM1574;
1503407	+	TSS_008797	1000	162	0	1	Ai	antisense to gene(s) PMM1575;
1504039	-	TSS_020812	1000	168	0	0	I	within gene(s) PMM1575;
1504074	+	TSS_008803	1000	10	0.0000064	0	Ai	antisense to gene(s) PMM1575;
1504964	-	TSS_020824	1000	178	0	0	I	within gene(s) PMM1575;
1505706	-	TSS_020830	1000	648	0	0	I	within gene(s) PMM1575;
1508411	-	TSS_020839	1000	249	0	1	P	11nt upstream of gene PMM1577;
1508562	+	TSS_008812	1000	255	0	1	Ai	antisense to gene(s) PMM1578;
1508903	-	TSS_027468	1000	72639	0	6	P	21nt upstream of gene PMM1578;
1510763	-	TSS_020866	1000	151	0	0	I	within gene(s) PMM1580;
1511027	+	TSS_008820	1000	993	0	1	Ai	antisense to gene(s) PMM1580;
1512795	-	TSS_020872	1000	948	0	1	P	92nt upstream of gene PMM1581;
1513258	-	TSS_020877	1000	116	0	0	Ai	antisense to gene(s) PMM1582;
1513707	-	TSS_020878	1000	363	0	0	Ai	antisense to gene(s) PMM1582;
1514262	-	TSS_020880	1000	155	0	0	I	within gene(s) PMM1583;
1515544	-	TSS_020887	1000	644	0	1	I	within gene(s) PMM1585;
1517074	-	TSS_020891	1000	115	0	0	P	173nt upstream of gene PMM1586;
1518093	-	TSS_020891	1000		0	3	I	51nt upstream of gene PMM1588;
1518815	-	TSS_020898	1000	154	0	3	I	within gene(s) PMM1589;

1519244	-	TSS_020908	1000	971	0	1	P	24nt upstream of gene PMM1589;
1523570	-	TSS_020922	1000	1103	0	1	P	23nt upstream of gene PMM1594;
1524215	+	TSS_008856	1000	233	0	2	P	40nt upstream of gene PMM1596;
1525299	+	TSS_008863	1000	107	0	9	I	within gene(s) PMM1596;
1527577	+	TSS_008869	1000	100	0	1	Ai	antisense to gene(s) PMM1598;
1528833	-	TSS_020944	1000	255	0	4	P	16nt upstream of gene PMM1599;
1529501	-	TSS_020964	1000	296	0	0	I	within gene(s) PMM1600;
1530109	+	TSS_008881	1000	311	0	0	Ai	antisense to gene(s) PMM1600;
1530254	-	TSS_020983	1000	4762	0	2	P	27nt upstream of gene PMM1600;
1530378	+	TSS_008884	1000	7600	0	3	P	3nt upstream of gene PMM1601;
1531400	-	TSS_020992	1000	336	0	0	Ai	antisense to gene(s) PMM1601;
1533752	-	TSS_021001	1000	10495	0	8	P	14nt upstream of gene PMM1602;
1533863	-	TSS_021007	1000	118	0	0	P	125nt upstream of gene PMM1602;
1533931	+	TSS_021008	1000		0	0	O	-
1535492	-	TSS_021021	1000	257	0	0	PAi	224nt upstream of gene PMM1604; antisense to gene(s) PMM1605;
1536359	-	TSS_021027	1000	112	0	1	I	within gene(s) PMM1606;
1537533	-	TSS_021034	1000	468	0	0	I	within gene(s) PMM1606;
1537744	+	TSS_008932	1000	218	0	0	Ai	antisense to gene(s) PMM1606;
1538095	-	TSS_027681	1000	29801	0	9	P	22nt upstream of gene PMM1607;
1538185	+	TSS_012854	1000	8049	0	2	P	23nt upstream of gene PMM1608;
1539467	+	TSS_008960	1000	2715	0	1	I	within gene(s) PMM1609;
1539568	+	TSS_008963	1000	758	0	2	IP	within gene(s) PMM1609; 176nt upstream of gene PMM1610;
1539778	-	TSS_021060	1000	353	0	2	Ai	antisense to gene(s) PMM1610;
1543254	-	TSS_021122	1000	2481	0	2	P	126nt upstream of gene PMM1611;
1543584	+	TSS_009046	1000	437	0	0	Ai	antisense to gene(s) PMM1613;
1543786	+	TSS_009047	1000	343	0	0	Ai	antisense to gene(s) PMM1613;
1546552	-	TSS_021140	1000	613	0	0	I	within gene(s) PMM1615;
1547526	+	TSS_009063	1000	438	0	0	Ai	antisense to gene(s) PMM1618;
1548296	+	TSS_009070	1000	105	0	7	Ai	antisense to gene(s) PMM1618;
1548968	-	TSS_021150	1000	148	0	0	P	25nt upstream of gene PMM1618;
1549235	-	TSS_021156	1000	1221	0	0	I	within gene(s) PMM1619;
1549500	-	TSS_021169	1000	241	0	12	I	within gene(s) PMM1619;
1549798	-	TSS_027963	1000	7806	0	8	P	58nt upstream of gene PMM1619;
1550719	-	TSS_021174	1000	110	0	0	I	within gene(s) PMM1621;
1551244	+	TSS_009076	1000	106	0	2	Ai	antisense to gene(s) PMM1622;
1552273	-	TSS_021192	1000	2162	0	4	P	14nt upstream of gene PMM1622;
1552380	+	TSS_009083	1000	268	0	1	P	12nt upstream of gene PMM1623;
1552921	+	TSS_009089	1000	180	0	0	Ai	antisense to gene(s) PMM1624;
1554498	-	TSS_021285	1000	881	0	15	I	within gene(s) PMM1625;
1554856	-	TSS_021329	1000	7453	0	4	P	16nt upstream of gene PMM1625;
1556300	-	TSS_021336	1000	156	0	0	P	17nt upstream of gene PMM1627;
1557432	+	TSS_009123	1000	356	0	1	Ai	antisense to gene(s) PMM1629;
1557486	-	TSS_021342	1000	146	0	13	I	within gene(s) PMM1629;
1558291	-	TSS_021383	1000	1119	0	2	P	18nt upstream of gene PMM1629;
1558425	+	TSS_009132	1000	228	0	0	Ai	antisense to gene(s) PMM1630;
1559126	-	TSS_021401	1000	183	0	0	I	within gene(s) PMM1630;
1559756	-	TSS_021427	1000	9490	0	9	I	within gene(s) PMM1630;
1561061	-	TSS_021445	1000	8699	0	3	IP	within gene(s) PMM1634; 205nt upstream of gene PMM1633;
1561701	+	TSS_009149	1000	177	0	2	Ai	antisense to gene(s) PMM1634;
1562872	-	TSS_028599	1000	700	0	1	P	52nt upstream of gene PMM1634;
1563062	-	TSS_021488	1000	268	0	4	PAi	242nt upstream of gene PMM1634; antisense to gene(s) PMM1635;
1563853	+	TSS_028602	1000		0	0	P	71nt upstream of gene PMM1636;
1563968	-	TSS_021493	1000	559	0	0	Ai	antisense to gene(s) PMM1636;
1564062	+	TSS_009161	1000	721	0	0	I	within gene(s) PMM1636;
1564123	-	TSS_021494	1000	732	0	0	Ai	antisense to gene(s) PMM1636;
1565055	-	TSS_021497	1000	266	0	0	Ai	antisense to gene(s) PMM1637;
1566190	-	TSS_021501	1000	164	0	0	Ai	antisense to gene(s) PMM1638;
1566861	+	TSS_009176	1000	151	0	1	Ai	antisense to gene(s) PMM1639;
1567306	-	TSS_021525	1000	152	0	3	I	within gene(s) PMM1639;
1568018	-	TSS_021542	1000	192	0	9	I	within gene(s) PMM1639;
1568329	+	TSS_009191	1000	221	0	0	Ai	antisense to gene(s) PMM1639;
1569177	-	TSS_021576	1000	487	0	0	P	31nt upstream of gene PMM1639;
1569383	+	TSS_009200	1000	386	0	1	I	within gene(s) PMM1640;
1570531	-	TSS_021581	1000	3692	0	2	I	within gene(s) PMM1642;
1570651	-	TSS_021585	1000		0	0	P	14nt upstream of gene PMM1642;
1571379	-	TSS_021592	1000	164	0	0	P	16nt upstream of gene PMM1643;
1571635	-	TSS_028741	1000	4670	0	0	P	26nt upstream of gene PMM1644;
1571739	+	TSS_009205	1000	287	0	0	P	14nt upstream of gene PMM1645;
1573488	-	TSS_021600	1000	121	0	0	Ai	antisense to gene(s) PMM1645;
1576258	+	TSS_009214	1000	396	0	0	I	within gene(s) PMM1648;
1576861	+	TSS_009219	1000	797	0	2	I	within gene(s) PMM1648;
1579478	-	TSS_028794	1000		0	0	I	within gene(s) PMM1649;
1580087	-	TSS_021636	1000	214	0	3	P	22nt upstream of gene PMM1649;
1580887	-	TSS_021645	1000	1840	0	3	I	within gene(s) PMM1650;
1582484	+	TSS_009246	1000	275	0	0	Ai	antisense to gene(s) PMM1652;
1582766	-	TSS_021667	1000	343	0	2	I	within gene(s) PMM1652;
1583497	-	TSS_021692	1000	283	0	0	I	within gene(s) PMM1652;
1584409	-	TSS_021701	1000	3969	0	2	I	within gene(s) PMM1653;
1584725	+	TSS_009262	1000	219	0	1	Ai	antisense to gene(s) PMM1653;
1585023	-	TSS_021709	1000	1473	0	5	IP	within gene(s) PMM1654; 71nt upstream of gene PMM1653;
1586076	+	TSS_009270	1000	144	0	8	P	32nt upstream of gene PMM1655;

1587579 +	TSS_009276	1000	768	0	2	P	16nt upstream of gene PMM1656;
1588301 +	TSS_009285	1000	2386	0	5	P	45nt upstream of gene PMM1657;
1589048 +	TSS_009308	1000	582	0	12	I	within gene(s) PMM1657;
1590631 -	TSS_021725	1000	231	0	0	Ai	antisense to gene(s) PMM1658;
1594453 +	TSS_009330	1000	2540	0	5	P	19nt upstream of gene PMM1661;
1594701 +	TSS_009332	1000	500	0	0	I	within gene(s) PMM1662;
1596577 +	TSS_013259	1000	1191	0	4	P	32nt upstream of gene PMM1665;
1597137 +	TSS_009358	1000	227	0	11	I	within gene(s) PMM1665;
1599261 -	TSS_021749	1000	827	0	0	P	34nt upstream of gene PMM1667;
1602622 -	TSS_021788	1000	106	0	4	I	within gene(s) PMM1669;
1604240 -	TSS_021796	1000	272	0	1	IP	within gene(s) PMM1671; 236nt upstream of gene PMM1670;
1604740 -	TSS_021800	1000	273	0	2	P	24nt upstream of gene PMM1671;
1604757 +	TSS_009398	1000	108	0	4	I	within gene(s) PMM1672;
1605445 +	TSS_009413	1000	212	0	4	I	within gene(s) PMM1672;
1605722 -	TSS_021804	1000	124	0	0	Ai	antisense to gene(s) PMM1672;
1606251 +	TSS_009426	1000	252	0	2	P	15nt upstream of gene PMM1674;
1607611 +	TSS_009433	1000	1085	0	2	IP	within gene(s) PMM1674; 49nt upstream of gene PMM1675;
1607768 -	TSS_021809	1000	767	0	1	Ai	antisense to gene(s) PMM1675;
1609627 +	TSS_009442	1000	523	0	2	P	4nt upstream of gene PMM1676;
1610827 -	TSS_021827	1000	206	0	0	O	-
1611481 +	TSS_009445	1000	1933	0	1	P	15nt upstream of gene PMM1678;
1616290 +	TSS_009466	1000	437	0	0	Ai	antisense to gene(s) PMM1682;
1616930 -	TSS_021857	1000	727	0	6	P	26nt upstream of gene PMM1682;
1617422 -	TSS_021863	1000	704	0	9	P	93nt upstream of gene PMM1683;
1618289 +	TSS_009474	1000	128	0	6	IP	within gene(s) PMM1684; 78nt upstream of gene PMM1685;
1618516 +	TSS_009477	1000	160	0	0	I	within gene(s) PMM1685;
1618595 +	TSS_009478	1000	144	0	0	I	within gene(s) PMM1685;
1619698 -	TSS_021867	1000	219	0	0	Ai	antisense to gene(s) PMM1686;
1619840 -	TSS_021868	1000	208	0	0	Ai	antisense to gene(s) PMM1686;
1620131 +	TSS_009482	1000	327	0	4	P	24nt upstream of gene PMM1687;
1621301 +	TSS_009487	1000	510	0	4	P	25nt upstream of gene PMM1688;
1622476 -	TSS_021873	1000	1298	0	7	Ai	antisense to gene(s) PMM1688;
1623168 +	TSS_009497	1000	1215	0	7	P	29nt upstream of gene PMM1689;
1623293 +	TSS_009503	1000	435	0	2	I	within gene(s) PMM1689;
1624375 +	TSS_009510	1000	228	0	0	I	within gene(s) PMM1689;
1624911 -	TSS_021879	1000	104	0	0	Ai	antisense to gene(s) PMM1690;
1625074 -	TSS_021880	1000	249	0	0	Ai	antisense to gene(s) PMM1690;
1628185 -	TSS_021885	1000	229	0	1	Ai	antisense to gene(s) PMM1693;
1628842 -	TSS_021886	1000	130	0	1	I	within gene(s) PMM1694;
1628980 +	TSS_009519	1000	431	0	2	PAi	173nt upstream of gene PMM1695; antisense to gene(s) PMM1694;
1629235 +	TSS_009520	1000	288	0	1	I	within gene(s) PMM1695;
1632609 +	TSS_009526	1000	153	0	1	P	98nt upstream of gene PMM1697;
1632679 +	TSS_013570	1000	286	0	0	P	28nt upstream of gene PMM1697;
1633040 +	TSS_009535	1000	229	0	0	I	within gene(s) PMM1697;
1633252 -	TSS_021896	1000	188	0	0	Ai	antisense to gene(s) PMM1697;
1634577 +	TSS_009550	1000	249	0	0	Ai	antisense to gene(s) PMM1699;
1634772 +	TSS_009551	1000	129	0	0	Ai	antisense to gene(s) PMM1699;
1635344 +	TSS_009552	1000	227	0	2	PAi	199nt upstream of gene PMM1700; antisense to gene(s) PMM1699;
1635526 +	TSS_009555	1000	389	0	5	P	17nt upstream of gene PMM1700;
1639698 -	TSS_021915	1000	174	0	0	I	within gene(s) PMM1702;
1640380 -	TSS_021917	1000	438	0	0	P	32nt upstream of gene PMM1702;
1640445 +	TSS_009576	1000	445	0	0	I	within gene(s) PMM1703;
1642285 -	TSS_022020	1000	438	0	24	I	within gene(s) PMM1704;
1642444 -	TSS_022043	1000	799	0	19	I	within gene(s) PMM1704;
1642606 -	TSS_022059	1000	704	0	3	I	within gene(s) PMM1704;
1643399 -	TSS_022155	1000	12967	0	7	P	19nt upstream of gene PMM1704;
1644415 +	TSS_009609	1000	2823	0	2	P	50nt upstream of gene PMM1706;
1644804 +	TSS_009619	1000	196	0	2	I	within gene(s) PMM1706;
1645357 -	TSS_022164	1000	189	0	0	I	within gene(s) PMM1707;
1646216 -	TSS_022178	1000	4639	0	0	P	69nt upstream of gene PMM1707;
1646419 +	TSS_013924	1000	566	0	2	P	20nt upstream of gene PMM1708;
1647767 +	TSS_009648	1000	108	0	2	IP	within gene(s) PMM1709; 21nt upstream of gene PMM1710;
1648727 +	TSS_009651	1000	92	0	0	Ai	antisense to gene(s) PMM1711;
1650015 -	TSS_022201	1000	134	0	0	I	within gene(s) PMM1712;
1654524 +	TSS_009664	1000	195	0	0	I	within gene(s) PMM1714;
1656926 +	TSS_009673	1000	199	0	5	I	within gene(s) PMM1716;

**Class represents the classification of the start site. I represents internal start sites, Ai represents antisense start sites, P represents primary start sites, IP represents internal or primary start sites, PAi represents primary or Antisense start sites, Ad represnts Antisense or downstream IAd represents internal Antisense or downstream and O represents orphan.
