# Supplementary material for: Nitrogen cost minimization is promoted by structural changes in the transcriptome of N-deprived Prochlorococcus cells
Source: ISME J. 2017 Jun 6;11(10):2267–78. doi: 10.1038/ismej.2017.88 (PMC5607370; doi:10.1038/ismej.2017.88)
Supplement: Supplementary Table 9 [file ismej201788x16.pdf]

Table S9. N-Starved Transcriptional Start Sites at 12 Hours Post Starvation Identified by TSSAR.

Position	Strand	ID	Score	Difference	p-Value	Positional (	Class	Comment
158	+	TSS_000004	1000	850	0	5	P	16nt upstream of gene PMM0001;
1089	+	TSS_000024	1000	198	0	12	IP	within gene(s) PMM0001; 244nt upstream of gene PMM0002;
1991	+	TSS_000029	1000	563	0	4	IP	within gene(s) PMM0002; 53nt upstream of gene PMM0003;
2084	+	TSS_000032	1000	5414	0	2	I	within gene(s) PMM0003;
2919	-	TSS_013990	1000	217	0	1	Ai	antisense to gene(s) PMM0003;
3197	+	TSS_000062	1000	374	0	5	I	within gene(s) PMM0003;
5103	+	TSS_000078	1000	242	0	0	I	within gene(s) PMM0004;
8361	-	TSS_014015	1000	1179	0	7	P	33nt upstream of gene PMM0005;
9568	+	TSS_000084	1000	70	0	0	Ai	antisense to gene(s) PMM0007;
10339	+	TSS_000087	1000	2189	0	4	P	28nt upstream of gene PMM0008;
11772	+	TSS_000101	1000	101	0	1	P	21nt upstream of gene PMM0010;
12129	+	TSS_000105	1000	71	0	3	I	within gene(s) PMM0010;
14116	-	TSS_014030	1000	1896	0	6	Ai	antisense to gene(s) PMM0011;
14568	+	TSS_000124	1000	1239	0	1	P	17nt upstream of gene PMM0012;
14964	-	TSS_014035	1000	119	0	0	Ai	antisense to gene(s) PMM0012;
15146	+	TSS_000146	1000	193	0	9	I	within gene(s) PMM0012;
16016	+	TSS_000160	1000	20080	0	16	P	2nt upstream of gene PMM0013;
16255	-	TSS_014041	1000	139	0	1	Ai	antisense to gene(s) PMM0013;
17066	+	TSS_000176	1000	115	0	1	Ai	antisense to gene(s) PMM0014;
17671	+	TSS_000179	1000	267	0	2	P	41nt upstream of gene PMM0015;
18267	+	TSS_000190	1000	160	0	0	P	16nt upstream of gene PMM0016;
23179	-	TSS_014061	1000	389	0	1	I	within gene(s) PMM0022;
24122	+	TSS_000226	1000	111	0	1	P	28nt upstream of gene PMM0023;
24575	-	TSS_014066	1000	113	0	0	Ai	antisense to gene(s) PMM0023;
25810	-	TSS_014072	1000	97	0	0	I	within gene(s) PMM0024;
25886	-	TSS_014074	1000	1148	0	1	I	within gene(s) PMM0024;
26468	-	TSS_014079	1000	720	0	1	I	within gene(s) PMM0025;
27201	+	TSS_000241	1000	150	0	0	P	86nt upstream of gene PMM0026;
27273	+	TSS_000242	1000	313	0	0	P	14nt upstream of gene PMM0026;
27367	+	TSS_000244	1000	196	0	0	I	within gene(s) PMM0026;
27478	-	TSS_014094	1000	385	0	1	Ai	antisense to gene(s) PMM0026;
27552	+	TSS_000247	1000	81	0	1	I	within gene(s) PMM0026;
27755	+	TSS_000249	1000	425	0	0	IP	within gene(s) PMM0026; 92nt upstream of gene PMM0027;
28211	+	TSS_000255	1000	132	0	1	I	within gene(s) PMM0027;
30271	+	TSS_000258	1000	1488	0	3	I	within gene(s) PMM0030;
30946	-	TSS_014113	1000	299	0	4	P	16nt upstream of gene PMM0031;
31393	-	TSS_014141	1000	363	0	10	I	within gene(s) PMM0032;
31536	-	TSS_014155	1000	10678	0	3	P	16nt upstream of gene PMM0032;
31640	+	TSS_000276	1000	485	0	2	Ai	antisense to gene(s) PMM0033;
32109	-	TSS_014163	1000	175	0	4	P	18nt upstream of gene PMM0033;
32317	+	TSS_000281	1000	4985	0	4	P	18nt upstream of gene PMM0034;
32608	-	TSS_014167	1000	153	0	0	I	within gene(s) PMM0035;
32866	-	TSS_014189	1000	222	0	20	I	within gene(s) PMM0035;
33078	+	TSS_000292	1000	748	0	1	Ai	antisense to gene(s) PMM0035;
33713	-	TSS_014274	1000	11654	0	2	P	16nt upstream of gene PMM0035;
34181	+	TSS_000301	1000	105	0	0	I	within gene(s) PMM0036;
34926	+	TSS_000302	1000	544	0	0	I	within gene(s) PMM0037;
34946	+	TSS_000303	1000	30	0	0	I	within gene(s) PMM0037;
35112	+	TSS_000304	1000	141	0	0	I	within gene(s) PMM0037;
36839	+	TSS_000319	1000	160	0	0	I	within gene(s) PMM0038;
37053	-	TSS_014282	1000	247	0	0	Ai	antisense to gene(s) PMM0038;
37342	+	TSS_000323	1000	1210	0	3	P	18nt upstream of gene PMM0039;
41761	-	TSS_014294	1000	145	0	0	I	within gene(s) PMM0042;
44029	+	TSS_000344	1000	338	0	1	Ai	antisense to gene(s) PMM0043;
44220	-	TSS_014327	1000	1003	0	0	P	30nt upstream of gene PMM0043;
44838	-	TSS_014330	1000	371	0	2	Ai	antisense to gene(s) PMM0044;
46192	+	TSS_000355	1000	111	0	7	I	within gene(s) PMM0044;
46967	-	TSS_014336	1000	211	0	7	IAd	within gene(s) PMM0045; antisense to gene(s) PMM0044 (3nt downstream);
47672	-	TSS_014342	1000	201	0	5	I	within gene(s) PMM0045;
47824	+	TSS_000363	1000	1337	0	4	Ai	antisense to gene(s) PMM0045;
49002	+	TSS_000368	1000	392	0	2	P	15nt upstream of gene PMM0046;
50616	+	TSS_000372	1000	607	0	0	P	56nt upstream of gene PMM0048;
52823	+	TSS_000424	1000	472	0	5	P	44nt upstream of gene PMM0050;
52948	-	TSS_014357	1000	131	0	1	PAi	187nt upstream of gene PMM0049; antisense to gene(s) PMM0050;
54384	-	TSS_014363	1000	355	0	63	I	within gene(s) PMM0051;
56528	-	TSS_014400	1000	4036	0	0	O	-
56601	-	TSS_014401	1000	1566	0	0	O	-
58622	-	TSS_014433	1000	160	0	3	I	within gene(s) PMM0055;
58631	-	TSS_014434	1000	93	0	0	I	within gene(s) PMM0055;
58796	+	TSS_000439	1000	6465	0	2	O	-
59479	+	TSS_000447	1000	1183	0	1	P	17nt upstream of gene PMM0057;
63006	+	TSS_000471	1000	5405	0	2	IP	within gene(s) PMM0057; 126nt upstream of gene PMM0058;
63116	+	TSS_000473	1000	301	0	2	P	16nt upstream of gene PMM0058;
63461	+	TSS_000483	1000	146	0	3	I	within gene(s) PMM0058;
66139	+	TSS_000493	1000	112	0	0	I	within gene(s) PMM0060;
66518	+	TSS_000496	1000	202	0	2	I	within gene(s) PMM0060;
66654	+	TSS_000499	1000	79	0	1	I	within gene(s) PMM0060;
67529	-	TSS_014465	1000	148	0	2	P	16nt upstream of gene PMM0061;

67586	+	TSS_000507	1000	1846	0	2 P	66nt upstream of gene PMM0062;
67893	+	TSS_000516	1000	541	0	2 I	within gene(s) PMM0063;
68237	+	TSS_000555	1000	243	0	21 I	within gene(s) PMM0063;
68394	-	TSS_014482	1000	237	0	0 Ai	antisense to gene(s) PMM0063;
68452	+	TSS_000595	1000	382	0	21 I	within gene(s) PMM0063;
69179	-	TSS_014492	1000	136	0	1 IP	within gene(s) PMM0065; 143nt upstream of gene PMM0064;
70887	-	TSS_014503	1000	90	0	0 I	within gene(s) PMM0065;
72400	-	TSS_014518	1000	282	0	0 P	32nt upstream of gene PMM0068;
73910	-	TSS_014521	1000	269	0	0 Ai	antisense to gene(s) PMM0069;
74118	-	TSS_014523	1000	87	0	0 Ai	antisense to gene(s) PMM0069;
75013	+	TSS_000645	1000	58	0	0 Ai	antisense to gene(s) PMM0070;
75278	-	TSS_014527	1000	70	0	0 I	within gene(s) PMM0070;
77020	+	TSS_000647	1000	73	0	2 Ai	antisense to gene(s) PMM0072;
78158	-	TSS_014552	1000	169	0	0 I	within gene(s) PMM0073;
78218	-	TSS_014553	1000	209	0	0 I	within gene(s) PMM0073;
78295	-	TSS_014555	1000	458	0	1 I	within gene(s) PMM0073;
79154	-	TSS_014587	1000	779	0	4 P	33nt upstream of gene PMM0073;
79823	+	TSS_000655	1000	112	0	0 P	22nt upstream of gene PMM0075;
80192	-	TSS_014591	1000	88	0	0 Ai	antisense to gene(s) PMM0075;
80289	+	TSS_000660	1000	369	0	14 I	within gene(s) PMM0075;
82229	-	TSS_014598	1000	148	0	0 Ai	antisense to gene(s) PMM0076;
83123	+	TSS_000691	1000	249	0	3 I	within gene(s) PMM0077;
88866	+	TSS_000709	1000	192	0	6 I	within gene(s) PMM0083;
89200	-	TSS_014605	1000	160	0	4 Ai	antisense to gene(s) PMM0083;
90051	+	TSS_000721	1000	1090	0	1 I	within gene(s) PMM0084;
91200	+	TSS_000731	1000	1766	0	7 IP	within gene(s) PMM0084; 60nt upstream of gene PMM0085;
91487	-	TSS_014615	1000	195	0	0 Ai	antisense to gene(s) PMM0085;
91506	+	TSS_000736	1000	212	0	0 I	within gene(s) PMM0085;
91711	-	TSS_014617	1000	848	0	1 Ai	antisense to gene(s) PMM0085;
92216	-	TSS_014618	1000	142	0	0 I	within gene(s) PMM0086;
92293	-	TSS_014621	1000	3117	0	3 P	20nt upstream of gene PMM0086;
92350	+	TSS_000746	1000	2741	0	1 P	40nt upstream of gene PMM0087;
92441	+	TSS_000752	1000	191	0	15 I	within gene(s) PMM0087;
92747	+	TSS_000769	1000	1834	0	1 P	17nt upstream of gene PMM0088;
93143	-	TSS_014632	1000	1026	0	2 O	-
93899	+	TSS_000778	1000	84	0	1 I	within gene(s) PMM0089;
96428	+	TSS_000782	1000	401	0	2 P	24nt upstream of gene PMM0091;
96530	+	TSS_000795	1000	136	0	11 I	within gene(s) PMM0091;
96699	+	TSS_000815	1000	422	0	19 IP	within gene(s) PMM0091; 121nt upstream of gene PMM0092;
97181	+	TSS_000830	1000	8423	0	5 P	19nt upstream of gene PMM0093;
98015	-	TSS_014647	1000	192	0	0 I	within gene(s) PMM0095;
98676	-	TSS_014651	1000	260	0	1 P	16nt upstream of gene PMM0095;
99057	+	TSS_000836	1000	111	0	0 Ai	antisense to gene(s) PMM0096;
99177	+	TSS_000837	1000	138	0	0 Ai	antisense to gene(s) PMM0096;
104291	+	TSS_000856	1000	199	0	0 I	within gene(s) PMM0100;
105223	-	TSS_014665	1000	144	0	0 I	within gene(s) PMM0101;
105437	-	TSS_014669	1000	297	0	3 I	within gene(s) PMM0101;
105946	-	TSS_014676	1000	430	0	0 P	30nt upstream of gene PMM0101;
107782	-	TSS_014682	1000	156	0	5 I	within gene(s) PMM0103;
107836	-	TSS_014684	1000	101	0	0 I	within gene(s) PMM0103;
109827	+	TSS_000864	1000	112	0	0 Ai	antisense to gene(s) PMM0105;
110281	+	TSS_000865	1000	899	0	1 Ai	antisense to gene(s) PMM0106;
110766	-	TSS_014698	1000	12848	0	10 P	12nt upstream of gene PMM0106;
114981	-	TSS_014707	1000	162	0	3 IP	within gene(s) PMM0115; 35nt upstream of gene PMM0114;
116133	-	TSS_014733	1000	70	0	3 I	within gene(s) PMM0115;
116412	-	TSS_014741	1000	66	0	8 P	9nt upstream of gene PMM0115;
116480	-	TSS_010218	1000	9543	0	0 P	20nt upstream of gene PMM0116;
117055	+	TSS_000922	1000	123	0	5 I	within gene(s) PMM0117;
118346	-	TSS_014750	1000	226	0	0 Ai	antisense to gene(s) PMM0118;
119698	-	TSS_014784	1000	395	0	3 P	16nt upstream of gene PMM0120;
119798	+	TSS_000933	1000	2206	0	2 P	28nt upstream of gene PMM0121;
119905	+	TSS_000937	1000	314	0	0 I	within gene(s) PMM0121;
120922	-	TSS_014793	1000	123	0	0 IP	within gene(s) PMM0123; 172nt upstream of gene PMM0122;
120990	-	TSS_014800	1000	754	0	13 IP	within gene(s) PMM0123; 240nt upstream of gene PMM0122;
121450	-	TSS_014816	1000	102	0	12 I	within gene(s) PMM0123;
121764	-	TSS_014822	1000	259	0	0 P	29nt upstream of gene PMM0123;
122897	+	TSS_000946	1000	906	0	1 O	-
123251	+	TSS_000948	1000	543	0	1 P	68nt upstream of gene PMM0126;
123709	-	TSS_014832	1000	55	0	2 Ai	antisense to gene(s) PMM0126;
123779	+	TSS_000953	1000	2133	0	2 I	within gene(s) PMM0126;
125323	+	TSS_000965	1000	1778	0	1 P	35nt upstream of gene PMM0128;
125778	+	TSS_001001	1000	132	0	27 I	within gene(s) PMM0128;
128699	-	TSS_014848	1000	72	0	0 I	within gene(s) PMM0131;
129278	-	TSS_014851	1000	146	0	3 I	within gene(s) PMM0131;
130071	-	TSS_014856	1000	104	0	3 P	34nt upstream of gene PMM0131;
130794	+	TSS_001018	1000	1680	0	0 Ai	antisense to gene(s) PMM0133;
130945	+	TSS_001020	1000	135	0	0 Ai	antisense to gene(s) PMM0133;
131956	-	TSS_014865	1000	94	0	0 I	within gene(s) PMM0133;
132089	-	TSS_014867	1000	117	0	3 P	48nt upstream of gene PMM0133;
132109	+	TSS_001026	1000	762	0	3 P	39nt upstream of gene PMM0134;

132594	+	TSS_001042	1000	66	0	0	I	within gene(s) PMM0134;
132850	-	TSS_014870	1000	443	0	0	Ai	antisense to gene(s) PMM0134;
134228	-	TSS_014873	1000	1683	0	3	Ai	antisense to gene(s) PMM0135;
134332	+	TSS_001051	1000	217	0	0	P	17nt upstream of gene PMM0136;
135334	+	TSS_001054	1000	162	0	0	IP	within gene(s) PMM0136; 37nt upstream of gene PMM0137;
136633	+	TSS_001059	1000	109	0	0	I	within gene(s) PMM0138;
139538	-	TSS_014885	1000	114	0	3	IAd	within gene(s) PMM0143; antisense to gene(s) PMM0142 (22nt downstream);
139888	+	TSS_001065	1000	1752	0	1	Ai	antisense to gene(s) PMM0143;
140081	-	TSS_014893	1000	696	0	0	I	within gene(s) PMM0143;
140446	-	TSS_014896	1000	79	0	1	P	20nt upstream of gene PMM0143;
141294	-	TSS_014931	1000	107	0	0	I	within gene(s) PMM0144;
141905	-	TSS_014949	1000	881	0	0	P	38nt upstream of gene PMM0144;
141924	+	TSS_001070	1000	528	0	5	P	34nt upstream of gene PMM0145;
142219	+	TSS_001093	1000	165	0	8	IP	within gene(s) PMM0145; 83nt upstream of gene PMM0146;
142730	-	TSS_014954	1000	74	0	1	Ai	antisense to gene(s) PMM0146;
142788	+	TSS_001112	1000	62	0	3	I	within gene(s) PMM0146;
142832	-	TSS_014956	1000	189	0	0	Ai	antisense to gene(s) PMM0146;
143021	-	TSS_014962	1000	146	0	2	I	within gene(s) PMM0147;
143488	-	TSS_014969	1000	259	0	3	I	within gene(s) PMM0147;
143883	-	TSS_014979	1000	218	0	12	P	14nt upstream of gene PMM0147;
143931	+	TSS_001116	1000	113	0	3	P	23nt upstream of gene PMM0148;
144023	+	TSS_001117	1000	82	0	0	I	within gene(s) PMM0148;
144701	+	TSS_001120	1000	1620	0	3	P	15nt upstream of gene PMM0149;
144965	+	TSS_001131	1000	345	0	9	I	within gene(s) PMM0149;
145139	+	TSS_001136	1000	145	0	0	I	within gene(s) PMM0149;
145211	+	TSS_001137	1000	431	0	3	I	within gene(s) PMM0149;
145511	+	TSS_001155	1000	399	0	3	I	within gene(s) PMM0149;
145715	+	TSS_001168	1000	199	0	9	I	within gene(s) PMM0149;
146423	-	TSS_014996	1000	179	0	0	Ai	antisense to gene(s) PMM0149;
146611	+	TSS_001209	1000	106	0	2	IP	within gene(s) PMM0149; 202nt upstream of gene PMM0150;
146794	+	TSS_001212	1000	33574	0	2	P	19nt upstream of gene PMM0150;
147116	+	TSS_001224	1000	263	0	6	I	within gene(s) PMM0150;
147332	+	TSS_001231	1000	436	0	0	I	within gene(s) PMM0150;
147440	+	TSS_001236	1000	453	0	0	I	within gene(s) PMM0150;
147473	+	TSS_001238	1000	238	0	4	I	within gene(s) PMM0150;
147731	-	TSS_015002	1000	192	0	1	Ai	antisense to gene(s) PMM0150;
147971	+	TSS_001271	1000	221	0	6	I	within gene(s) PMM0150;
148515	+	TSS_001281	1000	166	0	1	P	169nt upstream of gene PMM0151;
149428	+	TSS_001286	1000	156	0	1	P	19nt upstream of gene PMM0152;
150290	+	TSS_001294	1000	211	0	7	I	within gene(s) PMM0152;
151121	+	TSS_001297	1000	71	0	0	Ai	antisense to gene(s) PMM0153;
151351	-	TSS_015018	1000	189	0	0	I	within gene(s) PMM0153;
151556	+	TSS_001301	1000	65	0	3	P	21nt upstream of gene PMM0154;
154617	-	TSS_015052	1000	3082	0	60	IP	within gene(s) PMM0159; 214nt upstream of gene PMM0158;
154809	-	TSS_015069	1000	372	0	18	I	within gene(s) PMM0159;
154860	-	TSS_015079	1000	114	0	33	I	within gene(s) PMM0159;
154964	-	TSS_015097	1000	122	0	3	I	within gene(s) PMM0159;
155061	-	TSS_015098	1000	756	0	4	P	18nt upstream of gene PMM0159;
155334	-	TSS_015103	1000	89	0	0	I	within gene(s) PMM0160;
155927	-	TSS_015116	1000	253	0	6	I	within gene(s) PMM0160;
156249	-	TSS_015126	1000	340	0	6	P	18nt upstream of gene PMM0160;
157478	-	TSS_015144	1000	222	0	0	P	26nt upstream of gene PMM0161;
159159	+	TSS_001319	1000	87	0	0	I	within gene(s) PMM0163;
160039	-	TSS_015153	1000	84	0	0	I	within gene(s) PMM0164;
160552	-	TSS_015186	1000	188	0	1	I	within gene(s) PMM0164;
160684	-	TSS_015195	1000	2024	0	5	P	18nt upstream of gene PMM0164;
161898	+	TSS_001329	1000	261	0	0	Ai	antisense to gene(s) PMM0167;
162263	-	TSS_015203	1000	309	0	2	P	61nt upstream of gene PMM0167;
162318	+	TSS_001331	1000	694	0	1	P	21nt upstream of gene PMM0168;
163563	-	TSS_015219	1000	114	0	0	I	within gene(s) PMM0169;
163802	-	TSS_015226	1000	408	0	0	P	32nt upstream of gene PMM0169;
163830	+	TSS_001336	1000	301	0	0	I	within gene(s) PMM0170;
165865	+	TSS_001343	1000	113	0	6	PAi	60nt upstream of gene PMM0172; antisense to gene(s) PMM0171;
165910	+	TSS_001346	1000	23006	0	3	P	15nt upstream of gene PMM0172;
166228	+	TSS_001376	1000	214	0	16	I	within gene(s) PMM0172;
166513	+	TSS_001406	1000	471	0	6	I	within gene(s) PMM0172;
172122	-	TSS_015248	1000	182	0	0	Ai	antisense to gene(s) PMM0177;
173360	-	TSS_015252	1000	160	0	2	P	17nt upstream of gene PMM0179;
173404	+	TSS_001482	1000	1077	0	0	P	16nt upstream of gene PMM0180;
176333	+	TSS_001488	1000	73	0	0	P	11nt upstream of gene PMM0185;
176661	+	TSS_001491	1000	71	0	5	I	within gene(s) PMM0185;
176904	-	TSS_015261	1000	114	0	0	Ai	antisense to gene(s) PMM0185;
178914	-	TSS_015265	1000	1449	0	1	I	within gene(s) PMM0187;
180315	+	TSS_001498	1000	517	0	0	Ai	antisense to gene(s) PMM0188;
182433	-	TSS_015271	1000	60	0	0	PAi	92nt upstream of gene PMM0189; antisense to gene(s) PMM0190;
183419	-	TSS_015274	1000	339	0	3	I	within gene(s) PMM0191;
185776	+	TSS_001509	1000	61	0	0	I	within gene(s) PMM0192;
187884	-	TSS_015294	1000	111	0	0	IP	within gene(s) PMM0194; 90nt upstream of gene PMM0193;
188529	+	TSS_001515	1000	73	0	0	Ai	antisense to gene(s) PMM0194;
188865	+	TSS_001516	1000	220	0	0	P	18nt upstream of gene PMM0195;

189007	+	TSS_001518	1000	234	0	6	I	within gene(s) PMM0195;
195017	-	TSS_015312	1000	200	0	1	P	46nt upstream of gene PMM0200;
195266	+	TSS_001543	1000	217	0	0	Ai	antisense to gene(s) PMM0201;
195331	-	TSS_015322	1000	61	0	3	I	within gene(s) PMM0201;
196137	-	TSS_015338	1000	5218	0	5	P	169nt upstream of gene PMM0202;
196154	+	TSS_001545	1000	205	0	0	Ad	antisense to gene(s) PMM0203 (8nt downstream);
196639	-	TSS_015343	1000	226	0	0	I	within gene(s) PMM0203;
197001	-	TSS_015349	1000	126	0	0	IP	within gene(s) PMM0204; 133nt upstream of gene PMM0203;
197448	-	TSS_015357	1000	188	0	1	IP	within gene(s) PMM0205; 88nt upstream of gene PMM0204;
197685	+	TSS_001550	1000	188	0	0	Ai	antisense to gene(s) PMM0205;
197771	-	TSS_015365	1000	259	0	0	I	within gene(s) PMM0205;
197876	-	TSS_015373	1000	95	0	5	I	within gene(s) PMM0205;
198069	-	TSS_015381	1000	125	0	1	P	34nt upstream of gene PMM0205;
198181	-	TSS_015383	1000	646	0	0	IP	within gene(s) PMM0206; 146nt upstream of gene PMM0205;
199333	-	TSS_015392	1000	119	0	0	I	within gene(s) PMM0207;
199933	-	TSS_015396	1000	201	0	3	I	within gene(s) PMM0207;
201442	+	TSS_001560	1000	3059	0	4	P	14nt upstream of gene PMM0208;
203219	-	TSS_015418	1000	421	0	0	I	within gene(s) PMM0209;
203802	-	TSS_015422	1000	218	0	0	I	within gene(s) PMM0209;
204777	-	TSS_015424	1000	121	0	5	P	41nt upstream of gene PMM0210;
204910	+	TSS_001617	1000	1103	0	4	P	82nt upstream of gene PMM0211;
205032	+	TSS_001621	1000	92	0	0	I	within gene(s) PMM0211;
205343	+	TSS_001635	1000	92	0	11	I	within gene(s) PMM0211;
205900	-	TSS_015439	1000	1470	0	1	Ai	antisense to gene(s) PMM0211;
206424	-	TSS_015441	1000	1996	0	2	IP	within gene(s) PMM0213; 172nt upstream of gene PMM0212;
207728	-	TSS_015450	1000	179	0	0	I	within gene(s) PMM0214;
207839	-	TSS_015451	1000	70	0	0	I	within gene(s) PMM0214;
208944	-	TSS_015460	1000	386	0	0	P	28nt upstream of gene PMM0214;
209113	+	TSS_001659	1000	79	0	1	P	31nt upstream of gene PMM0215;
210185	+	TSS_001662	1000	121	0	1	P	16nt upstream of gene PMM0216;
211988	-	TSS_015463	1000	968	0	2	Ai	antisense to gene(s) PMM0217;
212045	+	TSS_001665	1000	53	0	1	I	within gene(s) PMM0217;
212245	+	TSS_001668	1000	66	0	0	I	within gene(s) PMM0217;
212936	-	TSS_015465	1000	91	0	0	Ai	antisense to gene(s) PMM0217;
213029	+	TSS_001673	1000	570	0	2	P	33nt upstream of gene PMM0218;
214116	+	TSS_001681	1000	1274	0	2	P	17nt upstream of gene PMM0219;
214675	-	TSS_015472	1000	11989	0	3	P	19nt upstream of gene PMM0220;
215929	+	TSS_001685	1000	82	0	0	I	within gene(s) PMM0222;
216078	+	TSS_001686	1000	408	0	0	I	within gene(s) PMM0222;
216745	+	TSS_001696	1000	560094	0	7	P	62nt upstream of gene PMM0223;
216760	+	TSS_001698	1000		0	0	P	47nt upstream of gene PMM0223;
217197	+	TSS_001906	1000	13390	0	135	I	within gene(s) PMM0223;
217978	+	TSS_002190	1000	3125	0	3	P	24nt upstream of gene PMM0224;
218599	+	TSS_002225	1000	386	0	30	I	within gene(s) PMM0224;
219845	+	TSS_002253	1000	707	0	1	Ai	antisense to gene(s) PMM0226;
219897	-	TSS_015514	1000	17	0	0	IP	within gene(s) PMM0226; 149nt upstream of gene PMM0225;
220009	+	TSS_002255	1000	603	0	0	Ai	antisense to gene(s) PMM0226;
220578	-	TSS_015598	1000	481	0	15	I	within gene(s) PMM0226;
221632	-	TSS_015707	1000	2678	0	3	P	24nt upstream of gene PMM0226;
221652	+	TSS_002275	1000	71	0	0	Ad	antisense to gene(s) PMM0227 (12nt downstream);
221701	+	TSS_002276	1000	289	0	0	Ai	antisense to gene(s) PMM0227;
221978	+	TSS_002277	1000	112	0	0	Ai	antisense to gene(s) PMM0227;
223706	-	TSS_015742	1000	1129	0	1	I	within gene(s) PMM0228;
223847	-	TSS_015744	1000	224	0	1	P	140nt upstream of gene PMM0228;
225576	+	TSS_002284	1000	1686	0	0	P	14nt upstream of gene PMM0231;
225783	+	TSS_002288	1000	293	0	0	P	26nt upstream of gene PMM0232;
227265	-	TSS_015753	1000	116	0	0	IP	within gene(s) PMM0234; 96nt upstream of gene PMM0233;
227959	+	TSS_002292	1000	891	0	0	P	28nt upstream of gene PMM0235;
228037	+	TSS_002293	1000	163	0	0	I	within gene(s) PMM0235;
229314	-	TSS_015770	1000	284	0	0	O	-
229472	+	TSS_002309	1000	69	0	2	P	167nt upstream of gene PMM0237;
230878	-	TSS_015775	1000	320	0	5	Ai	antisense to gene(s) PMM0238;
232150	+	TSS_002332	1000	62	0	0	I	within gene(s) PMM0238;
232913	-	TSS_015782	1000	91	0	0	Ai	antisense to gene(s) PMM0238;
237520	-	TSS_015811	1000	751	0	15	IP	within gene(s) PMM0244; 196nt upstream of gene PMM0243;
237737	-	TSS_015823	1000	393	0	4	I	within gene(s) PMM0244;
237987	-	TSS_015826	1000	122	0	4	IP	within gene(s) PMM0245; 68nt upstream of gene PMM0244;
238520	-	TSS_015839	1000	1471	0	2	P	20nt upstream of gene PMM0245;
238693	+	TSS_002343	1000	2431	0	4	P	12nt upstream of gene PMM0246;
239672	-	TSS_015845	1000	204	0	4	Ai	antisense to gene(s) PMM0247;
241176	-	TSS_015848	1000	306	0	3	IP	within gene(s) PMM0250; 35nt upstream of gene PMM0249;
242059	+	TSS_002350	1000	200	0	0	PAi	162nt upstream of gene PMM0252; antisense to gene(s) PMM0251;
242165	-	TSS_015853	1000	121	0	4	P	24nt upstream of gene PMM0251;
242185	+	TSS_002352	1000	2370	0	2	P	36nt upstream of gene PMM0252;
242483	+	TSS_002357	1000	388	0	0	P	19nt upstream of gene PMM0253;
244978	+	TSS_002362	1000	253	0	4	Ai	antisense to gene(s) PMM0255;
245524	-	TSS_015860	1000	878	0	11	IP	within gene(s) PMM0256; 219nt upstream of gene PMM0255;
245888	-	TSS_015867	1000	354	0	2	I	within gene(s) PMM0256;
246248	+	TSS_002366	1000	4629	0	4	Ai	antisense to gene(s) PMM0256;
246731	-	TSS_015870	1000	898	0	1	IP	within gene(s) PMM0257; 20nt upstream of gene PMM0256;

248216	-	TSS_015880	1000	345	0	18	IP	within gene(s) PMM0258; 190nt upstream of gene PMM0257;
249242	-	TSS_015984	1000	1558	0	2	I	within gene(s) PMM0258;
249485	+	TSS_002385	1000	152	0	2	P	22nt upstream of gene PMM0259;
251358	+	TSS_002391	1000	172	0	3	Ai	antisense to gene(s) PMM0261;
252586	+	TSS_002398	1000	798562	0	8	P	46nt upstream of gene PMM0263;
252875	+	TSS_002439	1000	5646	0	9	I	within gene(s) PMM0263;
253072	+	TSS_002460	1000	9359	0	0	I	within gene(s) PMM0263;
254055	-	TSS_016021	1000	509	0	0	Ai	antisense to gene(s) PMM0263;
254181	+	TSS_002563	1000	111	0	4	P	1nt upstream of gene PMM0264;
255509	+	TSS_002566	1000	157	0	1	I	within gene(s) PMM0265;
258382	+	TSS_002605	1000	147	0	0	Ai	antisense to gene(s) PMM0268;
258478	-	TSS_016059	1000	241	0	30	I	within gene(s) PMM0268;
258539	+	TSS_002606	1000	521	0	0	Ai	antisense to gene(s) PMM0268;
258616	-	TSS_016075	1000	218	0	2	P	12nt upstream of gene PMM0268;
261925	+	TSS_002620	1000	391	0	0	P	23nt upstream of gene PMM0272;
263543	+	TSS_002624	1000	460	0	0	Ai	antisense to gene(s) PMM0274;
264013	+	TSS_002625	1000	101	0	0	Ai	antisense to gene(s) PMM0274;
264640	+	TSS_002627	1000	72	0	2	P	18nt upstream of gene PMM0275;
268849	+	TSS_002639	1000	121	0	0	IP	within gene(s) PMM0278; 175nt upstream of gene PMM0279;
269162	+	TSS_002643	1000	735	0	2	I	within gene(s) PMM0279;
271937	-	TSS_016138	1000	550	0	0	IP	within gene(s) PMM0282; 173nt upstream of gene PMM0281;
272594	-	TSS_016154	1000	698	0	1	P	27nt upstream of gene PMM0282;
273322	+	TSS_002655	1000	103	0	3	I	within gene(s) PMM0284;
274872	-	TSS_016158	1000	206	0	0	I	within gene(s) PMM0285;
274917	+	TSS_002658	1000	127	0	0	Ai	antisense to gene(s) PMM0285;
280333	-	TSS_016170	1000	1512	0	1	IP	within gene(s) PMM0290; 149nt upstream of gene PMM0289;
283519	-	TSS_016216	1000	436	0	13	I	within gene(s) PMM0293;
283811	-	TSS_016241	1000	3629	0	0	IP	within gene(s) PMM0294; 52nt upstream of gene PMM0293;
283904	-	TSS_016246	1000	5625	0	4	IP	within gene(s) PMM0294; 145nt upstream of gene PMM0293;
284179	-	TSS_016253	1000	1769	0	10	P	53nt upstream of gene PMM0294;
284302	-	TSS_016259	1000	4763	0	2	PAi	176nt upstream of gene PMM0294; antisense to gene(s) PMM0295;
284638	+	TSS_002694	1000	416	0	0	I	within gene(s) PMM0296;
284851	+	TSS_002699	1000	292	0	7	I	within gene(s) PMM0296;
285370	+	TSS_002713	1000	119	0	0	I	within gene(s) PMM0296;
285545	-	TSS_016265	1000	150	0	1	Ai	antisense to gene(s) PMM0296;
285768	+	TSS_002716	1000	20709	0	2	P	9nt upstream of gene PMM0297;
286195	+	TSS_002745	1000	768	0	9	IP	within gene(s) PMM0299; 123nt upstream of gene PMM0300;
286270	+	TSS_002755	1000	1312	0	7	IP	within gene(s) PMM0299; 48nt upstream of gene PMM0300;
286877	+	TSS_002770	1000	182	0	1	Ai	antisense to gene(s) PMM0301;
287552	-	TSS_016304	1000	1179	0	1	PAi	83nt upstream of gene PMM0301; antisense to gene(s) PMM0302;
287702	-	TSS_016306	1000	337	0	4	PAi	233nt upstream of gene PMM0301; antisense to gene(s) PMM0302;
290102	+	TSS_002778	1000	202	0	0	Ai	antisense to gene(s) PMM0303;
290195	+	TSS_002779	1000	627	0	0	Ai	antisense to gene(s) PMM0303;
291317	-	TSS_016313	1000	319	0	0	I	within gene(s) PMM0304;
291416	-	TSS_016314	1000	134	0	0	I	within gene(s) PMM0304;
292892	-	TSS_016319	1000	115	0	5	I	within gene(s) PMM0304;
293682	+	TSS_002782	1000	247	0	3	P	28nt upstream of gene PMM0305;
294128	-	TSS_016323	1000	469	0	1	Ai	antisense to gene(s) PMM0305;
294947	-	TSS_016326	1000	71	0	1	P	21nt upstream of gene PMM0307;
299042	-	TSS_016344	1000	1190	0	5	P	18nt upstream of gene PMM0311;
299836	-	TSS_016360	1000	249	0	0	I	within gene(s) PMM0312;
299950	-	TSS_016361	1000	219	0	0	I	within gene(s) PMM0312;
300325	-	TSS_016364	1000	550	0	9	P	75nt upstream of gene PMM0312;
300858	-	TSS_016373	1000	4552	0	7	P	21nt upstream of gene PMM0313;
301023	+	TSS_002803	1000	1218	0	0	Ai	antisense to gene(s) PMM0314;
302590	-	TSS_016519	1000	388	0	2	P	3nt upstream of gene PMM0315;
303256	+	TSS_002835	1000	702	0	12	P	24nt upstream of gene PMM0317;
305442	-	TSS_016534	1000	144	0	0	I	within gene(s) PMM0320;
305727	-	TSS_016550	1000	1582	0	7	IP	within gene(s) PMM0321; 174nt upstream of gene PMM0320;
306125	-	TSS_016557	1000	128	0	0	I	within gene(s) PMM0321;
306386	-	TSS_016559	1000	941	0	2	P	15nt upstream of gene PMM0321;
308758	+	TSS_002853	1000	14712	0	3	Ai	antisense to gene(s) PMM0324;
309008	+	TSS_002859	1000	246	0	0	Ai	antisense to gene(s) PMM0324;
309757	-	TSS_016603	1000	315	0	0	P	22nt upstream of gene PMM0324;
309786	+	TSS_002869	1000	16768	0	3	P	20nt upstream of gene PMM0325;
309929	-	TSS_016606	1000	4570	0	2	PAi	194nt upstream of gene PMM0324; antisense to gene(s) PMM0325;
310023	+	TSS_002878	1000	1004	0	0	I	within gene(s) PMM0325;
310271	+	TSS_002892	1000	473	0	7	IP	within gene(s) PMM0325; 234nt upstream of gene PMM0326;
310480	+	TSS_002901	1000	400	0	2	P	25nt upstream of gene PMM0326;
310880	+	TSS_002942	1000	287	0	12	I	within gene(s) PMM0326;
312446	-	TSS_016674	1000	2808	0	3	P	16nt upstream of gene PMM0327;
312554	+	TSS_002964	1000	1830	0	0	O	-
312729	+	TSS_002977	1000	367	0	10	O	-
312873	-	TSS_016677	1000	814	0	1	O	-
313374	-	TSS_016694	1000	612	0	1	O	-
313535	-	TSS_016699	1000	1908	0	7	O	-
313616	-	TSS_016704	1000	403	0	3	O	-
314130	+	TSS_003195	1000		0	4	O	-
314529	-	TSS_016731	1000	3194	0	3	O	-
314959	+	TSS_003214	1000	1061	0	0	O	-

315080	+	TSS_003215	1000		0	12	O	-
315599	-	TSS_016750	1000	138	0	0	O	-
315908	-	TSS_016759	1000	637	0	8	O	-
316065	-	TSS_016765	1000	693	0	6	O	-
316220	-	TSS_016771	1000	1403	0	4	O	-
316340	-	TSS_016772	1000	596	0	3	O	-
318015	-	TSS_016830	1000		0	4	O	-
319267	-	TSS_016834	1000	671	0	0	P	0nt upstream of gene PMM0329;
320607	+	TSS_003854	1000	92	0	0	Ai	antisense to gene(s) PMM0331;
322362	+	TSS_003856	1000	413	0	5	P	0nt upstream of gene PMM0333;
322674	+	TSS_003860	1000	110	0	0	I	within gene(s) PMM0333;
323620	-	TSS_016852	1000	1647	0	5	P	21nt upstream of gene PMM0334;
324251	-	TSS_016854	1000	1009	0	0	O	-
324470	-	TSS_016855	1000	105	0	0	O	-
324964	+	TSS_003870	1000	3029	0	1	I	within gene(s) PMM0335;
325557	-	TSS_016942	1000	69441	0	0	P	15nt upstream of gene PMM0336;
326449	-	TSS_016972	1000	1024	0	1	P	15nt upstream of gene PMM0337;
326712	+	TSS_003888	1000	2285	0	2	O	-
327570	-	TSS_016992	1000	4937	0	2	O	-
327681	+	TSS_003901	1000	70	0	3	Ai	antisense to gene(s) PMM0339;
328681	-	TSS_016997	1000	209	0	1	I	within gene(s) PMM0339;
329348	-	TSS_017002	1000	1709	0	0	P	157nt upstream of gene PMM0339;
330221	-	TSS_017006	1000	724	0	1	P	15nt upstream of gene PMM0341;
330306	+	TSS_003911	1000	1911	0	2	P	35nt upstream of gene PMM0342;
330978	-	TSS_017009	1000	626	0	3	O	-
333569	-	TSS_017029	1000	414	0	0	P	108nt upstream of gene PMM0347;
333947	-	TSS_017032	1000	10530	0	2	P	20nt upstream of gene PMM0348;
339021	+	TSS_003932	1000	288	0	1	P	18nt upstream of gene PMM0355;
339316	-	TSS_017041	1000	273	0	0	Ai	antisense to gene(s) PMM0355;
339953	+	TSS_003938	1000	15777	0	2	O	-
340175	-	TSS_017043	1000	141	0	2	O	-
340326	+	TSS_003940	1000	138	0	0	I	within gene(s) PMM0356;
342272	+	TSS_003945	1000	84	0	0	I	within gene(s) PMM0358;
343318	-	TSS_017051	1000	107	0	5	P	49nt upstream of gene PMM0359;
343464	+	TSS_003948	1000	113	0	4	O	-
345398	+	TSS_003958	1000	287	0	3	P	22nt upstream of gene PMM0363;
346829	+	TSS_003963	1000	187	0	3	O	-
347280	+	TSS_003964	1000	29790	0	1	P	16nt upstream of gene PMM0364;
347925	-	TSS_017062	1000	62	0	12	I	within gene(s) PMM0365;
348066	-	TSS_011864	1000	8542	0	1	P	21nt upstream of gene PMM0365;
348397	+	TSS_003997	1000	302	0	1	I	within gene(s) PMM0366;
348935	+	TSS_004006	1000	403	0	1	P	33nt upstream of gene PMM0367;
349333	-	TSS_017072	1000	255	0	3	Ai	antisense to gene(s) PMM0367;
349634	-	TSS_017084	1000	614	0	0	I	within gene(s) PMM0368;
349673	-	TSS_017086	1000	125	0	0	I	within gene(s) PMM0368;
349709	-	TSS_011873	1000	3487	0	1	P	18nt upstream of gene PMM0368;
350069	-	TSS_017087	1000	827	0	0	O	-
350388	+	TSS_004009	1000	321	0	1	O	-
350523	-	TSS_017092	1000	59488	0	4	O	-
351169	-	TSS_017097	1000	7094	0	6	O	-
352103	-	TSS_017108	1000	457	0	2	O	-
352188	+	TSS_004014	1000	2048	0	2	P	7nt upstream of gene PMM0369;
352957	+	TSS_004017	1000	1387	0	1	P	18nt upstream of gene PMM0370;
353381	+	TSS_004069	1000	455	0	15	I	within gene(s) PMM0370;
354391	+	TSS_004218	1000	906	0	69	I	within gene(s) PMM0370;
355246	+	TSS_004267	1000	115	0	3	IP	within gene(s) PMM0371; 244nt upstream of gene PMM0372;
355412	-	TSS_017130	1000	404	0	0	Ai	antisense to gene(s) PMM0371;
356326	+	TSS_004280	1000	473	0	3	IP	within gene(s) PMM0372; 51nt upstream of gene PMM0373;
358183	-	TSS_017137	1000	1490	0	2	P	16nt upstream of gene PMM0377;
358512	-	TSS_017147	1000	24265	0	2	P	22nt upstream of gene PMM0378;
358753	-	TSS_017150	1000	1589	0	2	O	-
359627	-	TSS_017152	1000	877	0	0	O	-
359991	-	TSS_017158	1000	1461	0	3	P	20nt upstream of gene PMM0379;
361663	+	TSS_004299	1000	145	0	0	O	-
362873	-	TSS_017167	1000	1659	0	4	P	140nt upstream of gene PMM0383;
366037	-	TSS_017185	1000	143	0	9	O	-
366442	-	TSS_017199	1000	415	0	10	O	-
366522	-	TSS_017205	1000	2507	0	4	O	-
367091	-	TSS_017210	1000	317	0	3	I	within gene(s) PMM0386;
367935	-	TSS_017212	1000	546	0	0	O	-
368274	+	TSS_004318	1000	1094	0	2	O	-
368596	+	TSS_004321	1000	117	0	5	P	17nt upstream of gene PMM0387;
370557	+	TSS_004328	1000	72	0	0	I	within gene(s) PMM0390;
370969	+	TSS_004331	1000	3175	0	3	P	24nt upstream of gene PMM0391;
373282	+	TSS_004339	1000	65	0	1	IP	within gene(s) PMM0392; 19nt upstream of gene PMM0393;
374217	-	TSS_017226	1000	106	0	4	Ad	antisense to gene(s) PMM0394 (9nt downstream);
374270	+	TSS_004341	1000	1398	0	0	P	97nt upstream of gene PMM0395;
376301	-	TSS_017243	1000	151	0	0	I	within gene(s) PMM0397;
380710	-	TSS_017253	1000	161	0	0	Ai	antisense to gene(s) PMM0402;
381726	+	TSS_004440	1000	1644	0	1	P	14nt upstream of gene PMM0403;

382963	+	TSS_004445	1000	117	0	1 O	-
383390	+	TSS_004447	1000	151	0	0 O	-
383585	+	TSS_004450	1000	7649	0	4 P	85nt upstream of gene PMM0405;
383961	+	TSS_004480	1000	109	0	21 I	within gene(s) PMM0405;
384035	-	TSS_017259	1000	320	0	1 Ai	antisense to gene(s) PMM0405;
384144	+	TSS_004505	1000	211	0	33 I	within gene(s) PMM0405;
384519	+	TSS_004541	1000	404	0	15 I	within gene(s) PMM0405;
384720	+	TSS_004566	1000	343	0	15 I	within gene(s) PMM0405;
385716	+	TSS_004579	1000	191	0	0 I	within gene(s) PMM0406;
386603	+	TSS_004593	1000	2651	0	1 Ai	antisense to gene(s) PMM0407;
386764	-	TSS_017359	1000	2019	0	144 I	within gene(s) PMM0407;
387174	-	TSS_017436	1000	45887	0	7 P	17nt upstream of gene PMM0407;
390631	+	TSS_004617	1000	196	0	3 P	26nt upstream of gene PMM0411;
390633	-	TSS_017453	1000	1221	0	2 P	72nt upstream of gene PMM0410;
392813	+	TSS_004623	1000	393	0	1 P	15nt upstream of gene PMM0414;
395057	+	TSS_004624	1000	96	0	0 Ai	antisense to gene(s) PMM0416;
395539	-	TSS_017466	1000	1138	0	2 P	16nt upstream of gene PMM0416;
395864	-	TSS_017469	1000	1717	0	5 O	-
396587	+	TSS_004627	1000	215	0	6 P	42nt upstream of gene PMM0419;
398147	+	TSS_004634	1000	720	0	1 P	32nt upstream of gene PMM0420;
398719	+	TSS_004637	1000	366	0	3 I	within gene(s) PMM0420;
401612	-	TSS_017482	1000	483	0	0 P	18nt upstream of gene PMM0422;
401747	+	TSS_004646	1000	3164	0	3 O	-
401864	-	TSS_017485	1000	129	0	1 O	-
402007	-	TSS_017486	1000	176	0	0 O	-
405868	-	TSS_017489	1000	79	0	0 Ai	antisense to gene(s) PMM0426;
409562	-	TSS_017499	1000	135	0	0 Ai	antisense to gene(s) PMM0430;
409694	+	TSS_004663	1000	71	0	0 I	within gene(s) PMM0430;
412572	-	TSS_017504	1000	104	0	0 I	within gene(s) PMM0434;
412935	+	TSS_004667	1000	132	0	6 Ai	antisense to gene(s) PMM0435;
413484	+	TSS_004670	1000	667	0	3 Ai	antisense to gene(s) PMM0435;
414359	-	TSS_017521	1000	448	0	1 P	44nt upstream of gene PMM0435;
414382	+	TSS_004676	1000	787	0	2 P	102nt upstream of gene PMM0436;
421671	-	TSS_017536	1000	96	0	0 P	22nt upstream of gene PMM0441;
422795	-	TSS_017541	1000	725	0	6 P	16nt upstream of gene PMM0443;
423280	+	TSS_004740	1000	116	0	2 Ai	antisense to gene(s) PMM0444;
423635	-	TSS_017555	1000	194	0	12 IP	within gene(s) PMM0445; 156nt upstream of gene PMM0444;
423797	-	TSS_017564	1000	331	0	6 I	within gene(s) PMM0445;
423944	-	TSS_017574	1000	396	0	6 I	within gene(s) PMM0445;
424706	-	TSS_017605	1000	527	0	6 I	within gene(s) PMM0445;
424790	-	TSS_017612	1000	676	0	18 I	within gene(s) PMM0445;
424880	-	TSS_017622	1000	1458	0	5 I	within gene(s) PMM0445;
425395	-	TSS_017647	1000	547	0	0 I	within gene(s) PMM0446;
425629	-	TSS_017668	1000	169	0	3 I	within gene(s) PMM0446;
426162	+	TSS_004762	1000	2722	0	4 P	8nt upstream of gene PMM0447;
426441	-	TSS_017675	1000	116	0	0 Ai	antisense to gene(s) PMM0447;
427201	+	TSS_004793	1000	589	0	30 I	within gene(s) PMM0448;
427279	+	TSS_004800	1000	674	0	6 I	within gene(s) PMM0448;
427558	+	TSS_004812	1000	551	0	3 I	within gene(s) PMM0448;
429988	-	TSS_017690	1000	302	0	1 Ai	antisense to gene(s) PMM0450;
430126	+	TSS_004848	1000	366	0	0 I	within gene(s) PMM0451;
430534	+	TSS_004856	1000	94	0	5 IAd	within gene(s) PMM0451; antisense to gene(s) PMM0452 (23nt downstream);
431182	-	TSS_017698	1000	92	0	23 I	within gene(s) PMM0452;
432317	-	TSS_017777	1000	6334	0	9 P	16nt upstream of gene PMM0452;
432436	+	TSS_004874	1000	211	0	0 O	-
433437	+	TSS_004878	1000	171	0	0 P	21nt upstream of gene PMM0454;
433897	+	TSS_004880	1000	79	0	2 I	within gene(s) PMM0454;
435917	-	TSS_017791	1000	293	0	0 P	19nt upstream of gene PMM0456;
440026	-	TSS_017803	1000	186	0	0 IP	within gene(s) PMM0461; 221nt upstream of gene PMM0460;
440888	-	TSS_017842	1000	96	0	6 IP	within gene(s) PMM0462; 118nt upstream of gene PMM0461;
441340	-	TSS_017857	1000	517	0	3 P	29nt upstream of gene PMM0462;
442840	-	TSS_017867	1000	2145	0	6 P	17nt upstream of gene PMM0465;
444471	+	TSS_004908	1000	171	0	1 PAi	157nt upstream of gene PMM0467; antisense to gene(s) PMM0466;
444597	-	TSS_017873	1000	72	0	1 P	44nt upstream of gene PMM0466;
445961	-	TSS_017911	1000	4230	0	4 P	42nt upstream of gene PMM0469;
445980	+	TSS_004916	1000	241	0	1 P	14nt upstream of gene PMM0470;
446903	+	TSS_004926	1000	107	0	0 IP	within gene(s) PMM0470; 167nt upstream of gene PMM0471;
447381	+	TSS_004931	1000	969	0	5 P	29nt upstream of gene PMM0472;
449593	-	TSS_017921	1000	93	0	1 I	within gene(s) PMM0473;
450716	-	TSS_017932	1000	1580	0	3 IP	within gene(s) PMM0475; 235nt upstream of gene PMM0474;
450998	+	TSS_004946	1000	486	0	1 Ai	antisense to gene(s) PMM0475;
451134	-	TSS_017945	1000	1183	0	8 P	4nt upstream of gene PMM0475;
451442	+	TSS_004950	1000	186	0	4 PAi	101nt upstream of gene PMM0477; antisense to gene(s) PMM0476;
452132	+	TSS_005032	1000	9282	0	8 I	within gene(s) PMM0477;
453247	+	TSS_005035	1000	348	0	0 P	22nt upstream of gene PMM0479;
454387	+	TSS_002819	1000	2574	0	3 P	17nt upstream of gene PMM0480;
455492	+	TSS_005063	1000	224	0	0 O	-
455674	-	TSS_017972	1000	101	0	4 O	-
456313	+	TSS_005137	1000	441	0	60 I	within gene(s) PMM0482;
456940	+	TSS_005165	1000	356	0	3 Ai	antisense to gene(s) PMM0483;

457405	+	TSS_005168	1000	147	0	1	Ai	antisense to gene(s) PMM0483;
457755	-	TSS_017991	1000	163	0	0	I	within gene(s) PMM0483;
457975	-	TSS_017993	1000	287	0	3	P	36nt upstream of gene PMM0483;
461083	+	TSS_005180	1000	99	0	0	IP	within gene(s) PMM0487; 242nt upstream of gene PMM0488;
463104	+	TSS_005183	1000	109	0	0	Ai	antisense to gene(s) PMM0489;
465270	-	TSS_018007	1000	167	0	0	IP	within gene(s) PMM0492; 16nt upstream of gene PMM0491;
465840	+	TSS_005192	1000	669	0	3	P	16nt upstream of gene PMM0493;
466192	+	TSS_005194	1000	497	0	1	I	within gene(s) PMM0493;
467553	+	TSS_005207	1000	293	0	0	I	within gene(s) PMM0494;
468497	-	TSS_018015	1000	900	0	7	I	within gene(s) PMM0495;
469000	-	TSS_018021	1000	101	0	0	P	32nt upstream of gene PMM0495;
470267	-	TSS_018060	1000	584	0	1	P	14nt upstream of gene PMM0496;
474859	-	TSS_018075	1000	372	0	0	IP	within gene(s) PMM0500; 37nt upstream of gene PMM0499;
475328	-	TSS_018081	1000	201	0	4	I	within gene(s) PMM0500;
475534	-	TSS_018083	1000	115	0	0	PAi	170nt upstream of gene PMM0500; antisense to gene(s) PMM0501;
475685	+	TSS_005221	1000	753	0	10	Ai	antisense to gene(s) PMM0502;
475749	+	TSS_005227	1000	1664	0	3	Ai	antisense to gene(s) PMM0502;
476045	-	TSS_018111	1000	1342	0	1	P	16nt upstream of gene PMM0502;
476504	+	TSS_005228	1000	90	0	0	I	within gene(s) PMM0503;
479540	-	TSS_018118	1000	211	0	2	P	24nt upstream of gene PMM0506;
480066	-	TSS_018128	1000	207	0	3	IP	within gene(s) PMM0508; 31nt upstream of gene PMM0507;
481882	-	TSS_018139	1000	241	0	1	P	18nt upstream of gene PMM0508;
482442	+	TSS_005242	1000	206	0	0	P	36nt upstream of gene PMM0510;
483044	-	TSS_018143	1000	745	0	2	Ai	antisense to gene(s) PMM0511;
483868	+	TSS_005244	1000	99	0	1	I	within gene(s) PMM0513;
486912	-	TSS_018149	1000	243	0	0	P	21nt upstream of gene PMM0515;
487573	+	TSS_005252	1000	139	0	0	I	within gene(s) PMM0516;
490430	+	TSS_005257	1000	162	0	3	I	within gene(s) PMM0518;
490706	+	TSS_005262	1000	7561	0	0	P	20nt upstream of gene PMM0519;
491260	+	TSS_005334	1000	1210	0	60	I	within gene(s) PMM0519;
491974	-	TSS_018163	1000	175	0	0	I	within gene(s) PMM0520;
493398	-	TSS_018172	1000	654	0	4	I	within gene(s) PMM0521;
493513	-	TSS_018175	1000	153	0	0	IP	within gene(s) PMM0522; 82nt upstream of gene PMM0521;
493630	+	TSS_005388	1000	176	0	3	Ai	antisense to gene(s) PMM0522;
493996	-	TSS_018181	1000	210	0	4	I	within gene(s) PMM0522;
494177	-	TSS_018185	1000	552	0	0	P	13nt upstream of gene PMM0522;
494529	+	TSS_005392	1000	349	0	0	Ai	antisense to gene(s) PMM0523;
495001	-	TSS_018191	1000	1099	0	8	P	12nt upstream of gene PMM0523;
496211	+	TSS_005396	1000	464	0	0	P	44nt upstream of gene PMM0525;
497254	+	TSS_005402	1000	315	0	6	I	within gene(s) PMM0525;
497550	+	TSS_005405	1000	3916	0	2	P	15nt upstream of gene PMM0526;
498249	+	TSS_005443	1000	206	0	27	I	within gene(s) PMM0526;
498873	+	TSS_005478	1000	629	0	21	I	within gene(s) PMM0526;
499089	+	TSS_005510	1000	465	0	41	I	within gene(s) PMM0526;
502682	-	TSS_018233	1000	804	0	13	P	24nt upstream of gene PMM0530;
503597	+	TSS_005527	1000	809	0	1	P	34nt upstream of gene PMM0532;
504905	+	TSS_005585	1000	196	0	1	I	within gene(s) PMM0533;
505257	-	TSS_018245	1000	110	0	0	Ai	antisense to gene(s) PMM0533;
505261	+	TSS_005586	1000	117	0	0	IP	within gene(s) PMM0533; 240nt upstream of gene PMM0534;
505757	+	TSS_005589	1000	418	0	0	I	within gene(s) PMM0534;
507310	+	TSS_005596	1000	172	0	0	P	62nt upstream of gene PMM0536;
508770	-	TSS_018249	1000	213	0	0	I	within gene(s) PMM0537;
510847	+	TSS_005613	1000	113	0	2	P	19nt upstream of gene PMM0540;
512559	+	TSS_005620	1000	193	0	0	Ai	antisense to gene(s) PMM0543;
512853	+	TSS_005622	1000	5899	0	3	Ai	antisense to gene(s) PMM0543;
513385	-	TSS_018316	1000	7121	0	5	P	24nt upstream of gene PMM0543;
513547	+	TSS_005633	1000	294	0	0	Ad	antisense to gene(s) PMM0544 (7nt downstream);
515373	-	TSS_018417	1000	251	0	36	IP	within gene(s) PMM0545; 240nt upstream of gene PMM0544;
516192	-	TSS_018506	1000	670	0	10	I	within gene(s) PMM0545;
516813	-	TSS_018524	1000	205	0	5	I	within gene(s) PMM0546;
516828	-	TSS_018528	1000	92	0	6	I	within gene(s) PMM0546;
516846	-	TSS_018529	1000	62	0	0	I	within gene(s) PMM0546;
516873	-	TSS_018530	1000	76	0	6	I	within gene(s) PMM0546;
516933	-	TSS_018536	1000		0	13	P	15nt upstream of gene PMM0546;
518485	-	TSS_018544	1000	216	0	0	P	102nt upstream of gene PMM0548;
518681	+	TSS_005665	1000	1859	0	2	P	44nt upstream of gene PMM0549;
519603	+	TSS_005671	1000	571	0	5	I	within gene(s) PMM0550;
520384	-	TSS_018550	1000	500	0	1	Ai	antisense to gene(s) PMM0550;
524032	-	TSS_018559	1000	134	0	2	Ai	antisense to gene(s) PMM0553;
524269	-	TSS_018561	1000	155	0	0	Ai	antisense to gene(s) PMM0553;
524632	-	TSS_018563	1000	4866	0	2	Ai	antisense to gene(s) PMM0553;
524671	+	TSS_005696	1000	352	0	0	IP	within gene(s) PMM0553; 191nt upstream of gene PMM0554;
524738	+	TSS_005697	1000	664	0	0	IP	within gene(s) PMM0553; 124nt upstream of gene PMM0554;
525973	-	TSS_018570	1000	536	0	0	P	18nt upstream of gene PMM0557;
526448	-	TSS_013314	1000	1876	0	2	P	15nt upstream of gene PMM0558;
527254	+	TSS_005718	1000	477	0	6	I	within gene(s) PMM0560;
528660	+	TSS_005728	1000	103	0	2	I	within gene(s) PMM0561;
531701	+	TSS_005754	1000	346	0	4	P	15nt upstream of gene PMM0565;
532356	-	TSS_018595	1000	493	0	1	Ai	antisense to gene(s) PMM0565;
532769	+	TSS_005766	1000	165	0	3	I	within gene(s) PMM0565;

534371	+	TSS_005771	1000	172	0	2	P	6nt upstream of gene PMM0567;
538166	+	TSS_005781	1000	975	0	2	P	179nt upstream of gene PMM0570;
539279	-	TSS_018612	1000	182	0	3	Ai	antisense to gene(s) PMM0572;
539865	+	TSS_005786	1000	403	0	0	Ad	antisense to gene(s) PMM0573 (15nt downstream);
539992	+	TSS_005787	1000	400	0	0	Ai	antisense to gene(s) PMM0573;
540221	-	TSS_013363	1000		0	12	P	97nt upstream of gene PMM0573;
540317	-	TSS_018628	1000	107	0	0	P	163nt upstream of gene PMM0573;
542147	-	TSS_018662	1000	3521	0	6	I	within gene(s) PMM0577;
543284	-	TSS_018683	1000	601	0	3	P	38nt upstream of gene PMM0578;
545216	+	TSS_005808	1000	366	0	1	Ai	antisense to gene(s) PMM0580;
546045	-	TSS_018690	1000	660	0	1	I	within gene(s) PMM0580;
546530	+	TSS_005812	1000	854	0	1	Ai	antisense to gene(s) PMM0581;
546844	-	TSS_013445	1000		0	5	I	17nt upstream of gene PMM0581;
547595	-	TSS_018729	1000	143	0	0	I	within gene(s) PMM0582;
548961	-	TSS_018736	1000	97	0	0	P	43nt upstream of gene PMM0583;
552915	-	TSS_018749	1000	103	0	3	I	within gene(s) PMM0585;
558188	+	TSS_005826	1000	150	0	1	P	14nt upstream of gene PMM0590;
561219	-	TSS_018764	1000	144	0	0	I	within gene(s) PMM0593;
562701	+	TSS_005834	1000	279	0	0	Ai	antisense to gene(s) PMM0594;
565420	-	TSS_018819	1000	916	0	1	I	within gene(s) PMM0596;
567864	+	TSS_005848	1000	109	0	0	Ai	antisense to gene(s) PMM0597;
569600	-	TSS_018828	1000	109	0	4	P	20nt upstream of gene PMM0599;
571068	-	TSS_018832	1000	107	0	1	Ai	antisense to gene(s) PMM0600;
572225	+	TSS_005865	1000	99	0	7	I	within gene(s) PMM0602;
573526	-	TSS_018837	1000	122	0	1	Ai	antisense to gene(s) PMM0603;
575199	-	TSS_018839	1000	209	0	0	P	16nt upstream of gene PMM0605;
577744	+	TSS_005882	1000	141	0	0	I	within gene(s) PMM0608;
578017	-	TSS_018845	1000	329	0	0	Ai	antisense to gene(s) PMM0608;
578318	+	TSS_005887	1000	154	0	0	IP	within gene(s) PMM0608; 247nt upstream of gene PMM0609;
579495	+	TSS_005891	1000	486	0	0	I	within gene(s) PMM0609;
582602	-	TSS_018857	1000	169	0	3	I	within gene(s) PMM0611;
582767	-	TSS_018860	1000	199	0	6	P	17nt upstream of gene PMM0611;
584058	-	TSS_018866	1000	247	0	2	I	within gene(s) PMM0613;
585123	+	TSS_005904	1000	1561	0	4	P	120nt upstream of gene PMM0614;
585737	+	TSS_005908	1000	383	0	1	IP	within gene(s) PMM0614; 160nt upstream of gene PMM0615;
586359	+	TSS_005916	1000	480	0	3	I	within gene(s) PMM0615;
587070	-	TSS_018870	1000	307	0	0	Ai	antisense to gene(s) PMM0616;
588622	+	TSS_005922	1000	1292	0	3	P	9nt upstream of gene PMM0618;
588874	+	TSS_005927	1000	241	0	1	I	within gene(s) PMM0618;
590670	-	TSS_018876	1000	826	0	3	Ai	antisense to gene(s) PMM0619;
591008	+	TSS_005958	1000	330	0	0	I	within gene(s) PMM0619;
593663	-	TSS_018881	1000	129	0	0	I	within gene(s) PMM0622;
594134	+	TSS_005965	1000	109	0	0	I	within gene(s) PMM0623;
594680	+	TSS_005968	1000	100	0	2	I	within gene(s) PMM0623;
595886	+	TSS_005970	1000	288	0	0	P	18nt upstream of gene PMM0626;
596385	-	TSS_018888	1000	1055	0	1	O	-
599253	+	TSS_006047	1000	763	0	2	P	15nt upstream of gene PMM0629;
600230	+	TSS_006048	1000	453	0	0	P	26nt upstream of gene PMM0631;
602377	-	TSS_018902	1000	560	0	2	I	within gene(s) PMM0633;
605795	+	TSS_006056	1000	203	0	4	P	18nt upstream of gene PMM0637;
610210	+	TSS_006070	1000	1987	0	2	P	16nt upstream of gene PMM0641;
610941	+	TSS_006075	1000	2768	0	3	P	17nt upstream of gene PMM0642;
611411	+	TSS_006094	1000	346	0	24	I	within gene(s) PMM0642;
614781	-	TSS_018958	1000	134	0	0	P	15nt upstream of gene PMM0644;
617157	-	TSS_018969	1000	8782	0	9	Ai	antisense to gene(s) PMM0646;
619598	+	TSS_006181	1000	109	0	1	P	18nt upstream of gene PMM0649;
620989	-	TSS_018983	1000	3453	0	6	P	13nt upstream of gene PMM0651;
621700	-	TSS_018988	1000	112	0	0	P	156nt upstream of gene PMM0652;
624976	+	TSS_006191	1000	354	0	4	I	within gene(s) PMM0658;
626301	+	TSS_006195	1000	251	0	0	I	within gene(s) PMM0659;
627045	-	TSS_018996	1000	285	0	1	O	-
627516	+	TSS_006197	1000	100	0	0	O	-
627971	-	TSS_019002	1000	3122	0	3	O	-
628169	+	TSS_006198	1000	402	0	0	O	-
628505	-	TSS_019027	1000	137	0	12	I	within gene(s) PMM0660;
628656	-	TSS_019030	1000	342	0	0	P	10nt upstream of gene PMM0660;
629577	-	TSS_019122	1000	828	0	48	I	within gene(s) PMM0661;
630363	-	TSS_019287	1000	1381	0	36	I	within gene(s) PMM0661;
631116	-	TSS_019393	1000	13447	0	2	I	within gene(s) PMM0661;
631165	+	TSS_006243	1000	223	0	0	P	27nt upstream of gene PMM0662;
633583	+	TSS_006254	1000	219	0	1	P	23nt upstream of gene PMM0664;
635224	-	TSS_019403	1000	108	0	0	IP	within gene(s) PMM0666; 40nt upstream of gene PMM0665;
635882	+	TSS_006260	1000	158	0	2	P	25nt upstream of gene PMM0667;
638760	-	TSS_019414	1000	114	0	3	IP	within gene(s) PMM0671; 217nt upstream of gene PMM0670;
643136	+	TSS_006285	1000	197	0	2	P	21nt upstream of gene PMM0675;
643669	+	TSS_006286	1000	291	0	0	P	25nt upstream of gene PMM0676;
644399	+	TSS_006291	1000	127	0	0	I	within gene(s) PMM0676;
644589	+	TSS_006296	1000	116	0	3	I	within gene(s) PMM0676;
644914	-	TSS_019428	1000	251	0	6	Ad	antisense to gene(s) PMM0676 (4nt downstream);
647282	-	TSS_019432	1000	1714	0	2	P	69nt upstream of gene PMM0678;

648438	+	TSS_006311	1000	250	0	0	Ai	antisense to gene(s) PMM0680;
649461	+	TSS_006315	1000	230	0	6	I	within gene(s) PMM0681;
651413	+	TSS_006319	1000	356	0	0	I	within gene(s) PMM0683;
652626	+	TSS_006323	1000	755	0	0	O	-
652926	+	TSS_004002	1000	120843	0	3	I	within gene(s) PMM0684;
653980	-	TSS_019452	1000	23297	0	2	O	-
655603	-	TSS_019458	1000	2581	0	3	I	within gene(s) PMM0687;
655934	-	TSS_019464	1000	4542	0	2	O	-
656679	-	TSS_019475	1000	336	0	0	P	87nt upstream of gene PMM0688;
656940	-	TSS_019481	1000	107	0	2	O	-
657528	-	TSS_019486	1000	708	0	6	O	-
659043	-	TSS_013967	1000	4198	0	3	P	15nt upstream of gene PMM0690;
659754	+	TSS_006357	1000	125	0	0	Ad	antisense to gene(s) PMM0691 (2nt downstream);
660094	-	TSS_013973	1000	98	0	6	P	133nt upstream of gene PMM0691;
660419	-	TSS_019505	1000	132	0	6	P	22nt upstream of gene PMM0692;
661103	-	TSS_019509	1000	157	0	0	I	within gene(s) PMM0693;
661982	+	TSS_006362	1000	596	0	4	O	-
663342	+	TSS_006366	1000	103	0	0	Ai	antisense to gene(s) PMM0696;
663678	-	TSS_019514	1000	168	0	6	P	16nt upstream of gene PMM0697;
664058	-	TSS_019517	1000	248	0	4	I	within gene(s) PMM0698;
665165	+	TSS_006371	1000	306	0	4	P	16nt upstream of gene PMM0699;
667290	+	TSS_006376	1000	180	0	2	P	16nt upstream of gene PMM0703;
668059	+	TSS_006383	1000	358	0	2	I	within gene(s) PMM0704;
668368	+	TSS_006384	1000	139	0	0	I	within gene(s) PMM0704;
669388	+	TSS_006389	1000	187	0	0	P	12nt upstream of gene PMM0705;
671507	-	TSS_019531	1000	119	0	0	I	within gene(s) PMM0707;
671615	-	TSS_019532	1000	170	0	0	I	within gene(s) PMM0707;
672523	-	TSS_019535	1000	125	0	0	I	within gene(s) PMM0708;
674151	+	TSS_006403	1000	2450	0	1	Ai	antisense to gene(s) PMM0709;
674830	-	TSS_019553	1000	447	0	1	I	within gene(s) PMM0709;
675828	+	TSS_006411	1000	1225	0	5	P	26nt upstream of gene PMM0710;
676471	-	TSS_019559	1000	144	0	6	Ai	antisense to gene(s) PMM0710;
678373	+	TSS_006417	1000	162	0	0	Ai	antisense to gene(s) PMM0712;
680440	-	TSS_019576	1000	950	0	0	P	15nt upstream of gene PMM0713;
680511	-	TSS_019577	1000	269	0	0	PAi	86nt upstream of gene PMM0713; antisense to gene(s) PMM0714;
680855	-	TSS_019578	1000	106	0	0	Ai	antisense to gene(s) PMM0714;
685615	-	TSS_019582	1000	992	0	5	P	13nt upstream of gene PMM0722;
686751	-	TSS_019585	1000	133	0	2	Ai	antisense to gene(s) PMM0723;
687667	+	TSS_006440	1000	732	0	0	IP	within gene(s) PMM0724; 61nt upstream of gene PMM0725;
688353	+	TSS_006464	1000	261	0	0	I	within gene(s) PMM0725;
688866	+	TSS_006470	1000	10627	0	3	O	-
688965	+	TSS_006475	1000	262	0	9	O	-
689153	+	TSS_006477	1000	337	0	0	O	-
689593	-	TSS_019603	1000	269	0	4	P	17nt upstream of gene PMM0726;
696382	-	TSS_019612	1000	96	0	0	P	18nt upstream of gene PMM0732;
698300	-	TSS_019615	1000	538	0	3	P	15nt upstream of gene PMM0734;
701382	+	TSS_006486	1000	108	0	0	P	23nt upstream of gene PMM0738;
702227	-	TSS_019631	1000	330	0	1	P	16nt upstream of gene PMM0739;
702332	+	TSS_006489	1000	1075	0	3	P	11nt upstream of gene PMM0740;
702516	-	TSS_019633	1000	350	0	0	O	-
704397	-	TSS_019669	1000	312	0	55	I	within gene(s) PMM0743;
705815	-	TSS_019768	1000	270	0	1	P	23nt upstream of gene PMM0743;
708607	-	TSS_019777	1000	684	0	6	P	14nt upstream of gene PMM0746;
709324	+	TSS_006571	1000	165	0	0	IP	within gene(s) PMM0747; 32nt upstream of gene PMM0748;
710247	+	TSS_006575	1000	218	0	1	IP	within gene(s) PMM0748; 23nt upstream of gene PMM0749;
711092	+	TSS_006584	1000	164	0	0	I	within gene(s) PMM0749;
711431	-	TSS_019784	1000	94	0	0	Ai	antisense to gene(s) PMM0749;
711539	+	TSS_006586	1000	4345	0	2	I	within gene(s) PMM0750;
711829	-	TSS_019787	1000	1051	0	6	Ai	antisense to gene(s) PMM0750;
712163	+	TSS_006588	1000	238	0	0	IP	within gene(s) PMM0750; 42nt upstream of gene PMM0751;
713805	+	TSS_006604	1000	174	0	0	I	within gene(s) PMM0753;
714106	-	TSS_019798	1000	147	0	2	Ai	antisense to gene(s) PMM0753;
714352	+	TSS_006617	1000	116	0	1	I	within gene(s) PMM0754;
714804	-	TSS_019802	1000	175	0	0	Ai	antisense to gene(s) PMM0754;
719033	+	TSS_006630	1000	205	0	6	I	within gene(s) PMM0757;
720208	+	TSS_006635	1000	116	0	0	Ai	antisense to gene(s) PMM0758;
720651	-	TSS_019859	1000	692	0	25	I	within gene(s) PMM0758;
721002	-	TSS_014217	1000		0	0	P	15nt upstream of gene PMM0758;
723763	-	TSS_019917	1000	671	0	24	I	within gene(s) PMM0760;
724438	-	TSS_019979	1000	152	0	6	I	within gene(s) PMM0760;
724578	-	TSS_019983	1000	2549	0	2	P	20nt upstream of gene PMM0760;
725488	+	TSS_006654	1000	154	0	4	P	25nt upstream of gene PMM0762;
728301	-	TSS_019992	1000	157	0	0	Ai	antisense to gene(s) PMM0764;
729981	-	TSS_020004	1000	133	0	3	I	within gene(s) PMM0766;
730435	+	TSS_006719	1000	1185	0	1	I	within gene(s) PMM0767;
732872	+	TSS_006732	1000	235	0	0	P	21nt upstream of gene PMM0769;
734112	-	TSS_020022	1000	153	0	4	Ai	antisense to gene(s) PMM0769;
734269	+	TSS_006750	1000	6225	0	4	P	18nt upstream of gene PMM0770;
734570	+	TSS_006758	1000	106	0	1	I	within gene(s) PMM0770;
736446	+	TSS_006779	1000	100	0	5	P	15nt upstream of gene PMM0772;

737771	-	TSS_020038	1000	163	0	14	I	within gene(s) PMM0774;
737882	-	TSS_020050	1000	305	0	0	I	within gene(s) PMM0774;
738561	+	TSS_006793	1000	524	0	0	Ai	antisense to gene(s) PMM0774;
738857	-	TSS_020089	1000	150	0	12	I	within gene(s) PMM0774;
739095	-	TSS_020100	1000	3171	0	4	P	13nt upstream of gene PMM0774;
739410	-	TSS_020106	1000	659	0	10	P	14nt upstream of gene PMM0775;
740068	+	TSS_006801	1000	694	0	2	P	38nt upstream of gene PMM0777;
741992	+	TSS_006829	1000	1445	0	0	P	15nt upstream of gene PMM0779;
742370	+	TSS_006835	1000	188	0	3	I	within gene(s) PMM0780;
744091	-	TSS_020193	1000	1766	0	2	P	43nt upstream of gene PMM0781;
746286	-	TSS_020205	1000	296	0	2	I	within gene(s) PMM0784;
746584	-	TSS_020211	1000	282	0	5	P	18nt upstream of gene PMM0784;
747387	-	TSS_020214	1000	510	0	5	I	within gene(s) PMM0785;
747664	-	TSS_020215	1000	185	0	2	P	15nt upstream of gene PMM0785;
749173	-	TSS_020221	1000	1267	0	4	I	within gene(s) PMM0787;
752029	-	TSS_020242	1000	186	0	8	P	25nt upstream of gene PMM0790;
752047	+	TSS_006865	1000	336	0	12	Ai	antisense to gene(s) PMM0791;
752129	+	TSS_006866	1000	100	0	0	Ai	antisense to gene(s) PMM0791;
755386	-	TSS_020247	1000	276	0	1	IP	within gene(s) PMM0795; 114nt upstream of gene PMM0794;
756926	-	TSS_020251	1000	119	0	0	P	17nt upstream of gene PMM0795;
757372	-	TSS_020252	1000	110	0	0	I	within gene(s) PMM0796;
757822	-	TSS_020259	1000	320	0	1	P	66nt upstream of gene PMM0796;
760238	-	TSS_020263	1000	94	0	11	P	6nt upstream of gene PMM0800;
762992	+	TSS_006874	1000	2772	0	0	P	16nt upstream of gene PMM0804;
763062	+	TSS_006882	1000	1309	0	17	I	within gene(s) PMM0804;
763761	+	TSS_006935	1000	160	0	0	Ai	antisense to gene(s) PMM0805;
764067	+	TSS_006938	1000	229	0	0	Ai	antisense to gene(s) PMM0806;
764468	-	TSS_020284	1000	167	0	3	P	26nt upstream of gene PMM0806;
768634	-	TSS_020291	1000	1957	0	1	P	16nt upstream of gene PMM0810;
769201	-	TSS_020295	1000	500	0	0	O	-
771730	-	TSS_020301	1000	120	0	0	P	22nt upstream of gene PMM0814;
772207	-	TSS_020305	1000	1195	0	1	O	-
773190	-	TSS_020323	1000	771	0	2	I	within gene(s) PMM0815;
773382	-	TSS_020352	1000	1262	0	10	IP	within gene(s) PMM0816; 189nt upstream of gene PMM0815;
773816	-	TSS_020404	1000	57347	0	6	P	16nt upstream of gene PMM0818;
774713	-	TSS_020416	1000	125	0	1	I	within gene(s) PMM0819;
774773	-	TSS_020419	1000	12393	0	3	P	18nt upstream of gene PMM0819;
775810	-	TSS_020425	1000	839	0	1	O	-
776471	-	TSS_020428	1000	199	0	4	O	-
777149	-	TSS_020435	1000	489	0	1	I	within gene(s) PMM0821;
778975	-	TSS_020453	1000	1967	0	7	P	14nt upstream of gene PMM0824;
779053	+	TSS_006974	1000	100	0	1	P	17nt upstream of gene PMM0825;
779674	-	TSS_020460	1000	178	0	0	Ai	antisense to gene(s) PMM0825;
780730	-	TSS_020463	1000	360	0	0	Ai	antisense to gene(s) PMM0825;
781041	+	TSS_007009	1000	210	0	15	I	within gene(s) PMM0825;
784835	+	TSS_007050	1000	198	0	0	I	within gene(s) PMM0829;
787909	-	TSS_020511	1000	192	0	9	I	within gene(s) PMM0831;
790206	-	TSS_014848	1000		0	1	I	17nt upstream of gene PMM0831;
796520	+	TSS_007070	1000	201	0	1	Ai	antisense to gene(s) PMM0839;
803506	-	TSS_020584	1000	109	0	6	I	within gene(s) PMM0844;
804298	-	TSS_020612	1000	474	0	1	P	14nt upstream of gene PMM0844;
807760	-	TSS_020621	1000	172	0	2	P	27nt upstream of gene PMM0847;
808842	+	TSS_007086	1000	1057	0	2	P	117nt upstream of gene PMM0851;
810059	-	TSS_020628	1000	98	0	3	IAd	within gene(s) PMM0853; antisense to gene(s) PMM0852 (24nt downstream);
810075	+	TSS_007090	1000	33	0	0	Ai	antisense to gene(s) PMM0853;
810098	-	TSS_020634	1000	142	0	13	I	within gene(s) PMM0853;
810119	-	TSS_020636	1000	115	0	9	I	within gene(s) PMM0853;
810137	-	TSS_020638	1000	79	0	0	I	within gene(s) PMM0853;
810173	-	TSS_020649	1000	637	0	41	I	within gene(s) PMM0853;
810194	-	TSS_020655	1000	302	0	0	I	within gene(s) PMM0853;
810209	-	TSS_020657	1000	239	0	6	I	within gene(s) PMM0853;
810234	-	TSS_014951	1000		0	0	P	16nt upstream of gene PMM0853;
811735	-	TSS_020662	1000	356	0	0	Ai	antisense to gene(s) PMM0854;
813572	-	TSS_020715	1000	2177	0	12	P	45nt upstream of gene PMM0856;
814913	+	TSS_007109	1000	2209	0	2	O	-
815258	-	TSS_020727	1000	101	0	0	O	-
815652	-	TSS_020728	1000	132	0	0	O	-
816028	+	TSS_007114	1000	114	0	0	I	within gene(s) PMM0858;
816976	+	TSS_007117	1000	608	0	0	O	-
817333	+	TSS_007119	1000	481	0	1	O	-
818060	-	TSS_020731	1000	121	0	0	O	-
818750	-	TSS_020733	1000	114	0	0	P	24nt upstream of gene PMM0860;
819403	-	TSS_020738	1000	212	0	2	O	-
820064	-	TSS_020741	1000	97	0	0	O	-
821072	-	TSS_020745	1000	97	0	2	I	within gene(s) PMM0863;
826307	-	TSS_020771	1000	722	0	6	P	24nt upstream of gene PMM0870;
827556	-	TSS_020775	1000	510	0	0	Ai	antisense to gene(s) PMM0871;
828957	+	TSS_007140	1000	4821	0	2	I	within gene(s) PMM0872;
830651	-	TSS_020786	1000	109	0	5	P	17nt upstream of gene PMM0873;
831934	+	TSS_007146	1000	151	0	0	Ai	antisense to gene(s) PMM0875;

834878	-	TSS_020798	1000	1528	0	3	I	within gene(s) PMM0877;
837146	-	TSS_020822	1000	734	0	2	P	38nt upstream of gene PMM0878;
840244	-	TSS_020827	1000	164	0	2	Ai	antisense to gene(s) PMM0879;
841241	-	TSS_020832	1000	117	0	0	I	within gene(s) PMM0880;
844697	-	TSS_020861	1000	3903	0	5	P	15nt upstream of gene PMM0883;
855371	+	TSS_007185	1000	140	0	3	I	within gene(s) PMM0893;
857107	+	TSS_007194	1000	95	0	1	Ai	antisense to gene(s) PMM0894;
857549	-	TSS_020905	1000	2450	0	2	P	18nt upstream of gene PMM0894;
858686	-	TSS_020928	1000	487	0	0	I	within gene(s) PMM0896;
858957	-	TSS_020931	1000	538	0	2	IP	within gene(s) PMM0897; 21nt upstream of gene PMM0896;
859899	+	TSS_007202	1000	194	0	0	Ai	antisense to gene(s) PMM0897;
860406	-	TSS_020949	1000	1882	0	1	I	within gene(s) PMM0897;
860980	-	TSS_020953	1000	594	0	2	P	63nt upstream of gene PMM0897;
862187	+	TSS_007213	1000	242	0	6	I	within gene(s) PMM0899;
863809	+	TSS_007219	1000	258	0	1	P	23nt upstream of gene PMM0901;
865759	+	TSS_007230	1000	1816	0	6	P	17nt upstream of gene PMM0902;
867288	+	TSS_007235	1000	140	0	0	I	within gene(s) PMM0906;
867687	+	TSS_007237	1000	113	0	0	Ai	antisense to gene(s) PMM0907;
867831	+	TSS_007239	1000	98	0	0	Ai	antisense to gene(s) PMM0907;
868133	+	TSS_007241	1000	113	0	0	Ai	antisense to gene(s) PMM0907;
869447	-	TSS_020979	1000	1684	0	4	P	16nt upstream of gene PMM0907;
869921	+	TSS_007247	1000	147	0	0	I	within gene(s) PMM0908;
870149	+	TSS_007250	1000	250	0	4	I	within gene(s) PMM0908;
873612	+	TSS_007287	1000	823	0	3	I	within gene(s) PMM0912;
874281	+	TSS_007293	1000	90	0	1	I	within gene(s) PMM0913;
874787	+	TSS_007294	1000	103	0	0	I	within gene(s) PMM0913;
879143	+	TSS_007301	1000	150	0	0	Ai	antisense to gene(s) PMM0917;
880878	+	TSS_007311	1000	117	0	0	Ai	antisense to gene(s) PMM0919;
881167	-	TSS_021010	1000	914	0	0	P	15nt upstream of gene PMM0919;
881330	+	TSS_007314	1000	361	0	1	P	37nt upstream of gene PMM0920;
881352	+	TSS_007315	1000	37561	0	0	P	15nt upstream of gene PMM0920;
883958	+	TSS_007618	1000	91	0	2	P	15nt upstream of gene PMM0922;
886557	+	TSS_007630	1000	1006	0	7	P	14nt upstream of gene PMM0926;
889213	+	TSS_007636	1000	105	0	0	Ai	antisense to gene(s) PMM0929;
891601	-	TSS_021061	1000	1057	0	4	P	23nt upstream of gene PMM0930;
894754	+	TSS_007649	1000	180	0	0	P	45nt upstream of gene PMM0936;
899977	+	TSS_007662	1000	926	0	3	P	13nt upstream of gene PMM0941;
900041	+	TSS_007669	1000	820	0	13	I	within gene(s) PMM0941;
901047	+	TSS_007711	1000	8304	0	9	P	14nt upstream of gene PMM0943;
901817	-	TSS_021079	1000	91	0	9	I	within gene(s) PMM0945;
905299	-	TSS_021099	1000	1491	0	2	P	14nt upstream of gene PMM0945;
905410	-	TSS_021102	1000	108	0	2	IP	within gene(s) PMM0946; 125nt upstream of gene PMM0945;
905848	-	TSS_021108	1000	167	0	2	I	within gene(s) PMM0946;
906046	-	TSS_021112	1000	91	0	3	I	within gene(s) PMM0946;
907099	-	TSS_021122	1000	767	0	2	P	6nt upstream of gene PMM0947;
913287	+	TSS_007729	1000	163	0	0	I	within gene(s) PMM0954;
913440	+	TSS_007731	1000	936	0	1	IP	within gene(s) PMM0954; 246nt upstream of gene PMM0955;
913823	+	TSS_007734	1000	260	0	2	I	within gene(s) PMM0955;
914442	+	TSS_007737	1000	112	0	0	O	-
914689	-	TSS_021139	1000	102	0	0	O	-
915624	+	TSS_007740	1000	481	0	2	P	2nt upstream of gene PMM0957;
916221	-	TSS_021152	1000	427	0	2	O	-
916530	-	TSS_021155	1000	561	0	1	P	16nt upstream of gene PMM0958;
918232	+	TSS_007749	1000	389	0	0	Ai	antisense to gene(s) PMM0960;
919141	-	TSS_021161	1000	314	0	1	I	within gene(s) PMM0961;
920682	-	TSS_021164	1000	251	0	0	Ai	antisense to gene(s) PMM0962;
922689	+	TSS_007776	1000	357	0	2	Ai	antisense to gene(s) PMM0963;
923470	+	TSS_007777	1000	103	0	1	Ai	antisense to gene(s) PMM0965;
923723	-	TSS_021192	1000	522	0	0	I	within gene(s) PMM0965;
923809	-	TSS_021194	1000	371	0	5	IP	within gene(s) PMM0966; 85nt upstream of gene PMM0965;
924547	-	TSS_021198	1000	848	0	0	I	within gene(s) PMM0966;
925793	+	TSS_007785	1000	256	0	2	IP	within gene(s) PMM0968; 65nt upstream of gene PMM0969;
926558	+	TSS_005119	1000	255832	0	5	P	10nt upstream of gene PMM0970;
926661	+	TSS_007807	1000	1759	0	37	I	within gene(s) PMM0970;
927441	+	TSS_007993	1000	3020	0	88	I	within gene(s) PMM0970;
929465	-	TSS_021235	1000	161	0	1	Ai	antisense to gene(s) PMM0972;
930151	+	TSS_008074	1000	184	0	7	IP	within gene(s) PMM0972; 101nt upstream of gene PMM0973;
931059	+	TSS_008086	1000	98	0	0	I	within gene(s) PMM0974;
933017	+	TSS_008092	1000	870	0	0	I	within gene(s) PMM0975;
936929	+	TSS_008097	1000	367	0	1	O	-
937963	+	TSS_008099	1000	672	0	1	P	15nt upstream of gene PMM0982;
939028	-	TSS_021253	1000	109	0	0	P	17nt upstream of gene PMM0983;
939168	+	TSS_008103	1000	237	0	2	O	-
942377	-	TSS_021268	1000	706	0	1	P	126nt upstream of gene PMM0988;
946441	+	TSS_008118	1000	217	0	0	Ai	antisense to gene(s) PMM0993;
948048	+	TSS_008123	1000		0	9	P	17nt upstream of gene PMM0996;
948628	+	TSS_008123	1000		0	1	O	-
949329	-	TSS_021293	1000	1173	0	1	O	-
950002	+	TSS_008138	1000	215	0	0	P	15nt upstream of gene PMM0999;
950681	+	TSS_008139	1000	96	0	0	P	15nt upstream of gene PMM1001;

956897	-	TSS_021298	1000	740	0	2	P	17nt upstream of gene PMM1005;
958314	-	TSS_021421	1000	203	0	0	P	15nt upstream of gene PMM1007;
961818	+	TSS_008201	1000	981	0	3	P	14nt upstream of gene PMM1013;
963397	-	TSS_021425	1000	101	0	1	P	14nt upstream of gene PMM1015;
965105	+	TSS_008209	1000	318	0	0	O	-
965725	-	TSS_021428	1000	108	0	1	P	238nt upstream of gene PMM1018a;
971676	-	TSS_021439	1000	166	0	0	P	4nt upstream of gene PMM1026;
972175	-	TSS_021440	1000	2037	0	2	O	-
973076	-	TSS_021443	1000	423	0	1	Ai	antisense to gene(s) PMM1028;
973232	+	TSS_008229	1000	957	0	0	O	-
974221	+	TSS_008234	1000	1481	0	3	P	27nt upstream of gene PMM1030;
975550	+	TSS_008241	1000	443	0	0	P	17nt upstream of gene PMM1032;
977043	+	TSS_008322	1000	1115	0	6	P	32nt upstream of gene PMM1033;
977946	+	TSS_008346	1000	124	0	0	I	within gene(s) PMM1033;
978424	+	TSS_008365	1000	281	0	1	I	within gene(s) PMM1033 PMM1034;
979901	+	TSS_015644	1000		0	1	O	-
981473	+	TSS_008374	1000	92	0	1	P	1nt upstream of gene PMM1038;
981600	+	TSS_008375	1000	184	0	0	I	within gene(s) PMM1038;
984201	+	TSS_008389	1000	95	0	3	I	within gene(s) PMM1040;
984870	+	TSS_008394	1000	680	0	5	I	within gene(s) PMM1041;
985398	+	TSS_008397	1000	119	0	0	IP	within gene(s) PMM1041; 91nt upstream of gene PMM1042;
985468	+	TSS_008399	1000	394	0	7	P	21nt upstream of gene PMM1042;
996144	-	TSS_021492	1000	613	0	0	IP	within gene(s) PMM1054; 183nt upstream of gene PMM1053;
997065	-	TSS_021500	1000	302	0	1	I	within gene(s) PMM1055;
998833	+	TSS_008421	1000	103	0	1	PAi	156nt upstream of gene PMM1058; antisense to gene(s) PMM1057;
998966	+	TSS_008423	1000	1036	0	0	P	23nt upstream of gene PMM1058;
1000663	-	TSS_021515	1000	269	0	2	P	16nt upstream of gene PMM1061;
1000796	-	TSS_021518	1000	7631	0	3	P	149nt upstream of gene PMM1061;
1000995	-	TSS_021520	1000	195	0	1	I	within gene(s) PMM1062;
1001106	+	TSS_008427	1000	739	0	1	Ai	antisense to gene(s) PMM1062;
1001916	-	TSS_021526	1000	114	0	0	I	within gene(s) PMM1062;
1002186	+	TSS_005367	1000	3197	0	8	P	13nt upstream of gene PMM1063;
1007850	+	TSS_008477	1000	7089	0	5	P	14nt upstream of gene PMM1066;
1008779	+	TSS_008503	1000	161	0	9	I	within gene(s) PMM1066;
1008963	+	TSS_008515	1000	1188	0	1	I	within gene(s) PMM1066;
1009058	+	TSS_008517	1000	159	0	3	I	within gene(s) PMM1066;
1009601	+	TSS_008549	1000	216	0	3	I	within gene(s) PMM1067;
1011525	+	TSS_008555	1000	129	0	11	P	21nt upstream of gene PMM1069;
1012326	+	TSS_008570	1000	297	0	6	IP	within gene(s) PMM1069; 154nt upstream of gene PMM1070;
1012395	+	TSS_008578	1000	120	0	12	IP	within gene(s) PMM1069; 85nt upstream of gene PMM1070;
1013842	-	TSS_021560	1000	174	0	0	Ai	antisense to gene(s) PMM1071;
1016820	-	TSS_021592	1000	241	0	3	IP	within gene(s) PMM1074; 241nt upstream of gene PMM1073;
1018128	-	TSS_021690	1000	3145	0	3	P	18nt upstream of gene PMM1074;
1018655	-	TSS_021747	1000	2310	0	10	I	within gene(s) PMM1075;
1019222	-	TSS_021849	1000	2139	0	12	I	within gene(s) PMM1075;
1019366	-	TSS_021860	1000	3182	0	1	P	30nt upstream of gene PMM1075;
1019487	+	TSS_008630	1000	1860	0	1	P	32nt upstream of gene PMM1076;
1020379	-	TSS_021867	1000	2997	0	2	I	within gene(s) PMM1077;
1021282	-	TSS_021874	1000	384	0	0	I	within gene(s) PMM1077;
1022762	-	TSS_021876	1000	251	0	0	I	within gene(s) PMM1080;
1023134	+	TSS_008647	1000	278	0	6	Ai	antisense to gene(s) PMM1080;
1023493	-	TSS_021925	1000	2704	0	1	P	19nt upstream of gene PMM1080;
1023698	+	TSS_008655	1000	168	0	3	P	26nt upstream of gene PMM1081;
1024480	+	TSS_008664	1000	607	0	4	I	within gene(s) PMM1081;
1028961	+	TSS_008674	1000	142	0	0	IP	within gene(s) PMM1084; 27nt upstream of gene PMM1085;
1029223	-	TSS_021937	1000	328	0	0	Ai	antisense to gene(s) PMM1085;
1029801	-	TSS_021939	1000	381	0	0	Ai	antisense to gene(s) PMM1085;
1033430	-	TSS_022195	1000	718	0	129	I	within gene(s) PMM1088;
1034417	-	TSS_022375	1000	596	0	48	I	within gene(s) PMM1088;
1034569	-	TSS_022398	1000	15472	0	2	P	17nt upstream of gene PMM1088;
1035252	+	TSS_008723	1000	476	0	1	P	14nt upstream of gene PMM1090;
1036076	+	TSS_008730	1000	453	0	0	I	within gene(s) PMM1090;
1037053	+	TSS_008743	1000	151	0	1	I	within gene(s) PMM1091;
1037521	+	TSS_008746	1000	261	0	0	IP	within gene(s) PMM1091; 41nt upstream of gene PMM1092;
1037579	-	TSS_022409	1000	161	0	0	Ai	antisense to gene(s) PMM1092;
1038020	+	TSS_008748	1000	173	0	0	I	within gene(s) PMM1092;
1039004	+	TSS_008755	1000	131	0	0	I	within gene(s) PMM1093;
1041398	-	TSS_022419	1000	183	0	0	I	within gene(s) PMM1096;
1041770	+	TSS_008762	1000	712	0	0	Ai	antisense to gene(s) PMM1097;
1042334	-	TSS_022428	1000	986	0	0	P	14nt upstream of gene PMM1097;
1042406	+	TSS_008766	1000	2108	0	0	P	20nt upstream of gene PMM1098;
1042908	-	TSS_022432	1000	100	0	1	Ai	antisense to gene(s) PMM1098;
1056495	-	TSS_022440	1000	122	0	0	IP	within gene(s) PMM1107; 182nt upstream of gene PMM1106;
1057074	-	TSS_022450	1000	2491	0	1	P	35nt upstream of gene PMM1107;
1058522	+	TSS_008806	1000	1328	0	3	IP	within gene(s) PMM1110; 95nt upstream of gene PMM1111;
1059299	+	TSS_008813	1000	333	0	0	P	47nt upstream of gene PMM1113;
1059858	+	TSS_008821	1000	290	0	2	I	within gene(s) PMM1113;
1063713	+	TSS_005674	1000		0	1	P	21nt upstream of gene PMM1117;
1063850	+	TSS_008835	1000	131	0	4	O	-
1064477	+	TSS_008836	1000	164	0	0	P	24nt upstream of gene PMM1118;

1065756	-	TSS_022609	1000	6931	0	90	I	within gene(s) PMM1119;
1066209	-	TSS_022609	1000		0	0	P	9nt upstream of gene PMM1119;
1068972	-	TSS_022850	1000	6931	0	153	I	within gene(s) PMM1121;
1069447	-	TSS_022964	1000	3699	0	5	P	28nt upstream of gene PMM1121;
1070354	-	TSS_022966	1000	94	0	1	O	-
1070531	+	TSS_008912	1000	103	0	1	O	-
1071780	-	TSS_022997	1000	2501	0	0	I	within gene(s) PMM1123;
1072201	-	TSS_023072	1000	1342	0	18	I	within gene(s) PMM1123;
1072469	-	TSS_005754	1000		0	0	P	94nt upstream of gene PMM1123;
1073585	-	TSS_023108	1000	121	0	0	I	within gene(s) PMM1124;
1074287	-	TSS_023111	1000	884	0	1	I	within gene(s) PMM1124;
1078286	-	TSS_023121	1000	1100	0	4	I	within gene(s) PMM1127;
1079173	+	TSS_008943	1000	145	0	0	Ad	antisense to gene(s) PMM1128 (19nt downstream);
1079462	-	TSS_023130	1000	3850	0	4	P	17nt upstream of gene PMM1128;
1082063	-	TSS_023137	1000	3511	0	3	O	-
1082218	+	TSS_008954	1000	2814	0	4	P	51nt upstream of gene PMM1132;
1083365	-	TSS_023145	1000	180	0	1	Ai	antisense to gene(s) PMM1132;
1083716	+	TSS_008966	1000	181	0	0	I	within gene(s) PMM1132;
1084946	-	TSS_023152	1000	99	0	4	O	-
1085043	+	TSS_008970	1000	6952	0	3	P	19nt upstream of gene PMM1134;
1085641	+	TSS_008983	1000	8525	0	3	P	15nt upstream of gene PMM1135;
1087615	+	TSS_009016	1000	389	0	0	O	-
1089687	+	TSS_009019	1000	203	0	0	I	within gene(s) PMM1139;
1090775	+	TSS_009021	1000	879	0	0	I	within gene(s) PMM1140;
1095730	+	TSS_009029	1000	283	0	0	I	within gene(s) PMM1142;
1097928	-	TSS_023185	1000	135	0	12	I	within gene(s) PMM1145;
1098690	-	TSS_023221	1000	124	0	9	I	within gene(s) PMM1145;
1098755	-	TSS_023229	1000	199	0	3	I	within gene(s) PMM1145;
1099273	-	TSS_023246	1000	1018	0	6	IP	within gene(s) PMM1147; 96nt upstream of gene PMM1146;
1100340	-	TSS_023272	1000	1684	0	5	P	17nt upstream of gene PMM1147;
1100440	+	TSS_009046	1000	339	0	1	P	16nt upstream of gene PMM1148;
1101175	+	TSS_009100	1000	7298	0	2	P	15nt upstream of gene PMM1149;
1101469	+	TSS_005889	1000		0	0	P	8nt upstream of gene PMM1149;
1103158	-	TSS_023291	1000	1965	0	3	P	16nt upstream of gene PMM1151;
1103474	-	TSS_023296	1000	3469	0	3	O	-
1104586	-	TSS_023310	1000	821	0	12	IP	within gene(s) PMM1152a; 112nt upstream of gene PMM1152;
1105595	+	TSS_009199	1000	1047	0	5	IP	within gene(s) PMM1153; 99nt upstream of gene PMM1154;
1107430	-	TSS_023319	1000	760	0	3	P	2nt upstream of gene PMM1156;
1107587	+	TSS_023319	1000		0	0	P	26nt upstream of gene PMM1157;
1108327	+	TSS_009223	1000	609	0	6	I	within gene(s) PMM1157;
1108805	+	TSS_009254	1000	3163	0	8	I	within gene(s) PMM1158;
1115277	+	TSS_009375	1000	315	0	0	O	-
1115437	+	TSS_009377	1000	215	0	0	O	-
1118179	-	TSS_023397	1000	358	0	1	P	33nt upstream of gene PMM1165;
1120049	+	TSS_009386	1000	1949	0	1	P	15nt upstream of gene PMM1169;
1121159	+	TSS_009395	1000	145	0	0	Ai	antisense to gene(s) PMM1171;
1121569	-	TSS_023421	1000	4562	0	3	P	16nt upstream of gene PMM1171;
1122779	+	TSS_009398	1000	188	0	1	P	17nt upstream of gene PMM1174;
1122905	+	TSS_009400	1000	118	0	1	I	within gene(s) PMM1174;
1123430	-	TSS_023428	1000	408	0	6	Ai	antisense to gene(s) PMM1175;
1124119	+	TSS_009403	1000	95	0	0	I	within gene(s) PMM1176;
1126503	-	TSS_023437	1000	116	0	2	I	within gene(s) PMM1178;
1127018	+	TSS_009408	1000	1094	0	1	Ai	antisense to gene(s) PMM1178;
1127288	-	TSS_023442	1000	138	0	1	P	26nt upstream of gene PMM1178;
1127626	+	TSS_009414	1000	1748	0	2	Ai	antisense to gene(s) PMM1179;
1128259	+	TSS_009419	1000	767	0	1	P	28nt upstream of gene PMM1180;
1128570	-	TSS_023449	1000	628	0	0	Ai	antisense to gene(s) PMM1180;
1130146	+	TSS_009470	1000	3194	0	4	P	13nt upstream of gene PMM1183;
1130398	-	TSS_023454	1000	294	0	0	Ai	antisense to gene(s) PMM1184;
1130649	+	TSS_009475	1000	507	0	0	IP	within gene(s) PMM1184; 44nt upstream of gene PMM1185;
1131150	+	TSS_009479	1000	4125	0	3	P	29nt upstream of gene PMM1186;
1131452	+	TSS_009499	1000	195	0	18	I	within gene(s) PMM1186;
1131719	+	TSS_009506	1000	756	0	0	I	within gene(s) PMM1186;
1131989	+	TSS_009521	1000	204	0	4	I	within gene(s) PMM1186;
1133995	+	TSS_009531	1000	401	0	0	I	within gene(s) PMM1188;
1136337	+	TSS_009539	1000	686	0	6	P	22nt upstream of gene PMM1190;
1136852	+	TSS_009543	1000	2137	0	1	P	0nt upstream of gene PMM1191;
1137382	-	TSS_023471	1000	377	0	0	Ai	antisense to gene(s) PMM1191;
1138412	+	TSS_009588	1000	149	0	1	I	within gene(s) PMM1191;
1138845	-	TSS_023478	1000	155	0	1	Ai	antisense to gene(s) PMM1191;
1139305	-	TSS_023485	1000	114	0	0	I	within gene(s) PMM1192;
1139830	+	TSS_009604	1000	186	0	0	PAi	95nt upstream of gene PMM1193; antisense to gene(s) PMM1192;
1142062	+	TSS_009608	1000	126	0	9	I	within gene(s) PMM1196;
1150601	-	TSS_023495	1000	147	0	0	I	within gene(s) PMM1202;
1152750	-	TSS_023501	1000	292	0	2	I	within gene(s) PMM1204;
1152998	-	TSS_023503	1000	126	0	0	IP	within gene(s) PMM1205; 88nt upstream of gene PMM1204;
1153530	-	TSS_023505	1000	91	0	0	I	within gene(s) PMM1205;
1168566	-	TSS_023529	1000	98	0	6	O	-
1175722	+	TSS_009671	1000	117	0	0	I	within gene(s) PMM1225;
1181446	-	TSS_023536	1000	167	0	2	Ai	antisense to gene(s) PMM1229;

1182738	-	TSS_023539	1000	101	0	0 Ai	antisense to gene(s) PMM1230;
1183984	+	TSS_009678	1000	433	0	6 I	within gene(s) PMM1232;
1186173	+	TSS_009681	1000	735	0	2 I	within gene(s) PMM1234;
1186359	-	TSS_023543	1000	483	0	1 Ai	antisense to gene(s) PMM1234;
1186823	+	TSS_009688	1000	2510	0	2 I	within gene(s) PMM1234;
1187310	+	TSS_009692	1000	125	0	0 I	within gene(s) PMM1235;
1192406	+	TSS_009703	1000	113	0	3 IP	within gene(s) PMM1239; 15nt upstream of gene PMM1240;
1201748	-	TSS_023557	1000	140	0	2 I	within gene(s) PMM1249;
1202454	-	TSS_023563	1000	144	0	1 I	within gene(s) PMM1250;
1203802	+	TSS_009715	1000	432	0	0 Ai	antisense to gene(s) PMM1251;
1209541	-	TSS_023582	1000	1404	0	1 I	within gene(s) PMM1256;
1211786	+	TSS_009730	1000	451	0	5 I	within gene(s) PMM1258;
1212532	+	TSS_009734	1000	244	0	0 IP	within gene(s) PMM1258; 148nt upstream of gene PMM1259;
1212906	+	TSS_009735	1000	604	0	0 I	within gene(s) PMM1259;
1213317	+	TSS_009742	1000	1295	0	4 I	within gene(s) PMM1259;
1214272	+	TSS_009745	1000	223	0	0 I	within gene(s) PMM1260;
1214724	+	TSS_009748	1000	672	0	0 IP	within gene(s) PMM1260; 140nt upstream of gene PMM1261;
1216927	-	TSS_023617	1000	17065	0	1 P	26nt upstream of gene PMM1262;
1218465	-	TSS_023642	1000	331	0	27 I	within gene(s) PMM1264;
1218623	+	TSS_009773	1000	456	0	1 Ai	antisense to gene(s) PMM1264;
1219792	-	TSS_023740	1000	1706	0	10 P	15nt upstream of gene PMM1264;
1219908	-	TSS_023746	1000	174	0	0 IP	within gene(s) PMM1265; 131nt upstream of gene PMM1264;
1220160	+	TSS_009783	1000	126	0	5 Ai	antisense to gene(s) PMM1265;
1223096	+	TSS_009793	1000	116	0	0 Ai	antisense to gene(s) PMM1269;
1223575	-	TSS_023765	1000	139	0	3 I	within gene(s) PMM1269;
1224017	-	TSS_023771	1000	3919	0	10 P	16nt upstream of gene PMM1269;
1224107	+	TSS_009796	1000	445	0	2 Ai	antisense to gene(s) PMM1270;
1224306	-	TSS_023777	1000	232	0	3 I	within gene(s) PMM1270;
1224483	+	TSS_009801	1000	1218	0	1 Ai	antisense to gene(s) PMM1270;
1225100	-	TSS_023803	1000	548	0	2 P	20nt upstream of gene PMM1270;
1226399	+	TSS_009808	1000	1924	0	2 PAi	139nt upstream of gene PMM1273; antisense to gene(s) PMM1272;
1226493	-	TSS_023810	1000	8758	0	2 P	23nt upstream of gene PMM1272;
1228488	-	TSS_023814	1000	106	0	0 Ai	antisense to gene(s) PMM1274;
1228824	+	TSS_009814	1000	158	0	0 P	13nt upstream of gene PMM1276;
1229056	+	TSS_009815	1000	182	0	0 P	15nt upstream of gene PMM1277;
1229316	+	TSS_009817	1000	222	0	0 Ai	antisense to gene(s) PMM1278;
1229883	-	TSS_023821	1000	161	0	1 IP	within gene(s) PMM1279; 18nt upstream of gene PMM1278;
1230756	-	TSS_023824	1000	174	0	0 I	within gene(s) PMM1280;
1231722	-	TSS_023830	1000	1404	0	1 IP	within gene(s) PMM1281; 194nt upstream of gene PMM1280;
1233089	+	TSS_009829	1000	2384	0	1 Ai	antisense to gene(s) PMM1283;
1233817	-	TSS_023952	1000	47287	0	8 P	17nt upstream of gene PMM1283;
1235226	-	TSS_023964	1000	957	0	3 P	16nt upstream of gene PMM1285;
1235758	+	TSS_009864	1000	174	0	0 Ai	antisense to gene(s) PMM1286;
1236777	-	TSS_023996	1000	3936	0	7 P	16nt upstream of gene PMM1286;
1238666	-	TSS_024013	1000	115	0	0 I	within gene(s) PMM1287;
1238943	-	TSS_024014	1000	1584	0	2 P	24nt upstream of gene PMM1287;
1239042	+	TSS_009875	1000	4547	0	4 P	27nt upstream of gene PMM1288;
1239358	-	TSS_024017	1000	129	0	1 Ai	antisense to gene(s) PMM1288;
1239822	+	TSS_009912	1000	177	0	24 I	within gene(s) PMM1288;
1239949	-	TSS_024024	1000	455	0	0 Ai	antisense to gene(s) PMM1288;
1240190	+	TSS_009919	1000	257	0	2 Ai	antisense to gene(s) PMM1289;
1240490	-	TSS_024057	1000	816	0	9 I	within gene(s) PMM1289;
1241067	-	TSS_024102	1000	11109	0	2 P	18nt upstream of gene PMM1289;
1241474	-	TSS_024109	1000	144	0	2 I	within gene(s) PMM1290;
1242168	+	TSS_009940	1000	109	0	0 Ai	antisense to gene(s) PMM1290;
1242445	+	TSS_009941	1000	290	0	0 Ai	antisense to gene(s) PMM1290;
1243570	-	TSS_024120	1000	129	0	0 Ai	antisense to gene(s) PMM1291;
1244009	+	TSS_009957	1000	680	0	1 Ai	antisense to gene(s) PMM1292;
1245509	+	TSS_009960	1000	1458	0	0 P	17nt upstream of gene PMM1293;
1245700	+	TSS_009975	1000	749	0	0 I	within gene(s) PMM1293;
1246156	+	TSS_009993	1000		0	0 I	17nt upstream of gene PMM1294;
1246482	+	TSS_010031	1000	131	0	3 IP	within gene(s) PMM1294; 248nt upstream of gene PMM1295;
1247681	-	TSS_024137	1000	150	0	0 P	20nt upstream of gene PMM1296;
1250117	+	TSS_010040	1000	161	0	0 Ai	antisense to gene(s) PMM1299;
1250468	-	TSS_024147	1000	401	0	4 I	within gene(s) PMM1299;
1251088	+	TSS_010042	1000	380	0	0 P	15nt upstream of gene PMM1300;
1252379	+	TSS_010051	1000	100	0	0 I	within gene(s) PMM1300;
1252727	-	TSS_024172	1000	5644	0	5 O	-
1252970	+	TSS_010065	1000	827	0	1 P	32nt upstream of gene PMM1301;
1253102	+	TSS_010066	1000	251	0	0 I	within gene(s) PMM1301;
1256224	+	TSS_010078	1000	152	0	1 Ai	antisense to gene(s) PMM1303;
1258142	-	TSS_024189	1000	195	0	12 P	16nt upstream of gene PMM1304;
1258258	+	TSS_010083	1000	560	0	6 P	151nt upstream of gene PMM1305;
1259122	-	TSS_024197	1000	254	0	1 Ai	antisense to gene(s) PMM1305;
1260859	+	TSS_010105	1000		0	0 IP	within gene(s) PMM1306; 33nt upstream of gene PMM1307;
1262176	+	TSS_010112	1000	1504	0	2 P	26nt upstream of gene PMM1309;
1263425	-	TSS_024217	1000	567	0	1 O	-
1263674	+	TSS_010205	1000	2229	0	2 I	within gene(s) PMM1310;
1265561	+	TSS_010215	1000	25275	0	4 I	within gene(s) PMM1312;
1266213	-	TSS_024227	1000	308	0	1 Ai	antisense to gene(s) PMM1312;

1266690	+	TSS_010272	1000	25778	0	4	P	20nt upstream of gene PMM1313;
1268104	+	TSS_010459	1000	1220	0	1	P	24nt upstream of gene PMM1315;
1270161	+	TSS_010510	1000	153	0	4	P	15nt upstream of gene PMM1317;
1272067	-	TSS_017393	1000	159813	0	8	P	65nt upstream of gene PMM1321;
1273518	-	TSS_024265	1000	283	0	1	Ai	antisense to gene(s) PMM1322;
1275301	-	TSS_024272	1000	212	0	0	Ai	antisense to gene(s) PMM1323;
1275591	+	TSS_010536	1000	220	0	0	I	within gene(s) PMM1323;
1276011	-	TSS_024274	1000	685	0	3	Ai	antisense to gene(s) PMM1323;
1276210	+	TSS_010540	1000	466	0	0	I	within gene(s) PMM1324;
1276620	-	TSS_024279	1000	306	0	0	Ai	antisense to gene(s) PMM1324;
1276641	+	TSS_010546	1000	493	0	0	I	within gene(s) PMM1324;
1278318	+	TSS_010553	1000	437	0	0	IP	within gene(s) PMM1326; 106nt upstream of gene PMM1327;
1281106	-	TSS_024296	1000	750	0	1	IP	within gene(s) PMM1330; 132nt upstream of gene PMM1329;
1281349	-	TSS_024299	1000	99	0	1	P	30nt upstream of gene PMM1330;
1284193	-	TSS_024308	1000	147	0	0	I	within gene(s) PMM1333;
1285667	-	TSS_024311	1000	163	0	3	I	within gene(s) PMM1335;
1285747	-	TSS_024313	1000	248	0	0	I	within gene(s) PMM1335;
1286762	+	TSS_010588	1000	170	0	1	Ai	antisense to gene(s) PMM1337;
1287887	-	TSS_024329	1000	468	0	1	I	within gene(s) PMM1338;
1287977	-	TSS_024330	1000	190	0	0	I	within gene(s) PMM1338;
1288162	+	TSS_010595	1000	171	0	0	Ai	antisense to gene(s) PMM1338;
1288975	-	TSS_024341	1000	158	0	0	I	within gene(s) PMM1338;
1289984	-	TSS_024349	1000	118	0	0	I	within gene(s) PMM1339;
1291318	-	TSS_024357	1000	177	0	0	Ai	antisense to gene(s) PMM1340;
1291478	+	TSS_010606	1000	1516	0	2	I	within gene(s) PMM1340;
1291709	-	TSS_024359	1000	113	0	0	Ai	antisense to gene(s) PMM1340;
1293438	+	TSS_010632	1000	173	0	7	I	within gene(s) PMM1341;
1293604	+	TSS_010636	1000	130	0	0	I	within gene(s) PMM1341;
1293828	+	TSS_010638	1000	137	0	2	I	within gene(s) PMM1341;
1294828	-	TSS_024392	1000	176	0	6	I	within gene(s) PMM1342;
1295467	-	TSS_024423	1000	145	0	6	I	within gene(s) PMM1342;
1295547	-	TSS_024426	1000	203	0	6	P	38nt upstream of gene PMM1342;
1295966	+	TSS_010643	1000	258	0	2	P	75nt upstream of gene PMM1344;
1296395	+	TSS_010652	1000	620	0	6	IP	within gene(s) PMM1344; 96nt upstream of gene PMM1345;
1299151	+	TSS_010661	1000	568	0	0	Ai	antisense to gene(s) PMM1348;
1299740	+	TSS_010662	1000	735	0	0	P	25nt upstream of gene PMM1349;
1300005	+	TSS_010667	1000	211	0	5	I	within gene(s) PMM1349;
1300764	+	TSS_010676	1000	612	0	0	I	within gene(s) PMM1350;
1302130	-	TSS_024482	1000	19481	0	2	P	33nt upstream of gene PMM1352;
1304765	-	TSS_024542	1000	554	0	0	P	19nt upstream of gene PMM1354;
1307622	+	TSS_010718	1000	428	0	2	O	-
1309146	-	TSS_024552	1000	164	0	0	I	within gene(s) PMM1360;
1309784	+	TSS_010720	1000	91	0	2	P	56nt upstream of gene PMM1361;
1313108	+	TSS_010730	1000	647	0	2	P	16nt upstream of gene PMM1365;
1315214	+	TSS_010748	1000	95	0	2	Ai	antisense to gene(s) PMM1368;
1315509	-	TSS_024565	1000	1055	0	7	P	15nt upstream of gene PMM1368;
1315687	-	TSS_024573	1000	107	0	0	IP	within gene(s) PMM1369; 193nt upstream of gene PMM1368;
1318282	-	TSS_024583	1000	504	0	3	O	-
1319591	+	TSS_010759	1000	2002	0	2	O	-
1319966	+	TSS_010766	1000	689	0	2	P	13nt upstream of gene PMM1372;
1321010	+	TSS_010771	1000	6369	0	2	O	-
1322462	-	TSS_024590	1000	261	0	0	O	-
1323807	+	TSS_010782	1000	134	0	6	P	34nt upstream of gene PMM1376;
1324248	+	TSS_010788	1000	301	0	0	P	19nt upstream of gene PMM1377;
1324447	+	TSS_010789	1000	22	9.4E-14	3	I	within gene(s) PMM1377;
1326668	-	TSS_024599	1000	1198	0	1	O	-
1328956	-	TSS_024603	1000	491	0	0	O	-
1330723	-	TSS_024606	1000	189	0	0	Ai	antisense to gene(s) PMM1381;
1332432	+	TSS_010813	1000	250	0	0	O	-
1332932	-	TSS_024612	1000	135	0	2	I	within gene(s) PMM1383;
1333562	-	TSS_024617	1000	147	0	2	P	188nt upstream of gene PMM1383;
1334143	+	TSS_010816	1000	644	0	0	O	-
1334592	+	TSS_007415	1000		0	5	P	21nt upstream of gene PMM1384;
1335359	+	TSS_010827	1000	1705	0	1	O	-
1336436	+	TSS_010831	1000	883	0	2	O	-
1338319	-	TSS_024627	1000	122	0	5	Ai	antisense to gene(s) PMM1386;
1338981	+	TSS_010838	1000	252	0	2	P	17nt upstream of gene PMM1387;
1339834	-	TSS_024631	1000	509	0	1	P	32nt upstream of gene PMM1388;
1341205	-	TSS_024640	1000	476	0	1	P	20nt upstream of gene PMM1390;
1341449	+	TSS_010843	1000	237	0	0	P	15nt upstream of gene PMM1391;
1343374	+	TSS_010846	1000	169	0	0	I	within gene(s) PMM1394;
1343856	-	TSS_024644	1000	1921	0	7	O	-
1344024	-	TSS_024649	1000	426	0	3	O	-
1345064	-	TSS_024667	1000	771	0	2	I	within gene(s) PMM1396;
1345256	-	TSS_024696	1000	1262	0	10	IP	within gene(s) PMM1397; 189nt upstream of gene PMM1396;
1345690	-	TSS_024748	1000	57347	0	5	P	16nt upstream of gene PMM1399;
1346865	-	TSS_024786	1000	13348	0	3	P	239nt upstream of gene PMM1400;
1346985	+	TSS_010866	1000	271	0	0	P	146nt upstream of gene PMM1401;
1347056	+	TSS_010868	1000	281	0	2	P	75nt upstream of gene PMM1401;
1347115	+	TSS_010871	1000	1475	0	2	P	16nt upstream of gene PMM1401;

1350449	-	TSS_024796	1000	517	0	3 P	81nt upstream of gene PMM1404;
1351013	-	TSS_024800	1000	979	0	3 P	26nt upstream of gene PMM1405;
1352055	-	TSS_024805	1000	196	0	0 P	24nt upstream of gene PMM1408;
1352429	-	TSS_024811	1000	3746	0	3 P	18nt upstream of gene PMM1409;
1353625	-	TSS_024830	1000	480	0	10 O	-
1353716	-	TSS_024834	1000	1700	0	1 O	-
1355165	+	TSS_010926	1000	1806	0	3 P	17nt upstream of gene PMM1413;
1355538	-	TSS_024841	1000	203	0	0 O	-
1355578	+	TSS_010932	1000	308	0	6 P	14nt upstream of gene PMM1414;
1355765	+	TSS_010935	1000	94	0	1 IP	within gene(s) PMM1414; 230nt upstream of gene PMM1415;
1356437	-	TSS_024843	1000	252	0	0 Ai	antisense to gene(s) PMM1415;
1356695	+	TSS_010938	1000	150	0	1 I	within gene(s) PMM1415;
1358558	+	TSS_010961	1000	139	0	0 I	within gene(s) PMM1416;
1358652	+	TSS_010966	1000	1078	0	1 I	within gene(s) PMM1416;
1358898	-	TSS_024858	1000	399	0	0 Ai	antisense to gene(s) PMM1416;
1359810	-	TSS_024867	1000	143	0	6 Ai	antisense to gene(s) PMM1416;
1360185	-	TSS_024876	1000	2438	0	2 O	-
1362875	+	TSS_010989	1000	417	0	0 Ai	antisense to gene(s) PMM1421 PMM1422;
1363264	-	TSS_024893	1000	180	0	15 I	within gene(s) PMM1422;
1363417	-	TSS_024895	1000	229	0	0 P	27nt upstream of gene PMM1422;
1364352	-	TSS_024900	1000	1435	0	0 P	202nt upstream of gene PMM1424;
1364483	+	TSS_010993	1000	103	0	0 O	-
1366152	-	TSS_024904	1000	196	0	2 Ai	antisense to gene(s) PMM1425;
1366222	-	TSS_024905	1000	181	0	0 Ai	antisense to gene(s) PMM1425;
1367108	+	TSS_011000	1000		0	0 P	18nt upstream of gene PMM1427;
1367587	+	TSS_011006	1000	363	0	2 P	92nt upstream of gene PMM1428;
1367686	+	TSS_011008	1000	207	0	0 I	within gene(s) PMM1428;
1369350	-	TSS_024909	1000	91	0	2 P	19nt upstream of gene PMM1431;
1369959	+	TSS_011016	1000	410	0	1 I	within gene(s) PMM1432;
1370154	-	TSS_024914	1000	124	0	0 Ai	antisense to gene(s) PMM1432;
1371646	+	TSS_007561	1000	854	0	4 P	31nt upstream of gene PMM1434;
1371875	+	TSS_011034	1000	251	0	24 I	within gene(s) PMM1434;
1372149	-	TSS_024925	1000	2093	0	2 Ai	antisense to gene(s) PMM1434;
1373308	+	TSS_007573	1000	3164	0	4 P	17nt upstream of gene PMM1435;
1374729	+	TSS_011085	1000	592	0	2 Ai	antisense to gene(s) PMM1436;
1374822	+	TSS_011088	1000	614	0	4 Ai	antisense to gene(s) PMM1436;
1375708	-	TSS_025122	1000		0	4 P	71nt upstream of gene PMM1437;
1375811	+	TSS_011104	1000	261	0	3 P	47nt upstream of gene PMM1438;
1378269	-	TSS_025142	1000	847	0	3 P	18nt upstream of gene PMM1440;
1379477	+	TSS_011126	1000	3808	0	7 Ai	antisense to gene(s) PMM1442;
1379556	+	TSS_011129	1000	1065	0	0 Ai	antisense to gene(s) PMM1442;
1379949	+	TSS_011131	1000	272	0	0 P	27nt upstream of gene PMM1443;
1380027	+	TSS_011134	1000	605	0	0 I	within gene(s) PMM1443;
1380210	+	TSS_011135	1000	97	0	3 I	within gene(s) PMM1443;
1380876	+	TSS_011155	1000	2412	0	2 IP	within gene(s) PMM1443; 80nt upstream of gene PMM1444;
1382749	+	TSS_011168	1000	113	0	1 IP	within gene(s) PMM1445; 31nt upstream of gene PMM1446;
1385792	+	TSS_011176	1000	247	0	3 Ai	antisense to gene(s) PMM1449;
1386056	-	TSS_025179	1000	177	0	1 I	within gene(s) PMM1449;
1386700	+	TSS_011180	1000	616	0	0 Ai	antisense to gene(s) PMM1450;
1387066	+	TSS_011182	1000	216	0	1 Ai	antisense to gene(s) PMM1451;
1388669	-	TSS_025213	1000	164	0	0 IP	within gene(s) PMM1452; 88nt upstream of gene PMM1451;
1389346	-	TSS_025215	1000	114	0	3 IP	within gene(s) PMM1453; 191nt upstream of gene PMM1452;
1390543	-	TSS_018403	1000		0	0 P	104nt upstream of gene PMM1455;
1392965	-	TSS_025226	1000	175	0	0 Ai	antisense to gene(s) PMM1458;
1393186	+	TSS_011202	1000	321	0	0 I	within gene(s) PMM1458;
1393368	+	TSS_011204	1000	1495	0	1 P	69nt upstream of gene PMM1459;
1395953	+	TSS_011213	1000	718	0	5 P	14nt upstream of gene PMM1462;
1396223	+	TSS_011223	1000	4100	0	4 P	14nt upstream of gene PMM1463;
1397666	+	TSS_011241	1000	5215	0	2 P	16nt upstream of gene PMM1465;
1398489	+	TSS_011316	1000	435	0	78 I	within gene(s) PMM1465;
1401712	-	TSS_025249	1000	184	0	0 I	within gene(s) PMM1467;
1403187	-	TSS_025257	1000	1477	0	1 P	46nt upstream of gene PMM1467;
1410409	-	TSS_025271	1000	220	0	1 I	within gene(s) PMM1476;
1411585	-	TSS_025306	1000	6030	0	10 P	26nt upstream of gene PMM1479;
1412048	+	TSS_011368	1000	265	0	0 Ai	antisense to gene(s) PMM1480;
1412609	-	TSS_025312	1000	364	0	0 P	161nt upstream of gene PMM1480;
1413912	+	TSS_011371	1000	1221	0	6 O	-
1414968	-	TSS_025357	1000	323	0	10 I	within gene(s) PMM1483;
1415400	-	TSS_025394	1000	371	0	12 I	within gene(s) PMM1483;
1416227	-	TSS_025447	1000	511	0	3 I	within gene(s) PMM1483;
1416999	-	TSS_025496	1000	1049	0	16 I	within gene(s) PMM1483;
1417887	-	TSS_025560	1000	200	0	9 I	within gene(s) PMM1483;
1418150	+	TSS_011401	1000	685	0	1 Ai	antisense to gene(s) PMM1484;
1418507	+	TSS_011408	1000	2551	0	1 Ai	antisense to gene(s) PMM1484;
1419267	-	TSS_025636	1000	403	0	9 I	within gene(s) PMM1484;
1420706	-	TSS_025705	1000	854	0	1 I	within gene(s) PMM1485;
1420978	-	TSS_025720	1000	339	0	0 I	within gene(s) PMM1485;
1421293	-	TSS_025730	1000	190	0	1 I	within gene(s) PMM1485;
1422856	-	TSS_025770	1000	978	0	0 I	within gene(s) PMM1485;
1423046	-	TSS_025773	1000	283	0	0 I	within gene(s) PMM1485;

1423524 -	TSS_025776	1000	276	0	4	P	211nt upstream of gene PMM1485;
1423961 +	TSS_011452	1000	556	0	1	Ai	antisense to gene(s) PMM1486;
1424666 -	TSS_025783	1000	134	0	2	P	20nt upstream of gene PMM1487;
1427278 +	TSS_011466	1000	477	0	2	Ai	antisense to gene(s) PMM1490;
1428507 +	TSS_011471	1000	341	0	0	IP	within gene(s) PMM1491; 98nt upstream of gene PMM1492;
1429727 +	TSS_011531	1000	488	0	3	I	within gene(s) PMM1492;
1431135 +	TSS_011558	1000	622	0	7	I	within gene(s) PMM1494;
1431241 +	TSS_011563	1000	303	0	6	I	within gene(s) PMM1494;
1431469 +	TSS_011575	1000	265	0	3	I	within gene(s) PMM1494;
1433023 +	TSS_011635	1000	711	0	7	I	within gene(s) PMM1494;
1433296 +	TSS_011641	1000	486	0	0	I	within gene(s) PMM1494;
1435445 +	TSS_011653	1000	156	0	0	I	within gene(s) PMM1496;
1436999 +	TSS_011655	1000	753	0	0	P	24nt upstream of gene PMM1498;
1437357 -	TSS_025849	1000	510	0	1	Ai	antisense to gene(s) PMM1498;
1439023 -	TSS_025856	1000	985	0	0	I	within gene(s) PMM1499;
1439138 +	TSS_011706	1000	237	0	1	Ai	antisense to gene(s) PMM1500;
1439422 -	TSS_025879	1000	354	0	0	I	within gene(s) PMM1500;
1440154 -	TSS_025922	1000	169	0	1	I	within gene(s) PMM1500;
1440282 -	TSS_025928	1000	1598	0	2	P	23nt upstream of gene PMM1500;
1440561 +	TSS_011717	1000	112	0	0	I	within gene(s) PMM1501;
1441843 +	TSS_011722	1000	1512	0	3	I	within gene(s) PMM1501;
1443305 +	TSS_011729	1000	644	0	4	PAi	201nt upstream of gene PMM1504; antisense to gene(s) PMM1503;
1446946 +	TSS_011755	1000	773	0	8	Ai	antisense to gene(s) PMM1508;
1447690 -	TSS_026094	1000	1177	0	4	IP	within gene(s) PMM1509; 61nt upstream of gene PMM1508;
1447936 -	TSS_026113	1000	4556	0	7	I	within gene(s) PMM1509;
1448025 -	TSS_026120	1000	789	0	7	I	within gene(s) PMM1509;
1448485 -	TSS_026157	1000	1112	0	45	I	within gene(s) PMM1509;
1449812 -	TSS_026256	1000	1581	0	5	P	64nt upstream of gene PMM1509;
1450432 -	TSS_026274	1000	604	0	5	IP	within gene(s) PMM1511; 109nt upstream of gene PMM1510;
1450752 -	TSS_026278	1000	187	0	5	P	25nt upstream of gene PMM1511;
1450891 +	TSS_011781	1000	119	0	5	P	31nt upstream of gene PMM1512;
1454166 +	TSS_011834	1000	273	0	2	I	within gene(s) PMM1512;
1454543 +	TSS_011846	1000	232	0	0	I	within gene(s) PMM1512;
1456713 -	TSS_019390	1000	5241	0	4	P	20nt upstream of gene PMM1514;
1459395 -	TSS_026307	1000	187	0	0	Ai	antisense to gene(s) PMM1518;
1460021 -	TSS_026313	1000	458	0	1	I	within gene(s) PMM1519;
1460363 -	TSS_026317	1000	1678	0	3	P	15nt upstream of gene PMM1520;
1462416 -	TSS_026376	1000	1224	0	39	I	within gene(s) PMM1523;
1464296 -	TSS_026610	1000	388	0	14	IP	within gene(s) PMM1524; 163nt upstream of gene PMM1523;
1465661 -	TSS_019863	1000	2937	0	33	I	within gene(s) PMM1524;
1466574 -	TSS_026889	1000	40164	0	2	P	109nt upstream of gene PMM1524;
1468533 -	TSS_026896	1000	176	0	0	Ai	antisense to gene(s) PMM1525;
1468704 +	TSS_011939	1000	134	0	7	P	21nt upstream of gene PMM1526;
1469172 +	TSS_011941	1000	346	0	0	I	within gene(s) PMM1526;
1469928 -	TSS_026900	1000	158	0	0	I	within gene(s) PMM1527;
1470954 +	TSS_011946	1000	518	0	2	P	15nt upstream of gene PMM1528;
1471756 +	TSS_011950	1000	232	0	0	Ai	antisense to gene(s) PMM1529;
1473040 -	TSS_026921	1000	165	0	7	IP	within gene(s) PMM1531; 169nt upstream of gene PMM1530;
1473409 -	TSS_019991	1000	124	0	6	IP	within gene(s) PMM1532; 114nt upstream of gene PMM1531;
1473764 -	TSS_026938	1000	521	0	4	P	28nt upstream of gene PMM1532;
1474126 -	TSS_026941	1000	284	0	1	I	within gene(s) PMM1533;
1475305 -	TSS_026961	1000	501	0	0	I	within gene(s) PMM1535;
1475507 -	TSS_026966	1000	256	0	0	I	within gene(s) PMM1535;
1477037 -	TSS_027000	1000	1892	0	2	P	21nt upstream of gene PMM1538;
1479684 +	TSS_011971	1000	975	0	0	Ai	antisense to gene(s) PMM1542;
1480004 -	TSS_027028	1000	736	0	19	I	within gene(s) PMM1542;
1480604 -	TSS_027069	1000	1050	0	10	IP	within gene(s) PMM1544; 178nt upstream of gene PMM1543;
1482034 -	TSS_027146	1000	159	0	0	IP	within gene(s) PMM1547; 93nt upstream of gene PMM1546;
1484971 -	TSS_027231	1000	217	0	2	IP	within gene(s) PMM1554; 134nt upstream of gene PMM1553;
1485370 -	TSS_027241	1000	354	0	0	I	within gene(s) PMM1555;
1486430 -	TSS_027269	1000	141	0	0	IP	within gene(s) PMM1557; 94nt upstream of gene PMM1556;
1487844 -	TSS_027287	1000	97	0	1	P	229nt upstream of gene PMM1558;
1487895 +	TSS_012035	1000	348	0	6	P	13nt upstream of gene PMM1559;
1490373 +	TSS_012048	1000	7393	0	2	P	85nt upstream of gene PMM1562;
1490794 +	TSS_012124	1000	1906	0	68	I	within gene(s) PMM1562;
1491478 +	TSS_012250	1000	931	0	69	I	within gene(s) PMM1562;
1491832 -	TSS_027323	1000	114	0	2	P	14nt upstream of gene PMM1563;
1494808 -	TSS_027330	1000	123	0	3	I	within gene(s) PMM1566;
1495329 +	TSS_012277	1000	2329	0	5	Ai	antisense to gene(s) PMM1566;
1495758 -	TSS_027334	1000	695	0	2	PAi	19nt upstream of gene PMM1566; antisense to gene(s) PMM1567;
1496620 +	TSS_012281	1000	954	0	0	IAd	within gene(s) PMM1567; antisense to gene(s) PMM1568 (11nt downstream);
1497016 -	TSS_027352	1000	6133	0	9	P	21nt upstream of gene PMM1568;
1497103 -	TSS_027356	1000	168	0	2	P	108nt upstream of gene PMM1568;
1499500 -	TSS_027366	1000	134	0	2	I	within gene(s) PMM1570;
1499639 -	TSS_027371	1000	966	0	2	P	33nt upstream of gene PMM1570;
1499646 +	TSS_012298	1000	488	0	14	P	71nt upstream of gene PMM1571;
1501198 +	TSS_012307	1000	127	0	0	Ai	antisense to gene(s) PMM1573;
1502320 -	TSS_027377	1000	91	0	0	I	within gene(s) PMM1574;
1503685 -	TSS_027398	1000	155	0	3	I	within gene(s) PMM1575;
1504039 -	TSS_027414	1000	140	0	3	I	within gene(s) PMM1575;

1505392	-	TSS_027438	1000	107	0	1 I	within gene(s) PMM1575;
1505706	-	TSS_027443	1000	519	0	0 I	within gene(s) PMM1575;
1507214	-	TSS_027453	1000	97	0	11 I	within gene(s) PMM1577;
1508411	-	TSS_027459	1000	790	0	2 P	11nt upstream of gene PMM1577;
1508903	-	TSS_027468	1000	7339	0	6 P	21nt upstream of gene PMM1578;
1511027	+	TSS_012327	1000	710	0	0 Ai	antisense to gene(s) PMM1580;
1511176	+	TSS_012328	1000	120	0	0 Ai	antisense to gene(s) PMM1580;
1512795	-	TSS_027487	1000	451	0	2 P	92nt upstream of gene PMM1581;
1513707	-	TSS_027493	1000	898	0	1 Ai	antisense to gene(s) PMM1582;
1515544	-	TSS_027497	1000	331	0	0 I	within gene(s) PMM1585;
1518093	-	TSS_020891	1000		0	3 I	51nt upstream of gene PMM1588;
1518815	-	TSS_027523	1000	346	0	7 I	within gene(s) PMM1589;
1519244	-	TSS_020908	1000	1735	0	1 P	24nt upstream of gene PMM1589;
1523398	-	TSS_027547	1000	145	0	0 I	within gene(s) PMM1594;
1523570	-	TSS_027549	1000	1173	0	0 P	23nt upstream of gene PMM1594;
1523718	-	TSS_027550	1000	133	0	0 IP	within gene(s) PMM1595; 171nt upstream of gene PMM1594;
1524053	-	TSS_027551	1000	128	0	1 I	within gene(s) PMM1595;
1525296	+	TSS_012386	1000	198	0	12 I	within gene(s) PMM1596;
1528833	-	TSS_027576	1000	241	0	2 P	16nt upstream of gene PMM1599;
1530109	+	TSS_012411	1000	684	0	0 Ai	antisense to gene(s) PMM1600;
1530254	-	TSS_027601	1000	847	0	0 P	27nt upstream of gene PMM1600;
1530378	+	TSS_012416	1000	23475	0	3 P	3nt upstream of gene PMM1601;
1533752	-	TSS_027634	1000	24554	0	8 P	14nt upstream of gene PMM1602;
1533931	+	TSS_021008	1000		0	0 O	-
1533945	+	TSS_012693	1000	14	0	43 O	-
1535304	+	TSS_012844	1000	210	0	3 I	within gene(s) PMM1605;
1535492	-	TSS_027654	1000	217	0	0 PAi	224nt upstream of gene PMM1604; antisense to gene(s) PMM1605;
1536359	-	TSS_027660	1000	132	0	0 I	within gene(s) PMM1606;
1537533	-	TSS_027672	1000	819	0	2 I	within gene(s) PMM1606;
1538095	-	TSS_027681	1000	3070	0	9 P	22nt upstream of gene PMM1607;
1538185	+	TSS_012854	1000	1197	0	2 P	23nt upstream of gene PMM1608;
1539568	+	TSS_012862	1000	194	0	2 IP	within gene(s) PMM1609; 176nt upstream of gene PMM1610;
1539778	-	TSS_027692	1000	3006	0	4 Ai	antisense to gene(s) PMM1610;
1543254	-	TSS_027897	1000	2750	0	3 P	126nt upstream of gene PMM1611;
1543786	+	TSS_012913	1000	315	0	1 Ai	antisense to gene(s) PMM1613;
1546552	-	TSS_027918	1000	851	0	1 I	within gene(s) PMM1615;
1547526	+	TSS_012928	1000	277	0	0 Ai	antisense to gene(s) PMM1618;
1549235	-	TSS_027936	1000	1487	0	0 I	within gene(s) PMM1619;
1549500	-	TSS_027954	1000	196	0	9 I	within gene(s) PMM1619;
1549798	-	TSS_027963	1000	5956	0	8 P	58nt upstream of gene PMM1619;
1550719	-	TSS_027968	1000	92	0	0 I	within gene(s) PMM1621;
1551244	+	TSS_012941	1000	256	0	2 Ai	antisense to gene(s) PMM1622;
1551449	-	TSS_027979	1000	263	0	1 IP	within gene(s) PMM1622; 247nt upstream of gene PMM1621;
1552273	-	TSS_028021	1000	2863	0	4 P	14nt upstream of gene PMM1622;
1552380	+	TSS_012948	1000	657	0	1 P	12nt upstream of gene PMM1623;
1552921	+	TSS_012968	1000	188	0	0 Ai	antisense to gene(s) PMM1624;
1554498	-	TSS_028202	1000	2326	0	90 I	within gene(s) PMM1625;
1554856	-	TSS_028275	1000	11928	0	4 P	16nt upstream of gene PMM1625;
1554868	+	TSS_012991	1000	352	0	0 P	22nt upstream of gene PMM1626;
1557432	+	TSS_013003	1000	301	0	1 Ai	antisense to gene(s) PMM1629;
1557493	-	TSS_028288	1000	157	0	31 I	within gene(s) PMM1629;
1558291	-	TSS_028366	1000	159	0	2 P	18nt upstream of gene PMM1629;
1558456	+	TSS_013012	1000	368	0	6 Ai	antisense to gene(s) PMM1630;
1559240	+	TSS_013020	1000	523	0	1 Ai	antisense to gene(s) PMM1630;
1559806	-	TSS_028498	1000	4458	0	4 P	23nt upstream of gene PMM1630;
1561061	-	TSS_028510	1000	16601	0	16 IP	within gene(s) PMM1634; 205nt upstream of gene PMM1633;
1562872	-	TSS_028599	1000	1194	0	1 P	52nt upstream of gene PMM1634;
1563062	-	TSS_028602	1000	208	0	2 PAi	242nt upstream of gene PMM1634; antisense to gene(s) PMM1635;
1563853	+	TSS_028602	1000		0	0 P	71nt upstream of gene PMM1636;
1563968	-	TSS_028605	1000	416	0	0 Ai	antisense to gene(s) PMM1636;
1564062	+	TSS_013045	1000	692	0	0 I	within gene(s) PMM1636;
1564123	-	TSS_028606	1000	863	0	1 Ai	antisense to gene(s) PMM1636;
1566011	+	TSS_013064	1000	164	0	0 I	within gene(s) PMM1638;
1566190	-	TSS_028614	1000	209	0	0 Ai	antisense to gene(s) PMM1638;
1567303	-	TSS_028641	1000	116	0	3 I	within gene(s) PMM1639;
1568020	-	TSS_028672	1000	157	0	27 I	within gene(s) PMM1639;
1569177	-	TSS_028715	1000	201	0	0 P	31nt upstream of gene PMM1639;
1570531	-	TSS_028725	1000	2237	0	0 I	within gene(s) PMM1642;
1570651	-	TSS_021585	1000		0	0 P	14nt upstream of gene PMM1642;
1571420	+	TSS_013086	1000	159	0	0 Ai	antisense to gene(s) PMM1644;
1571635	-	TSS_028741	1000	551	0	0 P	26nt upstream of gene PMM1644;
1571739	+	TSS_013087	1000	212	0	0 P	14nt upstream of gene PMM1645;
1573488	-	TSS_028744	1000	93	0	0 Ai	antisense to gene(s) PMM1645;
1576258	+	TSS_013100	1000	508	0	0 I	within gene(s) PMM1648;
1576619	-	TSS_028756	1000	169	0	0 Ai	antisense to gene(s) PMM1648;
1576861	+	TSS_013109	1000	673	0	2 I	within gene(s) PMM1648;
1579478	-	TSS_028794	1000		0	0 I	within gene(s) PMM1649;
1580087	-	TSS_028809	1000	323	0	3 P	22nt upstream of gene PMM1649;
1580887	-	TSS_028818	1000	308	0	3 I	within gene(s) PMM1650;
1582233	-	TSS_028821	1000	314	0	9 I	within gene(s) PMM1652;

1582766	-	TSS_028877	1000	360	0	6	I	within gene(s) PMM1652;
1583497	-	TSS_028927	1000	449	0	4	I	within gene(s) PMM1652;
1584409	-	TSS_028950	1000	3510	0	7	I	within gene(s) PMM1653;
1584725	+	TSS_013150	1000	107	0	0	Ai	antisense to gene(s) PMM1653;
1585023	-	TSS_028955	1000	938	0	1	IP	within gene(s) PMM1654; 71nt upstream of gene PMM1653;
1587579	+	TSS_013159	1000	576	0	2	P	16nt upstream of gene PMM1656;
1588301	+	TSS_013183	1000	809	0	4	P	45nt upstream of gene PMM1657;
1589048	+	TSS_013226	1000	534	0	12	I	within gene(s) PMM1657;
1594043	-	TSS_028966	1000	104	0	0	I	within gene(s) PMM1660;
1594453	+	TSS_013250	1000	228	0	0	P	19nt upstream of gene PMM1661;
1594701	+	TSS_013255	1000	431	0	0	I	within gene(s) PMM1662;
1595654	+	TSS_013257	1000	103	0	0	I	within gene(s) PMM1664;
1596577	+	TSS_013259	1000	292	0	4	P	32nt upstream of gene PMM1665;
1598158	-	TSS_028977	1000	120	0	0	Ai	antisense to gene(s) PMM1666;
1599097	-	TSS_028992	1000	121	0	23	I	within gene(s) PMM1667;
1599261	-	TSS_029002	1000	259	0	0	P	34nt upstream of gene PMM1667;
1599742	-	TSS_029006	1000	96	0	9	I	within gene(s) PMM1668;
1600936	+	TSS_013306	1000	536	0	1	Ai	antisense to gene(s) PMM1668;
1604240	-	TSS_029126	1000	803	0	2	IP	within gene(s) PMM1671; 236nt upstream of gene PMM1670;
1604740	-	TSS_029129	1000	224	0	2	P	24nt upstream of gene PMM1671;
1604757	+	TSS_013314	1000	284	0	8	I	within gene(s) PMM1672;
1605445	+	TSS_013344	1000	260	0	4	I	within gene(s) PMM1672;
1606251	+	TSS_013379	1000	208	0	2	P	15nt upstream of gene PMM1674;
1606665	+	TSS_013382	1000	119	0	6	I	within gene(s) PMM1674;
1607611	+	TSS_013392	1000	378	0	1	IP	within gene(s) PMM1674; 49nt upstream of gene PMM1675;
1607789	+	TSS_013394	1000	120	0	0	I	within gene(s) PMM1675;
1609627	+	TSS_013401	1000	421	0	2	P	4nt upstream of gene PMM1676;
1609831	+	TSS_013402	1000	90	0	0	I	within gene(s) PMM1676;
1610827	-	TSS_029155	1000	257	0	0	O	-
1611481	+	TSS_009445	1000	3246	0	1	P	15nt upstream of gene PMM1678;
1611841	+	TSS_013419	1000	262	0	16	IP	within gene(s) PMM1678; 92nt upstream of gene PMM1679;
1612758	+	TSS_013426	1000	104	0	2	I	within gene(s) PMM1679;
1616290	+	TSS_013437	1000	964	0	2	Ai	antisense to gene(s) PMM1682;
1616930	-	TSS_029181	1000	683	0	6	P	26nt upstream of gene PMM1682;
1617422	-	TSS_029185	1000	187	0	0	P	93nt upstream of gene PMM1683;
1618013	+	TSS_013447	1000	90	0	0	I	within gene(s) PMM1684;
1618516	+	TSS_013451	1000	375	0	1	I	within gene(s) PMM1685;
1618595	+	TSS_013453	1000	124	0	3	I	within gene(s) PMM1685;
1619698	-	TSS_029189	1000	218	0	0	Ai	antisense to gene(s) PMM1686;
1619840	-	TSS_029190	1000	181	0	0	Ai	antisense to gene(s) PMM1686;
1620131	+	TSS_013460	1000	833	0	4	P	24nt upstream of gene PMM1687;
1621782	+	TSS_013470	1000	109	0	1	I	within gene(s) PMM1688;
1622476	-	TSS_029195	1000	1441	0	6	Ai	antisense to gene(s) PMM1688;
1623293	+	TSS_013483	1000	366	0	3	I	within gene(s) PMM1689;
1624911	-	TSS_029207	1000	201	0	0	Ai	antisense to gene(s) PMM1690;
1625074	-	TSS_029208	1000	184	0	0	Ai	antisense to gene(s) PMM1690;
1628185	-	TSS_029211	1000	261	0	4	Ai	antisense to gene(s) PMM1693;
1628843	-	TSS_029215	1000	186	0	1	I	within gene(s) PMM1694;
1628980	+	TSS_013557	1000	523	0	2	PAi	173nt upstream of gene PMM1695; antisense to gene(s) PMM1694;
1629107	-	TSS_029218	1000	347	0	0	P	0nt upstream of gene PMM1694;
1630728	+	TSS_013566	1000	128	0	1	I	within gene(s) PMM1696;
1632609	+	TSS_013569	1000	165	0	0	P	98nt upstream of gene PMM1697;
1632679	+	TSS_013570	1000	122	0	0	P	28nt upstream of gene PMM1697;
1633014	+	TSS_013585	1000	267	0	12	I	within gene(s) PMM1697;
1634577	+	TSS_013652	1000	220	0	0	Ai	antisense to gene(s) PMM1699;
1634772	+	TSS_013653	1000	362	0	0	Ai	antisense to gene(s) PMM1699;
1635344	+	TSS_013654	1000	140	0	2	PAi	199nt upstream of gene PMM1700; antisense to gene(s) PMM1699;
1635526	+	TSS_009555	1000	5321	0	5	P	17nt upstream of gene PMM1700;
1636527	+	TSS_013754	1000	178	0	15	I	within gene(s) PMM1700;
1638726	-	TSS_029256	1000	107	0	0	Ai	antisense to gene(s) PMM1701;
1638821	-	TSS_029257	1000	116	0	5	Ai	antisense to gene(s) PMM1701;
1639548	+	TSS_013884	1000	137	0	0	Ai	antisense to gene(s) PMM1702;
1640380	-	TSS_029263	1000	208	0	0	P	32nt upstream of gene PMM1702;
1640445	+	TSS_013886	1000	2205	0	2	I	within gene(s) PMM1703;
1642050	+	TSS_013891	1000	1029	0	1	Ai	antisense to gene(s) PMM1704;
1643399	-	TSS_029430	1000	774	0	7	P	19nt upstream of gene PMM1704;
1644415	+	TSS_013907	1000	102	0	0	P	50nt upstream of gene PMM1706;
1645357	-	TSS_029444	1000	271	0	10	I	within gene(s) PMM1707;
1646216	-	TSS_029475	1000	1910	0	0	P	69nt upstream of gene PMM1707;
1646419	+	TSS_013924	1000	1676	0	2	P	20nt upstream of gene PMM1708;
1647512	-	TSS_029480	1000	149	0	1	Ai	antisense to gene(s) PMM1709;
1648218	+	TSS_013942	1000	100	0	3	Ai	antisense to gene(s) PMM1711;
1649316	-	TSS_029485	1000	152	0	0	I	within gene(s) PMM1711;
1654524	+	TSS_013965	1000	183	0	4	I	within gene(s) PMM1714;
1656926	+	TSS_013976	1000	204	0	6	I	within gene(s) PMM1716;

**Class represents the classification of the start site. I represents internal start sites, Ai represents antisense start sites, P represents primary start sites,
IP represents internal or primary start sites, PAi represents primary or Antisense start sites, Ad represnts Antisense or downstream
IAd represents internal Antisense or downstream and O represents orphan.
