# Supplementary material for: Nitrogen cost minimization is promoted by structural changes in the transcriptome of N-deprived Prochlorococcus cells
Source: ISME J. 2017 Jun 6;11(10):2267–78. doi: 10.1038/ismej.2017.88 (PMC5607370; doi:10.1038/ismej.2017.88)
Supplement: Supplementary Table 10 [file ismej201788x17.pdf]

Table S10. N-Replete Transcriptional Start Sites at 24 Hours Post Starvation Identified by TSSAR.

Position	Strand	ID	Score	Difference	p.Value	Positional.Ur	Class	Comment
158	+	TSS_000003	1000	390	0	5	P	16nt upstream of gene PMM0001;
1089	+	TSS_000014	1000	101	0	15	IP	within gene(s) PMM0001; 244nt upstream of gene PMM0002;
1991	+	TSS_000017	1000	160	0	4	IP	within gene(s) PMM0002; 53nt upstream of gene PMM0003;
2084	+	TSS_000020	1000	2180	0	1	I	within gene(s) PMM0003;
3197	+	TSS_000029	1000	197	0	5	I	within gene(s) PMM0003;
5103	+	TSS_000034	1000	114	0	0	I	within gene(s) PMM0004;
8361	-	TSS_012240	1000	519	0	0	P	33nt upstream of gene PMM0005;
9568	+	TSS_000039	1000	153	0	5	Ai	antisense to gene(s) PMM0007;
10218	-	TSS_012245	1000	459	0	0	I	within gene(s) PMM0007;
10339	+	TSS_000042	1000	1741	0	4	P	28nt upstream of gene PMM0008;
10349	+	TSS_000045	1000	547	0	3	P	18nt upstream of gene PMM0008;
11776	+	TSS_000056	1000	171	0	4	P	17nt upstream of gene PMM0010;
14116	-	TSS_012254	1000	1436	0	3	Ai	antisense to gene(s) PMM0011;
14568	+	TSS_000073	1000	435	0	0	P	17nt upstream of gene PMM0012;
14964	-	TSS_012257	1000	235	0	6	Ai	antisense to gene(s) PMM0012;
16016	+	TSS_000082	1000	22430	0	3	P	2nt upstream of gene PMM0013;
16255	-	TSS_012262	1000	254	0	1	Ai	antisense to gene(s) PMM0013;
16313	-	TSS_012263	1000	186	0	1	Ai	antisense to gene(s) PMM0013;
16507	+	TSS_000097	1000	119	0	3	I	within gene(s) PMM0013;
17671	+	TSS_000102	1000	409	0	2	PAi	41nt upstream of gene PMM0015; antisense to gene(s) PMM0014;
18081	-	TSS_012267	1000	140	0	0	Ai	antisense to gene(s) PMM0015;
18267	+	TSS_000109	1000	751	0	3	P	16nt upstream of gene PMM0016;
18637	+	TSS_000129	1000	103	0	3	I	within gene(s) PMM0016;
18649	+	TSS_000132	1000	113	0	0	I	within gene(s) PMM0016;
18667	+	TSS_000135	1000	146	0	15	I	within gene(s) PMM0016;
18739	+	TSS_000142	1000	106	0	6	I	within gene(s) PMM0016;
18784	-	TSS_012272	1000	115	0	1	Ai	antisense to gene(s) PMM0016;
18852	-	TSS_012276	1000	158	0	0	Ai	antisense to gene(s) PMM0016;
23179	-	TSS_012283	1000	141	0	1	I	within gene(s) PMM0022;
24122	+	TSS_000171	1000	1355	0	3	P	28nt upstream of gene PMM0023;
24891	+	TSS_000195	1000	111	0	0	I	within gene(s) PMM0023;
25886	-	TSS_012300	1000	872	0	0	I	within gene(s) PMM0024;
26468	-	TSS_012304	1000	295	0	0	I	within gene(s) PMM0025;
27273	+	TSS_000221	1000	1554	0	2	P	14nt upstream of gene PMM0026;
27294	-	TSS_012316	1000	155	0	0	PAi	51nt upstream of gene PMM0025; antisense to gene(s) PMM0026;
27367	+	TSS_000223	1000	647	0	0	I	within gene(s) PMM0026;
27478	-	TSS_012318	1000	286	0	1	PAi	235nt upstream of gene PMM0025; antisense to gene(s) PMM0026;
27552	+	TSS_000225	1000	105	0	4	I	within gene(s) PMM0026;
27755	+	TSS_000229	1000	504	0	0	IP	within gene(s) PMM0026; 92nt upstream of gene PMM0027;
30271	+	TSS_000235	1000	480	0	2	I	within gene(s) PMM0030;
30946	-	TSS_012332	1000	194	0	1	P	16nt upstream of gene PMM0031;
31307	-	TSS_012336	1000	167	0	4	I	within gene(s) PMM0032;
31319	-	TSS_012338	1000	119	0	1	I	within gene(s) PMM0032;
31334	-	TSS_012341	1000	269	0	6	I	within gene(s) PMM0032;
31393	-	TSS_012345	1000	481	0	2	I	within gene(s) PMM0032;
31536	-	TSS_012353	1000	5579	0	3	P	16nt upstream of gene PMM0032;
31640	+	TSS_000246	1000	312	0	1	Ai	antisense to gene(s) PMM0033;
31718	+	TSS_000248	1000	129	0	0	Ai	antisense to gene(s) PMM0033;
32109	-	TSS_012361	1000	117	0	4	P	18nt upstream of gene PMM0033;
32374	+	TSS_000251	1000	190	0	4	I	within gene(s) PMM0034;
33078	+	TSS_000258	1000	177	0	1	Ai	antisense to gene(s) PMM0035;
33332	+	TSS_000259	1000	241	0	1	Ai	antisense to gene(s) PMM0035;
33511	-	TSS_012383	1000	106	0	9	I	within gene(s) PMM0035;
33713	-	TSS_012393	1000	3236	0	2	P	16nt upstream of gene PMM0035;
34926	+	TSS_000264	1000	274	0	0	I	within gene(s) PMM0037;
37053	-	TSS_012401	1000	167	0	0	Ai	antisense to gene(s) PMM0038;
37342	+	TSS_000274	1000	564	0	0	P	18nt upstream of gene PMM0039;
43346	+	TSS_000284	1000	152	0	0	Ai	antisense to gene(s) PMM0043;
43359	+	TSS_000285	1000	141	0	0	Ai	antisense to gene(s) PMM0043;
44029	+	TSS_000286	1000	404	0	1	Ai	antisense to gene(s) PMM0043;
44177	-	TSS_012440	1000	255	0	0	I	within gene(s) PMM0043;
44205	-	TSS_012441	1000	488	0	0	P	15nt upstream of gene PMM0043;
44220	-	TSS_012442	1000	517	0	0	P	30nt upstream of gene PMM0043;
44838	-	TSS_012445	1000	363	0	2	Ai	antisense to gene(s) PMM0044;
47672	-	TSS_012458	1000	213	0	5	I	within gene(s) PMM0045;
49002	+	TSS_000313	1000	3015	0	3	P	15nt upstream of gene PMM0046;
49452	-	TSS_012463	1000	166	0	0	Ai	antisense to gene(s) PMM0046;
50616	+	TSS_000326	1000	393	0	0	P	56nt upstream of gene PMM0048;
50659	+	TSS_000328	1000	647	0	3	P	13nt upstream of gene PMM0048;
52823	+	TSS_000359	1000	301	0	5	P	44nt upstream of gene PMM0050;
52948	-	TSS_012475	1000	240	0	1	PAi	186nt upstream of gene PMM0049; antisense to gene(s) PMM0050;
54387	-	TSS_012485	1000	272	0	21	I	within gene(s) PMM0051;
56405	+	TSS_000369	1000	166	0	1	O	-
56601	-	TSS_012500	1000	1923	0	0	O	-
58844	+	TSS_000377	1000	476	0	1	P	207nt upstream of gene PMM0056;
59479	+	TSS_000381	1000	431	0	1	P	17nt upstream of gene PMM0057;
63006	+	TSS_000395	1000	1695	0	0	IP	within gene(s) PMM0057; 126nt upstream of gene PMM0058;

63116	+	TSS_000396	1000	340	0	1	P	16nt upstream of gene PMM0058;
63461	+	TSS_000399	1000	157	0	3	I	within gene(s) PMM0058;
65570	-	TSS_012538	1000	101	0	0	P	121nt upstream of gene PMM0059;
65752	+	TSS_000405	1000	224	0	1	P	89nt upstream of gene PMM0060;
66151	+	TSS_000408	1000	184	0	0	I	within gene(s) PMM0060;
66518	+	TSS_000412	1000	155	0	5	I	within gene(s) PMM0060;
67529	-	TSS_012545	1000	229	0	1	P	16nt upstream of gene PMM0061;
67586	+	TSS_000421	1000	2519	0	2	P	66nt upstream of gene PMM0062;
67872	-	TSS_012547	1000	135	0	0	Ai	antisense to gene(s) PMM0063;
67893	+	TSS_000426	1000	396	0	6	I	within gene(s) PMM0063;
68237	+	TSS_000443	1000	272	0	3	I	within gene(s) PMM0063;
68332	+	TSS_000452	1000	115	0	28	I	within gene(s) PMM0063;
68340	-	TSS_012555	1000	171	0	2	Ai	antisense to gene(s) PMM0063;
68356	-	TSS_012557	1000	110	0	0	Ai	antisense to gene(s) PMM0063;
68372	-	TSS_012558	1000	107	0	0	Ai	antisense to gene(s) PMM0063;
68394	-	TSS_012559	1000	138	0	0	Ai	antisense to gene(s) PMM0063;
68438	-	TSS_012562	1000	187	0	8	Ai	antisense to gene(s) PMM0063;
68453	+	TSS_000466	1000	209	0	1	I	within gene(s) PMM0063;
68473	+	TSS_000468	1000	121	0	0	I	within gene(s) PMM0063;
68533	+	TSS_000473	1000	182	0	0	I	within gene(s) PMM0063;
68543	+	TSS_000475	1000	138	0	1	I	within gene(s) PMM0063;
68569	+	TSS_000482	1000	270	0	15	I	within gene(s) PMM0063;
68620	+	TSS_000486	1000	466	0	21	I	within gene(s) PMM0063;
68680	+	TSS_000496	1000	178	0	3	I	within gene(s) PMM0063;
69179	-	TSS_012565	1000	248	0	1	IP	within gene(s) PMM0065; 143nt upstream of gene PMM0064;
70887	-	TSS_012578	1000	159	0	0	I	within gene(s) PMM0065;
72400	-	TSS_012584	1000	119	0	0	P	32nt upstream of gene PMM0068;
73190	-	TSS_012586	1000	105	0	0	Ai	antisense to gene(s) PMM0069;
73910	-	TSS_012589	1000	196	0	0	Ai	antisense to gene(s) PMM0069;
74082	-	TSS_012590	1000	150	0	0	Ai	antisense to gene(s) PMM0069;
74118	-	TSS_012591	1000	277	0	0	Ai	antisense to gene(s) PMM0069;
78158	-	TSS_012609	1000	307	0	1	I	within gene(s) PMM0073;
78218	-	TSS_012611	1000	355	0	0	I	within gene(s) PMM0073;
78295	-	TSS_012612	1000	270	0	7	I	within gene(s) PMM0073;
78481	-	TSS_012619	1000	248	0	0	I	within gene(s) PMM0073;
79127	-	TSS_012635	1000	125	0	4	P	6nt upstream of gene PMM0073;
79154	-	TSS_012637	1000	728	0	4	P	33nt upstream of gene PMM0073;
80289	+	TSS_000522	1000	240	0	6	I	within gene(s) PMM0075;
80352	+	TSS_000525	1000	188	0	0	I	within gene(s) PMM0075;
82229	-	TSS_012648	1000	143	0	0	Ai	antisense to gene(s) PMM0076;
89200	-	TSS_012665	1000	195	0	4	Ai	antisense to gene(s) PMM0083;
90051	+	TSS_000566	1000	301	0	0	I	within gene(s) PMM0084;
91200	+	TSS_000576	1000	2612	0	7	IP	within gene(s) PMM0084; 60nt upstream of gene PMM0085;
91487	-	TSS_012680	1000	412	0	0	Ai	antisense to gene(s) PMM0085;
91711	-	TSS_012685	1000	764	0	1	Ai	antisense to gene(s) PMM0085;
91842	+	TSS_000586	1000	102	0	9	I	within gene(s) PMM0085;
92293	-	TSS_012688	1000	2120	0	2	P	20nt upstream of gene PMM0086;
92350	+	TSS_000587	1000	911	0	0	P	40nt upstream of gene PMM0087;
92747	+	TSS_000592	1000	1110	0	0	P	17nt upstream of gene PMM0088;
93143	-	TSS_012692	1000	707	0	2	O	-
94679	-	TSS_012694	1000	172	0	3	Ai	antisense to gene(s) PMM0089;
96428	+	TSS_000605	1000	227	0	2	P	24nt upstream of gene PMM0091;
96699	+	TSS_000625	1000	108	0	1	IP	within gene(s) PMM0091; 121nt upstream of gene PMM0092;
96919	-	TSS_012702	1000	171	0	0	Ai	antisense to gene(s) PMM0092;
97181	+	TSS_000633	1000	17424	0	10	P	19nt upstream of gene PMM0093;
98015	-	TSS_012706	1000	440	0	4	I	within gene(s) PMM0095;
98676	-	TSS_012710	1000	208	0	1	P	16nt upstream of gene PMM0095;
101137	+	TSS_000642	1000	105	0	0	Ai	antisense to gene(s) PMM0098;
105437	-	TSS_012728	1000	221	0	2	I	within gene(s) PMM0101;
105946	-	TSS_012734	1000	381	0	0	P	30nt upstream of gene PMM0101;
107836	-	TSS_012739	1000	106	0	0	I	within gene(s) PMM0103;
110281	+	TSS_000666	1000	992	0	1	Ai	antisense to gene(s) PMM0106;
114786	+	TSS_000679	1000	166	0	1	Ai	antisense to gene(s) PMM0114;
115971	+	TSS_000686	1000	110	0	0	Ai	antisense to gene(s) PMM0115;
116575	+	TSS_000689	1000	102	0	6	I	within gene(s) PMM0116;
118346	-	TSS_012782	1000	223	0	0	Ai	antisense to gene(s) PMM0118;
119698	-	TSS_012793	1000	215	0	2	P	16nt upstream of gene PMM0120;
119798	+	TSS_000715	1000	1367	0	2	P	28nt upstream of gene PMM0121;
120990	-	TSS_012801	1000	579	0	5	IP	within gene(s) PMM0123; 240nt upstream of gene PMM0122;
121764	-	TSS_012809	1000	115	0	0	P	29nt upstream of gene PMM0123;
122897	+	TSS_000726	1000	625	0	2	O	-
123251	+	TSS_000727	1000	625	0	0	P	68nt upstream of gene PMM0126;
123779	+	TSS_000730	1000	1378	0	1	I	within gene(s) PMM0126;
123796	+	TSS_000731	1000	337	0	0	I	within gene(s) PMM0126;
125323	+	TSS_000740	1000	1838	0	1	P	35nt upstream of gene PMM0128;
125778	+	TSS_000763	1000	161	0	9	I	within gene(s) PMM0128;
125808	+	TSS_000769	1000	158	0	0	I	within gene(s) PMM0128;
129278	-	TSS_012839	1000	142	0	3	I	within gene(s) PMM0131;
130071	-	TSS_012841	1000	198	0	3	P	34nt upstream of gene PMM0131;

130794	+	TSS_000781	1000	2032	0	0 Ai	antisense to gene(s) PMM0133;
130807	+	TSS_000782	1000	1063	0	0 Ai	antisense to gene(s) PMM0133;
132850	-	TSS_012851	1000	264	0	0 Ai	antisense to gene(s) PMM0134;
134228	-	TSS_012858	1000	2202	0	6 Ai	antisense to gene(s) PMM0135;
134332	+	TSS_000831	1000	312	0	1 P	17nt upstream of gene PMM0136;
135334	+	TSS_000836	1000	249	0	2 IP	within gene(s) PMM0136; 37nt upstream of gene PMM0137;
139049	+	TSS_000845	1000	133	0	0 P	49nt upstream of gene PMM0142;
139888	+	TSS_000852	1000	1082	0	1 Ai	antisense to gene(s) PMM0143;
140081	-	TSS_012872	1000	676	0	1 I	within gene(s) PMM0143;
141905	-	TSS_012895	1000	661	0	0 P	38nt upstream of gene PMM0144;
141924	+	TSS_000856	1000	216	0	5 P	34nt upstream of gene PMM0145;
142832	-	TSS_012900	1000	134	0	0 Ai	antisense to gene(s) PMM0146;
143021	-	TSS_012905	1000	105	0	2 I	within gene(s) PMM0147;
143488	-	TSS_012909	1000	614	0	3 I	within gene(s) PMM0147;
143536	-	TSS_012917	1000	144	0	12 I	within gene(s) PMM0147;
143883	-	TSS_012923	1000	234	0	12 P	14nt upstream of gene PMM0147;
143931	+	TSS_000878	1000	224	0	3 P	23nt upstream of gene PMM0148;
144701	+	TSS_000885	1000	1350	0	4 P	15nt upstream of gene PMM0149;
144965	+	TSS_000889	1000	349	0	6 I	within gene(s) PMM0149;
145139	+	TSS_000894	1000	158	0	0 I	within gene(s) PMM0149;
145211	+	TSS_000895	1000	313	0	6 I	within gene(s) PMM0149;
145226	+	TSS_000900	1000	526	0	3 I	within gene(s) PMM0149;
145511	+	TSS_000909	1000	253	0	1 I	within gene(s) PMM0149;
145715	+	TSS_000918	1000	199	0	10 I	within gene(s) PMM0149;
145730	+	TSS_000921	1000	117	0	6 I	within gene(s) PMM0149;
145799	+	TSS_000926	1000	120	0	0 I	within gene(s) PMM0149;
146423	-	TSS_012937	1000	177	0	0 Ai	antisense to gene(s) PMM0149;
146543	+	TSS_000941	1000	103	0	3 I	within gene(s) PMM0149;
146611	+	TSS_000943	1000	105	0	1 IP	within gene(s) PMM0149; 202nt upstream of gene PMM0150;
146794	+	TSS_000945	1000	18717	0	2 P	19nt upstream of gene PMM0150;
147116	+	TSS_000949	1000	103	0	0 I	within gene(s) PMM0150;
147332	+	TSS_000953	1000	388	0	0 I	within gene(s) PMM0150;
147440	+	TSS_000956	1000	128	0	0 I	within gene(s) PMM0150;
147622	-	TSS_012941	1000	141	0	0 Ai	antisense to gene(s) PMM0150;
147731	-	TSS_012942	1000	149	0	1 Ai	antisense to gene(s) PMM0150;
147807	+	TSS_000964	1000	178	0	0 I	within gene(s) PMM0150;
148515	+	TSS_000977	1000	166	0	1 P	169nt upstream of gene PMM0151;
149427	+	TSS_000981	1000	324	0	1 P	20nt upstream of gene PMM0152;
150290	+	TSS_000993	1000	207	0	0 I	within gene(s) PMM0152;
150329	+	TSS_000995	1000	110	0	3 I	within gene(s) PMM0152;
150387	-	TSS_012948	1000	195	0	4 Ai	antisense to gene(s) PMM0152;
151372	+	TSS_001000	1000	121	0	0 PAi	205nt upstream of gene PMM0154; antisense to gene(s) PMM0153;
151412	+	TSS_001002	1000	339	0	2 PAi	165nt upstream of gene PMM0154; antisense to gene(s) PMM0153;
151546	-	TSS_012959	1000	164	0	2 P	47nt upstream of gene PMM0153;
151556	+	TSS_001004	1000	883	0	5 P	21nt upstream of gene PMM0154;
151566	+	TSS_001006	1000	2253	0	1 P	11nt upstream of gene PMM0154;
154617	-	TSS_012990	1000	3508	0	42 IP	within gene(s) PMM0159; 214nt upstream of gene PMM0158;
154809	-	TSS_012997	1000	157	0	0 I	within gene(s) PMM0159;
154964	-	TSS_013012	1000	308	0	3 I	within gene(s) PMM0159;
155061	-	TSS_013013	1000	435	0	1 P	18nt upstream of gene PMM0159;
155927	-	TSS_013021	1000	131	0	6 I	within gene(s) PMM0160;
156247	-	TSS_013026	1000	130	0	4 P	16nt upstream of gene PMM0160;
157478	-	TSS_013037	1000	175	0	0 P	26nt upstream of gene PMM0161;
160552	-	TSS_013063	1000	146	0	1 I	within gene(s) PMM0164;
160684	-	TSS_013067	1000	1079	0	5 P	18nt upstream of gene PMM0164;
161048	+	TSS_001026	1000	468	0	0 P	21nt upstream of gene PMM0166;
161898	+	TSS_001033	1000	107	0	0 Ai	antisense to gene(s) PMM0167;
162263	-	TSS_013073	1000	233	0	2 P	61nt upstream of gene PMM0167;
162318	+	TSS_001035	1000	994	0	2 P	21nt upstream of gene PMM0168;
163802	-	TSS_013084	1000	390	0	0 P	32nt upstream of gene PMM0169;
163830	+	TSS_001044	1000	286	0	0 I	within gene(s) PMM0170;
165910	+	TSS_001051	1000	15044	0	3 P	15nt upstream of gene PMM0172;
166513	+	TSS_001075	1000	234	0	7 I	within gene(s) PMM0172;
166534	+	TSS_001080	1000	103	0	12 I	within gene(s) PMM0172;
166564	+	TSS_001089	1000	517	0	22 I	within gene(s) PMM0172;
166591	+	TSS_001096	1000	132	0	12 I	within gene(s) PMM0172;
166605	-	TSS_013092	1000	285	0	0 Ai	antisense to gene(s) PMM0172;
172122	-	TSS_013102	1000	101	0	0 Ai	antisense to gene(s) PMM0177;
173360	-	TSS_013104	1000	421	0	2 P	17nt upstream of gene PMM0179;
173404	+	TSS_001120	1000	709	0	0 P	16nt upstream of gene PMM0180;
178914	-	TSS_013117	1000	831	0	2 I	within gene(s) PMM0187;
180315	+	TSS_001133	1000	135	0	0 Ai	antisense to gene(s) PMM0188;
182433	-	TSS_013121	1000	260	0	2 PAi	92nt upstream of gene PMM0189; antisense to gene(s) PMM0190;
183419	-	TSS_013124	1000	166	0	0 I	within gene(s) PMM0191;
188865	+	TSS_001152	1000	540	0	1 P	18nt upstream of gene PMM0195;
189007	+	TSS_001154	1000	259	0	0 I	within gene(s) PMM0195;
189285	+	TSS_001161	1000	135	0	0 I	within gene(s) PMM0195;
189333	+	TSS_001165	1000	143	0	7 I	within gene(s) PMM0195;
189342	+	TSS_001167	1000	177	0	0 I	within gene(s) PMM0195;

189435	+	TSS_001174	1000	116	0	6 I	within gene(s) PMM0195;
189468	+	TSS_001181	1000	246	0	12 I	within gene(s) PMM0195;
189748	-	TSS_013141	1000	122	0	0 Ai	antisense to gene(s) PMM0195;
195220	-	TSS_013155	1000	112	0	0 IP	within gene(s) PMM0201; 249nt upstream of gene PMM0200;
195266	+	TSS_001210	1000	1474	0	4 Ai	antisense to gene(s) PMM0201;
195316	-	TSS_013163	1000	254	0	12 I	within gene(s) PMM0201;
195328	-	TSS_013165	1000	292	0	3 I	within gene(s) PMM0201;
195358	-	TSS_013168	1000	103	0	0 I	within gene(s) PMM0201;
195367	-	TSS_013169	1000	241	0	3 I	within gene(s) PMM0201;
195388	-	TSS_013173	1000	213	0	1 I	within gene(s) PMM0201;
195626	-	TSS_013181	1000	119	0	7 IP	within gene(s) PMM0202; 214nt upstream of gene PMM0201;
195665	-	TSS_013184	1000	263	0	4 I	within gene(s) PMM0202;
195760	-	TSS_013191	1000	393	0	2 I	within gene(s) PMM0202;
195809	-	TSS_013197	1000	229	0	3 I	within gene(s) PMM0202;
195869	-	TSS_013204	1000	151	0	2 I	within gene(s) PMM0202;
196098	-	TSS_013208	1000	103	0	0 P	130nt upstream of gene PMM0202;
196510	+	TSS_001220	1000	230	0	1 Ai	antisense to gene(s) PMM0203;
196627	-	TSS_013229	1000	117	0	6 I	within gene(s) PMM0203;
196639	-	TSS_013230	1000	171	0	0 I	within gene(s) PMM0203;
196677	+	TSS_001223	1000	173	0	0 Ai	antisense to gene(s) PMM0203;
197001	-	TSS_013242	1000	854	0	2 IP	within gene(s) PMM0204; 133nt upstream of gene PMM0203;
197448	-	TSS_013256	1000	559	0	3 IP	within gene(s) PMM0205; 88nt upstream of gene PMM0204;
197685	+	TSS_001226	1000	156	0	0 Ai	antisense to gene(s) PMM0205;
197771	-	TSS_013269	1000	182	0	0 I	within gene(s) PMM0205;
197837	-	TSS_013272	1000	161	0	0 I	within gene(s) PMM0205;
197876	-	TSS_013276	1000	156	0	5 I	within gene(s) PMM0205;
198069	-	TSS_013284	1000	654	0	5 P	34nt upstream of gene PMM0205;
198181	-	TSS_013289	1000	290	0	0 IP	within gene(s) PMM0206; 146nt upstream of gene PMM0205;
198382	-	TSS_013290	1000	250	0	0 P	33nt upstream of gene PMM0206;
199933	-	TSS_013297	1000	141	0	3 I	within gene(s) PMM0207;
201442	+	TSS_001235	1000	3101	0	5 P	14nt upstream of gene PMM0208;
202297	-	TSS_013306	1000	118	0	0 Ai	antisense to gene(s) PMM0208;
202306	-	TSS_013307	1000	116	0	0 Ai	antisense to gene(s) PMM0208;
203176	-	TSS_013316	1000	193	0	1 I	within gene(s) PMM0209;
203219	-	TSS_013318	1000	609	0	0 I	within gene(s) PMM0209;
204910	+	TSS_001276	1000	1328	0	4 P	82nt upstream of gene PMM0211;
205563	-	TSS_013326	1000	206	0	6 Ai	antisense to gene(s) PMM0211;
205900	-	TSS_013334	1000	1548	0	1 Ai	antisense to gene(s) PMM0211;
206424	-	TSS_013337	1000	495	0	2 IP	within gene(s) PMM0213; 172nt upstream of gene PMM0212;
207635	-	TSS_013349	1000	102	0	0 I	within gene(s) PMM0214;
207653	-	TSS_013350	1000	125	0	12 I	within gene(s) PMM0214;
207728	-	TSS_013359	1000	117	0	17 I	within gene(s) PMM0214;
208325	-	TSS_013395	1000	139	0	1 I	within gene(s) PMM0214;
208516	-	TSS_013400	1000	108	0	1 I	within gene(s) PMM0214;
208538	-	TSS_013405	1000	194	0	12 I	within gene(s) PMM0214;
208631	+	TSS_001325	1000	111	0	0 Ai	antisense to gene(s) PMM0214;
208702	+	TSS_001327	1000	226	0	1 Ai	antisense to gene(s) PMM0214;
208724	-	TSS_013417	1000	122	0	13 I	within gene(s) PMM0214;
208811	-	TSS_013426	1000	206	0	1 I	within gene(s) PMM0214;
208944	-	TSS_013435	1000	7143	0	4 P	28nt upstream of gene PMM0214;
209113	+	TSS_001329	1000	527	0	1 P	31nt upstream of gene PMM0215;
210185	+	TSS_001341	1000	193	0	1 P	16nt upstream of gene PMM0216;
211988	-	TSS_013443	1000	664	0	1 Ai	antisense to gene(s) PMM0217;
213029	+	TSS_001353	1000	472	0	6 P	33nt upstream of gene PMM0218;
214116	+	TSS_001359	1000	1375	0	3 P	17nt upstream of gene PMM0219;
214675	-	TSS_013448	1000	4502	0	5 P	19nt upstream of gene PMM0220;
216078	+	TSS_001365	1000	104	0	0 I	within gene(s) PMM0222;
216861	+	TSS_001367	1000	22207	0	4 I	within gene(s) PMM0223;
216876	+	TSS_001370	1000	682	0	0 I	within gene(s) PMM0223;
216900	+	TSS_001373	1000	1252	0	3 I	within gene(s) PMM0223;
216909	+	TSS_001374	1000	527	0	3 I	within gene(s) PMM0223;
216924	+	TSS_001376	1000	460	0	0 I	within gene(s) PMM0223;
216936	+	TSS_001377	1000	1402	0	12 I	within gene(s) PMM0223;
217002	+	TSS_001393	1000	7965	0	63 I	within gene(s) PMM0223;
217029	+	TSS_001399	1000	4334	0	1 I	within gene(s) PMM0223;
217050	+	TSS_001403	1000	2180	0	10 I	within gene(s) PMM0223;
217078	+	TSS_001407	1000	907	0	9 I	within gene(s) PMM0223;
217090	-	TSS_013455	1000	782	0	6 Ai	antisense to gene(s) PMM0223;
217099	+	TSS_001412	1000	1655	0	16 I	within gene(s) PMM0223;
217119	+	TSS_001416	1000	1887	0	15 I	within gene(s) PMM0223;
217164	+	TSS_001427	1000	2250	0	16 I	within gene(s) PMM0223;
217169	-	TSS_013457	1000	4802	0	1 Ai	antisense to gene(s) PMM0223;
217173	+	TSS_001429	1000	561	0	0 I	within gene(s) PMM0223;
217184	-	TSS_013459	1000	230	0	0 Ai	antisense to gene(s) PMM0223;
217185	+	TSS_001430	1000	482	0	0 I	within gene(s) PMM0223;
217194	+	TSS_001433	1000	10329	0	27 I	within gene(s) PMM0223;
217301	-	TSS_013460	1000	198	0	0 Ai	antisense to gene(s) PMM0223;
217328	-	TSS_013461	1000	1610	0	0 Ai	antisense to gene(s) PMM0223;
217407	-	TSS_013465	1000	310	0	1 Ai	antisense to gene(s) PMM0223;

217415	-	TSS_013466	1000	272	0	0	Ai	antisense to gene(s) PMM0223;
217496	-	TSS_013468	1000	103	0	1	Ai	antisense to gene(s) PMM0223;
217587	-	TSS_013469	1000	110	0	0	Ai	antisense to gene(s) PMM0223;
217670	-	TSS_013471	1000	298	0	4	Ai	antisense to gene(s) PMM0223;
217681	-	TSS_013472	1000	175	0	0	Ai	antisense to gene(s) PMM0223;
217716	-	TSS_013473	1000	120	0	5	Ai	antisense to gene(s) PMM0223;
217753	-	TSS_013476	1000	103	0	0	Ai	antisense to gene(s) PMM0223;
217826	-	TSS_013477	1000	202	0	0	Ai	antisense to gene(s) PMM0223;
217978	+	TSS_001452	1000	1703	0	2	P	24nt upstream of gene PMM0224;
218599	+	TSS_001466	1000	337	0	10	I	within gene(s) PMM0224;
218695	+	TSS_001473	1000	155	0	6	I	within gene(s) PMM0224;
220029	-	TSS_013495	1000	367	0	7	I	within gene(s) PMM0226;
220073	+	TSS_001486	1000	789	0	0	Ai	antisense to gene(s) PMM0226;
220087	+	TSS_001487	1000	338	0	0	Ai	antisense to gene(s) PMM0226;
220272	-	TSS_013525	1000	759	0	55	I	within gene(s) PMM0226;
220296	-	TSS_013530	1000	615	0	1	I	within gene(s) PMM0226;
220320	-	TSS_013532	1000	314	0	9	I	within gene(s) PMM0226;
220338	-	TSS_013537	1000	217	0	26	I	within gene(s) PMM0226;
220370	-	TSS_013543	1000	470	0	0	I	within gene(s) PMM0226;
220389	-	TSS_013544	1000	114	0	0	I	within gene(s) PMM0226;
220444	+	TSS_001491	1000	196	0	4	Ai	antisense to gene(s) PMM0226;
220458	-	TSS_013546	1000	112	0	0	I	within gene(s) PMM0226;
220473	-	TSS_013547	1000	119	0	0	I	within gene(s) PMM0226;
220509	-	TSS_013550	1000	104	0	15	I	within gene(s) PMM0226;
220578	-	TSS_013562	1000	854	0	24	I	within gene(s) PMM0226;
220608	-	TSS_013569	1000	125	0	14	I	within gene(s) PMM0226;
220686	-	TSS_013580	1000	122	0	0	I	within gene(s) PMM0226;
220786	+	TSS_001495	1000	255	0	3	Ai	antisense to gene(s) PMM0226;
220908	-	TSS_013595	1000	110	0	3	I	within gene(s) PMM0226;
220950	-	TSS_013600	1000	239	0	13	I	within gene(s) PMM0226;
221025	-	TSS_013608	1000	174	0	15	I	within gene(s) PMM0226;
221070	-	TSS_013616	1000	140	0	2	I	within gene(s) PMM0226;
221154	-	TSS_013622	1000	399	0	3	I	within gene(s) PMM0226;
221163	-	TSS_013623	1000	109	0	0	I	within gene(s) PMM0226;
221259	-	TSS_013627	1000	216	0	4	I	within gene(s) PMM0226;
221460	-	TSS_013640	1000	228	0	13	I	within gene(s) PMM0226;
221632	-	TSS_013648	1000	4835	0	1	P	16nt upstream of gene PMM0226;
221701	+	TSS_001506	1000	245	0	0	Ai	antisense to gene(s) PMM0227;
221978	+	TSS_001509	1000	227	0	0	Ai	antisense to gene(s) PMM0227;
222942	+	TSS_001514	1000	143	0	0	Ai	antisense to gene(s) PMM0228;
223242	-	TSS_013665	1000	299	0	21	I	within gene(s) PMM0228;
223269	-	TSS_013672	1000	851	0	9	I	within gene(s) PMM0228;
223311	-	TSS_013681	1000	520	0	28	I	within gene(s) PMM0228;
223335	-	TSS_013687	1000	149	0	0	I	within gene(s) PMM0228;
223386	-	TSS_013689	1000	419	0	6	I	within gene(s) PMM0228;
223434	-	TSS_013692	1000	152	0	0	I	within gene(s) PMM0228;
223554	-	TSS_013700	1000	111	0	17	I	within gene(s) PMM0228;
223578	-	TSS_013707	1000	1224	0	19	I	within gene(s) PMM0228;
223651	-	TSS_013715	1000	111	0	1	I	within gene(s) PMM0228;
223762	-	TSS_013717	1000	1382	0	0	P	55nt upstream of gene PMM0228;
223847	-	TSS_013718	1000	598	0	1	P	140nt upstream of gene PMM0228;
225530	+	TSS_001522	1000	147	0	4	P	60nt upstream of gene PMM0231;
225576	+	TSS_001523	1000	691	0	0	P	14nt upstream of gene PMM0231;
225783	+	TSS_001526	1000	171	0	0	P	26nt upstream of gene PMM0232;
227959	+	TSS_001530	1000	871	0	0	P	28nt upstream of gene PMM0235;
228961	+	TSS_001540	1000	108	0	3	O	-
229314	-	TSS_013733	1000	167	0	0	O	-
229472	+	TSS_001546	1000	155	0	4	P	167nt upstream of gene PMM0237;
230878	-	TSS_013736	1000	561	0	5	Ai	antisense to gene(s) PMM0238;
232150	+	TSS_001575	1000	162	0	1	I	within gene(s) PMM0238;
237523	-	TSS_013760	1000	1703	0	3	IP	within gene(s) PMM0244; 199nt upstream of gene PMM0243;
237737	-	TSS_013761	1000	206	0	0	I	within gene(s) PMM0244;
238520	-	TSS_013765	1000	682	0	1	P	20nt upstream of gene PMM0245;
238693	+	TSS_001588	1000	2458	0	3	P	12nt upstream of gene PMM0246;
239672	-	TSS_013767	1000	133	0	3	Ai	antisense to gene(s) PMM0247;
241176	-	TSS_013770	1000	188	0	0	IP	within gene(s) PMM0250; 35nt upstream of gene PMM0249;
242059	+	TSS_001595	1000	179	0	0	PAi	162nt upstream of gene PMM0252; antisense to gene(s) PMM0251;
242165	-	TSS_013773	1000	419	0	4	P	24nt upstream of gene PMM0251;
242185	+	TSS_001596	1000	1358	0	1	P	36nt upstream of gene PMM0252;
242475	+	TSS_001599	1000	227	0	1	P	27nt upstream of gene PMM0253;
242483	+	TSS_001600	1000	515	0	0	P	19nt upstream of gene PMM0253;
244978	+	TSS_001603	1000	173	0	4	Ai	antisense to gene(s) PMM0255;
245524	-	TSS_013782	1000	628	0	10	IP	within gene(s) PMM0256; 219nt upstream of gene PMM0255;
245888	-	TSS_013791	1000	129	0	2	I	within gene(s) PMM0256;
246248	+	TSS_001607	1000	2696	0	1	Ai	antisense to gene(s) PMM0256;
246731	-	TSS_013797	1000	1762	0	3	IP	within gene(s) PMM0257; 20nt upstream of gene PMM0256;
248216	-	TSS_013802	1000	185	0	0	IP	within gene(s) PMM0258; 190nt upstream of gene PMM0257;
249242	-	TSS_013846	1000	576	0	2	I	within gene(s) PMM0258;
249485	+	TSS_001622	1000	142	0	2	P	22nt upstream of gene PMM0259;

251358	+	TSS_001626	1000	137	0	3	Ai	antisense to gene(s) PMM0261;
252586	+	TSS_001630	1000	405015	0	3	P	46nt upstream of gene PMM0263;
252656	+	TSS_001635	1000	4233	0	7	I	within gene(s) PMM0263;
252667	+	TSS_001637	1000	774	0	0	I	within gene(s) PMM0263;
252710	+	TSS_001642	1000	1535	0	4	I	within gene(s) PMM0263;
252719	+	TSS_001643	1000	3100	0	18	I	within gene(s) PMM0263;
252761	+	TSS_001651	1000	440	0	0	I	within gene(s) PMM0263;
252794	+	TSS_001652	1000	619	0	0	I	within gene(s) PMM0263;
252809	+	TSS_001654	1000	1363	0	6	I	within gene(s) PMM0263;
252835	-	TSS_013858	1000	233	0	0	Ai	antisense to gene(s) PMM0263;
252851	+	TSS_001657	1000	192	2.10E-07	0	I	within gene(s) PMM0263;
252875	+	TSS_001661	1000	3906	0	3	I	within gene(s) PMM0263;
252893	+	TSS_001662	1000	3058	0	0	I	within gene(s) PMM0263;
252895	-	TSS_013859	1000	260	0	0	Ai	antisense to gene(s) PMM0263;
252904	-	TSS_013860	1000	192	0	0	Ai	antisense to gene(s) PMM0263;
252905	+	TSS_001664	1000	551	0	6	I	within gene(s) PMM0263;
252926	+	TSS_001667	1000	2293	0	6	I	within gene(s) PMM0263;
252936	+	TSS_001670	1000	254	0	0	I	within gene(s) PMM0263;
252950	+	TSS_001671	1000	335	0	0	I	within gene(s) PMM0263;
252986	+	TSS_001672	1000	1023	0	0	I	within gene(s) PMM0263;
253009	+	TSS_001675	1000	488	0	2	I	within gene(s) PMM0263;
253023	-	TSS_013863	1000	1132	0	4	Ai	antisense to gene(s) PMM0263;
253040	+	TSS_001677	1000	458	0	15	I	within gene(s) PMM0263;
253056	+	TSS_001681	1000	213	0	0	I	within gene(s) PMM0263;
253072	+	TSS_001682	1000	4448	0	1	I	within gene(s) PMM0263;
253088	+	TSS_001687	1000	300	0	8	I	within gene(s) PMM0263;
253106	+	TSS_001688	1000	197	0	0	I	within gene(s) PMM0263;
253114	-	TSS_013866	1000	417	0	0	Ai	antisense to gene(s) PMM0263;
253118	+	TSS_001691	1000	209	0	3	I	within gene(s) PMM0263;
253136	+	TSS_001692	1000	1226	0	1	I	within gene(s) PMM0263;
253148	+	TSS_001696	1000	1243	0	13	I	within gene(s) PMM0263;
253169	+	TSS_001701	1000	7594	0	2	I	within gene(s) PMM0263;
253220	+	TSS_001705	1000	901	0	11	I	within gene(s) PMM0263;
253244	+	TSS_001710	1000	563	0	9	I	within gene(s) PMM0263;
253257	-	TSS_013868	1000	110	0	0	Ai	antisense to gene(s) PMM0263;
253265	+	TSS_001713	1000	601	0	12	I	within gene(s) PMM0263;
253271	-	TSS_013871	1000	673	0	7	Ai	antisense to gene(s) PMM0263;
253279	-	TSS_013872	1000	957	0	0	Ai	antisense to gene(s) PMM0263;
253286	+	TSS_001715	1000	549	0	1	I	within gene(s) PMM0263;
253289	-	TSS_013873	1000	1018	0	5	Ai	antisense to gene(s) PMM0263;
253302	+	TSS_001718	1000	347	0	3	I	within gene(s) PMM0263;
253321	-	TSS_013876	1000	577	0	0	Ai	antisense to gene(s) PMM0263;
253328	+	TSS_001724	1000	3290	0	90	I	within gene(s) PMM0263;
253329	-	TSS_013877	1000	137	0	0	Ai	antisense to gene(s) PMM0263;
253357	-	TSS_013878	1000	357	0	0	Ai	antisense to gene(s) PMM0263;
253418	+	TSS_001749	1000	922	0	15	I	within gene(s) PMM0263;
253435	-	TSS_013880	1000	394	0	0	Ai	antisense to gene(s) PMM0263;
253452	-	TSS_013882	1000	300	0	1	Ai	antisense to gene(s) PMM0263;
253460	+	TSS_001754	1000	314	0	0	I	within gene(s) PMM0263;
253469	+	TSS_001755	1000	194	6.70E-08	0	I	within gene(s) PMM0263;
253481	+	TSS_001758	1000	219	0	3	I	within gene(s) PMM0263;
253491	+	TSS_001760	1000	662	0	1	I	within gene(s) PMM0263;
253498	-	TSS_013883	1000	404	0	0	Ai	antisense to gene(s) PMM0263;
253502	+	TSS_001761	1000	198	1.40E-06	0	I	within gene(s) PMM0263;
253520	-	TSS_013885	1000	139	0	0	Ai	antisense to gene(s) PMM0263;
253529	+	TSS_001767	1000	2214	0	30	I	within gene(s) PMM0263;
253548	+	TSS_001773	1000	317	0	2	I	within gene(s) PMM0263;
253562	+	TSS_001776	1000	570	0	12	I	within gene(s) PMM0263;
253580	+	TSS_001780	1000	2353	0	1	I	within gene(s) PMM0263;
253592	+	TSS_001782	1000	1016	0	1	I	within gene(s) PMM0263;
253606	-	TSS_013886	1000	2753	0	0	Ai	antisense to gene(s) PMM0263;
253616	+	TSS_001791	1000	2271	0	18	I	within gene(s) PMM0263;
253641	+	TSS_001798	1000	1958	0	18	I	within gene(s) PMM0263;
253648	-	TSS_013888	1000	122	0	0	Ai	antisense to gene(s) PMM0263;
253667	+	TSS_001802	1000	4912	0	12	I	within gene(s) PMM0263;
253688	+	TSS_001809	1000	398	0	0	I	within gene(s) PMM0263;
253698	+	TSS_001810	1000	472	0	0	I	within gene(s) PMM0263;
253739	+	TSS_001815	1000	7231	0	42	I	within gene(s) PMM0263;
253778	+	TSS_001825	1000	1132	0	3	I	within gene(s) PMM0263;
253787	+	TSS_001826	1000	1973	0	6	I	within gene(s) PMM0263;
253811	+	TSS_001830	1000	705	0	0	I	within gene(s) PMM0263;
253820	+	TSS_001831	1000	300	0	1	I	within gene(s) PMM0263;
253836	+	TSS_001834	1000	2785	0	1	I	within gene(s) PMM0263;
253848	+	TSS_001838	1000	251	0	4	I	within gene(s) PMM0263;
254043	-	TSS_013889	1000	234	0	0	Ai	antisense to gene(s) PMM0263;
254055	-	TSS_013890	1000	442	0	0	Ai	antisense to gene(s) PMM0263;
254183	+	TSS_001848	1000	363	0	5	I	within gene(s) PMM0264;
255509	+	TSS_001850	1000	218	0	1	I	within gene(s) PMM0265;
255521	+	TSS_001853	1000	355	0	15	I	within gene(s) PMM0265;

255551	+	TSS_001859	1000	144	0	0 I	within gene(s) PMM0265;
255644	+	TSS_001862	1000	211	0	21 I	within gene(s) PMM0265;
255683	+	TSS_001869	1000	172	0	3 I	within gene(s) PMM0265;
255731	+	TSS_001878	1000	183	0	24 I	within gene(s) PMM0265;
255746	+	TSS_001882	1000	236	0	9 I	within gene(s) PMM0265;
255779	+	TSS_001890	1000	144	0	15 I	within gene(s) PMM0265;
255812	+	TSS_001896	1000	115	0	1 I	within gene(s) PMM0265;
256772	+	TSS_001902	1000	121	0	0 Ai	antisense to gene(s) PMM0266;
258284	+	TSS_001904	1000	109	0	0 Ai	antisense to gene(s) PMM0268;
258297	+	TSS_001906	1000	144	0	1 Ai	antisense to gene(s) PMM0268;
258382	+	TSS_001908	1000	152	0	0 Ai	antisense to gene(s) PMM0268;
258478	-	TSS_013913	1000	570	0	3 I	within gene(s) PMM0268;
258539	+	TSS_001909	1000	349	0	0 Ai	antisense to gene(s) PMM0268;
258616	-	TSS_013923	1000	187	0	3 P	12nt upstream of gene PMM0268;
261925	+	TSS_001924	1000	4850	0	4 P	23nt upstream of gene PMM0272;
263126	-	TSS_013938	1000	511	0	4 P	19nt upstream of gene PMM0273;
263543	+	TSS_001932	1000	376	0	1 Ai	antisense to gene(s) PMM0274;
264561	-	TSS_013945	1000	144	0	0 P	135nt upstream of gene PMM0274;
264640	+	TSS_001936	1000	207	0	2 P	18nt upstream of gene PMM0275;
269162	+	TSS_001954	1000	439	0	2 I	within gene(s) PMM0279;
271937	-	TSS_013985	1000	349	0	0 IP	within gene(s) PMM0282; 173nt upstream of gene PMM0281;
273322	+	TSS_001964	1000	325	0	3 I	within gene(s) PMM0284;
274872	-	TSS_014014	1000	194	0	0 I	within gene(s) PMM0285;
277117	+	TSS_001970	1000	394	0	0 PAi	54nt upstream of gene PMM0288; antisense to gene(s) PMM0287;
277159	+	TSS_001971	1000	179	0	0 P	12nt upstream of gene PMM0288;
279001	+	TSS_001990	1000	122	0	0 I	within gene(s) PMM0288;
280333	-	TSS_014032	1000	1154	0	1 IP	within gene(s) PMM0290; 149nt upstream of gene PMM0289;
281596	+	TSS_002000	1000	108	0	0 I	within gene(s) PMM0291;
283477	-	TSS_014053	1000	165	0	21 I	within gene(s) PMM0293;
283519	-	TSS_014060	1000	395	0	16 I	within gene(s) PMM0293;
283774	-	TSS_014078	1000	116	0	0 IP	within gene(s) PMM0294; 15nt upstream of gene PMM0293;
283811	-	TSS_014079	1000	3495	0	0 IP	within gene(s) PMM0294; 52nt upstream of gene PMM0293;
283840	-	TSS_014080	1000	200	0	0 IP	within gene(s) PMM0294; 81nt upstream of gene PMM0293;
283853	-	TSS_014081	1000	276	0	0 IP	within gene(s) PMM0294; 94nt upstream of gene PMM0293;
283904	-	TSS_014083	1000	2729	0	1 IP	within gene(s) PMM0294; 145nt upstream of gene PMM0293;
284179	-	TSS_014085	1000	608	0	10 P	53nt upstream of gene PMM0294;
284302	-	TSS_014090	1000	3581	0	2 PAi	176nt upstream of gene PMM0294; antisense to gene(s) PMM0295;
284638	+	TSS_002014	1000	1602	0	1 I	within gene(s) PMM0296;
284851	+	TSS_002022	1000	374	0	1 I	within gene(s) PMM0296;
285126	+	TSS_002034	1000	120	0	6 I	within gene(s) PMM0296;
285147	+	TSS_002037	1000	164	0	1 I	within gene(s) PMM0296;
285370	+	TSS_002047	1000	117	0	5 I	within gene(s) PMM0296;
285545	-	TSS_014101	1000	215	0	1 Ai	antisense to gene(s) PMM0296;
285768	+	TSS_002051	1000	35742	0	2 P	9nt upstream of gene PMM0297;
285794	+	TSS_002053	1000	171	0	5 IP	within gene(s) PMM0297; 240nt upstream of gene PMM0298;
285818	+	TSS_002057	1000	112	0	3 IP	within gene(s) PMM0297; 216nt upstream of gene PMM0298;
285844	+	TSS_002062	1000	239	0	3 IP	within gene(s) PMM0297; 190nt upstream of gene PMM0298;
285930	+	TSS_002069	1000	230	0	21 IP	within gene(s) PMM0297; 104nt upstream of gene PMM0298;
285945	+	TSS_002073	1000	454	0	10 IP	within gene(s) PMM0297; 89nt upstream of gene PMM0298;
285990	+	TSS_002081	1000	239	0	13 IP	within gene(s) PMM0297; 44nt upstream of gene PMM0298;
286014	+	TSS_002083	1000	128	0	0 IP	within gene(s) PMM0297; 20nt upstream of gene PMM0298;
286058	+	TSS_002088	1000	406	0	12 IP	within gene(s) PMM0298; 131nt upstream of gene PMM0299;
286103	+	TSS_002092	1000	151	0	6 IP	within gene(s) PMM0298; 86nt upstream of gene PMM0299;
286115	+	TSS_002093	1000	151	0	0 IP	within gene(s) PMM0298; 74nt upstream of gene PMM0299;
286157	+	TSS_002097	1000	124	0	16 IP	within gene(s) PMM0298; 32nt upstream of gene PMM0299;
286195	+	TSS_002101	1000	595	0	41 IP	within gene(s) PMM0299; 123nt upstream of gene PMM0300;
286246	+	TSS_002118	1000	426	0	6 IP	within gene(s) PMM0299; 72nt upstream of gene PMM0300;
286270	+	TSS_002122	1000	1598	0	22 IP	within gene(s) PMM0299; 48nt upstream of gene PMM0300;
286292	+	TSS_002129	1000	114	0	1 IP	within gene(s) PMM0299; 26nt upstream of gene PMM0300;
286319	+	TSS_002130	1000	329	0	2 I	within gene(s) PMM0300;
286339	+	TSS_002134	1000	425	0	18 I	within gene(s) PMM0300;
286530	-	TSS_014108	1000	138	0	0 Ad	antisense to gene(s) PMM0300 (19nt downstream);
286877	+	TSS_002145	1000	240	0	1 Ai	antisense to gene(s) PMM0301;
287552	-	TSS_014122	1000	785	0	1 PAi	83nt upstream of gene PMM0301; antisense to gene(s) PMM0302;
287702	-	TSS_014125	1000	482	0	4 PAi	233nt upstream of gene PMM0301; antisense to gene(s) PMM0302;
290102	+	TSS_002152	1000	155	0	0 Ai	antisense to gene(s) PMM0303;
290195	+	TSS_002153	1000	211	0	0 Ai	antisense to gene(s) PMM0303;
291317	-	TSS_014133	1000	188	0	0 I	within gene(s) PMM0304;
293682	+	TSS_002156	1000	792	0	2 P	28nt upstream of gene PMM0305;
294128	-	TSS_014143	1000	291	0	1 Ai	antisense to gene(s) PMM0305;
294947	-	TSS_014151	1000	1188	0	2 P	21nt upstream of gene PMM0307;
298142	-	TSS_014173	1000	106	0	15 I	within gene(s) PMM0311;
298181	-	TSS_014179	1000	134	0	3 I	within gene(s) PMM0311;
298704	+	TSS_002169	1000	109	0	0 Ai	antisense to gene(s) PMM0311;
299042	-	TSS_014213	1000	5214	0	6 P	18nt upstream of gene PMM0311;
299206	+	TSS_002170	1000	116	0	1 Ai	antisense to gene(s) PMM0312;
299219	+	TSS_002173	1000	140	0	1 Ai	antisense to gene(s) PMM0312;
299410	-	TSS_014226	1000	151	0	14 I	within gene(s) PMM0312;
299442	-	TSS_014231	1000	156	0	1 I	within gene(s) PMM0312;

299500	-	TSS_014235	1000	135	0	0	I	within gene(s) PMM0312;
299534	+	TSS_002176	1000	133	0	0	Ai	antisense to gene(s) PMM0312;
299595	+	TSS_002178	1000	109	0	1	Ai	antisense to gene(s) PMM0312;
299713	-	TSS_014243	1000	210	0	21	I	within gene(s) PMM0312;
299749	-	TSS_014249	1000	164	0	6	I	within gene(s) PMM0312;
299824	-	TSS_014254	1000	154	0	3	I	within gene(s) PMM0312;
299836	-	TSS_014256	1000	193	0	3	I	within gene(s) PMM0312;
299850	+	TSS_002182	1000	128	0	1	Ai	antisense to gene(s) PMM0312;
299950	-	TSS_014263	1000	170	0	3	I	within gene(s) PMM0312;
300325	-	TSS_014272	1000	5818	0	10	P	75nt upstream of gene PMM0312;
300858	-	TSS_014280	1000	3547	0	12	P	21nt upstream of gene PMM0313;
301015	+	TSS_002184	1000	128	0	0	Ai	antisense to gene(s) PMM0314;
301023	+	TSS_002185	1000	334	0	0	Ai	antisense to gene(s) PMM0314;
301087	+	TSS_002187	1000	101	0	0	Ai	antisense to gene(s) PMM0315;
301149	+	TSS_002188	1000	121	0	0	Ai	antisense to gene(s) PMM0315;
301160	+	TSS_002189	1000	276	0	0	Ai	antisense to gene(s) PMM0315;
301163	-	TSS_014292	1000	356	0	12	IP	within gene(s) PMM0315; 124nt upstream of gene PMM0314;
301180	-	TSS_014297	1000	604	0	6	IP	within gene(s) PMM0315; 141nt upstream of gene PMM0314;
301201	-	TSS_014300	1000	143	0	0	IP	within gene(s) PMM0315; 162nt upstream of gene PMM0314;
301210	-	TSS_014301	1000	114	0	0	IP	within gene(s) PMM0315; 171nt upstream of gene PMM0314;
301222	-	TSS_014302	1000	363	0	6	IP	within gene(s) PMM0315; 183nt upstream of gene PMM0314;
301258	-	TSS_014308	1000	216	0	6	IP	within gene(s) PMM0315; 219nt upstream of gene PMM0314;
301351	+	TSS_002191	1000	122	0	0	Ai	antisense to gene(s) PMM0315;
301547	+	TSS_002196	1000	176	0	0	Ai	antisense to gene(s) PMM0315;
301603	-	TSS_014309	1000	155	0	0	I	within gene(s) PMM0315;
301607	+	TSS_002198	1000	129	0	0	Ai	antisense to gene(s) PMM0315;
301612	-	TSS_014311	1000	844	0	7	I	within gene(s) PMM0315;
301642	-	TSS_014318	1000	1131	0	27	I	within gene(s) PMM0315;
301654	+	TSS_002200	1000	106	0	0	Ai	antisense to gene(s) PMM0315;
301672	-	TSS_014326	1000	166	0	0	I	within gene(s) PMM0315;
301701	-	TSS_014331	1000	2179	0	30	I	within gene(s) PMM0315;
301721	+	TSS_002203	1000	413	0	1	Ai	antisense to gene(s) PMM0315;
301735	+	TSS_002204	1000	242	0	0	Ai	antisense to gene(s) PMM0315;
301735	-	TSS_014339	1000	210	0	6	I	within gene(s) PMM0315;
301769	+	TSS_002207	1000	101	0	0	Ai	antisense to gene(s) PMM0315;
301771	-	TSS_014341	1000	191	0	0	I	within gene(s) PMM0315;
301780	+	TSS_002208	1000	136	0	0	Ai	antisense to gene(s) PMM0315;
301782	-	TSS_014342	1000	539	0	7	I	within gene(s) PMM0315;
301810	-	TSS_014345	1000	235	0	0	I	within gene(s) PMM0315;
301834	-	TSS_014347	1000	1025	0	10	I	within gene(s) PMM0315;
301852	-	TSS_014351	1000	250	0	0	I	within gene(s) PMM0315;
301897	-	TSS_014355	1000	592	0	12	I	within gene(s) PMM0315;
301915	-	TSS_014359	1000	1235	0	13	I	within gene(s) PMM0315;
301916	+	TSS_002211	1000	1234	0	3	Ai	antisense to gene(s) PMM0315;
301936	-	TSS_014364	1000	535	0	0	I	within gene(s) PMM0315;
301937	+	TSS_002212	1000	934	0	0	Ai	antisense to gene(s) PMM0315;
301945	-	TSS_014365	1000	260	0	0	I	within gene(s) PMM0315;
301964	+	TSS_002214	1000	213	0	0	Ai	antisense to gene(s) PMM0315;
301981	-	TSS_014366	1000	255	0	0	I	within gene(s) PMM0315;
302004	-	TSS_014368	1000	504	0	9	I	within gene(s) PMM0315;
302023	-	TSS_014373	1000	1487	0	2	I	within gene(s) PMM0315;
302044	-	TSS_014374	1000	215	0	0	I	within gene(s) PMM0315;
302062	-	TSS_014377	1000	2764	0	3	I	within gene(s) PMM0315;
302080	-	TSS_014378	1000	496	0	0	I	within gene(s) PMM0315;
302091	-	TSS_014379	1000	216	0	0	I	within gene(s) PMM0315;
302107	-	TSS_014383	1000	2117	0	15	I	within gene(s) PMM0315;
302114	+	TSS_002216	1000	281	0	0	Ai	antisense to gene(s) PMM0315;
302122	+	TSS_002218	1000	1237	0	1	Ai	antisense to gene(s) PMM0315;
302134	-	TSS_014387	1000	439	0	11	I	within gene(s) PMM0315;
302152	-	TSS_014391	1000	238	0	0	I	within gene(s) PMM0315;
302153	+	TSS_002219	1000	128	0	0	Ai	antisense to gene(s) PMM0315;
302182	-	TSS_014397	1000	3993	0	40	I	within gene(s) PMM0315;
302221	-	TSS_014410	1000	1888	0	1	I	within gene(s) PMM0315;
302233	-	TSS_014411	1000	315	0	0	I	within gene(s) PMM0315;
302244	-	TSS_014412	1000	372	0	4	I	within gene(s) PMM0315;
302272	-	TSS_014414	1000	2063	0	0	I	within gene(s) PMM0315;
302293	-	TSS_014415	1000	564	0	0	I	within gene(s) PMM0315;
302320	+	TSS_002222	1000	103	0	0	Ai	antisense to gene(s) PMM0315;
302327	+	TSS_002223	1000	263	0	0	Ai	antisense to gene(s) PMM0315;
302329	-	TSS_014418	1000	991	0	3	I	within gene(s) PMM0315;
302338	-	TSS_014419	1000	779	0	9	I	within gene(s) PMM0315;
302364	-	TSS_014423	1000	187	0	0	I	within gene(s) PMM0315;
302368	+	TSS_002224	1000	150	0	0	Ai	antisense to gene(s) PMM0315;
302385	-	TSS_014425	1000	454	0	4	I	within gene(s) PMM0315;
302398	-	TSS_014428	1000	177	0	0	I	within gene(s) PMM0315;
302418	-	TSS_014429	1000	165	0	0	I	within gene(s) PMM0315;
302449	-	TSS_014430	1000	177	0	0	I	within gene(s) PMM0315;
302461	-	TSS_014431	1000	1714	0	0	I	within gene(s) PMM0315;
302479	-	TSS_014433	1000	238	0	1	I	within gene(s) PMM0315;

302497	-	TSS_014435	1000	12881	0	2	I	within gene(s) PMM0315;
302527	-	TSS_014438	1000	252	0	3	I	within gene(s) PMM0315;
302539	-	TSS_014440	1000	532	0	4	I	within gene(s) PMM0315;
302557	-	TSS_014442	1000	518	0	0	I	within gene(s) PMM0315;
302590	-	TSS_014444	1000	3404	0	2	P	3nt upstream of gene PMM0315;
302980	+	TSS_002230	1000	174	0	6	I	within gene(s) PMM0316;
303250	-	TSS_014450	1000	146	0	6	O	-
303256	+	TSS_002235	1000	1872	0	18	P	24nt upstream of gene PMM0317;
305403	-	TSS_014460	1000	281	0	0	I	within gene(s) PMM0320;
305442	-	TSS_014461	1000	202	0	0	I	within gene(s) PMM0320;
305463	-	TSS_014464	1000	164	0	5	I	within gene(s) PMM0320;
305727	-	TSS_014468	1000	455	0	3	IP	within gene(s) PMM0321; 174nt upstream of gene PMM0320;
306386	-	TSS_014473	1000	927	0	2	P	15nt upstream of gene PMM0321;
308744	-	TSS_014482	1000	104	0	0	I	within gene(s) PMM0324;
308747	+	TSS_002255	1000	205	0	0	Ai	antisense to gene(s) PMM0324;
308758	+	TSS_002257	1000	12029	0	3	Ai	antisense to gene(s) PMM0324;
308935	+	TSS_002262	1000	103	0	0	Ai	antisense to gene(s) PMM0324;
309008	+	TSS_002264	1000	431	0	0	Ai	antisense to gene(s) PMM0324;
309261	-	TSS_014494	1000	101	0	9	I	within gene(s) PMM0324;
309313	+	TSS_002268	1000	103	0	7	Ai	antisense to gene(s) PMM0324;
309678	-	TSS_014511	1000	140	0	0	I	within gene(s) PMM0324;
309757	-	TSS_014512	1000	280	0	0	P	22nt upstream of gene PMM0324;
309902	-	TSS_014513	1000	734	0	0	PAi	167nt upstream of gene PMM0324; antisense to gene(s) PMM0325;
309920	+	TSS_002273	1000	139	0	3	I	within gene(s) PMM0325;
309929	-	TSS_014515	1000	7467	0	1	PAi	194nt upstream of gene PMM0324; antisense to gene(s) PMM0325;
309975	-	TSS_014516	1000	160	0	4	PAi	240nt upstream of gene PMM0324; antisense to gene(s) PMM0325;
310002	+	TSS_002278	1000	152	0	15	I	within gene(s) PMM0325;
310023	+	TSS_002281	1000	684	0	0	I	within gene(s) PMM0325;
310049	+	TSS_002282	1000	127	0	4	I	within gene(s) PMM0325;
310070	+	TSS_002286	1000	122	0	6	I	within gene(s) PMM0325;
310100	+	TSS_002287	1000	266	0	0	I	within gene(s) PMM0325;
310271	+	TSS_002299	1000	527	0	6	IP	within gene(s) PMM0325; 234nt upstream of gene PMM0326;
310445	+	TSS_002308	1000	158	0	12	IP	within gene(s) PMM0325; 60nt upstream of gene PMM0326;
310480	+	TSS_002309	1000	470	0	2	P	25nt upstream of gene PMM0326;
310538	+	TSS_002311	1000	286	0	0	I	within gene(s) PMM0326;
310550	+	TSS_002312	1000	106	0	0	I	within gene(s) PMM0326;
310559	+	TSS_002314	1000	319	0	17	I	within gene(s) PMM0326;
310592	+	TSS_002325	1000	568	0	18	I	within gene(s) PMM0326;
310622	+	TSS_002328	1000	300	0	3	I	within gene(s) PMM0326;
310640	+	TSS_002330	1000	177	0	3	I	within gene(s) PMM0326;
310655	+	TSS_002332	1000	175	0	1	I	within gene(s) PMM0326;
310676	+	TSS_002338	1000	106	0	12	I	within gene(s) PMM0326;
310715	+	TSS_002340	1000	113	0	0	I	within gene(s) PMM0326;
310880	+	TSS_002347	1000	312	0	12	I	within gene(s) PMM0326;
311306	+	TSS_002354	1000	214	0	0	Ai	antisense to gene(s) PMM0327;
311725	+	TSS_002356	1000	138	0	0	Ai	antisense to gene(s) PMM0327;
312157	-	TSS_014554	1000	172	0	0	I	within gene(s) PMM0327;
312178	-	TSS_014556	1000	103	0	20	I	within gene(s) PMM0327;
312446	-	TSS_014568	1000	2192	0	3	P	16nt upstream of gene PMM0327;
312554	+	TSS_002359	1000	1555	0	0	O	-
312873	-	TSS_014570	1000	710	0	1	O	-
313132	-	TSS_014578	1000	168	0	0	O	-
313374	-	TSS_014581	1000	297	0	1	O	-
313535	-	TSS_014584	1000	407	0	6	O	-
314529	-	TSS_014597	1000	419	0	1	O	-
314912	+	TSS_002449	1000	272	0	0	O	-
314959	+	TSS_002450	1000	323	0	0	O	-
315077	+	TSS_002462	1000	10220	0	27	O	-
315908	-	TSS_014609	1000	197	0	1	O	-
316320	-	TSS_014614	1000	105	0	1	O	-
318987	+	TSS_002591	1000	102	0	0	Ai	antisense to gene(s) PMM0328;
319201	-	TSS_014641	1000	112	0	3	IP	within gene(s) PMM0329; 148nt upstream of gene PMM0328;
319230	-	TSS_014644	1000	134	0	14	IP	within gene(s) PMM0329; 177nt upstream of gene PMM0328;
319267	-	TSS_014649	1000	270	0	0	P	0nt upstream of gene PMM0329;
319282	-	TSS_014651	1000	40065	0	7	P	15nt upstream of gene PMM0329;
322362	+	TSS_002597	1000	253	0	0	P	0nt upstream of gene PMM0333;
323620	-	TSS_014665	1000	765	0	5	P	21nt upstream of gene PMM0334;
324251	-	TSS_014668	1000	19240	0	4	O	-
324470	-	TSS_014673	1000	1416	0	3	O	-
324964	+	TSS_002611	1000	1325	0	1	I	within gene(s) PMM0335;
325272	-	TSS_014690	1000	231	0	24	I	within gene(s) PMM0336;
325302	-	TSS_014697	1000	173	0	45	I	within gene(s) PMM0336;
325368	-	TSS_014711	1000	183	0	33	I	within gene(s) PMM0336;
325410	-	TSS_014717	1000	180	0	6	I	within gene(s) PMM0336;
325485	-	TSS_014725	1000	110	0	18	I	within gene(s) PMM0336;
325506	-	TSS_014730	1000	228	0	10	I	within gene(s) PMM0336;
325557	-	TSS_014734	1000	27733	0	3	P	15nt upstream of gene PMM0336;
326449	-	TSS_014742	1000	345	0	1	P	15nt upstream of gene PMM0337;
326712	+	TSS_002625	1000	1119	0	2	O	-

327570	-	TSS_014758	1000	6136	0	2	O	-
327681	+	TSS_002628	1000	329	0	1	Ai	antisense to gene(s) PMM0339;
328681	-	TSS_014764	1000	194	0	1	I	within gene(s) PMM0339;
329348	-	TSS_014767	1000	548	0	0	P	157nt upstream of gene PMM0339;
330221	-	TSS_014769	1000	258	0	0	P	15nt upstream of gene PMM0341;
330306	+	TSS_002637	1000	1096	0	1	P	35nt upstream of gene PMM0342;
330978	-	TSS_014771	1000	346	0	1	O	-
331018	-	TSS_014773	1000	368	0	0	O	-
332301	+	TSS_002642	1000	147	0	6	P	16nt upstream of gene PMM0345;
332821	+	TSS_002648	1000	2219	0	3	I	within gene(s) PMM0346;
332939	-	TSS_014781	1000	146	0	1	Ai	antisense to gene(s) PMM0346;
332983	-	TSS_014782	1000	138	0	0	Ai	antisense to gene(s) PMM0346;
333569	-	TSS_014786	1000	290	0	0	P	108nt upstream of gene PMM0347;
333947	-	TSS_014789	1000	63918	0	2	P	20nt upstream of gene PMM0348;
339021	+	TSS_002660	1000	311	0	1	P	18nt upstream of gene PMM0355;
339316	-	TSS_014798	1000	364	0	0	Ai	antisense to gene(s) PMM0355;
339338	-	TSS_014799	1000	102	0	0	Ai	antisense to gene(s) PMM0355;
340326	+	TSS_002664	1000	386	0	2	I	within gene(s) PMM0356;
345398	+	TSS_002677	1000	117	0	3	P	22nt upstream of gene PMM0363;
346829	+	TSS_002680	1000	171	0	3	O	-
347280	+	TSS_002683	1000	10178	0	3	P	16nt upstream of gene PMM0364;
348066	-	TSS_014820	1000	3096	0	2	P	21nt upstream of gene PMM0365;
348206	+	TSS_002695	1000	590	0	5	P	17nt upstream of gene PMM0366;
348397	+	TSS_002700	1000	224	0	1	I	within gene(s) PMM0366;
348935	+	TSS_002704	1000	367	0	1	P	33nt upstream of gene PMM0367;
349333	-	TSS_014827	1000	193	0	2	Ai	antisense to gene(s) PMM0367;
349634	-	TSS_014829	1000	230	0	0	I	within gene(s) PMM0368;
349709	-	TSS_014830	1000	995	0	1	P	18nt upstream of gene PMM0368;
350069	-	TSS_014833	1000	458	0	0	O	-
350388	+	TSS_002708	1000	281	0	1	O	-
350523	-	TSS_014836	1000	42611	0	4	O	-
351169	-	TSS_014841	1000	17808	0	6	O	-
352103	-	TSS_014849	1000	492	0	2	O	-
352188	+	TSS_002716	1000	2285	0	2	P	7nt upstream of gene PMM0369;
352957	+	TSS_002718	1000	719	0	0	P	18nt upstream of gene PMM0370;
353380	+	TSS_002743	1000	146	0	10	I	within gene(s) PMM0370;
353529	-	TSS_014853	1000	231	0	0	Ai	antisense to gene(s) PMM0370;
353732	-	TSS_014854	1000	369	0	1	Ai	antisense to gene(s) PMM0370;
353890	+	TSS_002773	1000	327	0	14	I	within gene(s) PMM0370;
353992	+	TSS_002784	1000	149	0	4	I	within gene(s) PMM0370;
354088	+	TSS_002792	1000	253	0	9	I	within gene(s) PMM0370;
354139	+	TSS_002799	1000	281	0	18	I	within gene(s) PMM0370;
354178	+	TSS_002804	1000	435	0	1	I	within gene(s) PMM0370;
354259	+	TSS_002811	1000	123	0	4	I	within gene(s) PMM0370;
354439	-	TSS_014861	1000	133	0	1	Ai	antisense to gene(s) PMM0370;
354451	+	TSS_002830	1000	290	0	63	IP	within gene(s) PMM0370; 240nt upstream of gene PMM0371;
355412	-	TSS_014865	1000	235	0	0	Ai	antisense to gene(s) PMM0371;
356060	-	TSS_014866	1000	162	0	0	Ai	antisense to gene(s) PMM0372;
356326	+	TSS_002838	1000	133	0	1	IP	within gene(s) PMM0372; 51nt upstream of gene PMM0373;
358183	-	TSS_014873	1000	1142	0	2	P	16nt upstream of gene PMM0377;
358512	-	TSS_014878	1000	14280	0	2	P	22nt upstream of gene PMM0378;
358753	-	TSS_014881	1000	4805	0	2	O	-
359627	-	TSS_014883	1000	606	0	0	O	-
359991	-	TSS_014887	1000	1644	0	2	P	20nt upstream of gene PMM0379;
361648	+	TSS_002848	1000	212	0	0	O	-
361663	+	TSS_002849	1000	370	0	0	O	-
362873	-	TSS_014893	1000	1115	0	2	P	140nt upstream of gene PMM0383;
366442	-	TSS_014908	1000	199	0	2	O	-
366522	-	TSS_014910	1000	550	0	0	O	-
367091	-	TSS_014913	1000	131	0	1	I	within gene(s) PMM0386;
367935	-	TSS_014915	1000	292	0	1	O	-
368274	+	TSS_002869	1000	500	0	2	O	-
368514	-	TSS_014919	1000	288	0	4	O	-
370969	+	TSS_002878	1000	2033	0	1	P	24nt upstream of gene PMM0391;
374270	+	TSS_002886	1000	574	0	0	P	97nt upstream of gene PMM0395;
374286	+	TSS_002888	1000	16296	0	2	P	81nt upstream of gene PMM0395;
374685	+	TSS_002913	1000	115	0	0	I	within gene(s) PMM0395;
374701	+	TSS_002916	1000	256	0	9	IP	within gene(s) PMM0395; 249nt upstream of gene PMM0396;
374727	+	TSS_002923	1000	214	0	22	IP	within gene(s) PMM0395; 223nt upstream of gene PMM0396;
374754	+	TSS_002928	1000	188	0	10	IP	within gene(s) PMM0395; 196nt upstream of gene PMM0396;
377675	-	TSS_014946	1000	201	0	1	I	within gene(s) PMM0398;
379061	-	TSS_014950	1000	133	0	2	P	24nt upstream of gene PMM0400;
380710	-	TSS_014955	1000	132	0	0	Ai	antisense to gene(s) PMM0402;
381726	+	TSS_002957	1000	2507	0	2	P	14nt upstream of gene PMM0403;
383390	+	TSS_002967	1000	156	0	3	O	-
383585	+	TSS_002969	1000	7185	0	4	P	85nt upstream of gene PMM0405;
383856	+	TSS_002991	1000	118	0	4	I	within gene(s) PMM0405;
383907	+	TSS_002996	1000	130	0	0	I	within gene(s) PMM0405;
383961	+	TSS_003003	1000	151	0	24	I	within gene(s) PMM0405;

384035	-	TSS_014959	1000	524	0	1	Ai	antisense to gene(s) PMM0405;
384144	+	TSS_003021	1000	167	0	22	I	within gene(s) PMM0405;
384162	+	TSS_003026	1000	229	0	3	I	within gene(s) PMM0405;
384195	+	TSS_003031	1000	278	0	11	I	within gene(s) PMM0405;
384519	+	TSS_003048	1000	485	0	15	I	within gene(s) PMM0405;
384720	+	TSS_003070	1000	576	0	15	I	within gene(s) PMM0405;
385716	+	TSS_003083	1000	476	0	0	I	within gene(s) PMM0406;
386282	+	TSS_003086	1000	336	0	0	Ai	antisense to gene(s) PMM0407;
386350	-	TSS_014967	1000	113	0	15	I	within gene(s) PMM0407;
386383	-	TSS_014973	1000	403	0	18	I	within gene(s) PMM0407;
386391	+	TSS_003089	1000	101	0	0	Ai	antisense to gene(s) PMM0407;
386402	+	TSS_003090	1000	233	0	0	Ai	antisense to gene(s) PMM0407;
386404	-	TSS_014977	1000	321	0	0	I	within gene(s) PMM0407;
386413	-	TSS_014978	1000	129	0	0	I	within gene(s) PMM0407;
386467	-	TSS_014989	1000	1475	0	39	I	within gene(s) PMM0407;
386503	-	TSS_014998	1000	744	0	24	I	within gene(s) PMM0407;
386514	+	TSS_003093	1000	499	0	4	Ai	antisense to gene(s) PMM0407;
386530	-	TSS_015001	1000	297	0	3	I	within gene(s) PMM0407;
386539	-	TSS_015002	1000	288	0	0	I	within gene(s) PMM0407;
386551	-	TSS_015003	1000	547	0	0	I	within gene(s) PMM0407;
386598	-	TSS_015004	1000	213	0	1	I	within gene(s) PMM0407;
386603	+	TSS_003094	1000	2388	0	1	Ai	antisense to gene(s) PMM0407;
386611	-	TSS_015008	1000	722	0	48	I	within gene(s) PMM0407;
386615	+	TSS_003096	1000	185	0	0	Ai	antisense to gene(s) PMM0407;
386674	-	TSS_015022	1000	501	0	12	I	within gene(s) PMM0407;
386701	-	TSS_015027	1000	1492	0	36	I	within gene(s) PMM0407;
386764	-	TSS_015044	1000	776	0	33	I	within gene(s) PMM0407;
386777	+	TSS_003099	1000	280	0	7	Ai	antisense to gene(s) PMM0407;
386789	+	TSS_003100	1000	124	0	0	Ai	antisense to gene(s) PMM0407;
386815	-	TSS_015056	1000	1476	0	33	I	within gene(s) PMM0407;
386824	-	TSS_015057	1000	339	0	12	I	within gene(s) PMM0407;
386845	-	TSS_015060	1000	210	0	0	I	within gene(s) PMM0407;
386860	-	TSS_015061	1000	155	2.40E-07	0	I	within gene(s) PMM0407;
386861	+	TSS_003101	1000	385	0	13	Ai	antisense to gene(s) PMM0407;
386881	-	TSS_015066	1000	964	0	12	I	within gene(s) PMM0407;
386908	-	TSS_015072	1000	306	0	12	I	within gene(s) PMM0407;
386920	-	TSS_015075	1000	340	0	11	I	within gene(s) PMM0407;
386950	-	TSS_015083	1000	363	0	30	I	within gene(s) PMM0407;
386989	-	TSS_015093	1000	618	0	22	I	within gene(s) PMM0407;
387013	-	TSS_015096	1000	180	0	0	I	within gene(s) PMM0407;
387022	-	TSS_015097	1000	274	0	0	I	within gene(s) PMM0407;
387042	-	TSS_015101	1000	990	0	18	I	within gene(s) PMM0407;
387073	-	TSS_015107	1000	275	0	9	I	within gene(s) PMM0407;
387097	-	TSS_015110	1000	355	0	0	I	within gene(s) PMM0407;
387112	-	TSS_015112	1000	615	0	3	I	within gene(s) PMM0407;
387133	-	TSS_015113	1000	438	0	6	I	within gene(s) PMM0407;
387181	-	TSS_015115	1000	211	0	3	P	24nt upstream of gene PMM0407;
390342	-	TSS_015125	1000	126	0	4	I	within gene(s) PMM0410;
390555	-	TSS_015132	1000	461	0	1	I	within gene(s) PMM0410;
390616	-	TSS_015133	1000	535	0	1	P	55nt upstream of gene PMM0410;
390631	+	TSS_003117	1000	169	0	3	P	26nt upstream of gene PMM0411;
392813	+	TSS_003122	1000	428	0	2	P	15nt upstream of gene PMM0414;
395057	+	TSS_003125	1000	115	0	0	Ai	antisense to gene(s) PMM0416;
395539	-	TSS_015144	1000	1564	0	2	P	16nt upstream of gene PMM0416;
398147	+	TSS_003134	1000	878	0	2	P	32nt upstream of gene PMM0420;
398719	+	TSS_003137	1000	240	0	0	I	within gene(s) PMM0420;
401612	-	TSS_015174	1000	529	0	0	P	18nt upstream of gene PMM0422;
401747	+	TSS_003152	1000	5391	0	3	O	-
401864	-	TSS_015178	1000	234	0	1	O	-
402007	-	TSS_015179	1000	163	0	0	O	-
405550	+	TSS_003160	1000	150	0	1	I	within gene(s) PMM0426;
409230	-	TSS_015202	1000	254	0	3	P	20nt upstream of gene PMM0429;
409244	+	TSS_003170	1000	110	0	0	P	26nt upstream of gene PMM0430;
409562	-	TSS_015204	1000	231	0	0	Ai	antisense to gene(s) PMM0430;
412935	+	TSS_003178	1000	192	0	6	Ai	antisense to gene(s) PMM0435;
413484	+	TSS_003181	1000	407	0	3	Ai	antisense to gene(s) PMM0435;
414354	+	TSS_003187	1000	111	0	0	P	130nt upstream of gene PMM0436;
414359	-	TSS_015235	1000	855	0	0	P	44nt upstream of gene PMM0435;
414382	+	TSS_003188	1000	677	0	2	P	102nt upstream of gene PMM0436;
417856	+	TSS_003241	1000	164	0	0	I	within gene(s) PMM0438;
419521	-	TSS_015250	1000	296	0	1	Ai	antisense to gene(s) PMM0440;
422795	-	TSS_015261	1000	777	0	6	P	16nt upstream of gene PMM0443;
423632	-	TSS_015273	1000	184	0	12	IP	within gene(s) PMM0445; 153nt upstream of gene PMM0444;
423797	-	TSS_015279	1000	130	0	6	I	within gene(s) PMM0445;
423830	-	TSS_015282	1000	249	0	0	I	within gene(s) PMM0445;
423865	+	TSS_003255	1000	202	0	0	Ai	antisense to gene(s) PMM0445;
423944	-	TSS_015286	1000	256	0	3	I	within gene(s) PMM0445;
424676	+	TSS_003263	1000	182	0	0	Ai	antisense to gene(s) PMM0445;
424706	-	TSS_015303	1000	344	0	3	I	within gene(s) PMM0445;

424756	-	TSS_015306	1000	161	0	0	I	within gene(s) PMM0445;
424769	-	TSS_015308	1000	133	0	1	I	within gene(s) PMM0445;
424790	-	TSS_015311	1000	333	0	16	I	within gene(s) PMM0445;
424880	-	TSS_015322	1000	795	0	6	I	within gene(s) PMM0445;
425395	-	TSS_015338	1000	378	0	0	I	within gene(s) PMM0446;
425512	-	TSS_015340	1000	108	0	2	I	within gene(s) PMM0446;
426162	+	TSS_003268	1000	769	0	1	P	8nt upstream of gene PMM0447;
427201	+	TSS_003284	1000	168	0	18	I	within gene(s) PMM0448;
427279	+	TSS_003287	1000	181	0	2	I	within gene(s) PMM0448;
427561	+	TSS_003295	1000	170	0	3	I	within gene(s) PMM0448;
427588	+	TSS_003296	1000	218	0	4	I	within gene(s) PMM0448;
427696	+	TSS_003302	1000	175	0	0	I	within gene(s) PMM0448;
427762	+	TSS_003304	1000	102	0	0	I	within gene(s) PMM0448;
429348	+	TSS_003311	1000	127	0	1	I	within gene(s) PMM0450;
429366	+	TSS_003313	1000	131	0	3	I	within gene(s) PMM0450;
429988	-	TSS_015366	1000	115	0	1	Ai	antisense to gene(s) PMM0450;
430126	+	TSS_003318	1000	227	0	0	I	within gene(s) PMM0451;
430701	+	TSS_003331	1000	159	0	0	Ai	antisense to gene(s) PMM0452;
431111	+	TSS_003335	1000	108	0	0	Ai	antisense to gene(s) PMM0452;
431182	-	TSS_015382	1000	263	0	12	I	within gene(s) PMM0452;
431251	-	TSS_015393	1000	189	0	1	I	within gene(s) PMM0452;
431412	+	TSS_003342	1000	189	0	1	Ai	antisense to gene(s) PMM0452;
431485	-	TSS_015420	1000	202	0	48	I	within gene(s) PMM0452;
431803	-	TSS_015441	1000	153	0	3	I	within gene(s) PMM0452;
431848	-	TSS_015444	1000	147	0	21	I	within gene(s) PMM0452;
431908	-	TSS_015458	1000	102	0	13	I	within gene(s) PMM0452;
431932	-	TSS_015461	1000	133	0	0	I	within gene(s) PMM0452;
431969	+	TSS_003346	1000	246	0	1	Ai	antisense to gene(s) PMM0452;
431983	-	TSS_015465	1000	122	0	12	I	within gene(s) PMM0452;
432058	-	TSS_015478	1000	285	0	9	I	within gene(s) PMM0452;
432085	-	TSS_015482	1000	103	0	0	I	within gene(s) PMM0452;
432121	-	TSS_015487	1000	101	0	24	I	within gene(s) PMM0452;
432175	-	TSS_015496	1000	122	0	1	I	within gene(s) PMM0452;
432210	-	TSS_015499	1000	218	0	2	I	within gene(s) PMM0452;
432317	-	TSS_015501	1000	4064	0	3	P	16nt upstream of gene PMM0452;
432436	+	TSS_003350	1000	157	0	0	O	-
433380	-	TSS_015515	1000	128	0	0	P	16nt upstream of gene PMM0453;
433437	+	TSS_003353	1000	463	0	1	P	21nt upstream of gene PMM0454;
433825	-	TSS_015516	1000	154	0	0	Ai	antisense to gene(s) PMM0454;
433897	+	TSS_003357	1000	245	0	2	I	within gene(s) PMM0454;
434016	-	TSS_015517	1000	142	0	0	Ai	antisense to gene(s) PMM0454;
435917	-	TSS_015522	1000	289	0	0	P	19nt upstream of gene PMM0456;
437733	+	TSS_003368	1000	117	0	0	Ai	antisense to gene(s) PMM0458;
440026	-	TSS_015530	1000	101	0	0	IP	within gene(s) PMM0461; 221nt upstream of gene PMM0460;
440050	-	TSS_015531	1000	247	0	15	IP	within gene(s) PMM0461; 245nt upstream of gene PMM0460;
440074	-	TSS_015536	1000	149	0	12	I	within gene(s) PMM0461;
440245	-	TSS_015543	1000	193	0	18	I	within gene(s) PMM0461;
440290	-	TSS_015549	1000	152	0	12	I	within gene(s) PMM0461;
440581	-	TSS_015578	1000	142	0	0	I	within gene(s) PMM0461;
440695	-	TSS_015590	1000	130	0	1	I	within gene(s) PMM0461;
440885	-	TSS_015602	1000	193	0	9	IP	within gene(s) PMM0462; 115nt upstream of gene PMM0461;
441035	+	TSS_003385	1000	163	0	6	Ai	antisense to gene(s) PMM0462;
441185	-	TSS_015628	1000	176	0	2	I	within gene(s) PMM0462;
441340	-	TSS_015633	1000	1201	0	3	P	29nt upstream of gene PMM0462;
442840	-	TSS_015647	1000	1913	0	6	P	17nt upstream of gene PMM0465;
444471	+	TSS_003391	1000	119	0	0	PAi	157nt upstream of gene PMM0467; antisense to gene(s) PMM0466;
445213	+	TSS_003396	1000	417	0	1	Ai	antisense to gene(s) PMM0468;
445437	+	TSS_003398	1000	1014	0	1	Ai	antisense to gene(s) PMM0469;
445454	-	TSS_015663	1000	152	0	48	IP	within gene(s) PMM0469; 120nt upstream of gene PMM0468;
445514	-	TSS_015674	1000	153	0	3	IP	within gene(s) PMM0469; 180nt upstream of gene PMM0468;
445523	-	TSS_015675	1000	150	0	0	IP	within gene(s) PMM0469; 189nt upstream of gene PMM0468;
445547	-	TSS_015676	1000	1874	0	12	IP	within gene(s) PMM0469; 213nt upstream of gene PMM0468;
445571	-	TSS_015680	1000	951	0	15	IP	within gene(s) PMM0469; 237nt upstream of gene PMM0468;
445575	+	TSS_003400	1000	519	0	0	Ai	antisense to gene(s) PMM0469;
445601	-	TSS_015688	1000	1052	0	12	I	within gene(s) PMM0469;
445625	-	TSS_015694	1000	349	0	27	I	within gene(s) PMM0469;
445658	-	TSS_015701	1000	425	0	3	I	within gene(s) PMM0469;
445694	-	TSS_015710	1000	687	0	39	I	within gene(s) PMM0469;
445754	-	TSS_015718	1000	128	0	6	I	within gene(s) PMM0469;
445796	-	TSS_015722	1000	354	0	1	I	within gene(s) PMM0469;
445823	-	TSS_015724	1000	168	0	0	I	within gene(s) PMM0469;
445832	-	TSS_015726	1000	107	0	1	I	within gene(s) PMM0469;
445961	-	TSS_015730	1000	35375	0	4	P	42nt upstream of gene PMM0469;
445980	+	TSS_003402	1000	233	0	1	P	14nt upstream of gene PMM0470;
446547	+	TSS_003409	1000	130	0	0	I	within gene(s) PMM0470;
446903	+	TSS_003415	1000	323	0	0	IP	within gene(s) PMM0470; 167nt upstream of gene PMM0471;
447381	+	TSS_003419	1000	1090	0	4	P	29nt upstream of gene PMM0472;
450442	+	TSS_003425	1000	163	0	2	Ai	antisense to gene(s) PMM0474;
450594	-	TSS_015752	1000	159	0	0	P	113nt upstream of gene PMM0474;

450629	-	TSS_015754	1000	1372	0	1	P	148nt upstream of gene PMM0474;
450680	-	TSS_015756	1000	453	0	0	IP	within gene(s) PMM0475; 199nt upstream of gene PMM0474;
450696	-	TSS_015757	1000	170	0	0	IP	within gene(s) PMM0475; 215nt upstream of gene PMM0474;
450716	-	TSS_015759	1000	5264	0	3	IP	within gene(s) PMM0475; 235nt upstream of gene PMM0474;
450872	+	TSS_003429	1000	322	0	2	Ai	antisense to gene(s) PMM0475;
450998	+	TSS_003430	1000	232	0	1	Ai	antisense to gene(s) PMM0475;
451103	-	TSS_015769	1000	123	0	0	I	within gene(s) PMM0475;
451134	-	TSS_015776	1000	5175	0	8	P	4nt upstream of gene PMM0475;
451442	+	TSS_003436	1000	339	0	4	PAi	101nt upstream of gene PMM0477; antisense to gene(s) PMM0476;
451486	+	TSS_003438	1000	1598	0	0	P	57nt upstream of gene PMM0477;
451522	-	TSS_015780	1000	276	0	0	P	69nt upstream of gene PMM0476;
451687	+	TSS_003447	1000	167	0	1	I	within gene(s) PMM0477;
451719	-	TSS_015782	1000	165	0	2	Ai	antisense to gene(s) PMM0477;
451771	+	TSS_003454	1000	109	0	3	I	within gene(s) PMM0477;
451802	+	TSS_003460	1000	164	0	18	I	within gene(s) PMM0477;
451813	+	TSS_003462	1000	272	0	0	I	within gene(s) PMM0477;
451849	+	TSS_003466	1000	111	0	27	I	within gene(s) PMM0477;
451908	-	TSS_015791	1000	174	0	0	Ai	antisense to gene(s) PMM0477;
451913	+	TSS_003477	1000	630	0	12	I	within gene(s) PMM0477;
451936	+	TSS_003482	1000	254	0	1	I	within gene(s) PMM0477;
452077	+	TSS_003490	1000	162	0	0	I	within gene(s) PMM0477;
452104	+	TSS_003496	1000	267	0	12	I	within gene(s) PMM0477;
452132	+	TSS_003499	1000	9103	0	2	I	within gene(s) PMM0477;
453247	+	TSS_003505	1000	415	0	2	P	22nt upstream of gene PMM0479;
453732	-	TSS_015806	1000	223	0	0	Ai	antisense to gene(s) PMM0479;
453884	+	TSS_003522	1000	111	0	4	I	within gene(s) PMM0479;
454387	+	TSS_003528	1000	1038	0	3	P	17nt upstream of gene PMM0480;
455492	+	TSS_003534	1000	107	0	0	O	-
455674	-	TSS_015815	1000	127	0	3	O	-
455989	+	TSS_003549	1000	124	0	0	I	within gene(s) PMM0482;
456019	+	TSS_003551	1000	111	0	0	I	within gene(s) PMM0482;
456144	-	TSS_015821	1000	474	0	1	Ai	antisense to gene(s) PMM0482;
456205	+	TSS_003565	1000	242	0	9	I	within gene(s) PMM0482;
456226	+	TSS_003567	1000	186	0	24	I	within gene(s) PMM0482;
456319	+	TSS_003586	1000	408	0	24	I	within gene(s) PMM0482;
456568	+	TSS_003593	1000	195	0	1	I	within gene(s) PMM0482;
456940	+	TSS_003598	1000	247	0	3	Ai	antisense to gene(s) PMM0483;
457405	+	TSS_003604	1000	717	0	2	Ai	antisense to gene(s) PMM0483;
457551	+	TSS_003606	1000	216	0	1	Ai	antisense to gene(s) PMM0483;
457975	-	TSS_015843	1000	1133	0	5	P	36nt upstream of gene PMM0483;
458013	-	TSS_015848	1000	281	0	2	P	74nt upstream of gene PMM0483;
459029	-	TSS_015851	1000	132	0	0	P	17nt upstream of gene PMM0484;
459043	+	TSS_003609	1000	110	0	0	P	42nt upstream of gene PMM0485;
465840	+	TSS_003624	1000	476	0	3	P	16nt upstream of gene PMM0493;
466192	+	TSS_003626	1000	630	0	1	I	within gene(s) PMM0493;
466610	+	TSS_003628	1000	123	0	0	I	within gene(s) PMM0493;
467553	+	TSS_003634	1000	331	0	2	I	within gene(s) PMM0494;
468497	-	TSS_015872	1000	578	0	2	I	within gene(s) PMM0495;
469000	-	TSS_015877	1000	273	0	0	P	32nt upstream of gene PMM0495;
469311	-	TSS_015884	1000	237	0	0	I	within gene(s) PMM0496;
469398	-	TSS_015896	1000	113	0	4	I	within gene(s) PMM0496;
469488	+	TSS_003655	1000	210	0	1	Ai	antisense to gene(s) PMM0496;
470267	-	TSS_015927	1000	2917	0	2	P	14nt upstream of gene PMM0496;
474859	-	TSS_015937	1000	302	0	0	IP	within gene(s) PMM0500; 37nt upstream of gene PMM0499;
475324	-	TSS_015943	1000	355	0	4	I	within gene(s) PMM0500;
475383	-	TSS_015946	1000	372	0	0	P	19nt upstream of gene PMM0500;
475534	-	TSS_015947	1000	212	0	0	PAi	170nt upstream of gene PMM0500; antisense to gene(s) PMM0501;
475685	+	TSS_003668	1000	794	0	10	Ai	antisense to gene(s) PMM0502;
475749	+	TSS_003674	1000	1996	0	3	Ai	antisense to gene(s) PMM0502;
476045	-	TSS_015955	1000	835	0	1	P	16nt upstream of gene PMM0502;
479542	-	TSS_015968	1000	525	0	2	P	26nt upstream of gene PMM0506;
479651	-	TSS_015969	1000	120	0	0	IP	within gene(s) PMM0507; 135nt upstream of gene PMM0506;
480046	-	TSS_015974	1000	102	0	1	P	11nt upstream of gene PMM0507;
480066	-	TSS_015976	1000	197	0	3	IP	within gene(s) PMM0508; 31nt upstream of gene PMM0507;
481882	-	TSS_015993	1000	759	0	1	P	18nt upstream of gene PMM0508;
482442	+	TSS_003692	1000	534	0	1	P	36nt upstream of gene PMM0510;
483044	-	TSS_015997	1000	302	0	0	Ai	antisense to gene(s) PMM0511;
483869	+	TSS_003698	1000	267	0	3	I	within gene(s) PMM0513;
486912	-	TSS_016007	1000	564	0	6	P	21nt upstream of gene PMM0515;
487573	+	TSS_003706	1000	112	0	0	I	within gene(s) PMM0516;
490431	+	TSS_003711	1000	135	0	2	I	within gene(s) PMM0518;
490807	+	TSS_003714	1000	103	0	6	I	within gene(s) PMM0519;
490882	+	TSS_003723	1000	129	0	21	I	within gene(s) PMM0519;
491260	+	TSS_003750	1000	473	0	60	I	within gene(s) PMM0519;
491487	-	TSS_016016	1000	117	0	6	Ai	antisense to gene(s) PMM0519;
493398	-	TSS_016028	1000	420	0	4	I	within gene(s) PMM0521;
493513	-	TSS_016031	1000	261	0	0	IP	within gene(s) PMM0522; 82nt upstream of gene PMM0521;
493630	+	TSS_003770	1000	228	0	3	Ai	antisense to gene(s) PMM0522;
493755	+	TSS_003772	1000	177	0	0	Ai	antisense to gene(s) PMM0522;

493996	-	TSS_016037	1000	106	0	3	I	within gene(s) PMM0522;
494163	-	TSS_016039	1000	768	0	0	I	within gene(s) PMM0522;
494177	-	TSS_016040	1000	697	0	0	P	13nt upstream of gene PMM0522;
494529	+	TSS_003777	1000	140	0	0	Ai	antisense to gene(s) PMM0523;
495001	-	TSS_016044	1000	845	0	8	P	12nt upstream of gene PMM0523;
496211	+	TSS_003781	1000	1148	0	2	P	44nt upstream of gene PMM0525;
497254	+	TSS_003787	1000	213	0	6	I	within gene(s) PMM0525;
497550	+	TSS_003791	1000	2737	0	2	P	15nt upstream of gene PMM0526;
498172	+	TSS_003811	1000	101	0	4	I	within gene(s) PMM0526;
498247	+	TSS_003820	1000	117	0	12	I	within gene(s) PMM0526;
498643	+	TSS_003834	1000	183	0	0	I	within gene(s) PMM0526;
498840	+	TSS_003848	1000	239	0	4	I	within gene(s) PMM0526;
498873	+	TSS_003853	1000	190	0	4	I	within gene(s) PMM0526;
498964	-	TSS_016059	1000	239	0	2	Ai	antisense to gene(s) PMM0526;
499020	+	TSS_003860	1000	266	0	3	I	within gene(s) PMM0526;
499041	+	TSS_003864	1000	147	0	15	I	within gene(s) PMM0526;
499074	+	TSS_003873	1000	251	0	30	I	within gene(s) PMM0526;
502199	-	TSS_016078	1000	103	0	0	I	within gene(s) PMM0530;
502675	+	TSS_003891	1000	266	0	0	P	47nt upstream of gene PMM0531;
502682	-	TSS_016095	1000	1170	0	11	P	24nt upstream of gene PMM0530;
503597	+	TSS_003898	1000	1133	0	1	P	34nt upstream of gene PMM0532;
503618	+	TSS_003904	1000	143	0	10	P	13nt upstream of gene PMM0532;
503904	+	TSS_003912	1000	150	0	12	I	within gene(s) PMM0532;
504000	+	TSS_003918	1000	276	0	0	I	within gene(s) PMM0532;
504156	+	TSS_003933	1000	127	0	6	I	within gene(s) PMM0532;
504428	+	TSS_003938	1000	244	0	5	P	29nt upstream of gene PMM0533;
505257	-	TSS_016103	1000	124	0	0	Ai	antisense to gene(s) PMM0533;
505261	+	TSS_003942	1000	495	0	7	IP	within gene(s) PMM0533; 240nt upstream of gene PMM0534;
505757	+	TSS_003947	1000	174	0	0	I	within gene(s) PMM0534;
506152	+	TSS_003950	1000	131	0	0	I	within gene(s) PMM0534;
507310	+	TSS_003955	1000	458	0	0	P	62nt upstream of gene PMM0536;
507537	+	TSS_003963	1000	199	0	45	I	within gene(s) PMM0536;
507738	+	TSS_003980	1000	180	0	6	I	within gene(s) PMM0536;
507970	-	TSS_016106	1000	106	0	1	Ai	antisense to gene(s) PMM0536;
508770	-	TSS_016108	1000	132	0	0	I	within gene(s) PMM0537;
510847	+	TSS_003987	1000	554	0	2	P	19nt upstream of gene PMM0540;
512559	+	TSS_003996	1000	180	0	0	Ai	antisense to gene(s) PMM0543;
512746	-	TSS_016115	1000	197	0	13	I	within gene(s) PMM0543;
512853	+	TSS_003999	1000	3420	0	3	Ai	antisense to gene(s) PMM0543;
512911	-	TSS_016128	1000	284	0	3	I	within gene(s) PMM0543;
512922	-	TSS_016131	1000	101	0	1	I	within gene(s) PMM0543;
512932	-	TSS_016133	1000	162	0	3	I	within gene(s) PMM0543;
512959	-	TSS_016136	1000	1058	0	9	I	within gene(s) PMM0543;
513022	-	TSS_016140	1000	546	0	5	I	within gene(s) PMM0543;
513034	-	TSS_016143	1000	148	0	0	I	within gene(s) PMM0543;
513036	+	TSS_004003	1000	195	0	1	Ai	antisense to gene(s) PMM0543;
513063	+	TSS_004005	1000	176	0	0	Ai	antisense to gene(s) PMM0543;
513094	-	TSS_016147	1000	119	0	9	I	within gene(s) PMM0543;
513118	-	TSS_016152	1000	195	0	6	I	within gene(s) PMM0543;
513169	-	TSS_016159	1000	197	0	30	I	within gene(s) PMM0543;
513340	-	TSS_016180	1000	148	0	0	I	within gene(s) PMM0543;
513385	-	TSS_016182	1000	9602	0	5	P	24nt upstream of gene PMM0543;
513547	+	TSS_004010	1000	303	0	0	Ad	antisense to gene(s) PMM0544 (7nt downstream);
513696	-	TSS_016189	1000	125	0	9	I	within gene(s) PMM0544;
513757	+	TSS_004016	1000	142	0	1	Ai	antisense to gene(s) PMM0544;
513854	-	TSS_016197	1000	120	0	7	I	within gene(s) PMM0544;
513927	-	TSS_016202	1000	213	0	4	I	within gene(s) PMM0544;
514062	-	TSS_016216	1000	112	0	9	I	within gene(s) PMM0544;
514308	-	TSS_016240	1000	145	0	9	I	within gene(s) PMM0544;
514356	-	TSS_016250	1000	795	0	15	I	within gene(s) PMM0544;
514455	-	TSS_016255	1000	153	0	5	I	within gene(s) PMM0544;
514479	-	TSS_016258	1000	131	0	9	I	within gene(s) PMM0544;
514548	-	TSS_016269	1000	132	0	22	I	within gene(s) PMM0544;
514620	-	TSS_016277	1000	108	0	6	I	within gene(s) PMM0544;
514665	-	TSS_016284	1000	267	0	12	I	within gene(s) PMM0544;
514869	-	TSS_016298	1000	160	0	9	I	within gene(s) PMM0544;
514896	-	TSS_016302	1000	335	0	24	I	within gene(s) PMM0544;
514960	+	TSS_004027	1000	205	0	0	Ai	antisense to gene(s) PMM0544;
515006	+	TSS_004029	1000	360	0	4	Ai	antisense to gene(s) PMM0544;
515091	-	TSS_016319	1000	541	0	15	I	within gene(s) PMM0544;
515115	-	TSS_016324	1000	286	0	1	I	within gene(s) PMM0544;
515154	-	TSS_016327	1000	468	0	9	IP	within gene(s) PMM0545; 21nt upstream of gene PMM0544;
515174	-	TSS_016331	1000	218	0	13	IP	within gene(s) PMM0545; 41nt upstream of gene PMM0544;
515202	-	TSS_016338	1000	218	0	15	IP	within gene(s) PMM0545; 69nt upstream of gene PMM0544;
515274	-	TSS_016344	1000	104	0	3	IP	within gene(s) PMM0545; 141nt upstream of gene PMM0544;
515289	-	TSS_016346	1000	109	0	0	IP	within gene(s) PMM0545; 156nt upstream of gene PMM0544;
515316	-	TSS_016347	1000	220	0	0	IP	within gene(s) PMM0545; 183nt upstream of gene PMM0544;
515331	-	TSS_016349	1000	123	0	9	IP	within gene(s) PMM0545; 198nt upstream of gene PMM0544;
515349	-	TSS_016352	1000	630	0	37	IP	within gene(s) PMM0545; 216nt upstream of gene PMM0544;

515412	-	TSS_016371	1000	224	0	9 I	within gene(s) PMM0545;
515454	-	TSS_016377	1000	218	0	15 I	within gene(s) PMM0545;
515461	+	TSS_004038	1000	136	0	0 Ai	antisense to gene(s) PMM0545;
515529	-	TSS_016382	1000	105	0	0 I	within gene(s) PMM0545;
515643	-	TSS_016385	1000	112	0	15 I	within gene(s) PMM0545;
515694	-	TSS_016394	1000	206	0	6 I	within gene(s) PMM0545;
515718	-	TSS_016398	1000	235	0	3 I	within gene(s) PMM0545;
515763	-	TSS_016406	1000	296	0	21 I	within gene(s) PMM0545;
515778	-	TSS_016410	1000	216	0	3 I	within gene(s) PMM0545;
515800	+	TSS_004042	1000	457	0	1 Ai	antisense to gene(s) PMM0545;
515817	-	TSS_016415	1000	244	0	21 I	within gene(s) PMM0545;
515884	+	TSS_004045	1000	179	0	0 Ai	antisense to gene(s) PMM0545;
515916	+	TSS_004046	1000	270	0	4 Ai	antisense to gene(s) PMM0545;
515930	+	TSS_004049	1000	368	0	0 Ai	antisense to gene(s) PMM0545;
515964	-	TSS_016428	1000	169	0	0 I	within gene(s) PMM0545;
515970	+	TSS_004050	1000	268	0	0 Ai	antisense to gene(s) PMM0545;
515985	-	TSS_016431	1000	146	0	9 I	within gene(s) PMM0545;
516012	-	TSS_016439	1000	198	0	27 I	within gene(s) PMM0545;
516030	-	TSS_016442	1000	103	0	3 I	within gene(s) PMM0545;
516048	-	TSS_016445	1000	452	0	13 I	within gene(s) PMM0545;
516078	-	TSS_016451	1000	156	0	10 I	within gene(s) PMM0545;
516105	-	TSS_016456	1000	406	0	9 I	within gene(s) PMM0545;
516144	-	TSS_016460	1000	233	0	15 I	within gene(s) PMM0545;
516192	-	TSS_016468	1000	1112	0	6 I	within gene(s) PMM0545;
516228	-	TSS_016472	1000	203	0	4 I	within gene(s) PMM0545;
516267	-	TSS_016474	1000	256	0	0 I	within gene(s) PMM0545;
516294	-	TSS_016475	1000	167	0	0 I	within gene(s) PMM0545;
516308	-	TSS_016478	1000	189	0	9 I	within gene(s) PMM0545;
516345	-	TSS_016482	1000	236	0	3 I	within gene(s) PMM0545;
516813	-	TSS_016487	1000	135	0	2 I	within gene(s) PMM0546;
516902	-	TSS_016495	1000	117	0	9 I	within gene(s) PMM0546;
518401	-	TSS_016499	1000	148	0	1 P	18nt upstream of gene PMM0548;
518485	-	TSS_016501	1000	462	0	0 P	102nt upstream of gene PMM0548;
518726	+	TSS_004064	1000	204	0	0 I	within gene(s) PMM0549;
518824	+	TSS_004071	1000	115	0	0 I	within gene(s) PMM0549;
518878	+	TSS_004074	1000	236	0	9 IP	within gene(s) PMM0549; 209nt upstream of gene PMM0550;
518917	+	TSS_004083	1000	347	0	10 IP	within gene(s) PMM0549; 170nt upstream of gene PMM0550;
518971	+	TSS_004097	1000	784	0	43 IP	within gene(s) PMM0549; 116nt upstream of gene PMM0550;
519004	+	TSS_004103	1000	283	0	1 IP	within gene(s) PMM0549; 83nt upstream of gene PMM0550;
519082	-	TSS_016506	1000	525	0	1 O	-
519114	+	TSS_004106	1000	126	0	8 I	within gene(s) PMM0550;
519198	+	TSS_004113	1000	164	0	15 I	within gene(s) PMM0550;
519216	+	TSS_004117	1000	943	0	19 I	within gene(s) PMM0550;
519246	+	TSS_004125	1000	301	0	0 I	within gene(s) PMM0550;
519258	+	TSS_004126	1000	211	0	0 I	within gene(s) PMM0550;
519354	+	TSS_004129	1000	262	0	1 I	within gene(s) PMM0550;
519381	+	TSS_004131	1000	236	0	0 I	within gene(s) PMM0550;
519402	+	TSS_004134	1000	176	0	2 I	within gene(s) PMM0550;
519459	+	TSS_004139	1000	210	0	4 I	within gene(s) PMM0550;
519513	+	TSS_004149	1000	795	0	15 I	within gene(s) PMM0550;
519545	-	TSS_016512	1000	130	0	0 Ai	antisense to gene(s) PMM0550;
519562	+	TSS_004154	1000	190	0	12 I	within gene(s) PMM0550;
519579	+	TSS_004157	1000	514	0	3 I	within gene(s) PMM0550;
519588	+	TSS_004158	1000	636	0	0 I	within gene(s) PMM0550;
519602	-	TSS_016514	1000	131	0	0 Ai	antisense to gene(s) PMM0550;
519603	+	TSS_004159	1000	403	0	5 I	within gene(s) PMM0550;
519669	+	TSS_004167	1000	570	0	23 I	within gene(s) PMM0550;
519693	-	TSS_016515	1000	145	0	0 Ai	antisense to gene(s) PMM0550;
519837	+	TSS_004174	1000	122	0	21 I	within gene(s) PMM0550;
519874	+	TSS_004180	1000	137	0	0 I	within gene(s) PMM0550;
519993	+	TSS_004189	1000	2383	0	19 I	within gene(s) PMM0550;
520013	-	TSS_016518	1000	143	0	1 Ai	antisense to gene(s) PMM0550;
520043	-	TSS_016520	1000	196	0	0 Ai	antisense to gene(s) PMM0550;
520056	-	TSS_016521	1000	392	0	0 Ai	antisense to gene(s) PMM0550;
520155	+	TSS_004196	1000	281	0	9 I	within gene(s) PMM0550;
520175	-	TSS_016522	1000	466	0	0 Ai	antisense to gene(s) PMM0550;
520182	+	TSS_004197	1000	247	0	0 I	within gene(s) PMM0550;
520216	+	TSS_004200	1000	167	0	4 I	within gene(s) PMM0550;
520224	+	TSS_004201	1000	1236	0	1 I	within gene(s) PMM0550;
520239	+	TSS_004203	1000	261	0	6 I	within gene(s) PMM0550;
520278	+	TSS_004207	1000	226	0	7 I	within gene(s) PMM0550;
520352	-	TSS_016523	1000	185	0	12 Ai	antisense to gene(s) PMM0550;
520384	-	TSS_016528	1000	468	0	0 Ai	antisense to gene(s) PMM0550;
520485	+	TSS_004219	1000	194	0	0 IP	within gene(s) PMM0550; 107nt upstream of gene PMM0551;
520703	+	TSS_004232	1000	117	0	3 I	within gene(s) PMM0551;
521513	+	TSS_004239	1000	216	0	6 I	within gene(s) PMM0552;
521558	+	TSS_004245	1000	119	0	12 I	within gene(s) PMM0552;
521579	+	TSS_004249	1000	184	0	0 I	within gene(s) PMM0552;
521588	+	TSS_004250	1000	101	0	0 I	within gene(s) PMM0552;

521628	+	TSS_004253	1000	109	0	0	I	within gene(s) PMM0552;
521779	-	TSS_016542	1000	262	0	0	Ai	antisense to gene(s) PMM0552;
521930	+	TSS_004274	1000	105	0	0	I	within gene(s) PMM0552;
521945	-	TSS_016544	1000	103	0	0	Ai	antisense to gene(s) PMM0552;
521962	-	TSS_016547	1000	444	0	4	Ai	antisense to gene(s) PMM0552;
521966	+	TSS_004277	1000	137	0	6	I	within gene(s) PMM0552;
521993	+	TSS_004282	1000	277	0	9	I	within gene(s) PMM0552;
522030	+	TSS_004286	1000	137	0	1	I	within gene(s) PMM0552;
522065	+	TSS_004290	1000	111	0	15	I	within gene(s) PMM0552;
522119	+	TSS_004299	1000	273	0	6	I	within gene(s) PMM0552;
522140	+	TSS_004302	1000	229	0	16	I	within gene(s) PMM0552;
522292	-	TSS_016555	1000	314	0	0	Ai	antisense to gene(s) PMM0552;
522328	-	TSS_016556	1000	172	0	1	Ai	antisense to gene(s) PMM0552;
522365	+	TSS_004320	1000	138	0	9	I	within gene(s) PMM0552;
522474	-	TSS_016558	1000	128	0	0	Ai	antisense to gene(s) PMM0552;
522506	+	TSS_004335	1000	179	0	18	I	within gene(s) PMM0552;
522611	+	TSS_004340	1000	115	0	1	I	within gene(s) PMM0552;
522626	+	TSS_004343	1000	187	0	3	I	within gene(s) PMM0552;
522659	+	TSS_004345	1000	159	0	6	I	within gene(s) PMM0552;
522690	+	TSS_004349	1000	166	0	1	I	within gene(s) PMM0552;
523106	-	TSS_016565	1000	488	0	11	Ai	antisense to gene(s) PMM0552;
523135	-	TSS_016568	1000	321	0	0	Ai	antisense to gene(s) PMM0552;
523142	-	TSS_016569	1000	149	0	2	Ai	antisense to gene(s) PMM0552;
523163	-	TSS_016571	1000	485	0	0	Ai	antisense to gene(s) PMM0552;
523173	-	TSS_016573	1000	750	0	4	Ai	antisense to gene(s) PMM0552;
524269	-	TSS_016582	1000	121	0	0	Ai	antisense to gene(s) PMM0553;
524632	-	TSS_016585	1000	3311	0	2	Ai	antisense to gene(s) PMM0553;
524671	+	TSS_004398	1000	165	0	0	IP	within gene(s) PMM0553; 191nt upstream of gene PMM0554;
524738	+	TSS_004401	1000	201	0	0	IP	within gene(s) PMM0553; 124nt upstream of gene PMM0554;
524791	+	TSS_004403	1000	1207	0	1	IP	within gene(s) PMM0553; 71nt upstream of gene PMM0554;
525973	-	TSS_016593	1000	4448	0	4	P	18nt upstream of gene PMM0557;
526448	-	TSS_016600	1000	1231	0	4	P	15nt upstream of gene PMM0558;
527220	+	TSS_004421	1000	1521	0	0	P	22nt upstream of gene PMM0560;
527254	+	TSS_004422	1000	538	0	6	I	within gene(s) PMM0560;
529035	+	TSS_004436	1000	185	0	4	I	within gene(s) PMM0561;
529358	+	TSS_004440	1000	217	0	9	I	within gene(s) PMM0561;
531559	-	TSS_016614	1000	118	0	0	P	40nt upstream of gene PMM0564;
531701	+	TSS_004455	1000	301	0	4	P	15nt upstream of gene PMM0565;
532356	-	TSS_016617	1000	307	0	1	Ai	antisense to gene(s) PMM0565;
532769	+	TSS_004463	1000	114	0	3	I	within gene(s) PMM0565;
534371	+	TSS_004467	1000	229	0	7	P	6nt upstream of gene PMM0567;
538166	+	TSS_004476	1000	743	0	2	P	179nt upstream of gene PMM0570;
539865	+	TSS_004481	1000	333	0	0	Ad	antisense to gene(s) PMM0573 (15nt downstream);
539992	+	TSS_004483	1000	931	0	0	Ai	antisense to gene(s) PMM0573;
540074	+	TSS_004487	1000	127	0	8	Ai	antisense to gene(s) PMM0573;
540155	-	TSS_016636	1000	305	0	1	P	1nt upstream of gene PMM0573;
540186	-	TSS_016640	1000	221	0	9	P	32nt upstream of gene PMM0573;
540220	-	TSS_016647	1000	183	0	12	P	66nt upstream of gene PMM0573;
540317	-	TSS_016649	1000	211	0	0	P	163nt upstream of gene PMM0573;
541550	+	TSS_004494	1000	213	0	0	Ai	antisense to gene(s) PMM0577;
541837	-	TSS_016665	1000	313	0	6	I	within gene(s) PMM0577;
542147	-	TSS_016682	1000	2965	0	6	I	within gene(s) PMM0577;
542296	-	TSS_016691	1000	174	0	1	I	within gene(s) PMM0577;
542434	-	TSS_016694	1000	106	0	1	P	0nt upstream of gene PMM0577;
543050	+	TSS_004501	1000	112	0	0	Ai	antisense to gene(s) PMM0578;
543260	+	TSS_004503	1000	297	0	2	P	50nt upstream of gene PMM0579;
543284	-	TSS_016706	1000	630	0	4	P	38nt upstream of gene PMM0578;
546045	-	TSS_016715	1000	1774	0	1	I	within gene(s) PMM0580;
546519	+	TSS_004514	1000	129	0	0	Ai	antisense to gene(s) PMM0581;
546530	+	TSS_004516	1000	1329	0	2	Ai	antisense to gene(s) PMM0581;
546549	+	TSS_004518	1000	129	0	0	Ai	antisense to gene(s) PMM0581;
546713	-	TSS_016728	1000	169	0	39	I	within gene(s) PMM0581;
546779	-	TSS_016741	1000	497	0	12	I	within gene(s) PMM0581;
546808	-	TSS_016750	1000	203	0	18	I	within gene(s) PMM0581;
546819	-	TSS_016752	1000	374	0	5	I	within gene(s) PMM0581;
548375	-	TSS_016777	1000	101	0	0	I	within gene(s) PMM0583;
548771	-	TSS_016788	1000	131	0	3	I	within gene(s) PMM0583;
548961	-	TSS_016799	1000	2954	0	2	P	43nt upstream of gene PMM0583;
550115	+	TSS_004533	1000	163	0	0	Ai	antisense to gene(s) PMM0584;
551319	-	TSS_016819	1000	362	0	2	P	13nt upstream of gene PMM0584;
561219	-	TSS_016844	1000	223	0	6	I	within gene(s) PMM0593;
562701	+	TSS_004552	1000	288	0	0	Ai	antisense to gene(s) PMM0594;
562722	+	TSS_004554	1000	104	0	6	Ai	antisense to gene(s) PMM0594;
563071	-	TSS_016879	1000	104	0	1	I	within gene(s) PMM0594;
563080	-	TSS_016880	1000	137	0	0	I	within gene(s) PMM0594;
563864	-	TSS_016891	1000	134	0	2	I	within gene(s) PMM0594;
564355	+	TSS_004563	1000	144	0	1	Ai	antisense to gene(s) PMM0595;
564712	-	TSS_016901	1000	117	0	9	I	within gene(s) PMM0595;
564862	-	TSS_016905	1000	117	0	0	I	within gene(s) PMM0595;

565234	-	TSS_016908	1000	146	0	4 IP	within gene(s) PMM0596; 90nt upstream of gene PMM0595;
565420	-	TSS_016911	1000	947	0	1 I	within gene(s) PMM0596;
569418	-	TSS_016928	1000	134	0	7 I	within gene(s) PMM0599;
569475	-	TSS_016934	1000	113	0	6 I	within gene(s) PMM0599;
569600	-	TSS_016938	1000	965	0	8 P	20nt upstream of gene PMM0599;
570717	+	TSS_004588	1000	196	0	6 I	within gene(s) PMM0600;
571704	+	TSS_004596	1000	107	0	2 I	within gene(s) PMM0601;
573409	+	TSS_004610	1000	115	0	0 I	within gene(s) PMM0603;
573526	-	TSS_016952	1000	587	0	1 Ai	antisense to gene(s) PMM0603;
574977	+	TSS_004617	1000	210	0	1 Ai	antisense to gene(s) PMM0605;
575199	-	TSS_016959	1000	1743	0	1 P	16nt upstream of gene PMM0605;
577744	+	TSS_004624	1000	127	0	0 I	within gene(s) PMM0608;
578017	-	TSS_016965	1000	216	0	0 Ai	antisense to gene(s) PMM0608;
578318	+	TSS_004631	1000	726	0	2 IP	within gene(s) PMM0608; 247nt upstream of gene PMM0609;
579495	+	TSS_004642	1000	269	0	0 I	within gene(s) PMM0609;
582602	-	TSS_016978	1000	424	0	3 I	within gene(s) PMM0611;
582767	-	TSS_016981	1000	104	0	5 P	17nt upstream of gene PMM0611;
584058	-	TSS_016987	1000	140	0	2 I	within gene(s) PMM0613;
585123	+	TSS_004661	1000	1375	0	6 P	120nt upstream of gene PMM0614;
585737	+	TSS_004665	1000	536	0	1 IP	within gene(s) PMM0614; 160nt upstream of gene PMM0615;
585797	+	TSS_004667	1000	331	0	4 IP	within gene(s) PMM0614; 100nt upstream of gene PMM0615;
586359	+	TSS_004675	1000	162	0	3 I	within gene(s) PMM0615;
587070	-	TSS_016994	1000	193	0	0 Ai	antisense to gene(s) PMM0616;
588622	+	TSS_004680	1000	1026	0	2 P	9nt upstream of gene PMM0618;
588874	+	TSS_004682	1000	226	0	1 I	within gene(s) PMM0618;
589671	+	TSS_004687	1000	141	0	0 P	29nt upstream of gene PMM0619;
590069	+	TSS_004693	1000	307	0	12 I	within gene(s) PMM0619;
590180	+	TSS_004706	1000	375	0	40 I	within gene(s) PMM0619;
590216	+	TSS_004714	1000	425	0	0 I	within gene(s) PMM0619;
590314	-	TSS_016999	1000	246	0	1 Ai	antisense to gene(s) PMM0619;
590488	-	TSS_017002	1000	116	0	0 Ai	antisense to gene(s) PMM0619;
590670	-	TSS_017003	1000	1429	0	3 Ai	antisense to gene(s) PMM0619;
590828	+	TSS_004731	1000	116	0	0 I	within gene(s) PMM0619;
591023	+	TSS_004751	1000	467	0	19 I	within gene(s) PMM0619;
591039	+	TSS_004755	1000	118	0	12 I	within gene(s) PMM0619;
591055	-	TSS_017007	1000	597	0	0 Ai	antisense to gene(s) PMM0619;
591103	-	TSS_017008	1000	143	0	0 Ai	antisense to gene(s) PMM0619;
591311	+	TSS_004771	1000	111	0	0 I	within gene(s) PMM0619;
593663	-	TSS_017024	1000	145	0	0 I	within gene(s) PMM0622;
593808	-	TSS_017026	1000	704	0	1 P	16nt upstream of gene PMM0622;
594678	+	TSS_004781	1000	155	0	2 I	within gene(s) PMM0623;
595886	+	TSS_004785	1000	109	0	1 P	18nt upstream of gene PMM0626;
596385	-	TSS_017034	1000	971	0	1 O	-
597035	-	TSS_017037	1000	4954	0	2 Ai	antisense to gene(s) PMM0627;
597084	-	TSS_017039	1000	134	0	0 Ai	antisense to gene(s) PMM0627;
597087	+	TSS_004789	1000	1155	0	12 I	within gene(s) PMM0627;
597111	+	TSS_004792	1000	635	0	0 I	within gene(s) PMM0627;
597120	+	TSS_004793	1000	1575	0	4 I	within gene(s) PMM0627;
597162	+	TSS_004802	1000	2440	0	34 I	within gene(s) PMM0627;
597165	-	TSS_017040	1000	2739	0	0 Ai	antisense to gene(s) PMM0627;
597182	-	TSS_017041	1000	128	0	0 Ai	antisense to gene(s) PMM0627;
597189	+	TSS_004809	1000	1932	0	3 I	within gene(s) PMM0627;
597198	+	TSS_004810	1000	1041	0	0 I	within gene(s) PMM0627;
597249	-	TSS_017043	1000	112	0	0 Ai	antisense to gene(s) PMM0627;
597255	+	TSS_004828	1000	2444	0	51 I	within gene(s) PMM0627;
597291	+	TSS_004833	1000	1178	0	18 I	within gene(s) PMM0627;
597318	+	TSS_004836	1000	499	0	0 I	within gene(s) PMM0627;
597430	-	TSS_017047	1000	230	0	4 Ai	antisense to gene(s) PMM0627;
600230	+	TSS_004848	1000	424	0	0 P	26nt upstream of gene PMM0631;
602377	-	TSS_017057	1000	241	0	0 I	within gene(s) PMM0633;
602756	-	TSS_017058	1000	109	0	0 I	within gene(s) PMM0633;
602896	-	TSS_017063	1000	500	0	5 P	98nt upstream of gene PMM0633;
605794	+	TSS_004856	1000	158	0	6 P	19nt upstream of gene PMM0637;
609209	-	TSS_017089	1000	488	0	0 Ai	antisense to gene(s) PMM0640;
609497	+	TSS_004864	1000	132	0	0 I	within gene(s) PMM0640;
610210	+	TSS_004870	1000	2978	0	7 P	16nt upstream of gene PMM0641;
610941	+	TSS_004876	1000	7037	0	3 P	17nt upstream of gene PMM0642;
611411	+	TSS_004908	1000	643	0	30 I	within gene(s) PMM0642;
611447	+	TSS_004914	1000	260	0	0 I	within gene(s) PMM0642;
611474	+	TSS_004915	1000	244	0	3 I	within gene(s) PMM0642;
611513	+	TSS_004918	1000	311	0	1 I	within gene(s) PMM0642;
611588	+	TSS_004923	1000	107	0	0 I	within gene(s) PMM0642;
611626	-	TSS_017094	1000	727	0	10 Ai	antisense to gene(s) PMM0642;
611674	-	TSS_017098	1000	424	0	0 Ai	antisense to gene(s) PMM0642;
611699	-	TSS_017099	1000	346	0	4 Ai	antisense to gene(s) PMM0642;
611855	+	TSS_004954	1000	219	0	9 I	within gene(s) PMM0642;
611880	+	TSS_004956	1000	110	0	1 I	within gene(s) PMM0642;
611903	+	TSS_004959	1000	149	0	16 I	within gene(s) PMM0642;
612008	+	TSS_004968	1000	228	0	9 I	within gene(s) PMM0642;

612040	-	TSS_017104	1000	160	0	0 Ai	antisense to gene(s) PMM0642;
612065	+	TSS_004979	1000	109	0	3 IP	within gene(s) PMM0642; 236nt upstream of gene PMM0643;
612104	+	TSS_004986	1000	190	0	24 IP	within gene(s) PMM0642; 197nt upstream of gene PMM0643;
614001	-	TSS_017120	1000	109	0	1 I	within gene(s) PMM0644;
614082	-	TSS_017123	1000	278	0	7 I	within gene(s) PMM0644;
614781	-	TSS_017134	1000	313	0	2 P	15nt upstream of gene PMM0644;
617157	-	TSS_017143	1000	6978	0	9 Ai	antisense to gene(s) PMM0646;
618720	+	TSS_005009	1000	242	0	8 Ai	antisense to gene(s) PMM0648;
619598	+	TSS_005011	1000	192	0	1 P	18nt upstream of gene PMM0649;
620989	-	TSS_017156	1000	2374	0	5 P	13nt upstream of gene PMM0651;
621700	-	TSS_017160	1000	256	0	0 P	156nt upstream of gene PMM0652;
624033	-	TSS_017166	1000	187	0	5 O	-
624976	+	TSS_005022	1000	226	0	2 I	within gene(s) PMM0658;
626301	+	TSS_005026	1000	364	0	0 I	within gene(s) PMM0659;
627045	-	TSS_017175	1000	1291	0	7 O	-
627516	+	TSS_005030	1000	155	0	0 O	-
627971	-	TSS_017179	1000	12326	0	3 O	-
628169	+	TSS_005033	1000	2374	0	2 O	-
628451	-	TSS_017189	1000	105	0	9 I	within gene(s) PMM0660;
628505	-	TSS_017195	1000	230	0	6 I	within gene(s) PMM0660;
628656	-	TSS_017199	1000	421	0	0 P	10nt upstream of gene PMM0660;
629265	-	TSS_017220	1000	106	0	7 I	within gene(s) PMM0661;
629355	-	TSS_017224	1000	120	0	1 I	within gene(s) PMM0661;
629366	-	TSS_017226	1000	129	0	4 I	within gene(s) PMM0661;
629577	-	TSS_017238	1000	171	0	12 I	within gene(s) PMM0661;
629618	-	TSS_017242	1000	201	0	3 I	within gene(s) PMM0661;
629637	-	TSS_017246	1000	227	0	15 I	within gene(s) PMM0661;
629655	-	TSS_017250	1000	123	0	8 I	within gene(s) PMM0661;
629664	+	TSS_005044	1000	170	0	9 Ai	antisense to gene(s) PMM0661;
630039	-	TSS_017281	1000	127	0	9 I	within gene(s) PMM0661;
630075	-	TSS_017285	1000	309	0	3 I	within gene(s) PMM0661;
630111	-	TSS_017287	1000	120	0	0 I	within gene(s) PMM0661;
630158	+	TSS_005056	1000	180	0	7 Ai	antisense to gene(s) PMM0661;
630201	-	TSS_017296	1000	176	0	7 I	within gene(s) PMM0661;
630210	-	TSS_017298	1000	145	0	6 I	within gene(s) PMM0661;
630270	-	TSS_017308	1000	132	0	11 I	within gene(s) PMM0661;
630363	-	TSS_017323	1000	348	0	24 I	within gene(s) PMM0661;
630390	-	TSS_017328	1000	105	0	3 I	within gene(s) PMM0661;
630465	-	TSS_017336	1000	292	0	6 I	within gene(s) PMM0661;
630554	+	TSS_005061	1000	172	0	0 Ai	antisense to gene(s) PMM0661;
630684	-	TSS_017348	1000	211	0	18 I	within gene(s) PMM0661;
630807	-	TSS_017356	1000	169	0	21 I	within gene(s) PMM0661;
631086	-	TSS_017372	1000	1344	0	0 I	within gene(s) PMM0661;
631116	-	TSS_017374	1000	5069	0	1 I	within gene(s) PMM0661;
631165	+	TSS_005067	1000	151	0	0 P	27nt upstream of gene PMM0662;
633583	+	TSS_005079	1000	450	0	1 P	23nt upstream of gene PMM0664;
633593	+	TSS_005080	1000	110	0	0 P	13nt upstream of gene PMM0664;
635224	-	TSS_017386	1000	190	0	0 IP	within gene(s) PMM0666; 40nt upstream of gene PMM0665;
635265	-	TSS_017387	1000	170	0	0 IP	within gene(s) PMM0666; 81nt upstream of gene PMM0665;
638295	+	TSS_005094	1000	225	0	1 Ai	antisense to gene(s) PMM0670;
638760	-	TSS_017397	1000	126	0	3 IP	within gene(s) PMM0671; 217nt upstream of gene PMM0670;
643136	+	TSS_005099	1000	170	0	2 P	21nt upstream of gene PMM0675;
643669	+	TSS_005100	1000	328	0	0 P	25nt upstream of gene PMM0676;
644589	+	TSS_005112	1000	147	0	3 I	within gene(s) PMM0676;
644684	+	TSS_005115	1000	116	0	1 I	within gene(s) PMM0676;
644903	-	TSS_017410	1000	122	0	2 Ai	antisense to gene(s) PMM0676;
644914	-	TSS_017412	1000	711	0	0 Ad	antisense to gene(s) PMM0676 (4nt downstream);
647282	-	TSS_017416	1000	1813	0	2 P	69nt upstream of gene PMM0678;
649461	+	TSS_005130	1000	142	0	6 I	within gene(s) PMM0681;
651413	+	TSS_005134	1000	267	0	0 I	within gene(s) PMM0683;
652626	+	TSS_005137	1000	1641	0	0 O	-
652951	+	TSS_005140	1000	185	0	4 I	within gene(s) PMM0684;
653026	+	TSS_005146	1000	143	0	0 I	within gene(s) PMM0684;
653294	+	TSS_005154	1000	137	0	9 P	83nt upstream of gene PMM0685;
653400	-	TSS_017433	1000	626	0	1 Ai	antisense to gene(s) PMM0685;
653980	-	TSS_017439	1000	14157	0	2 O	-
655603	-	TSS_017444	1000	2100	0	2 I	within gene(s) PMM0687;
655934	-	TSS_017447	1000	2080	0	1 O	-
656634	-	TSS_017452	1000	101	0	2 P	42nt upstream of gene PMM0688;
656661	-	TSS_017455	1000	108	0	0 P	69nt upstream of gene PMM0688;
656679	-	TSS_017457	1000	427	0	0 P	87nt upstream of gene PMM0688;
656913	-	TSS_017458	1000	122	0	12 O	-
656940	-	TSS_017462	1000	151	0	2 O	-
657528	-	TSS_017467	1000	1763	0	4 O	-
657710	+	TSS_005164	1000	143	0	2 O	-
657998	+	TSS_005168	1000	187	0	4 O	-
659043	-	TSS_017475	1000	1276	0	3 P	15nt upstream of gene PMM0690;
659754	+	TSS_005173	1000	900	0	1 Ad	antisense to gene(s) PMM0691 (2nt downstream);
660094	-	TSS_017481	1000	248	0	4 P	133nt upstream of gene PMM0691;

660419	-	TSS_017484	1000	270	0	6	P	22nt upstream of gene PMM0692;
661103	-	TSS_017487	1000	126	0	2	I	within gene(s) PMM0693;
661983	+	TSS_005183	1000	205	0	2	O	-
663678	-	TSS_017493	1000	374	0	7	P	16nt upstream of gene PMM0697;
664058	-	TSS_017496	1000	110	0	0	I	within gene(s) PMM0698;
665165	+	TSS_005193	1000	1780	0	4	P	16nt upstream of gene PMM0699;
667292	+	TSS_005197	1000	210	0	2	P	14nt upstream of gene PMM0703;
668059	+	TSS_005202	1000	362	0	2	I	within gene(s) PMM0704;
669388	+	TSS_005206	1000	150	0	0	P	12nt upstream of gene PMM0705;
671615	-	TSS_017508	1000	140	0	0	I	within gene(s) PMM0707;
674151	+	TSS_005222	1000	1011	0	0	Ai	antisense to gene(s) PMM0709;
674830	-	TSS_017525	1000	353	0	1	I	within gene(s) PMM0709;
674981	+	TSS_005227	1000	140	0	0	Ai	antisense to gene(s) PMM0709;
675828	+	TSS_005232	1000	16597	0	6	P	26nt upstream of gene PMM0710;
675938	+	TSS_005239	1000	194	0	0	I	within gene(s) PMM0710;
675986	+	TSS_005241	1000	124	0	0	I	within gene(s) PMM0710;
676023	+	TSS_005244	1000	176	0	7	I	within gene(s) PMM0710;
676095	-	TSS_017530	1000	107	0	0	Ai	antisense to gene(s) PMM0710;
676283	+	TSS_005259	1000	278	0	1	I	within gene(s) PMM0710;
676460	+	TSS_005272	1000	152	0	0	I	within gene(s) PMM0710;
676791	-	TSS_017538	1000	535	0	1	Ai	antisense to gene(s) PMM0710;
678373	+	TSS_005278	1000	152	0	0	Ai	antisense to gene(s) PMM0712;
678437	+	TSS_005281	1000	224	0	0	Ai	antisense to gene(s) PMM0712;
680440	-	TSS_017549	1000	586	0	0	P	15nt upstream of gene PMM0713;
680511	-	TSS_017550	1000	173	0	0	PAi	86nt upstream of gene PMM0713; antisense to gene(s) PMM0714;
682685	-	TSS_017552	1000	125	0	0	P	0nt upstream of gene PMM0717;
685615	-	TSS_017554	1000	1310	0	5	P	13nt upstream of gene PMM0722;
687667	+	TSS_005297	1000	369	0	0	IP	within gene(s) PMM0724; 61nt upstream of gene PMM0725;
688326	-	TSS_017561	1000	370	0	0	Ai	antisense to gene(s) PMM0725;
688353	+	TSS_005306	1000	251	0	0	I	within gene(s) PMM0725;
688629	+	TSS_005307	1000	111	0	0	O	-
688866	+	TSS_005310	1000	2158	0	1	O	-
689153	+	TSS_005313	1000	398	0	0	O	-
689593	-	TSS_017567	1000	140	0	10	P	17nt upstream of gene PMM0726;
696382	-	TSS_017574	1000	200	0	0	P	18nt upstream of gene PMM0732;
698300	-	TSS_017577	1000	328	0	3	P	15nt upstream of gene PMM0734;
699080	+	TSS_005320	1000	117	0	7	O	-
702227	-	TSS_017592	1000	174	0	1	P	16nt upstream of gene PMM0739;
702332	+	TSS_005328	1000	1152	0	5	P	11nt upstream of gene PMM0740;
702507	-	TSS_017593	1000	130	0	0	O	-
702516	-	TSS_017594	1000	599	0	0	O	-
703275	+	TSS_005333	1000	789	0	1	P	17nt upstream of gene PMM0742;
704155	+	TSS_005356	1000	193	0	1	Ai	antisense to gene(s) PMM0743;
704189	+	TSS_005357	1000	128	0	0	Ai	antisense to gene(s) PMM0743;
704207	+	TSS_005358	1000	382	0	0	Ai	antisense to gene(s) PMM0743;
704304	-	TSS_017608	1000	125	0	0	I	within gene(s) PMM0743;
704315	+	TSS_005362	1000	356	0	1	Ai	antisense to gene(s) PMM0743;
704361	-	TSS_017609	1000	258	0	0	I	within gene(s) PMM0743;
704397	-	TSS_017615	1000	332	0	33	I	within gene(s) PMM0743;
704440	-	TSS_017622	1000	115	0	8	I	within gene(s) PMM0743;
704459	-	TSS_017630	1000	296	0	0	I	within gene(s) PMM0743;
704526	-	TSS_017634	1000	197	0	7	I	within gene(s) PMM0743;
704718	-	TSS_017651	1000	128	0	0	I	within gene(s) PMM0743;
704789	+	TSS_005369	1000	150	0	0	Ai	antisense to gene(s) PMM0743;
704796	-	TSS_017660	1000	183	0	7	I	within gene(s) PMM0743;
704808	-	TSS_017661	1000	219	0	0	I	within gene(s) PMM0743;
704823	-	TSS_017665	1000	266	0	9	I	within gene(s) PMM0743;
704949	+	TSS_005374	1000	217	0	9	Ai	antisense to gene(s) PMM0743;
705078	-	TSS_017674	1000	306	0	19	I	within gene(s) PMM0743;
705171	-	TSS_017692	1000	101	0	15	I	within gene(s) PMM0743;
705360	-	TSS_017698	1000	117	0	6	I	within gene(s) PMM0743;
705461	-	TSS_017704	1000	206	0	0	I	within gene(s) PMM0743;
705815	-	TSS_017714	1000	336	0	1	P	23nt upstream of gene PMM0743;
708310	+	TSS_005389	1000	1266	0	2	Ai	antisense to gene(s) PMM0746;
708607	-	TSS_017719	1000	445	0	6	P	14nt upstream of gene PMM0746;
710247	+	TSS_005394	1000	268	0	1	IP	within gene(s) PMM0748; 23nt upstream of gene PMM0749;
710300	+	TSS_005396	1000	145	0	1	I	within gene(s) PMM0749;
711092	+	TSS_005403	1000	139	0	0	I	within gene(s) PMM0749;
711431	-	TSS_017726	1000	165	0	1	Ai	antisense to gene(s) PMM0749;
711539	+	TSS_005404	1000	1596	0	1	I	within gene(s) PMM0750;
711829	-	TSS_017729	1000	660	0	6	Ai	antisense to gene(s) PMM0750;
712163	+	TSS_005407	1000	1585	0	0	IP	within gene(s) PMM0750; 42nt upstream of gene PMM0751;
713413	+	TSS_005414	1000	163	0	7	P	65nt upstream of gene PMM0753;
713700	+	TSS_005425	1000	384	0	5	I	within gene(s) PMM0753;
713901	+	TSS_005434	1000	266	0	14	I	within gene(s) PMM0753;
713922	+	TSS_005437	1000	263	0	0	I	within gene(s) PMM0753;
713937	+	TSS_005438	1000	172	0	4	I	within gene(s) PMM0753;
713970	+	TSS_005442	1000	151	0	0	I	within gene(s) PMM0753;
714097	+	TSS_005458	1000	1136	0	27	IP	within gene(s) PMM0753; 130nt upstream of gene PMM0754;

714137	-	TSS_017743	1000	104	0	0	Ai	antisense to gene(s) PMM0753;
714364	-	TSS_017747	1000	175	0	1	Ai	antisense to gene(s) PMM0754;
714683	+	TSS_005473	1000	130	0	0	IP	within gene(s) PMM0754; 206nt upstream of gene PMM0755;
714804	-	TSS_017750	1000	124	0	0	Ai	antisense to gene(s) PMM0754;
720208	+	TSS_005489	1000	133	0	0	Ai	antisense to gene(s) PMM0758;
720550	-	TSS_017776	1000	199	0	21	I	within gene(s) PMM0758;
720569	+	TSS_005492	1000	118	0	0	Ai	antisense to gene(s) PMM0758;
720579	-	TSS_017781	1000	150	0	0	I	within gene(s) PMM0758;
720641	-	TSS_017783	1000	627	0	10	I	within gene(s) PMM0758;
720675	-	TSS_017788	1000	171	0	0	I	within gene(s) PMM0758;
720702	-	TSS_017792	1000	144	0	4	I	within gene(s) PMM0758;
723610	-	TSS_017826	1000	104	0	0	I	within gene(s) PMM0760;
723718	-	TSS_017834	1000	308	0	18	I	within gene(s) PMM0760;
723763	-	TSS_017849	1000	1768	0	51	I	within gene(s) PMM0760;
723796	-	TSS_017856	1000	112	0	12	I	within gene(s) PMM0760;
723823	-	TSS_017860	1000	152	0	39	I	within gene(s) PMM0760;
723871	-	TSS_017869	1000	395	0	2	I	within gene(s) PMM0760;
723880	-	TSS_017871	1000	206	0	3	I	within gene(s) PMM0760;
723898	-	TSS_017873	1000	120	0	9	I	within gene(s) PMM0760;
723931	-	TSS_017878	1000	123	0	0	I	within gene(s) PMM0760;
723949	-	TSS_017881	1000	164	0	3	I	within gene(s) PMM0760;
723979	-	TSS_017884	1000	107	0	0	I	within gene(s) PMM0760;
723994	-	TSS_017886	1000	317	0	6	I	within gene(s) PMM0760;
724072	-	TSS_017894	1000	126	0	4	I	within gene(s) PMM0760;
724249	-	TSS_017907	1000	134	0	9	I	within gene(s) PMM0760;
724284	-	TSS_017912	1000	186	0	8	I	within gene(s) PMM0760;
724366	-	TSS_017922	1000	140	0	6	I	within gene(s) PMM0760;
724438	-	TSS_017929	1000	274	0	18	I	within gene(s) PMM0760;
724578	-	TSS_017934	1000	4421	0	2	P	20nt upstream of gene PMM0760;
725488	+	TSS_005512	1000	167	0	0	P	25nt upstream of gene PMM0762;
725918	+	TSS_005516	1000	105	0	0	I	within gene(s) PMM0762;
726575	+	TSS_005537	1000	139	0	6	I	within gene(s) PMM0762;
728301	-	TSS_017946	1000	241	0	0	Ai	antisense to gene(s) PMM0764;
729871	-	TSS_017964	1000	198	0	5	I	within gene(s) PMM0766;
729981	-	TSS_017976	1000	186	0	8	I	within gene(s) PMM0766;
730261	-	TSS_017980	1000	806	0	4	P	15nt upstream of gene PMM0766;
730331	+	TSS_005584	1000	584	0	0	P	103nt upstream of gene PMM0767;
730435	+	TSS_005585	1000	1049	0	0	I	within gene(s) PMM0767;
730710	+	TSS_005595	1000	121	0	12	I	within gene(s) PMM0767;
730737	+	TSS_005601	1000	459	0	4	I	within gene(s) PMM0767;
730749	+	TSS_005603	1000	126	0	0	I	within gene(s) PMM0767;
731037	+	TSS_005621	1000	226	0	9	I	within gene(s) PMM0767;
731067	+	TSS_005629	1000	362	0	15	I	within gene(s) PMM0767;
731240	-	TSS_017991	1000	184	0	0	Ai	antisense to gene(s) PMM0767;
732872	+	TSS_005638	1000	1624	0	6	P	21nt upstream of gene PMM0769;
733079	+	TSS_005643	1000	108	0	1	I	within gene(s) PMM0769;
733157	+	TSS_005647	1000	149	0	0	I	within gene(s) PMM0769;
733214	-	TSS_017999	1000	125	0	0	Ai	antisense to gene(s) PMM0769;
733226	-	TSS_018000	1000	346	0	0	Ai	antisense to gene(s) PMM0769;
733478	+	TSS_005661	1000	150	0	9	I	within gene(s) PMM0769;
733779	+	TSS_005676	1000	126	0	10	I	within gene(s) PMM0769;
733808	+	TSS_005678	1000	144	0	0	I	within gene(s) PMM0769;
733838	+	TSS_005688	1000	346	0	19	I	within gene(s) PMM0769;
733922	+	TSS_005698	1000	123	0	6	I	within gene(s) PMM0769;
733937	+	TSS_005699	1000	113	0	0	I	within gene(s) PMM0769;
734059	-	TSS_018005	1000	1114	0	1	Ai	antisense to gene(s) PMM0769;
734072	-	TSS_018007	1000	350	0	7	Ai	antisense to gene(s) PMM0769;
734116	-	TSS_018013	1000	419	0	6	Ai	antisense to gene(s) PMM0769;
734269	+	TSS_005709	1000	3600	0	4	P	18nt upstream of gene PMM0770;
737785	-	TSS_018026	1000	139	0	0	I	within gene(s) PMM0774;
737882	-	TSS_018030	1000	141	0	0	I	within gene(s) PMM0774;
738561	+	TSS_005734	1000	324	0	0	Ai	antisense to gene(s) PMM0774;
738616	-	TSS_018037	1000	190	0	0	I	within gene(s) PMM0774;
738854	-	TSS_018041	1000	125	0	6	I	within gene(s) PMM0774;
738964	-	TSS_018045	1000	178	0	0	I	within gene(s) PMM0774;
739095	-	TSS_018047	1000	1506	0	2	P	13nt upstream of gene PMM0774;
739318	-	TSS_018051	1000	144	0	28	IP	within gene(s) PMM0775; 236nt upstream of gene PMM0774;
739410	-	TSS_018057	1000	1619	0	10	P	14nt upstream of gene PMM0775;
740068	+	TSS_005740	1000	514	0	2	P	38nt upstream of gene PMM0777;
741992	+	TSS_005763	1000	836	0	0	P	15nt upstream of gene PMM0779;
743202	-	TSS_018073	1000	124	0	3	I	within gene(s) PMM0781;
743244	-	TSS_018078	1000	128	0	9	I	within gene(s) PMM0781;
743280	-	TSS_018086	1000	232	0	12	I	within gene(s) PMM0781;
743304	-	TSS_018090	1000	311	0	6	I	within gene(s) PMM0781;
743337	-	TSS_018092	1000	115	0	3	I	within gene(s) PMM0781;
743394	-	TSS_018096	1000	520	0	9	I	within gene(s) PMM0781;
743405	-	TSS_018098	1000	154	0	0	I	within gene(s) PMM0781;
743421	-	TSS_018100	1000	936	0	18	I	within gene(s) PMM0781;
743442	-	TSS_018105	1000	137	0	0	I	within gene(s) PMM0781;

743454	-	TSS_018107	1000	399	0	3	I	within gene(s) PMM0781;
743484	-	TSS_018111	1000	260	0	14	I	within gene(s) PMM0781;
743520	-	TSS_018115	1000	186	0	9	I	within gene(s) PMM0781;
743544	-	TSS_018118	1000	116	0	0	I	within gene(s) PMM0781;
743565	-	TSS_018119	1000	258	0	6	I	within gene(s) PMM0781;
743601	-	TSS_018121	1000	141	0	6	I	within gene(s) PMM0781;
743622	-	TSS_018124	1000	490	0	1	I	within gene(s) PMM0781;
743631	-	TSS_018125	1000	327	0	3	I	within gene(s) PMM0781;
743664	-	TSS_018132	1000	529	0	15	I	within gene(s) PMM0781;
743682	-	TSS_018133	1000	234	0	3	I	within gene(s) PMM0781;
743694	-	TSS_018135	1000	277	0	0	I	within gene(s) PMM0781;
743706	-	TSS_018136	1000	113	3.90E-06	0	I	within gene(s) PMM0781;
743715	-	TSS_018138	1000	209	0	10	I	within gene(s) PMM0781;
743745	-	TSS_018142	1000	930	0	1	I	within gene(s) PMM0781;
743763	-	TSS_018143	1000	175	0	9	I	within gene(s) PMM0781;
743790	-	TSS_018147	1000	384	0	3	I	within gene(s) PMM0781;
743799	-	TSS_018148	1000	2366	0	33	I	within gene(s) PMM0781;
743847	-	TSS_018161	1000	200	0	6	I	within gene(s) PMM0781;
743859	-	TSS_018165	1000	407	0	27	I	within gene(s) PMM0781;
743904	-	TSS_018172	1000	107	0	0	I	within gene(s) PMM0781;
743916	-	TSS_018174	1000	482	0	4	I	within gene(s) PMM0781;
743937	-	TSS_018176	1000	281	0	0	I	within gene(s) PMM0781;
743949	-	TSS_018180	1000	152	0	6	I	within gene(s) PMM0781;
744091	-	TSS_018186	1000	10061	0	3	P	43nt upstream of gene PMM0781;
745876	+	TSS_005780	1000	222	0	2	Ai	antisense to gene(s) PMM0784;
746288	-	TSS_018207	1000	242	0	2	I	within gene(s) PMM0784;
746583	-	TSS_018218	1000	1201	0	5	P	17nt upstream of gene PMM0784;
747217	-	TSS_018230	1000	233	0	5	I	within gene(s) PMM0785;
747247	-	TSS_018238	1000	107	0	6	I	within gene(s) PMM0785;
747387	-	TSS_018250	1000	335	0	9	I	within gene(s) PMM0785;
747484	-	TSS_018255	1000	162	0	4	I	within gene(s) PMM0785;
749173	-	TSS_018266	1000	399	0	4	I	within gene(s) PMM0787;
752029	-	TSS_018281	1000	206	0	8	P	25nt upstream of gene PMM0790;
752047	+	TSS_005807	1000	1287	0	12	Ai	antisense to gene(s) PMM0791;
755386	-	TSS_018286	1000	457	0	3	IP	within gene(s) PMM0795; 114nt upstream of gene PMM0794;
757822	-	TSS_018294	1000	112	0	0	P	66nt upstream of gene PMM0796;
760249	-	TSS_018301	1000	150	0	11	P	17nt upstream of gene PMM0800;
760930	-	TSS_018307	1000	128	0	5	P	14nt upstream of gene PMM0801;
762992	+	TSS_005820	1000	1228	0	0	P	16nt upstream of gene PMM0804;
763062	+	TSS_005822	1000	462	0	17	I	within gene(s) PMM0804;
763152	+	TSS_005834	1000	120	0	0	I	within gene(s) PMM0804;
763194	+	TSS_005841	1000	149	0	27	I	within gene(s) PMM0804;
763266	+	TSS_005848	1000	203	0	7	I	within gene(s) PMM0804;
764067	+	TSS_005860	1000	372	0	0	Ai	antisense to gene(s) PMM0806;
764468	-	TSS_018319	1000	159	0	3	P	26nt upstream of gene PMM0806;
768634	-	TSS_018326	1000	1152	0	4	P	16nt upstream of gene PMM0810;
769201	-	TSS_018331	1000	161	0	0	O	-
771730	-	TSS_018339	1000	207	0	1	P	22nt upstream of gene PMM0814;
772207	-	TSS_018340	1000	545	0	0	O	-
773190	-	TSS_018345	1000	185	0	1	I	within gene(s) PMM0815;
773322	-	TSS_018353	1000	168	0	23	IP	within gene(s) PMM0816; 129nt upstream of gene PMM0815;
773791	-	TSS_018372	1000	139	0	0	IP	within gene(s) PMM0818; 98nt upstream of gene PMM0817;
773820	-	TSS_018373	1000	662	0	0	P	20nt upstream of gene PMM0818;
774773	-	TSS_018379	1000	6267	0	3	P	18nt upstream of gene PMM0819;
775070	-	TSS_018384	1000	163	0	1	P	49nt upstream of gene PMM0820;
775810	-	TSS_018385	1000	202	0	0	O	-
776471	-	TSS_018388	1000	234	0	4	O	-
777149	-	TSS_018394	1000	450	0	0	I	within gene(s) PMM0821;
778499	-	TSS_018398	1000	109	0	0	IP	within gene(s) PMM0824; 166nt upstream of gene PMM0823;
778555	-	TSS_018404	1000	106	0	6	IP	within gene(s) PMM0824; 222nt upstream of gene PMM0823;
778980	-	TSS_018407	1000	141	0	2	P	19nt upstream of gene PMM0824;
780730	-	TSS_018415	1000	111	0	0	Ai	antisense to gene(s) PMM0825;
781041	+	TSS_005911	1000	110	0	15	I	within gene(s) PMM0825;
784601	+	TSS_005930	1000	213	0	0	P	14nt upstream of gene PMM0829;
784835	+	TSS_005931	1000	116	0	0	I	within gene(s) PMM0829;
786463	-	TSS_018434	1000	128	0	4	I	within gene(s) PMM0831;
786571	-	TSS_018445	1000	243	0	3	I	within gene(s) PMM0831;
786988	-	TSS_018480	1000	165	0	7	I	within gene(s) PMM0831;
787168	-	TSS_018491	1000	235	0	12	I	within gene(s) PMM0831;
787885	-	TSS_018528	1000	359	0	18	I	within gene(s) PMM0831;
787909	-	TSS_018536	1000	702	0	16	I	within gene(s) PMM0831;
787995	+	TSS_005953	1000	205	0	0	Ai	antisense to gene(s) PMM0831;
788167	-	TSS_018557	1000	183	0	6	I	within gene(s) PMM0831;
788179	-	TSS_018558	1000	111	0	3	I	within gene(s) PMM0831;
788562	-	TSS_018585	1000	200	0	10	I	within gene(s) PMM0831;
788662	-	TSS_018593	1000	130	0	3	I	within gene(s) PMM0831;
788713	-	TSS_018599	1000	172	0	1	I	within gene(s) PMM0831;
788890	-	TSS_018602	1000	178	0	1	I	within gene(s) PMM0831;
788971	-	TSS_018609	1000	120	0	9	I	within gene(s) PMM0831;

789218	+	TSS_005965	1000	200	0	0 Ai	antisense to gene(s) PMM0831;
789844	-	TSS_018653	1000	194	0	2 I	within gene(s) PMM0831;
790093	-	TSS_018659	1000	106	0	9 I	within gene(s) PMM0831;
796520	+	TSS_005979	1000	478	0	1 Ai	antisense to gene(s) PMM0839;
803495	-	TSS_018701	1000	116	0	12 I	within gene(s) PMM0844;
803921	-	TSS_018715	1000	151	0	28 I	within gene(s) PMM0844;
804065	-	TSS_018738	1000	174	0	15 I	within gene(s) PMM0844;
804298	-	TSS_018752	1000	2345	0	2 P	14nt upstream of gene PMM0844;
807760	-	TSS_018764	1000	138	0	2 P	27nt upstream of gene PMM0847;
808842	+	TSS_006000	1000	4247	0	6 P	117nt upstream of gene PMM0851;
810059	-	TSS_018774	1000	158	0	0 IAd	within gene(s) PMM0853; antisense to gene(s) PMM0852 (24nt downstream);
810098	-	TSS_018777	1000	116	0	0 I	within gene(s) PMM0853;
810137	-	TSS_018778	1000	202	0	0 I	within gene(s) PMM0853;
810158	-	TSS_018782	1000	159	0	9 I	within gene(s) PMM0853;
810173	-	TSS_018786	1000	634	0	6 I	within gene(s) PMM0853;
810203	-	TSS_018788	1000	232	0	6 I	within gene(s) PMM0853;
811735	-	TSS_018794	1000	401	0	0 Ai	antisense to gene(s) PMM0854;
813130	+	TSS_006018	1000	196	0	1 Ai	antisense to gene(s) PMM0856;
813260	-	TSS_018817	1000	147	0	6 I	within gene(s) PMM0856;
813272	-	TSS_018819	1000	148	0	15 I	within gene(s) PMM0856;
813326	-	TSS_018830	1000	131	0	21 I	within gene(s) PMM0856;
813356	-	TSS_018832	1000	203	0	12 I	within gene(s) PMM0856;
813434	-	TSS_018842	1000	130	0	9 I	within gene(s) PMM0856;
813446	-	TSS_018846	1000	111	0	1 I	within gene(s) PMM0856;
813572	-	TSS_018849	1000	4557	0	12 P	45nt upstream of gene PMM0856;
813592	-	TSS_018852	1000	1278	0	1 P	65nt upstream of gene PMM0856;
814913	+	TSS_006024	1000	1390	0	1 O	-
816976	+	TSS_006029	1000	1166	0	2 O	-
817333	+	TSS_006032	1000	1276	0	3 O	-
819054	+	TSS_006040	1000	268	0	7 P	79nt upstream of gene PMM0861;
819132	+	TSS_006054	1000	586	0	15 P	1nt upstream of gene PMM0861;
819403	-	TSS_018879	1000	128	0	2 O	-
826236	-	TSS_018925	1000	434	0	7 IP	within gene(s) PMM0870; 156nt upstream of gene PMM0869;
827556	-	TSS_018930	1000	259	0	0 Ai	antisense to gene(s) PMM0871;
828957	+	TSS_006074	1000	11299	0	2 I	within gene(s) PMM0872;
832333	+	TSS_006082	1000	460	0	3 P	14nt upstream of gene PMM0876;
832360	-	TSS_018940	1000	147	0	0 PAi	122nt upstream of gene PMM0875; antisense to gene(s) PMM0876;
832529	-	TSS_018941	1000	187	0	2 Ai	antisense to gene(s) PMM0876;
834878	-	TSS_018948	1000	914	0	3 I	within gene(s) PMM0877;
836586	-	TSS_018952	1000	115	0	0 I	within gene(s) PMM0878;
836727	-	TSS_018962	1000	166	0	4 I	within gene(s) PMM0878;
837146	-	TSS_018966	1000	481	0	2 P	38nt upstream of gene PMM0878;
840244	-	TSS_018973	1000	140	0	2 Ai	antisense to gene(s) PMM0879;
841241	-	TSS_018977	1000	123	0	0 I	within gene(s) PMM0880;
842149	-	TSS_018980	1000	104	0	0 I	within gene(s) PMM0881;
844688	-	TSS_018997	1000	279	0	0 P	6nt upstream of gene PMM0883;
844697	-	TSS_018999	1000	4073	0	5 P	15nt upstream of gene PMM0883;
855371	+	TSS_006122	1000	116	0	3 I	within gene(s) PMM0893;
857390	-	TSS_019035	1000	224	0	12 I	within gene(s) PMM0894;
857431	-	TSS_019043	1000	124	0	6 I	within gene(s) PMM0894;
857485	-	TSS_019046	1000	317	0	0 I	within gene(s) PMM0894;
857549	-	TSS_019049	1000	5917	0	3 P	18nt upstream of gene PMM0894;
858604	-	TSS_019057	1000	141	0	0 I	within gene(s) PMM0896;
858648	-	TSS_019060	1000	204	0	8 I	within gene(s) PMM0896;
858686	-	TSS_019062	1000	559	0	0 I	within gene(s) PMM0896;
858957	-	TSS_019063	1000	601	0	0 IP	within gene(s) PMM0897; 21nt upstream of gene PMM0896;
859899	+	TSS_006139	1000	455	0	0 Ai	antisense to gene(s) PMM0897;
860406	-	TSS_019075	1000	1509	0	1 I	within gene(s) PMM0897;
860930	-	TSS_019078	1000	124	0	0 P	13nt upstream of gene PMM0897;
860982	-	TSS_019081	1000	254	0	2 P	65nt upstream of gene PMM0897;
862187	+	TSS_006152	1000	143	0	6 I	within gene(s) PMM0899;
862487	-	TSS_019085	1000	138	0	0 Ai	antisense to gene(s) PMM0899;
863809	+	TSS_006157	1000	2174	0	2 P	23nt upstream of gene PMM0901;
863967	+	TSS_006162	1000	277	0	4 I	within gene(s) PMM0901;
865761	+	TSS_006187	1000	426	0	1 P	15nt upstream of gene PMM0902;
865990	-	TSS_019095	1000	157	0	0 Ai	antisense to gene(s) PMM0902;
865997	-	TSS_019096	1000	121	0	0 Ai	antisense to gene(s) PMM0902;
867262	+	TSS_006189	1000	163	0	18 I	within gene(s) PMM0906;
867288	+	TSS_006196	1000	1246	0	1 I	within gene(s) PMM0906;
867467	-	TSS_019099	1000	185	0	0 Ai	antisense to gene(s) PMM0906;
867687	+	TSS_006204	1000	140	0	0 Ai	antisense to gene(s) PMM0907;
867708	+	TSS_006205	1000	128	0	0 Ai	antisense to gene(s) PMM0907;
867992	-	TSS_019108	1000	108	0	1 I	within gene(s) PMM0907;
869043	+	TSS_006212	1000	101	0	0 Ai	antisense to gene(s) PMM0907;
869052	+	TSS_006214	1000	242	0	7 Ai	antisense to gene(s) PMM0907;
869447	-	TSS_019140	1000	7656	0	5 P	16nt upstream of gene PMM0907;
869708	+	TSS_006221	1000	149	0	0 I	within gene(s) PMM0908;
870149	+	TSS_006225	1000	494	0	2 I	within gene(s) PMM0908;
873315	+	TSS_006251	1000	102	0	0 I	within gene(s) PMM0912;

873612	+	TSS_006256	1000	462	0	0	I	within gene(s) PMM0912;
881167	-	TSS_019172	1000	1103	0	2	P	15nt upstream of gene PMM0919;
881330	+	TSS_006279	1000	226	0	1	P	37nt upstream of gene PMM0920;
881367	+	TSS_006280	1000	127	0	0	P	0nt upstream of gene PMM0920;
881514	+	TSS_006287	1000	178	0	3	I	within gene(s) PMM0920;
881640	+	TSS_006301	1000	189	0	17	I	within gene(s) PMM0920;
881661	+	TSS_006306	1000	148	0	33	I	within gene(s) PMM0920;
881697	+	TSS_006315	1000	148	0	9	I	within gene(s) PMM0920;
881724	+	TSS_006320	1000	127	0	17	I	within gene(s) PMM0920;
881760	+	TSS_006324	1000	153	0	17	I	within gene(s) PMM0920;
881833	+	TSS_006334	1000	214	0	4	I	within gene(s) PMM0920;
881871	+	TSS_006341	1000	343	0	27	I	within gene(s) PMM0920;
881904	+	TSS_006344	1000	203	0	0	I	within gene(s) PMM0920;
881949	+	TSS_006351	1000	131	0	12	I	within gene(s) PMM0920;
882006	+	TSS_006361	1000	206	0	27	I	within gene(s) PMM0920;
882039	+	TSS_006368	1000	126	0	1	I	within gene(s) PMM0920;
882142	+	TSS_006383	1000	125	0	30	I	within gene(s) PMM0920;
882186	-	TSS_019184	1000	400	0	0	Ai	antisense to gene(s) PMM0920;
882219	-	TSS_019186	1000	280	0	1	Ai	antisense to gene(s) PMM0920;
882256	-	TSS_019187	1000	132	0	5	Ai	antisense to gene(s) PMM0920;
882363	+	TSS_006409	1000	106	0	3	I	within gene(s) PMM0920;
882426	+	TSS_006416	1000	253	0	12	I	within gene(s) PMM0920;
882438	+	TSS_006418	1000	106	0	3	I	within gene(s) PMM0920;
882462	+	TSS_006422	1000	134	0	18	I	within gene(s) PMM0920;
882489	+	TSS_006429	1000	196	0	9	I	within gene(s) PMM0920;
882673	-	TSS_019193	1000	186	0	5	Ai	antisense to gene(s) PMM0920;
886557	+	TSS_006463	1000	1306	0	7	P	14nt upstream of gene PMM0926;
886578	+	TSS_006466	1000	146	0	4	I	within gene(s) PMM0926;
889213	+	TSS_006469	1000	210	0	0	Ai	antisense to gene(s) PMM0929;
890971	+	TSS_006473	1000	104	0	0	Ai	antisense to gene(s) PMM0930;
891212	+	TSS_006480	1000	229	0	1	Ai	antisense to gene(s) PMM0930;
891599	-	TSS_019242	1000	3369	0	1	P	21nt upstream of gene PMM0930;
894754	+	TSS_006491	1000	103	0	0	P	45nt upstream of gene PMM0936;
899977	+	TSS_006504	1000	744	0	3	P	13nt upstream of gene PMM0941;
900041	+	TSS_006505	1000	753	0	1	I	within gene(s) PMM0941;
900053	+	TSS_006507	1000	449	0	0	I	within gene(s) PMM0941;
900080	+	TSS_006510	1000	158	0	12	I	within gene(s) PMM0941;
900096	+	TSS_006514	1000	275	0	1	I	within gene(s) PMM0941;
900199	-	TSS_019253	1000	223	0	4	Ai	antisense to gene(s) PMM0941;
900229	-	TSS_019257	1000	260	0	0	Ai	antisense to gene(s) PMM0941;
900245	+	TSS_006525	1000	292	0	15	IP	within gene(s) PMM0941; 105nt upstream of gene PMM0942;
901047	+	TSS_006536	1000	25028	0	9	P	14nt upstream of gene PMM0943;
901085	+	TSS_006538	1000	152	0	0	I	within gene(s) PMM0943;
901115	+	TSS_006539	1000	190	0	1	IP	within gene(s) PMM0943; 225nt upstream of gene PMM0944;
901142	+	TSS_006542	1000	105	0	0	IP	within gene(s) PMM0943; 198nt upstream of gene PMM0944;
905299	-	TSS_019274	1000	1149	0	2	P	14nt upstream of gene PMM0945;
906542	-	TSS_019293	1000	167	0	0	I	within gene(s) PMM0946;
907099	-	TSS_019296	1000	1089	0	2	P	6nt upstream of gene PMM0947;
913344	+	TSS_006564	1000	311	0	0	I	within gene(s) PMM0954;
913440	+	TSS_006565	1000	310	0	0	IP	within gene(s) PMM0954; 246nt upstream of gene PMM0955;
913823	+	TSS_006568	1000	234	0	3	I	within gene(s) PMM0955;
914442	+	TSS_006570	1000	275	0	1	O	-
914689	-	TSS_019319	1000	390	0	1	O	-
915624	+	TSS_006575	1000	240	0	2	P	2nt upstream of gene PMM0957;
916151	-	TSS_019326	1000	109	0	0	O	-
916169	-	TSS_019327	1000	262	0	0	O	-
916221	-	TSS_019328	1000	206	0	2	O	-
916530	-	TSS_019330	1000	519	0	1	P	16nt upstream of gene PMM0958;
918232	+	TSS_006581	1000	173	0	0	Ai	antisense to gene(s) PMM0960;
919141	-	TSS_019336	1000	149	0	0	I	within gene(s) PMM0961;
920682	-	TSS_019338	1000	139	0	0	Ai	antisense to gene(s) PMM0962;
922689	+	TSS_006603	1000	170	0	2	Ai	antisense to gene(s) PMM0963;
923723	-	TSS_019354	1000	553	0	0	I	within gene(s) PMM0965;
923809	-	TSS_019356	1000	130	0	4	IP	within gene(s) PMM0966; 85nt upstream of gene PMM0965;
924547	-	TSS_019359	1000	591	0	0	I	within gene(s) PMM0966;
926578	+	TSS_006618	1000	136	0	0	I	within gene(s) PMM0970;
926625	+	TSS_006619	1000	205	0	0	I	within gene(s) PMM0970;
926661	+	TSS_006626	1000	357	0	25	I	within gene(s) PMM0970;
926710	-	TSS_019363	1000	151	0	0	Ai	antisense to gene(s) PMM0970;
926805	+	TSS_006637	1000	175	0	15	I	within gene(s) PMM0970;
926853	+	TSS_006640	1000	170	0	3	I	within gene(s) PMM0970;
926913	+	TSS_006649	1000	171	0	42	I	within gene(s) PMM0970;
926952	+	TSS_006658	1000	243	0	0	I	within gene(s) PMM0970;
927021	+	TSS_006664	1000	122	0	12	I	within gene(s) PMM0970;
927117	+	TSS_006682	1000	128	0	6	I	within gene(s) PMM0970;
927237	+	TSS_006688	1000	120	0	8	I	within gene(s) PMM0970;
927259	+	TSS_006691	1000	188	0	0	I	within gene(s) PMM0970;
927291	+	TSS_006694	1000	149	0	3	I	within gene(s) PMM0970;
927369	+	TSS_006706	1000	163	0	18	I	within gene(s) PMM0970;

927379	+	TSS_006708	1000	151	0	2	I	within gene(s) PMM0970;
927405	+	TSS_006712	1000	190	0	1	I	within gene(s) PMM0970;
927417	+	TSS_006714	1000	108	0	0	I	within gene(s) PMM0970;
927442	+	TSS_006719	1000	586	0	18	I	within gene(s) PMM0970;
927568	-	TSS_019368	1000	924	0	0	Ai	antisense to gene(s) PMM0970;
927597	-	TSS_019369	1000	134	0	0	Ai	antisense to gene(s) PMM0970;
927626	-	TSS_019371	1000	181	0	0	Ai	antisense to gene(s) PMM0970;
927755	-	TSS_019374	1000	102	0	0	Ai	antisense to gene(s) PMM0970;
929465	-	TSS_019380	1000	132	0	1	Ai	antisense to gene(s) PMM0972;
930117	+	TSS_006741	1000	131	0	0	IP	within gene(s) PMM0972; 135nt upstream of gene PMM0973;
930151	+	TSS_006742	1000	233	0	1	IP	within gene(s) PMM0972; 101nt upstream of gene PMM0973;
930208	-	TSS_019384	1000	623	0	2	Ai	antisense to gene(s) PMM0972;
932988	+	TSS_006751	1000	104	0	2	P	2nt upstream of gene PMM0975;
933017	+	TSS_006752	1000	351	0	0	I	within gene(s) PMM0975;
936929	+	TSS_006756	1000	475	0	1	O	-
937963	+	TSS_006759	1000	1239	0	1	P	15nt upstream of gene PMM0982;
939028	-	TSS_019397	1000	181	0	0	P	17nt upstream of gene PMM0983;
939290	-	TSS_019398	1000	187	0	0	O	-
941587	+	TSS_006765	1000	388	0	6	P	17nt upstream of gene PMM0987;
942377	-	TSS_019402	1000	287	0	0	P	126nt upstream of gene PMM0988;
945784	+	TSS_006773	1000	913	0	3	P	20nt upstream of gene PMM0992;
946441	+	TSS_006775	1000	317	0	0	Ai	antisense to gene(s) PMM0993;
946927	-	TSS_019408	1000	831	0	4	P	16nt upstream of gene PMM0993;
949329	-	TSS_019422	1000	10118	0	2	O	-
950002	+	TSS_006783	1000	318	0	0	P	15nt upstream of gene PMM0999;
950681	+	TSS_006784	1000	127	0	0	P	15nt upstream of gene PMM1001;
956897	-	TSS_019435	1000	2117	0	3	P	17nt upstream of gene PMM1005;
957665	+	TSS_006793	1000	137	0	0	Ai	antisense to gene(s) PMM1007;
957700	+	TSS_006796	1000	226	0	4	Ai	antisense to gene(s) PMM1007;
957713	+	TSS_006799	1000	148	0	3	Ai	antisense to gene(s) PMM1007;
957732	-	TSS_019441	1000	238	0	150	I	within gene(s) PMM1007;
957751	+	TSS_006803	1000	226	0	4	Ai	antisense to gene(s) PMM1007;
957764	+	TSS_006806	1000	148	0	3	Ai	antisense to gene(s) PMM1007;
957802	+	TSS_006810	1000	226	0	4	Ai	antisense to gene(s) PMM1007;
957815	+	TSS_006813	1000	148	0	0	Ai	antisense to gene(s) PMM1007;
957885	-	TSS_019487	1000	240	0	0	I	within gene(s) PMM1007;
957904	+	TSS_006816	1000	226	0	4	Ai	antisense to gene(s) PMM1007;
957917	+	TSS_006819	1000	148	0	3	Ai	antisense to gene(s) PMM1007;
957955	+	TSS_006823	1000	226	0	4	Ai	antisense to gene(s) PMM1007;
957968	+	TSS_006826	1000	148	0	3	Ai	antisense to gene(s) PMM1007;
957987	-	TSS_019503	1000	238	0	135	I	within gene(s) PMM1007;
958006	+	TSS_006830	1000	226	0	16	Ai	antisense to gene(s) PMM1007;
958089	-	TSS_019533	1000	240	0	12	I	within gene(s) PMM1007;
958314	-	TSS_019543	1000	172	0	0	P	15nt upstream of gene PMM1007;
960692	-	TSS_019548	1000	159	0	2	P	16nt upstream of gene PMM1011;
961818	+	TSS_006844	1000	358	0	3	P	14nt upstream of gene PMM1013;
965105	+	TSS_006852	1000	312	0	0	O	-
971676	-	TSS_019563	1000	220	0	0	P	4nt upstream of gene PMM1026;
972175	-	TSS_019564	1000	1054	0	2	O	-
972740	+	TSS_006873	1000	413	0	0	P	166nt upstream of gene PMM1028;
973076	-	TSS_019568	1000	257	0	0	Ai	antisense to gene(s) PMM1028;
973232	+	TSS_006877	1000	1174	0	0	O	-
974212	+	TSS_006881	1000	429	0	2	P	36nt upstream of gene PMM1030;
974221	+	TSS_006882	1000	1393	0	1	P	27nt upstream of gene PMM1030;
975550	+	TSS_006890	1000	139	0	0	P	17nt upstream of gene PMM1032;
977043	+	TSS_006918	1000	1054	0	4	P	32nt upstream of gene PMM1033;
977685	-	TSS_019581	1000	102	0	0	Ai	antisense to gene(s) PMM1033;
978424	+	TSS_006960	1000	127	0	0	I	within gene(s) PMM1033 PMM1034;
979706	-	TSS_019594	1000	118	0	1	O	-
981600	+	TSS_006968	1000	109	0	0	I	within gene(s) PMM1038;
984870	+	TSS_006981	1000	285	0	4	I	within gene(s) PMM1041;
985398	+	TSS_006982	1000	421	0	0	IP	within gene(s) PMM1041; 91nt upstream of gene PMM1042;
985474	+	TSS_006987	1000	4489	0	7	P	15nt upstream of gene PMM1042;
985522	+	TSS_006988	1000	560	0	0	I	within gene(s) PMM1042;
985651	+	TSS_006989	1000	246	0	0	I	within gene(s) PMM1042;
995402	+	TSS_007000	1000	104	0	0	Ad	antisense to gene(s) PMM1053 (3nt downstream);
996144	-	TSS_019620	1000	314	0	0	IP	within gene(s) PMM1054; 183nt upstream of gene PMM1053;
996203	-	TSS_019622	1000	101	0	0	IP	within gene(s) PMM1054; 242nt upstream of gene PMM1053;
997065	-	TSS_019632	1000	145	0	1	I	within gene(s) PMM1055;
998966	+	TSS_007010	1000	1564	0	2	P	23nt upstream of gene PMM1058;
1000663	-	TSS_019650	1000	843	0	2	P	16nt upstream of gene PMM1061;
1000796	-	TSS_019653	1000	13371	0	3	P	149nt upstream of gene PMM1061;
1000995	-	TSS_019655	1000	262	0	1	I	within gene(s) PMM1062;
1001106	+	TSS_007017	1000	609	0	0	Ai	antisense to gene(s) PMM1062;
1002186	+	TSS_007023	1000	971	0	8	P	13nt upstream of gene PMM1063;
1004709	+	TSS_007051	1000	101	0	9	IP	within gene(s) PMM1063; 118nt upstream of gene PMM1064;
1008963	+	TSS_007084	1000	529	0	0	I	within gene(s) PMM1066;
1009566	+	TSS_007103	1000	143	0	0	P	29nt upstream of gene PMM1067;
1009601	+	TSS_007105	1000	128	0	3	I	within gene(s) PMM1067;

1011515	+	TSS_007111	1000	130	0	0	P	31nt upstream of gene PMM1069;
1012301	+	TSS_007119	1000	305	0	0	IP	within gene(s) PMM1069; 179nt upstream of gene PMM1070;
1013805	-	TSS_019690	1000	176	0	0	Ai	antisense to gene(s) PMM1071;
1013842	-	TSS_019691	1000	123	0	0	Ai	antisense to gene(s) PMM1071;
1018128	-	TSS_019714	1000	1079	0	0	P	18nt upstream of gene PMM1074;
1018304	+	TSS_007142	1000	403	0	1	Ai	antisense to gene(s) PMM1075;
1018315	+	TSS_007146	1000	182	0	8	Ai	antisense to gene(s) PMM1075;
1018329	+	TSS_007149	1000	113	0	0	Ai	antisense to gene(s) PMM1075;
1018424	-	TSS_019721	1000	390	0	4	I	within gene(s) PMM1075;
1018453	+	TSS_007152	1000	148	0	1	Ai	antisense to gene(s) PMM1075;
1018466	-	TSS_019726	1000	137	0	6	I	within gene(s) PMM1075;
1018469	+	TSS_007153	1000	335	0	0	Ai	antisense to gene(s) PMM1075;
1018476	+	TSS_007154	1000	183	0	0	Ai	antisense to gene(s) PMM1075;
1018484	-	TSS_019728	1000	101	0	3	I	within gene(s) PMM1075;
1018496	-	TSS_019730	1000	101	0	3	I	within gene(s) PMM1075;
1018534	+	TSS_007155	1000	119	0	0	Ai	antisense to gene(s) PMM1075;
1018598	+	TSS_007158	1000	717	0	3	Ai	antisense to gene(s) PMM1075;
1018619	-	TSS_019744	1000	138	0	12	I	within gene(s) PMM1075;
1018629	+	TSS_007160	1000	134	0	4	Ai	antisense to gene(s) PMM1075;
1018655	-	TSS_019748	1000	1853	0	1	I	within gene(s) PMM1075;
1018673	-	TSS_019751	1000	563	0	12	I	within gene(s) PMM1075;
1018700	-	TSS_019754	1000	113	0	6	I	within gene(s) PMM1075;
1018735	-	TSS_019761	1000	443	0	23	I	within gene(s) PMM1075;
1018757	-	TSS_019766	1000	1526	0	15	I	within gene(s) PMM1075;
1018758	+	TSS_007163	1000	166	0	1	Ai	antisense to gene(s) PMM1075;
1018769	-	TSS_019768	1000	235	0	0	I	within gene(s) PMM1075;
1018796	-	TSS_019773	1000	315	0	30	I	within gene(s) PMM1075;
1018832	-	TSS_019781	1000	178	0	6	I	within gene(s) PMM1075;
1018856	-	TSS_019785	1000	512	0	12	I	within gene(s) PMM1075;
1018865	-	TSS_019786	1000	119	0	0	I	within gene(s) PMM1075;
1018895	-	TSS_019790	1000	109	0	15	I	within gene(s) PMM1075;
1018949	-	TSS_019803	1000	273	0	67	I	within gene(s) PMM1075;
1018979	-	TSS_019809	1000	204	0	6	I	within gene(s) PMM1075;
1019132	-	TSS_019821	1000	104	0	0	I	within gene(s) PMM1075;
1019153	-	TSS_019823	1000	511	0	12	I	within gene(s) PMM1075;
1019195	-	TSS_019830	1000	338	0	18	I	within gene(s) PMM1075;
1019219	-	TSS_019832	1000	772	0	0	I	within gene(s) PMM1075;
1019366	-	TSS_019833	1000	1178	0	0	P	30nt upstream of gene PMM1075;
1020266	+	TSS_007170	1000	296	0	0	Ai	antisense to gene(s) PMM1077;
1020379	-	TSS_019839	1000	3267	0	2	I	within gene(s) PMM1077;
1020982	-	TSS_019844	1000	183	0	1	I	within gene(s) PMM1077;
1021282	-	TSS_019845	1000	340	0	0	I	within gene(s) PMM1077;
1022015	+	TSS_007175	1000	204	0	4	P	18nt upstream of gene PMM1079;
1022762	-	TSS_019850	1000	260	0	0	I	within gene(s) PMM1080;
1022836	+	TSS_007180	1000	109	0	0	Ai	antisense to gene(s) PMM1080;
1022879	+	TSS_007181	1000	166	0	0	Ai	antisense to gene(s) PMM1080;
1022993	-	TSS_019866	1000	166	0	27	I	within gene(s) PMM1080;
1023018	-	TSS_019870	1000	207	0	5	I	within gene(s) PMM1080;
1023140	+	TSS_007186	1000	219	0	6	Ai	antisense to gene(s) PMM1080;
1023402	-	TSS_019885	1000	151	0	0	I	within gene(s) PMM1080;
1023493	-	TSS_019889	1000	2030	0	1	P	19nt upstream of gene PMM1080;
1024480	+	TSS_007197	1000	289	0	10	I	within gene(s) PMM1081;
1024757	-	TSS_019891	1000	170	0	0	Ai	antisense to gene(s) PMM1081;
1029223	-	TSS_019902	1000	177	0	0	Ai	antisense to gene(s) PMM1085;
1029801	-	TSS_019903	1000	272	0	0	Ai	antisense to gene(s) PMM1085;
1032141	+	TSS_007211	1000	505	0	0	Ai	antisense to gene(s) PMM1088;
1032155	+	TSS_007212	1000	1041	0	1	Ai	antisense to gene(s) PMM1088;
1032254	-	TSS_019934	1000	231	0	15	IP	within gene(s) PMM1088; 245nt upstream of gene PMM1087;
1032281	-	TSS_019940	1000	160	0	10	I	within gene(s) PMM1088;
1032308	-	TSS_019946	1000	239	0	27	I	within gene(s) PMM1088;
1032374	-	TSS_019958	1000	136	0	24	I	within gene(s) PMM1088;
1032392	-	TSS_019962	1000	140	0	5	I	within gene(s) PMM1088;
1032423	+	TSS_007216	1000	347	0	0	Ai	antisense to gene(s) PMM1088;
1032451	+	TSS_007217	1000	132	0	0	Ai	antisense to gene(s) PMM1088;
1032479	+	TSS_007219	1000	149	0	4	Ai	antisense to gene(s) PMM1088;
1032608	+	TSS_007223	1000	148	0	0	Ai	antisense to gene(s) PMM1088;
1032676	-	TSS_019982	1000	110	0	5	I	within gene(s) PMM1088;
1032686	-	TSS_019984	1000	191	0	4	I	within gene(s) PMM1088;
1032714	+	TSS_007226	1000	239	0	0	Ai	antisense to gene(s) PMM1088;
1032743	-	TSS_020001	1000	176	0	46	I	within gene(s) PMM1088;
1032755	-	TSS_020003	1000	148	0	9	I	within gene(s) PMM1088;
1032779	-	TSS_020009	1000	209	0	9	I	within gene(s) PMM1088;
1032803	-	TSS_020015	1000	216	0	18	I	within gene(s) PMM1088;
1032827	-	TSS_020019	1000	524	0	3	I	within gene(s) PMM1088;
1032845	-	TSS_020021	1000	393	0	0	I	within gene(s) PMM1088;
1032863	-	TSS_020023	1000	250	0	18	I	within gene(s) PMM1088;
1032902	-	TSS_020031	1000	140	0	12	I	within gene(s) PMM1088;
1032953	-	TSS_020038	1000	236	0	3	I	within gene(s) PMM1088;
1033079	-	TSS_020049	1000	138	0	6	I	within gene(s) PMM1088;

1033127	-	TSS_020057	1000	123	0	33	I	within gene(s) PMM1088;
1033178	-	TSS_020068	1000	162	0	30	I	within gene(s) PMM1088;
1033268	+	TSS_007234	1000	115	0	0	Ai	antisense to gene(s) PMM1088;
1033286	-	TSS_020077	1000	155	0	0	I	within gene(s) PMM1088;
1033346	-	TSS_020085	1000	359	0	24	I	within gene(s) PMM1088;
1033361	-	TSS_020088	1000	149	0	6	I	within gene(s) PMM1088;
1033430	-	TSS_020107	1000	537	0	78	I	within gene(s) PMM1088;
1033472	-	TSS_020114	1000	349	0	12	I	within gene(s) PMM1088;
1033493	-	TSS_020118	1000	446	0	0	I	within gene(s) PMM1088;
1033547	-	TSS_020123	1000	210	0	12	I	within gene(s) PMM1088;
1033637	-	TSS_020131	1000	125	0	12	I	within gene(s) PMM1088;
1033667	-	TSS_020136	1000	252	0	6	I	within gene(s) PMM1088;
1033688	-	TSS_020139	1000	166	0	9	I	within gene(s) PMM1088;
1033787	+	TSS_007237	1000	119	0	0	Ai	antisense to gene(s) PMM1088;
1033787	-	TSS_020149	1000	171	0	15	I	within gene(s) PMM1088;
1033806	+	TSS_007238	1000	128	0	0	Ai	antisense to gene(s) PMM1088;
1033847	-	TSS_020167	1000	272	0	54	I	within gene(s) PMM1088;
1033868	-	TSS_020171	1000	450	0	7	I	within gene(s) PMM1088;
1033904	-	TSS_020178	1000	198	0	21	I	within gene(s) PMM1088;
1033940	-	TSS_020186	1000	719	0	21	I	within gene(s) PMM1088;
1033961	-	TSS_020189	1000	175	0	0	I	within gene(s) PMM1088;
1034009	-	TSS_020191	1000	201	0	6	I	within gene(s) PMM1088;
1034030	-	TSS_020194	1000	117	0	9	I	within gene(s) PMM1088;
1034052	+	TSS_007240	1000	110	0	0	Ai	antisense to gene(s) PMM1088;
1034057	-	TSS_020197	1000	105	0	3	I	within gene(s) PMM1088;
1034120	-	TSS_020203	1000	211	0	19	I	within gene(s) PMM1088;
1034144	-	TSS_020208	1000	157	0	12	I	within gene(s) PMM1088;
1034168	-	TSS_020212	1000	115	0	12	I	within gene(s) PMM1088;
1034221	+	TSS_007248	1000	227	0	13	Ai	antisense to gene(s) PMM1088;
1034242	+	TSS_007250	1000	201	0	0	Ai	antisense to gene(s) PMM1088;
1034242	-	TSS_020219	1000	592	0	1	I	within gene(s) PMM1088;
1034266	+	TSS_007252	1000	105	0	0	Ai	antisense to gene(s) PMM1088;
1034282	-	TSS_020225	1000	134	0	12	I	within gene(s) PMM1088;
1034297	-	TSS_020227	1000	335	0	13	I	within gene(s) PMM1088;
1034333	-	TSS_020233	1000	173	0	3	I	within gene(s) PMM1088;
1034343	+	TSS_007254	1000	188	0	0	Ai	antisense to gene(s) PMM1088;
1034369	-	TSS_020240	1000	340	0	33	I	within gene(s) PMM1088;
1034380	+	TSS_007258	1000	400	0	4	Ai	antisense to gene(s) PMM1088;
1034390	-	TSS_020243	1000	311	0	9	I	within gene(s) PMM1088;
1034423	-	TSS_020254	1000	486	0	30	I	within gene(s) PMM1088;
1034447	-	TSS_020260	1000	240	0	0	I	within gene(s) PMM1088;
1034467	-	TSS_020261	1000	220	0	1	I	within gene(s) PMM1088;
1034492	-	TSS_020263	1000	134	0	0	I	within gene(s) PMM1088;
1034507	-	TSS_020265	1000	280	0	9	I	within gene(s) PMM1088;
1035252	+	TSS_007260	1000	628	0	1	P	14nt upstream of gene PMM1090;
1036076	+	TSS_007272	1000	611	0	7	I	within gene(s) PMM1090;
1037521	+	TSS_007286	1000	147	0	0	IP	within gene(s) PMM1091; 41nt upstream of gene PMM1092;
1037579	-	TSS_020276	1000	412	0	0	Ai	antisense to gene(s) PMM1092;
1038879	-	TSS_020281	1000	156	0	0	Ai	antisense to gene(s) PMM1093;
1039004	+	TSS_007298	1000	170	0	2	I	within gene(s) PMM1093;
1041219	+	TSS_007306	1000	129	0	0	Ai	antisense to gene(s) PMM1096;
1041770	+	TSS_007307	1000	804	0	1	Ai	antisense to gene(s) PMM1097;
1042334	-	TSS_020287	1000	231	0	0	P	14nt upstream of gene PMM1097;
1042406	+	TSS_007312	1000	3259	0	1	P	20nt upstream of gene PMM1098;
1042483	+	TSS_007317	1000	107	0	9	I	within gene(s) PMM1098;
1042908	-	TSS_020293	1000	181	0	1	Ai	antisense to gene(s) PMM1098;
1043114	+	TSS_007337	1000	133	0	0	Ai	antisense to gene(s) PMM1099;
1057074	-	TSS_020304	1000	2912	0	1	P	35nt upstream of gene PMM1107;
1057110	-	TSS_020306	1000	166	0	0	P	71nt upstream of gene PMM1107;
1058110	+	TSS_007360	1000	156	0	1	I	within gene(s) PMM1109;
1058522	+	TSS_007366	1000	689	0	3	IP	within gene(s) PMM1110; 95nt upstream of gene PMM1111;
1059299	+	TSS_007373	1000	651	0	2	P	47nt upstream of gene PMM1113;
1059838	+	TSS_007383	1000	116	0	2	I	within gene(s) PMM1113;
1059858	+	TSS_007385	1000	422	0	2	I	within gene(s) PMM1113;
1063653	-	TSS_020334	1000	106	0	9	IP	within gene(s) PMM1117; 84nt upstream of gene PMM1116;
1063680	-	TSS_020336	1000	119	0	4	IP	within gene(s) PMM1117; 111nt upstream of gene PMM1116;
1063850	+	TSS_007403	1000	185	0	4	O	-
1064477	+	TSS_007404	1000	130	0	0	P	24nt upstream of gene PMM1118;
1065133	+	TSS_007407	1000	116	0	0	Ai	antisense to gene(s) PMM1119;
1065289	+	TSS_007411	1000	749	0	0	Ai	antisense to gene(s) PMM1119;
1065304	+	TSS_007412	1000	1804	0	0	Ai	antisense to gene(s) PMM1119;
1065312	+	TSS_007413	1000	491	0	0	Ai	antisense to gene(s) PMM1119;
1065322	+	TSS_007414	1000	129	0	0	Ai	antisense to gene(s) PMM1119;
1065335	+	TSS_007415	1000	113	0	0	Ai	antisense to gene(s) PMM1119;
1065409	+	TSS_007417	1000	523	0	5	Ai	antisense to gene(s) PMM1119;
1065446	+	TSS_007421	1000	287	0	0	Ai	antisense to gene(s) PMM1119;
1065544	+	TSS_007422	1000	132	0	0	Ai	antisense to gene(s) PMM1119;
1065751	+	TSS_007424	1000	249	0	0	Ai	antisense to gene(s) PMM1119;
1068349	+	TSS_007435	1000	116	0	0	Ai	antisense to gene(s) PMM1121;

1068501	-	TSS_020346	1000	652	0	3 I	within gene(s) PMM1121;
1068505	+	TSS_007440	1000	749	0	0 Ai	antisense to gene(s) PMM1121;
1068519	-	TSS_020348	1000	1699	0	15 I	within gene(s) PMM1121;
1068520	+	TSS_007441	1000	1804	0	0 Ai	antisense to gene(s) PMM1121;
1068528	+	TSS_007442	1000	491	0	0 Ai	antisense to gene(s) PMM1121;
1068538	+	TSS_007443	1000	129	0	0 Ai	antisense to gene(s) PMM1121;
1068551	+	TSS_007444	1000	113	0	0 Ai	antisense to gene(s) PMM1121;
1068552	-	TSS_020353	1000	395	0	3 I	within gene(s) PMM1121;
1068564	+	TSS_007445	1000	138	0	0 Ai	antisense to gene(s) PMM1121;
1068570	-	TSS_020357	1000	1511	0	9 I	within gene(s) PMM1121;
1068588	-	TSS_020360	1000	478	0	3 I	within gene(s) PMM1121;
1068603	-	TSS_020363	1000	2418	0	20 I	within gene(s) PMM1121;
1068625	+	TSS_007449	1000	523	0	13 Ai	antisense to gene(s) PMM1121;
1068645	-	TSS_020368	1000	324	0	0 I	within gene(s) PMM1121;
1068662	+	TSS_007453	1000	287	0	0 Ai	antisense to gene(s) PMM1121;
1068666	-	TSS_020374	1000	2059	0	26 I	within gene(s) PMM1121;
1068693	-	TSS_020381	1000	599	0	12 I	within gene(s) PMM1121;
1068760	+	TSS_007454	1000	132	0	0 Ai	antisense to gene(s) PMM1121;
1068967	+	TSS_007456	1000	249	0	0 Ai	antisense to gene(s) PMM1121;
1069050	-	TSS_020387	1000	1845	0	18 I	within gene(s) PMM1121;
1069074	-	TSS_020391	1000	1030	0	48 I	within gene(s) PMM1121;
1069166	+	TSS_007461	1000	140	0	1 Ai	antisense to gene(s) PMM1121;
1069167	-	TSS_020421	1000	802	0	36 I	within gene(s) PMM1121;
1069179	-	TSS_020423	1000	636	0	3 I	within gene(s) PMM1121;
1069197	-	TSS_020427	1000	1097	0	18 I	within gene(s) PMM1121;
1069204	+	TSS_007463	1000	128	0	0 Ai	antisense to gene(s) PMM1121;
1069227	-	TSS_020433	1000	263	0	0 I	within gene(s) PMM1121;
1069243	+	TSS_007465	1000	1192	0	1 Ai	antisense to gene(s) PMM1121;
1069251	-	TSS_020434	1000	629	0	3 I	within gene(s) PMM1121;
1069261	+	TSS_007466	1000	279	0	1 Ai	antisense to gene(s) PMM1121;
1069269	-	TSS_020438	1000	410	0	3 I	within gene(s) PMM1121;
1069296	-	TSS_020441	1000	126	0	12 I	within gene(s) PMM1121;
1069339	+	TSS_007468	1000	276	0	0 Ai	antisense to gene(s) PMM1121;
1069447	-	TSS_020442	1000	1914	0	2 P	28nt upstream of gene PMM1121;
1071728	+	TSS_007475	1000	810	0	0 Ai	antisense to gene(s) PMM1123;
1071742	-	TSS_020460	1000	105	0	0 I	within gene(s) PMM1123;
1071763	-	TSS_020463	1000	124	0	10 I	within gene(s) PMM1123;
1071771	+	TSS_007476	1000	110	0	3 Ai	antisense to gene(s) PMM1123;
1071780	-	TSS_020466	1000	596	0	0 I	within gene(s) PMM1123;
1071792	-	TSS_020467	1000	142	0	0 I	within gene(s) PMM1123;
1071805	-	TSS_020469	1000	295	0	20 I	within gene(s) PMM1123;
1071856	-	TSS_020477	1000	117	0	0 I	within gene(s) PMM1123;
1071889	-	TSS_020482	1000	129	0	24 I	within gene(s) PMM1123;
1071928	-	TSS_020488	1000	709	0	16 I	within gene(s) PMM1123;
1071975	-	TSS_020498	1000	183	0	12 I	within gene(s) PMM1123;
1072021	-	TSS_020503	1000	152	0	12 I	within gene(s) PMM1123;
1072087	-	TSS_020508	1000	249	0	1 I	within gene(s) PMM1123;
1072132	-	TSS_020515	1000	560	0	30 I	within gene(s) PMM1123;
1072161	-	TSS_020525	1000	109	0	2 I	within gene(s) PMM1123;
1072179	-	TSS_020527	1000	154	0	3 I	within gene(s) PMM1123;
1072201	-	TSS_020532	1000	950	0	21 I	within gene(s) PMM1123;
1072334	-	TSS_020540	1000	141	0	1 I	within gene(s) PMM1123;
1072352	-	TSS_020543	1000	342	0	6 I	within gene(s) PMM1123;
1072364	+	TSS_007478	1000	249	0	0 Ai	antisense to gene(s) PMM1123;
1072388	-	TSS_020548	1000	134	0	6 P	13nt upstream of gene PMM1123;
1074287	-	TSS_020555	1000	634	0	1 I	within gene(s) PMM1124;
1078286	-	TSS_020560	1000	784	0	2 I	within gene(s) PMM1127;
1078972	-	TSS_020563	1000	110	0	0 O	-
1079462	-	TSS_020565	1000	1897	0	3 P	17nt upstream of gene PMM1128;
1080845	+	TSS_007500	1000	192	0	1 P	24nt upstream of gene PMM1131;
1082063	-	TSS_020572	1000	2016	0	3 O	-
1082218	+	TSS_007509	1000	2110	0	4 P	51nt upstream of gene PMM1132;
1083365	-	TSS_020576	1000	201	0	1 Ai	antisense to gene(s) PMM1132;
1083716	+	TSS_007520	1000	122	0	0 I	within gene(s) PMM1132;
1085043	+	TSS_007523	1000	2727	0	1 P	19nt upstream of gene PMM1134;
1085641	+	TSS_007530	1000	4065	0	4 P	15nt upstream of gene PMM1135;
1087615	+	TSS_007541	1000	500	0	0 O	-
1090775	+	TSS_007546	1000	395	0	0 I	within gene(s) PMM1140;
1094754	+	TSS_007555	1000	477	0	1 P	29nt upstream of gene PMM1142;
1095730	+	TSS_007561	1000	217	0	0 I	within gene(s) PMM1142;
1098755	-	TSS_020628	1000	126	0	2 I	within gene(s) PMM1145;
1099273	-	TSS_020634	1000	225	0	0 IP	within gene(s) PMM1147; 96nt upstream of gene PMM1146;
1100340	-	TSS_020654	1000	1394	0	7 P	17nt upstream of gene PMM1147;
1100440	+	TSS_007578	1000	611	0	1 P	16nt upstream of gene PMM1148;
1100588	+	TSS_007582	1000	111	0	0 I	within gene(s) PMM1148;
1100603	+	TSS_007584	1000	258	0	3 I	within gene(s) PMM1148;
1100633	+	TSS_007589	1000	249	0	40 I	within gene(s) PMM1148;
1100672	+	TSS_007599	1000	110	0	0 I	within gene(s) PMM1148;
1100708	+	TSS_007607	1000	222	0	21 I	within gene(s) PMM1148;

1100717	+	TSS_007608	1000	124	0	0	I	within gene(s) PMM1148;
1100756	+	TSS_007612	1000	180	0	22	I	within gene(s) PMM1148;
1100792	+	TSS_007618	1000	234	0	15	I	within gene(s) PMM1148;
1100867	+	TSS_007633	1000	271	0	5	I	within gene(s) PMM1148;
1100965	-	TSS_020659	1000	151	0	0	Ai	antisense to gene(s) PMM1148;
1101175	+	TSS_007638	1000	5020	0	2	P	15nt upstream of gene PMM1149;
1102029	+	TSS_007680	1000	149	0	2	I	within gene(s) PMM1150;
1103158	-	TSS_020670	1000	2067	0	2	P	16nt upstream of gene PMM1151;
1103474	-	TSS_020672	1000	929	0	2	O	-
1104384	+	TSS_007707	1000	107	0	0	Ai	antisense to gene(s) PMM1152;
1104451	+	TSS_007708	1000	405	0	0	PAi	217nt upstream of gene PMM1153; antisense to gene(s) PMM1152;
1104492	-	TSS_020680	1000	279	0	0	P	18nt upstream of gene PMM1152;
1104544	-	TSS_020682	1000	307	0	6	IP	within gene(s) PMM1152a; 70nt upstream of gene PMM1152;
1104586	-	TSS_020686	1000	3384	0	1	IP	within gene(s) PMM1152a; 112nt upstream of gene PMM1152;
1105595	+	TSS_007711	1000	476	0	5	IP	within gene(s) PMM1153; 99nt upstream of gene PMM1154;
1107430	-	TSS_020693	1000	1140	0	3	P	2nt upstream of gene PMM1156;
1108089	+	TSS_007720	1000	985	0	9	I	within gene(s) PMM1157;
1108099	+	TSS_007722	1000	362	0	0	I	within gene(s) PMM1157;
1108108	+	TSS_007723	1000	2406	0	1	I	within gene(s) PMM1157;
1108118	+	TSS_007726	1000	586	0	1	I	within gene(s) PMM1157;
1108135	+	TSS_007730	1000	247	0	9	I	within gene(s) PMM1157;
1108144	+	TSS_007731	1000	196	0	3	I	within gene(s) PMM1157;
1108162	+	TSS_007733	1000	772	0	1	I	within gene(s) PMM1157;
1108207	+	TSS_007735	1000	181	0	0	I	within gene(s) PMM1157;
1108225	+	TSS_007737	1000	519	0	6	I	within gene(s) PMM1157;
1108300	+	TSS_007751	1000	2212	0	93	I	within gene(s) PMM1157;
1108361	-	TSS_020701	1000	1327	0	4	Ai	antisense to gene(s) PMM1157;
1108375	+	TSS_007770	1000	717	0	6	I	within gene(s) PMM1157;
1108441	+	TSS_007787	1000	4344	0	50	IP	within gene(s) PMM1157; 232nt upstream of gene PMM1158;
1108450	+	TSS_007788	1000	3103	0	10	IP	within gene(s) PMM1157; 223nt upstream of gene PMM1158;
1108498	+	TSS_007798	1000	5902	0	15	IP	within gene(s) PMM1157; 175nt upstream of gene PMM1158;
1108513	+	TSS_007801	1000	1352	0	9	IP	within gene(s) PMM1157; 160nt upstream of gene PMM1158;
1108534	+	TSS_007803	1000	399	0	0	IP	within gene(s) PMM1157; 139nt upstream of gene PMM1158;
1108543	+	TSS_007804	1000	261	0	0	IP	within gene(s) PMM1157; 130nt upstream of gene PMM1158;
1108576	+	TSS_007805	1000	338	0	0	IP	within gene(s) PMM1157; 97nt upstream of gene PMM1158;
1108597	+	TSS_007806	1000	293	0	0	IP	within gene(s) PMM1157; 76nt upstream of gene PMM1158;
1108620	+	TSS_007812	1000	18120	0	21	IP	within gene(s) PMM1157; 53nt upstream of gene PMM1158;
1108639	+	TSS_007816	1000	585	0	6	IP	within gene(s) PMM1157; 34nt upstream of gene PMM1158;
1108654	+	TSS_007820	1000	527	0	0	IP	within gene(s) PMM1157; 19nt upstream of gene PMM1158;
1108670	+	TSS_007821	1000	1039	0	2	IP	within gene(s) PMM1157; 3nt upstream of gene PMM1158;
1108757	+	TSS_007827	1000	881	0	12	I	within gene(s) PMM1158;
1108805	+	TSS_007834	1000	4289	0	23	I	within gene(s) PMM1158;
1108911	-	TSS_020708	1000	156	0	0	Ai	antisense to gene(s) PMM1158;
1108924	-	TSS_020709	1000	240	0	0	Ai	antisense to gene(s) PMM1158;
1108925	+	TSS_007839	1000	167	0	0	I	within gene(s) PMM1158;
1108940	+	TSS_007840	1000	468	0	0	I	within gene(s) PMM1158;
1108955	+	TSS_007841	1000	232	0	0	I	within gene(s) PMM1158;
1108982	+	TSS_007848	1000	1466	0	19	I	within gene(s) PMM1158;
1109017	-	TSS_020710	1000	144	0	0	Ai	antisense to gene(s) PMM1158;
1109018	+	TSS_007853	1000	1382	0	10	I	within gene(s) PMM1158;
1109031	-	TSS_020712	1000	207	0	1	Ai	antisense to gene(s) PMM1158;
1109039	+	TSS_007855	1000	228	0	3	I	within gene(s) PMM1158;
1109043	-	TSS_020714	1000	240	0	6	Ai	antisense to gene(s) PMM1158;
1109073	+	TSS_007861	1000	373	0	27	I	within gene(s) PMM1158;
1109084	-	TSS_020717	1000	1125	0	0	Ai	antisense to gene(s) PMM1158;
1109099	+	TSS_007866	1000	446	0	6	I	within gene(s) PMM1158;
1109108	+	TSS_007867	1000	213	0	0	I	within gene(s) PMM1158;
1109147	+	TSS_007868	1000	225	0	0	I	within gene(s) PMM1158;
1109165	-	TSS_020719	1000	268	0	1	Ai	antisense to gene(s) PMM1158;
1109196	-	TSS_020721	1000	1089	0	3	Ai	antisense to gene(s) PMM1158;
1109204	+	TSS_007876	1000	862	0	36	I	within gene(s) PMM1158;
1109212	-	TSS_020726	1000	467	0	6	Ai	antisense to gene(s) PMM1158;
1109264	+	TSS_007889	1000	713	0	22	I	within gene(s) PMM1158;
1109279	+	TSS_007892	1000	281	0	9	I	within gene(s) PMM1158;
1109298	-	TSS_020727	1000	109	0	0	Ai	antisense to gene(s) PMM1158;
1109375	+	TSS_007897	1000	103	0	0	I	within gene(s) PMM1158;
1109387	+	TSS_007898	1000	180	0	3	I	within gene(s) PMM1158;
1109398	-	TSS_020728	1000	830	0	1	Ai	antisense to gene(s) PMM1158;
1109399	+	TSS_007901	1000	300	0	16	I	within gene(s) PMM1158;
1109436	+	TSS_007911	1000	190	0	6	I	within gene(s) PMM1158;
1109461	-	TSS_020730	1000	722	0	0	Ai	antisense to gene(s) PMM1158;
1109465	+	TSS_007918	1000	254	0	18	I	within gene(s) PMM1158;
1109477	+	TSS_007920	1000	318	0	1	I	within gene(s) PMM1158;
1109540	+	TSS_007925	1000	542	0	35	I	within gene(s) PMM1158;
1109582	-	TSS_020732	1000	105	0	0	Ai	antisense to gene(s) PMM1158;
1109618	+	TSS_007943	1000	126	0	0	I	within gene(s) PMM1158;
1109653	-	TSS_020734	1000	289	0	0	Ai	antisense to gene(s) PMM1158;
1109680	-	TSS_020736	1000	109	0	1	Ai	antisense to gene(s) PMM1158;
1109694	-	TSS_020737	1000	1675	0	1	Ai	antisense to gene(s) PMM1158;

1109705	-	TSS_020739	1000	286	0	1	Ai	antisense to gene(s) PMM1158;
1109792	-	TSS_020741	1000	480	0	1	Ai	antisense to gene(s) PMM1158;
1109834	+	TSS_007962	1000	149	0	0	I	within gene(s) PMM1158;
1109873	+	TSS_007963	1000	187	0	0	I	within gene(s) PMM1158;
1115277	+	TSS_007978	1000	140	0	1	O	-
1115437	+	TSS_007981	1000	405	0	1	O	-
1118179	-	TSS_020766	1000	312	0	1	P	33nt upstream of gene PMM1165;
1120049	+	TSS_007990	1000	1097	0	1	P	15nt upstream of gene PMM1169;
1121150	+	TSS_007994	1000	313	0	0	Ai	antisense to gene(s) PMM1171;
1121159	+	TSS_007995	1000	448	0	0	Ai	antisense to gene(s) PMM1171;
1121311	+	TSS_007997	1000	154	0	0	Ai	antisense to gene(s) PMM1171;
1121377	+	TSS_007999	1000	159	0	1	Ai	antisense to gene(s) PMM1171;
1121400	-	TSS_020791	1000	338	0	0	I	within gene(s) PMM1171;
1121448	-	TSS_020792	1000	135	0	3	I	within gene(s) PMM1171;
1121466	-	TSS_020795	1000	529	0	7	I	within gene(s) PMM1171;
1121481	-	TSS_020798	1000	114	0	0	I	within gene(s) PMM1171;
1121493	-	TSS_020799	1000	151	0	0	I	within gene(s) PMM1171;
1121508	-	TSS_020800	1000	276	0	15	I	within gene(s) PMM1171;
1121580	-	TSS_020805	1000	4491	0	0	P	27nt upstream of gene PMM1171;
1122779	+	TSS_008001	1000	400	0	1	P	17nt upstream of gene PMM1174;
1123430	-	TSS_020808	1000	662	0	6	Ai	antisense to gene(s) PMM1175;
1124119	+	TSS_008006	1000	155	0	0	I	within gene(s) PMM1176;
1127018	+	TSS_008013	1000	499	0	0	Ai	antisense to gene(s) PMM1178;
1127626	+	TSS_008018	1000	3732	0	2	Ai	antisense to gene(s) PMM1179;
1128259	+	TSS_008025	1000	699	0	1	P	28nt upstream of gene PMM1180;
1128268	+	TSS_008027	1000	437	0	3	P	19nt upstream of gene PMM1180;
1128474	-	TSS_020829	1000	1259	0	2	PAi	241nt upstream of gene PMM1179; antisense to gene(s) PMM1180;
1128570	-	TSS_020830	1000	962	0	0	Ai	antisense to gene(s) PMM1180;
1130036	+	TSS_008058	1000	164	0	15	IP	within gene(s) PMM1182; 123nt upstream of gene PMM1183;
1130146	+	TSS_008065	1000	17023	0	4	P	13nt upstream of gene PMM1183;
1130398	-	TSS_020839	1000	362	0	0	Ai	antisense to gene(s) PMM1184;
1130619	+	TSS_008067	1000	116	0	1	IP	within gene(s) PMM1184; 74nt upstream of gene PMM1185;
1130649	+	TSS_008070	1000	1022	0	0	IP	within gene(s) PMM1184; 44nt upstream of gene PMM1185;
1131120	+	TSS_008074	1000	244	0	2	P	59nt upstream of gene PMM1186;
1131150	+	TSS_008076	1000	5266	0	4	P	29nt upstream of gene PMM1186;
1131452	+	TSS_008089	1000	142	0	12	I	within gene(s) PMM1186;
1131719	+	TSS_008097	1000	637	0	12	I	within gene(s) PMM1186;
1131752	+	TSS_008101	1000	113	0	1	I	within gene(s) PMM1186;
1131989	+	TSS_008109	1000	179	0	1	I	within gene(s) PMM1186;
1133995	+	TSS_008119	1000	678	0	5	I	within gene(s) PMM1188;
1136341	+	TSS_008129	1000	154	0	0	P	18nt upstream of gene PMM1190;
1136852	+	TSS_008136	1000	1079	0	1	P	0nt upstream of gene PMM1191;
1136948	+	TSS_008141	1000	132	0	0	I	within gene(s) PMM1191;
1136957	+	TSS_008142	1000	122	0	0	I	within gene(s) PMM1191;
1137017	+	TSS_008147	1000	169	0	18	I	within gene(s) PMM1191;
1137053	+	TSS_008151	1000	240	0	7	I	within gene(s) PMM1191;
1137057	-	TSS_020853	1000	101	0	0	Ai	antisense to gene(s) PMM1191;
1137073	-	TSS_020856	1000	286	0	11	Ai	antisense to gene(s) PMM1191;
1137086	+	TSS_008154	1000	165	0	3	I	within gene(s) PMM1191;
1137131	+	TSS_008158	1000	124	0	4	I	within gene(s) PMM1191;
1137144	+	TSS_008162	1000	610	0	15	I	within gene(s) PMM1191;
1137185	+	TSS_008169	1000	156	0	0	I	within gene(s) PMM1191;
1137200	+	TSS_008171	1000	245	0	8	I	within gene(s) PMM1191;
1137233	+	TSS_008175	1000	208	0	0	I	within gene(s) PMM1191;
1137285	-	TSS_020860	1000	208	0	0	Ai	antisense to gene(s) PMM1191;
1137321	-	TSS_020861	1000	270	0	0	Ai	antisense to gene(s) PMM1191;
1137362	+	TSS_008187	1000	102	0	12	I	within gene(s) PMM1191;
1137382	-	TSS_020863	1000	1874	0	1	Ai	antisense to gene(s) PMM1191;
1137394	-	TSS_020864	1000	387	0	0	Ai	antisense to gene(s) PMM1191;
1137842	+	TSS_008207	1000	203	0	6	I	within gene(s) PMM1191;
1137863	+	TSS_008212	1000	106	0	6	I	within gene(s) PMM1191;
1137881	+	TSS_008214	1000	149	0	4	I	within gene(s) PMM1191;
1138019	+	TSS_008226	1000	129	0	23	I	within gene(s) PMM1191;
1138050	-	TSS_020872	1000	185	0	1	Ai	antisense to gene(s) PMM1191;
1138133	+	TSS_008241	1000	164	0	21	I	within gene(s) PMM1191;
1138172	+	TSS_008252	1000	597	0	36	I	within gene(s) PMM1191;
1138187	+	TSS_008256	1000	112	0	0	I	within gene(s) PMM1191;
1138235	+	TSS_008260	1000	214	0	15	I	within gene(s) PMM1191;
1138412	+	TSS_008269	1000	917	0	15	I	within gene(s) PMM1191;
1138460	+	TSS_008277	1000	120	0	3	I	within gene(s) PMM1191;
1138493	+	TSS_008280	1000	167	0	3	I	within gene(s) PMM1191;
1138553	+	TSS_008288	1000	158	0	1	I	within gene(s) PMM1191;
1138651	-	TSS_020883	1000	174	0	0	Ai	antisense to gene(s) PMM1191;
1138670	+	TSS_008297	1000	139	0	12	I	within gene(s) PMM1191;
1138673	-	TSS_020884	1000	113	0	0	Ai	antisense to gene(s) PMM1191;
1138742	+	TSS_008310	1000	159	0	51	I	within gene(s) PMM1191;
1138799	+	TSS_008320	1000	145	0	18	I	within gene(s) PMM1191;
1138805	-	TSS_020886	1000	142	0	1	Ai	antisense to gene(s) PMM1191;
1138862	-	TSS_020888	1000	1187	0	0	Ai	antisense to gene(s) PMM1191;

1138882	-	TSS_020891	1000	347	0	5 Ai	antisense to gene(s) PMM1191;
1138904	-	TSS_020893	1000	317	0	0 Ai	antisense to gene(s) PMM1191;
1138932	-	TSS_020896	1000	229	0	0 Ai	antisense to gene(s) PMM1191;
1139830	+	TSS_008325	1000	152	0	0 PAi	95nt upstream of gene PMM1193; antisense to gene(s) PMM1192;
1152750	-	TSS_020919	1000	145	0	0 I	within gene(s) PMM1204;
1152998	-	TSS_020920	1000	257	0	0 IP	within gene(s) PMM1205; 88nt upstream of gene PMM1204;
1157220	+	TSS_008358	1000	491	0	0 Ai	antisense to gene(s) PMM1208;
1181446	-	TSS_020966	1000	245	0	2 Ai	antisense to gene(s) PMM1229;
1183984	+	TSS_008415	1000	581	0	6 I	within gene(s) PMM1232;
1186173	+	TSS_008418	1000	399	0	2 I	within gene(s) PMM1234;
1186359	-	TSS_020975	1000	482	0	2 Ai	antisense to gene(s) PMM1234;
1186823	+	TSS_008424	1000	2357	0	2 I	within gene(s) PMM1234;
1187821	+	TSS_008434	1000	447	0	2 IP	within gene(s) PMM1235; 110nt upstream of gene PMM1236;
1192382	+	TSS_008443	1000	156	0	8 IP	within gene(s) PMM1239; 39nt upstream of gene PMM1240;
1192406	+	TSS_008445	1000	545	0	2 IP	within gene(s) PMM1239; 15nt upstream of gene PMM1240;
1201748	-	TSS_021001	1000	162	0	0 I	within gene(s) PMM1249;
1202453	-	TSS_021005	1000	124	0	1 I	within gene(s) PMM1250;
1203802	+	TSS_008457	1000	339	0	1 Ai	antisense to gene(s) PMM1251;
1204621	-	TSS_021015	1000	235	0	0 P	82nt upstream of gene PMM1251;
1206887	+	TSS_008463	1000	102	0	0 Ai	antisense to gene(s) PMM1254;
1209541	-	TSS_021027	1000	973	0	1 I	within gene(s) PMM1256;
1211786	+	TSS_008473	1000	215	0	0 I	within gene(s) PMM1258;
1212906	+	TSS_008479	1000	314	0	0 I	within gene(s) PMM1259;
1213317	+	TSS_008487	1000	511	0	1 I	within gene(s) PMM1259;
1214724	+	TSS_008492	1000	712	0	0 IP	within gene(s) PMM1260; 140nt upstream of gene PMM1261;
1214740	+	TSS_008493	1000	107	0	0 IP	within gene(s) PMM1260; 124nt upstream of gene PMM1261;
1216537	+	TSS_008504	1000	102	0	0 Ai	antisense to gene(s) PMM1262;
1216690	+	TSS_008506	1000	102	0	0 Ai	antisense to gene(s) PMM1262;
1218110	+	TSS_008514	1000	185	0	1 Ai	antisense to gene(s) PMM1264;
1218175	-	TSS_021056	1000	342	0	6 I	within gene(s) PMM1264;
1218209	+	TSS_008516	1000	148	0	0 Ai	antisense to gene(s) PMM1264;
1218313	-	TSS_021070	1000	194	0	15 I	within gene(s) PMM1264;
1218440	+	TSS_008522	1000	103	0	0 Ai	antisense to gene(s) PMM1264;
1218445	-	TSS_021082	1000	157	0	19 I	within gene(s) PMM1264;
1218465	-	TSS_021087	1000	280	0	1 I	within gene(s) PMM1264;
1218507	+	TSS_008524	1000	142	0	0 Ai	antisense to gene(s) PMM1264;
1218563	+	TSS_008525	1000	328	0	4 Ai	antisense to gene(s) PMM1264;
1218613	-	TSS_021099	1000	171	0	3 I	within gene(s) PMM1264;
1218623	+	TSS_008528	1000	1842	0	1 Ai	antisense to gene(s) PMM1264;
1218649	-	TSS_021104	1000	904	0	27 I	within gene(s) PMM1264;
1218735	+	TSS_008529	1000	115	0	1 Ai	antisense to gene(s) PMM1264;
1218757	-	TSS_021115	1000	481	0	2 I	within gene(s) PMM1264;
1218769	-	TSS_021117	1000	152	0	2 I	within gene(s) PMM1264;
1218787	-	TSS_021119	1000	103	0	3 I	within gene(s) PMM1264;
1218832	-	TSS_021123	1000	161	0	0 I	within gene(s) PMM1264;
1218868	-	TSS_021124	1000	103	0	6 I	within gene(s) PMM1264;
1218904	-	TSS_021133	1000	204	0	6 I	within gene(s) PMM1264;
1218920	+	TSS_008532	1000	158	0	0 Ai	antisense to gene(s) PMM1264;
1218931	-	TSS_021138	1000	101	0	10 I	within gene(s) PMM1264;
1218939	+	TSS_008533	1000	513	0	0 Ai	antisense to gene(s) PMM1264;
1218943	-	TSS_021141	1000	382	0	3 I	within gene(s) PMM1264;
1218964	-	TSS_021145	1000	278	0	12 I	within gene(s) PMM1264;
1218994	-	TSS_021148	1000	124	0	12 I	within gene(s) PMM1264;
1219090	-	TSS_021153	1000	336	0	6 I	within gene(s) PMM1264;
1219102	-	TSS_021154	1000	109	0	0 I	within gene(s) PMM1264;
1219126	-	TSS_021157	1000	186	0	10 I	within gene(s) PMM1264;
1219135	-	TSS_021160	1000	370	0	13 I	within gene(s) PMM1264;
1219165	-	TSS_021167	1000	513	0	14 I	within gene(s) PMM1264;
1219183	-	TSS_021173	1000	166	0	1 I	within gene(s) PMM1264;
1219195	-	TSS_021175	1000	228	0	18 I	within gene(s) PMM1264;
1219255	+	TSS_008537	1000	301	0	1 Ai	antisense to gene(s) PMM1264;
1219300	-	TSS_021182	1000	206	0	9 I	within gene(s) PMM1264;
1219348	-	TSS_021187	1000	117	0	6 I	within gene(s) PMM1264;
1219417	-	TSS_021193	1000	403	0	12 I	within gene(s) PMM1264;
1219521	-	TSS_021210	1000	116	0	6 I	within gene(s) PMM1264;
1219704	-	TSS_021220	1000	109	0	7 I	within gene(s) PMM1264;
1219762	-	TSS_021223	1000	188	0	0 I	within gene(s) PMM1264;
1219801	-	TSS_021224	1000	304	0	1 P	24nt upstream of gene PMM1264;
1219908	-	TSS_021226	1000	140	0	0 IP	within gene(s) PMM1265; 131nt upstream of gene PMM1264;
1220160	+	TSS_008542	1000	173	0	5 Ai	antisense to gene(s) PMM1265;
1221551	-	TSS_021233	1000	103	0	2 IP	within gene(s) PMM1267; 57nt upstream of gene PMM1266;
1223066	+	TSS_008550	1000	170	0	2 IAd	within gene(s) PMM1268; antisense to gene(s) PMM1269 (28nt downstream);
1223096	+	TSS_008553	1000	160	0	0 Ai	antisense to gene(s) PMM1269;
1223572	-	TSS_021241	1000	127	0	3 I	within gene(s) PMM1269;
1224017	-	TSS_021249	1000	4451	0	8 P	16nt upstream of gene PMM1269;
1224107	+	TSS_008556	1000	987	0	2 Ai	antisense to gene(s) PMM1270;
1224306	-	TSS_021254	1000	551	0	3 I	within gene(s) PMM1270;
1224483	+	TSS_008561	1000	1634	0	1 Ai	antisense to gene(s) PMM1270;
1225097	+	TSS_008563	1000	354	0	0 P	51nt upstream of gene PMM1271;

1225100	-	TSS_021280	1000	606	0	2 P	20nt upstream of gene PMM1270;
1226226	+	TSS_008565	1000	111	0	0 Ai	antisense to gene(s) PMM1272;
1226399	+	TSS_008567	1000	2457	0	2 PAi	139nt upstream of gene PMM1273; antisense to gene(s) PMM1272;
1226493	-	TSS_021285	1000	5091	0	2 P	23nt upstream of gene PMM1272;
1228824	+	TSS_008574	1000	528	0	4 P	13nt upstream of gene PMM1276;
1229316	+	TSS_008579	1000	742	0	1 Ai	antisense to gene(s) PMM1278;
1230756	-	TSS_021305	1000	171	0	0 I	within gene(s) PMM1280;
1230808	-	TSS_021306	1000	415	0	2 I	within gene(s) PMM1280;
1231722	-	TSS_021313	1000	711	0	2 IP	within gene(s) PMM1281; 194nt upstream of gene PMM1280;
1233089	+	TSS_008592	1000	2654	0	1 Ai	antisense to gene(s) PMM1283;
1233102	+	TSS_008593	1000	249	0	1 Ai	antisense to gene(s) PMM1283;
1233159	+	TSS_008595	1000	207	0	0 Ai	antisense to gene(s) PMM1283;
1233255	+	TSS_008597	1000	227	0	5 Ai	antisense to gene(s) PMM1283;
1233266	-	TSS_021316	1000	446	0	3 I	within gene(s) PMM1283;
1233281	-	TSS_021319	1000	563	0	3 I	within gene(s) PMM1283;
1233285	+	TSS_008600	1000	206	0	0 Ai	antisense to gene(s) PMM1283;
1233298	-	TSS_021322	1000	575	0	9 I	within gene(s) PMM1283;
1233300	+	TSS_008601	1000	478	0	0 Ai	antisense to gene(s) PMM1283;
1233311	+	TSS_008603	1000	702	0	1 Ai	antisense to gene(s) PMM1283;
1233320	-	TSS_021325	1000	285	0	0 I	within gene(s) PMM1283;
1233322	+	TSS_008605	1000	342	0	1 Ai	antisense to gene(s) PMM1283;
1233365	-	TSS_021336	1000	804	0	75 I	within gene(s) PMM1283;
1233366	+	TSS_008606	1000	104	0	0 Ai	antisense to gene(s) PMM1283;
1233384	+	TSS_008608	1000	200	0	1 Ai	antisense to gene(s) PMM1283;
1233399	+	TSS_008610	1000	529	0	1 Ai	antisense to gene(s) PMM1283;
1233419	-	TSS_021352	1000	312	0	0 I	within gene(s) PMM1283;
1233429	+	TSS_008613	1000	191	0	1 Ai	antisense to gene(s) PMM1283;
1233437	-	TSS_021354	1000	292	0	6 I	within gene(s) PMM1283;
1233452	-	TSS_021358	1000	693	0	12 I	within gene(s) PMM1283;
1233465	+	TSS_008614	1000	253	0	0 Ai	antisense to gene(s) PMM1283;
1233473	+	TSS_008615	1000	431	0	4 Ai	antisense to gene(s) PMM1283;
1233494	-	TSS_021368	1000	830	0	42 I	within gene(s) PMM1283;
1233547	-	TSS_021380	1000	967	0	60 I	within gene(s) PMM1283;
1233611	-	TSS_021394	1000	294	0	0 I	within gene(s) PMM1283;
1233623	-	TSS_021395	1000	175	0	0 I	within gene(s) PMM1283;
1233635	-	TSS_021396	1000	312	0	6 I	within gene(s) PMM1283;
1233652	-	TSS_021400	1000	448	0	3 I	within gene(s) PMM1283;
1233677	-	TSS_021405	1000	460	0	6 I	within gene(s) PMM1283;
1233722	-	TSS_021415	1000	518	0	48 I	within gene(s) PMM1283;
1233764	-	TSS_021426	1000	310	0	9 I	within gene(s) PMM1283;
1233822	-	TSS_021428	1000	652	0	2 P	22nt upstream of gene PMM1283;
1235226	-	TSS_021447	1000	9509	0	4 P	16nt upstream of gene PMM1285;
1235758	+	TSS_008625	1000	241	0	0 Ai	antisense to gene(s) PMM1286;
1236777	-	TSS_021486	1000	5818	0	7 P	16nt upstream of gene PMM1286;
1238666	-	TSS_021500	1000	217	0	0 I	within gene(s) PMM1287;
1238943	-	TSS_021501	1000	1469	0	2 P	24nt upstream of gene PMM1287;
1239039	+	TSS_008635	1000	381	0	0 P	30nt upstream of gene PMM1288;
1239358	-	TSS_021504	1000	253	0	1 Ai	antisense to gene(s) PMM1288;
1239789	-	TSS_021507	1000	104	0	0 Ai	antisense to gene(s) PMM1288;
1239822	+	TSS_008671	1000	241	0	19 I	within gene(s) PMM1288;
1239889	-	TSS_021509	1000	121	0	1 Ai	antisense to gene(s) PMM1288;
1239949	-	TSS_021512	1000	442	0	0 Ai	antisense to gene(s) PMM1288;
1240190	+	TSS_008676	1000	902	0	1 Ai	antisense to gene(s) PMM1289;
1240340	+	TSS_008683	1000	288	0	0 Ai	antisense to gene(s) PMM1289;
1240490	-	TSS_021526	1000	578	0	2 I	within gene(s) PMM1289;
1240521	-	TSS_021528	1000	212	0	9 I	within gene(s) PMM1289;
1240674	-	TSS_021537	1000	142	0	10 I	within gene(s) PMM1289;
1240780	+	TSS_008687	1000	195	0	0 Ai	antisense to gene(s) PMM1289;
1240970	-	TSS_021544	1000	250	0	0 I	within gene(s) PMM1289;
1241067	-	TSS_021550	1000	12375	0	2 P	18nt upstream of gene PMM1289;
1241345	+	TSS_008696	1000	112	0	0 Ai	antisense to gene(s) PMM1290;
1242168	+	TSS_008699	1000	240	0	0 Ai	antisense to gene(s) PMM1290;
1242445	+	TSS_008700	1000	712	0	0 Ai	antisense to gene(s) PMM1290;
1242497	+	TSS_008701	1000	200	0	0 Ai	antisense to gene(s) PMM1290;
1243222	+	TSS_008708	1000	121	0	0 I	within gene(s) PMM1291;
1243570	-	TSS_021566	1000	103	0	0 Ai	antisense to gene(s) PMM1291;
1244009	+	TSS_008715	1000	247	0	0 Ai	antisense to gene(s) PMM1292;
1245509	+	TSS_008720	1000	1670	0	0 P	17nt upstream of gene PMM1293;
1245566	+	TSS_008722	1000	417	0	5 I	within gene(s) PMM1293;
1245584	+	TSS_008724	1000	280	0	0 I	within gene(s) PMM1293;
1245700	+	TSS_008729	1000	691	0	0 I	within gene(s) PMM1293;
1245796	+	TSS_008740	1000	1773	0	6 I	within gene(s) PMM1293;
1245812	+	TSS_008743	1000	510	0	4 I	within gene(s) PMM1293;
1245832	+	TSS_008747	1000	136	0	3 I	within gene(s) PMM1293;
1245841	+	TSS_008748	1000	217	0	3 I	within gene(s) PMM1293;
1245861	+	TSS_008750	1000	108	0	0 I	within gene(s) PMM1293;
1247681	-	TSS_021585	1000	153	0	0 P	20nt upstream of gene PMM1296;
1250117	+	TSS_008786	1000	101	0	0 Ai	antisense to gene(s) PMM1299;
1250130	-	TSS_021621	1000	1890	0	6 IP	within gene(s) PMM1299; 75nt upstream of gene PMM1298;

1250468	-	TSS_021626	1000	440	0	3	I	within gene(s) PMM1299;
1251088	+	TSS_008789	1000	167	0	0	P	15nt upstream of gene PMM1300;
1252148	+	TSS_008795	1000	112	0	2	I	within gene(s) PMM1300;
1252727	-	TSS_021638	1000	1662	0	1	O	-
1252970	+	TSS_008806	1000	922	0	6	P	32nt upstream of gene PMM1301;
1253102	+	TSS_008807	1000	212	0	5	I	within gene(s) PMM1301;
1258142	-	TSS_021650	1000	214	0	12	P	16nt upstream of gene PMM1304;
1258258	+	TSS_008821	1000	689	0	6	P	151nt upstream of gene PMM1305;
1259122	-	TSS_021659	1000	161	0	1	Ai	antisense to gene(s) PMM1305;
1261120	+	TSS_008832	1000	125	0	0	IP	within gene(s) PMM1307; 179nt upstream of gene PMM1308;
1262176	+	TSS_008836	1000	1346	0	2	P	26nt upstream of gene PMM1309;
1262799	+	TSS_008860	1000	110	0	4	I	within gene(s) PMM1309;
1262859	+	TSS_008868	1000	121	0	9	I	within gene(s) PMM1309;
1262904	+	TSS_008873	1000	115	0	1	I	within gene(s) PMM1309;
1263425	-	TSS_021678	1000	522	0	1	O	-
1263674	+	TSS_008890	1000	857	0	1	I	within gene(s) PMM1310;
1264866	+	TSS_008896	1000	140	0	4	Ai	antisense to gene(s) PMM1311;
1265561	+	TSS_008901	1000	14113	0	4	I	within gene(s) PMM1312;
1266213	-	TSS_021686	1000	228	0	1	Ai	antisense to gene(s) PMM1312;
1266337	+	TSS_008927	1000	105	0	1	I	within gene(s) PMM1312;
1266690	+	TSS_008933	1000	16907	0	2	P	20nt upstream of gene PMM1313;
1266702	+	TSS_008935	1000	355	0	0	P	8nt upstream of gene PMM1313;
1266713	+	TSS_008936	1000	106	0	0	I	within gene(s) PMM1313;
1266750	+	TSS_008942	1000	138	0	16	I	within gene(s) PMM1313;
1266761	+	TSS_008943	1000	133	0	18	I	within gene(s) PMM1313;
1267025	+	TSS_008965	1000	108	0	3	I	within gene(s) PMM1313;
1267067	+	TSS_008971	1000	508	0	66	I	within gene(s) PMM1313;
1267140	-	TSS_021696	1000	140	0	1	Ai	antisense to gene(s) PMM1313;
1267176	-	TSS_021698	1000	156	0	0	Ai	antisense to gene(s) PMM1313;
1267212	-	TSS_021699	1000	200	0	6	Ai	antisense to gene(s) PMM1313;
1267259	+	TSS_008999	1000	166	0	12	IP	within gene(s) PMM1313; 147nt upstream of gene PMM1314;
1267775	+	TSS_009032	1000	112	0	15	I	within gene(s) PMM1314;
1268104	+	TSS_009038	1000	2547	0	2	P	24nt upstream of gene PMM1315;
1270161	+	TSS_009088	1000	225	0	7	P	15nt upstream of gene PMM1317;
1270931	-	TSS_021721	1000	177	0	8	I	within gene(s) PMM1318;
1272067	-	TSS_021730	1000	76260	0	8	P	65nt upstream of gene PMM1321;
1273518	-	TSS_021736	1000	1364	0	2	Ai	antisense to gene(s) PMM1322;
1273866	-	TSS_021738	1000	109	0	0	Ai	antisense to gene(s) PMM1322;
1274764	+	TSS_009112	1000	717	0	1	P	86nt upstream of gene PMM1323;
1275301	-	TSS_021744	1000	132	0	0	Ai	antisense to gene(s) PMM1323;
1276011	-	TSS_021746	1000	709	0	2	Ai	antisense to gene(s) PMM1323;
1276210	+	TSS_009126	1000	227	0	0	I	within gene(s) PMM1324;
1276620	-	TSS_021750	1000	306	0	0	Ai	antisense to gene(s) PMM1324;
1276627	-	TSS_021751	1000	154	0	0	Ai	antisense to gene(s) PMM1324;
1276641	+	TSS_009130	1000	376	0	0	I	within gene(s) PMM1324;
1278318	+	TSS_009140	1000	399	0	4	IP	within gene(s) PMM1326; 106nt upstream of gene PMM1327;
1281106	-	TSS_021761	1000	273	0	0	IP	within gene(s) PMM1330; 132nt upstream of gene PMM1329;
1281349	-	TSS_021764	1000	103	0	0	P	30nt upstream of gene PMM1330;
1285667	-	TSS_021775	1000	120	0	3	I	within gene(s) PMM1335;
1285747	-	TSS_021778	1000	111	0	0	I	within gene(s) PMM1335;
1286203	+	TSS_009172	1000	117	0	2	Ai	antisense to gene(s) PMM1335;
1286762	+	TSS_009175	1000	258	0	5	Ai	antisense to gene(s) PMM1337;
1287550	-	TSS_021787	1000	136	0	0	IP	within gene(s) PMM1338; 17nt upstream of gene PMM1337;
1287887	-	TSS_021797	1000	377	0	1	I	within gene(s) PMM1338;
1287977	-	TSS_021798	1000	261	0	0	I	within gene(s) PMM1338;
1288162	+	TSS_009182	1000	208	0	6	Ai	antisense to gene(s) PMM1338;
1288975	-	TSS_021813	1000	211	0	2	I	within gene(s) PMM1338;
1291245	-	TSS_021830	1000	144	0	0	Ai	antisense to gene(s) PMM1340;
1291318	-	TSS_021831	1000	182	0	0	Ai	antisense to gene(s) PMM1340;
1291478	+	TSS_009198	1000	1785	0	2	I	within gene(s) PMM1340;
1291709	-	TSS_021832	1000	123	0	0	Ai	antisense to gene(s) PMM1340;
1293363	+	TSS_009216	1000	119	0	0	I	within gene(s) PMM1341;
1293438	+	TSS_009220	1000	385	0	5	I	within gene(s) PMM1341;
1293828	+	TSS_009225	1000	154	0	2	I	within gene(s) PMM1341;
1294828	-	TSS_021853	1000	157	0	3	I	within gene(s) PMM1342;
1295467	-	TSS_021870	1000	167	0	6	I	within gene(s) PMM1342;
1295522	-	TSS_021872	1000	102	0	0	P	13nt upstream of gene PMM1342;
1295547	-	TSS_021873	1000	121	0	1	P	38nt upstream of gene PMM1342;
1295976	+	TSS_009237	1000	1070	0	10	P	65nt upstream of gene PMM1344;
1296395	+	TSS_009250	1000	8460	0	7	IP	within gene(s) PMM1344; 96nt upstream of gene PMM1345;
1296404	+	TSS_009253	1000	161	0	4	IP	within gene(s) PMM1344; 87nt upstream of gene PMM1345;
1296628	-	TSS_021884	1000	238	0	0	Ai	antisense to gene(s) PMM1345;
1297508	-	TSS_021886	1000	346	0	6	P	89nt upstream of gene PMM1346;
1299151	+	TSS_009265	1000	362	0	0	Ai	antisense to gene(s) PMM1348;
1299740	+	TSS_009266	1000	184	0	0	P	25nt upstream of gene PMM1349;
1299756	+	TSS_009267	1000	103	0	2	P	9nt upstream of gene PMM1349;
1300005	+	TSS_009269	1000	113	0	5	I	within gene(s) PMM1349;
1300739	+	TSS_009275	1000	203	0	0	P	24nt upstream of gene PMM1350;
1300764	+	TSS_009277	1000	7149	0	2	I	within gene(s) PMM1350;

1300820	+	TSS_009282	1000	185	0	0	I	within gene(s) PMM1350;
1300862	+	TSS_009289	1000	234	0	18	I	within gene(s) PMM1350;
1300907	+	TSS_009295	1000	182	0	21	I	within gene(s) PMM1350;
1300946	+	TSS_009302	1000	135	0	0	I	within gene(s) PMM1350;
1300973	+	TSS_009303	1000	148	0	0	I	within gene(s) PMM1350;
1301001	+	TSS_009313	1000	245	0	39	I	within gene(s) PMM1350;
1301039	+	TSS_009321	1000	103	0	0	I	within gene(s) PMM1350;
1301738	-	TSS_021902	1000	251	0	3	P	22nt upstream of gene PMM1351;
1301991	-	TSS_021906	1000	366	0	22	I	within gene(s) PMM1352;
1302034	-	TSS_021914	1000	369	0	12	I	within gene(s) PMM1352;
1302064	-	TSS_021921	1000	387	0	3	I	within gene(s) PMM1352;
1302079	-	TSS_021924	1000	123	0	6	I	within gene(s) PMM1352;
1302130	-	TSS_021928	1000	12800	0	2	P	33nt upstream of gene PMM1352;
1304163	+	TSS_009334	1000	635	0	0	Ai	antisense to gene(s) PMM1354;
1304348	+	TSS_009336	1000	168	0	1	Ai	antisense to gene(s) PMM1354;
1304765	-	TSS_021972	1000	996	0	0	P	19nt upstream of gene PMM1354;
1304776	-	TSS_021973	1000	293	0	6	P	30nt upstream of gene PMM1354;
1307622	+	TSS_009348	1000	460	0	0	O	-
1309146	-	TSS_021985	1000	133	0	0	I	within gene(s) PMM1360;
1313108	+	TSS_009360	1000	904	0	2	P	16nt upstream of gene PMM1365;
1313370	+	TSS_009368	1000	102	0	12	I	within gene(s) PMM1365;
1315509	-	TSS_021995	1000	1269	0	15	P	15nt upstream of gene PMM1368;
1316344	-	TSS_022008	1000	481	0	5	P	10nt upstream of gene PMM1369;
1318282	-	TSS_022017	1000	184	0	0	O	-
1319591	+	TSS_009381	1000	2747	0	2	O	-
1319966	+	TSS_009388	1000	438	0	2	P	13nt upstream of gene PMM1372;
1319980	+	TSS_009389	1000	1817	0	5	I	within gene(s) PMM1372;
1320260	-	TSS_022021	1000	186	0	1	Ad	antisense to gene(s) PMM1372 (4nt downstream);
1321010	+	TSS_009393	1000	4163	0	2	O	-
1322462	-	TSS_022026	1000	443	0	1	O	-
1322954	-	TSS_022028	1000	101	0	0	P	15nt upstream of gene PMM1374;
1324248	+	TSS_009403	1000	324	0	0	P	19nt upstream of gene PMM1377;
1326668	-	TSS_022036	1000	6129	0	2	O	-
1327175	+	TSS_009414	1000	472	0	1	O	-
1328956	-	TSS_022041	1000	3951	0	2	O	-
1329106	+	TSS_009420	1000	215	0	1	O	-
1332432	+	TSS_009433	1000	1863	0	2	O	-
1334143	+	TSS_009437	1000	468	0	0	O	-
1335359	+	TSS_009441	1000	20628	0	2	O	-
1336436	+	TSS_009446	1000	774	0	1	O	-
1338319	-	TSS_022065	1000	180	0	5	Ai	antisense to gene(s) PMM1386;
1338981	+	TSS_009453	1000	177	0	0	P	17nt upstream of gene PMM1387;
1339834	-	TSS_022068	1000	289	0	0	P	32nt upstream of gene PMM1388;
1341205	-	TSS_022072	1000	240	0	1	P	20nt upstream of gene PMM1390;
1341449	+	TSS_009458	1000	196	0	0	P	15nt upstream of gene PMM1391;
1343374	+	TSS_009461	1000	112	0	0	I	within gene(s) PMM1394;
1343856	-	TSS_022077	1000	2847	0	7	O	-
1344022	-	TSS_022079	1000	153	0	2	O	-
1345064	-	TSS_022086	1000	185	0	1	I	within gene(s) PMM1396;
1345196	-	TSS_022094	1000	168	0	23	IP	within gene(s) PMM1397; 129nt upstream of gene PMM1396;
1345665	-	TSS_022114	1000	139	0	0	IP	within gene(s) PMM1399; 98nt upstream of gene PMM1398;
1346141	+	TSS_009470	1000	132	0	1	Ai	antisense to gene(s) PMM1400;
1346356	-	TSS_022133	1000	150	0	3	I	within gene(s) PMM1400;
1346398	-	TSS_022139	1000	305	0	15	I	within gene(s) PMM1400;
1346413	-	TSS_022143	1000	168	0	6	I	within gene(s) PMM1400;
1346865	-	TSS_022147	1000	38541	0	2	P	239nt upstream of gene PMM1400;
1346985	+	TSS_009478	1000	1282	0	0	P	146nt upstream of gene PMM1401;
1347056	+	TSS_009479	1000	158	0	0	P	75nt upstream of gene PMM1401;
1347808	+	TSS_009482	1000	185	0	2	P	21nt upstream of gene PMM1402;
1350449	-	TSS_022157	1000	384	0	2	P	81nt upstream of gene PMM1404;
1351013	-	TSS_022159	1000	548	0	4	P	26nt upstream of gene PMM1405;
1352055	-	TSS_022163	1000	361	0	0	P	24nt upstream of gene PMM1408;
1352429	-	TSS_022164	1000	1277	0	2	P	18nt upstream of gene PMM1409;
1353625	-	TSS_022173	1000	256	0	7	O	-
1353716	-	TSS_022177	1000	1380	0	1	O	-
1354656	+	TSS_009501	1000	960	0	1	P	26nt upstream of gene PMM1412;
1354742	+	TSS_009503	1000	141	0	9	I	within gene(s) PMM1412;
1354868	+	TSS_009516	1000	129	0	17	I	within gene(s) PMM1412;
1355165	+	TSS_009521	1000	970	0	3	P	17nt upstream of gene PMM1413;
1355765	+	TSS_009529	1000	116	0	1	IP	within gene(s) PMM1414; 230nt upstream of gene PMM1415;
1356437	-	TSS_022186	1000	273	0	0	Ai	antisense to gene(s) PMM1415;
1358219	+	TSS_009547	1000	112	0	2	I	within gene(s) PMM1416;
1358428	-	TSS_022195	1000	114	0	1	Ai	antisense to gene(s) PMM1416;
1358652	+	TSS_009554	1000	675	0	1	I	within gene(s) PMM1416;
1358898	-	TSS_022197	1000	403	0	0	Ai	antisense to gene(s) PMM1416;
1359128	-	TSS_022199	1000	130	0	0	Ai	antisense to gene(s) PMM1416;
1359810	-	TSS_022204	1000	407	0	1	Ai	antisense to gene(s) PMM1416;
1359899	-	TSS_022211	1000	361	0	7	O	-
1360152	+	TSS_009564	1000	113	0	1	O	-

1360185	-	TSS_022214	1000	10601	0	2	O	-
1362875	+	TSS_009575	1000	187	0	0	Ai	antisense to gene(s) PMM1421 PMM1422;
1363264	-	TSS_022221	1000	252	0	0	I	within gene(s) PMM1422;
1363417	-	TSS_022222	1000	141	0	0	P	27nt upstream of gene PMM1422;
1364219	-	TSS_022223	1000	111	0	12	P	69nt upstream of gene PMM1424;
1364352	-	TSS_022229	1000	1567	0	1	P	202nt upstream of gene PMM1424;
1364483	+	TSS_009579	1000	152	0	0	O	-
1366150	-	TSS_022234	1000	144	0	2	Ai	antisense to gene(s) PMM1425;
1366222	-	TSS_022236	1000	242	0	0	Ai	antisense to gene(s) PMM1425;
1367120	+	TSS_009587	1000	132	0	0	P	6nt upstream of gene PMM1427;
1367587	+	TSS_009590	1000	238	0	1	P	92nt upstream of gene PMM1428;
1369959	+	TSS_009596	1000	183	0	4	I	within gene(s) PMM1432;
1370154	-	TSS_022246	1000	107	0	0	Ai	antisense to gene(s) PMM1432;
1371646	+	TSS_009605	1000	896	0	4	P	31nt upstream of gene PMM1434;
1371875	+	TSS_009609	1000	175	0	10	I	within gene(s) PMM1434;
1372149	-	TSS_022253	1000	1739	0	2	Ai	antisense to gene(s) PMM1434;
1372986	+	TSS_009622	1000	141	0	0	I	within gene(s) PMM1434;
1373308	+	TSS_009626	1000	1295	0	4	P	17nt upstream of gene PMM1435;
1373532	+	TSS_009629	1000	513	0	1	I	within gene(s) PMM1435;
1373670	+	TSS_009632	1000	203	0	2	Ai	antisense to gene(s) PMM1436;
1373679	+	TSS_009633	1000	1833	0	0	Ai	antisense to gene(s) PMM1436;
1373844	+	TSS_009635	1000	717	0	1	Ai	antisense to gene(s) PMM1436;
1373856	+	TSS_009636	1000	383	0	0	Ai	antisense to gene(s) PMM1436;
1373858	-	TSS_022261	1000	136	0	0	I	within gene(s) PMM1436;
1373890	-	TSS_022265	1000	184	0	12	I	within gene(s) PMM1436;
1373898	+	TSS_009637	1000	277	0	0	Ai	antisense to gene(s) PMM1436;
1373900	-	TSS_022267	1000	181	0	2	I	within gene(s) PMM1436;
1373912	-	TSS_022270	1000	388	0	16	I	within gene(s) PMM1436;
1373925	+	TSS_009638	1000	128	0	0	Ai	antisense to gene(s) PMM1436;
1373945	-	TSS_022278	1000	111	0	3	I	within gene(s) PMM1436;
1373993	-	TSS_022282	1000	191	0	3	I	within gene(s) PMM1436;
1374014	-	TSS_022285	1000	241	0	14	I	within gene(s) PMM1436;
1374056	-	TSS_022295	1000	335	0	27	I	within gene(s) PMM1436;
1374134	-	TSS_022303	1000	272	0	0	I	within gene(s) PMM1436;
1374185	-	TSS_022315	1000	413	0	36	I	within gene(s) PMM1436;
1374224	-	TSS_022320	1000	640	0	9	I	within gene(s) PMM1436;
1374229	+	TSS_009643	1000	1059	0	0	Ai	antisense to gene(s) PMM1436;
1374236	+	TSS_009644	1000	160	0	0	Ai	antisense to gene(s) PMM1436;
1374253	+	TSS_009646	1000	542	0	1	Ai	antisense to gene(s) PMM1436;
1374254	-	TSS_022327	1000	591	0	15	I	within gene(s) PMM1436;
1374273	+	TSS_009647	1000	226	0	0	Ai	antisense to gene(s) PMM1436;
1374296	-	TSS_022338	1000	727	0	24	I	within gene(s) PMM1436;
1374323	-	TSS_022344	1000	572	0	27	I	within gene(s) PMM1436;
1374347	-	TSS_022352	1000	209	0	6	I	within gene(s) PMM1436;
1374365	-	TSS_022355	1000	169	0	3	I	within gene(s) PMM1436;
1374386	-	TSS_022358	1000	218	0	6	I	within gene(s) PMM1436;
1374404	-	TSS_022361	1000	479	0	12	I	within gene(s) PMM1436;
1374444	+	TSS_009651	1000	410	0	4	Ai	antisense to gene(s) PMM1436;
1374445	-	TSS_022369	1000	675	0	60	I	within gene(s) PMM1436;
1374468	+	TSS_009653	1000	158	0	0	Ai	antisense to gene(s) PMM1436;
1374509	-	TSS_022388	1000	224	0	12	I	within gene(s) PMM1436;
1374560	-	TSS_022401	1000	238	0	51	I	within gene(s) PMM1436;
1374596	-	TSS_022408	1000	204	0	12	I	within gene(s) PMM1436;
1374643	-	TSS_022412	1000	161	0	6	I	within gene(s) PMM1436;
1374653	-	TSS_022414	1000	230	0	0	I	within gene(s) PMM1436;
1374662	-	TSS_022415	1000	214	0	3	I	within gene(s) PMM1436;
1374674	-	TSS_022417	1000	252	0	3	I	within gene(s) PMM1436;
1374675	+	TSS_009659	1000	116	0	2	Ai	antisense to gene(s) PMM1436;
1374692	-	TSS_022421	1000	635	0	12	I	within gene(s) PMM1436;
1374706	+	TSS_009661	1000	282	0	0	Ai	antisense to gene(s) PMM1436;
1374707	-	TSS_022425	1000	136	0	0	I	within gene(s) PMM1436;
1374722	-	TSS_022426	1000	203	0	0	I	within gene(s) PMM1436;
1374752	-	TSS_022429	1000	616	0	31	I	within gene(s) PMM1436;
1374806	-	TSS_022443	1000	2357	0	30	I	within gene(s) PMM1436;
1374836	-	TSS_022449	1000	122	1.10E-08	0	I	within gene(s) PMM1436;
1374851	-	TSS_022451	1000	483	0	9	I	within gene(s) PMM1436;
1374887	+	TSS_009662	1000	392	0	0	Ai	antisense to gene(s) PMM1436;
1374911	-	TSS_022469	1000	406	0	60	I	within gene(s) PMM1436;
1374953	-	TSS_022478	1000	232	0	21	I	within gene(s) PMM1436;
1374974	-	TSS_022483	1000	154	0	15	I	within gene(s) PMM1436;
1375031	-	TSS_022493	1000	221	0	21	I	within gene(s) PMM1436;
1375038	+	TSS_009664	1000	501	0	0	Ai	antisense to gene(s) PMM1436;
1375058	-	TSS_022499	1000	573	0	1	I	within gene(s) PMM1436;
1375076	-	TSS_022502	1000	113	0	9	I	within gene(s) PMM1436;
1375139	-	TSS_022505	1000	165	0	6	I	within gene(s) PMM1436;
1375217	-	TSS_022514	1000	291	0	12	I	within gene(s) PMM1436;
1375244	-	TSS_022519	1000	236	0	15	I	within gene(s) PMM1436;
1375257	+	TSS_009668	1000	3530	0	1	Ai	antisense to gene(s) PMM1436;
1375334	-	TSS_022523	1000	241	0	0	IP	within gene(s) PMM1437; 60nt upstream of gene PMM1436;

1375343	-	TSS_022525	1000	610	0	1	IP	within gene(s) PMM1437; 69nt upstream of gene PMM1436;
1375373	-	TSS_022528	1000	317	0	21	IP	within gene(s) PMM1437; 99nt upstream of gene PMM1436;
1375391	+	TSS_009669	1000	200	0	1	Ai	antisense to gene(s) PMM1437;
1375404	+	TSS_009671	1000	142	0	0	Ai	antisense to gene(s) PMM1437;
1375412	-	TSS_022538	1000	336	0	14	IP	within gene(s) PMM1437; 138nt upstream of gene PMM1436;
1375436	-	TSS_022542	1000	228	0	4	IP	within gene(s) PMM1437; 162nt upstream of gene PMM1436;
1375451	-	TSS_022544	1000	131	0	2	IP	within gene(s) PMM1437; 177nt upstream of gene PMM1436;
1375472	-	TSS_022546	1000	359	0	0	IP	within gene(s) PMM1437; 198nt upstream of gene PMM1436;
1375502	-	TSS_022554	1000	358	0	21	IP	within gene(s) PMM1437; 228nt upstream of gene PMM1436;
1375529	-	TSS_022555	1000	113	0	0	I	within gene(s) PMM1437;
1375544	-	TSS_022558	1000	281	0	9	I	within gene(s) PMM1437;
1375586	-	TSS_022567	1000	737	0	21	I	within gene(s) PMM1437;
1375613	-	TSS_022568	1000	454	0	18	I	within gene(s) PMM1437;
1375640	-	TSS_022573	1000	374	0	0	P	3nt upstream of gene PMM1437;
1375676	-	TSS_022574	1000	131	0	0	P	39nt upstream of gene PMM1437;
1375697	-	TSS_022575	1000	402	0	0	P	60nt upstream of gene PMM1437;
1375837	+	TSS_009676	1000	909	0	0	P	21nt upstream of gene PMM1438;
1376056	+	TSS_009683	1000	102	0	6	I	within gene(s) PMM1438;
1376113	+	TSS_009690	1000	213	0	2	I	within gene(s) PMM1438;
1376221	+	TSS_009698	1000	224	0	1	I	within gene(s) PMM1438;
1376350	+	TSS_009706	1000	101	0	0	I	within gene(s) PMM1438;
1376400	-	TSS_022581	1000	233	0	1	Ai	antisense to gene(s) PMM1438;
1376407	+	TSS_009717	1000	611	0	16	I	within gene(s) PMM1438;
1376440	-	TSS_022584	1000	484	0	0	Ai	antisense to gene(s) PMM1438;
1376473	+	TSS_009727	1000	177	0	6	I	within gene(s) PMM1438;
1376500	+	TSS_009729	1000	211	0	6	I	within gene(s) PMM1438;
1376542	+	TSS_009734	1000	188	0	11	I	within gene(s) PMM1438;
1376547	-	TSS_022589	1000	564	0	4	Ai	antisense to gene(s) PMM1438;
1376572	+	TSS_009738	1000	227	0	6	I	within gene(s) PMM1438;
1376665	+	TSS_009744	1000	257	0	36	I	within gene(s) PMM1438;
1376719	+	TSS_009757	1000	152	0	12	I	within gene(s) PMM1438;
1376761	+	TSS_009764	1000	161	0	18	I	within gene(s) PMM1438;
1376776	+	TSS_009766	1000	218	0	1	I	within gene(s) PMM1438;
1376893	+	TSS_009770	1000	141	0	12	I	within gene(s) PMM1438;
1377151	+	TSS_009796	1000	153	0	19	IP	within gene(s) PMM1438; 216nt upstream of gene PMM1439;
1377944	+	TSS_009824	1000	218	0	1	Ai	antisense to gene(s) PMM1440;
1378081	+	TSS_009827	1000	177	0	0	Ai	antisense to gene(s) PMM1440;
1378110	-	TSS_022617	1000	158	0	6	I	within gene(s) PMM1440;
1378250	-	TSS_022623	1000	250	0	0	I	within gene(s) PMM1440;
1378674	+	TSS_009831	1000	251	0	2	Ai	antisense to gene(s) PMM1442;
1379477	+	TSS_009835	1000	8362	0	7	Ai	antisense to gene(s) PMM1442;
1379514	+	TSS_009837	1000	142	0	0	Ai	antisense to gene(s) PMM1442;
1379556	+	TSS_009838	1000	1235	0	0	Ai	antisense to gene(s) PMM1442;
1380019	+	TSS_009844	1000	270	0	6	I	within gene(s) PMM1443;
1380027	+	TSS_009845	1000	237	0	0	I	within gene(s) PMM1443;
1380876	+	TSS_009853	1000	2074	0	1	IP	within gene(s) PMM1443; 80nt upstream of gene PMM1444;
1382749	+	TSS_009860	1000	105	0	0	IP	within gene(s) PMM1445; 31nt upstream of gene PMM1446;
1385792	+	TSS_009869	1000	715	0	3	Ai	antisense to gene(s) PMM1449;
1385823	+	TSS_009871	1000	124	0	0	Ai	antisense to gene(s) PMM1449;
1386095	+	TSS_009873	1000	372	0	1	Ai	antisense to gene(s) PMM1450;
1386549	+	TSS_009875	1000	109	0	0	Ai	antisense to gene(s) PMM1450;
1386617	-	TSS_022660	1000	115	0	15	I	within gene(s) PMM1450;
1386624	+	TSS_009878	1000	213	0	1	Ai	antisense to gene(s) PMM1450;
1386653	-	TSS_022667	1000	105	0	19	I	within gene(s) PMM1450;
1386700	+	TSS_009879	1000	288	0	0	Ai	antisense to gene(s) PMM1450;
1386725	-	TSS_022679	1000	143	0	42	I	within gene(s) PMM1450;
1386887	-	TSS_022700	1000	258	0	13	I	within gene(s) PMM1450;
1386935	-	TSS_022707	1000	107	0	11	I	within gene(s) PMM1450;
1387001	-	TSS_022717	1000	112	0	9	I	within gene(s) PMM1450;
1387066	+	TSS_009880	1000	182	0	1	Ai	antisense to gene(s) PMM1451;
1387078	+	TSS_009882	1000	106	0	0	Ai	antisense to gene(s) PMM1451;
1387102	-	TSS_022729	1000	113	0	15	IP	within gene(s) PMM1451; 59nt upstream of gene PMM1450;
1387132	-	TSS_022734	1000	112	0	3	IP	within gene(s) PMM1451; 89nt upstream of gene PMM1450;
1387162	-	TSS_022737	1000	119	0	6	IP	within gene(s) PMM1451; 119nt upstream of gene PMM1450;
1387408	-	TSS_022763	1000	122	0	3	I	within gene(s) PMM1451;
1387543	-	TSS_022776	1000	186	0	3	I	within gene(s) PMM1451;
1387588	-	TSS_022783	1000	294	0	0	I	within gene(s) PMM1451;
1387615	-	TSS_022785	1000	184	0	15	I	within gene(s) PMM1451;
1387660	-	TSS_022794	1000	230	0	6	I	within gene(s) PMM1451;
1387661	+	TSS_009889	1000	291	0	2	Ai	antisense to gene(s) PMM1451;
1387681	-	TSS_022798	1000	194	0	1	I	within gene(s) PMM1451;
1387738	-	TSS_022802	1000	117	0	0	I	within gene(s) PMM1451;
1387867	-	TSS_022814	1000	237	0	5	I	within gene(s) PMM1451;
1387900	-	TSS_022821	1000	364	0	24	I	within gene(s) PMM1451;
1387904	+	TSS_009895	1000	146	0	8	Ai	antisense to gene(s) PMM1451;
1387921	-	TSS_022827	1000	261	0	0	I	within gene(s) PMM1451;
1387936	-	TSS_022830	1000	236	0	18	I	within gene(s) PMM1451;
1387990	-	TSS_022834	1000	390	0	3	I	within gene(s) PMM1451;
1388011	-	TSS_022837	1000	406	0	12	I	within gene(s) PMM1451;

1388096	-	TSS_022844	1000	116	0	0	I	within gene(s) PMM1451;
1388119	-	TSS_022845	1000	127	0	0	I	within gene(s) PMM1451;
1388140	-	TSS_022847	1000	639	0	12	I	within gene(s) PMM1451;
1388164	-	TSS_022850	1000	326	0	6	I	within gene(s) PMM1451;
1388180	+	TSS_009904	1000	775	0	6	Ai	antisense to gene(s) PMM1451;
1388188	-	TSS_022856	1000	485	0	18	I	within gene(s) PMM1451;
1388209	-	TSS_022860	1000	188	0	7	I	within gene(s) PMM1451;
1388224	-	TSS_022862	1000	384	0	6	I	within gene(s) PMM1451;
1388251	-	TSS_022865	1000	204	0	1	I	within gene(s) PMM1451;
1388308	-	TSS_022881	1000	571	0	49	I	within gene(s) PMM1451;
1388326	-	TSS_022883	1000	454	0	0	I	within gene(s) PMM1451;
1388338	-	TSS_022885	1000	197	0	7	I	within gene(s) PMM1451;
1388346	+	TSS_009909	1000	193	0	1	Ai	antisense to gene(s) PMM1451;
1388353	-	TSS_022889	1000	123	0	0	I	within gene(s) PMM1451;
1388530	-	TSS_022904	1000	374	0	9	I	within gene(s) PMM1451;
1388669	-	TSS_022915	1000	3658	0	8	IP	within gene(s) PMM1452; 88nt upstream of gene PMM1451;
1388726	-	TSS_022922	1000	189	0	9	IP	within gene(s) PMM1452; 145nt upstream of gene PMM1451;
1388825	-	TSS_022925	1000	302	0	3	IP	within gene(s) PMM1452; 244nt upstream of gene PMM1451;
1389346	-	TSS_022943	1000	1030	0	13	IP	within gene(s) PMM1453; 191nt upstream of gene PMM1452;
1389389	+	TSS_009916	1000	138	0	9	Ai	antisense to gene(s) PMM1453;
1390725	-	TSS_022970	1000	160	0	0	I	within gene(s) PMM1456;
1390773	-	TSS_022973	1000	120	0	2	I	within gene(s) PMM1456;
1390805	-	TSS_022976	1000	190	0	15	I	within gene(s) PMM1456;
1391900	-	TSS_023018	1000	173	0	1	P	44nt upstream of gene PMM1457;
1392904	-	TSS_023021	1000	132	0	0	Ai	antisense to gene(s) PMM1458;
1393119	+	TSS_009926	1000	114	0	0	I	within gene(s) PMM1458;
1393186	+	TSS_009927	1000	414	0	0	I	within gene(s) PMM1458;
1393368	+	TSS_009929	1000	1342	0	1	P	69nt upstream of gene PMM1459;
1393733	+	TSS_009934	1000	101	0	0	I	within gene(s) PMM1459;
1395953	+	TSS_009937	1000	1201	0	1	P	14nt upstream of gene PMM1462;
1396223	+	TSS_009945	1000	3110	0	4	P	14nt upstream of gene PMM1463;
1397666	+	TSS_009961	1000	2452	0	2	P	16nt upstream of gene PMM1465;
1398489	+	TSS_009981	1000	176	0	9	I	within gene(s) PMM1465;
1398534	+	TSS_009986	1000	107	0	1	I	within gene(s) PMM1465;
1401712	-	TSS_023039	1000	107	0	0	I	within gene(s) PMM1467;
1403187	-	TSS_023044	1000	1071	0	1	P	46nt upstream of gene PMM1467;
1411576	-	TSS_023074	1000	8556	0	10	P	17nt upstream of gene PMM1479;
1412048	+	TSS_010017	1000	228	0	0	Ai	antisense to gene(s) PMM1480;
1412609	-	TSS_023081	1000	345	0	0	P	161nt upstream of gene PMM1480;
1413912	+	TSS_010021	1000	538	0	0	O	-
1414000	+	TSS_010023	1000	104	0	0	Ai	antisense to gene(s) PMM1483;
1414077	-	TSS_023094	1000	112	0	1	IP	within gene(s) PMM1483; 184nt upstream of gene PMM1482;
1414293	-	TSS_023100	1000	136	0	1	I	within gene(s) PMM1483;
1414327	+	TSS_010027	1000	127	0	7	Ai	antisense to gene(s) PMM1483;
1414329	-	TSS_023105	1000	111	0	0	I	within gene(s) PMM1483;
1414401	-	TSS_023115	1000	142	0	3	I	within gene(s) PMM1483;
1414437	-	TSS_023121	1000	196	0	6	I	within gene(s) PMM1483;
1414482	-	TSS_023126	1000	194	0	15	I	within gene(s) PMM1483;
1414653	-	TSS_023138	1000	288	0	12	I	within gene(s) PMM1483;
1414707	-	TSS_023147	1000	113	0	15	I	within gene(s) PMM1483;
1414911	-	TSS_023166	1000	141	0	15	I	within gene(s) PMM1483;
1414936	+	TSS_010038	1000	199	0	0	Ai	antisense to gene(s) PMM1483;
1414968	-	TSS_023172	1000	681	0	10	I	within gene(s) PMM1483;
1414998	-	TSS_023177	1000	135	0	21	I	within gene(s) PMM1483;
1415040	-	TSS_023184	1000	188	0	0	I	within gene(s) PMM1483;
1415079	-	TSS_023185	1000	137	0	3	I	within gene(s) PMM1483;
1415124	-	TSS_023189	1000	135	0	1	I	within gene(s) PMM1483;
1415133	-	TSS_023191	1000	303	0	19	I	within gene(s) PMM1483;
1415169	-	TSS_023197	1000	258	0	12	I	within gene(s) PMM1483;
1415199	-	TSS_023203	1000	281	0	7	I	within gene(s) PMM1483;
1415214	-	TSS_023205	1000	124	0	4	I	within gene(s) PMM1483;
1415226	+	TSS_010044	1000	199	0	0	Ai	antisense to gene(s) PMM1483;
1415400	-	TSS_023217	1000	506	0	12	I	within gene(s) PMM1483;
1415466	-	TSS_023227	1000	239	0	18	I	within gene(s) PMM1483;
1415715	+	TSS_010047	1000	187	0	0	Ai	antisense to gene(s) PMM1483;
1415901	-	TSS_023248	1000	125	0	15	I	within gene(s) PMM1483;
1415907	+	TSS_010050	1000	605	0	2	Ai	antisense to gene(s) PMM1483;
1415943	-	TSS_023254	1000	122	0	0	I	within gene(s) PMM1483;
1415974	+	TSS_010052	1000	116	0	0	Ai	antisense to gene(s) PMM1483;
1415981	-	TSS_023256	1000	113	0	0	I	within gene(s) PMM1483;
1416012	-	TSS_023261	1000	298	0	18	I	within gene(s) PMM1483;
1416036	-	TSS_023266	1000	375	0	30	I	within gene(s) PMM1483;
1416081	-	TSS_023280	1000	296	0	42	I	within gene(s) PMM1483;
1416227	-	TSS_023296	1000	244	0	1	I	within gene(s) PMM1483;
1416255	-	TSS_023300	1000	163	0	39	I	within gene(s) PMM1483;
1416383	+	TSS_010059	1000	135	0	0	Ai	antisense to gene(s) PMM1483;
1416401	+	TSS_010060	1000	444	0	0	Ai	antisense to gene(s) PMM1483;
1416408	-	TSS_023319	1000	108	0	19	I	within gene(s) PMM1483;
1416430	+	TSS_010062	1000	878	0	6	Ai	antisense to gene(s) PMM1483;

1416495	-	TSS_023332	1000	166	0	0 I	within gene(s) PMM1483;
1416518	+	TSS_010065	1000	152	0	0 Ai	antisense to gene(s) PMM1483;
1416930	-	TSS_023365	1000	137	0	0 I	within gene(s) PMM1483;
1416931	+	TSS_010071	1000	200	0	0 Ai	antisense to gene(s) PMM1483;
1416963	-	TSS_023370	1000	119	0	6 I	within gene(s) PMM1483;
1416988	+	TSS_010072	1000	137	0	0 Ai	antisense to gene(s) PMM1483;
1416999	-	TSS_023376	1000	2663	0	15 I	within gene(s) PMM1483;
1417014	-	TSS_023382	1000	190	0	0 I	within gene(s) PMM1483;
1417047	+	TSS_010074	1000	127	0	1 Ai	antisense to gene(s) PMM1483;
1417062	-	TSS_023388	1000	317	0	10 I	within gene(s) PMM1483;
1417098	-	TSS_023392	1000	195	0	9 I	within gene(s) PMM1483;
1417128	-	TSS_023396	1000	308	0	0 I	within gene(s) PMM1483;
1417253	+	TSS_010076	1000	197	0	0 Ai	antisense to gene(s) PMM1483;
1417319	+	TSS_010080	1000	281	0	7 Ai	antisense to gene(s) PMM1483;
1417332	+	TSS_010082	1000	275	0	0 Ai	antisense to gene(s) PMM1483;
1417470	-	TSS_023435	1000	118	0	0 I	within gene(s) PMM1483;
1417512	-	TSS_023442	1000	172	0	30 I	within gene(s) PMM1483;
1417554	-	TSS_023451	1000	183	0	3 I	within gene(s) PMM1483;
1417887	-	TSS_023478	1000	317	0	9 I	within gene(s) PMM1483;
1418150	+	TSS_010089	1000	1074	0	0 Ai	antisense to gene(s) PMM1484;
1418301	+	TSS_010096	1000	208	0	0 Ai	antisense to gene(s) PMM1484;
1418436	-	TSS_023508	1000	112	0	0 I	within gene(s) PMM1484;
1418469	-	TSS_023513	1000	208	0	18 I	within gene(s) PMM1484;
1418489	+	TSS_010097	1000	681	0	4 Ai	antisense to gene(s) PMM1484;
1418507	+	TSS_010099	1000	1756	0	1 Ai	antisense to gene(s) PMM1484;
1418673	-	TSS_023526	1000	139	0	14 I	within gene(s) PMM1484;
1418723	-	TSS_023534	1000	203	0	23 I	within gene(s) PMM1484;
1418763	-	TSS_023541	1000	206	0	6 I	within gene(s) PMM1484;
1418780	+	TSS_010103	1000	382	0	2 Ai	antisense to gene(s) PMM1484;
1418902	+	TSS_010107	1000	206	0	4 Ai	antisense to gene(s) PMM1484;
1418919	-	TSS_023554	1000	150	0	4 I	within gene(s) PMM1484;
1418948	-	TSS_023556	1000	190	0	2 I	within gene(s) PMM1484;
1418994	-	TSS_023565	1000	334	0	45 I	within gene(s) PMM1484;
1419034	+	TSS_010111	1000	430	0	9 Ai	antisense to gene(s) PMM1484;
1419062	+	TSS_010113	1000	178	0	0 Ai	antisense to gene(s) PMM1484;
1419177	-	TSS_023576	1000	433	0	17 I	within gene(s) PMM1484;
1419214	+	TSS_010114	1000	426	0	0 Ai	antisense to gene(s) PMM1484;
1419222	-	TSS_023587	1000	238	0	0 I	within gene(s) PMM1484;
1419267	-	TSS_023595	1000	851	0	5 I	within gene(s) PMM1484;
1419332	+	TSS_010115	1000	124	0	0 Ai	antisense to gene(s) PMM1484;
1419537	-	TSS_023610	1000	102	0	3 I	within gene(s) PMM1484;
1419648	+	TSS_010121	1000	612	0	0 Ai	antisense to gene(s) PMM1484;
1419651	-	TSS_023614	1000	685	0	12 I	within gene(s) PMM1484;
1419666	-	TSS_023620	1000	388	0	28 I	within gene(s) PMM1484;
1419730	+	TSS_010123	1000	494	0	0 Ai	antisense to gene(s) PMM1484;
1419792	-	TSS_023637	1000	147	0	3 I	within gene(s) PMM1484;
1419819	-	TSS_023641	1000	223	0	0 I	within gene(s) PMM1484;
1419883	+	TSS_010127	1000	443	0	1 Ai	antisense to gene(s) PMM1484;
1420037	-	TSS_023652	1000	236	0	1 IP	within gene(s) PMM1485; 56nt upstream of gene PMM1484;
1420066	-	TSS_023656	1000	128	0	9 IP	within gene(s) PMM1485; 85nt upstream of gene PMM1484;
1420171	-	TSS_023668	1000	273	0	10 IP	within gene(s) PMM1485; 190nt upstream of gene PMM1484;
1420202	-	TSS_023673	1000	158	0	3 IP	within gene(s) PMM1485; 221nt upstream of gene PMM1484;
1420412	-	TSS_023686	1000	200	0	7 I	within gene(s) PMM1485;
1420518	+	TSS_010131	1000	226	0	2 Ai	antisense to gene(s) PMM1485;
1420690	+	TSS_010134	1000	121	0	0 Ai	antisense to gene(s) PMM1485;
1420706	-	TSS_023707	1000	550	0	0 I	within gene(s) PMM1485;
1420720	+	TSS_010137	1000	563	0	4 Ai	antisense to gene(s) PMM1485;
1420736	-	TSS_023708	1000	158	0	0 I	within gene(s) PMM1485;
1420845	+	TSS_010142	1000	147	0	3 Ai	antisense to gene(s) PMM1485;
1420925	-	TSS_023721	1000	111	0	3 I	within gene(s) PMM1485;
1420978	-	TSS_023724	1000	124	0	0 I	within gene(s) PMM1485;
1421000	-	TSS_023726	1000	127	0	6 I	within gene(s) PMM1485;
1421015	+	TSS_010147	1000	248	0	3 Ai	antisense to gene(s) PMM1485;
1421414	-	TSS_023748	1000	103	0	13 I	within gene(s) PMM1485;
1421475	+	TSS_010149	1000	132	0	0 Ai	antisense to gene(s) PMM1485;
1421499	+	TSS_010150	1000	170	0	0 Ai	antisense to gene(s) PMM1485;
1421567	+	TSS_010151	1000	125	0	0 Ai	antisense to gene(s) PMM1485;
1421594	-	TSS_023756	1000	197	0	4 I	within gene(s) PMM1485;
1421612	-	TSS_023759	1000	303	0	19 I	within gene(s) PMM1485;
1421688	+	TSS_010153	1000	426	0	1 Ai	antisense to gene(s) PMM1485;
1421690	-	TSS_023769	1000	124	0	0 I	within gene(s) PMM1485;
1421699	+	TSS_010157	1000	107	0	3 Ai	antisense to gene(s) PMM1485;
1422014	-	TSS_023780	1000	158	0	12 I	within gene(s) PMM1485;
1422142	+	TSS_010163	1000	350	0	0 Ai	antisense to gene(s) PMM1485;
1422371	-	TSS_023801	1000	122	0	3 I	within gene(s) PMM1485;
1422611	-	TSS_023807	1000	131	0	3 I	within gene(s) PMM1485;
1422752	-	TSS_023812	1000	324	0	2 I	within gene(s) PMM1485;
1422856	-	TSS_023814	1000	580	0	0 I	within gene(s) PMM1485;
1423046	-	TSS_023817	1000	117	0	0 I	within gene(s) PMM1485;

1423524	-	TSS_023822	1000	778	0	5 P	211nt upstream of gene PMM1485;
1423961	+	TSS_010170	1000	380	0	1 Ai	antisense to gene(s) PMM1486;
1424666	-	TSS_023832	1000	208	0	6 P	20nt upstream of gene PMM1487;
1426785	-	TSS_023852	1000	576	0	3 P	38nt upstream of gene PMM1489;
1427278	+	TSS_010185	1000	271	0	2 Ai	antisense to gene(s) PMM1490;
1428507	+	TSS_010187	1000	254	0	0 IP	within gene(s) PMM1491; 98nt upstream of gene PMM1492;
1428986	+	TSS_010196	1000	129	0	0 I	within gene(s) PMM1492;
1429041	-	TSS_023868	1000	120	0	0 Ai	antisense to gene(s) PMM1492;
1429590	+	TSS_010218	1000	297	0	9 I	within gene(s) PMM1492;
1429682	+	TSS_010225	1000	149	0	12 I	within gene(s) PMM1492;
1429712	+	TSS_010231	1000	235	0	10 I	within gene(s) PMM1492;
1429727	+	TSS_010235	1000	269	0	3 I	within gene(s) PMM1492;
1429742	+	TSS_010237	1000	255	0	1 I	within gene(s) PMM1492;
1431083	+	TSS_010259	1000	117	0	0 I	within gene(s) PMM1494;
1431142	+	TSS_010263	1000	649	0	7 I	within gene(s) PMM1494;
1431241	+	TSS_010267	1000	543	0	7 I	within gene(s) PMM1494;
1431352	+	TSS_010273	1000	130	0	1 I	within gene(s) PMM1494;
1431379	+	TSS_010275	1000	142	0	0 I	within gene(s) PMM1494;
1431397	+	TSS_010278	1000	105	0	6 I	within gene(s) PMM1494;
1431469	+	TSS_010282	1000	331	0	0 I	within gene(s) PMM1494;
1431481	+	TSS_010285	1000	471	0	3 I	within gene(s) PMM1494;
1431508	+	TSS_010289	1000	145	0	3 I	within gene(s) PMM1494;
1431529	+	TSS_010293	1000	312	0	12 I	within gene(s) PMM1494;
1431553	+	TSS_010295	1000	112	0	0 I	within gene(s) PMM1494;
1431808	+	TSS_010308	1000	122	0	6 I	within gene(s) PMM1494;
1431838	+	TSS_010309	1000	348	0	1 I	within gene(s) PMM1494;
1431862	+	TSS_010316	1000	160	0	21 I	within gene(s) PMM1494;
1431913	+	TSS_010325	1000	143	0	9 I	within gene(s) PMM1494;
1432697	-	TSS_023901	1000	189	0	0 Ai	antisense to gene(s) PMM1494;
1432963	+	TSS_010353	1000	183	0	1 I	within gene(s) PMM1494;
1432991	+	TSS_010357	1000	512	0	4 I	within gene(s) PMM1494;
1433023	+	TSS_010360	1000	771	0	7 I	within gene(s) PMM1494;
1433164	+	TSS_010364	1000	165	0	0 I	within gene(s) PMM1494;
1433296	+	TSS_010367	1000	322	0	0 I	within gene(s) PMM1494;
1433326	+	TSS_010368	1000	156	0	6 I	within gene(s) PMM1494;
1435445	+	TSS_010380	1000	138	0	0 I	within gene(s) PMM1496;
1436999	+	TSS_010384	1000	708	0	0 P	24nt upstream of gene PMM1498;
1437357	-	TSS_023917	1000	526	0	1 Ai	antisense to gene(s) PMM1498;
1438103	+	TSS_010415	1000	175	0	7 I	within gene(s) PMM1498;
1439023	-	TSS_023928	1000	771	0	0 I	within gene(s) PMM1499;
1439138	+	TSS_010424	1000	242	0	0 Ai	antisense to gene(s) PMM1500;
1439203	-	TSS_023931	1000	120	0	0 IP	within gene(s) PMM1500; 170nt upstream of gene PMM1499;
1439379	-	TSS_023937	1000	116	0	4 I	within gene(s) PMM1500;
1439399	+	TSS_010427	1000	145	0	0 Ai	antisense to gene(s) PMM1500;
1439422	-	TSS_023941	1000	130	0	0 I	within gene(s) PMM1500;
1439647	-	TSS_023953	1000	101	0	1 I	within gene(s) PMM1500;
1440282	-	TSS_023971	1000	1043	0	1 P	23nt upstream of gene PMM1500;
1440561	+	TSS_010434	1000	168	0	0 I	within gene(s) PMM1501;
1443305	+	TSS_010441	1000	1408	0	2 PAi	201nt upstream of gene PMM1504; antisense to gene(s) PMM1503;
1443593	-	TSS_023980	1000	551	0	0 PAi	140nt upstream of gene PMM1503; antisense to gene(s) PMM1504;
1446052	+	TSS_010448	1000	147	0	0 Ai	antisense to gene(s) PMM1507;
1446371	-	TSS_023996	1000	124	0	1 P	51nt upstream of gene PMM1507;
1446380	-	TSS_023999	1000	3710	0	8 P	60nt upstream of gene PMM1507;
1446495	-	TSS_024007	1000	112	0	15 IP	within gene(s) PMM1508; 175nt upstream of gene PMM1507;
1446507	+	TSS_010456	1000	181	0	2 Ai	antisense to gene(s) PMM1508;
1446537	-	TSS_024012	1000	184	0	1 IP	within gene(s) PMM1508; 217nt upstream of gene PMM1507;
1446580	+	TSS_010461	1000	354	0	0 Ai	antisense to gene(s) PMM1508;
1446684	-	TSS_024016	1000	206	0	7 I	within gene(s) PMM1508;
1446705	-	TSS_024018	1000	294	0	12 I	within gene(s) PMM1508;
1446732	-	TSS_024024	1000	183	0	12 I	within gene(s) PMM1508;
1446753	-	TSS_024028	1000	402	0	15 I	within gene(s) PMM1508;
1446789	-	TSS_024036	1000	143	0	12 I	within gene(s) PMM1508;
1446807	-	TSS_024039	1000	129	0	6 I	within gene(s) PMM1508;
1446814	+	TSS_010469	1000	403	0	6 Ai	antisense to gene(s) PMM1508;
1446816	-	TSS_024041	1000	163	0	1 I	within gene(s) PMM1508;
1446855	-	TSS_024048	1000	174	0	24 I	within gene(s) PMM1508;
1446894	-	TSS_024052	1000	125	0	1 I	within gene(s) PMM1508;
1446906	-	TSS_024054	1000	151	0	9 I	within gene(s) PMM1508;
1446963	-	TSS_024064	1000	149	0	15 I	within gene(s) PMM1508;
1446987	-	TSS_024065	1000	461	0	7 I	within gene(s) PMM1508;
1447023	-	TSS_024077	1000	800	0	27 I	within gene(s) PMM1508;
1447047	+	TSS_010471	1000	157	0	0 Ai	antisense to gene(s) PMM1508;
1447050	-	TSS_024082	1000	638	0	9 I	within gene(s) PMM1508;
1447058	+	TSS_010473	1000	2374	0	1 Ai	antisense to gene(s) PMM1508;
1447062	-	TSS_024083	1000	124	0	0 I	within gene(s) PMM1508;
1447079	+	TSS_010474	1000	501	0	0 Ai	antisense to gene(s) PMM1508;
1447086	-	TSS_024084	1000	3366	0	0 I	within gene(s) PMM1508;
1447098	-	TSS_024085	1000	1887	0	3 I	within gene(s) PMM1508;
1447116	-	TSS_024087	1000	131	0	0 I	within gene(s) PMM1508;

1447125	-	TSS_024088	1000	747	0	81 I	within gene(s) PMM1508;
1447224	+	TSS_010475	1000	228	0	0 Ai	antisense to gene(s) PMM1508;
1447254	-	TSS_024121	1000	130	0	30 I	within gene(s) PMM1508;
1447398	-	TSS_024134	1000	119	0	18 I	within gene(s) PMM1508;
1447433	-	TSS_024136	1000	245	0	0 I	within gene(s) PMM1508;
1447577	-	TSS_024151	1000	144	0	14 I	within gene(s) PMM1508;
1447617	-	TSS_024156	1000	121	0	0 I	within gene(s) PMM1508;
1447634	+	TSS_010481	1000	702	0	1 O	-
1447711	-	TSS_024157	1000	486	0	0 IP	within gene(s) PMM1509; 82nt upstream of gene PMM1508;
1447723	-	TSS_024159	1000	186	0	3 IP	within gene(s) PMM1509; 94nt upstream of gene PMM1508;
1447735	-	TSS_024161	1000	399	0	11 IP	within gene(s) PMM1509; 106nt upstream of gene PMM1508;
1447786	-	TSS_024166	1000	266	0	3 IP	within gene(s) PMM1509; 157nt upstream of gene PMM1508;
1447795	-	TSS_024167	1000	161	0	3 IP	within gene(s) PMM1509; 166nt upstream of gene PMM1508;
1447813	-	TSS_024169	1000	385	0	0 IP	within gene(s) PMM1509; 184nt upstream of gene PMM1508;
1447831	-	TSS_024170	1000	345	0	0 IP	within gene(s) PMM1509; 202nt upstream of gene PMM1508;
1447864	-	TSS_024173	1000	493	0	9 IP	within gene(s) PMM1509; 235nt upstream of gene PMM1508;
1447894	-	TSS_024175	1000	182	0	3 I	within gene(s) PMM1509;
1447903	-	TSS_024176	1000	112	0	0 I	within gene(s) PMM1509;
1448269	-	TSS_024190	1000	101	0	12 I	within gene(s) PMM1509;
1448310	-	TSS_024192	1000	275	0	3 I	within gene(s) PMM1509;
1448332	-	TSS_024194	1000	354	0	12 I	within gene(s) PMM1509;
1448434	-	TSS_024203	1000	241	0	6 I	within gene(s) PMM1509;
1448485	-	TSS_024210	1000	1197	0	42 I	within gene(s) PMM1509;
1448548	-	TSS_024223	1000	143	0	3 I	within gene(s) PMM1509;
1448767	-	TSS_024241	1000	273	0	9 I	within gene(s) PMM1509;
1448818	-	TSS_024251	1000	371	0	11 I	within gene(s) PMM1509;
1448845	-	TSS_024257	1000	110	0	6 I	within gene(s) PMM1509;
1448863	-	TSS_024260	1000	328	0	0 I	within gene(s) PMM1509;
1449130	-	TSS_024272	1000	134	0	15 I	within gene(s) PMM1509;
1449152	+	TSS_010495	1000	185	0	1 Ai	antisense to gene(s) PMM1509;
1449247	-	TSS_024282	1000	117	0	21 I	within gene(s) PMM1509;
1449559	-	TSS_024303	1000	125	0	6 I	within gene(s) PMM1509;
1449812	-	TSS_024308	1000	3344	0	5 P	64nt upstream of gene PMM1509;
1449934	+	TSS_010503	1000	104	0	0 Ai	antisense to gene(s) PMM1510;
1449943	+	TSS_010504	1000	200	0	0 Ai	antisense to gene(s) PMM1510;
1450004	+	TSS_010505	1000	286	0	4 Ai	antisense to gene(s) PMM1510;
1450080	-	TSS_024316	1000	140	0	9 I	within gene(s) PMM1510;
1450188	-	TSS_024326	1000	479	0	0 I	within gene(s) PMM1510;
1450209	-	TSS_024329	1000	263	0	4 I	within gene(s) PMM1510;
1450230	-	TSS_024332	1000	200	0	2 I	within gene(s) PMM1510;
1450257	-	TSS_024334	1000	167	0	0 I	within gene(s) PMM1510;
1450269	-	TSS_024335	1000	122	0	0 I	within gene(s) PMM1510;
1450367	-	TSS_024339	1000	134	0	1 IP	within gene(s) PMM1511; 44nt upstream of gene PMM1510;
1450427	-	TSS_024349	1000	1247	0	37 IP	within gene(s) PMM1511; 104nt upstream of gene PMM1510;
1450569	-	TSS_024363	1000	122	0	4 IP	within gene(s) PMM1511; 246nt upstream of gene PMM1510;
1450891	+	TSS_010509	1000	107	0	1 P	31nt upstream of gene PMM1512;
1450919	+	TSS_010511	1000	145	0	0 P	3nt upstream of gene PMM1512;
1454166	+	TSS_010541	1000	140	0	15 I	within gene(s) PMM1512;
1454543	+	TSS_010551	1000	176	0	0 I	within gene(s) PMM1512;
1454745	+	TSS_010553	1000	106	0	1 I	within gene(s) PMM1512;
1456713	-	TSS_024389	1000	2878	0	3 P	20nt upstream of gene PMM1514;
1459395	-	TSS_024395	1000	164	0	0 Ai	antisense to gene(s) PMM1518;
1459609	+	TSS_010568	1000	120	0	0 Ai	antisense to gene(s) PMM1519;
1459813	+	TSS_010571	1000	125	0	0 Ai	antisense to gene(s) PMM1519;
1459836	-	TSS_024398	1000	152	0	3 I	within gene(s) PMM1519;
1460021	-	TSS_024417	1000	139	0	1 I	within gene(s) PMM1519;
1460363	-	TSS_024431	1000	9060	0	3 P	15nt upstream of gene PMM1520;
1462056	+	TSS_010576	1000	473	0	0 Ai	antisense to gene(s) PMM1523;
1462103	-	TSS_024440	1000	193	0	10 I	within gene(s) PMM1523;
1462123	+	TSS_010579	1000	127	0	1 Ai	antisense to gene(s) PMM1523;
1462135	-	TSS_024443	1000	250	0	15 I	within gene(s) PMM1523;
1462153	-	TSS_024446	1000	502	0	24 I	within gene(s) PMM1523;
1462195	-	TSS_024458	1000	1412	0	18 I	within gene(s) PMM1523;
1462225	-	TSS_024467	1000	3184	0	17 I	within gene(s) PMM1523;
1462258	-	TSS_024474	1000	977	0	4 I	within gene(s) PMM1523;
1462266	+	TSS_010581	1000	260	0	0 Ai	antisense to gene(s) PMM1523;
1462270	-	TSS_024477	1000	1858	0	12 I	within gene(s) PMM1523;
1462291	-	TSS_024484	1000	8221	0	13 I	within gene(s) PMM1523;
1462301	+	TSS_010582	1000	139	0	0 Ai	antisense to gene(s) PMM1523;
1462312	-	TSS_024490	1000	611	0	1 I	within gene(s) PMM1523;
1462327	-	TSS_024491	1000	764	0	0 I	within gene(s) PMM1523;
1462339	-	TSS_024492	1000	505	0	0 I	within gene(s) PMM1523;
1462348	-	TSS_024493	1000	530	0	0 I	within gene(s) PMM1523;
1462369	-	TSS_024494	1000	422	0	0 I	within gene(s) PMM1523;
1462387	-	TSS_024495	1000	315	0	0 I	within gene(s) PMM1523;
1462416	-	TSS_024497	1000	9356	0	30 I	within gene(s) PMM1523;
1462468	-	TSS_024509	1000	286	0	6 I	within gene(s) PMM1523;
1462477	-	TSS_024510	1000	143	0	0 I	within gene(s) PMM1523;
1462492	-	TSS_024511	1000	209	0	0 I	within gene(s) PMM1523;

1462501	-	TSS_024513	1000	279	0	1 I	within gene(s) PMM1523;
1462519	-	TSS_024517	1000	544	0	9 I	within gene(s) PMM1523;
1462546	-	TSS_024518	1000	310	0	0 I	within gene(s) PMM1523;
1462555	-	TSS_024519	1000	245	0	2 I	within gene(s) PMM1523;
1462573	-	TSS_024523	1000	732	0	12 I	within gene(s) PMM1523;
1462603	-	TSS_024526	1000	160	0	6 I	within gene(s) PMM1523;
1462630	-	TSS_024528	1000	139	0	0 I	within gene(s) PMM1523;
1462639	-	TSS_024529	1000	378	0	2 I	within gene(s) PMM1523;
1462651	-	TSS_024531	1000	630	0	33 I	within gene(s) PMM1523;
1462696	-	TSS_024544	1000	299	0	6 I	within gene(s) PMM1523;
1462714	-	TSS_024546	1000	758	0	21 I	within gene(s) PMM1523;
1462747	-	TSS_024552	1000	258	0	3 I	within gene(s) PMM1523;
1462771	-	TSS_024559	1000	1904	0	33 I	within gene(s) PMM1523;
1462817	+	TSS_010588	1000	160	0	0 Ai	antisense to gene(s) PMM1523;
1462819	-	TSS_024566	1000	177	0	5 I	within gene(s) PMM1523;
1462831	-	TSS_024568	1000	164	0	0 I	within gene(s) PMM1523;
1462833	+	TSS_010590	1000	241	0	1 Ai	antisense to gene(s) PMM1523;
1462846	-	TSS_024572	1000	2476	0	6 I	within gene(s) PMM1523;
1462857	+	TSS_010591	1000	470	0	0 Ai	antisense to gene(s) PMM1523;
1462873	-	TSS_024578	1000	562	0	30 I	within gene(s) PMM1523;
1462918	-	TSS_024586	1000	213	0	18 I	within gene(s) PMM1523;
1462930	-	TSS_024588	1000	328	0	3 I	within gene(s) PMM1523;
1462947	-	TSS_024589	1000	678	0	1 I	within gene(s) PMM1523;
1462965	-	TSS_024593	1000	180	0	9 I	within gene(s) PMM1523;
1462981	-	TSS_024598	1000	644	0	25 I	within gene(s) PMM1523;
1463008	-	TSS_024603	1000	103	5.30E-06	0 I	within gene(s) PMM1523;
1463026	-	TSS_024604	1000	183	0	0 I	within gene(s) PMM1523;
1463062	-	TSS_024606	1000	545	0	13 I	within gene(s) PMM1523;
1463103	-	TSS_024616	1000	1212	0	12 I	within gene(s) PMM1523;
1463146	-	TSS_024626	1000	689	0	33 I	within gene(s) PMM1523;
1463155	-	TSS_024627	1000	138	0	0 I	within gene(s) PMM1523;
1463179	-	TSS_024628	1000	104	1.10E-07	0 I	within gene(s) PMM1523;
1463197	-	TSS_024632	1000	770	0	39 I	within gene(s) PMM1523;
1463251	-	TSS_024645	1000	325	0	30 I	within gene(s) PMM1523;
1463410	-	TSS_024658	1000	431	0	3 I	within gene(s) PMM1523;
1463421	+	TSS_010596	1000	107	0	0 Ai	antisense to gene(s) PMM1523;
1463428	-	TSS_024662	1000	163	0	33 I	within gene(s) PMM1523;
1463467	-	TSS_024672	1000	202	0	2 I	within gene(s) PMM1523;
1463485	-	TSS_024675	1000	142	0	3 I	within gene(s) PMM1523;
1463515	-	TSS_024679	1000	446	0	24 I	within gene(s) PMM1523;
1463537	+	TSS_010599	1000	170	0	1 Ai	antisense to gene(s) PMM1523;
1463545	-	TSS_024684	1000	471	0	3 I	within gene(s) PMM1523;
1463565	-	TSS_024691	1000	255	0	21 I	within gene(s) PMM1523;
1463595	-	TSS_024699	1000	208	0	15 I	within gene(s) PMM1523;
1463617	-	TSS_024705	1000	104	1.90E-09	0 I	within gene(s) PMM1523;
1463659	-	TSS_024713	1000	362	0	36 I	within gene(s) PMM1523;
1463677	-	TSS_024718	1000	447	0	3 I	within gene(s) PMM1523;
1463689	-	TSS_024721	1000	202	0	0 I	within gene(s) PMM1523;
1463698	-	TSS_024722	1000	247	0	9 I	within gene(s) PMM1523;
1463722	-	TSS_024725	1000	242	0	5 I	within gene(s) PMM1523;
1463734	-	TSS_024728	1000	159	0	3 I	within gene(s) PMM1523;
1463744	+	TSS_010601	1000	153	0	0 Ai	antisense to gene(s) PMM1523;
1463746	-	TSS_024731	1000	555	0	10 I	within gene(s) PMM1523;
1463763	-	TSS_024735	1000	156	0	0 I	within gene(s) PMM1523;
1463781	-	TSS_024737	1000	114	2.60E-14	2 I	within gene(s) PMM1523;
1463821	-	TSS_024741	1000	160	0	15 I	within gene(s) PMM1523;
1463829	+	TSS_010606	1000	544	0	1 Ai	antisense to gene(s) PMM1523;
1463857	-	TSS_024745	1000	905	0	3 I	within gene(s) PMM1523;
1463878	-	TSS_024750	1000	358	0	18 I	within gene(s) PMM1523;
1463899	-	TSS_024756	1000	576	0	6 I	within gene(s) PMM1523;
1463923	-	TSS_024758	1000	324	0	9 I	within gene(s) PMM1523;
1463941	-	TSS_024762	1000	179	0	6 I	within gene(s) PMM1523;
1463950	-	TSS_024763	1000	143	0	0 I	within gene(s) PMM1523;
1463951	+	TSS_010608	1000	230	0	1 Ai	antisense to gene(s) PMM1523;
1463962	-	TSS_024764	1000	248	0	0 I	within gene(s) PMM1523;
1464007	-	TSS_024766	1000	142	0	1 I	within gene(s) PMM1523;
1464180	-	TSS_024767	1000	467	0	0 IP	within gene(s) PMM1524; 47nt upstream of gene PMM1523;
1464188	-	TSS_024768	1000	462	0	0 IP	within gene(s) PMM1524; 55nt upstream of gene PMM1523;
1464233	-	TSS_024769	1000	1283	0	0 IP	within gene(s) PMM1524; 100nt upstream of gene PMM1523;
1464257	-	TSS_024770	1000	330	0	0 IP	within gene(s) PMM1524; 124nt upstream of gene PMM1523;
1464278	-	TSS_024776	1000	5371	0	10 IP	within gene(s) PMM1524; 145nt upstream of gene PMM1523;
1464296	-	TSS_024781	1000	869	0	9 IP	within gene(s) PMM1524; 163nt upstream of gene PMM1523;
1464314	-	TSS_024784	1000	1859	0	12 IP	within gene(s) PMM1524; 181nt upstream of gene PMM1523;
1464338	-	TSS_024791	1000	1965	0	4 IP	within gene(s) PMM1524; 205nt upstream of gene PMM1523;
1464356	-	TSS_024794	1000	372	0	0 IP	within gene(s) PMM1524; 223nt upstream of gene PMM1523;
1464382	+	TSS_010614	1000	129	0	7 Ai	antisense to gene(s) PMM1524;
1464383	-	TSS_024795	1000	366	0	0 IP	within gene(s) PMM1524; 250nt upstream of gene PMM1523;
1464397	-	TSS_024796	1000	373	0	4 I	within gene(s) PMM1524;
1464434	-	TSS_024799	1000	327	0	0 I	within gene(s) PMM1524;

1464464	-	TSS_024804	1000	883	0	12 I	within gene(s) PMM1524;
1464474	-	TSS_024806	1000	475	0	1 I	within gene(s) PMM1524;
1464503	-	TSS_024807	1000	1126	0	0 I	within gene(s) PMM1524;
1464512	-	TSS_024808	1000	1604	0	0 I	within gene(s) PMM1524;
1464527	-	TSS_024809	1000	901	0	9 I	within gene(s) PMM1524;
1464560	-	TSS_024812	1000	480	0	0 I	within gene(s) PMM1524;
1464569	+	TSS_010623	1000	131	0	1 Ai	antisense to gene(s) PMM1524;
1464575	-	TSS_024815	1000	3274	0	9 I	within gene(s) PMM1524;
1464596	-	TSS_024819	1000	508	0	1 I	within gene(s) PMM1524;
1464612	+	TSS_010624	1000	220	0	0 Ai	antisense to gene(s) PMM1524;
1464662	-	TSS_024828	1000	1456	0	36 I	within gene(s) PMM1524;
1464704	-	TSS_024841	1000	4983	0	84 I	within gene(s) PMM1524;
1464785	-	TSS_024871	1000	209	0	0 I	within gene(s) PMM1524;
1464806	-	TSS_024872	1000	627	0	6 I	within gene(s) PMM1524;
1464839	-	TSS_024874	1000	914	0	0 I	within gene(s) PMM1524;
1464848	-	TSS_024875	1000	144	6.30E-06	0 I	within gene(s) PMM1524;
1464869	-	TSS_024876	1000	201	0	0 I	within gene(s) PMM1524;
1464876	+	TSS_010626	1000	531	0	4 Ai	antisense to gene(s) PMM1524;
1464887	+	TSS_010629	1000	590	0	0 Ai	antisense to gene(s) PMM1524;
1464887	-	TSS_024877	1000	191	0	0 I	within gene(s) PMM1524;
1464911	-	TSS_024879	1000	158	0	6 I	within gene(s) PMM1524;
1464927	-	TSS_024882	1000	921	0	18 I	within gene(s) PMM1524;
1464959	+	TSS_010630	1000	1184	0	0 Ai	antisense to gene(s) PMM1524;
1464959	-	TSS_024889	1000	839	0	0 I	within gene(s) PMM1524;
1464968	-	TSS_024890	1000	1478	0	2 I	within gene(s) PMM1524;
1464980	-	TSS_024892	1000	125	0	0 I	within gene(s) PMM1524;
1465007	-	TSS_024894	1000	423	0	6 I	within gene(s) PMM1524;
1465017	+	TSS_010631	1000	188	0	0 Ai	antisense to gene(s) PMM1524;
1465031	-	TSS_024895	1000	169	0	0 I	within gene(s) PMM1524;
1465069	-	TSS_024896	1000	533	0	1 I	within gene(s) PMM1524;
1465079	-	TSS_024898	1000	304	0	15 I	within gene(s) PMM1524;
1465112	-	TSS_024908	1000	274	0	12 I	within gene(s) PMM1524;
1465113	+	TSS_010633	1000	1076	0	8 Ai	antisense to gene(s) PMM1524;
1465127	+	TSS_010637	1000	267	0	0 Ai	antisense to gene(s) PMM1524;
1465139	-	TSS_024912	1000	128	0	12 I	within gene(s) PMM1524;
1465140	+	TSS_010638	1000	130	0	0 Ai	antisense to gene(s) PMM1524;
1465157	-	TSS_024915	1000	301	0	12 I	within gene(s) PMM1524;
1465185	+	TSS_010642	1000	264	0	9 Ai	antisense to gene(s) PMM1524;
1465196	-	TSS_024920	1000	462	0	18 I	within gene(s) PMM1524;
1465197	+	TSS_010643	1000	377	0	6 Ai	antisense to gene(s) PMM1524;
1465286	-	TSS_024926	1000	337	0	27 I	within gene(s) PMM1524;
1465310	-	TSS_024935	1000	131	0	6 I	within gene(s) PMM1524;
1465322	-	TSS_024937	1000	146	0	0 I	within gene(s) PMM1524;
1465341	-	TSS_024940	1000	261	0	4 I	within gene(s) PMM1524;
1465363	-	TSS_024945	1000	934	0	30 I	within gene(s) PMM1524;
1465394	-	TSS_024955	1000	287	0	12 I	within gene(s) PMM1524;
1465412	-	TSS_024959	1000	431	0	0 I	within gene(s) PMM1524;
1465442	-	TSS_024964	1000	589	0	12 I	within gene(s) PMM1524;
1465453	-	TSS_024965	1000	408	0	1 I	within gene(s) PMM1524;
1465475	-	TSS_024967	1000	910	0	3 I	within gene(s) PMM1524;
1465524	+	TSS_010650	1000	117	0	5 Ai	antisense to gene(s) PMM1524;
1465553	-	TSS_024971	1000	370	0	3 I	within gene(s) PMM1524;
1465574	-	TSS_024975	1000	410	0	18 I	within gene(s) PMM1524;
1465592	-	TSS_024978	1000	152	0	0 I	within gene(s) PMM1524;
1465619	-	TSS_024985	1000	569	0	18 I	within gene(s) PMM1524;
1465640	-	TSS_024986	1000	496	0	0 I	within gene(s) PMM1524;
1465661	-	TSS_024992	1000	7318	0	21 I	within gene(s) PMM1524;
1465682	-	TSS_024997	1000	118	0	0 I	within gene(s) PMM1524;
1465694	-	TSS_025000	1000	390	0	10 I	within gene(s) PMM1524;
1465736	-	TSS_025004	1000	183	0	0 I	within gene(s) PMM1524;
1465769	-	TSS_025005	1000	129	0	0 I	within gene(s) PMM1524;
1465793	-	TSS_025008	1000	253	0	12 I	within gene(s) PMM1524;
1465843	-	TSS_025012	1000	319	0	1 I	within gene(s) PMM1524;
1465864	-	TSS_025017	1000	189	0	8 I	within gene(s) PMM1524;
1465874	-	TSS_025018	1000	186	0	0 I	within gene(s) PMM1524;
1465885	-	TSS_025019	1000	332	0	1 I	within gene(s) PMM1524;
1465907	-	TSS_025023	1000	219	0	15 I	within gene(s) PMM1524;
1465964	-	TSS_025030	1000	636	0	21 I	within gene(s) PMM1524;
1465998	+	TSS_010654	1000	276	0	0 Ai	antisense to gene(s) PMM1524;
1466014	-	TSS_025037	1000	202	0	12 I	within gene(s) PMM1524;
1466027	-	TSS_025041	1000	603	0	12 I	within gene(s) PMM1524;
1466034	+	TSS_010655	1000	136	0	0 Ai	antisense to gene(s) PMM1524;
1466051	-	TSS_025046	1000	175	0	3 I	within gene(s) PMM1524;
1466060	-	TSS_025047	1000	109	0	0 I	within gene(s) PMM1524;
1466114	-	TSS_025048	1000	1362	0	2 I	within gene(s) PMM1524;
1466147	-	TSS_025050	1000	140	0	3 I	within gene(s) PMM1524;
1466177	-	TSS_025054	1000	667	0	10 I	within gene(s) PMM1524;
1466180	+	TSS_010658	1000	2245	0	2 Ai	antisense to gene(s) PMM1524;
1466189	-	TSS_025055	1000	690	0	9 I	within gene(s) PMM1524;

1466213	-	TSS_025059	1000	159	0	0	I	within gene(s) PMM1524;
1466231	-	TSS_025061	1000	149	0	3	I	within gene(s) PMM1524;
1466264	-	TSS_025067	1000	219	0	21	I	within gene(s) PMM1524;
1466300	-	TSS_025070	1000	220	0	0	I	within gene(s) PMM1524;
1466330	-	TSS_025072	1000	137	0	0	I	within gene(s) PMM1524;
1466354	-	TSS_025074	1000	273	0	0	I	within gene(s) PMM1524;
1466366	-	TSS_025077	1000	649	0	4	I	within gene(s) PMM1524;
1466429	-	TSS_025081	1000	272	0	7	I	within gene(s) PMM1524;
1466585	-	TSS_025082	1000	1075	0	0	P	120nt upstream of gene PMM1524;
1468500	-	TSS_025086	1000	148	0	0	Ai	antisense to gene(s) PMM1525;
1468533	-	TSS_025088	1000	248	0	0	Ai	antisense to gene(s) PMM1525;
1469172	+	TSS_010666	1000	269	0	0	I	within gene(s) PMM1526;
1470954	+	TSS_010672	1000	531	0	2	P	15nt upstream of gene PMM1528;
1471756	+	TSS_010677	1000	207	0	0	Ai	antisense to gene(s) PMM1529;
1473037	-	TSS_025128	1000	777	0	18	IP	within gene(s) PMM1531; 166nt upstream of gene PMM1530;
1473343	-	TSS_025140	1000	122	0	0	IP	within gene(s) PMM1532; 48nt upstream of gene PMM1531;
1473409	-	TSS_025141	1000	252	0	6	IP	within gene(s) PMM1532; 114nt upstream of gene PMM1531;
1473445	-	TSS_025149	1000	127	0	6	IP	within gene(s) PMM1532; 150nt upstream of gene PMM1531;
1473676	-	TSS_025167	1000	113	0	24	I	within gene(s) PMM1532;
1473735	-	TSS_025170	1000	134	0	2	I	within gene(s) PMM1532;
1473764	-	TSS_025172	1000	3346	0	4	P	28nt upstream of gene PMM1532;
1474126	-	TSS_025177	1000	166	0	0	I	within gene(s) PMM1533;
1474891	-	TSS_025182	1000	141	0	12	IP	within gene(s) PMM1534; 232nt upstream of gene PMM1533;
1474906	-	TSS_025185	1000	378	0	4	IP	within gene(s) PMM1534; 247nt upstream of gene PMM1533;
1474933	-	TSS_025189	1000	192	0	1	I	within gene(s) PMM1534;
1474954	-	TSS_025190	1000	146	0	6	I	within gene(s) PMM1534;
1474993	-	TSS_025194	1000	380	0	9	I	within gene(s) PMM1534;
1475170	-	TSS_025198	1000	137	0	0	IP	within gene(s) PMM1535; 123nt upstream of gene PMM1534;
1475257	-	TSS_025199	1000	518	0	0	IP	within gene(s) PMM1535; 210nt upstream of gene PMM1534;
1475305	-	TSS_025202	1000	812	0	0	I	within gene(s) PMM1535;
1475407	-	TSS_025204	1000	218	0	4	I	within gene(s) PMM1535;
1475507	-	TSS_025212	1000	209	0	0	I	within gene(s) PMM1535;
1475749	-	TSS_025221	1000	103	0	3	I	within gene(s) PMM1535;
1475761	-	TSS_025223	1000	131	0	0	I	within gene(s) PMM1535;
1476176	-	TSS_025241	1000	382	0	21	IP	within gene(s) PMM1536; 175nt upstream of gene PMM1535;
1476251	-	TSS_025249	1000	146	0	12	IP	within gene(s) PMM1536; 250nt upstream of gene PMM1535;
1476308	-	TSS_025255	1000	102	0	12	I	within gene(s) PMM1536;
1476326	-	TSS_025258	1000	561	0	24	I	within gene(s) PMM1536;
1476380	-	TSS_025265	1000	111	0	9	I	within gene(s) PMM1536;
1476390	+	TSS_010693	1000	141	0	0	Ai	antisense to gene(s) PMM1536;
1476428	-	TSS_025272	1000	108	0	1	I	within gene(s) PMM1536;
1476535	-	TSS_025277	1000	121	0	4	IP	within gene(s) PMM1537; 95nt upstream of gene PMM1536;
1476546	+	TSS_010696	1000	206	0	7	Ai	antisense to gene(s) PMM1537;
1476569	-	TSS_025280	1000	157	0	0	IP	within gene(s) PMM1537; 129nt upstream of gene PMM1536;
1476583	+	TSS_010701	1000	169	0	0	Ai	antisense to gene(s) PMM1537;
1476589	-	TSS_025281	1000	178	0	13	IP	within gene(s) PMM1537; 149nt upstream of gene PMM1536;
1476679	+	TSS_010703	1000	196	0	0	Ai	antisense to gene(s) PMM1537;
1476725	-	TSS_025293	1000	433	0	0	I	within gene(s) PMM1537;
1476743	-	TSS_025296	1000	129	0	19	I	within gene(s) PMM1537;
1476767	-	TSS_025302	1000	167	0	3	I	within gene(s) PMM1537;
1476782	-	TSS_025305	1000	319	0	19	I	within gene(s) PMM1537;
1478505	-	TSS_025344	1000	231	0	0	I	within gene(s) PMM1540;
1478682	+	TSS_010711	1000	117	0	0	Ai	antisense to gene(s) PMM1540;
1478791	+	TSS_010712	1000	163	0	0	Ai	antisense to gene(s) PMM1540;
1479302	-	TSS_025358	1000	227	0	7	I	within gene(s) PMM1541;
1479329	-	TSS_025364	1000	135	0	13	I	within gene(s) PMM1541;
1479460	-	TSS_025373	1000	118	0	9	IP	within gene(s) PMM1542; 43nt upstream of gene PMM1541;
1479524	+	TSS_010720	1000	115	0	0	Ai	antisense to gene(s) PMM1542;
1479631	-	TSS_025387	1000	151	0	9	IP	within gene(s) PMM1542; 214nt upstream of gene PMM1541;
1479671	+	TSS_010722	1000	344	0	0	Ai	antisense to gene(s) PMM1542;
1479684	+	TSS_010723	1000	1018	0	0	Ai	antisense to gene(s) PMM1542;
1479695	+	TSS_010724	1000	225	0	1	Ai	antisense to gene(s) PMM1542;
1479743	+	TSS_010726	1000	193	0	0	Ai	antisense to gene(s) PMM1542;
1479770	+	TSS_010727	1000	102	0	0	Ai	antisense to gene(s) PMM1542;
1479809	+	TSS_010729	1000	617	0	3	Ai	antisense to gene(s) PMM1542;
1479840	+	TSS_010731	1000	149	0	0	Ai	antisense to gene(s) PMM1542;
1479903	+	TSS_010733	1000	271	0	8	Ai	antisense to gene(s) PMM1542;
1479910	-	TSS_025417	1000	141	0	6	I	within gene(s) PMM1542;
1479934	-	TSS_025421	1000	124	0	0	I	within gene(s) PMM1542;
1479946	-	TSS_025423	1000	148	0	3	I	within gene(s) PMM1542;
1479953	+	TSS_010738	1000	348	0	1	Ai	antisense to gene(s) PMM1542;
1479980	+	TSS_010739	1000	152	0	0	Ai	antisense to gene(s) PMM1542;
1480030	-	TSS_025426	1000	103	0	9	I	within gene(s) PMM1542;
1480042	-	TSS_025429	1000	137	0	3	P	0nt upstream of gene PMM1542;
1480075	-	TSS_025430	1000	101	0	0	IP	within gene(s) PMM1543; 33nt upstream of gene PMM1542;
1480129	+	TSS_010740	1000	143	0	0	Ai	antisense to gene(s) PMM1543;
1480219	-	TSS_025437	1000	213	0	6	IP	within gene(s) PMM1543; 177nt upstream of gene PMM1542;
1480234	-	TSS_025439	1000	248	0	12	IP	within gene(s) PMM1543; 192nt upstream of gene PMM1542;
1480267	-	TSS_025443	1000	132	0	24	IP	within gene(s) PMM1543; 225nt upstream of gene PMM1542;

1480372	-	TSS_025454	1000	111	0	2	I	within gene(s) PMM1543;
1480470	-	TSS_025463	1000	168	0	12	IP	within gene(s) PMM1544; 44nt upstream of gene PMM1543;
1480500	-	TSS_025469	1000	280	0	7	IP	within gene(s) PMM1544; 74nt upstream of gene PMM1543;
1480526	-	TSS_025475	1000	543	0	18	IP	within gene(s) PMM1544; 100nt upstream of gene PMM1543;
1480548	-	TSS_025479	1000	280	0	12	IP	within gene(s) PMM1544; 122nt upstream of gene PMM1543;
1480596	-	TSS_025485	1000	338	0	1	IP	within gene(s) PMM1544; 170nt upstream of gene PMM1543;
1480604	-	TSS_025487	1000	10845	0	5	IP	within gene(s) PMM1544; 178nt upstream of gene PMM1543;
1480623	-	TSS_025491	1000	129	0	12	IP	within gene(s) PMM1544; 197nt upstream of gene PMM1543;
1480632	+	TSS_010751	1000	207	0	5	Ai	antisense to gene(s) PMM1544;
1480662	-	TSS_025495	1000	354	0	3	IP	within gene(s) PMM1544; 236nt upstream of gene PMM1543;
1480779	-	TSS_025500	1000	164	0	10	I	within gene(s) PMM1544;
1480796	-	TSS_025503	1000	109	0	1	I	within gene(s) PMM1544;
1480830	-	TSS_025514	1000	300	0	46	I	within gene(s) PMM1544;
1480899	-	TSS_025530	1000	155	0	33	I	within gene(s) PMM1544;
1480929	-	TSS_025534	1000	103	0	0	I	within gene(s) PMM1544;
1480950	-	TSS_025538	1000	181	0	21	I	within gene(s) PMM1544;
1480977	-	TSS_025544	1000	122	0	1	I	within gene(s) PMM1544;
1480992	-	TSS_025546	1000	592	0	1	P	12nt upstream of gene PMM1544;
1481019	-	TSS_025548	1000	102	0	0	IP	within gene(s) PMM1545; 39nt upstream of gene PMM1544;
1481060	+	TSS_010764	1000	483	0	4	Ai	antisense to gene(s) PMM1545;
1481067	-	TSS_025550	1000	625	0	1	IP	within gene(s) PMM1545; 87nt upstream of gene PMM1544;
1481088	-	TSS_025552	1000	240	0	4	IP	within gene(s) PMM1545; 108nt upstream of gene PMM1544;
1481118	-	TSS_025556	1000	175	0	10	IP	within gene(s) PMM1545; 138nt upstream of gene PMM1544;
1481145	-	TSS_025560	1000	103	0	0	IP	within gene(s) PMM1545; 165nt upstream of gene PMM1544;
1481181	-	TSS_025563	1000	165	0	3	IP	within gene(s) PMM1545; 201nt upstream of gene PMM1544;
1481443	-	TSS_025577	1000	414	0	0	IP	within gene(s) PMM1546; 49nt upstream of gene PMM1545;
1481455	-	TSS_025580	1000	239	0	18	IP	within gene(s) PMM1546; 61nt upstream of gene PMM1545;
1481518	-	TSS_025594	1000	267	0	9	IP	within gene(s) PMM1546; 124nt upstream of gene PMM1545;
1481554	-	TSS_025603	1000	168	0	9	IP	within gene(s) PMM1546; 160nt upstream of gene PMM1545;
1481701	-	TSS_025608	1000	148	0	3	I	within gene(s) PMM1546;
1481752	-	TSS_025614	1000	378	0	0	I	within gene(s) PMM1546;
1481803	-	TSS_025615	1000	415	0	0	I	within gene(s) PMM1546;
1481827	-	TSS_025617	1000	182	0	21	I	within gene(s) PMM1546;
1481890	-	TSS_025624	1000	130	0	0	I	within gene(s) PMM1546;
1482034	-	TSS_025629	1000	477	0	1	IP	within gene(s) PMM1547; 93nt upstream of gene PMM1546;
1482391	-	TSS_025639	1000	574	0	21	IP	within gene(s) PMM1548; 18nt upstream of gene PMM1547;
1482442	-	TSS_025649	1000	131	0	15	IP	within gene(s) PMM1548; 69nt upstream of gene PMM1547;
1482512	+	TSS_010782	1000	233	0	0	Ai	antisense to gene(s) PMM1548;
1482526	+	TSS_010783	1000	397	0	1	Ai	antisense to gene(s) PMM1548;
1482634	-	TSS_025663	1000	227	0	0	I	within gene(s) PMM1548;
1482685	+	TSS_010792	1000	105	0	7	Ai	antisense to gene(s) PMM1548;
1482715	-	TSS_025677	1000	447	0	39	I	within gene(s) PMM1548;
1482753	-	TSS_025680	1000	615	0	5	IP	within gene(s) PMM1549; 14nt upstream of gene PMM1548;
1482783	-	TSS_025684	1000	138	0	12	IP	within gene(s) PMM1549; 44nt upstream of gene PMM1548;
1482804	-	TSS_025686	1000	1324	0	10	IP	within gene(s) PMM1549; 65nt upstream of gene PMM1548;
1482821	-	TSS_025691	1000	349	0	3	IP	within gene(s) PMM1549; 82nt upstream of gene PMM1548;
1482876	-	TSS_025699	1000	1430	0	27	IP	within gene(s) PMM1549; 137nt upstream of gene PMM1548;
1483028	-	TSS_025710	1000	107	0	9	IP	within gene(s) PMM1550; 26nt upstream of gene PMM1549;
1483052	-	TSS_025714	1000	177	0	14	IP	within gene(s) PMM1550; 50nt upstream of gene PMM1549;
1483243	-	TSS_025733	1000	392	0	32	IP	within gene(s) PMM1551; 14nt upstream of gene PMM1550;
1483278	+	TSS_010796	1000	118	0	1	Ai	antisense to gene(s) PMM1551;
1483391	+	TSS_010802	1000	175	0	4	Ai	antisense to gene(s) PMM1551;
1483398	+	TSS_010803	1000	272	0	1	Ai	antisense to gene(s) PMM1551;
1483415	+	TSS_010805	1000	120	0	0	Ai	antisense to gene(s) PMM1551;
1483457	-	TSS_025752	1000	113	0	4	IP	within gene(s) PMM1551; 228nt upstream of gene PMM1550;
1483479	-	TSS_025757	1000	193	0	9	IP	within gene(s) PMM1551; 250nt upstream of gene PMM1550;
1483513	-	TSS_025763	1000	168	0	18	I	within gene(s) PMM1551;
1483528	-	TSS_025768	1000	207	0	4	I	within gene(s) PMM1551;
1483591	-	TSS_025773	1000	127	0	3	I	within gene(s) PMM1551;
1483603	-	TSS_025776	1000	132	0	24	I	within gene(s) PMM1551;
1483651	-	TSS_025782	1000	110	0	0	I	within gene(s) PMM1551;
1483672	-	TSS_025786	1000	183	0	19	I	within gene(s) PMM1551;
1483720	-	TSS_025791	1000	192	0	4	P	12nt upstream of gene PMM1551;
1483737	-	TSS_025793	1000	114	0	4	IP	within gene(s) PMM1552; 29nt upstream of gene PMM1551;
1483738	+	TSS_010809	1000	260	0	0	Ai	antisense to gene(s) PMM1552;
1483752	-	TSS_025797	1000	313	0	9	IP	within gene(s) PMM1552; 44nt upstream of gene PMM1551;
1483824	-	TSS_025806	1000	106	0	9	IP	within gene(s) PMM1552; 116nt upstream of gene PMM1551;
1483845	-	TSS_025811	1000	128	0	6	IP	within gene(s) PMM1552; 137nt upstream of gene PMM1551;
1483902	-	TSS_025814	1000	162	0	0	IP	within gene(s) PMM1552; 194nt upstream of gene PMM1551;
1483955	+	TSS_010813	1000	186	0	8	Ai	antisense to gene(s) PMM1552;
1483975	+	TSS_010817	1000	2982	0	1	Ai	antisense to gene(s) PMM1552;
1483989	-	TSS_025818	1000	122	0	24	I	within gene(s) PMM1552;
1484022	+	TSS_010818	1000	109	0	0	Ai	antisense to gene(s) PMM1552;
1484058	-	TSS_025829	1000	119	0	5	I	within gene(s) PMM1552;
1484143	+	TSS_010822	1000	222	0	0	Ai	antisense to gene(s) PMM1552;
1484217	+	TSS_010825	1000	257	0	1	Ai	antisense to gene(s) PMM1552;
1484310	-	TSS_025848	1000	215	0	12	I	within gene(s) PMM1552;
1484358	-	TSS_025854	1000	106	0	18	I	within gene(s) PMM1552;
1484379	+	TSS_010828	1000	167	0	0	Ai	antisense to gene(s) PMM1552;

1484481	-	TSS_025862	1000	123	0	2 IP	within gene(s) PMM1553; 30nt upstream of gene PMM1552;
1484552	-	TSS_025866	1000	117	0	9 IP	within gene(s) PMM1553; 101nt upstream of gene PMM1552;
1484630	-	TSS_025873	1000	380	0	7 IP	within gene(s) PMM1553; 179nt upstream of gene PMM1552;
1484657	-	TSS_025877	1000	167	0	3 IP	within gene(s) PMM1553; 206nt upstream of gene PMM1552;
1484672	-	TSS_025878	1000	131	0	21 IP	within gene(s) PMM1553; 221nt upstream of gene PMM1552;
1484792	-	TSS_025893	1000	153	0	0 I	within gene(s) PMM1553;
1484951	-	TSS_025900	1000	130	0	0 IP	within gene(s) PMM1554; 114nt upstream of gene PMM1553;
1484971	-	TSS_025903	1000	961	0	10 IP	within gene(s) PMM1554; 134nt upstream of gene PMM1553;
1485026	-	TSS_025915	1000	210	0	0 IP	within gene(s) PMM1554; 189nt upstream of gene PMM1553;
1485044	-	TSS_025917	1000	112	0	6 IP	within gene(s) PMM1554; 207nt upstream of gene PMM1553;
1485191	+	TSS_010832	1000	368	0	2 Ai	antisense to gene(s) PMM1555;
1485223	+	TSS_010834	1000	332	0	0 Ai	antisense to gene(s) PMM1555;
1485343	-	TSS_025934	1000	1066	0	51 IP	within gene(s) PMM1555; 230nt upstream of gene PMM1554;
1485370	-	TSS_025941	1000	3143	0	1 I	within gene(s) PMM1555;
1485379	-	TSS_025942	1000	304	0	0 I	within gene(s) PMM1555;
1485406	-	TSS_025943	1000	102	0	0 I	within gene(s) PMM1555;
1485407	+	TSS_010838	1000	456	0	9 Ai	antisense to gene(s) PMM1555;
1485418	-	TSS_025944	1000	146	0	0 I	within gene(s) PMM1555;
1485420	+	TSS_010841	1000	115	0	0 Ai	antisense to gene(s) PMM1555;
1485445	-	TSS_025946	1000	295	0	15 I	within gene(s) PMM1555;
1485484	-	TSS_025952	1000	230	0	15 I	within gene(s) PMM1555;
1485505	-	TSS_025956	1000	358	0	21 I	within gene(s) PMM1555;
1485534	+	TSS_010843	1000	605	0	5 Ai	antisense to gene(s) PMM1555;
1485547	-	TSS_025965	1000	126	0	3 I	within gene(s) PMM1555;
1485567	+	TSS_010845	1000	300	0	1 Ai	antisense to gene(s) PMM1555;
1485568	-	TSS_025967	1000	115	0	0 I	within gene(s) PMM1555;
1485649	-	TSS_025975	1000	282	0	24 I	within gene(s) PMM1555;
1485667	-	TSS_025978	1000	273	0	9 I	within gene(s) PMM1555;
1485685	-	TSS_025981	1000	156	0	0 I	within gene(s) PMM1555;
1485699	-	TSS_025982	1000	108	0	10 I	within gene(s) PMM1555;
1485727	-	TSS_025988	1000	123	0	4 I	within gene(s) PMM1555;
1485748	+	TSS_010847	1000	221	0	0 Ai	antisense to gene(s) PMM1555;
1485766	-	TSS_025994	1000	136	0	39 I	within gene(s) PMM1555;
1485826	-	TSS_026002	1000	101	0	0 I	within gene(s) PMM1555;
1485844	+	TSS_010851	1000	199	0	7 Ai	antisense to gene(s) PMM1555;
1485868	-	TSS_026003	1000	122	0	12 I	within gene(s) PMM1555;
1485958	-	TSS_026007	1000	121	0	6 I	within gene(s) PMM1555;
1485992	+	TSS_010852	1000	108	0	0 Ai	antisense to gene(s) PMM1555;
1486047	+	TSS_010854	1000	168	0	1 Ai	antisense to gene(s) PMM1556;
1486051	-	TSS_026020	1000	169	0	3 IP	within gene(s) PMM1556; 30nt upstream of gene PMM1555;
1486078	-	TSS_026026	1000	216	0	15 IP	within gene(s) PMM1556; 57nt upstream of gene PMM1555;
1486129	-	TSS_026030	1000	494	0	1 IP	within gene(s) PMM1556; 108nt upstream of gene PMM1555;
1486161	-	TSS_026033	1000	544	0	11 IP	within gene(s) PMM1556; 140nt upstream of gene PMM1555;
1486264	-	TSS_026041	1000	137	0	6 IP	within gene(s) PMM1556; 243nt upstream of gene PMM1555;
1486353	-	TSS_026048	1000	119	0	23 IP	within gene(s) PMM1557; 17nt upstream of gene PMM1556;
1486430	-	TSS_026056	1000	553	0	1 IP	within gene(s) PMM1557; 94nt upstream of gene PMM1556;
1486752	-	TSS_026070	1000	104	0	10 I	within gene(s) PMM1557;
1486769	+	TSS_010860	1000	110	0	1 Ai	antisense to gene(s) PMM1557;
1486832	-	TSS_026082	1000	114	0	0 I	within gene(s) PMM1557;
1486899	-	TSS_026089	1000	710	0	21 I	within gene(s) PMM1557;
1486929	-	TSS_026093	1000	152	0	9 I	within gene(s) PMM1557;
1486979	-	TSS_026102	1000	247	0	14 IP	within gene(s) PMM1558; 14nt upstream of gene PMM1557;
1487004	+	TSS_010866	1000	179	0	3 Ai	antisense to gene(s) PMM1558;
1487021	+	TSS_010867	1000	177	0	0 Ai	antisense to gene(s) PMM1558;
1487036	-	TSS_026108	1000	249	0	1 IP	within gene(s) PMM1558; 71nt upstream of gene PMM1557;
1487133	+	TSS_010868	1000	103	0	0 Ai	antisense to gene(s) PMM1558;
1487189	-	TSS_026118	1000	118	0	9 IP	within gene(s) PMM1558; 224nt upstream of gene PMM1557;
1487222	-	TSS_026121	1000	716	0	26 I	within gene(s) PMM1558;
1487273	-	TSS_026130	1000	156	0	0 I	within gene(s) PMM1558;
1487282	-	TSS_026132	1000	179	0	1 I	within gene(s) PMM1558;
1487291	-	TSS_026133	1000	162	0	0 I	within gene(s) PMM1558;
1487366	-	TSS_026138	1000	151	0	2 I	within gene(s) PMM1558;
1487895	+	TSS_010874	1000	427	0	6 P	13nt upstream of gene PMM1559;
1489480	+	TSS_010886	1000	242	0	6 P	92nt upstream of gene PMM1561;
1490373	+	TSS_010891	1000	3164	0	1 P	85nt upstream of gene PMM1562;
1490482	+	TSS_010893	1000	170	0	3 I	within gene(s) PMM1562;
1490563	+	TSS_010901	1000	157	0	12 I	within gene(s) PMM1562;
1490578	+	TSS_010907	1000	387	0	12 I	within gene(s) PMM1562;
1490650	+	TSS_010917	1000	745	0	15 I	within gene(s) PMM1562;
1490668	+	TSS_010919	1000	142	0	9 I	within gene(s) PMM1562;
1490695	+	TSS_010926	1000	200	0	6 I	within gene(s) PMM1562;
1490734	+	TSS_010927	1000	103	0	0 I	within gene(s) PMM1562;
1490764	+	TSS_010933	1000	237	0	5 I	within gene(s) PMM1562;
1490788	-	TSS_026153	1000	148	0	2 Ai	antisense to gene(s) PMM1562;
1490794	+	TSS_010937	1000	504	0	30 I	within gene(s) PMM1562;
1490827	+	TSS_010944	1000	108	0	6 I	within gene(s) PMM1562;
1490845	+	TSS_010946	1000	189	0	5 I	within gene(s) PMM1562;
1490881	+	TSS_010951	1000	467	0	30 I	within gene(s) PMM1562;
1490956	-	TSS_026156	1000	122	0	3 Ai	antisense to gene(s) PMM1562;

1491011	-	TSS_026159	1000	154	0	0	Ai	antisense to gene(s) PMM1562;
1491067	+	TSS_010969	1000	416	0	6	I	within gene(s) PMM1562;
1491070	-	TSS_026160	1000	125	0	1	Ai	antisense to gene(s) PMM1562;
1491092	+	TSS_010970	1000	116	0	0	I	within gene(s) PMM1562;
1491099	-	TSS_026162	1000	106	0	0	Ai	antisense to gene(s) PMM1562;
1491112	+	TSS_010972	1000	183	0	6	I	within gene(s) PMM1562;
1491124	+	TSS_010974	1000	125	0	6	I	within gene(s) PMM1562;
1491478	+	TSS_011008	1000	254	0	36	I	within gene(s) PMM1562;
1491629	-	TSS_026167	1000	109	0	0	I	within gene(s) PMM1563;
1491832	-	TSS_026170	1000	171	0	2	P	14nt upstream of gene PMM1563;
1494281	+	TSS_011020	1000	254	0	0	Ai	antisense to gene(s) PMM1566;
1495329	+	TSS_011024	1000	1412	0	5	Ai	antisense to gene(s) PMM1566;
1495758	-	TSS_026182	1000	943	0	2	PAi	19nt upstream of gene PMM1566; antisense to gene(s) PMM1567;
1495801	-	TSS_026184	1000	197	0	0	PAi	62nt upstream of gene PMM1566; antisense to gene(s) PMM1567;
1496620	+	TSS_011028	1000	659	0	0	IAd	within gene(s) PMM1567; antisense to gene(s) PMM1568 (11nt downstream);
1496904	-	TSS_026194	1000	132	0	0	I	within gene(s) PMM1568;
1497016	-	TSS_026197	1000	7109	0	10	P	21nt upstream of gene PMM1568;
1499500	-	TSS_026211	1000	207	0	2	I	within gene(s) PMM1570;
1499641	-	TSS_026213	1000	215	0	0	P	35nt upstream of gene PMM1570;
1499646	+	TSS_011046	1000	219	0	11	P	71nt upstream of gene PMM1571;
1499680	-	TSS_026214	1000	139	0	0	P	74nt upstream of gene PMM1570;
1499710	+	TSS_011051	1000	194	0	0	P	7nt upstream of gene PMM1571;
1501198	+	TSS_011054	1000	122	0	0	Ai	antisense to gene(s) PMM1573;
1502320	-	TSS_026218	1000	132	0	0	I	within gene(s) PMM1574;
1503407	+	TSS_011059	1000	150	0	1	Ai	antisense to gene(s) PMM1575;
1504039	-	TSS_026250	1000	134	0	9	I	within gene(s) PMM1575;
1504888	-	TSS_026260	1000	119	0	0	I	within gene(s) PMM1575;
1504964	-	TSS_026261	1000	161	0	0	I	within gene(s) PMM1575;
1505706	-	TSS_026268	1000	339	0	0	I	within gene(s) PMM1575;
1508411	-	TSS_026278	1000	315	0	1	P	11nt upstream of gene PMM1577;
1508562	+	TSS_011073	1000	373	0	1	Ai	antisense to gene(s) PMM1578;
1508766	+	TSS_011074	1000	118	0	0	Ai	antisense to gene(s) PMM1578;
1508792	-	TSS_026290	1000	275	0	4	I	within gene(s) PMM1578;
1508833	-	TSS_026295	1000	403	0	11	I	within gene(s) PMM1578;
1508855	-	TSS_026302	1000	362	0	10	I	within gene(s) PMM1578;
1508882	-	TSS_026307	1000	115	0	3	P	0nt upstream of gene PMM1578;
1510763	-	TSS_026312	1000	109	0	0	I	within gene(s) PMM1580;
1511027	+	TSS_011079	1000	679	0	0	Ai	antisense to gene(s) PMM1580;
1512795	-	TSS_026320	1000	319	0	0	P	92nt upstream of gene PMM1581;
1513707	-	TSS_026324	1000	395	0	0	Ai	antisense to gene(s) PMM1582;
1513724	-	TSS_026325	1000	396	0	0	Ai	antisense to gene(s) PMM1582;
1514262	-	TSS_026327	1000	115	0	0	I	within gene(s) PMM1583;
1515544	-	TSS_026331	1000	617	0	1	I	within gene(s) PMM1585;
1518815	-	TSS_026347	1000	169	0	3	I	within gene(s) PMM1589;
1519244	-	TSS_026356	1000	735	0	1	P	24nt upstream of gene PMM1589;
1523570	-	TSS_026370	1000	1076	0	0	P	23nt upstream of gene PMM1594;
1524215	+	TSS_011115	1000	176	0	2	P	40nt upstream of gene PMM1596;
1525299	+	TSS_011129	1000	156	0	9	I	within gene(s) PMM1596;
1528833	-	TSS_026398	1000	187	0	2	P	16nt upstream of gene PMM1599;
1529156	-	TSS_026405	1000	178	0	3	I	within gene(s) PMM1600;
1529429	-	TSS_026418	1000	196	0	6	I	within gene(s) PMM1600;
1529501	-	TSS_026421	1000	314	0	0	I	within gene(s) PMM1600;
1529522	-	TSS_026422	1000	137	0	0	I	within gene(s) PMM1600;
1529573	-	TSS_026423	1000	217	0	6	I	within gene(s) PMM1600;
1530109	+	TSS_011152	1000	338	0	0	Ai	antisense to gene(s) PMM1600;
1530254	-	TSS_026444	1000	3740	0	2	P	27nt upstream of gene PMM1600;
1530284	-	TSS_026449	1000	228	0	1	P	57nt upstream of gene PMM1600;
1530378	+	TSS_011157	1000	10424	0	3	P	3nt upstream of gene PMM1601;
1530519	+	TSS_011161	1000	171	0	3	I	within gene(s) PMM1601;
1531203	-	TSS_026453	1000	104	0	1	Ai	antisense to gene(s) PMM1601;
1531400	-	TSS_026454	1000	280	0	0	Ai	antisense to gene(s) PMM1601;
1533693	-	TSS_026461	1000	141	0	3	I	within gene(s) PMM1602;
1533752	-	TSS_026464	1000	11649	0	8	P	14nt upstream of gene PMM1602;
1533794	-	TSS_026467	1000	1096	0	0	P	56nt upstream of gene PMM1602;
1534044	-	TSS_026470	1000	170	0	0	O	-
1534092	-	TSS_026472	1000	440	0	1	O	-
1534100	-	TSS_026474	1000	151	0	1	O	-
1534301	-	TSS_026480	1000	243	0	2	O	-
1535304	+	TSS_011236	1000	107	0	7	I	within gene(s) PMM1605;
1535492	-	TSS_026484	1000	139	0	0	PAi	224nt upstream of gene PMM1604; antisense to gene(s) PMM1605;
1537533	-	TSS_026497	1000	508	0	0	I	within gene(s) PMM1606;
1537744	+	TSS_011242	1000	372	0	0	Ai	antisense to gene(s) PMM1606;
1537968	-	TSS_026505	1000	125	0	3	IP	within gene(s) PMM1607; 222nt upstream of gene PMM1606;
1538040	-	TSS_026513	1000	126	0	7	I	within gene(s) PMM1607;
1538095	-	TSS_026519	1000	19583	0	13	P	22nt upstream of gene PMM1607;
1538226	+	TSS_011250	1000	168	0	0	IP	within gene(s) PMM1608; 231nt upstream of gene PMM1609;
1538236	+	TSS_011251	1000	117	0	0	IP	within gene(s) PMM1608; 221nt upstream of gene PMM1609;
1538256	+	TSS_011253	1000	129	0	0	IP	within gene(s) PMM1608; 201nt upstream of gene PMM1609;
1538307	+	TSS_011256	1000	184	0	0	IP	within gene(s) PMM1608; 150nt upstream of gene PMM1609;

1539467	+	TSS_011273	1000	2607	0	2	I	within gene(s) PMM1609;
1539568	+	TSS_011276	1000	622	0	2	IP	within gene(s) PMM1609; 176nt upstream of gene PMM1610;
1539778	-	TSS_026533	1000	1023	0	4	Ai	antisense to gene(s) PMM1610;
1539903	+	TSS_011283	1000	141	0	3	I	within gene(s) PMM1610;
1539930	+	TSS_011287	1000	406	0	9	I	within gene(s) PMM1610;
1539957	+	TSS_011289	1000	121	0	9	I	within gene(s) PMM1610;
1540053	+	TSS_011295	1000	162	0	6	I	within gene(s) PMM1610;
1540104	+	TSS_011300	1000	108	0	3	I	within gene(s) PMM1610;
1540526	-	TSS_026542	1000	124	0	1	Ai	antisense to gene(s) PMM1610;
1540533	+	TSS_011328	1000	238	0	9	I	within gene(s) PMM1610;
1540560	+	TSS_011330	1000	104	0	1	I	within gene(s) PMM1610;
1540599	+	TSS_011333	1000	230	0	4	I	within gene(s) PMM1610;
1540767	+	TSS_011337	1000	101	0	12	I	within gene(s) PMM1610;
1541079	+	TSS_011355	1000	142	0	0	I	within gene(s) PMM1610;
1541091	+	TSS_011356	1000	337	0	0	I	within gene(s) PMM1610;
1541109	+	TSS_011358	1000	248	0	2	I	within gene(s) PMM1610;
1541313	+	TSS_011365	1000	102	0	8	I	within gene(s) PMM1610;
1542021	+	TSS_011383	1000	167	0	3	Ai	antisense to gene(s) PMM1611;
1542045	-	TSS_026564	1000	222	0	12	I	within gene(s) PMM1611;
1542189	-	TSS_026576	1000	128	0	3	I	within gene(s) PMM1611;
1542336	-	TSS_026587	1000	185	0	9	I	within gene(s) PMM1611;
1542447	-	TSS_026596	1000	144	0	3	I	within gene(s) PMM1611;
1542801	-	TSS_026616	1000	170	0	0	I	within gene(s) PMM1611;
1542926	-	TSS_026625	1000	135	0	27	I	within gene(s) PMM1611;
1543254	-	TSS_026636	1000	2749	0	3	P	126nt upstream of gene PMM1611;
1543584	+	TSS_011393	1000	316	0	0	Ai	antisense to gene(s) PMM1613;
1543786	+	TSS_011394	1000	244	0	0	Ai	antisense to gene(s) PMM1613;
1546552	-	TSS_026650	1000	383	0	0	I	within gene(s) PMM1615;
1547526	+	TSS_011413	1000	139	0	0	Ai	antisense to gene(s) PMM1618;
1549170	-	TSS_026663	1000	120	0	6	IP	within gene(s) PMM1619; 227nt upstream of gene PMM1618;
1549210	-	TSS_026667	1000	131	0	0	I	within gene(s) PMM1619;
1549218	+	TSS_011421	1000	110	0	1	Ai	antisense to gene(s) PMM1619;
1549235	-	TSS_026668	1000	902	0	0	I	within gene(s) PMM1619;
1549500	-	TSS_026686	1000	172	0	12	I	within gene(s) PMM1619;
1549539	-	TSS_026688	1000	104	0	1	I	within gene(s) PMM1619;
1551244	+	TSS_011424	1000	138	0	2	Ai	antisense to gene(s) PMM1622;
1551407	-	TSS_026698	1000	130	0	1	IP	within gene(s) PMM1622; 205nt upstream of gene PMM1621;
1551449	-	TSS_026700	1000	132	0	0	IP	within gene(s) PMM1622; 247nt upstream of gene PMM1621;
1552273	-	TSS_026725	1000	2355	0	4	P	14nt upstream of gene PMM1622;
1552380	+	TSS_011431	1000	293	0	1	P	12nt upstream of gene PMM1623;
1552921	+	TSS_011440	1000	216	0	0	Ai	antisense to gene(s) PMM1624;
1553709	-	TSS_026736	1000	115	0	12	I	within gene(s) PMM1625;
1553802	-	TSS_026741	1000	168	0	3	I	within gene(s) PMM1625;
1553970	-	TSS_026754	1000	212	0	21	I	within gene(s) PMM1625;
1553991	-	TSS_026757	1000	312	0	9	I	within gene(s) PMM1625;
1554018	-	TSS_026764	1000	224	0	12	I	within gene(s) PMM1625;
1554027	-	TSS_026765	1000	200	0	12	I	within gene(s) PMM1625;
1554048	-	TSS_026770	1000	288	0	3	I	within gene(s) PMM1625;
1554065	+	TSS_011451	1000	493	0	4	Ai	antisense to gene(s) PMM1625;
1554069	-	TSS_026775	1000	116	0	7	I	within gene(s) PMM1625;
1554078	-	TSS_026776	1000	104	0	0	I	within gene(s) PMM1625;
1554099	-	TSS_026778	1000	171	0	6	I	within gene(s) PMM1625;
1554138	-	TSS_026788	1000	214	0	27	I	within gene(s) PMM1625;
1554156	-	TSS_026792	1000	155	0	12	I	within gene(s) PMM1625;
1554168	-	TSS_026794	1000	110	0	3	I	within gene(s) PMM1625;
1554183	-	TSS_026797	1000	340	0	72	I	within gene(s) PMM1625;
1554300	-	TSS_026818	1000	222	0	15	I	within gene(s) PMM1625;
1554387	-	TSS_026826	1000	101	0	6	I	within gene(s) PMM1625;
1554435	-	TSS_026830	1000	169	0	12	I	within gene(s) PMM1625;
1554450	-	TSS_026832	1000	228	0	3	I	within gene(s) PMM1625;
1554465	-	TSS_026836	1000	650	0	4	I	within gene(s) PMM1625;
1554486	-	TSS_026838	1000	143	0	2	I	within gene(s) PMM1625;
1554498	-	TSS_026841	1000	1080	0	15	I	within gene(s) PMM1625;
1554519	-	TSS_026847	1000	179	0	0	I	within gene(s) PMM1625;
1554538	+	TSS_011461	1000	121	0	0	Ai	antisense to gene(s) PMM1625;
1554543	-	TSS_026849	1000	522	0	28	I	within gene(s) PMM1625;
1554588	-	TSS_026859	1000	104	6.80E-13	3	I	within gene(s) PMM1625;
1554603	-	TSS_026862	1000	225	0	30	I	within gene(s) PMM1625;
1554651	-	TSS_026870	1000	119	0	0	I	within gene(s) PMM1625;
1554666	-	TSS_026871	1000	242	0	0	I	within gene(s) PMM1625;
1554678	-	TSS_026873	1000	129	0	6	I	within gene(s) PMM1625;
1554690	-	TSS_026875	1000	153	0	0	I	within gene(s) PMM1625;
1554708	-	TSS_026879	1000	479	0	32	I	within gene(s) PMM1625;
1554741	-	TSS_026888	1000	142	0	6	I	within gene(s) PMM1625;
1554856	-	TSS_026896	1000	5625	0	4	P	16nt upstream of gene PMM1625;
1554868	+	TSS_011463	1000	102	0	0	P	22nt upstream of gene PMM1626;
1557422	+	TSS_011476	1000	147	0	0	Ai	antisense to gene(s) PMM1629;
1557432	+	TSS_011478	1000	843	0	1	Ai	antisense to gene(s) PMM1629;
1557493	-	TSS_026913	1000	216	0	10	I	within gene(s) PMM1629;

1557508	-	TSS_026915	1000	131	0	0	I	within gene(s) PMM1629;
1557604	-	TSS_026927	1000	243	0	6	I	within gene(s) PMM1629;
1557658	-	TSS_026931	1000	160	0	0	I	within gene(s) PMM1629;
1557709	+	TSS_011482	1000	146	0	0	Ai	antisense to gene(s) PMM1629;
1557709	-	TSS_026934	1000	391	0	3	I	within gene(s) PMM1629;
1557730	-	TSS_026939	1000	145	0	24	I	within gene(s) PMM1629;
1557781	-	TSS_026948	1000	221	0	3	I	within gene(s) PMM1629;
1557793	-	TSS_026950	1000	117	0	0	I	within gene(s) PMM1629;
1557838	-	TSS_026954	1000	157	0	10	I	within gene(s) PMM1629;
1557985	-	TSS_026965	1000	263	0	9	I	within gene(s) PMM1629;
1558039	-	TSS_026970	1000	265	0	13	I	within gene(s) PMM1629;
1558072	-	TSS_026976	1000	250	0	7	I	within gene(s) PMM1629;
1558291	-	TSS_026980	1000	580	0	2	P	18nt upstream of gene PMM1629;
1558425	+	TSS_011487	1000	251	0	0	Ai	antisense to gene(s) PMM1630;
1558456	+	TSS_011488	1000	190	0	0	Ai	antisense to gene(s) PMM1630;
1558709	-	TSS_026987	1000	126	0	1	I	within gene(s) PMM1630;
1559126	-	TSS_027006	1000	292	0	1	I	within gene(s) PMM1630;
1559240	+	TSS_011496	1000	1185	0	4	Ai	antisense to gene(s) PMM1630;
1559402	-	TSS_027016	1000	157	0	4	I	within gene(s) PMM1630;
1559495	-	TSS_027025	1000	105	0	9	I	within gene(s) PMM1630;
1559513	-	TSS_027027	1000	111	0	0	I	within gene(s) PMM1630;
1559546	-	TSS_027029	1000	110	0	0	I	within gene(s) PMM1630;
1559756	-	TSS_027037	1000	6258	0	6	I	within gene(s) PMM1630;
1559776	-	TSS_027042	1000	207	0	4	I	within gene(s) PMM1630;
1559806	-	TSS_027046	1000	2345	0	3	P	23nt upstream of gene PMM1630;
1561053	-	TSS_027050	1000	281	0	6	IP	within gene(s) PMM1634; 197nt upstream of gene PMM1633;
1561061	-	TSS_027052	1000	7012	0	3	IP	within gene(s) PMM1634; 205nt upstream of gene PMM1633;
1561614	+	TSS_011506	1000	256	0	1	Ai	antisense to gene(s) PMM1634;
1561620	-	TSS_027081	1000	106	0	12	I	within gene(s) PMM1634;
1561701	+	TSS_011510	1000	292	0	2	Ai	antisense to gene(s) PMM1634;
1562007	-	TSS_027099	1000	105	0	3	I	within gene(s) PMM1634;
1562815	-	TSS_027128	1000	257	0	0	I	within gene(s) PMM1634;
1562872	-	TSS_027129	1000	772	0	2	P	52nt upstream of gene PMM1634;
1563062	-	TSS_027133	1000	108	0	2	PAi	242nt upstream of gene PMM1634; antisense to gene(s) PMM1635;
1563883	+	TSS_011523	1000	155	0	5	P	41nt upstream of gene PMM1636;
1563968	-	TSS_027136	1000	282	0	0	Ai	antisense to gene(s) PMM1636;
1564062	+	TSS_011526	1000	686	0	0	I	within gene(s) PMM1636;
1564123	-	TSS_027137	1000	623	0	0	Ai	antisense to gene(s) PMM1636;
1565022	-	TSS_027140	1000	153	0	0	Ai	antisense to gene(s) PMM1637;
1565055	-	TSS_027141	1000	199	0	0	Ai	antisense to gene(s) PMM1637;
1566190	-	TSS_027147	1000	177	0	0	Ai	antisense to gene(s) PMM1638;
1566861	+	TSS_011541	1000	209	0	0	Ai	antisense to gene(s) PMM1639;
1566981	+	TSS_011544	1000	107	0	0	Ai	antisense to gene(s) PMM1639;
1567157	+	TSS_011549	1000	206	0	7	Ai	antisense to gene(s) PMM1639;
1567175	-	TSS_027167	1000	173	0	12	I	within gene(s) PMM1639;
1567265	-	TSS_027180	1000	175	0	3	I	within gene(s) PMM1639;
1567784	-	TSS_027201	1000	117	0	9	I	within gene(s) PMM1639;
1567848	+	TSS_011556	1000	121	0	1	Ai	antisense to gene(s) PMM1639;
1567886	-	TSS_027211	1000	111	0	6	I	within gene(s) PMM1639;
1568020	-	TSS_027226	1000	200	0	24	I	within gene(s) PMM1639;
1568329	+	TSS_011558	1000	207	0	0	Ai	antisense to gene(s) PMM1639;
1569177	-	TSS_027291	1000	282	0	0	P	31nt upstream of gene PMM1639;
1569383	+	TSS_011566	1000	135	0	1	I	within gene(s) PMM1640;
1570531	-	TSS_027297	1000	2311	0	1	I	within gene(s) PMM1642;
1570615	-	TSS_027299	1000	317	0	0	I	within gene(s) PMM1642;
1570654	-	TSS_027300	1000	1679	0	4	P	17nt upstream of gene PMM1642;
1571379	-	TSS_027313	1000	117	0	0	P	16nt upstream of gene PMM1643;
1571555	-	TSS_027317	1000	121	0	4	IP	within gene(s) PMM1644; 192nt upstream of gene PMM1643;
1571567	-	TSS_027318	1000	124	0	0	IP	within gene(s) PMM1644; 204nt upstream of gene PMM1643;
1571578	-	TSS_027319	1000	128	0	0	IP	within gene(s) PMM1644; 215nt upstream of gene PMM1643;
1571608	-	TSS_027320	1000	168	0	0	IP	within gene(s) PMM1644; 245nt upstream of gene PMM1643;
1571739	+	TSS_011572	1000	175	0	0	P	14nt upstream of gene PMM1645;
1571777	+	TSS_011574	1000	254	0	1	I	within gene(s) PMM1645;
1576258	+	TSS_011583	1000	460	0	0	I	within gene(s) PMM1648;
1576861	+	TSS_011592	1000	562	0	2	I	within gene(s) PMM1648;
1578502	-	TSS_027340	1000	185	0	0	I	within gene(s) PMM1649;
1578931	-	TSS_027350	1000	136	0	6	I	within gene(s) PMM1649;
1580087	-	TSS_027370	1000	205	0	3	P	22nt upstream of gene PMM1649;
1580706	-	TSS_027382	1000	152	0	0	I	within gene(s) PMM1650;
1580887	-	TSS_027387	1000	1167	0	3	I	within gene(s) PMM1650;
1582153	+	TSS_011617	1000	110	0	0	Ai	antisense to gene(s) PMM1652;
1582233	-	TSS_027392	1000	314	0	18	I	within gene(s) PMM1652;
1582254	-	TSS_027397	1000	127	0	3	I	within gene(s) PMM1652;
1582484	+	TSS_011620	1000	195	0	0	Ai	antisense to gene(s) PMM1652;
1582766	-	TSS_027426	1000	243	0	1	I	within gene(s) PMM1652;
1582966	+	TSS_011625	1000	121	0	0	Ai	antisense to gene(s) PMM1652;
1583497	-	TSS_027460	1000	273	0	0	I	within gene(s) PMM1652;
1584409	-	TSS_027474	1000	3917	0	7	I	within gene(s) PMM1653;
1584725	+	TSS_011638	1000	144	0	1	Ai	antisense to gene(s) PMM1653;

1585023	-	TSS_027481	1000	1286	0	1 IP	within gene(s) PMM1654; 71nt upstream of gene PMM1653;
1586076	+	TSS_011647	1000	136	0	8 P	32nt upstream of gene PMM1655;
1587579	+	TSS_011659	1000	344	0	2 P	16nt upstream of gene PMM1656;
1587964	+	TSS_011671	1000	138	0	3 I	within gene(s) PMM1656;
1588301	+	TSS_011677	1000	1485	0	4 P	45nt upstream of gene PMM1657;
1588838	+	TSS_011689	1000	116	0	18 I	within gene(s) PMM1657;
1588914	+	TSS_011703	1000	150	0	24 I	within gene(s) PMM1657;
1588997	+	TSS_011708	1000	106	0	3 I	within gene(s) PMM1657;
1589048	+	TSS_011717	1000	685	0	12 I	within gene(s) PMM1657;
1590631	-	TSS_027496	1000	106	0	0 Ai	antisense to gene(s) PMM1658;
1594453	+	TSS_011747	1000	1115	0	4 P	19nt upstream of gene PMM1661;
1594701	+	TSS_011749	1000	525	0	0 I	within gene(s) PMM1662;
1596579	+	TSS_011758	1000	1420	0	2 P	30nt upstream of gene PMM1665;
1596903	+	TSS_011764	1000	118	0	18 I	within gene(s) PMM1665;
1597137	+	TSS_011782	1000	151	0	11 I	within gene(s) PMM1665;
1597361	-	TSS_027508	1000	106	0	0 Ai	antisense to gene(s) PMM1665;
1597536	+	TSS_011795	1000	142	0	7 I	within gene(s) PMM1665;
1597545	+	TSS_011797	1000	111	0	0 I	within gene(s) PMM1665;
1599261	-	TSS_027521	1000	424	0	0 P	34nt upstream of gene PMM1667;
1599320	-	TSS_027522	1000	292	0	0 P	93nt upstream of gene PMM1667;
1600936	+	TSS_011821	1000	355	0	1 Ai	antisense to gene(s) PMM1668;
1604240	-	TSS_027585	1000	323	0	0 IP	within gene(s) PMM1671; 236nt upstream of gene PMM1670;
1604740	-	TSS_027587	1000	461	0	2 P	24nt upstream of gene PMM1671;
1604757	+	TSS_011829	1000	346	0	12 I	within gene(s) PMM1672;
1604952	+	TSS_011837	1000	180	0	1 I	within gene(s) PMM1672;
1604976	+	TSS_011840	1000	128	0	9 I	within gene(s) PMM1672;
1605372	+	TSS_011854	1000	112	0	0 I	within gene(s) PMM1672;
1605445	+	TSS_011858	1000	158	0	4 I	within gene(s) PMM1672;
1605722	-	TSS_027591	1000	104	0	0 Ai	antisense to gene(s) PMM1672;
1605872	+	TSS_011868	1000	160	0	21 I	within gene(s) PMM1673;
1606251	+	TSS_011882	1000	161	0	2 P	15nt upstream of gene PMM1674;
1606665	+	TSS_011884	1000	124	0	0 I	within gene(s) PMM1674;
1607611	+	TSS_011889	1000	788	0	2 IP	within gene(s) PMM1674; 49nt upstream of gene PMM1675;
1607768	-	TSS_027596	1000	352	0	0 Ai	antisense to gene(s) PMM1675;
1609627	+	TSS_011898	1000	413	0	2 P	4nt upstream of gene PMM1676;
1611481	+	TSS_011901	1000	1938	0	1 P	15nt upstream of gene PMM1678;
1611842	+	TSS_011913	1000	104	0	1 IP	within gene(s) PMM1678; 91nt upstream of gene PMM1679;
1612164	+	TSS_011915	1000	113	0	0 I	within gene(s) PMM1679;
1616290	+	TSS_011926	1000	381	0	0 Ai	antisense to gene(s) PMM1682;
1616930	-	TSS_027649	1000	564	0	6 P	26nt upstream of gene PMM1682;
1617422	-	TSS_027656	1000	462	0	9 P	93nt upstream of gene PMM1683;
1618289	+	TSS_011932	1000	150	0	6 IP	within gene(s) PMM1684; 78nt upstream of gene PMM1685;
1618516	+	TSS_011934	1000	209	0	0 I	within gene(s) PMM1685;
1618595	+	TSS_011935	1000	159	0	0 I	within gene(s) PMM1685;
1619698	-	TSS_027661	1000	165	0	0 Ai	antisense to gene(s) PMM1686;
1619840	-	TSS_027662	1000	150	0	0 Ai	antisense to gene(s) PMM1686;
1620131	+	TSS_011939	1000	269	0	4 P	24nt upstream of gene PMM1687;
1621301	+	TSS_011944	1000	359	0	4 P	25nt upstream of gene PMM1688;
1622476	-	TSS_027665	1000	1385	0	6 Ai	antisense to gene(s) PMM1688;
1623168	+	TSS_011958	1000	1839	0	7 P	29nt upstream of gene PMM1689;
1623293	+	TSS_011964	1000	272	0	2 I	within gene(s) PMM1689;
1624375	+	TSS_011977	1000	117	0	0 I	within gene(s) PMM1689;
1625074	-	TSS_027674	1000	199	0	0 Ai	antisense to gene(s) PMM1690;
1628185	-	TSS_027678	1000	196	0	0 Ai	antisense to gene(s) PMM1693;
1628980	+	TSS_011987	1000	277	0	2 PAi	173nt upstream of gene PMM1695; antisense to gene(s) PMM1694;
1629107	-	TSS_027684	1000	165	0	0 P	0nt upstream of gene PMM1694;
1632609	+	TSS_011994	1000	105	0	0 P	98nt upstream of gene PMM1697;
1632963	-	TSS_027689	1000	109	0	0 Ai	antisense to gene(s) PMM1697;
1633040	+	TSS_012004	1000	140	0	0 I	within gene(s) PMM1697;
1633252	-	TSS_027691	1000	217	0	0 Ai	antisense to gene(s) PMM1697;
1633316	+	TSS_012015	1000	177	0	3 I	within gene(s) PMM1697;
1633425	-	TSS_027695	1000	159	0	1 Ai	antisense to gene(s) PMM1697;
1634577	+	TSS_012033	1000	157	0	0 Ai	antisense to gene(s) PMM1699;
1634772	+	TSS_012034	1000	172	0	0 Ai	antisense to gene(s) PMM1699;
1635344	+	TSS_012035	1000	134	0	2 PAi	199nt upstream of gene PMM1700; antisense to gene(s) PMM1699;
1635526	+	TSS_012039	1000	3838	0	7 P	17nt upstream of gene PMM1700;
1636725	+	TSS_012087	1000	106	0	3 I	within gene(s) PMM1700;
1640380	-	TSS_027718	1000	202	0	0 P	32nt upstream of gene PMM1702;
1640445	+	TSS_012121	1000	669	0	0 I	within gene(s) PMM1703;
1641448	+	TSS_012125	1000	447	0	0 IAd	within gene(s) PMM1703; antisense to gene(s) PMM1704 (29nt downstream);
1641583	-	TSS_027724	1000	208	0	12 I	within gene(s) PMM1704;
1641604	-	TSS_027727	1000	165	0	0 I	within gene(s) PMM1704;
1641616	-	TSS_027728	1000	112	0	3 I	within gene(s) PMM1704;
1641634	-	TSS_027732	1000	190	0	6 I	within gene(s) PMM1704;
1641652	-	TSS_027734	1000	131	0	0 I	within gene(s) PMM1704;
1641673	-	TSS_027735	1000	163	0	6 I	within gene(s) PMM1704;
1641692	-	TSS_027739	1000	180	0	4 I	within gene(s) PMM1704;
1641724	-	TSS_027751	1000	894	0	48 I	within gene(s) PMM1704;
1641808	-	TSS_027760	1000	209	0	2 I	within gene(s) PMM1704;

1641841	-	TSS_027764	1000	182	0	24 I	within gene(s) PMM1704;
1641892	-	TSS_027771	1000	116	0	3 I	within gene(s) PMM1704;
1641901	-	TSS_027772	1000	133	0	0 I	within gene(s) PMM1704;
1641937	-	TSS_027780	1000	587	0	43 I	within gene(s) PMM1704;
1641979	-	TSS_027792	1000	119	0	9 I	within gene(s) PMM1704;
1642006	-	TSS_027796	1000	348	0	21 I	within gene(s) PMM1704;
1642036	-	TSS_027801	1000	122	0	0 I	within gene(s) PMM1704;
1642050	+	TSS_012131	1000	404	0	0 Ai	antisense to gene(s) PMM1704;
1642057	-	TSS_027804	1000	151	0	3 I	within gene(s) PMM1704;
1642069	-	TSS_027805	1000	272	0	6 I	within gene(s) PMM1704;
1642093	-	TSS_027810	1000	116	0	3 I	within gene(s) PMM1704;
1642100	+	TSS_012133	1000	104	0	0 Ai	antisense to gene(s) PMM1704;
1642108	-	TSS_027811	1000	128	0	6 I	within gene(s) PMM1704;
1642126	-	TSS_027813	1000	106	0	0 I	within gene(s) PMM1704;
1642135	-	TSS_027815	1000	113	0	2 I	within gene(s) PMM1704;
1642147	-	TSS_027816	1000	176	0	0 I	within gene(s) PMM1704;
1642183	-	TSS_027817	1000	107	0	0 I	within gene(s) PMM1704;
1642195	-	TSS_027818	1000	106	0	2 I	within gene(s) PMM1704;
1642222	-	TSS_027826	1000	310	0	24 I	within gene(s) PMM1704;
1642229	+	TSS_012134	1000	174	0	2 Ai	antisense to gene(s) PMM1704;
1642264	-	TSS_027833	1000	208	0	21 I	within gene(s) PMM1704;
1642285	-	TSS_027837	1000	627	0	39 I	within gene(s) PMM1704;
1642348	-	TSS_027851	1000	186	0	0 I	within gene(s) PMM1704;
1642378	-	TSS_027853	1000	122	0	3 I	within gene(s) PMM1704;
1642387	-	TSS_027854	1000	161	0	0 I	within gene(s) PMM1704;
1642402	-	TSS_027855	1000	117	0	0 I	within gene(s) PMM1704;
1642444	-	TSS_027862	1000	894	0	36 I	within gene(s) PMM1704;
1642477	-	TSS_027869	1000	436	0	27 I	within gene(s) PMM1704;
1642606	-	TSS_027884	1000	580	0	16 I	within gene(s) PMM1704;
1642639	-	TSS_027891	1000	157	0	9 I	within gene(s) PMM1704;
1642648	-	TSS_027892	1000	104	0	0 I	within gene(s) PMM1704;
1642678	-	TSS_027894	1000	110	0	3 I	within gene(s) PMM1704;
1642717	-	TSS_027896	1000	117	0	0 I	within gene(s) PMM1704;
1642759	-	TSS_027904	1000	360	0	30 I	within gene(s) PMM1704;
1642771	-	TSS_027905	1000	262	0	15 I	within gene(s) PMM1704;
1642795	-	TSS_027911	1000	263	0	37 I	within gene(s) PMM1704;
1642810	+	TSS_012142	1000	201	0	0 Ai	antisense to gene(s) PMM1704;
1642864	-	TSS_027928	1000	178	0	36 I	within gene(s) PMM1704;
1642900	-	TSS_027935	1000	568	0	42 I	within gene(s) PMM1704;
1642954	-	TSS_027949	1000	307	0	4 I	within gene(s) PMM1704;
1643008	-	TSS_027956	1000	102	0	3 I	within gene(s) PMM1704;
1643137	-	TSS_027969	1000	118	0	0 I	within gene(s) PMM1704;
1643144	+	TSS_012145	1000	409	0	5 Ai	antisense to gene(s) PMM1704;
1643153	+	TSS_012147	1000	362	0	1 Ai	antisense to gene(s) PMM1704;
1643179	-	TSS_027976	1000	150	0	15 I	within gene(s) PMM1704;
1643197	-	TSS_027979	1000	196	0	4 I	within gene(s) PMM1704;
1643239	-	TSS_027985	1000	523	0	6 I	within gene(s) PMM1704;
1643316	+	TSS_012149	1000	160	0	0 PAi	182nt upstream of gene PMM1705; antisense to gene(s) PMM1704;
1643320	-	TSS_027991	1000	107	0	9 I	within gene(s) PMM1704;
1643332	-	TSS_027992	1000	102	0	3 I	within gene(s) PMM1704;
1643399	-	TSS_027997	1000	12841	0	7 P	19nt upstream of gene PMM1704;
1644415	+	TSS_012152	1000	1676	0	2 P	50nt upstream of gene PMM1706;
1645357	-	TSS_028006	1000	225	0	0 I	within gene(s) PMM1707;
1645691	-	TSS_028017	1000	131	0	0 I	within gene(s) PMM1707;
1646195	-	TSS_028026	1000	950	0	5 P	48nt upstream of gene PMM1707;
1646216	-	TSS_028029	1000	2235	0	0 P	69nt upstream of gene PMM1707;
1646481	+	TSS_012177	1000	130	0	0 IP	within gene(s) PMM1708; 217nt upstream of gene PMM1709;
1646490	+	TSS_012178	1000	136	0	0 IP	within gene(s) PMM1708; 208nt upstream of gene PMM1709;
1646517	+	TSS_012181	1000	126	0	7 IP	within gene(s) PMM1708; 181nt upstream of gene PMM1709;
1646542	+	TSS_012184	1000	142	0	3 IP	within gene(s) PMM1708; 156nt upstream of gene PMM1709;
1647512	-	TSS_028034	1000	107	0	0 Ai	antisense to gene(s) PMM1709;
1650015	-	TSS_028048	1000	103	0	3 I	within gene(s) PMM1712;
1654524	+	TSS_012211	1000	159	0	2 I	within gene(s) PMM1714;
1656926	+	TSS_012217	1000	160	0	0 I	within gene(s) PMM1716;

**Class represents the classification of the start site. I represents internal start sites, Ai represents antisense start sites, P represents primary start sites, IP represents internal or primary start sites, PAi represents primary or Antisense start sites, Ad represnts Antisense or downstream IAd represents internal Antisense or downstream and O represents orphan.
