# Supplementary material for: Nitrogen cost minimization is promoted by structural changes in the transcriptome of N-deprived Prochlorococcus cells
Source: ISME J. 2017 Jun 6;11(10):2267–78. doi: 10.1038/ismej.2017.88 (PMC5607370; doi:10.1038/ismej.2017.88)
Supplement: Supplementary Table 11 [file ismej201788x18.pdf]

Table S11. N-Starved Transcriptional Start Sites at 24 Hours Post Starvation Identified by TSSAR.

Position	Strand	ID	Score	Difference	p.Value	Positional.Ur	Class	Comment
158	+	TSS_000004	1000	889	0	5	P	16nt upstream of gene PMM0001;
1080	+	TSS_000021	1000	195	0	12	I	within gene(s) PMM0001;
1991	+	TSS_000032	1000	409	0	4	IP	within gene(s) PMM0002; 53nt upstream of gene PMM0003;
2075	+	TSS_000035	1000	137	0	0	I	within gene(s) PMM0003;
2084	+	TSS_000036	1000	3263	0	1	I	within gene(s) PMM0003;
2919	-	TSS_016789	1000	127	0	1	Ai	antisense to gene(s) PMM0003;
3197	+	TSS_000055	1000	184	0	5	I	within gene(s) PMM0003;
5103	+	TSS_000067	1000	183	0	0	I	within gene(s) PMM0004;
8361	-	TSS_016812	1000	673	0	0	P	33nt upstream of gene PMM0005;
9568	+	TSS_000072	1000	168	0	5	Ai	antisense to gene(s) PMM0007;
10218	-	TSS_016822	1000	559	0	0	I	within gene(s) PMM0007;
10339	+	TSS_000075	1000	2534	0	4	P	28nt upstream of gene PMM0008;
10349	+	TSS_000078	1000	787	0	3	P	18nt upstream of gene PMM0008;
14116	-	TSS_016833	1000	1806	0	3	Ai	antisense to gene(s) PMM0011;
14568	+	TSS_000124	1000	654	0	1	P	17nt upstream of gene PMM0012;
14769	-	TSS_016835	1000	205	0	0	Ai	antisense to gene(s) PMM0012;
14964	-	TSS_016836	1000	408	0	6	Ai	antisense to gene(s) PMM0012;
15146	+	TSS_000131	1000	101	0	0	I	within gene(s) PMM0012;
16016	+	TSS_000139	1000	24112	0	3	P	2nt upstream of gene PMM0013;
16255	-	TSS_016844	1000	277	0	1	Ai	antisense to gene(s) PMM0013;
16313	-	TSS_016846	1000	184	0	1	Ai	antisense to gene(s) PMM0013;
16507	+	TSS_000151	1000	131	0	21	I	within gene(s) PMM0013;
17066	+	TSS_000154	1000	105	0	1	Ai	antisense to gene(s) PMM0014;
17671	+	TSS_000157	1000	466	0	2	PAi	41nt upstream of gene PMM0015; antisense to gene(s) PMM0014;
17711	+	TSS_000160	1000	137	0	4	P	1nt upstream of gene PMM0015;
18081	-	TSS_016853	1000	180	0	0	Ai	antisense to gene(s) PMM0015;
18267	+	TSS_000168	1000	913	0	0	P	16nt upstream of gene PMM0016;
18667	+	TSS_000212	1000	245	0	21	I	within gene(s) PMM0016;
18703	+	TSS_000214	1000	145	0	0	I	within gene(s) PMM0016;
18739	+	TSS_000223	1000	221	0	30	I	within gene(s) PMM0016;
18793	+	TSS_000236	1000	108	0	41	IP	within gene(s) PMM0016; 241nt upstream of gene PMM0017;
18817	+	TSS_000238	1000	108	0	0	IP	within gene(s) PMM0016; 217nt upstream of gene PMM0017;
18852	-	TSS_016862	1000	115	0	0	Ai	antisense to gene(s) PMM0016;
18886	+	TSS_000241	1000	130	0	0	IP	within gene(s) PMM0016; 148nt upstream of gene PMM0017;
19598	+	TSS_000256	1000	128	0	11	I	within gene(s) PMM0017;
23179	-	TSS_016874	1000	234	0	1	I	within gene(s) PMM0022;
24122	+	TSS_000270	1000	1946	0	3	P	28nt upstream of gene PMM0023;
25132	+	TSS_000311	1000	115	0	5	I	within gene(s) PMM0023;
25886	-	TSS_016893	1000	1213	0	0	I	within gene(s) PMM0024;
26468	-	TSS_016897	1000	432	0	1	I	within gene(s) PMM0025;
27201	+	TSS_000321	1000	122	0	0	PAi	86nt upstream of gene PMM0026; antisense to gene(s) PMM0025;
27273	+	TSS_000322	1000	2020	0	2	P	14nt upstream of gene PMM0026;
27294	-	TSS_016911	1000	113	0	0	PAi	51nt upstream of gene PMM0025; antisense to gene(s) PMM0026;
27367	+	TSS_000324	1000	831	0	0	I	within gene(s) PMM0026;
27478	-	TSS_016913	1000	523	0	1	PAi	235nt upstream of gene PMM0025; antisense to gene(s) PMM0026;
27755	+	TSS_000331	1000	850	0	1	IP	within gene(s) PMM0026; 92nt upstream of gene PMM0027;
28211	+	TSS_000340	1000	106	0	0	I	within gene(s) PMM0027;
30271	+	TSS_000343	1000	1191	0	3	I	within gene(s) PMM0030;
30911	-	TSS_016931	1000	189	0	0	I	within gene(s) PMM0031;
30946	-	TSS_016933	1000	311	0	4	P	16nt upstream of gene PMM0031;
31307	-	TSS_016937	1000	244	0	16	I	within gene(s) PMM0032;
31334	-	TSS_016945	1000	374	0	14	I	within gene(s) PMM0032;
31393	-	TSS_016956	1000	343	0	8	I	within gene(s) PMM0032;
31536	-	TSS_016967	1000	6322	0	3	P	16nt upstream of gene PMM0032;
31640	+	TSS_000362	1000	386	0	2	Ai	antisense to gene(s) PMM0033;
31718	+	TSS_000364	1000	124	0	0	Ai	antisense to gene(s) PMM0033;
32109	-	TSS_016976	1000	156	0	6	P	18nt upstream of gene PMM0033;
32374	+	TSS_000368	1000	365	0	7	I	within gene(s) PMM0034;
33078	+	TSS_000378	1000	203	0	0	Ai	antisense to gene(s) PMM0035;
33332	+	TSS_000380	1000	238	0	1	Ai	antisense to gene(s) PMM0035;
33511	-	TSS_017018	1000	139	0	21	I	within gene(s) PMM0035;
33547	-	TSS_017025	1000	144	0	8	I	within gene(s) PMM0035;
33713	-	TSS_017037	1000	4942	0	2	P	16nt upstream of gene PMM0035;
34181	+	TSS_000385	1000	108	0	0	I	within gene(s) PMM0036;
34926	+	TSS_000386	1000	515	0	0	I	within gene(s) PMM0037;
37053	-	TSS_017048	1000	225	0	1	Ai	antisense to gene(s) PMM0038;
37342	+	TSS_000403	1000	947	0	1	P	18nt upstream of gene PMM0039;
43346	+	TSS_000418	1000	201	0	0	Ai	antisense to gene(s) PMM0043;
43359	+	TSS_000419	1000	211	0	0	Ai	antisense to gene(s) PMM0043;
44029	+	TSS_000420	1000	774	0	1	Ai	antisense to gene(s) PMM0043;
44177	-	TSS_017100	1000	341	0	0	I	within gene(s) PMM0043;
44205	-	TSS_017101	1000	736	0	0	P	15nt upstream of gene PMM0043;
44220	-	TSS_017102	1000	873	0	0	P	30nt upstream of gene PMM0043;
44838	-	TSS_017105	1000	275	0	2	Ai	antisense to gene(s) PMM0044;
46967	-	TSS_017111	1000	122	0	10	IAd	within gene(s) PMM0045; antisense to gene(s) PMM0044 (3nt downstream);

47672	-	TSS_017117	1000	233	0	5	I	within gene(s) PMM0045;
47889	+	TSS_000447	1000	163	0	3	Ai	antisense to gene(s) PMM0045;
49002	+	TSS_000451	1000	3169	0	3	P	15nt upstream of gene PMM0046;
49452	-	TSS_017124	1000	137	0	0	Ai	antisense to gene(s) PMM0046;
50616	+	TSS_000473	1000	542	0	0	P	56nt upstream of gene PMM0048;
50659	+	TSS_000475	1000	770	0	3	P	13nt upstream of gene PMM0048;
52823	+	TSS_000531	1000	432	0	5	P	44nt upstream of gene PMM0050;
52948	-	TSS_017139	1000	264	0	1	PAi	186nt upstream of gene PMM0049; antisense to gene(s) PMM0050;
54396	-	TSS_017150	1000	127	0	60	I	within gene(s) PMM0051;
54638	+	TSS_000537	1000	103	0	0	Ai	antisense to gene(s) PMM0051;
56405	+	TSS_000540	1000	161	0	1	O	-
56601	-	TSS_017174	1000	1888	0	0	O	-
57896	-	TSS_017180	1000	108	0	3	I	within gene(s) PMM0054;
58844	+	TSS_000548	1000	465	0	3	P	207nt upstream of gene PMM0056;
58871	+	TSS_000551	1000	221	0	1	P	180nt upstream of gene PMM0056;
59035	-	TSS_017210	1000	106	0	1	O	-
59479	+	TSS_000556	1000	812	0	1	P	17nt upstream of gene PMM0057;
63006	+	TSS_000573	1000	2784	0	2	IP	within gene(s) PMM0057; 126nt upstream of gene PMM0058;
63116	+	TSS_000575	1000	401	0	1	P	16nt upstream of gene PMM0058;
63461	+	TSS_000580	1000	115	0	3	I	within gene(s) PMM0058;
65570	-	TSS_017232	1000	107	0	1	P	121nt upstream of gene PMM0059;
65752	+	TSS_000587	1000	311	0	1	P	89nt upstream of gene PMM0060;
66151	+	TSS_000590	1000	108	0	1	I	within gene(s) PMM0060;
66518	+	TSS_000597	1000	183	0	9	I	within gene(s) PMM0060;
67529	-	TSS_017243	1000	367	0	2	P	16nt upstream of gene PMM0061;
67586	+	TSS_000614	1000	4504	0	2	P	66nt upstream of gene PMM0062;
67856	+	TSS_000622	1000	106	0	6	P	14nt upstream of gene PMM0063;
67872	-	TSS_017248	1000	151	0	0	Ai	antisense to gene(s) PMM0063;
67893	+	TSS_000625	1000	500	0	6	I	within gene(s) PMM0063;
68237	+	TSS_000653	1000	192	0	24	I	within gene(s) PMM0063;
68251	+	TSS_000655	1000	132	0	0	I	within gene(s) PMM0063;
68340	-	TSS_017257	1000	218	0	2	Ai	antisense to gene(s) PMM0063;
68347	+	TSS_000675	1000	141	0	54	I	within gene(s) PMM0063;
68356	-	TSS_017259	1000	108	0	0	Ai	antisense to gene(s) PMM0063;
68372	-	TSS_017260	1000	126	0	0	Ai	antisense to gene(s) PMM0063;
68394	-	TSS_017261	1000	118	0	0	Ai	antisense to gene(s) PMM0063;
68431	+	TSS_000689	1000	108	0	9	I	within gene(s) PMM0063;
68439	-	TSS_017265	1000	133	0	8	Ai	antisense to gene(s) PMM0063;
68453	+	TSS_000691	1000	229	0	1	I	within gene(s) PMM0063;
68473	+	TSS_000695	1000	110	0	3	I	within gene(s) PMM0063;
68500	+	TSS_000698	1000	138	0	15	I	within gene(s) PMM0063;
68533	+	TSS_000701	1000	157	0	10	I	within gene(s) PMM0063;
68569	+	TSS_000709	1000	308	0	15	I	within gene(s) PMM0063;
68620	+	TSS_000715	1000	555	0	33	I	within gene(s) PMM0063;
68680	+	TSS_000727	1000	132	0	36	I	within gene(s) PMM0063;
69179	-	TSS_017268	1000	224	0	1	IP	within gene(s) PMM0065; 143nt upstream of gene PMM0064;
69361	-	TSS_017275	1000	130	0	0	I	within gene(s) PMM0065;
70887	-	TSS_017296	1000	105	0	0	I	within gene(s) PMM0065;
72284	-	TSS_017308	1000	102	0	0	I	within gene(s) PMM0068;
72400	-	TSS_017309	1000	260	0	0	P	32nt upstream of gene PMM0068;
73190	-	TSS_017313	1000	102	0	0	Ai	antisense to gene(s) PMM0069;
73910	-	TSS_017317	1000	207	0	6	Ai	antisense to gene(s) PMM0069;
74082	-	TSS_017318	1000	172	0	0	Ai	antisense to gene(s) PMM0069;
74118	-	TSS_017319	1000	352	0	0	Ai	antisense to gene(s) PMM0069;
78158	-	TSS_017350	1000	301	0	1	I	within gene(s) PMM0073;
78218	-	TSS_017352	1000	400	0	1	I	within gene(s) PMM0073;
78295	-	TSS_017354	1000	381	0	7	I	within gene(s) PMM0073;
78481	-	TSS_017363	1000	299	0	10	I	within gene(s) PMM0073;
79094	-	TSS_017385	1000	257	0	15	I	within gene(s) PMM0073;
79127	-	TSS_017391	1000	145	0	4	P	6nt upstream of gene PMM0073;
79154	-	TSS_017393	1000	890	0	10	P	33nt upstream of gene PMM0073;
79823	+	TSS_000756	1000	160	0	0	P	22nt upstream of gene PMM0075;
80289	+	TSS_000760	1000	261	0	14	I	within gene(s) PMM0075;
80352	+	TSS_000767	1000	123	0	0	I	within gene(s) PMM0075;
82229	-	TSS_017407	1000	186	0	0	Ai	antisense to gene(s) PMM0076;
83123	+	TSS_000788	1000	159	0	0	I	within gene(s) PMM0077;
84767	+	TSS_000793	1000	106	0	0	PAi	19nt upstream of gene PMM0079; antisense to gene(s) PMM0078;
88872	+	TSS_000818	1000	151	0	6	I	within gene(s) PMM0083;
89200	-	TSS_017429	1000	226	0	5	Ai	antisense to gene(s) PMM0083;
89701	-	TSS_017435	1000	102	0	0	Ai	antisense to gene(s) PMM0083;
90051	+	TSS_000828	1000	363	0	1	I	within gene(s) PMM0084;
91200	+	TSS_000841	1000	3431	0	9	IP	within gene(s) PMM0084; 60nt upstream of gene PMM0085;
91487	-	TSS_017444	1000	329	0	0	Ai	antisense to gene(s) PMM0085;
91506	+	TSS_000851	1000	116	0	3	I	within gene(s) PMM0085;
91711	-	TSS_017446	1000	828	0	1	Ai	antisense to gene(s) PMM0085;
91842	+	TSS_000863	1000	254	0	9	I	within gene(s) PMM0085;
92216	-	TSS_017447	1000	134	0	0	I	within gene(s) PMM0086;

92293	-	TSS_017449	1000	2891	0	1	P	20nt upstream of gene PMM0086;
92350	+	TSS_000864	1000	1691	0	1	P	40nt upstream of gene PMM0087;
92747	+	TSS_000883	1000	1991	0	2	P	17nt upstream of gene PMM0088;
93143	-	TSS_017458	1000	897	0	2	O	-
94679	-	TSS_017460	1000	283	0	3	Ai	antisense to gene(s) PMM0089;
94700	-	TSS_017462	1000	103	0	0	Ai	antisense to gene(s) PMM0089;
94719	-	TSS_017464	1000	146	0	3	Ai	antisense to gene(s) PMM0089;
96428	+	TSS_000903	1000	454	0	2	P	24nt upstream of gene PMM0091;
96671	+	TSS_000925	1000	143	0	9	IP	within gene(s) PMM0091; 149nt upstream of gene PMM0092;
96699	+	TSS_000930	1000	163	0	4	IP	within gene(s) PMM0091; 121nt upstream of gene PMM0092;
96919	-	TSS_017472	1000	185	0	1	Ai	antisense to gene(s) PMM0092;
97181	+	TSS_000942	1000	23806	0	10	P	19nt upstream of gene PMM0093;
98015	-	TSS_017477	1000	505	0	4	I	within gene(s) PMM0095;
98676	-	TSS_017484	1000	315	0	1	P	16nt upstream of gene PMM0095;
104291	+	TSS_000978	1000	144	0	0	I	within gene(s) PMM0100;
105223	-	TSS_017505	1000	111	0	0	I	within gene(s) PMM0101;
105240	-	TSS_017506	1000	106	0	0	I	within gene(s) PMM0101;
105437	-	TSS_017510	1000	420	0	3	I	within gene(s) PMM0101;
105798	-	TSS_017516	1000	119	0	2	I	within gene(s) PMM0101;
105946	-	TSS_017520	1000	502	0	2	P	30nt upstream of gene PMM0101;
107782	-	TSS_017526	1000	115	0	3	I	within gene(s) PMM0103;
107836	-	TSS_017527	1000	109	0	0	I	within gene(s) PMM0103;
109827	+	TSS_000986	1000	113	0	0	Ai	antisense to gene(s) PMM0105;
110281	+	TSS_000988	1000	1422	0	1	Ai	antisense to gene(s) PMM0106;
110757	-	TSS_017548	1000	107	0	1	P	3nt upstream of gene PMM0106;
114786	+	TSS_001005	1000	180	0	0	Ai	antisense to gene(s) PMM0114;
114981	-	TSS_017560	1000	137	0	3	IP	within gene(s) PMM0115; 35nt upstream of gene PMM0114;
115971	+	TSS_001013	1000	167	0	0	Ai	antisense to gene(s) PMM0115;
116575	+	TSS_001016	1000	151	0	18	I	within gene(s) PMM0116;
116623	+	TSS_001026	1000	154	0	21	I	within gene(s) PMM0116;
117055	+	TSS_001046	1000	149	0	6	I	within gene(s) PMM0117;
118346	-	TSS_017603	1000	270	0	1	Ai	antisense to gene(s) PMM0118;
119698	-	TSS_017617	1000	280	0	7	P	16nt upstream of gene PMM0120;
119798	+	TSS_001056	1000	2133	0	2	P	28nt upstream of gene PMM0121;
119905	+	TSS_001060	1000	161	0	0	I	within gene(s) PMM0121;
120990	-	TSS_017630	1000	714	0	12	IP	within gene(s) PMM0123; 240nt upstream of gene PMM0122;
121450	-	TSS_017638	1000	103	0	0	I	within gene(s) PMM0123;
121764	-	TSS_017643	1000	190	0	0	P	29nt upstream of gene PMM0123;
122897	+	TSS_001071	1000	692	0	2	O	-
123251	+	TSS_001072	1000	701	0	1	P	68nt upstream of gene PMM0126;
123779	+	TSS_001076	1000	1394	0	1	I	within gene(s) PMM0126;
123796	+	TSS_001078	1000	294	0	0	I	within gene(s) PMM0126;
125323	+	TSS_001093	1000	2800	0	1	P	35nt upstream of gene PMM0128;
125344	+	TSS_001095	1000	106	0	0	P	14nt upstream of gene PMM0128;
125544	+	TSS_001114	1000	178	0	3	I	within gene(s) PMM0128;
125778	+	TSS_001141	1000	188	0	27	I	within gene(s) PMM0128;
125808	+	TSS_001148	1000	136	0	0	I	within gene(s) PMM0128;
125815	+	TSS_001149	1000	108	0	2	I	within gene(s) PMM0128;
126175	-	TSS_017665	1000	168	0	0	I	within gene(s) PMM0129;
129278	-	TSS_017678	1000	260	0	3	I	within gene(s) PMM0131;
130071	-	TSS_017684	1000	226	0	3	P	34nt upstream of gene PMM0131;
130794	+	TSS_001169	1000	2933	0	1	Ai	antisense to gene(s) PMM0133;
130807	+	TSS_001170	1000	1267	0	0	Ai	antisense to gene(s) PMM0133;
130945	+	TSS_001171	1000	110	0	1	Ai	antisense to gene(s) PMM0133;
132376	+	TSS_001191	1000	132	0	32	I	within gene(s) PMM0134;
132496	+	TSS_001210	1000	115	0	24	I	within gene(s) PMM0134;
132850	-	TSS_017700	1000	397	0	0	Ai	antisense to gene(s) PMM0134;
133247	+	TSS_001240	1000	115	0	3	I	within gene(s) PMM0135;
133300	+	TSS_001243	1000	121	0	1	I	within gene(s) PMM0135;
134228	-	TSS_017707	1000	1902	0	4	Ai	antisense to gene(s) PMM0135;
134332	+	TSS_001253	1000	487	0	1	P	17nt upstream of gene PMM0136;
135334	+	TSS_001257	1000	362	0	0	IP	within gene(s) PMM0136; 37nt upstream of gene PMM0137;
136633	+	TSS_001262	1000	110	0	0	I	within gene(s) PMM0138;
139049	+	TSS_001266	1000	274	0	0	P	49nt upstream of gene PMM0142;
139888	+	TSS_001275	1000	1484	0	1	Ai	antisense to gene(s) PMM0143;
139946	-	TSS_017722	1000	115	0	0	I	within gene(s) PMM0143;
140081	-	TSS_017725	1000	861	0	1	I	within gene(s) PMM0143;
141905	-	TSS_017764	1000	731	0	0	P	38nt upstream of gene PMM0144;
141924	+	TSS_001283	1000	467	0	5	P	34nt upstream of gene PMM0145;
141979	+	TSS_001284	1000	215	0	1	I	within gene(s) PMM0145;
142219	+	TSS_001303	1000	116	0	12	IP	within gene(s) PMM0145; 83nt upstream of gene PMM0146;
142832	-	TSS_017769	1000	113	0	0	Ai	antisense to gene(s) PMM0146;
143021	-	TSS_017778	1000	163	0	8	I	within gene(s) PMM0147;
143488	-	TSS_017786	1000	560	0	24	I	within gene(s) PMM0147;
143536	-	TSS_017799	1000	167	0	12	I	within gene(s) PMM0147;
143883	-	TSS_017808	1000	316	0	12	P	14nt upstream of gene PMM0147;
143931	+	TSS_001326	1000	234	0	3	P	23nt upstream of gene PMM0148;

144701	+	TSS_001337	1000	1794	0	5 P	15nt upstream of gene PMM0149;
144965	+	TSS_001347	1000	369	0	9 I	within gene(s) PMM0149;
145139	+	TSS_001352	1000	144	0	0 I	within gene(s) PMM0149;
145211	+	TSS_001353	1000	395	0	3 I	within gene(s) PMM0149;
145226	+	TSS_001357	1000	702	0	2 I	within gene(s) PMM0149;
145511	+	TSS_001367	1000	315	0	3 I	within gene(s) PMM0149;
145715	+	TSS_001381	1000	230	0	10 I	within gene(s) PMM0149;
145730	+	TSS_001383	1000	141	0	0 I	within gene(s) PMM0149;
145763	+	TSS_001387	1000	123	0	9 I	within gene(s) PMM0149;
146423	-	TSS_017823	1000	243	0	0 Ai	antisense to gene(s) PMM0149;
146794	+	TSS_001424	1000	19740	0	2 P	19nt upstream of gene PMM0150;
147116	+	TSS_001429	1000	108	0	0 I	within gene(s) PMM0150;
147332	+	TSS_001437	1000	420	0	0 I	within gene(s) PMM0150;
147440	+	TSS_001441	1000	220	0	0 I	within gene(s) PMM0150;
147622	-	TSS_017830	1000	142	0	0 Ai	antisense to gene(s) PMM0150;
147731	-	TSS_017831	1000	256	0	1 Ai	antisense to gene(s) PMM0150;
147807	+	TSS_001453	1000	234	0	1 I	within gene(s) PMM0150;
148515	+	TSS_001469	1000	259	0	1 P	169nt upstream of gene PMM0151;
149427	+	TSS_001472	1000	408	0	2 P	20nt upstream of gene PMM0152;
150290	+	TSS_001489	1000	265	0	7 I	within gene(s) PMM0152;
150329	+	TSS_001492	1000	120	0	6 I	within gene(s) PMM0152;
150387	-	TSS_017842	1000	222	0	4 Ai	antisense to gene(s) PMM0152;
151351	-	TSS_017854	1000	143	0	4 I	within gene(s) PMM0153;
151372	+	TSS_001497	1000	196	0	0 PAi	205nt upstream of gene PMM0154; antisense to gene(s) PMM0153;
151412	+	TSS_001499	1000	564	0	2 PAi	165nt upstream of gene PMM0154; antisense to gene(s) PMM0153;
151546	-	TSS_017856	1000	179	0	2 P	47nt upstream of gene PMM0153;
151556	+	TSS_001501	1000	1106	0	5 P	21nt upstream of gene PMM0154;
151566	+	TSS_001504	1000	2828	0	2 P	11nt upstream of gene PMM0154;
154617	-	TSS_017897	1000	4269	0	60 IP	within gene(s) PMM0159; 214nt upstream of gene PMM0158;
154809	-	TSS_017911	1000	381	0	18 I	within gene(s) PMM0159;
154854	-	TSS_017920	1000	116	0	27 I	within gene(s) PMM0159;
154964	-	TSS_017933	1000	441	0	2 I	within gene(s) PMM0159;
155061	-	TSS_017935	1000	427	0	6 P	18nt upstream of gene PMM0159;
155927	-	TSS_017950	1000	185	0	6 I	within gene(s) PMM0160;
156249	-	TSS_017959	1000	247	0	6 P	18nt upstream of gene PMM0160;
157478	-	TSS_017985	1000	408	0	0 P	26nt upstream of gene PMM0161;
160182	-	TSS_017999	1000	102	0	4 I	within gene(s) PMM0164;
160552	-	TSS_018022	1000	128	0	1 I	within gene(s) PMM0164;
160650	-	TSS_018028	1000	124	0	1 I	within gene(s) PMM0164;
160684	-	TSS_018030	1000	1683	0	5 P	18nt upstream of gene PMM0164;
161048	+	TSS_001534	1000	286	0	0 P	21nt upstream of gene PMM0166;
161898	+	TSS_001543	1000	149	0	0 Ai	antisense to gene(s) PMM0167;
162263	-	TSS_018036	1000	305	0	2 P	61nt upstream of gene PMM0167;
162318	+	TSS_001545	1000	971	0	2 P	21nt upstream of gene PMM0168;
163563	-	TSS_018047	1000	124	0	0 I	within gene(s) PMM0169;
163802	-	TSS_018054	1000	368	0	0 P	32nt upstream of gene PMM0169;
163830	+	TSS_001557	1000	347	0	0 I	within gene(s) PMM0170;
165910	+	TSS_001567	1000	15815	0	3 P	15nt upstream of gene PMM0172;
166228	+	TSS_001589	1000	180	0	15 I	within gene(s) PMM0172;
166270	+	TSS_001601	1000	367	0	15 I	within gene(s) PMM0172;
166453	+	TSS_001610	1000	108	0	0 I	within gene(s) PMM0172;
166513	+	TSS_001616	1000	362	0	7 I	within gene(s) PMM0172;
166537	+	TSS_001623	1000	186	0	15 I	within gene(s) PMM0172;
166576	+	TSS_001635	1000	626	0	25 I	within gene(s) PMM0172;
166597	+	TSS_001638	1000	175	0	12 I	within gene(s) PMM0172;
166605	-	TSS_018062	1000	213	0	0 Ai	antisense to gene(s) PMM0172;
166663	+	TSS_001644	1000	116	0	3 I	within gene(s) PMM0172;
166807	+	TSS_001657	1000	110	0	6 I	within gene(s) PMM0172;
172122	-	TSS_018072	1000	117	0	0 Ai	antisense to gene(s) PMM0177;
173360	-	TSS_018077	1000	310	0	2 P	17nt upstream of gene PMM0179;
173404	+	TSS_001682	1000	1083	0	1 P	16nt upstream of gene PMM0180;
176904	-	TSS_018089	1000	120	0	0 Ai	antisense to gene(s) PMM0185;
178914	-	TSS_018097	1000	1151	0	3 I	within gene(s) PMM0187;
180315	+	TSS_001708	1000	264	0	0 Ai	antisense to gene(s) PMM0188;
180517	+	TSS_001710	1000	118	0	0 Ai	antisense to gene(s) PMM0188;
182433	-	TSS_018104	1000	297	0	2 PAi	92nt upstream of gene PMM0189; antisense to gene(s) PMM0190;
183419	-	TSS_018108	1000	205	0	0 I	within gene(s) PMM0191;
188865	+	TSS_001733	1000	866	0	1 P	18nt upstream of gene PMM0195;
189007	+	TSS_001736	1000	434	0	1 I	within gene(s) PMM0195;
189468	+	TSS_001764	1000	139	0	0 I	within gene(s) PMM0195;
193821	+	TSS_001783	1000	103	0	0 Ai	antisense to gene(s) PMM0199;
195220	-	TSS_018155	1000	107	0	0 IP	within gene(s) PMM0201; 249nt upstream of gene PMM0200;
195266	+	TSS_001789	1000	1093	0	0 Ai	antisense to gene(s) PMM0201;
195316	-	TSS_018164	1000	216	0	15 I	within gene(s) PMM0201;
195331	-	TSS_018168	1000	324	0	9 I	within gene(s) PMM0201;
195367	-	TSS_018174	1000	211	0	14 I	within gene(s) PMM0201;
195387	-	TSS_018178	1000	245	0	3 I	within gene(s) PMM0201;

195641	-	TSS_018192	1000	104	0	12	IP	within gene(s) PMM0202; 229nt upstream of gene PMM0201;
195665	-	TSS_018195	1000	146	0	1	I	within gene(s) PMM0202;
195761	-	TSS_018203	1000	255	0	1	I	within gene(s) PMM0202;
195809	-	TSS_018207	1000	225	0	3	I	within gene(s) PMM0202;
195869	-	TSS_018215	1000	117	0	14	I	within gene(s) PMM0202;
196098	-	TSS_018220	1000	154	0	0	P	130nt upstream of gene PMM0202;
196154	+	TSS_001798	1000	145	0	0	Ad	antisense to gene(s) PMM0203 (8nt downstream);
196510	+	TSS_001802	1000	173	0	1	Ai	antisense to gene(s) PMM0203;
196639	-	TSS_018250	1000	230	0	19	I	within gene(s) PMM0203;
196677	+	TSS_001805	1000	131	0	0	Ai	antisense to gene(s) PMM0203;
196912	-	TSS_018258	1000	176	0	1	P	44nt upstream of gene PMM0203;
197001	-	TSS_018261	1000	1005	0	0	IP	within gene(s) PMM0204; 133nt upstream of gene PMM0203;
197448	-	TSS_018279	1000	967	0	9	IP	within gene(s) PMM0205; 88nt upstream of gene PMM0204;
197685	+	TSS_001809	1000	203	0	0	Ai	antisense to gene(s) PMM0205;
197771	-	TSS_018298	1000	232	0	0	I	within gene(s) PMM0205;
197837	-	TSS_018303	1000	249	0	2	I	within gene(s) PMM0205;
197876	-	TSS_018310	1000	188	0	23	I	within gene(s) PMM0205;
198069	-	TSS_018320	1000	716	0	5	P	34nt upstream of gene PMM0205;
198181	-	TSS_018325	1000	660	0	0	IP	within gene(s) PMM0206; 146nt upstream of gene PMM0205;
198382	-	TSS_018326	1000	465	0	0	P	33nt upstream of gene PMM0206;
199933	-	TSS_018334	1000	203	0	4	I	within gene(s) PMM0207;
201442	+	TSS_001822	1000	4280	0	5	P	14nt upstream of gene PMM0208;
202306	-	TSS_018347	1000	109	0	0	Ai	antisense to gene(s) PMM0208;
203176	-	TSS_018356	1000	332	0	2	I	within gene(s) PMM0209;
203219	-	TSS_018359	1000	737	0	0	I	within gene(s) PMM0209;
204777	-	TSS_018365	1000	151	0	3	P	41nt upstream of gene PMM0210;
204910	+	TSS_001874	1000	1999	0	4	P	82nt upstream of gene PMM0211;
205343	+	TSS_001905	1000	142	0	24	I	within gene(s) PMM0211;
205563	-	TSS_018371	1000	173	0	6	Ai	antisense to gene(s) PMM0211;
205900	-	TSS_018381	1000	1373	0	1	Ai	antisense to gene(s) PMM0211;
206424	-	TSS_018384	1000	987	0	3	IP	within gene(s) PMM0213; 172nt upstream of gene PMM0212;
206963	-	TSS_018392	1000	146	0	4	I	within gene(s) PMM0213;
207653	-	TSS_018403	1000	115	0	0	I	within gene(s) PMM0214;
207728	-	TSS_018409	1000	181	0	11	I	within gene(s) PMM0214;
207935	-	TSS_018421	1000	147	0	9	I	within gene(s) PMM0214;
208325	-	TSS_018450	1000	115	0	10	I	within gene(s) PMM0214;
208538	-	TSS_018465	1000	165	0	12	I	within gene(s) PMM0214;
208702	+	TSS_001946	1000	229	0	1	Ai	antisense to gene(s) PMM0214;
208724	-	TSS_018475	1000	157	0	16	I	within gene(s) PMM0214;
208811	-	TSS_018480	1000	215	0	2	I	within gene(s) PMM0214;
208853	-	TSS_018485	1000	116	0	6	I	within gene(s) PMM0214;
208944	-	TSS_018491	1000	8749	0	4	P	28nt upstream of gene PMM0214;
209113	+	TSS_001948	1000	698	0	1	P	31nt upstream of gene PMM0215;
210185	+	TSS_001966	1000	299	0	1	P	16nt upstream of gene PMM0216;
211988	-	TSS_018500	1000	1074	0	1	Ai	antisense to gene(s) PMM0217;
212936	-	TSS_018501	1000	104	0	0	Ai	antisense to gene(s) PMM0217;
213029	+	TSS_001981	1000	430	0	6	P	33nt upstream of gene PMM0218;
214116	+	TSS_001990	1000	2051	0	5	P	17nt upstream of gene PMM0219;
214675	-	TSS_018508	1000	7057	0	5	P	19nt upstream of gene PMM0220;
216078	+	TSS_001997	1000	255	0	0	I	within gene(s) PMM0222;
216745	+	TSS_002008	1000	340699	0	41	P	62nt upstream of gene PMM0223;
216803	+	TSS_002041	1000	2760	0	12	P	4nt upstream of gene PMM0223;
216861	+	TSS_002087	1000	9157	0	86	I	within gene(s) PMM0223;
217002	+	TSS_002179	1000	6314	0	134	I	within gene(s) PMM0223;
217090	-	TSS_018517	1000	514	0	6	Ai	antisense to gene(s) PMM0223;
217169	-	TSS_018519	1000	2532	0	1	Ai	antisense to gene(s) PMM0223;
217184	-	TSS_018521	1000	120	0	0	Ai	antisense to gene(s) PMM0223;
217197	+	TSS_002307	1000	8084	0	204	I	within gene(s) PMM0223;
217293	+	TSS_002360	1000	14735	0	52	I	within gene(s) PMM0223;
217301	-	TSS_018522	1000	113	0	0	Ai	antisense to gene(s) PMM0223;
217328	-	TSS_018523	1000	1002	0	0	Ai	antisense to gene(s) PMM0223;
217380	+	TSS_002417	1000	2773	0	169	I	within gene(s) PMM0223;
217407	-	TSS_018526	1000	143	0	0	Ai	antisense to gene(s) PMM0223;
217415	-	TSS_018527	1000	138	0	0	Ai	antisense to gene(s) PMM0223;
217551	+	TSS_002537	1000	1192	0	144	I	within gene(s) PMM0223;
217587	-	TSS_018530	1000	111	0	0	Ai	antisense to gene(s) PMM0223;
217666	-	TSS_018531	1000	194	0	4	Ai	antisense to gene(s) PMM0223;
217681	-	TSS_018533	1000	136	0	0	Ai	antisense to gene(s) PMM0223;
217826	-	TSS_018538	1000	142	0	0	Ai	antisense to gene(s) PMM0223;
217978	+	TSS_002639	1000	2712	0	2	P	24nt upstream of gene PMM0224;
218599	+	TSS_002670	1000	372	0	21	I	within gene(s) PMM0224;
218695	+	TSS_002682	1000	171	0	1	I	within gene(s) PMM0224;
218813	-	TSS_018549	1000	108	0	2	Ai	antisense to gene(s) PMM0224;
219988	+	TSS_002705	1000	179	0	0	Ai	antisense to gene(s) PMM0226;
220009	+	TSS_002707	1000	485	0	1	Ai	antisense to gene(s) PMM0226;
220029	-	TSS_018558	1000	457	0	27	I	within gene(s) PMM0226;
220055	+	TSS_002708	1000	494	0	0	Ai	antisense to gene(s) PMM0226;

220073	+	TSS_002709	1000	533	0	0	Ai	antisense to gene(s) PMM0226;
220087	+	TSS_002710	1000	252	0	0	Ai	antisense to gene(s) PMM0226;
220158	-	TSS_018579	1000	148	0	12	I	within gene(s) PMM0226;
220272	-	TSS_018599	1000	700	0	54	I	within gene(s) PMM0226;
220296	-	TSS_018605	1000	287	0	3	I	within gene(s) PMM0226;
220320	-	TSS_018607	1000	301	0	42	I	within gene(s) PMM0226;
220370	-	TSS_018620	1000	666	0	0	I	within gene(s) PMM0226;
220444	+	TSS_002714	1000	160	0	4	Ai	antisense to gene(s) PMM0226;
220473	-	TSS_018632	1000	131	0	3	I	within gene(s) PMM0226;
220488	-	TSS_018634	1000	120	0	3	I	within gene(s) PMM0226;
220509	-	TSS_018636	1000	173	0	21	I	within gene(s) PMM0226;
220578	-	TSS_018650	1000	575	0	72	I	within gene(s) PMM0226;
220638	-	TSS_018669	1000	144	0	8	I	within gene(s) PMM0226;
220686	-	TSS_018677	1000	103	0	18	I	within gene(s) PMM0226;
220701	-	TSS_018680	1000	114	0	8	I	within gene(s) PMM0226;
220764	-	TSS_018687	1000	108	0	21	I	within gene(s) PMM0226;
220786	+	TSS_002718	1000	165	0	3	Ai	antisense to gene(s) PMM0226;
220908	-	TSS_018701	1000	108	0	0	I	within gene(s) PMM0226;
221025	-	TSS_018713	1000	207	0	15	I	within gene(s) PMM0226;
221043	-	TSS_018716	1000	147	0	9	I	within gene(s) PMM0226;
221070	-	TSS_018722	1000	180	0	4	I	within gene(s) PMM0226;
221154	-	TSS_018733	1000	507	0	3	I	within gene(s) PMM0226;
221259	-	TSS_018737	1000	212	0	4	I	within gene(s) PMM0226;
221460	-	TSS_018754	1000	205	0	21	I	within gene(s) PMM0226;
221632	-	TSS_018767	1000	6567	0	4	P	16nt upstream of gene PMM0226;
221701	+	TSS_002727	1000	255	0	0	Ai	antisense to gene(s) PMM0227;
221978	+	TSS_002729	1000	431	0	0	Ai	antisense to gene(s) PMM0227;
222262	-	TSS_018775	1000	131	0	0	I	within gene(s) PMM0227;
223179	-	TSS_018787	1000	101	0	12	I	within gene(s) PMM0228;
223194	-	TSS_018789	1000	156	0	7	I	within gene(s) PMM0228;
223245	-	TSS_018795	1000	378	0	15	I	within gene(s) PMM0228;
223269	-	TSS_018802	1000	708	0	9	I	within gene(s) PMM0228;
223311	-	TSS_018810	1000	550	0	36	I	within gene(s) PMM0228;
223386	-	TSS_018821	1000	524	0	18	I	within gene(s) PMM0228;
223413	-	TSS_018827	1000	113	0	3	I	within gene(s) PMM0228;
223467	-	TSS_018831	1000	139	0	0	I	within gene(s) PMM0228;
223529	-	TSS_018834	1000	135	0	1	I	within gene(s) PMM0228;
223559	-	TSS_018838	1000	112	0	14	I	within gene(s) PMM0228;
223578	-	TSS_018843	1000	652	0	19	I	within gene(s) PMM0228;
223641	-	TSS_018853	1000	193	0	0	I	within gene(s) PMM0228;
223665	-	TSS_018856	1000	129	0	0	I	within gene(s) PMM0228;
223706	-	TSS_018858	1000	1138	0	0	I	within gene(s) PMM0228;
223847	-	TSS_018859	1000	1395	0	1	P	140nt upstream of gene PMM0228;
225530	+	TSS_002743	1000	195	0	4	P	60nt upstream of gene PMM0231;
225576	+	TSS_002744	1000	1320	0	0	P	14nt upstream of gene PMM0231;
225772	+	TSS_002747	1000	110	0	1	P	37nt upstream of gene PMM0232;
225783	+	TSS_002748	1000	201	0	0	P	26nt upstream of gene PMM0232;
227265	-	TSS_018873	1000	104	0	0	IP	within gene(s) PMM0234; 96nt upstream of gene PMM0233;
227959	+	TSS_002752	1000	1203	0	0	P	28nt upstream of gene PMM0235;
228037	+	TSS_002753	1000	123	0	0	I	within gene(s) PMM0235;
228140	+	TSS_002758	1000	127	0	3	I	within gene(s) PMM0235;
229314	-	TSS_018891	1000	218	0	0	O	-
229472	+	TSS_002769	1000	199	0	0	P	167nt upstream of gene PMM0237;
230878	-	TSS_018895	1000	601	0	5	Ai	antisense to gene(s) PMM0238;
231853	+	TSS_002799	1000	112	0	6	I	within gene(s) PMM0238;
232150	+	TSS_002801	1000	164	0	2	I	within gene(s) PMM0238;
232913	-	TSS_018903	1000	108	0	0	Ai	antisense to gene(s) PMM0238;
233913	-	TSS_018904	1000	110	0	2	P	20nt upstream of gene PMM0239;
237523	-	TSS_018934	1000	1856	0	18	IP	within gene(s) PMM0244; 199nt upstream of gene PMM0243;
237737	-	TSS_018943	1000	406	0	4	I	within gene(s) PMM0244;
237987	-	TSS_018946	1000	112	0	4	IP	within gene(s) PMM0245; 68nt upstream of gene PMM0244;
238520	-	TSS_018952	1000	936	0	1	P	20nt upstream of gene PMM0245;
238693	+	TSS_002819	1000	2873	0	3	P	12nt upstream of gene PMM0246;
239672	-	TSS_018956	1000	139	0	3	Ai	antisense to gene(s) PMM0247;
241176	-	TSS_018959	1000	211	0	0	IP	within gene(s) PMM0250; 35nt upstream of gene PMM0249;
242059	+	TSS_002831	1000	200	0	0	PAi	162nt upstream of gene PMM0252; antisense to gene(s) PMM0251;
242165	-	TSS_018962	1000	463	0	4	P	24nt upstream of gene PMM0251;
242185	+	TSS_002833	1000	2689	0	2	P	36nt upstream of gene PMM0252;
242475	+	TSS_002839	1000	548	0	1	P	27nt upstream of gene PMM0253;
242483	+	TSS_002840	1000	1196	0	0	P	19nt upstream of gene PMM0253;
244978	+	TSS_002847	1000	209	0	4	Ai	antisense to gene(s) PMM0255;
245524	-	TSS_018971	1000	769	0	11	IP	within gene(s) PMM0256; 219nt upstream of gene PMM0255;
245888	-	TSS_018983	1000	176	0	2	I	within gene(s) PMM0256;
246248	+	TSS_002851	1000	4186	0	4	Ai	antisense to gene(s) PMM0256;
246731	-	TSS_018998	1000	2410	0	5	IP	within gene(s) PMM0257; 20nt upstream of gene PMM0256;
248216	-	TSS_019005	1000	322	0	12	IP	within gene(s) PMM0258; 190nt upstream of gene PMM0257;
248609	-	TSS_019035	1000	201	0	9	I	within gene(s) PMM0258;

248630	-	TSS_019039	1000	120	0	15	I	within gene(s) PMM0258;
249242	-	TSS_019077	1000	807	0	2	I	within gene(s) PMM0258;
249485	+	TSS_002871	1000	275	0	2	P	22nt upstream of gene PMM0259;
251358	+	TSS_002876	1000	175	0	3	Ai	antisense to gene(s) PMM0261;
252586	+	TSS_002884	1000	530710	0	3	P	46nt upstream of gene PMM0263;
252656	+	TSS_002889	1000	3690	0	7	I	within gene(s) PMM0263;
252667	+	TSS_002891	1000	1114	0	0	I	within gene(s) PMM0263;
252683	+	TSS_002892	1000	279	0	0	I	within gene(s) PMM0263;
252710	+	TSS_002896	1000	2014	0	3	I	within gene(s) PMM0263;
252719	+	TSS_002897	1000	3584	0	0	I	within gene(s) PMM0263;
252735	+	TSS_002902	1000	1979	0	9	I	within gene(s) PMM0263;
252761	+	TSS_002905	1000	581	0	5	I	within gene(s) PMM0263;
252794	+	TSS_002906	1000	352	0	0	I	within gene(s) PMM0263;
252809	+	TSS_002908	1000	1822	0	12	I	within gene(s) PMM0263;
252835	-	TSS_019088	1000	140	0	0	Ai	antisense to gene(s) PMM0263;
252851	+	TSS_002913	1000	368	0	0	I	within gene(s) PMM0263;
252875	+	TSS_002918	1000	5844	0	6	I	within gene(s) PMM0263;
252893	+	TSS_002919	1000	3880	0	0	I	within gene(s) PMM0263;
252895	-	TSS_019089	1000	166	0	0	Ai	antisense to gene(s) PMM0263;
252904	-	TSS_019090	1000	123	0	0	Ai	antisense to gene(s) PMM0263;
252926	+	TSS_002925	1000	1698	0	24	I	within gene(s) PMM0263;
252936	+	TSS_002928	1000	352	0	0	I	within gene(s) PMM0263;
252950	+	TSS_002929	1000	600	0	0	I	within gene(s) PMM0263;
252986	+	TSS_002930	1000	998	0	4	I	within gene(s) PMM0263;
253007	+	TSS_002933	1000	462	0	2	I	within gene(s) PMM0263;
253023	-	TSS_019092	1000	838	0	4	Ai	antisense to gene(s) PMM0263;
253040	+	TSS_002937	1000	494	0	15	I	within gene(s) PMM0263;
253056	+	TSS_002941	1000	237	0	0	I	within gene(s) PMM0263;
253072	+	TSS_002942	1000	4618	0	1	I	within gene(s) PMM0263;
253080	+	TSS_002944	1000	654	0	8	I	within gene(s) PMM0263;
253114	-	TSS_019095	1000	210	0	0	Ai	antisense to gene(s) PMM0263;
253115	+	TSS_002948	1000	353	0	3	I	within gene(s) PMM0263;
253136	+	TSS_002950	1000	907	0	0	I	within gene(s) PMM0263;
253148	+	TSS_002953	1000	1242	0	13	I	within gene(s) PMM0263;
253169	+	TSS_002958	1000	6250	0	6	I	within gene(s) PMM0263;
253193	+	TSS_002963	1000	355	0	6	I	within gene(s) PMM0263;
253223	+	TSS_002970	1000	1469	0	12	I	within gene(s) PMM0263;
253244	+	TSS_002974	1000	834	0	15	I	within gene(s) PMM0263;
253265	+	TSS_002978	1000	951	0	12	I	within gene(s) PMM0263;
253271	-	TSS_019100	1000	403	0	7	Ai	antisense to gene(s) PMM0263;
253279	-	TSS_019101	1000	642	0	0	Ai	antisense to gene(s) PMM0263;
253286	+	TSS_002980	1000	348	0	0	I	within gene(s) PMM0263;
253289	-	TSS_019102	1000	632	0	5	Ai	antisense to gene(s) PMM0263;
253301	+	TSS_002982	1000	399	0	6	I	within gene(s) PMM0263;
253313	+	TSS_002985	1000	960	0	3	I	within gene(s) PMM0263;
253321	-	TSS_019105	1000	313	0	0	Ai	antisense to gene(s) PMM0263;
253328	+	TSS_002987	1000	4711	0	33	I	within gene(s) PMM0263;
253329	-	TSS_019106	1000	101	0	0	Ai	antisense to gene(s) PMM0263;
253357	-	TSS_019107	1000	182	0	0	Ai	antisense to gene(s) PMM0263;
253370	+	TSS_002996	1000	534	0	0	I	within gene(s) PMM0263;
253391	+	TSS_002998	1000	535	0	15	I	within gene(s) PMM0263;
253418	+	TSS_003003	1000	947	0	3	I	within gene(s) PMM0263;
253433	+	TSS_003006	1000	294	0	3	I	within gene(s) PMM0263;
253435	-	TSS_019108	1000	297	0	0	Ai	antisense to gene(s) PMM0263;
253451	-	TSS_019109	1000	181	0	1	Ai	antisense to gene(s) PMM0263;
253460	+	TSS_003007	1000	431	0	0	I	within gene(s) PMM0263;
253469	+	TSS_003008	1000	268	0	0	I	within gene(s) PMM0263;
253479	+	TSS_003009	1000	303	0	0	I	within gene(s) PMM0263;
253490	+	TSS_003011	1000	739	0	3	I	within gene(s) PMM0263;
253498	-	TSS_019111	1000	221	0	0	Ai	antisense to gene(s) PMM0263;
253511	+	TSS_003015	1000	614	0	18	I	within gene(s) PMM0263;
253529	+	TSS_003019	1000	2148	0	12	I	within gene(s) PMM0263;
253548	+	TSS_003025	1000	306	0	0	I	within gene(s) PMM0263;
253559	+	TSS_003026	1000	378	0	0	I	within gene(s) PMM0263;
253606	-	TSS_019113	1000	2669	0	0	Ai	antisense to gene(s) PMM0263;
253636	-	TSS_019114	1000	118	0	0	Ai	antisense to gene(s) PMM0263;
253889	+	TSS_003030	1000	153	0	12	I	within gene(s) PMM0263;
254003	-	TSS_019117	1000	11411	0	11	Ai	antisense to gene(s) PMM0263;
254043	-	TSS_019122	1000	112	0	0	Ai	antisense to gene(s) PMM0263;
254055	-	TSS_019123	1000	230	0	0	Ai	antisense to gene(s) PMM0263;
254183	+	TSS_003038	1000	519	0	5	I	within gene(s) PMM0264;
254564	-	TSS_019126	1000	113	0	0	Ai	antisense to gene(s) PMM0264;
255509	+	TSS_003046	1000	270	0	1	I	within gene(s) PMM0265;
255521	+	TSS_003050	1000	1199	0	21	I	within gene(s) PMM0265;
255551	+	TSS_003057	1000	174	0	0	I	within gene(s) PMM0265;
255566	+	TSS_003059	1000	171	0	1	I	within gene(s) PMM0265;
255617	+	TSS_003060	1000	103	0	0	I	within gene(s) PMM0265;

255644	+	TSS_003064	1000	217	0	5 I	within gene(s) PMM0265;
255659	+	TSS_003068	1000	207	0	10 I	within gene(s) PMM0265;
255683	+	TSS_003072	1000	182	0	9 I	within gene(s) PMM0265;
255746	+	TSS_003086	1000	280	0	42 I	within gene(s) PMM0265;
255779	+	TSS_003093	1000	125	0	27 I	within gene(s) PMM0265;
255812	+	TSS_003098	1000	125	0	1 I	within gene(s) PMM0265;
256772	+	TSS_003109	1000	109	0	0 Ai	antisense to gene(s) PMM0266;
257509	+	TSS_003113	1000	105	0	0 PAi	113nt upstream of gene PMM0267; antisense to gene(s) PMM0266;
258284	+	TSS_003116	1000	140	0	0 Ai	antisense to gene(s) PMM0268;
258297	+	TSS_003118	1000	175	0	1 Ai	antisense to gene(s) PMM0268;
258382	+	TSS_003120	1000	204	0	0 Ai	antisense to gene(s) PMM0268;
258478	-	TSS_019156	1000	372	0	2 I	within gene(s) PMM0268;
258496	-	TSS_019159	1000	116	0	12 I	within gene(s) PMM0268;
258539	+	TSS_003121	1000	350	0	0 Ai	antisense to gene(s) PMM0268;
258616	-	TSS_019169	1000	180	0	3 P	12nt upstream of gene PMM0268;
261874	+	TSS_003138	1000	110	0	1 IP	within gene(s) PMM0271; 74nt upstream of gene PMM0272;
261925	+	TSS_003141	1000	7930	0	4 P	23nt upstream of gene PMM0272;
263126	-	TSS_019190	1000	723	0	4 P	19nt upstream of gene PMM0273;
263533	-	TSS_019193	1000	107	0	3 I	within gene(s) PMM0274;
263543	+	TSS_003152	1000	596	0	1 Ai	antisense to gene(s) PMM0274;
264013	+	TSS_003154	1000	155	0	0 Ai	antisense to gene(s) PMM0274;
264561	-	TSS_019198	1000	197	0	0 P	135nt upstream of gene PMM0274;
264640	+	TSS_003156	1000	273	0	2 P	18nt upstream of gene PMM0275;
265763	+	TSS_003169	1000	142	0	1 I	within gene(s) PMM0276;
268849	+	TSS_003175	1000	118	0	0 IP	within gene(s) PMM0278; 175nt upstream of gene PMM0279;
269162	+	TSS_003179	1000	397	0	2 I	within gene(s) PMM0279;
271937	-	TSS_019246	1000	585	0	0 IP	within gene(s) PMM0282; 173nt upstream of gene PMM0281;
272579	-	TSS_019276	1000	103	0	1 P	12nt upstream of gene PMM0282;
273322	+	TSS_003196	1000	413	0	3 I	within gene(s) PMM0284;
273525	+	TSS_003199	1000	115	0	1 I	within gene(s) PMM0284;
274872	-	TSS_019284	1000	221	0	0 I	within gene(s) PMM0285;
274910	+	TSS_003202	1000	129	0	0 Ai	antisense to gene(s) PMM0285;
277117	+	TSS_003207	1000	496	0	0 PAi	54nt upstream of gene PMM0288; antisense to gene(s) PMM0287;
277159	+	TSS_003208	1000	265	0	0 P	12nt upstream of gene PMM0288;
279001	+	TSS_003238	1000	107	0	0 I	within gene(s) PMM0288;
280333	-	TSS_019307	1000	1791	0	2 IP	within gene(s) PMM0290; 149nt upstream of gene PMM0289;
281596	+	TSS_003253	1000	123	0	3 I	within gene(s) PMM0291;
283453	-	TSS_019340	1000	136	0	6 I	within gene(s) PMM0293;
283477	-	TSS_019343	1000	257	0	21 I	within gene(s) PMM0293;
283504	-	TSS_019350	1000	128	0	0 I	within gene(s) PMM0293;
283519	-	TSS_019352	1000	619	0	16 I	within gene(s) PMM0293;
283582	-	TSS_019363	1000	236	0	0 I	within gene(s) PMM0293;
283811	-	TSS_019374	1000	4145	0	0 IP	within gene(s) PMM0294; 52nt upstream of gene PMM0293;
283840	-	TSS_019375	1000	247	0	1 IP	within gene(s) PMM0294; 81nt upstream of gene PMM0293;
283853	-	TSS_019377	1000	397	0	0 IP	within gene(s) PMM0294; 94nt upstream of gene PMM0293;
283904	-	TSS_019379	1000	4086	0	4 IP	within gene(s) PMM0294; 145nt upstream of gene PMM0293;
284179	-	TSS_019386	1000	993	0	5 P	53nt upstream of gene PMM0294;
284302	-	TSS_019391	1000	6552	0	2 PAi	176nt upstream of gene PMM0294; antisense to gene(s) PMM0295;
284638	+	TSS_003270	1000	2165	0	2 I	within gene(s) PMM0296;
284851	+	TSS_003284	1000	442	0	7 I	within gene(s) PMM0296;
285147	+	TSS_003304	1000	186	0	4 I	within gene(s) PMM0296;
285370	+	TSS_003316	1000	158	0	5 I	within gene(s) PMM0296;
285545	-	TSS_019403	1000	143	0	1 Ai	antisense to gene(s) PMM0296;
285794	+	TSS_003322	1000	216	0	5 IP	within gene(s) PMM0297; 240nt upstream of gene PMM0298;
285816	+	TSS_003325	1000	384	0	4 IP	within gene(s) PMM0297; 218nt upstream of gene PMM0298;
285834	+	TSS_003329	1000	106	0	0 IP	within gene(s) PMM0297; 200nt upstream of gene PMM0298;
285844	+	TSS_003331	1000	591	0	3 IP	within gene(s) PMM0297; 190nt upstream of gene PMM0298;
285930	+	TSS_003339	1000	279	0	21 IP	within gene(s) PMM0297; 104nt upstream of gene PMM0298;
285945	+	TSS_003341	1000	463	0	0 IP	within gene(s) PMM0297; 89nt upstream of gene PMM0298;
285954	+	TSS_003343	1000	103	0	2 IP	within gene(s) PMM0297; 80nt upstream of gene PMM0298;
285978	+	TSS_003345	1000	105	0	0 IP	within gene(s) PMM0297; 56nt upstream of gene PMM0298;
285990	+	TSS_003348	1000	226	0	20 IP	within gene(s) PMM0297; 44nt upstream of gene PMM0298;
286014	+	TSS_003352	1000	214	0	0 IP	within gene(s) PMM0297; 20nt upstream of gene PMM0298;
286040	+	TSS_003353	1000	115	0	0 IP	within gene(s) PMM0298; 149nt upstream of gene PMM0299;
286056	+	TSS_003357	1000	735	0	24 IP	within gene(s) PMM0298; 133nt upstream of gene PMM0299;
286103	+	TSS_003364	1000	185	0	6 IP	within gene(s) PMM0298; 86nt upstream of gene PMM0299;
286115	+	TSS_003365	1000	201	0	0 IP	within gene(s) PMM0298; 74nt upstream of gene PMM0299;
286145	+	TSS_003366	1000	135	0	0 IP	within gene(s) PMM0298; 44nt upstream of gene PMM0299;
286157	+	TSS_003368	1000	230	0	7 IP	within gene(s) PMM0298; 32nt upstream of gene PMM0299;
286195	+	TSS_003372	1000	1001	0	41 IP	within gene(s) PMM0299; 123nt upstream of gene PMM0300;
286246	+	TSS_003386	1000	638	0	6 IP	within gene(s) PMM0299; 72nt upstream of gene PMM0300;
286270	+	TSS_003390	1000	1912	0	22 IP	within gene(s) PMM0299; 48nt upstream of gene PMM0300;
286291	+	TSS_003397	1000	432	0	4 IP	within gene(s) PMM0299; 27nt upstream of gene PMM0300;
286319	+	TSS_003400	1000	491	0	7 I	within gene(s) PMM0300;
286339	+	TSS_003403	1000	1134	0	15 I	within gene(s) PMM0300;
286366	+	TSS_003411	1000	190	0	10 I	within gene(s) PMM0300;
286530	-	TSS_019410	1000	144	0	0 Ad	antisense to gene(s) PMM0300 (19nt downstream);

286877	+	TSS_003419	1000	295	0	1	Ai	antisense to gene(s) PMM0301;
287552	-	TSS_019433	1000	1090	0	0	PAi	83nt upstream of gene PMM0301; antisense to gene(s) PMM0302;
287702	-	TSS_019435	1000	653	0	4	PAi	233nt upstream of gene PMM0301; antisense to gene(s) PMM0302;
290102	+	TSS_003431	1000	267	0	0	Ai	antisense to gene(s) PMM0303;
290195	+	TSS_003432	1000	414	0	0	Ai	antisense to gene(s) PMM0303;
291317	-	TSS_019442	1000	261	0	0	I	within gene(s) PMM0304;
291416	-	TSS_019443	1000	122	0	0	I	within gene(s) PMM0304;
293682	+	TSS_003437	1000	866	0	6	P	28nt upstream of gene PMM0305;
294064	+	TSS_003444	1000	125	0	0	I	within gene(s) PMM0305;
294128	-	TSS_019453	1000	433	0	1	Ai	antisense to gene(s) PMM0305;
297920	-	TSS_019469	1000	163	0	0	IP	within gene(s) PMM0311; 144nt upstream of gene PMM0310;
298142	-	TSS_019498	1000	247	0	33	I	within gene(s) PMM0311;
298181	-	TSS_019506	1000	335	0	6	I	within gene(s) PMM0311;
298403	-	TSS_019519	1000	123	0	9	I	within gene(s) PMM0311;
298704	+	TSS_003458	1000	111	0	0	Ai	antisense to gene(s) PMM0311;
298904	-	TSS_019554	1000	119	0	3	I	within gene(s) PMM0311;
299042	-	TSS_019563	1000	7848	0	2	P	18nt upstream of gene PMM0311;
299333	-	TSS_019566	1000	125	0	6	I	within gene(s) PMM0312;
299355	-	TSS_019570	1000	110	0	7	I	within gene(s) PMM0312;
299500	-	TSS_019588	1000	153	0	0	I	within gene(s) PMM0312;
299698	-	TSS_019597	1000	107	0	0	I	within gene(s) PMM0312;
299713	-	TSS_019599	1000	256	0	24	I	within gene(s) PMM0312;
299749	-	TSS_019607	1000	121	0	6	I	within gene(s) PMM0312;
299836	-	TSS_019615	1000	172	0	3	I	within gene(s) PMM0312;
299850	+	TSS_003472	1000	126	0	1	Ai	antisense to gene(s) PMM0312;
299950	-	TSS_019620	1000	147	0	3	I	within gene(s) PMM0312;
300325	-	TSS_019635	1000	6918	0	10	P	75nt upstream of gene PMM0312;
300858	-	TSS_019645	1000	5092	0	12	P	21nt upstream of gene PMM0313;
301015	+	TSS_003473	1000	112	0	0	Ai	antisense to gene(s) PMM0314;
301023	+	TSS_003474	1000	370	0	0	Ai	antisense to gene(s) PMM0314;
301120	-	TSS_019649	1000	115	0	12	IP	within gene(s) PMM0315; 81nt upstream of gene PMM0314;
301160	+	TSS_003479	1000	158	0	0	Ai	antisense to gene(s) PMM0315;
301180	-	TSS_019664	1000	802	0	30	IP	within gene(s) PMM0315; 141nt upstream of gene PMM0314;
301210	-	TSS_019670	1000	248	0	12	IP	within gene(s) PMM0315; 171nt upstream of gene PMM0314;
301222	-	TSS_019672	1000	342	0	6	IP	within gene(s) PMM0315; 183nt upstream of gene PMM0314;
301264	-	TSS_019680	1000	698	0	12	IP	within gene(s) PMM0315; 225nt upstream of gene PMM0314;
301276	-	TSS_019684	1000	2152	0	40	IP	within gene(s) PMM0315; 237nt upstream of gene PMM0314;
301336	-	TSS_019699	1000	751	0	18	I	within gene(s) PMM0315;
301351	+	TSS_003481	1000	178	0	0	Ai	antisense to gene(s) PMM0315;
301399	-	TSS_019709	1000	873	0	23	I	within gene(s) PMM0315;
301441	-	TSS_019715	1000	560	0	13	I	within gene(s) PMM0315;
301474	-	TSS_019718	1000	179	3.20E-10	0	I	within gene(s) PMM0315;
301483	-	TSS_019719	1000	242	0	0	I	within gene(s) PMM0315;
301501	-	TSS_019721	1000	2316	0	18	I	within gene(s) PMM0315;
301534	-	TSS_019731	1000	372	0	6	I	within gene(s) PMM0315;
301547	+	TSS_003484	1000	150	0	0	Ai	antisense to gene(s) PMM0315;
301567	-	TSS_019734	1000	1236	0	6	I	within gene(s) PMM0315;
301578	-	TSS_019736	1000	317	0	4	I	within gene(s) PMM0315;
301594	-	TSS_019739	1000	178	3.50E-07	0	I	within gene(s) PMM0315;
301607	+	TSS_003485	1000	121	0	0	Ai	antisense to gene(s) PMM0315;
301612	-	TSS_019743	1000	958	0	18	I	within gene(s) PMM0315;
301642	-	TSS_019751	1000	1196	0	27	I	within gene(s) PMM0315;
301669	-	TSS_019759	1000	218	0	9	I	within gene(s) PMM0315;
301701	-	TSS_019766	1000	1541	0	51	I	within gene(s) PMM0315;
301721	+	TSS_003488	1000	287	0	0	Ai	antisense to gene(s) PMM0315;
301735	+	TSS_003489	1000	200	0	0	Ai	antisense to gene(s) PMM0315;
301756	-	TSS_019779	1000	216	0	0	I	within gene(s) PMM0315;
301780	+	TSS_003493	1000	111	0	0	Ai	antisense to gene(s) PMM0315;
301782	-	TSS_019783	1000	434	0	18	I	within gene(s) PMM0315;
301810	-	TSS_019786	1000	300	0	3	I	within gene(s) PMM0315;
301822	-	TSS_019788	1000	194	4.70E-12	0	I	within gene(s) PMM0315;
301834	-	TSS_019791	1000	837	0	27	I	within gene(s) PMM0315;
301897	-	TSS_019810	1000	1081	0	61	I	within gene(s) PMM0315;
301916	+	TSS_003496	1000	904	0	3	Ai	antisense to gene(s) PMM0315;
301936	-	TSS_019821	1000	604	0	12	I	within gene(s) PMM0315;
301937	+	TSS_003497	1000	661	0	0	Ai	antisense to gene(s) PMM0315;
301964	+	TSS_003499	1000	142	0	0	Ai	antisense to gene(s) PMM0315;
301981	-	TSS_019825	1000	376	0	0	I	within gene(s) PMM0315;
302023	-	TSS_019833	1000	1842	0	21	I	within gene(s) PMM0315;
302044	-	TSS_019834	1000	342	0	6	I	within gene(s) PMM0315;
302062	-	TSS_019837	1000	1393	0	6	I	within gene(s) PMM0315;
302080	-	TSS_019839	1000	950	0	11	I	within gene(s) PMM0315;
302107	-	TSS_019844	1000	1267	0	41	I	within gene(s) PMM0315;
302114	+	TSS_003500	1000	162	0	0	Ai	antisense to gene(s) PMM0315;
302122	+	TSS_003502	1000	642	0	1	Ai	antisense to gene(s) PMM0315;
302152	-	TSS_019854	1000	375	0	0	I	within gene(s) PMM0315;
302182	-	TSS_019861	1000	3270	0	40	I	within gene(s) PMM0315;

302221	-	TSS_019874	1000	1580	0	3	I	within gene(s) PMM0315;
302233	-	TSS_019875	1000	464	0	32	I	within gene(s) PMM0315;
302272	-	TSS_019885	1000	1094	0	0	I	within gene(s) PMM0315;
302293	-	TSS_019886	1000	285	0	0	I	within gene(s) PMM0315;
302302	-	TSS_019887	1000	143	4.00E-07	0	I	within gene(s) PMM0315;
302317	-	TSS_019888	1000	171	0	0	I	within gene(s) PMM0315;
302320	+	TSS_003506	1000	105	0	0	Ai	antisense to gene(s) PMM0315;
302327	+	TSS_003507	1000	188	0	0	Ai	antisense to gene(s) PMM0315;
302329	-	TSS_019891	1000	567	0	3	I	within gene(s) PMM0315;
302338	-	TSS_019892	1000	1067	0	9	I	within gene(s) PMM0315;
302364	-	TSS_019896	1000	177	0	7	I	within gene(s) PMM0315;
302368	+	TSS_003508	1000	113	0	0	Ai	antisense to gene(s) PMM0315;
302385	-	TSS_019900	1000	252	0	4	I	within gene(s) PMM0315;
302401	-	TSS_019903	1000	174	0	3	I	within gene(s) PMM0315;
302418	-	TSS_019904	1000	609	0	1	I	within gene(s) PMM0315;
302449	-	TSS_019910	1000	260	0	18	I	within gene(s) PMM0315;
302461	-	TSS_019912	1000	885	0	6	I	within gene(s) PMM0315;
302479	-	TSS_019916	1000	234	0	3	I	within gene(s) PMM0315;
302497	-	TSS_019918	1000	5868	0	4	I	within gene(s) PMM0315;
302539	-	TSS_019925	1000	613	0	21	I	within gene(s) PMM0315;
302557	-	TSS_019929	1000	667	0	0	I	within gene(s) PMM0315;
302590	-	TSS_019931	1000	4562	0	2	P	3nt upstream of gene PMM0315;
302980	+	TSS_003519	1000	146	0	18	I	within gene(s) PMM0316;
303244	-	TSS_019936	1000	191	0	6	O	-
303256	+	TSS_003525	1000	5351	0	18	P	24nt upstream of gene PMM0317;
305403	-	TSS_019947	1000	281	0	2	I	within gene(s) PMM0320;
305442	-	TSS_019949	1000	218	0	0	I	within gene(s) PMM0320;
305463	-	TSS_019953	1000	186	0	7	I	within gene(s) PMM0320;
305727	-	TSS_019960	1000	905	0	4	IP	within gene(s) PMM0321; 174nt upstream of gene PMM0320;
306386	-	TSS_019968	1000	1408	0	2	P	15nt upstream of gene PMM0321;
308744	-	TSS_019984	1000	132	0	0	I	within gene(s) PMM0324;
308747	+	TSS_003555	1000	238	0	0	Ai	antisense to gene(s) PMM0324;
308758	+	TSS_003557	1000	11051	0	3	Ai	antisense to gene(s) PMM0324;
308787	-	TSS_019986	1000	167	0	0	I	within gene(s) PMM0324;
309008	+	TSS_003563	1000	389	0	0	Ai	antisense to gene(s) PMM0324;
309313	+	TSS_003566	1000	104	0	9	Ai	antisense to gene(s) PMM0324;
309678	-	TSS_020025	1000	156	0	0	I	within gene(s) PMM0324;
309757	-	TSS_020027	1000	314	0	0	P	22nt upstream of gene PMM0324;
309902	-	TSS_020029	1000	669	0	0	PAi	167nt upstream of gene PMM0324; antisense to gene(s) PMM0325;
309920	+	TSS_003573	1000	213	0	3	I	within gene(s) PMM0325;
309929	-	TSS_020031	1000	8113	0	2	PAi	194nt upstream of gene PMM0324; antisense to gene(s) PMM0325;
309975	-	TSS_020033	1000	216	0	4	PAi	240nt upstream of gene PMM0324; antisense to gene(s) PMM0325;
310004	+	TSS_003583	1000	227	0	12	I	within gene(s) PMM0325;
310023	+	TSS_003586	1000	995	0	3	I	within gene(s) PMM0325;
310053	+	TSS_003592	1000	132	0	33	I	within gene(s) PMM0325;
310100	+	TSS_003597	1000	358	0	9	I	within gene(s) PMM0325;
310184	+	TSS_003606	1000	148	0	9	I	within gene(s) PMM0325;
310271	+	TSS_003614	1000	664	0	8	IP	within gene(s) PMM0325; 234nt upstream of gene PMM0326;
310400	-	TSS_020039	1000	108	0	0	Ai	antisense to gene(s) PMM0325;
310445	+	TSS_003625	1000	267	0	12	IP	within gene(s) PMM0325; 60nt upstream of gene PMM0326;
310480	+	TSS_003626	1000	573	0	2	P	25nt upstream of gene PMM0326;
310538	+	TSS_003628	1000	574	0	0	I	within gene(s) PMM0326;
310550	+	TSS_003630	1000	189	0	2	I	within gene(s) PMM0326;
310574	+	TSS_003638	1000	553	0	17	I	within gene(s) PMM0326;
310592	+	TSS_003644	1000	1370	0	18	I	within gene(s) PMM0326;
310622	+	TSS_003648	1000	697	0	9	I	within gene(s) PMM0326;
310655	+	TSS_003653	1000	466	0	48	I	within gene(s) PMM0326;
310715	+	TSS_003665	1000	183	0	0	I	within gene(s) PMM0326;
310733	+	TSS_003666	1000	117	0	0	I	within gene(s) PMM0326;
310880	+	TSS_003675	1000	408	0	30	I	within gene(s) PMM0326;
311306	+	TSS_003689	1000	178	0	0	Ai	antisense to gene(s) PMM0327;
311725	+	TSS_003693	1000	109	0	0	Ai	antisense to gene(s) PMM0327;
311776	-	TSS_020078	1000	223	0	10	I	within gene(s) PMM0327;
312157	-	TSS_020093	1000	209	0	3	I	within gene(s) PMM0327;
312178	-	TSS_020096	1000	116	0	18	I	within gene(s) PMM0327;
312446	-	TSS_020110	1000	2453	0	3	P	16nt upstream of gene PMM0327;
312554	+	TSS_003698	1000	3362	0	0	O	-
312873	-	TSS_020113	1000	916	0	1	O	-
313101	+	TSS_003712	1000	385	0	2	O	-
313132	-	TSS_020121	1000	195	0	0	O	-
313374	-	TSS_020123	1000	202	0	1	O	-
313535	-	TSS_020126	1000	285	0	6	O	-
314529	-	TSS_020134	1000	229	0	1	O	-
314912	+	TSS_003779	1000	305	0	0	O	-
314959	+	TSS_003780	1000	380	0	0	O	-
319201	-	TSS_020166	1000	173	0	9	IP	within gene(s) PMM0329; 148nt upstream of gene PMM0328;
319230	-	TSS_020173	1000	273	0	18	IP	within gene(s) PMM0329; 177nt upstream of gene PMM0328;

319252	-	TSS_020178	1000	102	0	3	IP	within gene(s) PMM0329; 199nt upstream of gene PMM0328;
319267	-	TSS_020181	1000	572	0	0	P	0nt upstream of gene PMM0329;
320607	+	TSS_003866	1000	176	0	0	Ai	antisense to gene(s) PMM0331;
322362	+	TSS_003870	1000	283	0	5	P	0nt upstream of gene PMM0333;
323620	-	TSS_020198	1000	1356	0	5	P	21nt upstream of gene PMM0334;
324251	-	TSS_020201	1000	11258	0	4	O	-
324470	-	TSS_020206	1000	1511	0	2	O	-
324585	+	TSS_003886	1000	146	0	6	P	17nt upstream of gene PMM0335;
324964	+	TSS_003891	1000	1400	0	1	I	within gene(s) PMM0335;
325107	+	TSS_003895	1000	117	0	4	Ai	antisense to gene(s) PMM0336;
325272	-	TSS_020233	1000	426	0	51	I	within gene(s) PMM0336;
325326	-	TSS_020247	1000	216	0	18	I	within gene(s) PMM0336;
325368	-	TSS_020255	1000	468	0	36	I	within gene(s) PMM0336;
325410	-	TSS_020264	1000	318	0	18	I	within gene(s) PMM0336;
325431	-	TSS_020269	1000	120	0	2	I	within gene(s) PMM0336;
325449	-	TSS_020272	1000	155	0	6	I	within gene(s) PMM0336;
325485	-	TSS_020278	1000	363	0	60	I	within gene(s) PMM0336;
325557	-	TSS_020292	1000	40615	0	3	P	15nt upstream of gene PMM0336;
325983	+	TSS_003902	1000	173	0	2	O	-
326260	-	TSS_020305	1000	102	0	20	I	within gene(s) PMM0337;
326308	-	TSS_020315	1000	103	0	3	I	within gene(s) PMM0337;
326449	-	TSS_020332	1000	1091	0	1	P	15nt upstream of gene PMM0337;
326712	+	TSS_003908	1000	2083	0	2	O	-
327357	-	TSS_020338	1000	127	0	22	O	-
327387	-	TSS_020344	1000	153	0	3	O	-
327570	-	TSS_020357	1000	9781	0	2	O	-
327681	+	TSS_003920	1000	418	0	3	Ai	antisense to gene(s) PMM0339;
327887	+	TSS_003925	1000	103	0	0	Ai	antisense to gene(s) PMM0339;
328681	-	TSS_020363	1000	296	0	6	I	within gene(s) PMM0339;
329311	-	TSS_020366	1000	101	0	2	P	120nt upstream of gene PMM0339;
329348	-	TSS_020369	1000	1193	0	1	P	157nt upstream of gene PMM0339;
330221	-	TSS_020371	1000	489	0	1	P	15nt upstream of gene PMM0341;
330306	+	TSS_003933	1000	1628	0	1	P	35nt upstream of gene PMM0342;
330978	-	TSS_020374	1000	536	0	3	O	-
331018	-	TSS_020377	1000	452	0	0	O	-
331354	-	TSS_020390	1000	105	0	0	P	32nt upstream of gene PMM0343;
332301	+	TSS_003941	1000	184	0	6	P	16nt upstream of gene PMM0345;
332821	+	TSS_003947	1000	2422	0	3	I	within gene(s) PMM0346;
332939	-	TSS_020392	1000	167	0	1	Ai	antisense to gene(s) PMM0346;
332983	-	TSS_020393	1000	145	0	0	Ai	antisense to gene(s) PMM0346;
333569	-	TSS_020398	1000	522	0	0	P	108nt upstream of gene PMM0347;
333712	+	TSS_003950	1000	132	0	0	Ai	antisense to gene(s) PMM0348;
333733	+	TSS_003951	1000	118	0	0	Ai	antisense to gene(s) PMM0348;
336616	+	TSS_003958	1000	112	0	0	Ad	antisense to gene(s) PMM0353 (24nt downstream);
339021	+	TSS_003965	1000	466	0	1	P	18nt upstream of gene PMM0355;
339316	-	TSS_020409	1000	335	0	0	Ai	antisense to gene(s) PMM0355;
340326	+	TSS_003969	1000	470	0	2	I	within gene(s) PMM0356;
343466	+	TSS_003981	1000	168	0	6	O	-
345398	+	TSS_003991	1000	192	0	3	P	22nt upstream of gene PMM0363;
346829	+	TSS_003996	1000	178	0	3	O	-
347280	+	TSS_004000	1000	12100	0	4	P	16nt upstream of gene PMM0364;
347314	+	TSS_004002	1000	102	0	9	I	within gene(s) PMM0364;
348066	-	TSS_020438	1000	4244	0	2	P	21nt upstream of gene PMM0365;
348206	+	TSS_004014	1000	669	0	5	P	17nt upstream of gene PMM0366;
348397	+	TSS_004024	1000	306	0	1	I	within gene(s) PMM0366;
348935	+	TSS_004030	1000	599	0	1	P	33nt upstream of gene PMM0367;
349333	-	TSS_020444	1000	292	0	3	Ai	antisense to gene(s) PMM0367;
349634	-	TSS_020449	1000	344	0	0	I	within gene(s) PMM0368;
350069	-	TSS_020452	1000	1135	0	2	O	-
350388	+	TSS_004034	1000	224	0	1	O	-
350523	-	TSS_020456	1000	45543	0	4	O	-
351169	-	TSS_020463	1000	16988	0	6	O	-
352103	-	TSS_020475	1000	798	0	2	O	-
352188	+	TSS_004042	1000	3081	0	2	P	7nt upstream of gene PMM0369;
352957	+	TSS_004045	1000	1183	0	1	P	18nt upstream of gene PMM0370;
353347	+	TSS_004087	1000	165	0	24	I	within gene(s) PMM0370;
353380	+	TSS_004095	1000	293	0	15	I	within gene(s) PMM0370;
353416	+	TSS_004100	1000	182	0	0	I	within gene(s) PMM0370;
353434	+	TSS_004104	1000	136	0	8	I	within gene(s) PMM0370;
353529	-	TSS_020479	1000	281	0	0	Ai	antisense to gene(s) PMM0370;
353614	+	TSS_004116	1000	105	0	12	I	within gene(s) PMM0370;
353732	-	TSS_020480	1000	471	0	1	Ai	antisense to gene(s) PMM0370;
353827	+	TSS_004141	1000	106	0	36	I	within gene(s) PMM0370;
353890	+	TSS_004154	1000	689	0	15	I	within gene(s) PMM0370;
353932	+	TSS_004161	1000	124	0	6	I	within gene(s) PMM0370;
353959	+	TSS_004165	1000	207	0	6	I	within gene(s) PMM0370;
353992	+	TSS_004171	1000	361	0	15	I	within gene(s) PMM0370;

354022	+	TSS_004174	1000	120	0	0	I	within gene(s) PMM0370;
354046	+	TSS_004176	1000	152	0	0	I	within gene(s) PMM0370;
354058	+	TSS_004177	1000	132	0	0	I	within gene(s) PMM0370;
354088	+	TSS_004179	1000	527	0	9	I	within gene(s) PMM0370;
354139	+	TSS_004189	1000	526	0	19	I	within gene(s) PMM0370;
354154	+	TSS_004193	1000	116	0	3	I	within gene(s) PMM0370;
354178	+	TSS_004195	1000	925	0	9	I	within gene(s) PMM0370;
354199	+	TSS_004200	1000	121	0	3	I	within gene(s) PMM0370;
354223	+	TSS_004201	1000	105	0	0	I	within gene(s) PMM0370;
354238	+	TSS_004203	1000	168	0	9	I	within gene(s) PMM0370;
354259	+	TSS_004207	1000	237	0	12	I	within gene(s) PMM0370;
354346	+	TSS_004215	1000	204	0	9	I	within gene(s) PMM0370;
354423	-	TSS_020489	1000	142	0	2	Ai	antisense to gene(s) PMM0370;
354427	+	TSS_004232	1000	470	0	69	I	within gene(s) PMM0370;
354439	-	TSS_020492	1000	159	0	1	Ai	antisense to gene(s) PMM0370;
355412	-	TSS_020497	1000	395	0	0	Ai	antisense to gene(s) PMM0371;
356060	-	TSS_020499	1000	277	0	0	Ai	antisense to gene(s) PMM0372;
356326	+	TSS_004264	1000	187	0	2	IP	within gene(s) PMM0372; 51nt upstream of gene PMM0373;
358183	-	TSS_020511	1000	1534	0	2	P	16nt upstream of gene PMM0377;
358480	-	TSS_020515	1000	104	0	0	I	within gene(s) PMM0378;
358512	-	TSS_020518	1000	15213	0	2	P	22nt upstream of gene PMM0378;
358753	-	TSS_020521	1000	5396	0	2	O	-
359627	-	TSS_020524	1000	908	0	1	O	-
359991	-	TSS_020529	1000	1993	0	3	P	20nt upstream of gene PMM0379;
361648	+	TSS_004279	1000	240	0	0	O	-
361663	+	TSS_004280	1000	306	0	0	O	-
362873	-	TSS_020537	1000	1252	0	4	P	140nt upstream of gene PMM0383;
366442	-	TSS_020561	1000	464	0	7	O	-
366522	-	TSS_020566	1000	1326	0	1	O	-
367091	-	TSS_020570	1000	178	0	4	I	within gene(s) PMM0386;
367935	-	TSS_020573	1000	456	0	0	O	-
368234	-	TSS_020574	1000	107	0	0	O	-
368274	+	TSS_004307	1000	939	0	2	O	-
368514	-	TSS_020577	1000	295	0	4	O	-
370969	+	TSS_004323	1000	2308	0	3	P	24nt upstream of gene PMM0391;
374270	+	TSS_004334	1000	1057	0	0	P	97nt upstream of gene PMM0395;
374324	+	TSS_004335	1000	286	0	1	P	43nt upstream of gene PMM0395;
374511	+	TSS_004348	1000	137	0	27	I	within gene(s) PMM0395;
374559	+	TSS_004360	1000	193	0	16	I	within gene(s) PMM0395;
374631	+	TSS_004370	1000	168	0	12	I	within gene(s) PMM0395;
374685	+	TSS_004376	1000	243	0	6	I	within gene(s) PMM0395;
374701	+	TSS_004380	1000	332	0	36	IP	within gene(s) PMM0395; 249nt upstream of gene PMM0396;
374754	+	TSS_004394	1000	322	0	36	IP	within gene(s) PMM0395; 196nt upstream of gene PMM0396;
374796	+	TSS_004406	1000	223	0	9	IP	within gene(s) PMM0395; 154nt upstream of gene PMM0396;
374826	+	TSS_004409	1000	162	0	6	IP	within gene(s) PMM0395; 124nt upstream of gene PMM0396;
376105	+	TSS_004421	1000	132	0	1	Ai	antisense to gene(s) PMM0397;
376934	-	TSS_020603	1000	106	0	0	P	15nt upstream of gene PMM0397;
377675	-	TSS_020606	1000	229	0	1	I	within gene(s) PMM0398;
379061	-	TSS_020612	1000	134	0	2	P	24nt upstream of gene PMM0400;
380710	-	TSS_020616	1000	200	0	0	Ai	antisense to gene(s) PMM0402;
381726	+	TSS_004435	1000	3605	0	2	P	14nt upstream of gene PMM0403;
383390	+	TSS_004444	1000	112	0	0	O	-
383585	+	TSS_004447	1000	8797	0	6	P	85nt upstream of gene PMM0405;
383859	+	TSS_004479	1000	105	0	4	I	within gene(s) PMM0405;
383907	+	TSS_004483	1000	105	0	0	I	within gene(s) PMM0405;
383961	+	TSS_004490	1000	132	0	24	I	within gene(s) PMM0405;
384006	+	TSS_004494	1000	174	0	24	I	within gene(s) PMM0405;
384030	+	TSS_004500	1000	154	0	17	I	within gene(s) PMM0405;
384035	-	TSS_020623	1000	479	0	1	Ai	antisense to gene(s) PMM0405;
384162	+	TSS_004529	1000	768	0	46	I	within gene(s) PMM0405;
384195	+	TSS_004538	1000	356	0	29	I	within gene(s) PMM0405;
384222	+	TSS_004545	1000	183	0	12	I	within gene(s) PMM0405;
384243	+	TSS_004548	1000	113	0	15	I	within gene(s) PMM0405;
384519	+	TSS_004563	1000	667	0	24	I	within gene(s) PMM0405;
384720	+	TSS_004590	1000	611	0	15	I	within gene(s) PMM0405;
384729	+	TSS_004591	1000	174	0	0	I	within gene(s) PMM0405;
385716	+	TSS_004609	1000	303	0	0	I	within gene(s) PMM0406;
386282	+	TSS_004613	1000	342	0	0	Ai	antisense to gene(s) PMM0407;
386365	-	TSS_020637	1000	202	0	18	I	within gene(s) PMM0407;
386383	-	TSS_020642	1000	562	0	18	I	within gene(s) PMM0407;
386402	+	TSS_004616	1000	303	0	0	Ai	antisense to gene(s) PMM0407;
386404	-	TSS_020647	1000	346	0	9	I	within gene(s) PMM0407;
386467	-	TSS_020665	1000	2551	0	42	I	within gene(s) PMM0407;
386503	-	TSS_020673	1000	985	0	24	I	within gene(s) PMM0407;
386514	+	TSS_004619	1000	598	0	4	Ai	antisense to gene(s) PMM0407;
386527	-	TSS_020675	1000	687	0	15	I	within gene(s) PMM0407;
386551	-	TSS_020680	1000	653	0	0	I	within gene(s) PMM0407;

386578	-	TSS_020682	1000	282	0	12	I	within gene(s) PMM0407;
386603	+	TSS_004620	1000	1801	0	1	Ai	antisense to gene(s) PMM0407;
386611	-	TSS_020690	1000	889	0	60	I	within gene(s) PMM0407;
386615	+	TSS_004622	1000	134	0	0	Ai	antisense to gene(s) PMM0407;
386680	-	TSS_020706	1000	653	0	12	I	within gene(s) PMM0407;
386701	-	TSS_020709	1000	1985	0	78	I	within gene(s) PMM0407;
386777	+	TSS_004625	1000	293	0	7	Ai	antisense to gene(s) PMM0407;
386815	-	TSS_020745	1000	1361	0	56	I	within gene(s) PMM0407;
386845	-	TSS_020751	1000	257	0	0	I	within gene(s) PMM0407;
386861	+	TSS_004627	1000	331	0	13	Ai	antisense to gene(s) PMM0407;
386872	-	TSS_020754	1000	1097	0	24	I	within gene(s) PMM0407;
386989	-	TSS_020792	1000	737	0	102	I	within gene(s) PMM0407;
387013	-	TSS_020796	1000	319	0	0	I	within gene(s) PMM0407;
387022	-	TSS_020797	1000	707	0	0	I	within gene(s) PMM0407;
387042	-	TSS_020802	1000	1216	0	27	I	within gene(s) PMM0407;
387082	-	TSS_020811	1000	535	0	12	I	within gene(s) PMM0407;
387097	-	TSS_020812	1000	898	0	0	I	within gene(s) PMM0407;
387112	-	TSS_020813	1000	1115	0	0	I	within gene(s) PMM0407;
387124	-	TSS_020814	1000	320	0	0	I	within gene(s) PMM0407;
387133	-	TSS_020815	1000	1133	0	6	I	within gene(s) PMM0407;
387151	-	TSS_020817	1000	479	0	0	I	within gene(s) PMM0407;
387174	-	TSS_020819	1000	72622	0	11	P	17nt upstream of gene PMM0407;
390259	+	TSS_004647	1000	126	0	3	Ai	antisense to gene(s) PMM0410;
390555	-	TSS_020850	1000	541	0	1	I	within gene(s) PMM0410;
390616	-	TSS_020851	1000	774	0	1	P	55nt upstream of gene PMM0410;
390631	+	TSS_004652	1000	227	0	3	P	26nt upstream of gene PMM0411;
392813	+	TSS_004662	1000	458	0	2	P	15nt upstream of gene PMM0414;
395057	+	TSS_004666	1000	114	0	0	Ai	antisense to gene(s) PMM0416;
395539	-	TSS_020873	1000	1607	0	2	P	16nt upstream of gene PMM0416;
396587	+	TSS_004671	1000	138	0	6	P	42nt upstream of gene PMM0419;
398147	+	TSS_004680	1000	1163	0	2	P	32nt upstream of gene PMM0420;
398719	+	TSS_004685	1000	340	0	3	I	within gene(s) PMM0420;
401612	-	TSS_020889	1000	658	0	0	P	18nt upstream of gene PMM0422;
401747	+	TSS_004705	1000	6439	0	3	O	-
401864	-	TSS_020893	1000	193	0	1	O	-
402007	-	TSS_020894	1000	244	0	0	O	-
405550	+	TSS_004717	1000	187	0	1	I	within gene(s) PMM0426;
408258	-	TSS_020902	1000	144	0	1	IP	within gene(s) PMM0428; 237nt upstream of gene PMM0427;
409230	-	TSS_020919	1000	370	0	3	P	20nt upstream of gene PMM0429;
409244	+	TSS_004728	1000	122	0	0	P	26nt upstream of gene PMM0430;
409562	-	TSS_020921	1000	272	0	0	Ai	antisense to gene(s) PMM0430;
412935	+	TSS_004741	1000	252	0	8	Ai	antisense to gene(s) PMM0435;
413484	+	TSS_004745	1000	551	0	3	Ai	antisense to gene(s) PMM0435;
414354	+	TSS_004750	1000	215	0	0	P	130nt upstream of gene PMM0436;
414359	-	TSS_020957	1000	1260	0	2	P	44nt upstream of gene PMM0435;
414382	+	TSS_004751	1000	896	0	2	P	102nt upstream of gene PMM0436;
414424	-	TSS_020959	1000	207	0	4	P	109nt upstream of gene PMM0435;
417856	+	TSS_004817	1000	108	0	0	I	within gene(s) PMM0438;
419521	-	TSS_020971	1000	285	0	1	Ai	antisense to gene(s) PMM0440;
421671	-	TSS_020977	1000	111	0	0	P	22nt upstream of gene PMM0441;
422795	-	TSS_020984	1000	1028	0	6	P	16nt upstream of gene PMM0443;
423632	-	TSS_020999	1000	222	0	12	IP	within gene(s) PMM0445; 153nt upstream of gene PMM0444;
423797	-	TSS_021006	1000	280	0	6	I	within gene(s) PMM0445;
423830	-	TSS_021009	1000	385	0	0	I	within gene(s) PMM0445;
423865	+	TSS_004841	1000	189	0	1	Ai	antisense to gene(s) PMM0445;
423944	-	TSS_021014	1000	417	0	6	I	within gene(s) PMM0445;
424277	-	TSS_021028	1000	105	0	3	I	within gene(s) PMM0445;
424676	+	TSS_004849	1000	180	0	0	Ai	antisense to gene(s) PMM0445;
424706	-	TSS_021052	1000	477	0	3	I	within gene(s) PMM0445;
424756	-	TSS_021055	1000	179	0	0	I	within gene(s) PMM0445;
424769	-	TSS_021056	1000	195	0	0	I	within gene(s) PMM0445;
424790	-	TSS_021060	1000	565	0	21	I	within gene(s) PMM0445;
424829	-	TSS_021066	1000	171	0	1	I	within gene(s) PMM0445;
424853	-	TSS_021068	1000	141	0	3	I	within gene(s) PMM0445;
424880	-	TSS_021071	1000	1179	0	5	I	within gene(s) PMM0445;
424937	-	TSS_021076	1000	101	0	1	I	within gene(s) PMM0445;
425395	-	TSS_021095	1000	654	0	0	I	within gene(s) PMM0446;
425512	-	TSS_021098	1000	184	0	6	I	within gene(s) PMM0446;
425629	-	TSS_021113	1000	191	0	3	I	within gene(s) PMM0446;
425666	-	TSS_021117	1000	117	0	0	I	within gene(s) PMM0446;
426162	+	TSS_004855	1000	1255	0	3	P	8nt upstream of gene PMM0447;
427201	+	TSS_004882	1000	292	0	30	I	within gene(s) PMM0448;
427279	+	TSS_004887	1000	355	0	4	I	within gene(s) PMM0448;
427309	+	TSS_004890	1000	152	0	3	I	within gene(s) PMM0448;
427558	+	TSS_004894	1000	272	0	3	I	within gene(s) PMM0448;
427588	+	TSS_004898	1000	326	0	4	I	within gene(s) PMM0448;
427609	+	TSS_004901	1000	148	0	0	I	within gene(s) PMM0448;

427696	+	TSS_004905	1000	351	0	1	I	within gene(s) PMM0448;
427762	+	TSS_004910	1000	142	0	3	I	within gene(s) PMM0448;
429348	+	TSS_004917	1000	225	0	1	I	within gene(s) PMM0450;
429366	+	TSS_004919	1000	143	0	3	I	within gene(s) PMM0450;
429988	-	TSS_021136	1000	158	0	1	Ai	antisense to gene(s) PMM0450;
430126	+	TSS_004927	1000	426	0	6	I	within gene(s) PMM0451;
430701	+	TSS_004936	1000	124	0	0	Ai	antisense to gene(s) PMM0452;
431182	-	TSS_021161	1000	227	0	30	I	within gene(s) PMM0452;
431239	-	TSS_021175	1000	103	0	6	I	within gene(s) PMM0452;
431251	-	TSS_021177	1000	114	0	1	I	within gene(s) PMM0452;
431275	-	TSS_021179	1000	118	0	13	I	within gene(s) PMM0452;
431412	+	TSS_004945	1000	136	0	1	Ai	antisense to gene(s) PMM0452;
431485	-	TSS_021204	1000	141	0	27	I	within gene(s) PMM0452;
431710	-	TSS_021221	1000	124	0	1	I	within gene(s) PMM0452;
431803	-	TSS_021231	1000	322	0	15	I	within gene(s) PMM0452;
431848	-	TSS_021235	1000	226	0	39	I	within gene(s) PMM0452;
431905	-	TSS_021251	1000	162	0	10	I	within gene(s) PMM0452;
431932	-	TSS_021255	1000	246	0	12	I	within gene(s) PMM0452;
431969	+	TSS_004949	1000	142	0	1	Ai	antisense to gene(s) PMM0452;
431983	-	TSS_021262	1000	165	0	15	I	within gene(s) PMM0452;
432058	-	TSS_021276	1000	312	0	12	I	within gene(s) PMM0452;
432121	-	TSS_021286	1000	103	0	24	I	within gene(s) PMM0452;
432175	-	TSS_021293	1000	174	0	9	I	within gene(s) PMM0452;
432210	-	TSS_021296	1000	303	0	12	I	within gene(s) PMM0452;
432253	-	TSS_021301	1000	153	0	15	I	within gene(s) PMM0452;
432317	-	TSS_021307	1000	4193	0	3	P	16nt upstream of gene PMM0452;
432436	+	TSS_004954	1000	263	0	0	O	-
433380	-	TSS_021326	1000	278	0	5	P	16nt upstream of gene PMM0453;
433437	+	TSS_004958	1000	487	0	2	P	21nt upstream of gene PMM0454;
433825	-	TSS_021329	1000	239	0	4	Ai	antisense to gene(s) PMM0454;
433897	+	TSS_004963	1000	281	0	2	I	within gene(s) PMM0454;
434016	-	TSS_021331	1000	181	0	0	Ai	antisense to gene(s) PMM0454;
435917	-	TSS_021336	1000	399	0	0	P	19nt upstream of gene PMM0456;
437733	+	TSS_004981	1000	168	0	0	Ai	antisense to gene(s) PMM0458;
440026	-	TSS_021351	1000	146	0	3	IP	within gene(s) PMM0461; 221nt upstream of gene PMM0460;
440050	-	TSS_021352	1000	519	0	39	IP	within gene(s) PMM0461; 245nt upstream of gene PMM0460;
440245	-	TSS_021371	1000	200	0	21	I	within gene(s) PMM0461;
440290	-	TSS_021379	1000	264	0	18	I	within gene(s) PMM0461;
440323	-	TSS_021385	1000	113	0	12	I	within gene(s) PMM0461;
440341	-	TSS_021389	1000	283	0	18	I	within gene(s) PMM0461;
440437	-	TSS_021399	1000	105	0	3	I	within gene(s) PMM0461;
440473	-	TSS_021401	1000	113	0	6	I	within gene(s) PMM0461;
440497	-	TSS_021404	1000	138	0	6	I	within gene(s) PMM0461;
440515	-	TSS_021406	1000	155	0	12	I	within gene(s) PMM0461;
440581	-	TSS_021420	1000	248	0	33	I	within gene(s) PMM0461;
440608	-	TSS_021424	1000	108	0	9	I	within gene(s) PMM0461;
440626	-	TSS_021425	1000	124	0	3	I	within gene(s) PMM0461;
440658	-	TSS_021427	1000	111	0	0	I	within gene(s) PMM0461;
440695	-	TSS_021430	1000	148	0	0	I	within gene(s) PMM0461;
440885	-	TSS_021443	1000	362	0	18	IP	within gene(s) PMM0462; 115nt upstream of gene PMM0461;
440957	-	TSS_021455	1000	101	0	0	IP	within gene(s) PMM0462; 187nt upstream of gene PMM0461;
440990	-	TSS_021460	1000	121	0	39	IP	within gene(s) PMM0462; 220nt upstream of gene PMM0461;
441035	+	TSS_004998	1000	131	0	6	Ai	antisense to gene(s) PMM0462;
441068	-	TSS_021471	1000	163	0	18	I	within gene(s) PMM0462;
441185	-	TSS_021482	1000	190	0	4	I	within gene(s) PMM0462;
441296	-	TSS_021488	1000	135	0	0	I	within gene(s) PMM0462;
441308	-	TSS_021489	1000	109	0	0	I	within gene(s) PMM0462;
441340	-	TSS_021490	1000	1570	0	3	P	29nt upstream of gene PMM0462;
441384	-	TSS_021492	1000	111	0	0	P	73nt upstream of gene PMM0462;
442724	-	TSS_021501	1000	106	0	9	IP	within gene(s) PMM0465; 245nt upstream of gene PMM0464;
442840	-	TSS_021508	1000	2956	0	6	P	17nt upstream of gene PMM0465;
444471	+	TSS_005005	1000	153	0	1	PAi	157nt upstream of gene PMM0467; antisense to gene(s) PMM0466;
444597	-	TSS_021515	1000	177	0	1	P	44nt upstream of gene PMM0466;
445213	+	TSS_005010	1000	265	0	1	Ai	antisense to gene(s) PMM0468;
445383	-	TSS_021519	1000	137	0	7	IP	within gene(s) PMM0469; 49nt upstream of gene PMM0468;
445437	+	TSS_005012	1000	597	0	1	Ai	antisense to gene(s) PMM0469;
445460	-	TSS_021530	1000	223	0	66	IP	within gene(s) PMM0469; 126nt upstream of gene PMM0468;
445523	-	TSS_021546	1000	184	0	12	IP	within gene(s) PMM0469; 189nt upstream of gene PMM0468;
445535	-	TSS_021547	1000	113	0	0	IP	within gene(s) PMM0469; 201nt upstream of gene PMM0468;
445547	-	TSS_021548	1000	1042	0	0	IP	within gene(s) PMM0469; 213nt upstream of gene PMM0468;
445556	-	TSS_021549	1000	343	0	3	IP	within gene(s) PMM0469; 222nt upstream of gene PMM0468;
445575	+	TSS_005014	1000	364	0	0	Ai	antisense to gene(s) PMM0469;
445580	-	TSS_021555	1000	712	0	15	IP	within gene(s) PMM0469; 246nt upstream of gene PMM0468;
445601	-	TSS_021559	1000	743	0	12	I	within gene(s) PMM0469;
445625	-	TSS_021567	1000	286	0	30	I	within gene(s) PMM0469;
445658	-	TSS_021575	1000	552	0	0	I	within gene(s) PMM0469;
445694	-	TSS_021587	1000	1009	0	42	I	within gene(s) PMM0469;

445730	-	TSS_021597	1000	107	0	12	I	within gene(s) PMM0469;
445754	-	TSS_021601	1000	143	0	12	I	within gene(s) PMM0469;
445766	-	TSS_021603	1000	151	0	0	I	within gene(s) PMM0469;
445796	-	TSS_021604	1000	176	0	0	I	within gene(s) PMM0469;
445814	-	TSS_021605	1000	105	0	0	I	within gene(s) PMM0469;
445823	-	TSS_021606	1000	193	0	0	I	within gene(s) PMM0469;
445832	-	TSS_021607	1000	154	0	0	I	within gene(s) PMM0469;
445907	-	TSS_021609	1000	115	0	0	I	within gene(s) PMM0469;
445961	-	TSS_021612	1000	43946	0	7	P	42nt upstream of gene PMM0469;
445980	+	TSS_005016	1000	348	0	1	P	14nt upstream of gene PMM0470;
446547	+	TSS_005024	1000	147	0	0	I	within gene(s) PMM0470;
446689	+	TSS_005029	1000	110	0	2	I	within gene(s) PMM0470;
446903	+	TSS_005031	1000	330	0	0	IP	within gene(s) PMM0470; 167nt upstream of gene PMM0471;
447381	+	TSS_005035	1000	1214	0	5	P	29nt upstream of gene PMM0472;
450442	+	TSS_005047	1000	245	0	1	Ai	antisense to gene(s) PMM0474;
450594	-	TSS_021644	1000	194	0	0	P	113nt upstream of gene PMM0474;
450629	-	TSS_021646	1000	1744	0	1	P	148nt upstream of gene PMM0474;
450657	-	TSS_021647	1000	103	0	0	IP	within gene(s) PMM0475; 176nt upstream of gene PMM0474;
450680	-	TSS_021648	1000	579	0	0	IP	within gene(s) PMM0475; 199nt upstream of gene PMM0474;
450696	-	TSS_021649	1000	259	0	0	IP	within gene(s) PMM0475; 215nt upstream of gene PMM0474;
450872	+	TSS_005051	1000	270	0	2	Ai	antisense to gene(s) PMM0475;
450998	+	TSS_005052	1000	509	0	2	Ai	antisense to gene(s) PMM0475;
451103	-	TSS_021668	1000	153	0	0	I	within gene(s) PMM0475;
451442	+	TSS_005060	1000	480	0	8	PAi	101nt upstream of gene PMM0477; antisense to gene(s) PMM0476;
451486	+	TSS_005062	1000	1319	0	0	P	57nt upstream of gene PMM0477;
451522	-	TSS_021674	1000	425	0	8	P	69nt upstream of gene PMM0476;
451687	+	TSS_005076	1000	287	0	19	I	within gene(s) PMM0477;
451711	+	TSS_005081	1000	123	0	3	I	within gene(s) PMM0477;
451719	-	TSS_021676	1000	153	0	2	Ai	antisense to gene(s) PMM0477;
451768	+	TSS_005086	1000	127	0	3	I	within gene(s) PMM0477;
451801	+	TSS_005094	1000	284	0	24	I	within gene(s) PMM0477;
451813	+	TSS_005097	1000	276	0	3	I	within gene(s) PMM0477;
451849	+	TSS_005106	1000	276	0	36	I	within gene(s) PMM0477;
451913	+	TSS_005125	1000	1107	0	39	I	within gene(s) PMM0477;
451924	+	TSS_005127	1000	143	0	3	I	within gene(s) PMM0477;
451936	+	TSS_005129	1000	294	0	1	I	within gene(s) PMM0477;
452038	+	TSS_005133	1000	199	0	9	I	within gene(s) PMM0477;
452104	+	TSS_005145	1000	375	0	39	I	within gene(s) PMM0477;
452132	+	TSS_005151	1000	10218	0	12	I	within gene(s) PMM0477;
452249	-	TSS_021688	1000	137	0	0	Ai	antisense to gene(s) PMM0477;
453136	-	TSS_021692	1000	152	0	6	P	44nt upstream of gene PMM0478;
453247	+	TSS_005157	1000	764	0	2	P	22nt upstream of gene PMM0479;
453732	-	TSS_021699	1000	251	0	0	Ai	antisense to gene(s) PMM0479;
454968	+	TSS_005193	1000	162	0	4	P	16nt upstream of gene PMM0481;
455492	+	TSS_005194	1000	191	0	0	O	-
455674	-	TSS_021715	1000	144	0	6	O	-
455854	+	TSS_005197	1000	132	0	3	I	within gene(s) PMM0482;
455989	+	TSS_005215	1000	201	0	3	I	within gene(s) PMM0482;
456112	+	TSS_005221	1000	118	0	1	I	within gene(s) PMM0482;
456144	-	TSS_021722	1000	432	0	1	Ai	antisense to gene(s) PMM0482;
456178	+	TSS_005232	1000	157	0	6	I	within gene(s) PMM0482;
456250	+	TSS_005251	1000	377	0	60	I	within gene(s) PMM0482;
456313	+	TSS_005268	1000	435	0	57	I	within gene(s) PMM0482;
456568	+	TSS_005284	1000	316	0	2	I	within gene(s) PMM0482;
456940	+	TSS_005288	1000	479	0	3	Ai	antisense to gene(s) PMM0483;
457405	+	TSS_005293	1000	617	0	2	Ai	antisense to gene(s) PMM0483;
457551	+	TSS_005295	1000	155	0	1	Ai	antisense to gene(s) PMM0483;
457755	-	TSS_021747	1000	123	0	2	I	within gene(s) PMM0483;
457975	-	TSS_021754	1000	1838	0	5	P	36nt upstream of gene PMM0483;
458013	-	TSS_021759	1000	329	0	2	P	74nt upstream of gene PMM0483;
459029	-	TSS_021767	1000	297	0	0	P	17nt upstream of gene PMM0484;
459043	+	TSS_005298	1000	153	0	0	P	42nt upstream of gene PMM0485;
464573	+	TSS_005326	1000	208	0	1	Ai	antisense to gene(s) PMM0490;
465270	-	TSS_021777	1000	129	0	0	IP	within gene(s) PMM0492; 16nt upstream of gene PMM0491;
465840	+	TSS_005328	1000	722	0	3	P	16nt upstream of gene PMM0493;
466192	+	TSS_005330	1000	1080	0	2	I	within gene(s) PMM0493;
466610	+	TSS_005338	1000	154	0	0	I	within gene(s) PMM0493;
467409	+	TSS_005343	1000	158	0	3	P	15nt upstream of gene PMM0494;
467553	+	TSS_005346	1000	404	0	2	I	within gene(s) PMM0494;
467736	+	TSS_005364	1000	108	0	3	I	within gene(s) PMM0494;
468497	-	TSS_021798	1000	793	0	7	I	within gene(s) PMM0495;
469000	-	TSS_021809	1000	388	0	0	P	32nt upstream of gene PMM0495;
469311	-	TSS_021820	1000	190	0	0	I	within gene(s) PMM0496;
469488	+	TSS_005373	1000	131	0	1	Ai	antisense to gene(s) PMM0496;
470267	-	TSS_021877	1000	1373	0	1	P	14nt upstream of gene PMM0496;
474859	-	TSS_021894	1000	411	0	0	IP	within gene(s) PMM0500; 37nt upstream of gene PMM0499;
475324	-	TSS_021901	1000	469	0	4	I	within gene(s) PMM0500;

475383 -	TSS_021904	1000	475	0	0	P	19nt upstream of gene PMM0500;
475534 -	TSS_021905	1000	249	0	0	PAi	170nt upstream of gene PMM0500; antisense to gene(s) PMM0501;
475685 +	TSS_005389	1000	966	0	10	Ai	antisense to gene(s) PMM0502;
475749 +	TSS_005395	1000	2534	0	4	Ai	antisense to gene(s) PMM0502;
476045 -	TSS_021918	1000	1185	0	0	P	16nt upstream of gene PMM0502;
479542 -	TSS_021933	1000	775	0	2	P	26nt upstream of gene PMM0506;
479651 -	TSS_021934	1000	141	0	0	IP	within gene(s) PMM0507; 135nt upstream of gene PMM0506;
480046 -	TSS_021942	1000	164	0	1	P	11nt upstream of gene PMM0507;
480066 -	TSS_021944	1000	399	0	3	IP	within gene(s) PMM0508; 31nt upstream of gene PMM0507;
481882 -	TSS_021963	1000	705	0	2	P	18nt upstream of gene PMM0508;
482442 +	TSS_005417	1000	1765	0	1	P	36nt upstream of gene PMM0510;
483044 -	TSS_021967	1000	405	0	2	Ai	antisense to gene(s) PMM0511;
483815 +	TSS_005422	1000	115	0	0	I	within gene(s) PMM0513;
483869 +	TSS_005424	1000	372	0	3	I	within gene(s) PMM0513;
486912 -	TSS_021983	1000	790	0	6	P	21nt upstream of gene PMM0515;
487573 +	TSS_005436	1000	227	0	1	I	within gene(s) PMM0516;
490430 +	TSS_005444	1000	213	0	4	I	within gene(s) PMM0518;
490807 +	TSS_005449	1000	198	0	18	I	within gene(s) PMM0519;
490849 +	TSS_005457	1000	192	0	15	I	within gene(s) PMM0519;
490885 +	TSS_005465	1000	191	0	24	I	within gene(s) PMM0519;
491260 +	TSS_005509	1000	1046	0	60	I	within gene(s) PMM0519;
491296 +	TSS_005518	1000	154	0	21	I	within gene(s) PMM0519;
491487 -	TSS_021999	1000	140	0	0	Ai	antisense to gene(s) PMM0519;
491974 -	TSS_022004	1000	143	0	0	I	within gene(s) PMM0520;
493398 -	TSS_022018	1000	559	0	5	I	within gene(s) PMM0521;
493513 -	TSS_022025	1000	332	0	0	IP	within gene(s) PMM0522; 82nt upstream of gene PMM0521;
493630 +	TSS_005546	1000	191	0	3	Ai	antisense to gene(s) PMM0522;
493755 +	TSS_005548	1000	117	0	0	Ai	antisense to gene(s) PMM0522;
493996 -	TSS_022034	1000	183	0	3	I	within gene(s) PMM0522;
494529 +	TSS_005553	1000	238	0	0	Ai	antisense to gene(s) PMM0523;
495001 -	TSS_022042	1000	897	0	8	P	12nt upstream of gene PMM0523;
496211 +	TSS_005561	1000	2171	0	3	P	44nt upstream of gene PMM0525;
496222 +	TSS_005563	1000	134	0	0	P	33nt upstream of gene PMM0525;
497254 +	TSS_005574	1000	273	0	6	I	within gene(s) PMM0525;
497550 +	TSS_005578	1000	4519	0	2	P	15nt upstream of gene PMM0526;
498172 +	TSS_005614	1000	127	0	4	I	within gene(s) PMM0526;
498249 +	TSS_005628	1000	174	0	34	I	within gene(s) PMM0526;
498330 +	TSS_005637	1000	147	0	6	I	within gene(s) PMM0526;
498643 +	TSS_005652	1000	268	0	1	I	within gene(s) PMM0526;
498684 +	TSS_005655	1000	127	0	0	I	within gene(s) PMM0526;
498840 +	TSS_005672	1000	249	0	3	I	within gene(s) PMM0526;
498873 +	TSS_005678	1000	575	0	16	I	within gene(s) PMM0526;
498894 +	TSS_005681	1000	123	0	3	I	within gene(s) PMM0526;
498964 -	TSS_022061	1000	209	0	2	Ai	antisense to gene(s) PMM0526;
498978 +	TSS_005683	1000	110	0	3	I	within gene(s) PMM0526;
499020 +	TSS_005685	1000	325	0	3	I	within gene(s) PMM0526;
499041 +	TSS_005688	1000	138	0	12	I	within gene(s) PMM0526;
499074 +	TSS_005699	1000	474	0	38	I	within gene(s) PMM0526;
499119 +	TSS_005711	1000	146	0	0	I	within gene(s) PMM0526;
502199 -	TSS_022087	1000	118	0	0	I	within gene(s) PMM0530;
502675 +	TSS_005715	1000	331	0	0	P	47nt upstream of gene PMM0531;
502682 -	TSS_022106	1000	1411	0	11	P	24nt upstream of gene PMM0530;
503618 +	TSS_005730	1000	274	0	10	P	13nt upstream of gene PMM0532;
503904 +	TSS_005744	1000	135	0	0	I	within gene(s) PMM0532;
503914 -	TSS_022110	1000	120	0	0	Ai	antisense to gene(s) PMM0532;
504000 +	TSS_005754	1000	219	0	0	I	within gene(s) PMM0532;
504036 +	TSS_005757	1000	125	0	18	I	within gene(s) PMM0532;
504120 +	TSS_005774	1000	107	0	33	I	within gene(s) PMM0532;
504156 +	TSS_005781	1000	177	0	7	I	within gene(s) PMM0532;
504428 +	TSS_005786	1000	379	0	5	P	29nt upstream of gene PMM0533;
504905 +	TSS_005791	1000	142	0	1	I	within gene(s) PMM0533;
505257 -	TSS_022115	1000	156	0	0	Ai	antisense to gene(s) PMM0533;
505261 +	TSS_005797	1000	715	0	10	IP	within gene(s) PMM0533; 240nt upstream of gene PMM0534;
505757 +	TSS_005806	1000	253	0	0	I	within gene(s) PMM0534;
506152 +	TSS_005809	1000	208	0	0	I	within gene(s) PMM0534;
507310 +	TSS_005814	1000	652	0	1	P	62nt upstream of gene PMM0536;
507537 +	TSS_005826	1000	179	0	24	I	within gene(s) PMM0536;
507738 +	TSS_005850	1000	137	0	6	I	within gene(s) PMM0536;
508770 -	TSS_022121	1000	214	0	0	I	within gene(s) PMM0537;
511407 +	TSS_005863	1000	115	0	0	P	58nt upstream of gene PMM0542;
512559 +	TSS_005868	1000	166	0	0	Ai	antisense to gene(s) PMM0543;
512746 -	TSS_022132	1000	211	0	18	I	within gene(s) PMM0543;
512812 -	TSS_022145	1000	122	0	15	I	within gene(s) PMM0543;
512853 +	TSS_005872	1000	4056	0	3	Ai	antisense to gene(s) PMM0543;
512911 -	TSS_022149	1000	866	0	12	I	within gene(s) PMM0543;
512932 -	TSS_022155	1000	207	0	3	I	within gene(s) PMM0543;
512962 -	TSS_022157	1000	108	0	0	I	within gene(s) PMM0543;

512974	-	TSS_022158	1000	119	0	0	I	within gene(s) PMM0543;
513022	-	TSS_022159	1000	1094	0	5	I	within gene(s) PMM0543;
513034	-	TSS_022162	1000	135	0	0	I	within gene(s) PMM0543;
513036	+	TSS_005876	1000	148	0	1	Ai	antisense to gene(s) PMM0543;
513049	-	TSS_022163	1000	255	0	3	I	within gene(s) PMM0543;
513063	+	TSS_005878	1000	142	0	0	Ai	antisense to gene(s) PMM0543;
513082	-	TSS_022166	1000	149	0	3	I	within gene(s) PMM0543;
513094	-	TSS_022167	1000	194	0	15	I	within gene(s) PMM0543;
513118	-	TSS_022172	1000	269	0	36	I	within gene(s) PMM0543;
513169	-	TSS_022185	1000	354	0	21	I	within gene(s) PMM0543;
513211	-	TSS_022197	1000	150	0	22	I	within gene(s) PMM0543;
513328	-	TSS_022208	1000	194	0	12	I	within gene(s) PMM0543;
513385	-	TSS_022212	1000	11296	0	5	P	24nt upstream of gene PMM0543;
513547	+	TSS_005882	1000	248	0	0	Ad	antisense to gene(s) PMM0544 (7nt downstream);
513696	-	TSS_022222	1000	194	0	22	I	within gene(s) PMM0544;
513927	-	TSS_022251	1000	360	0	3	I	within gene(s) PMM0544;
513939	-	TSS_022253	1000	118	0	36	I	within gene(s) PMM0544;
513981	-	TSS_022261	1000	123	0	0	I	within gene(s) PMM0544;
514059	-	TSS_022270	1000	181	0	12	I	within gene(s) PMM0544;
514146	-	TSS_022279	1000	145	0	6	I	within gene(s) PMM0544;
514224	-	TSS_022290	1000	129	0	27	I	within gene(s) PMM0544;
514308	-	TSS_022309	1000	324	0	43	I	within gene(s) PMM0544;
514323	-	TSS_022313	1000	119	0	6	I	within gene(s) PMM0544;
514356	-	TSS_022321	1000	1218	0	27	I	within gene(s) PMM0544;
514401	-	TSS_022325	1000	273	0	6	I	within gene(s) PMM0544;
514419	-	TSS_022327	1000	101	0	0	I	within gene(s) PMM0544;
514455	-	TSS_022331	1000	244	0	21	I	within gene(s) PMM0544;
514479	-	TSS_022336	1000	274	0	15	I	within gene(s) PMM0544;
514548	-	TSS_022347	1000	250	0	49	I	within gene(s) PMM0544;
514620	-	TSS_022358	1000	196	0	12	I	within gene(s) PMM0544;
514656	-	TSS_022366	1000	244	0	12	I	within gene(s) PMM0544;
514668	-	TSS_022370	1000	549	0	27	I	within gene(s) PMM0544;
514800	-	TSS_022384	1000	126	0	3	I	within gene(s) PMM0544;
514833	-	TSS_022388	1000	112	0	9	I	within gene(s) PMM0544;
514869	-	TSS_022395	1000	247	0	33	I	within gene(s) PMM0544;
514896	-	TSS_022400	1000	515	0	24	I	within gene(s) PMM0544;
514938	-	TSS_022408	1000	129	0	24	I	within gene(s) PMM0544;
514960	+	TSS_005899	1000	139	0	0	Ai	antisense to gene(s) PMM0544;
515006	+	TSS_005900	1000	356	0	0	Ai	antisense to gene(s) PMM0544;
515031	-	TSS_022417	1000	148	0	6	I	within gene(s) PMM0544;
515064	-	TSS_022420	1000	128	0	12	I	within gene(s) PMM0544;
515091	-	TSS_022425	1000	906	0	42	I	within gene(s) PMM0544;
515154	-	TSS_022438	1000	1104	0	10	IP	within gene(s) PMM0545; 21nt upstream of gene PMM0544;
515174	-	TSS_022443	1000	255	0	13	IP	within gene(s) PMM0545; 41nt upstream of gene PMM0544;
515202	-	TSS_022449	1000	392	0	8	IP	within gene(s) PMM0545; 69nt upstream of gene PMM0544;
515223	-	TSS_022453	1000	173	0	0	IP	within gene(s) PMM0545; 90nt upstream of gene PMM0544;
515244	-	TSS_022454	1000	107	2.60E-10	0	IP	within gene(s) PMM0545; 111nt upstream of gene PMM0544;
515262	-	TSS_022456	1000	121	0	0	IP	within gene(s) PMM0545; 129nt upstream of gene PMM0544;
515274	-	TSS_022457	1000	136	0	3	IP	within gene(s) PMM0545; 141nt upstream of gene PMM0544;
515289	-	TSS_022459	1000	126	0	0	IP	within gene(s) PMM0545; 156nt upstream of gene PMM0544;
515316	-	TSS_022460	1000	342	0	0	IP	within gene(s) PMM0545; 183nt upstream of gene PMM0544;
515373	-	TSS_022477	1000	1271	0	69	IP	within gene(s) PMM0545; 240nt upstream of gene PMM0544;
515412	-	TSS_022490	1000	369	0	9	I	within gene(s) PMM0545;
515439	-	TSS_022492	1000	413	0	15	I	within gene(s) PMM0545;
515461	+	TSS_005909	1000	159	0	0	Ai	antisense to gene(s) PMM0545;
515472	-	TSS_022499	1000	176	0	18	I	within gene(s) PMM0545;
515529	-	TSS_022512	1000	209	0	30	I	within gene(s) PMM0545;
515583	-	TSS_022513	1000	128	0	0	I	within gene(s) PMM0545;
515622	-	TSS_022515	1000	112	0	6	I	within gene(s) PMM0545;
515643	-	TSS_022519	1000	178	0	16	I	within gene(s) PMM0545;
515673	-	TSS_022525	1000	124	0	0	I	within gene(s) PMM0545;
515694	-	TSS_022530	1000	295	0	15	I	within gene(s) PMM0545;
515718	-	TSS_022535	1000	356	0	12	I	within gene(s) PMM0545;
515763	-	TSS_022544	1000	541	0	21	I	within gene(s) PMM0545;
515781	-	TSS_022549	1000	489	0	3	I	within gene(s) PMM0545;
515800	+	TSS_005913	1000	378	0	1	Ai	antisense to gene(s) PMM0545;
515817	-	TSS_022555	1000	350	0	39	I	within gene(s) PMM0545;
515850	-	TSS_022561	1000	253	0	3	I	within gene(s) PMM0545;
515874	-	TSS_022563	1000	229	0	0	I	within gene(s) PMM0545;
515884	+	TSS_005919	1000	190	0	0	Ai	antisense to gene(s) PMM0545;
515889	-	TSS_022565	1000	283	0	30	I	within gene(s) PMM0545;
515916	+	TSS_005920	1000	270	0	1	Ai	antisense to gene(s) PMM0545;
515930	+	TSS_005922	1000	372	0	0	Ai	antisense to gene(s) PMM0545;
515937	-	TSS_022571	1000	144	0	0	I	within gene(s) PMM0545;
515964	-	TSS_022574	1000	301	0	12	I	within gene(s) PMM0545;
515970	+	TSS_005923	1000	381	0	0	Ai	antisense to gene(s) PMM0545;
516012	-	TSS_022587	1000	192	0	42	I	within gene(s) PMM0545;

516030	-	TSS_022591	1000	229	0	6	I	within gene(s) PMM0545;
516048	-	TSS_022595	1000	492	0	15	I	within gene(s) PMM0545;
516081	-	TSS_022603	1000	222	0	16	I	within gene(s) PMM0545;
516105	-	TSS_022607	1000	447	0	24	I	within gene(s) PMM0545;
516159	-	TSS_022616	1000	311	0	33	I	within gene(s) PMM0545;
516192	-	TSS_022623	1000	1400	0	12	I	within gene(s) PMM0545;
516228	-	TSS_022628	1000	373	0	4	I	within gene(s) PMM0545;
516267	-	TSS_022630	1000	323	0	0	I	within gene(s) PMM0545;
516294	-	TSS_022631	1000	165	0	0	I	within gene(s) PMM0545;
516308	-	TSS_022634	1000	241	0	13	I	within gene(s) PMM0545;
516327	-	TSS_022639	1000	124	0	0	I	within gene(s) PMM0545;
516345	-	TSS_022642	1000	377	0	7	I	within gene(s) PMM0545;
516366	-	TSS_022644	1000	154	0	0	I	within gene(s) PMM0545;
516813	-	TSS_022650	1000	137	0	2	I	within gene(s) PMM0546;
516894	-	TSS_022657	1000	109	0	12	I	within gene(s) PMM0546;
518401	-	TSS_022666	1000	159	0	1	P	18nt upstream of gene PMM0548;
518485	-	TSS_022668	1000	595	0	0	P	102nt upstream of gene PMM0548;
518726	+	TSS_005935	1000	165	0	0	I	within gene(s) PMM0549;
518767	+	TSS_005937	1000	129	0	6	I	within gene(s) PMM0549;
518776	+	TSS_005938	1000	153	0	18	I	within gene(s) PMM0549;
518833	+	TSS_005950	1000	173	0	30	I	within gene(s) PMM0549;
518878	+	TSS_005952	1000	170	0	9	IP	within gene(s) PMM0549; 209nt upstream of gene PMM0550;
518917	+	TSS_005959	1000	261	0	21	IP	within gene(s) PMM0549; 170nt upstream of gene PMM0550;
518950	+	TSS_005968	1000	196	0	18	IP	within gene(s) PMM0549; 137nt upstream of gene PMM0550;
518971	+	TSS_005972	1000	444	0	16	IP	within gene(s) PMM0549; 116nt upstream of gene PMM0550;
519004	+	TSS_005977	1000	347	0	1	IP	within gene(s) PMM0549; 83nt upstream of gene PMM0550;
519082	-	TSS_022672	1000	501	0	1	O	-
519114	+	TSS_005980	1000	139	0	0	I	within gene(s) PMM0550;
519141	+	TSS_005982	1000	156	0	6	I	within gene(s) PMM0550;
519189	+	TSS_005986	1000	104	0	15	I	within gene(s) PMM0550;
519216	+	TSS_005992	1000	846	0	19	I	within gene(s) PMM0550;
519246	+	TSS_005999	1000	201	0	0	I	within gene(s) PMM0550;
519258	+	TSS_006000	1000	217	0	0	I	within gene(s) PMM0550;
519354	+	TSS_006006	1000	225	0	1	I	within gene(s) PMM0550;
519381	+	TSS_006010	1000	147	0	0	I	within gene(s) PMM0550;
519402	+	TSS_006011	1000	126	0	0	I	within gene(s) PMM0550;
519459	+	TSS_006017	1000	138	0	3	I	within gene(s) PMM0550;
519486	+	TSS_006020	1000	118	0	6	I	within gene(s) PMM0550;
519513	+	TSS_006026	1000	782	0	16	I	within gene(s) PMM0550;
519588	+	TSS_006041	1000	488	0	36	I	within gene(s) PMM0550;
519603	+	TSS_006042	1000	602	0	9	I	within gene(s) PMM0550;
519669	+	TSS_006051	1000	397	0	21	I	within gene(s) PMM0550;
519837	+	TSS_006059	1000	128	0	6	I	within gene(s) PMM0550;
519874	+	TSS_006063	1000	187	0	0	I	within gene(s) PMM0550;
520043	-	TSS_022684	1000	119	0	0	Ai	antisense to gene(s) PMM0550;
520056	-	TSS_022685	1000	199	0	0	Ai	antisense to gene(s) PMM0550;
520155	+	TSS_006071	1000	217	0	3	I	within gene(s) PMM0550;
520175	-	TSS_022686	1000	283	0	0	Ai	antisense to gene(s) PMM0550;
520182	+	TSS_006072	1000	217	0	0	I	within gene(s) PMM0550;
520216	+	TSS_006076	1000	145	0	4	I	within gene(s) PMM0550;
520224	+	TSS_006077	1000	1227	0	1	I	within gene(s) PMM0550;
520239	+	TSS_006080	1000	152	0	12	I	within gene(s) PMM0550;
520278	+	TSS_006087	1000	240	0	7	I	within gene(s) PMM0550;
520352	-	TSS_022687	1000	123	0	1	Ai	antisense to gene(s) PMM0550;
520384	-	TSS_022691	1000	656	0	0	Ai	antisense to gene(s) PMM0550;
520485	+	TSS_006099	1000	141	0	0	IP	within gene(s) PMM0550; 107nt upstream of gene PMM0551;
520583	+	TSS_006101	1000	110	0	14	P	9nt upstream of gene PMM0551;
520703	+	TSS_006111	1000	102	0	6	I	within gene(s) PMM0551;
521513	+	TSS_006118	1000	125	0	1	I	within gene(s) PMM0552;
521962	-	TSS_022704	1000	138	0	4	Ai	antisense to gene(s) PMM0552;
521966	+	TSS_006155	1000	111	0	0	I	within gene(s) PMM0552;
521993	+	TSS_006158	1000	151	0	6	I	within gene(s) PMM0552;
522098	-	TSS_022706	1000	104	0	0	Ai	antisense to gene(s) PMM0552;
522119	+	TSS_006170	1000	237	0	34	I	within gene(s) PMM0552;
522292	-	TSS_022708	1000	130	0	0	Ai	antisense to gene(s) PMM0552;
522341	+	TSS_006191	1000	110	0	0	I	within gene(s) PMM0552;
522503	+	TSS_006206	1000	151	0	9	I	within gene(s) PMM0552;
522626	+	TSS_006216	1000	120	0	3	I	within gene(s) PMM0552;
522659	+	TSS_006218	1000	224	0	6	I	within gene(s) PMM0552;
522744	+	TSS_006230	1000	112	0	7	I	within gene(s) PMM0552;
523106	-	TSS_022717	1000	199	0	10	Ai	antisense to gene(s) PMM0552;
523135	-	TSS_022719	1000	123	0	0	Ai	antisense to gene(s) PMM0552;
523163	-	TSS_022721	1000	170	0	0	Ai	antisense to gene(s) PMM0552;
523173	-	TSS_022723	1000	257	0	4	Ai	antisense to gene(s) PMM0552;
524032	-	TSS_022732	1000	106	0	2	Ai	antisense to gene(s) PMM0553;
524269	-	TSS_022735	1000	182	0	0	Ai	antisense to gene(s) PMM0553;
524568	+	TSS_006280	1000	101	0	0	I	within gene(s) PMM0553;

524632	-	TSS_022739	1000	3025	0	2	Ai	antisense to gene(s) PMM0553;
524671	+	TSS_006281	1000	227	0	0	IP	within gene(s) PMM0553; 191nt upstream of gene PMM0554;
524722	+	TSS_006282	1000	112	0	2	IP	within gene(s) PMM0553; 140nt upstream of gene PMM0554;
524738	+	TSS_006284	1000	360	0	0	IP	within gene(s) PMM0553; 124nt upstream of gene PMM0554;
524791	+	TSS_006286	1000	1124	0	1	IP	within gene(s) PMM0553; 71nt upstream of gene PMM0554;
525973	-	TSS_022746	1000	5952	0	4	P	18nt upstream of gene PMM0557;
525984	-	TSS_022749	1000	107	0	1	P	29nt upstream of gene PMM0557;
526448	-	TSS_022754	1000	1520	0	4	P	15nt upstream of gene PMM0558;
527220	+	TSS_006312	1000	2780	0	1	P	22nt upstream of gene PMM0560;
527254	+	TSS_006313	1000	746	0	6	I	within gene(s) PMM0560;
527553	-	TSS_022758	1000	121	0	0	Ai	antisense to gene(s) PMM0560;
529035	+	TSS_006334	1000	344	0	4	I	within gene(s) PMM0561;
529418	+	TSS_006344	1000	108	0	0	I	within gene(s) PMM0561;
531701	+	TSS_006355	1000	487	0	4	P	15nt upstream of gene PMM0565;
532356	-	TSS_022777	1000	383	0	1	Ai	antisense to gene(s) PMM0565;
532769	+	TSS_006370	1000	206	0	3	I	within gene(s) PMM0565;
534371	+	TSS_006377	1000	244	0	7	P	6nt upstream of gene PMM0567;
538166	+	TSS_006389	1000	1331	0	2	P	179nt upstream of gene PMM0570;
539278	-	TSS_022799	1000	108	0	2	Ai	antisense to gene(s) PMM0572;
539865	+	TSS_006395	1000	425	0	0	Ad	antisense to gene(s) PMM0573 (15nt downstream);
539992	+	TSS_006398	1000	933	0	0	Ai	antisense to gene(s) PMM0573;
540038	+	TSS_006401	1000	113	0	0	Ai	antisense to gene(s) PMM0573;
540073	+	TSS_006402	1000	173	0	8	Ai	antisense to gene(s) PMM0573;
540156	-	TSS_022807	1000	513	0	13	P	2nt upstream of gene PMM0573;
540186	-	TSS_022814	1000	230	0	18	P	32nt upstream of gene PMM0573;
540215	-	TSS_022821	1000	211	0	18	P	61nt upstream of gene PMM0573;
540317	-	TSS_022826	1000	341	0	0	P	163nt upstream of gene PMM0573;
541550	+	TSS_006409	1000	164	0	0	Ai	antisense to gene(s) PMM0577;
541789	-	TSS_022851	1000	107	0	18	I	within gene(s) PMM0577;
541837	-	TSS_022860	1000	293	0	36	I	within gene(s) PMM0577;
542147	-	TSS_022883	1000	2682	0	24	I	within gene(s) PMM0577;
542296	-	TSS_022897	1000	117	0	0	I	within gene(s) PMM0577;
543050	+	TSS_006419	1000	140	0	1	Ai	antisense to gene(s) PMM0578;
543260	+	TSS_006421	1000	433	0	2	P	50nt upstream of gene PMM0579;
543284	-	TSS_022923	1000	929	0	4	P	38nt upstream of gene PMM0578;
546045	-	TSS_022939	1000	1724	0	1	I	within gene(s) PMM0580;
546519	+	TSS_006434	1000	151	0	0	Ai	antisense to gene(s) PMM0581;
546530	+	TSS_006436	1000	1288	0	2	Ai	antisense to gene(s) PMM0581;
546644	-	TSS_022945	1000	197	0	18	IP	within gene(s) PMM0581; 234nt upstream of gene PMM0580;
546683	-	TSS_022950	1000	144	0	12	I	within gene(s) PMM0581;
546698	-	TSS_022953	1000	123	0	3	I	within gene(s) PMM0581;
546722	-	TSS_022960	1000	192	0	45	I	within gene(s) PMM0581;
546782	-	TSS_022977	1000	487	0	17	I	within gene(s) PMM0581;
546791	-	TSS_022979	1000	189	0	0	I	within gene(s) PMM0581;
546806	-	TSS_022981	1000	189	0	9	I	within gene(s) PMM0581;
546819	-	TSS_022984	1000	422	0	0	I	within gene(s) PMM0581;
547595	-	TSS_022985	1000	146	0	0	I	within gene(s) PMM0582;
548120	-	TSS_022998	1000	152	0	27	I	within gene(s) PMM0583;
548321	-	TSS_023015	1000	137	0	6	I	within gene(s) PMM0583;
548504	-	TSS_023022	1000	108	0	0	I	within gene(s) PMM0583;
548744	-	TSS_023036	1000	143	0	4	I	within gene(s) PMM0583;
548771	-	TSS_023038	1000	131	0	6	I	within gene(s) PMM0583;
548792	-	TSS_023043	1000	113	0	21	I	within gene(s) PMM0583;
548961	-	TSS_023054	1000	4504	0	2	P	43nt upstream of gene PMM0583;
550115	+	TSS_006455	1000	179	0	0	Ai	antisense to gene(s) PMM0584;
551319	-	TSS_023085	1000	438	0	2	P	13nt upstream of gene PMM0584;
558189	+	TSS_006468	1000	133	0	1	P	13nt upstream of gene PMM0590;
558487	+	TSS_006474	1000	105	0	3	I	within gene(s) PMM0590;
561219	-	TSS_023121	1000	274	0	0	I	within gene(s) PMM0593;
562701	+	TSS_006486	1000	498	0	0	Ai	antisense to gene(s) PMM0594;
562722	+	TSS_006488	1000	193	0	6	Ai	antisense to gene(s) PMM0594;
563080	-	TSS_023180	1000	149	0	0	I	within gene(s) PMM0594;
563864	-	TSS_023192	1000	152	0	1	I	within gene(s) PMM0594;
564355	+	TSS_006501	1000	220	0	1	Ai	antisense to gene(s) PMM0595;
564712	-	TSS_023207	1000	225	0	9	I	within gene(s) PMM0595;
564862	-	TSS_023212	1000	121	0	0	I	within gene(s) PMM0595;
565234	-	TSS_023215	1000	169	0	4	IP	within gene(s) PMM0596; 90nt upstream of gene PMM0595;
565420	-	TSS_023218	1000	1282	0	1	I	within gene(s) PMM0596;
567864	+	TSS_006513	1000	146	0	5	Ai	antisense to gene(s) PMM0597;
569436	-	TSS_023243	1000	155	0	40	I	within gene(s) PMM0599;
569475	-	TSS_023251	1000	106	0	23	I	within gene(s) PMM0599;
569600	-	TSS_023258	1000	1072	0	8	P	20nt upstream of gene PMM0599;
569733	+	TSS_006520	1000	114	0	3	P	18nt upstream of gene PMM0600;
570537	+	TSS_006522	1000	122	0	7	I	within gene(s) PMM0600;
570717	+	TSS_006542	1000	239	0	24	I	within gene(s) PMM0600;
571068	-	TSS_023268	1000	129	0	1	Ai	antisense to gene(s) PMM0600;
571704	+	TSS_006554	1000	121	0	2	I	within gene(s) PMM0601;

572225	+	TSS_006556	1000	124	0	1	I	within gene(s) PMM0602;
572335	+	TSS_006560	1000	122	0	3	I	within gene(s) PMM0602;
572734	+	TSS_006566	1000	118	0	0	IP	within gene(s) PMM0602; 61nt upstream of gene PMM0603;
573409	+	TSS_006570	1000	132	0	1	I	within gene(s) PMM0603;
573526	-	TSS_023276	1000	646	0	1	Ai	antisense to gene(s) PMM0603;
573992	+	TSS_006573	1000	124	0	0	I	within gene(s) PMM0604;
574977	+	TSS_006578	1000	216	0	1	Ai	antisense to gene(s) PMM0605;
575199	-	TSS_023282	1000	2610	0	1	P	16nt upstream of gene PMM0605;
575286	-	TSS_023283	1000	115	0	0	IP	within gene(s) PMM0606; 103nt upstream of gene PMM0605;
577744	+	TSS_006586	1000	188	0	0	I	within gene(s) PMM0608;
578017	-	TSS_023290	1000	304	0	0	Ai	antisense to gene(s) PMM0608;
578318	+	TSS_006594	1000	1127	0	2	IP	within gene(s) PMM0608; 247nt upstream of gene PMM0609;
578964	-	TSS_023295	1000	168	0	0	Ai	antisense to gene(s) PMM0609;
579086	-	TSS_023298	1000	111	0	0	Ai	antisense to gene(s) PMM0609;
579495	+	TSS_006607	1000	464	0	0	I	within gene(s) PMM0609;
579542	+	TSS_006608	1000	118	0	6	I	within gene(s) PMM0609;
582602	-	TSS_023307	1000	419	0	5	I	within gene(s) PMM0611;
582767	-	TSS_023310	1000	231	0	6	P	17nt upstream of gene PMM0611;
584058	-	TSS_023316	1000	261	0	2	I	within gene(s) PMM0613;
585123	+	TSS_006633	1000	1803	0	6	P	120nt upstream of gene PMM0614;
585737	+	TSS_006639	1000	668	0	1	IP	within gene(s) PMM0614; 160nt upstream of gene PMM0615;
585797	+	TSS_006641	1000	375	0	4	IP	within gene(s) PMM0614; 100nt upstream of gene PMM0615;
585844	+	TSS_006644	1000	132	0	6	IP	within gene(s) PMM0614; 53nt upstream of gene PMM0615;
586359	+	TSS_006652	1000	279	0	3	I	within gene(s) PMM0615;
587070	-	TSS_023328	1000	358	0	0	Ai	antisense to gene(s) PMM0616;
588622	+	TSS_006660	1000	1408	0	4	P	9nt upstream of gene PMM0618;
588874	+	TSS_006665	1000	262	0	12	I	within gene(s) PMM0618;
589671	+	TSS_006673	1000	215	0	0	P	29nt upstream of gene PMM0619;
590069	+	TSS_006684	1000	418	0	15	I	within gene(s) PMM0619;
590180	+	TSS_006696	1000	311	0	25	I	within gene(s) PMM0619;
590216	+	TSS_006702	1000	310	0	0	I	within gene(s) PMM0619;
590270	+	TSS_006705	1000	129	0	12	I	within gene(s) PMM0619;
590314	-	TSS_023334	1000	144	0	1	Ai	antisense to gene(s) PMM0619;
590531	+	TSS_006716	1000	111	0	6	I	within gene(s) PMM0619;
590670	-	TSS_023337	1000	1340	0	3	Ai	antisense to gene(s) PMM0619;
590828	+	TSS_006727	1000	150	0	0	I	within gene(s) PMM0619;
591023	+	TSS_006746	1000	207	0	18	I	within gene(s) PMM0619;
591039	+	TSS_006749	1000	104	0	9	I	within gene(s) PMM0619;
591055	-	TSS_023339	1000	346	0	0	Ai	antisense to gene(s) PMM0619;
591311	+	TSS_006767	1000	211	0	1	I	within gene(s) PMM0619;
593663	-	TSS_023351	1000	232	0	0	I	within gene(s) PMM0622;
594134	+	TSS_006781	1000	105	0	0	I	within gene(s) PMM0623;
594680	+	TSS_006783	1000	151	0	2	I	within gene(s) PMM0623;
595886	+	TSS_006786	1000	188	0	1	P	18nt upstream of gene PMM0626;
596067	-	TSS_023356	1000	177	0	0	Ai	antisense to gene(s) PMM0626;
596385	-	TSS_023358	1000	1409	0	2	O	-
597027	+	TSS_006790	1000	993	0	6	I	within gene(s) PMM0627;
597035	-	TSS_023364	1000	2250	0	2	Ai	antisense to gene(s) PMM0627;
597045	+	TSS_006792	1000	1280	0	16	I	within gene(s) PMM0627;
597087	+	TSS_006800	1000	1470	0	18	I	within gene(s) PMM0627;
597111	+	TSS_006803	1000	750	0	0	I	within gene(s) PMM0627;
597120	+	TSS_006804	1000	2110	0	0	I	within gene(s) PMM0627;
597162	+	TSS_006813	1000	3214	0	51	I	within gene(s) PMM0627;
597165	-	TSS_023367	1000	1757	0	0	Ai	antisense to gene(s) PMM0627;
597198	+	TSS_006824	1000	1584	0	0	I	within gene(s) PMM0627;
597256	+	TSS_006845	1000	2310	0	51	I	within gene(s) PMM0627;
597282	+	TSS_006849	1000	1643	0	27	I	within gene(s) PMM0627;
597318	+	TSS_006858	1000	552	0	6	I	within gene(s) PMM0627;
597363	+	TSS_006860	1000	536	0	3	I	within gene(s) PMM0627;
597375	+	TSS_006862	1000	1264	0	9	I	within gene(s) PMM0627;
597393	+	TSS_006865	1000	3791	0	3	I	within gene(s) PMM0627;
597411	+	TSS_006870	1000	6852	0	12	I	within gene(s) PMM0627;
597430	-	TSS_023373	1000	130	0	4	Ai	antisense to gene(s) PMM0627;
597432	+	TSS_006875	1000	777	0	3	I	within gene(s) PMM0627;
597468	+	TSS_006878	1000	1160	0	9	I	within gene(s) PMM0627;
597501	+	TSS_006885	1000	135	0	3	I	within gene(s) PMM0627;
600230	+	TSS_006894	1000	659	0	1	P	26nt upstream of gene PMM0631;
601604	-	TSS_023383	1000	108	0	0	IP	within gene(s) PMM0633; 102nt upstream of gene PMM0632;
602377	-	TSS_023385	1000	423	0	2	I	within gene(s) PMM0633;
602756	-	TSS_023389	1000	105	0	0	I	within gene(s) PMM0633;
602896	-	TSS_023395	1000	771	0	5	P	98nt upstream of gene PMM0633;
605794	+	TSS_006906	1000	281	0	6	P	19nt upstream of gene PMM0637;
608933	-	TSS_023423	1000	172	0	0	Ai	antisense to gene(s) PMM0640;
609209	-	TSS_023425	1000	761	0	1	Ai	antisense to gene(s) PMM0640;
609497	+	TSS_006917	1000	151	0	0	I	within gene(s) PMM0640;
610210	+	TSS_006926	1000	3993	0	7	P	16nt upstream of gene PMM0641;
610941	+	TSS_006932	1000	9916	0	3	P	17nt upstream of gene PMM0642;

611018	+	TSS_006938	1000	146	0	9	I	within gene(s) PMM0642;
611411	+	TSS_006967	1000	939	0	30	I	within gene(s) PMM0642;
611432	+	TSS_006972	1000	128	0	3	I	within gene(s) PMM0642;
611447	+	TSS_006974	1000	160	0	3	I	within gene(s) PMM0642;
611474	+	TSS_006976	1000	362	0	6	I	within gene(s) PMM0642;
611513	+	TSS_006980	1000	369	0	9	I	within gene(s) PMM0642;
611626	-	TSS_023431	1000	495	0	10	Ai	antisense to gene(s) PMM0642;
611639	+	TSS_006991	1000	110	0	27	I	within gene(s) PMM0642;
611662	-	TSS_023434	1000	101	0	0	Ai	antisense to gene(s) PMM0642;
611674	-	TSS_023435	1000	331	0	0	Ai	antisense to gene(s) PMM0642;
611684	+	TSS_007002	1000	179	0	0	I	within gene(s) PMM0642;
611699	-	TSS_023436	1000	268	0	4	Ai	antisense to gene(s) PMM0642;
611753	+	TSS_007006	1000	195	0	2	I	within gene(s) PMM0642;
611810	+	TSS_007011	1000	117	0	6	I	within gene(s) PMM0642;
611855	+	TSS_007015	1000	234	0	3	I	within gene(s) PMM0642;
611880	+	TSS_007017	1000	132	0	1	I	within gene(s) PMM0642;
611906	+	TSS_007024	1000	139	0	30	I	within gene(s) PMM0642;
612008	+	TSS_007038	1000	337	0	27	I	within gene(s) PMM0642;
612038	+	TSS_007045	1000	212	0	15	I	within gene(s) PMM0642;
612040	-	TSS_023441	1000	118	0	0	Ai	antisense to gene(s) PMM0642;
612065	+	TSS_007051	1000	122	0	3	IP	within gene(s) PMM0642; 236nt upstream of gene PMM0643;
612104	+	TSS_007062	1000	217	0	33	IP	within gene(s) PMM0642; 197nt upstream of gene PMM0643;
614001	-	TSS_023464	1000	168	0	1	I	within gene(s) PMM0644;
614082	-	TSS_023467	1000	173	0	9	I	within gene(s) PMM0644;
614112	-	TSS_023471	1000	126	0	6	I	within gene(s) PMM0644;
614523	-	TSS_023474	1000	110	0	0	I	within gene(s) PMM0644;
614781	-	TSS_023480	1000	469	0	2	P	15nt upstream of gene PMM0644;
617157	-	TSS_023494	1000	6990	0	9	Ai	antisense to gene(s) PMM0646;
618720	+	TSS_007100	1000	368	0	8	Ai	antisense to gene(s) PMM0648;
618923	-	TSS_023500	1000	133	0	0	I	within gene(s) PMM0648;
618981	-	TSS_023501	1000	116	0	2	I	within gene(s) PMM0648;
619598	+	TSS_007103	1000	439	0	1	P	18nt upstream of gene PMM0649;
620989	-	TSS_023510	1000	4404	0	6	P	13nt upstream of gene PMM0651;
621700	-	TSS_023514	1000	396	0	0	P	156nt upstream of gene PMM0652;
624037	-	TSS_023525	1000	167	0	4	O	-
624976	+	TSS_007122	1000	353	0	4	I	within gene(s) PMM0658;
626301	+	TSS_007127	1000	449	0	0	I	within gene(s) PMM0659;
627516	+	TSS_007132	1000	531	0	0	O	-
627971	-	TSS_023537	1000	13861	0	3	O	-
628169	+	TSS_007135	1000	3674	0	2	O	-
628400	-	TSS_023542	1000	127	0	0	I	within gene(s) PMM0660;
628424	-	TSS_023548	1000	106	0	16	I	within gene(s) PMM0660;
628451	-	TSS_023552	1000	270	0	22	I	within gene(s) PMM0660;
628487	-	TSS_023561	1000	196	0	6	I	within gene(s) PMM0660;
628505	-	TSS_023566	1000	521	0	7	I	within gene(s) PMM0660;
628656	-	TSS_023570	1000	645	0	0	P	10nt upstream of gene PMM0660;
628980	-	TSS_023575	1000	186	0	48	I	within gene(s) PMM0661;
629258	-	TSS_023605	1000	127	0	12	I	within gene(s) PMM0661;
629355	-	TSS_023616	1000	233	0	4	I	within gene(s) PMM0661;
629367	-	TSS_023620	1000	273	0	4	I	within gene(s) PMM0661;
629577	-	TSS_023643	1000	432	0	48	I	within gene(s) PMM0661;
629637	-	TSS_023658	1000	363	0	27	I	within gene(s) PMM0661;
629655	-	TSS_023661	1000	181	0	11	I	within gene(s) PMM0661;
629664	+	TSS_007150	1000	145	0	9	Ai	antisense to gene(s) PMM0661;
629706	-	TSS_023668	1000	129	0	12	I	within gene(s) PMM0661;
629937	-	TSS_023698	1000	109	0	0	I	within gene(s) PMM0661;
630039	-	TSS_023710	1000	274	0	43	I	within gene(s) PMM0661;
630075	-	TSS_023714	1000	510	0	12	I	within gene(s) PMM0661;
630111	-	TSS_023718	1000	229	0	27	I	within gene(s) PMM0661;
630158	+	TSS_007164	1000	167	0	9	Ai	antisense to gene(s) PMM0661;
630171	-	TSS_023726	1000	124	0	0	I	within gene(s) PMM0661;
630201	-	TSS_023732	1000	383	0	27	I	within gene(s) PMM0661;
630225	-	TSS_023738	1000	152	0	0	I	within gene(s) PMM0661;
630243	-	TSS_023740	1000	266	0	15	I	within gene(s) PMM0661;
630270	-	TSS_023749	1000	331	0	38	I	within gene(s) PMM0661;
630318	-	TSS_023758	1000	139	0	6	I	within gene(s) PMM0661;
630339	-	TSS_023763	1000	159	0	3	I	within gene(s) PMM0661;
630363	-	TSS_023770	1000	780	0	51	I	within gene(s) PMM0661;
630426	-	TSS_023780	1000	126	0	9	I	within gene(s) PMM0661;
630465	-	TSS_023788	1000	260	0	18	I	within gene(s) PMM0661;
630486	-	TSS_023792	1000	107	0	18	I	within gene(s) PMM0661;
630525	-	TSS_023800	1000	102	0	3	I	within gene(s) PMM0661;
630554	+	TSS_007171	1000	203	0	0	Ai	antisense to gene(s) PMM0661;
630684	-	TSS_023813	1000	319	0	42	I	within gene(s) PMM0661;
630807	-	TSS_023827	1000	352	0	24	I	within gene(s) PMM0661;
630864	-	TSS_023839	1000	136	0	24	I	within gene(s) PMM0661;
630981	-	TSS_023850	1000	173	0	6	I	within gene(s) PMM0661;

631086	-	TSS_023856	1000	2081	0	0	I	within gene(s) PMM0661;
631116	-	TSS_023858	1000	7979	0	2	I	within gene(s) PMM0661;
631165	+	TSS_007178	1000	248	0	0	P	27nt upstream of gene PMM0662;
633583	+	TSS_007195	1000	493	0	1	P	23nt upstream of gene PMM0664;
633593	+	TSS_007196	1000	130	0	0	P	13nt upstream of gene PMM0664;
635224	-	TSS_023873	1000	160	0	3	IP	within gene(s) PMM0666; 40nt upstream of gene PMM0665;
635265	-	TSS_023875	1000	154	0	0	IP	within gene(s) PMM0666; 81nt upstream of gene PMM0665;
635882	+	TSS_007203	1000	118	0	2	P	25nt upstream of gene PMM0667;
638295	+	TSS_007225	1000	217	0	1	Ai	antisense to gene(s) PMM0670;
638760	-	TSS_023886	1000	105	0	3	IP	within gene(s) PMM0671; 217nt upstream of gene PMM0670;
642570	+	TSS_007231	1000	105	0	0	Ai	antisense to gene(s) PMM0674;
642971	-	TSS_023900	1000	104	0	0	P	49nt upstream of gene PMM0674;
643136	+	TSS_007234	1000	315	0	2	P	21nt upstream of gene PMM0675;
643669	+	TSS_007236	1000	667	0	1	P	25nt upstream of gene PMM0676;
644589	+	TSS_007256	1000	375	0	3	I	within gene(s) PMM0676;
644914	-	TSS_023909	1000	528	0	0	Ad	antisense to gene(s) PMM0676 (4nt downstream);
647282	-	TSS_023915	1000	2796	0	2	P	69nt upstream of gene PMM0678;
648438	+	TSS_007278	1000	145	0	0	Ai	antisense to gene(s) PMM0680;
649133	+	TSS_007281	1000	114	0	0	I	within gene(s) PMM0681;
649461	+	TSS_007283	1000	181	0	6	I	within gene(s) PMM0681;
651413	+	TSS_007287	1000	296	0	0	I	within gene(s) PMM0683;
652626	+	TSS_007292	1000	1570	0	0	O	-
653279	+	TSS_007298	1000	132	0	15	P	98nt upstream of gene PMM0685;
653294	+	TSS_007301	1000	295	0	18	P	83nt upstream of gene PMM0685;
653360	+	TSS_007317	1000	187	0	10	P	17nt upstream of gene PMM0685;
653400	-	TSS_023932	1000	740	0	0	Ai	antisense to gene(s) PMM0685;
653943	-	TSS_023935	1000	173	0	12	O	-
653980	-	TSS_023939	1000	26013	0	2	O	-
655603	-	TSS_023948	1000	3112	0	5	I	within gene(s) PMM0687;
655934	-	TSS_023955	1000	6550	0	2	O	-
656633	-	TSS_023962	1000	172	0	2	P	41nt upstream of gene PMM0688;
656661	-	TSS_023966	1000	107	0	0	P	69nt upstream of gene PMM0688;
656672	-	TSS_023967	1000	146	0	0	P	80nt upstream of gene PMM0688;
656679	-	TSS_023968	1000	518	0	0	P	87nt upstream of gene PMM0688;
656925	-	TSS_023974	1000	201	0	12	O	-
656940	-	TSS_023975	1000	116	0	2	O	-
657528	-	TSS_023980	1000	2243	0	2	O	-
657710	+	TSS_007327	1000	386	0	2	O	-
657998	+	TSS_007334	1000	289	0	6	O	-
659043	-	TSS_023990	1000	2099	0	3	P	15nt upstream of gene PMM0690;
659754	+	TSS_007341	1000	940	0	1	Ad	antisense to gene(s) PMM0691 (2nt downstream);
660094	-	TSS_023999	1000	353	0	6	P	133nt upstream of gene PMM0691;
660419	-	TSS_024002	1000	304	0	6	P	22nt upstream of gene PMM0692;
661103	-	TSS_024008	1000	199	0	2	I	within gene(s) PMM0693;
661646	+	TSS_007348	1000	117	0	0	I	within gene(s) PMM0694;
661982	+	TSS_007350	1000	285	0	4	O	-
663678	-	TSS_024014	1000	349	0	7	P	16nt upstream of gene PMM0697;
664058	-	TSS_024017	1000	164	0	0	I	within gene(s) PMM0698;
664076	-	TSS_024018	1000	131	0	0	I	within gene(s) PMM0698;
665165	+	TSS_007362	1000	1900	0	4	P	16nt upstream of gene PMM0699;
667292	+	TSS_007369	1000	235	0	2	P	14nt upstream of gene PMM0703;
668059	+	TSS_007376	1000	408	0	2	I	within gene(s) PMM0704;
668368	+	TSS_007377	1000	151	0	0	I	within gene(s) PMM0704;
669388	+	TSS_007384	1000	227	0	0	P	12nt upstream of gene PMM0705;
671507	-	TSS_024032	1000	103	0	0	I	within gene(s) PMM0707;
671615	-	TSS_024034	1000	158	0	0	I	within gene(s) PMM0707;
672523	-	TSS_024037	1000	103	0	0	I	within gene(s) PMM0708;
674151	+	TSS_007404	1000	1363	0	0	Ai	antisense to gene(s) PMM0709;
674830	-	TSS_024057	1000	362	0	1	I	within gene(s) PMM0709;
674981	+	TSS_007408	1000	154	0	0	Ai	antisense to gene(s) PMM0709;
675828	+	TSS_007412	1000	18132	0	6	P	26nt upstream of gene PMM0710;
675986	+	TSS_007423	1000	105	0	0	I	within gene(s) PMM0710;
676022	+	TSS_007424	1000	153	0	7	I	within gene(s) PMM0710;
676095	-	TSS_024065	1000	106	0	0	Ai	antisense to gene(s) PMM0710;
676283	+	TSS_007440	1000	171	0	0	I	within gene(s) PMM0710;
676460	+	TSS_007454	1000	121	0	0	I	within gene(s) PMM0710;
676471	-	TSS_024068	1000	105	0	6	Ai	antisense to gene(s) PMM0710;
676791	-	TSS_024071	1000	435	0	1	Ai	antisense to gene(s) PMM0710;
678373	+	TSS_007460	1000	190	0	0	Ai	antisense to gene(s) PMM0712;
678437	+	TSS_007463	1000	300	0	0	Ai	antisense to gene(s) PMM0712;
680440	-	TSS_024084	1000	533	0	0	P	15nt upstream of gene PMM0713;
680511	-	TSS_024085	1000	197	0	0	PAi	86nt upstream of gene PMM0713; antisense to gene(s) PMM0714;
680855	-	TSS_024086	1000	133	0	0	Ai	antisense to gene(s) PMM0714;
682685	-	TSS_024090	1000	200	0	0	P	0nt upstream of gene PMM0717;
683455	+	TSS_007474	1000	114	0	0	I	within gene(s) PMM0719;
685615	-	TSS_024093	1000	1115	0	5	P	13nt upstream of gene PMM0722;
686751	-	TSS_024096	1000	107	0	2	Ai	antisense to gene(s) PMM0723;

687667	+	TSS_007484	1000	603	0	1 IP	within gene(s) PMM0724; 61nt upstream of gene PMM0725;
688326	-	TSS_024104	1000	493	0	1 Ai	antisense to gene(s) PMM0725;
688353	+	TSS_007501	1000	417	0	2 I	within gene(s) PMM0725;
688629	+	TSS_007504	1000	205	0	0 O	-
688866	+	TSS_007507	1000	6883	0	2 O	-
689153	+	TSS_007514	1000	601	0	2 O	-
689536	-	TSS_024112	1000	112	0	11 I	within gene(s) PMM0726;
689593	-	TSS_024117	1000	217	0	10 P	17nt upstream of gene PMM0726;
696382	-	TSS_024127	1000	124	0	0 P	18nt upstream of gene PMM0732;
698300	-	TSS_024129	1000	557	0	3 P	15nt upstream of gene PMM0734;
699080	+	TSS_007525	1000	195	0	10 O	-
702227	-	TSS_024144	1000	290	0	2 P	16nt upstream of gene PMM0739;
702332	+	TSS_007534	1000	2785	0	5 P	11nt upstream of gene PMM0740;
702507	-	TSS_024146	1000	155	0	0 O	-
702516	-	TSS_024147	1000	716	0	0 O	-
703275	+	TSS_007538	1000	1307	0	3 P	17nt upstream of gene PMM0742;
703511	+	TSS_007551	1000	177	0	1 I	within gene(s) PMM0742;
703532	+	TSS_007556	1000	108	0	12 I	within gene(s) PMM0742;
703571	+	TSS_007560	1000	149	0	21 I	within gene(s) PMM0742;
703658	+	TSS_007566	1000	101	0	3 I	within gene(s) PMM0742;
704032	+	TSS_007571	1000	103	0	1 Ai	antisense to gene(s) PMM0743;
704207	+	TSS_007576	1000	229	0	0 Ai	antisense to gene(s) PMM0743;
704304	-	TSS_024163	1000	196	0	0 I	within gene(s) PMM0743;
704315	+	TSS_007581	1000	203	0	4 Ai	antisense to gene(s) PMM0743;
704361	-	TSS_024165	1000	157	0	0 I	within gene(s) PMM0743;
704397	-	TSS_024172	1000	240	0	36 I	within gene(s) PMM0743;
704440	-	TSS_024180	1000	179	0	8 I	within gene(s) PMM0743;
704459	-	TSS_024188	1000	321	0	0 I	within gene(s) PMM0743;
704526	-	TSS_024191	1000	176	0	7 I	within gene(s) PMM0743;
704643	-	TSS_024203	1000	111	0	3 I	within gene(s) PMM0743;
704789	+	TSS_007586	1000	107	0	0 Ai	antisense to gene(s) PMM0743;
704790	-	TSS_024218	1000	202	0	20 I	within gene(s) PMM0743;
704823	-	TSS_024225	1000	273	0	17 I	within gene(s) PMM0743;
704949	+	TSS_007588	1000	130	0	1 Ai	antisense to gene(s) PMM0743;
705087	-	TSS_024240	1000	150	0	9 I	within gene(s) PMM0743;
705171	-	TSS_024252	1000	142	0	36 I	within gene(s) PMM0743;
705461	-	TSS_024273	1000	245	0	0 I	within gene(s) PMM0743;
705815	-	TSS_024288	1000	795	0	1 P	23nt upstream of gene PMM0743;
708310	+	TSS_007600	1000	1565	0	2 Ai	antisense to gene(s) PMM0746;
708607	-	TSS_024294	1000	712	0	1 P	14nt upstream of gene PMM0746;
709324	+	TSS_007603	1000	118	0	0 IP	within gene(s) PMM0747; 32nt upstream of gene PMM0748;
710247	+	TSS_007609	1000	394	0	1 IP	within gene(s) PMM0748; 23nt upstream of gene PMM0749;
710300	+	TSS_007611	1000	110	0	1 I	within gene(s) PMM0749;
710413	+	TSS_007614	1000	106	0	1 I	within gene(s) PMM0749;
711092	+	TSS_007622	1000	168	0	0 I	within gene(s) PMM0749;
711431	-	TSS_024303	1000	144	0	0 Ai	antisense to gene(s) PMM0749;
711539	+	TSS_007624	1000	2632	0	2 I	within gene(s) PMM0750;
711829	-	TSS_024306	1000	992	0	6 Ai	antisense to gene(s) PMM0750;
712163	+	TSS_007628	1000	1014	0	0 IP	within gene(s) PMM0750; 42nt upstream of gene PMM0751;
712186	+	TSS_007630	1000	759	0	4 P	19nt upstream of gene PMM0751;
713413	+	TSS_007648	1000	207	0	13 P	65nt upstream of gene PMM0753;
713700	+	TSS_007661	1000	482	0	17 I	within gene(s) PMM0753;
713805	+	TSS_007667	1000	140	0	0 I	within gene(s) PMM0753;
713922	+	TSS_007677	1000	388	0	54 I	within gene(s) PMM0753;
713997	+	TSS_007687	1000	101	0	0 IP	within gene(s) PMM0753; 230nt upstream of gene PMM0754;
714097	+	TSS_007698	1000	936	0	21 IP	within gene(s) PMM0753; 130nt upstream of gene PMM0754;
714106	-	TSS_024320	1000	110	0	1 Ai	antisense to gene(s) PMM0753;
714137	-	TSS_024322	1000	101	0	0 Ai	antisense to gene(s) PMM0753;
714683	+	TSS_007717	1000	115	0	0 IP	within gene(s) PMM0754; 206nt upstream of gene PMM0755;
714804	-	TSS_024329	1000	120	0	0 Ai	antisense to gene(s) PMM0754;
716611	+	TSS_007720	1000	133	0	0 I	within gene(s) PMM0756;
719033	+	TSS_007728	1000	150	0	6 I	within gene(s) PMM0757;
719060	+	TSS_007729	1000	115	0	2 I	within gene(s) PMM0757;
720208	+	TSS_007735	1000	181	0	0 Ai	antisense to gene(s) PMM0758;
720552	-	TSS_024377	1000	269	0	18 I	within gene(s) PMM0758;
720569	+	TSS_007740	1000	224	0	0 Ai	antisense to gene(s) PMM0758;
720579	-	TSS_024381	1000	107	0	0 I	within gene(s) PMM0758;
720641	-	TSS_024385	1000	701	0	10 I	within gene(s) PMM0758;
720675	-	TSS_024393	1000	141	0	15 I	within gene(s) PMM0758;
720702	-	TSS_024396	1000	225	0	7 I	within gene(s) PMM0758;
721555	-	TSS_024406	1000	140	0	0 Ai	antisense to gene(s) PMM0759;
723439	-	TSS_024414	1000	131	0	15 I	within gene(s) PMM0760;
723520	-	TSS_024433	1000	129	0	19 I	within gene(s) PMM0760;
723718	-	TSS_024451	1000	165	0	18 I	within gene(s) PMM0760;
723763	-	TSS_024467	1000	1271	0	51 I	within gene(s) PMM0760;
723796	-	TSS_024475	1000	108	0	12 I	within gene(s) PMM0760;
723823	-	TSS_024479	1000	196	0	39 I	within gene(s) PMM0760;

723871	-	TSS_024487	1000	150	0	1	I	within gene(s) PMM0760;
723880	-	TSS_024488	1000	110	0	0	I	within gene(s) PMM0760;
723907	-	TSS_024491	1000	156	0	9	I	within gene(s) PMM0760;
723931	-	TSS_024494	1000	106	0	0	I	within gene(s) PMM0760;
723979	-	TSS_024498	1000	136	0	3	I	within gene(s) PMM0760;
723994	-	TSS_024502	1000	172	0	6	I	within gene(s) PMM0760;
724072	-	TSS_024515	1000	125	0	54	I	within gene(s) PMM0760;
724249	-	TSS_024534	1000	130	0	21	I	within gene(s) PMM0760;
724284	-	TSS_024542	1000	249	0	17	I	within gene(s) PMM0760;
724300	-	TSS_024545	1000	179	0	12	I	within gene(s) PMM0760;
724348	-	TSS_024553	1000	182	0	12	I	within gene(s) PMM0760;
724366	-	TSS_024556	1000	115	0	13	I	within gene(s) PMM0760;
724438	-	TSS_024563	1000	280	0	24	I	within gene(s) PMM0760;
724578	-	TSS_024569	1000	5739	0	2	P	20nt upstream of gene PMM0760;
725488	+	TSS_007756	1000	382	0	0	P	25nt upstream of gene PMM0762;
725918	+	TSS_007771	1000	105	0	6	I	within gene(s) PMM0762;
726095	+	TSS_007780	1000	141	0	27	I	within gene(s) PMM0762;
728301	-	TSS_024582	1000	371	0	0	Ai	antisense to gene(s) PMM0764;
729981	-	TSS_024618	1000	249	0	8	I	within gene(s) PMM0766;
730261	-	TSS_024623	1000	1033	0	5	P	15nt upstream of gene PMM0766;
730331	+	TSS_007858	1000	707	0	1	P	103nt upstream of gene PMM0767;
730435	+	TSS_007859	1000	1072	0	0	I	within gene(s) PMM0767;
730737	+	TSS_007875	1000	204	0	4	I	within gene(s) PMM0767;
730749	+	TSS_007877	1000	187	0	0	I	within gene(s) PMM0767;
730925	+	TSS_007889	1000	103	0	1	I	within gene(s) PMM0767;
730965	+	TSS_007893	1000	103	0	15	I	within gene(s) PMM0767;
731037	+	TSS_007904	1000	176	0	9	I	within gene(s) PMM0767;
731067	+	TSS_007913	1000	186	0	18	I	within gene(s) PMM0767;
731240	-	TSS_024634	1000	119	0	0	Ai	antisense to gene(s) PMM0767;
732872	+	TSS_007931	1000	1997	0	6	P	21nt upstream of gene PMM0769;
733079	+	TSS_007936	1000	136	0	1	I	within gene(s) PMM0769;
733157	+	TSS_007942	1000	110	0	0	I	within gene(s) PMM0769;
733214	-	TSS_024644	1000	130	0	0	Ai	antisense to gene(s) PMM0769;
733226	-	TSS_024645	1000	323	0	1	Ai	antisense to gene(s) PMM0769;
733478	+	TSS_007960	1000	120	0	6	I	within gene(s) PMM0769;
733779	+	TSS_007976	1000	114	0	16	I	within gene(s) PMM0769;
733808	+	TSS_007978	1000	119	0	3	I	within gene(s) PMM0769;
733838	+	TSS_007989	1000	192	0	18	I	within gene(s) PMM0769;
733859	+	TSS_007991	1000	133	0	7	I	within gene(s) PMM0769;
733922	+	TSS_008001	1000	119	0	9	I	within gene(s) PMM0769;
733937	+	TSS_008003	1000	132	0	0	I	within gene(s) PMM0769;
734059	-	TSS_024649	1000	463	0	1	Ai	antisense to gene(s) PMM0769;
734072	-	TSS_024651	1000	270	0	5	Ai	antisense to gene(s) PMM0769;
734116	-	TSS_024656	1000	313	0	6	Ai	antisense to gene(s) PMM0769;
734136	+	TSS_008015	1000	125	0	9	IP	within gene(s) PMM0769; 151nt upstream of gene PMM0770;
734269	+	TSS_008019	1000	4117	0	4	P	18nt upstream of gene PMM0770;
736446	+	TSS_008035	1000	188	0	5	P	15nt upstream of gene PMM0772;
737774	-	TSS_024669	1000	132	0	6	I	within gene(s) PMM0774;
737785	-	TSS_024671	1000	191	0	0	I	within gene(s) PMM0774;
737882	-	TSS_024677	1000	217	0	0	I	within gene(s) PMM0774;
738561	+	TSS_008048	1000	284	0	0	Ai	antisense to gene(s) PMM0774;
738616	-	TSS_024691	1000	274	0	4	I	within gene(s) PMM0774;
738854	-	TSS_024698	1000	139	0	6	I	within gene(s) PMM0774;
738964	-	TSS_024702	1000	251	0	1	I	within gene(s) PMM0774;
739095	-	TSS_024706	1000	1750	0	2	P	13nt upstream of gene PMM0774;
739410	-	TSS_024715	1000	1082	0	10	P	14nt upstream of gene PMM0775;
740068	+	TSS_008053	1000	810	0	2	P	38nt upstream of gene PMM0777;
740232	+	TSS_008064	1000	132	0	39	I	within gene(s) PMM0777;
741992	+	TSS_008085	1000	1246	0	1	P	15nt upstream of gene PMM0779;
742370	+	TSS_008094	1000	133	0	5	I	within gene(s) PMM0780;
743202	-	TSS_024738	1000	172	0	3	I	within gene(s) PMM0781;
743280	-	TSS_024758	1000	435	0	54	I	within gene(s) PMM0781;
743304	-	TSS_024762	1000	428	0	6	I	within gene(s) PMM0781;
743322	-	TSS_024765	1000	153	0	6	I	within gene(s) PMM0781;
743364	-	TSS_024774	1000	810	0	42	I	within gene(s) PMM0781;
743421	-	TSS_024791	1000	590	0	75	I	within gene(s) PMM0781;
743484	-	TSS_024809	1000	171	0	15	I	within gene(s) PMM0781;
743520	-	TSS_024814	1000	252	0	12	I	within gene(s) PMM0781;
743565	-	TSS_024818	1000	180	0	6	I	within gene(s) PMM0781;
743607	-	TSS_024822	1000	135	0	6	I	within gene(s) PMM0781;
743622	-	TSS_024824	1000	448	0	1	I	within gene(s) PMM0781;
743631	-	TSS_024825	1000	196	0	6	I	within gene(s) PMM0781;
743661	-	TSS_024834	1000	340	0	18	I	within gene(s) PMM0781;
743682	-	TSS_024838	1000	188	0	15	I	within gene(s) PMM0781;
743706	-	TSS_024842	1000	102	7.00E-13	0	I	within gene(s) PMM0781;
743715	-	TSS_024844	1000	178	0	4	I	within gene(s) PMM0781;
743745	-	TSS_024847	1000	356	0	1	I	within gene(s) PMM0781;

743763	-	TSS_024848	1000	155	0	12 I	within gene(s) PMM0781;
743799	-	TSS_024855	1000	1280	0	45 I	within gene(s) PMM0781;
743847	-	TSS_024867	1000	132	0	6 I	within gene(s) PMM0781;
743859	-	TSS_024871	1000	236	0	27 I	within gene(s) PMM0781;
743904	-	TSS_024878	1000	117	0	3 I	within gene(s) PMM0781;
743916	-	TSS_024881	1000	303	0	4 I	within gene(s) PMM0781;
743937	-	TSS_024884	1000	140	0	0 I	within gene(s) PMM0781;
743967	-	TSS_024890	1000	101	0	0 I	within gene(s) PMM0781;
744091	-	TSS_024893	1000	15402	0	3 P	43nt upstream of gene PMM0781;
745876	+	TSS_008111	1000	212	0	1 Ai	antisense to gene(s) PMM0784;
746288	-	TSS_024926	1000	287	0	28 I	within gene(s) PMM0784;
746583	-	TSS_024944	1000	1274	0	9 P	17nt upstream of gene PMM0784;
747217	-	TSS_024960	1000	214	0	5 I	within gene(s) PMM0785;
747301	-	TSS_024972	1000	105	0	21 I	within gene(s) PMM0785;
747387	-	TSS_024983	1000	490	0	18 I	within gene(s) PMM0785;
749173	-	TSS_025009	1000	493	0	4 I	within gene(s) PMM0787;
751730	-	TSS_025016	1000	118	0	7 I	within gene(s) PMM0790;
752029	-	TSS_025036	1000	253	0	8 P	25nt upstream of gene PMM0790;
752047	+	TSS_008135	1000	1429	0	12 Ai	antisense to gene(s) PMM0791;
752129	+	TSS_008136	1000	172	0	0 Ai	antisense to gene(s) PMM0791;
755386	-	TSS_025043	1000	1237	0	3 IP	within gene(s) PMM0795; 114nt upstream of gene PMM0794;
757822	-	TSS_025056	1000	190	0	1 P	66nt upstream of gene PMM0796;
759405	+	TSS_008147	1000	115	0	2 P	16nt upstream of gene PMM0799;
760249	-	TSS_025067	1000	129	0	11 P	17nt upstream of gene PMM0800;
760930	-	TSS_025073	1000	153	0	2 P	14nt upstream of gene PMM0801;
762992	+	TSS_008157	1000	2429	0	1 P	16nt upstream of gene PMM0804;
763053	+	TSS_008165	1000	2047	0	48 I	within gene(s) PMM0804;
763152	+	TSS_008190	1000	319	0	48 I	within gene(s) PMM0804;
763188	+	TSS_008199	1000	226	0	45 I	within gene(s) PMM0804;
763237	+	TSS_008208	1000	106	0	4 I	within gene(s) PMM0804;
763266	+	TSS_008214	1000	328	0	12 I	within gene(s) PMM0804;
763290	+	TSS_008219	1000	164	0	21 I	within gene(s) PMM0804;
764067	+	TSS_008238	1000	501	0	0 Ai	antisense to gene(s) PMM0806;
764468	-	TSS_025087	1000	250	0	3 P	26nt upstream of gene PMM0806;
768634	-	TSS_025095	1000	1841	0	1 P	16nt upstream of gene PMM0810;
769201	-	TSS_025099	1000	303	0	0 O	-
770293	-	TSS_025105	1000	141	0	2 O	-
771730	-	TSS_025110	1000	334	0	0 P	22nt upstream of gene PMM0814;
771982	+	TSS_008252	1000	171	0	5 O	-
772207	-	TSS_025113	1000	885	0	0 O	-
773190	-	TSS_025123	1000	457	0	2 I	within gene(s) PMM0815;
773277	-	TSS_025130	1000	121	0	12 IP	within gene(s) PMM0816; 84nt upstream of gene PMM0815;
773322	-	TSS_025143	1000	391	0	45 IP	within gene(s) PMM0816; 129nt upstream of gene PMM0815;
773382	-	TSS_025155	1000	596	0	3 IP	within gene(s) PMM0816; 189nt upstream of gene PMM0815;
773597	-	TSS_025168	1000	186	0	1 IP	within gene(s) PMM0817; 113nt upstream of gene PMM0816;
773663	-	TSS_025175	1000	192	0	2 IP	within gene(s) PMM0817; 179nt upstream of gene PMM0816;
773710	-	TSS_025179	1000	127	0	11 IP	within gene(s) PMM0818; 17nt upstream of gene PMM0817;
773767	-	TSS_025186	1000	112	0	0 IP	within gene(s) PMM0818; 74nt upstream of gene PMM0817;
773776	-	TSS_025187	1000	111	0	4 IP	within gene(s) PMM0818; 83nt upstream of gene PMM0817;
773791	-	TSS_025192	1000	517	0	4 IP	within gene(s) PMM0818; 98nt upstream of gene PMM0817;
773820	-	TSS_025193	1000	890	0	1 P	20nt upstream of gene PMM0818;
774773	-	TSS_025204	1000	9023	0	3 P	18nt upstream of gene PMM0819;
775070	-	TSS_025208	1000	155	0	0 P	49nt upstream of gene PMM0820;
775810	-	TSS_025210	1000	467	0	1 O	-
776220	+	TSS_008266	1000	105	0	0 O	-
776471	-	TSS_025213	1000	252	0	4 O	-
777149	-	TSS_025220	1000	502	0	0 I	within gene(s) PMM0821;
778499	-	TSS_025226	1000	120	0	0 IP	within gene(s) PMM0824; 166nt upstream of gene PMM0823;
778980	-	TSS_025241	1000	144	0	2 P	19nt upstream of gene PMM0824;
779062	-	TSS_025244	1000	108	0	1 P	101nt upstream of gene PMM0824;
779674	-	TSS_025245	1000	192	0	0 Ai	antisense to gene(s) PMM0825;
780730	-	TSS_025248	1000	248	0	3 Ai	antisense to gene(s) PMM0825;
780946	+	TSS_008292	1000	146	0	0 I	within gene(s) PMM0825;
784311	+	TSS_008331	1000	140	0	0 PAi	76nt upstream of gene PMM0828; antisense to gene(s) PMM0827;
784601	+	TSS_008332	1000	481	0	3 P	14nt upstream of gene PMM0829;
784835	+	TSS_008336	1000	172	0	0 I	within gene(s) PMM0829;
786463	-	TSS_025280	1000	129	0	9 I	within gene(s) PMM0831;
786571	-	TSS_025294	1000	525	0	3 I	within gene(s) PMM0831;
786861	-	TSS_025313	1000	104	0	0 I	within gene(s) PMM0831;
786877	-	TSS_025315	1000	115	0	12 I	within gene(s) PMM0831;
786988	-	TSS_025329	1000	103	0	6 I	within gene(s) PMM0831;
787168	-	TSS_025341	1000	157	0	42 I	within gene(s) PMM0831;
787324	-	TSS_025363	1000	158	0	18 I	within gene(s) PMM0831;
787582	-	TSS_025387	1000	111	0	13 I	within gene(s) PMM0831;
787909	-	TSS_025407	1000	598	0	36 I	within gene(s) PMM0831;
787995	+	TSS_008352	1000	110	0	0 Ai	antisense to gene(s) PMM0831;
788167	-	TSS_025430	1000	298	0	6 I	within gene(s) PMM0831;

788179	-	TSS_025431	1000	159	0	1	I	within gene(s) PMM0831;
788467	-	TSS_025466	1000	138	0	22	I	within gene(s) PMM0831;
788563	-	TSS_025471	1000	362	0	15	I	within gene(s) PMM0831;
788602	-	TSS_025477	1000	183	0	3	I	within gene(s) PMM0831;
788713	-	TSS_025485	1000	156	0	0	I	within gene(s) PMM0831;
788890	-	TSS_025490	1000	143	0	0	I	within gene(s) PMM0831;
788923	-	TSS_025494	1000	129	0	18	I	within gene(s) PMM0831;
789088	-	TSS_025512	1000	160	0	21	I	within gene(s) PMM0831;
789127	-	TSS_025516	1000	153	0	0	I	within gene(s) PMM0831;
789218	+	TSS_008363	1000	201	0	1	Ai	antisense to gene(s) PMM0831;
789262	-	TSS_025532	1000	154	0	6	I	within gene(s) PMM0831;
789502	-	TSS_025556	1000	108	0	9	I	within gene(s) PMM0831;
789844	-	TSS_025569	1000	312	0	12	I	within gene(s) PMM0831;
790790	+	TSS_008373	1000	102	0	5	I	within gene(s) PMM0832;
796520	+	TSS_008384	1000	466	0	1	Ai	antisense to gene(s) PMM0839;
803504	-	TSS_025645	1000	256	0	36	I	within gene(s) PMM0844;
803921	-	TSS_025673	1000	431	0	33	I	within gene(s) PMM0844;
803957	-	TSS_025681	1000	171	0	18	I	within gene(s) PMM0844;
804020	-	TSS_025691	1000	192	0	6	I	within gene(s) PMM0844;
804038	-	TSS_025694	1000	157	0	21	I	within gene(s) PMM0844;
804065	-	TSS_025699	1000	207	0	21	I	within gene(s) PMM0844;
804101	-	TSS_025709	1000	130	0	10	I	within gene(s) PMM0844;
804143	-	TSS_025715	1000	136	0	30	I	within gene(s) PMM0844;
804170	-	TSS_025720	1000	164	0	18	I	within gene(s) PMM0844;
804298	-	TSS_025729	1000	2075	0	2	P	14nt upstream of gene PMM0844;
804935	-	TSS_025732	1000	103	0	0	P	16nt upstream of gene PMM0845;
807762	-	TSS_025746	1000	145	0	2	P	29nt upstream of gene PMM0847;
808842	+	TSS_008413	1000	6663	0	6	P	117nt upstream of gene PMM0851;
810059	-	TSS_025754	1000	266	0	3	IAd	within gene(s) PMM0853; antisense to gene(s) PMM0852 (24nt downstream);
810098	-	TSS_025762	1000	175	0	13	I	within gene(s) PMM0853;
810125	-	TSS_025765	1000	121	0	0	I	within gene(s) PMM0853;
810137	-	TSS_025766	1000	215	0	0	I	within gene(s) PMM0853;
810149	-	TSS_025768	1000	290	0	12	I	within gene(s) PMM0853;
810173	-	TSS_025777	1000	1809	0	21	I	within gene(s) PMM0853;
810194	-	TSS_025782	1000	254	0	0	I	within gene(s) PMM0853;
810209	-	TSS_025785	1000	361	0	6	I	within gene(s) PMM0853;
811735	-	TSS_025793	1000	676	0	0	Ai	antisense to gene(s) PMM0854;
813130	+	TSS_008436	1000	182	0	1	Ai	antisense to gene(s) PMM0856;
813200	-	TSS_025807	1000	120	0	21	I	within gene(s) PMM0856;
813260	-	TSS_025820	1000	274	0	39	I	within gene(s) PMM0856;
813281	-	TSS_025826	1000	347	0	60	I	within gene(s) PMM0856;
813356	-	TSS_025839	1000	157	0	0	I	within gene(s) PMM0856;
813383	-	TSS_025843	1000	105	0	6	I	within gene(s) PMM0856;
813437	-	TSS_025850	1000	233	0	18	I	within gene(s) PMM0856;
813572	-	TSS_025860	1000	6522	0	8	P	45nt upstream of gene PMM0856;
813592	-	TSS_025863	1000	2128	0	1	P	65nt upstream of gene PMM0856;
814913	+	TSS_008444	1000	1730	0	2	O	-
815258	-	TSS_025876	1000	115	0	0	O	-
815652	-	TSS_025877	1000	115	0	0	O	-
816028	+	TSS_008449	1000	102	0	0	I	within gene(s) PMM0858;
816976	+	TSS_008451	1000	1183	0	1	O	-
817333	+	TSS_008454	1000	1543	0	3	O	-
818060	-	TSS_025880	1000	133	0	0	O	-
818781	+	TSS_008460	1000	127	0	0	O	-
819069	+	TSS_008462	1000	139	0	2	P	64nt upstream of gene PMM0861;
819084	+	TSS_008465	1000	161	0	6	P	49nt upstream of gene PMM0861;
819103	+	TSS_008469	1000	134	0	11	P	30nt upstream of gene PMM0861;
819132	+	TSS_008476	1000	776	0	15	P	1nt upstream of gene PMM0861;
819403	-	TSS_025892	1000	243	0	2	O	-
820064	-	TSS_025897	1000	123	0	0	O	-
821702	-	TSS_025916	1000	130	0	5	P	16nt upstream of gene PMM0864;
824596	-	TSS_025949	1000	108	0	10	P	21nt upstream of gene PMM0867;
826236	-	TSS_025961	1000	418	0	7	IP	within gene(s) PMM0870; 156nt upstream of gene PMM0869;
827556	-	TSS_025966	1000	482	0	0	Ai	antisense to gene(s) PMM0871;
831934	+	TSS_008508	1000	113	0	0	Ai	antisense to gene(s) PMM0875;
832333	+	TSS_008510	1000	827	0	3	P	14nt upstream of gene PMM0876;
832360	-	TSS_025980	1000	162	0	0	PAi	122nt upstream of gene PMM0875; antisense to gene(s) PMM0876;
832529	-	TSS_025981	1000	188	0	0	Ai	antisense to gene(s) PMM0876;
834878	-	TSS_025990	1000	1037	0	3	I	within gene(s) PMM0877;
835327	+	TSS_008520	1000	108	0	5	Ai	antisense to gene(s) PMM0877;
836569	+	TSS_008524	1000	107	0	0	Ai	antisense to gene(s) PMM0878;
836586	-	TSS_025998	1000	125	0	3	I	within gene(s) PMM0878;
836727	-	TSS_026009	1000	184	0	6	I	within gene(s) PMM0878;
837146	-	TSS_026012	1000	768	0	2	P	38nt upstream of gene PMM0878;
840244	-	TSS_026019	1000	182	0	2	Ai	antisense to gene(s) PMM0879;
841241	-	TSS_026025	1000	251	0	0	I	within gene(s) PMM0880;
842149	-	TSS_026029	1000	188	0	0	I	within gene(s) PMM0881;

844385	+	TSS_008541	1000	122	0	5	Ai	antisense to gene(s) PMM0883;
844688	-	TSS_026051	1000	338	0	0	P	6nt upstream of gene PMM0883;
844697	-	TSS_026053	1000	4343	0	5	P	15nt upstream of gene PMM0883;
845195	-	TSS_026058	1000	123	0	3	Ai	antisense to gene(s) PMM0884;
852542	-	TSS_026073	1000	127	0	3	I	within gene(s) PMM0890;
855315	+	TSS_008565	1000	119	0	2	P	49nt upstream of gene PMM0893;
855371	+	TSS_008567	1000	167	0	2	I	within gene(s) PMM0893;
856124	-	TSS_026098	1000	102	0	0	Ai	antisense to gene(s) PMM0893;
857107	+	TSS_008578	1000	114	0	1	Ai	antisense to gene(s) PMM0894;
857390	-	TSS_026115	1000	249	0	39	I	within gene(s) PMM0894;
857432	-	TSS_026126	1000	348	0	12	I	within gene(s) PMM0894;
857453	-	TSS_026129	1000	103	0	3	I	within gene(s) PMM0894;
857485	-	TSS_026131	1000	525	0	0	I	within gene(s) PMM0894;
857549	-	TSS_026133	1000	7242	0	3	P	18nt upstream of gene PMM0894;
858604	-	TSS_026149	1000	192	0	0	I	within gene(s) PMM0896;
858648	-	TSS_026153	1000	211	0	10	I	within gene(s) PMM0896;
858686	-	TSS_026156	1000	554	0	0	I	within gene(s) PMM0896;
858957	-	TSS_026158	1000	665	0	2	IP	within gene(s) PMM0897; 21nt upstream of gene PMM0896;
859899	+	TSS_008588	1000	603	0	0	Ai	antisense to gene(s) PMM0897;
860364	+	TSS_008592	1000	121	0	0	Ai	antisense to gene(s) PMM0897;
860406	-	TSS_026180	1000	1911	0	2	I	within gene(s) PMM0897;
860930	-	TSS_026185	1000	225	0	0	P	13nt upstream of gene PMM0897;
860947	-	TSS_026186	1000	103	0	0	P	30nt upstream of gene PMM0897;
860980	-	TSS_026187	1000	477	0	2	P	63nt upstream of gene PMM0897;
862187	+	TSS_008603	1000	200	0	6	I	within gene(s) PMM0899;
862363	+	TSS_008606	1000	115	0	3	IP	within gene(s) PMM0899; 168nt upstream of gene PMM0900;
862487	-	TSS_026192	1000	207	0	0	Ai	antisense to gene(s) PMM0899;
863809	+	TSS_008610	1000	1129	0	2	P	23nt upstream of gene PMM0901;
863967	+	TSS_008617	1000	163	0	6	I	within gene(s) PMM0901;
864201	+	TSS_008635	1000	113	0	1	I	within gene(s) PMM0901;
864270	+	TSS_008644	1000	181	0	9	I	within gene(s) PMM0901;
864531	+	TSS_008650	1000	150	0	12	I	within gene(s) PMM0901;
865761	+	TSS_008675	1000	488	0	1	P	15nt upstream of gene PMM0902;
867262	+	TSS_008680	1000	126	0	15	I	within gene(s) PMM0906;
867288	+	TSS_008685	1000	1638	0	1	I	within gene(s) PMM0906;
867467	-	TSS_026210	1000	212	0	0	Ai	antisense to gene(s) PMM0906;
867687	+	TSS_008699	1000	268	0	0	Ai	antisense to gene(s) PMM0907;
867708	+	TSS_008700	1000	166	0	6	Ai	antisense to gene(s) PMM0907;
867831	+	TSS_008702	1000	144	0	0	Ai	antisense to gene(s) PMM0907;
868162	-	TSS_026224	1000	117	0	0	I	within gene(s) PMM0907;
869052	+	TSS_008714	1000	191	0	7	Ai	antisense to gene(s) PMM0907;
869183	+	TSS_008719	1000	103	0	0	Ai	antisense to gene(s) PMM0907;
869213	-	TSS_026262	1000	113	0	1	I	within gene(s) PMM0907;
869447	-	TSS_026269	1000	9260	0	5	P	16nt upstream of gene PMM0907;
869708	+	TSS_008721	1000	212	0	0	I	within gene(s) PMM0908;
870149	+	TSS_008725	1000	499	0	2	I	within gene(s) PMM0908;
871669	+	TSS_008734	1000	150	0	1	P	16nt upstream of gene PMM0910;
872329	-	TSS_026283	1000	119	0	1	P	33nt upstream of gene PMM0911;
872338	+	TSS_008739	1000	130	0	2	P	20nt upstream of gene PMM0912;
872359	+	TSS_008740	1000	106	0	0	I	within gene(s) PMM0912;
873315	+	TSS_008763	1000	163	0	4	I	within gene(s) PMM0912;
873612	+	TSS_008774	1000	544	0	9	I	within gene(s) PMM0912;
879143	+	TSS_008797	1000	141	0	0	Ai	antisense to gene(s) PMM0917;
881167	-	TSS_026315	1000	1688	0	2	P	15nt upstream of gene PMM0919;
881330	+	TSS_008809	1000	256	0	1	P	37nt upstream of gene PMM0920;
881367	+	TSS_008810	1000	227	0	0	P	0nt upstream of gene PMM0920;
881514	+	TSS_008823	1000	281	0	18	I	within gene(s) PMM0920;
881556	+	TSS_008829	1000	120	0	15	I	within gene(s) PMM0920;
881574	+	TSS_008830	1000	170	0	0	I	within gene(s) PMM0920;
881583	+	TSS_008831	1000	152	0	3	I	within gene(s) PMM0920;
881607	+	TSS_008836	1000	214	0	12	I	within gene(s) PMM0920;
881640	+	TSS_008842	1000	402	0	78	I	within gene(s) PMM0920;
881724	+	TSS_008868	1000	286	0	69	I	within gene(s) PMM0920;
881796	+	TSS_008885	1000	191	0	0	I	within gene(s) PMM0920;
881805	+	TSS_008886	1000	194	0	6	I	within gene(s) PMM0920;
881833	+	TSS_008891	1000	271	0	4	I	within gene(s) PMM0920;
881871	+	TSS_008901	1000	603	0	30	I	within gene(s) PMM0920;
881883	+	TSS_008903	1000	191	0	0	I	within gene(s) PMM0920;
881904	+	TSS_008906	1000	328	0	9	I	within gene(s) PMM0920;
881916	+	TSS_008908	1000	199	0	3	I	within gene(s) PMM0920;
881949	+	TSS_008915	1000	276	0	27	I	within gene(s) PMM0920;
881967	+	TSS_008918	1000	154	0	15	I	within gene(s) PMM0920;
881994	+	TSS_008926	1000	366	0	33	I	within gene(s) PMM0920;
882081	+	TSS_008948	1000	203	0	30	I	within gene(s) PMM0920;
882096	+	TSS_008950	1000	140	0	15	I	within gene(s) PMM0920;
882142	+	TSS_008962	1000	251	0	30	I	within gene(s) PMM0920;
882159	+	TSS_008967	1000	178	0	15	I	within gene(s) PMM0920;

882186	-	TSS_026327	1000	499	0	0	Ai	antisense to gene(s) PMM0920;
882218	-	TSS_026328	1000	303	0	1	Ai	antisense to gene(s) PMM0920;
882219	+	TSS_008978	1000	161	0	28	I	within gene(s) PMM0920;
882252	+	TSS_008983	1000	152	0	6	I	within gene(s) PMM0920;
882256	-	TSS_026330	1000	136	0	5	Ai	antisense to gene(s) PMM0920;
882270	+	TSS_008987	1000	244	0	12	I	within gene(s) PMM0920;
882294	+	TSS_008989	1000	137	0	0	I	within gene(s) PMM0920;
882330	+	TSS_008994	1000	175	0	12	I	within gene(s) PMM0920;
882363	+	TSS_009001	1000	392	0	18	I	within gene(s) PMM0920;
882384	+	TSS_009004	1000	144	0	6	I	within gene(s) PMM0920;
882426	+	TSS_009010	1000	594	0	24	I	within gene(s) PMM0920;
882468	+	TSS_009021	1000	373	0	18	I	within gene(s) PMM0920;
882489	+	TSS_009026	1000	315	0	15	I	within gene(s) PMM0920;
882540	+	TSS_009039	1000	230	0	51	I	within gene(s) PMM0920;
882579	+	TSS_009049	1000	281	0	24	I	within gene(s) PMM0920;
882673	-	TSS_026336	1000	283	0	5	Ai	antisense to gene(s) PMM0920;
883958	+	TSS_009069	1000	137	0	3	P	15nt upstream of gene PMM0922;
885534	-	TSS_026346	1000	147	0	0	PAi	91nt upstream of gene PMM0924; antisense to gene(s) PMM0925;
886557	+	TSS_009083	1000	1687	0	7	P	14nt upstream of gene PMM0926;
886578	+	TSS_009086	1000	252	0	4	I	within gene(s) PMM0926;
889213	+	TSS_009092	1000	217	0	0	Ai	antisense to gene(s) PMM0929;
889246	+	TSS_009093	1000	102	0	0	Ai	antisense to gene(s) PMM0929;
891097	+	TSS_009102	1000	105	0	0	Ai	antisense to gene(s) PMM0930;
891212	+	TSS_009104	1000	267	0	1	Ai	antisense to gene(s) PMM0930;
891599	-	TSS_026391	1000	4997	0	1	P	21nt upstream of gene PMM0930;
894754	+	TSS_009112	1000	193	0	0	P	45nt upstream of gene PMM0936;
899977	+	TSS_009126	1000	797	0	3	P	13nt upstream of gene PMM0941;
900012	+	TSS_009127	1000	140	0	0	I	within gene(s) PMM0941;
900029	+	TSS_009128	1000	135	0	1	I	within gene(s) PMM0941;
900041	+	TSS_009130	1000	544	0	1	I	within gene(s) PMM0941;
900096	+	TSS_009145	1000	401	0	43	I	within gene(s) PMM0941;
900197	+	TSS_009150	1000	134	0	0	IP	within gene(s) PMM0941; 153nt upstream of gene PMM0942;
900199	-	TSS_026406	1000	229	0	4	Ai	antisense to gene(s) PMM0941;
900229	-	TSS_026410	1000	231	0	0	Ai	antisense to gene(s) PMM0941;
900236	+	TSS_009156	1000	457	0	45	IP	within gene(s) PMM0941; 114nt upstream of gene PMM0942;
901047	+	TSS_009173	1000	19128	0	9	P	14nt upstream of gene PMM0943;
901115	+	TSS_009177	1000	176	0	18	IP	within gene(s) PMM0943; 225nt upstream of gene PMM0944;
901817	-	TSS_026416	1000	103	0	6	I	within gene(s) PMM0945;
905299	-	TSS_026442	1000	1895	0	3	P	14nt upstream of gene PMM0945;
905741	-	TSS_026448	1000	133	0	6	I	within gene(s) PMM0946;
905848	-	TSS_026452	1000	104	0	1	I	within gene(s) PMM0946;
906542	-	TSS_026464	1000	239	0	0	I	within gene(s) PMM0946;
906583	-	TSS_026466	1000	110	0	2	I	within gene(s) PMM0946;
907099	-	TSS_026468	1000	1645	0	2	P	6nt upstream of gene PMM0947;
911843	-	TSS_026494	1000	109	0	3	P	14nt upstream of gene PMM0953;
913287	+	TSS_009210	1000	170	0	0	I	within gene(s) PMM0954;
913344	+	TSS_009211	1000	313	0	0	I	within gene(s) PMM0954;
913440	+	TSS_009212	1000	510	0	0	IP	within gene(s) PMM0954; 246nt upstream of gene PMM0955;
913823	+	TSS_009215	1000	231	0	3	I	within gene(s) PMM0955;
914442	+	TSS_009218	1000	355	0	1	O	-
914689	-	TSS_026499	1000	720	0	2	O	-
915624	+	TSS_009222	1000	496	0	2	P	2nt upstream of gene PMM0957;
916151	-	TSS_026506	1000	208	0	10	O	-
916169	-	TSS_026507	1000	269	0	6	O	-
916221	-	TSS_026511	1000	322	0	2	O	-
916530	-	TSS_026514	1000	1277	0	1	P	16nt upstream of gene PMM0958;
918232	+	TSS_009231	1000	215	0	0	Ai	antisense to gene(s) PMM0960;
918384	-	TSS_026519	1000	108	0	0	IP	within gene(s) PMM0961; 35nt upstream of gene PMM0960;
918396	-	TSS_026520	1000	201	0	0	IP	within gene(s) PMM0961; 47nt upstream of gene PMM0960;
919141	-	TSS_026522	1000	230	0	0	I	within gene(s) PMM0961;
920008	+	TSS_009239	1000	104	0	1	PAi	153nt upstream of gene PMM0962; antisense to gene(s) PMM0961;
920682	-	TSS_026525	1000	229	0	0	Ai	antisense to gene(s) PMM0962;
922689	+	TSS_009264	1000	213	0	2	Ai	antisense to gene(s) PMM0963;
923723	-	TSS_026552	1000	928	0	0	I	within gene(s) PMM0965;
923809	-	TSS_026554	1000	236	0	4	IP	within gene(s) PMM0966; 85nt upstream of gene PMM0965;
924547	-	TSS_026558	1000	789	0	0	I	within gene(s) PMM0966;
925793	+	TSS_009277	1000	129	0	2	IP	within gene(s) PMM0968; 65nt upstream of gene PMM0969;
926573	-	TSS_026561	1000	128	0	0	Ai	antisense to gene(s) PMM0970;
926578	+	TSS_009285	1000	150	0	0	I	within gene(s) PMM0970;
926611	+	TSS_009287	1000	141	0	0	I	within gene(s) PMM0970;
926625	+	TSS_009288	1000	228	0	0	I	within gene(s) PMM0970;
926661	+	TSS_009294	1000	403	0	25	I	within gene(s) PMM0970;
926710	-	TSS_026563	1000	171	0	0	Ai	antisense to gene(s) PMM0970;
926754	+	TSS_009304	1000	173	0	3	I	within gene(s) PMM0970;
926772	+	TSS_009307	1000	101	0	3	I	within gene(s) PMM0970;
926805	+	TSS_009314	1000	372	0	18	I	within gene(s) PMM0970;
926853	+	TSS_009318	1000	166	0	12	I	within gene(s) PMM0970;

926889	+	TSS_009325	1000	210	0	14	I	within gene(s) PMM0970;
926952	+	TSS_009343	1000	562	0	51	I	within gene(s) PMM0970;
926970	+	TSS_009345	1000	123	0	0	I	within gene(s) PMM0970;
926982	+	TSS_009347	1000	175	0	11	I	within gene(s) PMM0970;
927015	+	TSS_009355	1000	188	0	54	I	within gene(s) PMM0970;
927063	+	TSS_009369	1000	107	0	0	I	within gene(s) PMM0970;
927072	+	TSS_009370	1000	153	0	18	I	within gene(s) PMM0970;
927117	+	TSS_009380	1000	295	0	15	I	within gene(s) PMM0970;
927189	+	TSS_009386	1000	160	0	19	I	within gene(s) PMM0970;
927237	+	TSS_009399	1000	247	0	21	I	within gene(s) PMM0970;
927259	+	TSS_009402	1000	277	0	0	I	within gene(s) PMM0970;
927270	+	TSS_009404	1000	197	0	3	I	within gene(s) PMM0970;
927291	+	TSS_009408	1000	284	0	15	I	within gene(s) PMM0970;
927318	+	TSS_009411	1000	146	0	15	I	within gene(s) PMM0970;
927357	+	TSS_009420	1000	326	0	36	I	within gene(s) PMM0970;
927396	+	TSS_009432	1000	216	0	6	I	within gene(s) PMM0970;
927406	+	TSS_009434	1000	350	0	12	I	within gene(s) PMM0970;
927441	+	TSS_009440	1000	1310	0	16	I	within gene(s) PMM0970;
927474	+	TSS_009444	1000	147	0	3	I	within gene(s) PMM0970;
927489	+	TSS_009446	1000	145	0	6	I	within gene(s) PMM0970;
927522	+	TSS_009450	1000	128	0	12	I	within gene(s) PMM0970;
927568	-	TSS_026569	1000	1014	0	0	Ai	antisense to gene(s) PMM0970;
927597	-	TSS_026570	1000	106	0	0	Ai	antisense to gene(s) PMM0970;
927626	-	TSS_026572	1000	192	0	9	Ai	antisense to gene(s) PMM0970;
927755	-	TSS_026576	1000	142	0	0	Ai	antisense to gene(s) PMM0970;
929465	-	TSS_026584	1000	140	0	1	Ai	antisense to gene(s) PMM0972;
930151	+	TSS_009479	1000	176	0	7	IP	within gene(s) PMM0972; 101nt upstream of gene PMM0973;
930208	-	TSS_026593	1000	504	0	4	Ai	antisense to gene(s) PMM0972;
931059	+	TSS_009485	1000	137	0	0	I	within gene(s) PMM0974;
932988	+	TSS_009491	1000	216	0	3	P	2nt upstream of gene PMM0975;
933017	+	TSS_009493	1000	670	0	0	I	within gene(s) PMM0975;
936929	+	TSS_009500	1000	748	0	1	O	-
937963	+	TSS_009502	1000	1491	0	1	P	15nt upstream of gene PMM0982;
939028	-	TSS_026613	1000	370	0	0	P	17nt upstream of gene PMM0983;
939290	-	TSS_026614	1000	118	0	0	O	-
941587	+	TSS_009514	1000	540	0	6	P	17nt upstream of gene PMM0987;
942377	-	TSS_026628	1000	667	0	1	P	126nt upstream of gene PMM0988;
945784	+	TSS_009523	1000	635	0	3	P	20nt upstream of gene PMM0992;
946441	+	TSS_009525	1000	289	0	0	Ai	antisense to gene(s) PMM0993;
946927	-	TSS_026640	1000	1114	0	4	P	16nt upstream of gene PMM0993;
947802	+	TSS_009529	1000	123	0	0	O	-
949329	-	TSS_026659	1000	15525	0	2	O	-
950002	+	TSS_009541	1000	690	0	1	P	15nt upstream of gene PMM0999;
950681	+	TSS_009542	1000	235	0	0	P	15nt upstream of gene PMM1001;
956897	-	TSS_026674	1000	2506	0	3	P	17nt upstream of gene PMM1005;
957665	+	TSS_009553	1000	104	0	0	Ai	antisense to gene(s) PMM1007;
957700	+	TSS_009556	1000	118	0	16	Ai	antisense to gene(s) PMM1007;
957751	+	TSS_009563	1000	118	0	16	Ai	antisense to gene(s) PMM1007;
957764	-	TSS_026692	1000	342	0	150	I	within gene(s) PMM1007;
957802	+	TSS_009570	1000	118	0	13	Ai	antisense to gene(s) PMM1007;
957885	-	TSS_026731	1000	280	0	0	I	within gene(s) PMM1007;
957904	+	TSS_009576	1000	118	0	16	Ai	antisense to gene(s) PMM1007;
957955	+	TSS_009583	1000	118	0	16	Ai	antisense to gene(s) PMM1007;
957968	-	TSS_026741	1000	342	0	144	I	within gene(s) PMM1007;
958006	+	TSS_009590	1000	118	0	16	Ai	antisense to gene(s) PMM1007;
958089	-	TSS_026781	1000	280	0	0	I	within gene(s) PMM1007;
958146	-	TSS_026786	1000	105	0	6	I	within gene(s) PMM1007;
958314	-	TSS_026797	1000	270	0	0	P	15nt upstream of gene PMM1007;
958766	-	TSS_026798	1000	195	0	0	P	26nt upstream of gene PMM1008;
958974	+	TSS_009599	1000	113	0	0	I	within gene(s) PMM1009;
960692	-	TSS_026801	1000	254	0	4	P	16nt upstream of gene PMM1011;
961818	+	TSS_009606	1000	517	0	3	P	14nt upstream of gene PMM1013;
963397	-	TSS_026808	1000	117	0	0	P	14nt upstream of gene PMM1015;
965105	+	TSS_009617	1000	454	0	3	O	-
965725	-	TSS_026812	1000	146	0	1	P	238nt upstream of gene PMM1018a;
968005	+	TSS_009627	1000	157	0	5	I	within gene(s) PMM1022;
971676	-	TSS_026824	1000	250	0	0	P	4nt upstream of gene PMM1026;
972175	-	TSS_026825	1000	1729	0	2	O	-
972740	+	TSS_009639	1000	381	0	0	P	166nt upstream of gene PMM1028;
973076	-	TSS_026829	1000	291	0	0	Ai	antisense to gene(s) PMM1028;
973232	+	TSS_009650	1000	1629	0	6	O	-
974212	+	TSS_009654	1000	607	0	2	P	36nt upstream of gene PMM1030;
974221	+	TSS_009655	1000	1830	0	1	P	27nt upstream of gene PMM1030;
975550	+	TSS_009665	1000	465	0	1	P	17nt upstream of gene PMM1032;
977043	+	TSS_009718	1000	1554	0	6	P	32nt upstream of gene PMM1033;
977685	-	TSS_026851	1000	123	0	0	Ai	antisense to gene(s) PMM1033;
978424	+	TSS_009778	1000	190	0	0	I	within gene(s) PMM1033 PMM1034;

978771	+	TSS_009782	1000	101	0	0	I	within gene(s) PMM1034;
979706	-	TSS_026868	1000	277	0	2	O	-
979901	+	TSS_009785	1000	494	0	2	O	-
981473	+	TSS_009792	1000	130	0	1	P	1nt upstream of gene PMM1038;
981600	+	TSS_009793	1000	176	0	0	I	within gene(s) PMM1038;
984870	+	TSS_009812	1000	607	0	0	I	within gene(s) PMM1041;
985398	+	TSS_009813	1000	527	0	0	IP	within gene(s) PMM1041; 91nt upstream of gene PMM1042;
985474	+	TSS_009819	1000	5481	0	8	P	15nt upstream of gene PMM1042;
985522	+	TSS_009822	1000	565	0	0	I	within gene(s) PMM1042;
985651	+	TSS_009823	1000	190	0	0	I	within gene(s) PMM1042;
995402	+	TSS_009844	1000	203	0	0	Ad	antisense to gene(s) PMM1053 (3nt downstream);
996144	-	TSS_026906	1000	356	0	0	IP	within gene(s) PMM1054; 183nt upstream of gene PMM1053;
996203	-	TSS_026909	1000	118	0	0	IP	within gene(s) PMM1054; 242nt upstream of gene PMM1053;
996589	-	TSS_026918	1000	116	0	3	IP	within gene(s) PMM1055; 145nt upstream of gene PMM1054;
997065	-	TSS_026925	1000	128	0	1	I	within gene(s) PMM1055;
997098	+	TSS_009848	1000	111	0	0	Ai	antisense to gene(s) PMM1055;
997566	-	TSS_026935	1000	187	0	0	P	29nt upstream of gene PMM1055;
998948	-	TSS_026937	1000	113	0	0	I	within gene(s) PMM1057;
998966	+	TSS_009855	1000	2796	0	3	P	23nt upstream of gene PMM1058;
1000663	-	TSS_026949	1000	1056	0	3	P	16nt upstream of gene PMM1061;
1000796	-	TSS_026953	1000	15905	0	3	P	149nt upstream of gene PMM1061;
1000995	-	TSS_026955	1000	216	0	1	I	within gene(s) PMM1062;
1001106	+	TSS_009864	1000	666	0	1	Ai	antisense to gene(s) PMM1062;
1001916	-	TSS_026961	1000	153	0	0	I	within gene(s) PMM1062;
1002034	-	TSS_026964	1000	124	0	2	P	15nt upstream of gene PMM1062;
1004165	+	TSS_009892	1000	116	0	0	I	within gene(s) PMM1063;
1004709	+	TSS_009899	1000	170	0	9	IP	within gene(s) PMM1063; 118nt upstream of gene PMM1064;
1008308	+	TSS_009920	1000	142	0	19	I	within gene(s) PMM1066;
1008758	+	TSS_009940	1000	125	0	3	I	within gene(s) PMM1066;
1008779	+	TSS_009943	1000	183	0	0	I	within gene(s) PMM1066;
1008963	+	TSS_009953	1000	781	0	0	I	within gene(s) PMM1066;
1009058	+	TSS_009954	1000	183	0	3	I	within gene(s) PMM1066;
1009166	+	TSS_009965	1000	140	0	18	I	within gene(s) PMM1066;
1009566	+	TSS_009986	1000	232	0	0	P	29nt upstream of gene PMM1067;
1009601	+	TSS_009988	1000	149	0	3	I	within gene(s) PMM1067;
1012301	+	TSS_010005	1000	341	0	5	IP	within gene(s) PMM1069; 179nt upstream of gene PMM1070;
1012326	+	TSS_010008	1000	159	0	4	IP	within gene(s) PMM1069; 154nt upstream of gene PMM1070;
1013805	-	TSS_026998	1000	323	0	0	Ai	antisense to gene(s) PMM1071;
1013842	-	TSS_026999	1000	190	0	0	Ai	antisense to gene(s) PMM1071;
1016820	-	TSS_027011	1000	111	0	3	IP	within gene(s) PMM1074; 241nt upstream of gene PMM1073;
1018128	-	TSS_027052	1000	1323	0	2	P	18nt upstream of gene PMM1074;
1018304	+	TSS_010041	1000	286	0	0	Ai	antisense to gene(s) PMM1075;
1018320	+	TSS_010044	1000	157	0	7	Ai	antisense to gene(s) PMM1075;
1018329	+	TSS_010046	1000	114	0	0	Ai	antisense to gene(s) PMM1075;
1018379	-	TSS_027057	1000	159	0	6	I	within gene(s) PMM1075;
1018420	-	TSS_027068	1000	288	0	42	I	within gene(s) PMM1075;
1018463	-	TSS_027079	1000	220	0	42	I	within gene(s) PMM1075;
1018469	+	TSS_010051	1000	275	0	0	Ai	antisense to gene(s) PMM1075;
1018476	+	TSS_010052	1000	139	0	0	Ai	antisense to gene(s) PMM1075;
1018499	-	TSS_027088	1000	126	0	3	I	within gene(s) PMM1075;
1018511	-	TSS_027089	1000	111	0	9	I	within gene(s) PMM1075;
1018532	-	TSS_027092	1000	139	0	0	I	within gene(s) PMM1075;
1018550	-	TSS_027093	1000	186	0	15	I	within gene(s) PMM1075;
1018586	-	TSS_027100	1000	206	0	6	I	within gene(s) PMM1075;
1018595	-	TSS_027101	1000	230	0	0	I	within gene(s) PMM1075;
1018598	+	TSS_010056	1000	462	0	3	Ai	antisense to gene(s) PMM1075;
1018613	-	TSS_027103	1000	220	0	16	I	within gene(s) PMM1075;
1018629	+	TSS_010058	1000	140	0	4	Ai	antisense to gene(s) PMM1075;
1018655	-	TSS_027111	1000	1755	0	33	I	within gene(s) PMM1075;
1018735	-	TSS_027130	1000	343	0	48	I	within gene(s) PMM1075;
1018757	-	TSS_027135	1000	1163	0	15	I	within gene(s) PMM1075;
1018758	+	TSS_010061	1000	196	0	1	Ai	antisense to gene(s) PMM1075;
1018856	-	TSS_027160	1000	410	0	87	I	within gene(s) PMM1075;
1018877	-	TSS_027164	1000	230	0	21	I	within gene(s) PMM1075;
1018949	-	TSS_027183	1000	405	0	66	I	within gene(s) PMM1075;
1018979	-	TSS_027190	1000	294	0	24	I	within gene(s) PMM1075;
1019075	-	TSS_027203	1000	163	0	15	I	within gene(s) PMM1075;
1019084	-	TSS_027204	1000	116	0	0	I	within gene(s) PMM1075;
1019120	-	TSS_027205	1000	159	0	0	I	within gene(s) PMM1075;
1019153	-	TSS_027211	1000	513	0	35	I	within gene(s) PMM1075;
1019180	-	TSS_027216	1000	177	0	0	I	within gene(s) PMM1075;
1019195	-	TSS_027218	1000	222	0	6	I	within gene(s) PMM1075;
1019207	-	TSS_027220	1000	195	0	3	I	within gene(s) PMM1075;
1019222	-	TSS_027224	1000	2761	0	12	I	within gene(s) PMM1075;
1019249	-	TSS_027227	1000	191	0	6	I	within gene(s) PMM1075;
1019366	-	TSS_027229	1000	2221	0	1	P	30nt upstream of gene PMM1075;
1020266	+	TSS_010067	1000	236	0	0	Ai	antisense to gene(s) PMM1077;

1020379	-	TSS_027238	1000	3903	0	2	I	within gene(s) PMM1077;
1020982	-	TSS_027246	1000	206	0	1	I	within gene(s) PMM1077;
1021282	-	TSS_027248	1000	473	0	0	I	within gene(s) PMM1077;
1022015	+	TSS_010071	1000	256	0	0	P	18nt upstream of gene PMM1079;
1022762	-	TSS_027254	1000	291	0	2	I	within gene(s) PMM1080;
1022879	+	TSS_010078	1000	118	0	0	Ai	antisense to gene(s) PMM1080;
1022925	-	TSS_027259	1000	148	0	18	I	within gene(s) PMM1080;
1022955	-	TSS_027266	1000	166	0	66	I	within gene(s) PMM1080;
1023018	-	TSS_027280	1000	317	0	10	I	within gene(s) PMM1080;
1023140	+	TSS_010083	1000	231	0	6	Ai	antisense to gene(s) PMM1080;
1023357	-	TSS_027299	1000	189	0	4	I	within gene(s) PMM1080;
1023402	-	TSS_027300	1000	224	0	0	I	within gene(s) PMM1080;
1023447	-	TSS_027302	1000	196	0	3	I	within gene(s) PMM1080;
1023493	-	TSS_027306	1000	4086	0	1	P	19nt upstream of gene PMM1080;
1023698	+	TSS_010090	1000	143	0	3	P	26nt upstream of gene PMM1081;
1024480	+	TSS_010100	1000	340	0	10	I	within gene(s) PMM1081;
1024757	-	TSS_027310	1000	202	0	0	Ai	antisense to gene(s) PMM1081;
1028961	+	TSS_010112	1000	179	0	0	IP	within gene(s) PMM1084; 27nt upstream of gene PMM1085;
1029223	-	TSS_027325	1000	285	0	0	Ai	antisense to gene(s) PMM1085;
1029801	-	TSS_027327	1000	432	0	0	Ai	antisense to gene(s) PMM1085;
1032041	-	TSS_027344	1000	105	0	7	IP	within gene(s) PMM1088; 32nt upstream of gene PMM1087;
1032059	-	TSS_027347	1000	114	0	18	IP	within gene(s) PMM1088; 50nt upstream of gene PMM1087;
1032141	+	TSS_010119	1000	392	0	0	Ai	antisense to gene(s) PMM1088;
1032155	+	TSS_010120	1000	746	0	1	Ai	antisense to gene(s) PMM1088;
1032215	-	TSS_027363	1000	271	0	30	IP	within gene(s) PMM1088; 206nt upstream of gene PMM1087;
1032230	-	TSS_027366	1000	173	0	12	IP	within gene(s) PMM1088; 221nt upstream of gene PMM1087;
1032254	-	TSS_027371	1000	506	0	87	IP	within gene(s) PMM1088; 245nt upstream of gene PMM1087;
1032374	-	TSS_027404	1000	210	0	30	I	within gene(s) PMM1088;
1032392	-	TSS_027408	1000	180	0	5	I	within gene(s) PMM1088;
1032423	+	TSS_010122	1000	298	0	0	Ai	antisense to gene(s) PMM1088;
1032451	+	TSS_010123	1000	120	0	0	Ai	antisense to gene(s) PMM1088;
1032479	+	TSS_010125	1000	103	0	4	Ai	antisense to gene(s) PMM1088;
1032506	-	TSS_027412	1000	148	0	6	I	within gene(s) PMM1088;
1032599	-	TSS_027423	1000	142	0	27	I	within gene(s) PMM1088;
1032608	+	TSS_010129	1000	102	0	0	Ai	antisense to gene(s) PMM1088;
1032625	+	TSS_010130	1000	144	0	0	Ai	antisense to gene(s) PMM1088;
1032644	-	TSS_027431	1000	162	0	36	I	within gene(s) PMM1088;
1032676	-	TSS_027439	1000	231	0	5	I	within gene(s) PMM1088;
1032689	-	TSS_027442	1000	271	0	6	I	within gene(s) PMM1088;
1032698	-	TSS_027443	1000	229	0	30	I	within gene(s) PMM1088;
1032714	+	TSS_010132	1000	221	0	0	Ai	antisense to gene(s) PMM1088;
1032744	-	TSS_027461	1000	368	0	9	I	within gene(s) PMM1088;
1032755	-	TSS_027463	1000	180	0	11	I	within gene(s) PMM1088;
1032779	-	TSS_027469	1000	224	0	9	I	within gene(s) PMM1088;
1032803	-	TSS_027475	1000	278	0	18	I	within gene(s) PMM1088;
1032827	-	TSS_027480	1000	693	0	3	I	within gene(s) PMM1088;
1032845	-	TSS_027482	1000	214	0	0	I	within gene(s) PMM1088;
1032863	-	TSS_027484	1000	321	0	27	I	within gene(s) PMM1088;
1032911	-	TSS_027498	1000	175	0	36	I	within gene(s) PMM1088;
1032944	-	TSS_027504	1000	109	0	0	I	within gene(s) PMM1088;
1032953	-	TSS_027505	1000	407	0	12	I	within gene(s) PMM1088;
1032989	-	TSS_027509	1000	145	0	0	I	within gene(s) PMM1088;
1032998	-	TSS_027510	1000	146	0	0	I	within gene(s) PMM1088;
1033025	-	TSS_027514	1000	122	0	15	I	within gene(s) PMM1088;
1033052	-	TSS_027517	1000	178	0	18	I	within gene(s) PMM1088;
1033079	-	TSS_027521	1000	223	0	12	I	within gene(s) PMM1088;
1033109	-	TSS_027529	1000	343	0	48	I	within gene(s) PMM1088;
1033166	-	TSS_027547	1000	178	0	33	I	within gene(s) PMM1088;
1033286	-	TSS_027560	1000	287	0	0	I	within gene(s) PMM1088;
1033390	-	TSS_027583	1000	896	0	129	I	within gene(s) PMM1088;
1033463	-	TSS_027606	1000	144	0	0	I	within gene(s) PMM1088;
1033472	-	TSS_027607	1000	582	0	12	I	within gene(s) PMM1088;
1033493	-	TSS_027613	1000	626	0	0	I	within gene(s) PMM1088;
1033505	-	TSS_027614	1000	120	0	0	I	within gene(s) PMM1088;
1033550	-	TSS_027620	1000	327	0	12	I	within gene(s) PMM1088;
1033577	-	TSS_027621	1000	163	0	0	I	within gene(s) PMM1088;
1033637	-	TSS_027628	1000	291	0	15	I	within gene(s) PMM1088;
1033667	-	TSS_027632	1000	373	0	30	I	within gene(s) PMM1088;
1033714	-	TSS_027642	1000	125	0	2	I	within gene(s) PMM1088;
1033736	-	TSS_027643	1000	135	0	6	I	within gene(s) PMM1088;
1033757	-	TSS_027646	1000	162	0	0	I	within gene(s) PMM1088;
1033781	-	TSS_027649	1000	171	0	14	I	within gene(s) PMM1088;
1033847	-	TSS_027665	1000	347	0	45	I	within gene(s) PMM1088;
1033868	-	TSS_027668	1000	703	0	12	I	within gene(s) PMM1088;
1033898	-	TSS_027677	1000	242	0	18	I	within gene(s) PMM1088;
1033940	-	TSS_027686	1000	871	0	42	I	within gene(s) PMM1088;
1034009	-	TSS_027698	1000	282	0	3	I	within gene(s) PMM1088;

1034030	-	TSS_027703	1000	206	0	15	I	within gene(s) PMM1088;
1034052	+	TSS_010145	1000	114	0	0	Ai	antisense to gene(s) PMM1088;
1034060	-	TSS_027708	1000	101	0	0	I	within gene(s) PMM1088;
1034069	-	TSS_027709	1000	129	0	0	I	within gene(s) PMM1088;
1034123	-	TSS_027713	1000	292	0	21	I	within gene(s) PMM1088;
1034144	-	TSS_027717	1000	151	0	3	I	within gene(s) PMM1088;
1034168	-	TSS_027720	1000	222	0	15	I	within gene(s) PMM1088;
1034192	-	TSS_027726	1000	122	0	4	I	within gene(s) PMM1088;
1034213	-	TSS_027730	1000	174	0	9	I	within gene(s) PMM1088;
1034221	+	TSS_010153	1000	173	0	5	Ai	antisense to gene(s) PMM1088;
1034222	-	TSS_027731	1000	148	0	0	I	within gene(s) PMM1088;
1034242	+	TSS_010155	1000	159	0	0	Ai	antisense to gene(s) PMM1088;
1034242	-	TSS_027733	1000	761	0	1	I	within gene(s) PMM1088;
1034282	-	TSS_027743	1000	301	0	25	I	within gene(s) PMM1088;
1034309	-	TSS_027749	1000	309	0	12	I	within gene(s) PMM1088;
1034343	+	TSS_010159	1000	119	0	0	Ai	antisense to gene(s) PMM1088;
1034369	-	TSS_027764	1000	552	0	57	I	within gene(s) PMM1088;
1034380	+	TSS_010162	1000	343	0	3	Ai	antisense to gene(s) PMM1088;
1034390	-	TSS_027769	1000	862	0	48	I	within gene(s) PMM1088;
1034447	-	TSS_027788	1000	186	0	2	I	within gene(s) PMM1088;
1034467	-	TSS_027790	1000	158	9.40E-06	0	I	within gene(s) PMM1088;
1034492	-	TSS_027791	1000	184	0	0	I	within gene(s) PMM1088;
1034510	-	TSS_027794	1000	323	0	3	I	within gene(s) PMM1088;
1035252	+	TSS_010165	1000	767	0	0	P	14nt upstream of gene PMM1090;
1036076	+	TSS_010176	1000	929	0	13	I	within gene(s) PMM1090;
1037053	+	TSS_010192	1000	115	0	1	I	within gene(s) PMM1091;
1037521	+	TSS_010195	1000	215	0	0	IP	within gene(s) PMM1091; 41nt upstream of gene PMM1092;
1037544	-	TSS_027804	1000	116	0	0	Ai	antisense to gene(s) PMM1091;
1037579	-	TSS_027805	1000	481	0	0	Ai	antisense to gene(s) PMM1092;
1038879	-	TSS_027810	1000	163	0	0	Ai	antisense to gene(s) PMM1093;
1039004	+	TSS_010219	1000	207	0	2	I	within gene(s) PMM1093;
1040425	+	TSS_010227	1000	103	0	0	I	within gene(s) PMM1095;
1041190	+	TSS_010228	1000	113	0	0	Ai	antisense to gene(s) PMM1096;
1041219	+	TSS_010229	1000	138	0	0	Ai	antisense to gene(s) PMM1096;
1041398	-	TSS_027818	1000	135	0	0	I	within gene(s) PMM1096;
1041770	+	TSS_010230	1000	1335	0	1	Ai	antisense to gene(s) PMM1097;
1042334	-	TSS_027825	1000	642	0	0	P	14nt upstream of gene PMM1097;
1042406	+	TSS_010237	1000	3608	0	1	P	20nt upstream of gene PMM1098;
1042513	+	TSS_010250	1000	108	0	30	I	within gene(s) PMM1098;
1042908	-	TSS_027831	1000	170	0	1	Ai	antisense to gene(s) PMM1098;
1043114	+	TSS_010277	1000	106	0	0	Ai	antisense to gene(s) PMM1099;
1046932	-	TSS_027837	1000	156	0	0	P	81nt upstream of gene PMM1101;
1057074	-	TSS_027853	1000	4014	0	2	P	35nt upstream of gene PMM1107;
1057110	-	TSS_027855	1000	293	0	3	P	71nt upstream of gene PMM1107;
1058110	+	TSS_010310	1000	135	0	1	I	within gene(s) PMM1109;
1058522	+	TSS_010319	1000	958	0	3	IP	within gene(s) PMM1110; 95nt upstream of gene PMM1111;
1059270	+	TSS_010326	1000	122	0	1	P	76nt upstream of gene PMM1113;
1059299	+	TSS_010328	1000	738	0	2	P	47nt upstream of gene PMM1113;
1059640	+	TSS_010332	1000	107	0	0	I	within gene(s) PMM1113;
1059858	+	TSS_010339	1000	570	0	2	I	within gene(s) PMM1113;
1060619	+	TSS_010347	1000	169	0	2	Ai	antisense to gene(s) PMM1115;
1063397	+	TSS_010356	1000	127	0	6	Ai	antisense to gene(s) PMM1116;
1063626	-	TSS_027888	1000	115	0	6	IP	within gene(s) PMM1117; 57nt upstream of gene PMM1116;
1063653	-	TSS_027890	1000	239	0	6	IP	within gene(s) PMM1117; 84nt upstream of gene PMM1116;
1063680	-	TSS_027894	1000	170	0	5	IP	within gene(s) PMM1117; 111nt upstream of gene PMM1116;
1063850	+	TSS_010360	1000	358	0	4	O	-
1064477	+	TSS_010361	1000	124	0	0	P	24nt upstream of gene PMM1118;
1065289	+	TSS_010368	1000	420	0	0	Ai	antisense to gene(s) PMM1119;
1065300	-	TSS_027902	1000	625	0	6	I	within gene(s) PMM1119;
1065304	+	TSS_010369	1000	1241	0	0	Ai	antisense to gene(s) PMM1119;
1065312	+	TSS_010370	1000	351	0	0	Ai	antisense to gene(s) PMM1119;
1065315	-	TSS_027905	1000	310	0	0	I	within gene(s) PMM1119;
1065384	-	TSS_027925	1000	1924	0	67	I	within gene(s) PMM1119;
1065409	+	TSS_010373	1000	269	0	4	Ai	antisense to gene(s) PMM1119;
1065446	+	TSS_010376	1000	187	0	0	Ai	antisense to gene(s) PMM1119;
1065525	-	TSS_027965	1000	2976	0	125	I	within gene(s) PMM1119;
1065751	+	TSS_010379	1000	175	0	0	Ai	antisense to gene(s) PMM1119;
1065783	-	TSS_028082	1000	4468	0	219	I	within gene(s) PMM1119;
1065834	-	TSS_028109	1000	2784	0	242	I	within gene(s) PMM1119;
1066098	-	TSS_028253	1000	663	0	51	I	within gene(s) PMM1119;
1068483	-	TSS_028264	1000	245	0	27	I	within gene(s) PMM1121;
1068498	-	TSS_028268	1000	304	0	7	I	within gene(s) PMM1121;
1068505	+	TSS_010395	1000	420	0	0	Ai	antisense to gene(s) PMM1121;
1068519	-	TSS_028274	1000	1067	0	30	I	within gene(s) PMM1121;
1068520	+	TSS_010396	1000	1241	0	0	Ai	antisense to gene(s) PMM1121;
1068528	+	TSS_010397	1000	351	0	1	Ai	antisense to gene(s) PMM1121;
1068570	-	TSS_028297	1000	1174	0	39	I	within gene(s) PMM1121;

1068600	-	TSS_028307	1000	1897	0	30	I	within gene(s) PMM1121;
1068616	+	TSS_010402	1000	124	0	0	Ai	antisense to gene(s) PMM1121;
1068625	+	TSS_010403	1000	269	0	4	Ai	antisense to gene(s) PMM1121;
1068662	+	TSS_010406	1000	187	0	0	Ai	antisense to gene(s) PMM1121;
1068666	-	TSS_028336	1000	3106	0	138	I	within gene(s) PMM1121;
1068967	+	TSS_010409	1000	175	0	0	Ai	antisense to gene(s) PMM1121;
1068999	-	TSS_028491	1000	4468	0	390	I	within gene(s) PMM1121;
1069166	+	TSS_010414	1000	143	0	1	Ai	antisense to gene(s) PMM1121;
1069204	+	TSS_010416	1000	155	0	0	Ai	antisense to gene(s) PMM1121;
1069243	+	TSS_010418	1000	1276	0	1	Ai	antisense to gene(s) PMM1121;
1069261	+	TSS_010419	1000	295	0	1	Ai	antisense to gene(s) PMM1121;
1069311	-	TSS_028692	1000	1747	0	231	I	within gene(s) PMM1121;
1069339	+	TSS_010421	1000	341	0	0	Ai	antisense to gene(s) PMM1121;
1069432	-	TSS_028769	1000	1183808	0	7	P	13nt upstream of gene PMM1121;
1069447	-	TSS_028771	1000	2699	0	2	P	28nt upstream of gene PMM1121;
1070354	-	TSS_028774	1000	149	0	1	O	-
1070531	+	TSS_010422	1000	130	0	1	O	-
1071588	+	TSS_010427	1000	110	0	0	O	-
1071727	-	TSS_028794	1000	185	0	42	I	within gene(s) PMM1123;
1071728	+	TSS_010428	1000	688	0	0	Ai	antisense to gene(s) PMM1123;
1071757	-	TSS_028801	1000	230	0	24	I	within gene(s) PMM1123;
1071771	+	TSS_010429	1000	114	0	3	Ai	antisense to gene(s) PMM1123;
1071780	-	TSS_028805	1000	3048	0	0	I	within gene(s) PMM1123;
1071805	-	TSS_028811	1000	634	0	30	I	within gene(s) PMM1123;
1071829	-	TSS_028821	1000	164	0	0	I	within gene(s) PMM1123;
1071844	-	TSS_028823	1000	264	0	18	I	within gene(s) PMM1123;
1071889	-	TSS_028831	1000	392	0	24	I	within gene(s) PMM1123;
1071928	-	TSS_028838	1000	1181	0	34	I	within gene(s) PMM1123;
1071982	-	TSS_028853	1000	197	0	12	I	within gene(s) PMM1123;
1071997	-	TSS_028854	1000	112	1.80E-09	0	I	within gene(s) PMM1123;
1072015	-	TSS_028855	1000	150	0	6	I	within gene(s) PMM1123;
1072072	-	TSS_028858	1000	208	0	6	I	within gene(s) PMM1123;
1072087	-	TSS_028860	1000	364	0	0	I	within gene(s) PMM1123;
1072108	-	TSS_028861	1000	142	0	0	I	within gene(s) PMM1123;
1072132	-	TSS_028868	1000	1469	0	51	I	within gene(s) PMM1123;
1072179	-	TSS_028884	1000	161	0	2	I	within gene(s) PMM1123;
1072201	-	TSS_028888	1000	1487	0	18	I	within gene(s) PMM1123;
1072228	-	TSS_028894	1000	105	0	0	I	within gene(s) PMM1123;
1072255	-	TSS_028896	1000	115	0	1	I	within gene(s) PMM1123;
1072282	-	TSS_028898	1000	131	0	3	I	within gene(s) PMM1123;
1072334	-	TSS_028899	1000	134	0	0	I	within gene(s) PMM1123;
1072352	-	TSS_028902	1000	323	0	9	I	within gene(s) PMM1123;
1072364	+	TSS_010431	1000	247	0	5	Ai	antisense to gene(s) PMM1123;
1072367	-	TSS_028906	1000	177	0	6	I	within gene(s) PMM1123;
1072388	-	TSS_028910	1000	299	0	27	P	13nt upstream of gene PMM1123;
1072451	-	TSS_028916	1000	119	0	0	P	76nt upstream of gene PMM1123;
1074287	-	TSS_028925	1000	904	0	1	I	within gene(s) PMM1124;
1076222	+	TSS_010443	1000	140	0	0	Ai	antisense to gene(s) PMM1126;
1078286	-	TSS_028936	1000	1163	0	1	I	within gene(s) PMM1127;
1078419	+	TSS_010448	1000	112	0	0	Ai	antisense to gene(s) PMM1127;
1078972	-	TSS_028939	1000	163	0	1	O	-
1079173	+	TSS_010452	1000	111	0	0	Ad	antisense to gene(s) PMM1128 (19nt downstream);
1079462	-	TSS_028943	1000	2660	0	3	P	17nt upstream of gene PMM1128;
1080845	+	TSS_010455	1000	261	0	1	P	24nt upstream of gene PMM1131;
1082063	-	TSS_028951	1000	3392	0	3	O	-
1082218	+	TSS_010464	1000	3230	0	4	P	51nt upstream of gene PMM1132;
1083365	-	TSS_028959	1000	227	0	1	Ai	antisense to gene(s) PMM1132;
1083716	+	TSS_010477	1000	133	0	0	I	within gene(s) PMM1132;
1084462	-	TSS_028966	1000	114	0	0	P	43nt upstream of gene PMM1133;
1084946	-	TSS_028967	1000	200	0	4	O	-
1085043	+	TSS_010482	1000	4235	0	3	P	19nt upstream of gene PMM1134;
1085641	+	TSS_010490	1000	5417	0	4	P	15nt upstream of gene PMM1135;
1085683	+	TSS_010496	1000	113	0	6	I	within gene(s) PMM1135;
1085773	+	TSS_010504	1000	120	0	12	I	within gene(s) PMM1135;
1086451	-	TSS_028974	1000	129	0	0	O	-
1087615	+	TSS_010509	1000	763	0	0	O	-
1089084	-	TSS_028983	1000	163	0	6	P	38nt upstream of gene PMM1138;
1089687	+	TSS_010513	1000	136	0	0	I	within gene(s) PMM1139;
1090775	+	TSS_010515	1000	497	0	0	I	within gene(s) PMM1140;
1094754	+	TSS_010525	1000	723	0	1	P	29nt upstream of gene PMM1142;
1095730	+	TSS_010538	1000	228	0	0	I	within gene(s) PMM1142;
1098755	-	TSS_029037	1000	134	0	2	I	within gene(s) PMM1145;
1099231	-	TSS_029042	1000	103	0	0	IP	within gene(s) PMM1147; 54nt upstream of gene PMM1146;
1099273	-	TSS_029043	1000	445	0	0	IP	within gene(s) PMM1147; 96nt upstream of gene PMM1146;
1100340	-	TSS_029077	1000	1956	0	7	P	17nt upstream of gene PMM1147;
1100440	+	TSS_010556	1000	687	0	1	P	16nt upstream of gene PMM1148;
1100504	+	TSS_010558	1000	155	0	0	I	within gene(s) PMM1148;

1100537	+	TSS_010559	1000	138	0	0	I	within gene(s) PMM1148;
1100564	+	TSS_010560	1000	135	0	0	I	within gene(s) PMM1148;
1100603	+	TSS_010566	1000	262	0	16	I	within gene(s) PMM1148;
1100639	+	TSS_010576	1000	295	0	43	I	within gene(s) PMM1148;
1100672	+	TSS_010585	1000	232	0	0	I	within gene(s) PMM1148;
1100708	+	TSS_010595	1000	283	0	42	I	within gene(s) PMM1148;
1100762	+	TSS_010605	1000	268	0	31	I	within gene(s) PMM1148;
1100792	+	TSS_010610	1000	207	0	12	I	within gene(s) PMM1148;
1100819	+	TSS_010616	1000	122	0	9	I	within gene(s) PMM1148;
1100867	+	TSS_010626	1000	263	0	33	I	within gene(s) PMM1148;
1100882	+	TSS_010627	1000	156	0	6	I	within gene(s) PMM1148;
1100965	-	TSS_029083	1000	181	0	1	Ai	antisense to gene(s) PMM1148;
1101152	+	TSS_010635	1000	172	0	0	P	38nt upstream of gene PMM1149;
1101175	+	TSS_010637	1000	10039	0	2	P	15nt upstream of gene PMM1149;
1101223	+	TSS_010640	1000	185	0	18	I	within gene(s) PMM1149;
1101277	+	TSS_010648	1000	240	0	6	IP	within gene(s) PMM1149; 206nt upstream of gene PMM1150;
1101996	+	TSS_010700	1000	113	0	9	I	within gene(s) PMM1150;
1102380	+	TSS_010733	1000	116	0	1	I	within gene(s) PMM1150;
1103158	-	TSS_029100	1000	3046	0	3	P	16nt upstream of gene PMM1151;
1103474	-	TSS_029106	1000	2727	0	6	O	-
1104451	+	TSS_010747	1000	406	0	0	PAi	217nt upstream of gene PMM1153; antisense to gene(s) PMM1152;
1104492	-	TSS_029119	1000	313	0	0	P	18nt upstream of gene PMM1152;
1104544	-	TSS_029121	1000	379	0	9	IP	within gene(s) PMM1152a; 70nt upstream of gene PMM1152;
1104562	-	TSS_029126	1000	113	0	0	IP	within gene(s) PMM1152a; 88nt upstream of gene PMM1152;
1104586	-	TSS_029130	1000	4576	0	10	IP	within gene(s) PMM1152a; 112nt upstream of gene PMM1152;
1104607	-	TSS_029134	1000	204	0	9	IP	within gene(s) PMM1152a; 133nt upstream of gene PMM1152;
1105595	+	TSS_010753	1000	761	0	5	IP	within gene(s) PMM1153; 99nt upstream of gene PMM1154;
1107191	+	TSS_010762	1000	131	0	0	Ai	antisense to gene(s) PMM1156;
1107430	-	TSS_029143	1000	1553	0	3	P	2nt upstream of gene PMM1156;
1108300	+	TSS_010767	1000	1388	0	27	I	within gene(s) PMM1157;
1108348	+	TSS_010774	1000	1216	0	0	I	within gene(s) PMM1157;
1108361	-	TSS_029151	1000	841	0	4	Ai	antisense to gene(s) PMM1157;
1108375	+	TSS_010775	1000	544	0	0	I	within gene(s) PMM1157;
1108391	+	TSS_010776	1000	789	0	0	I	within gene(s) PMM1157;
1108399	+	TSS_010777	1000	1906	0	0	I	within gene(s) PMM1157;
1108411	+	TSS_010779	1000	1717	0	12	I	within gene(s) PMM1157;
1108441	+	TSS_010786	1000	5058	0	9	IP	within gene(s) PMM1157; 232nt upstream of gene PMM1158;
1108450	+	TSS_010787	1000	3585	0	10	IP	within gene(s) PMM1157; 223nt upstream of gene PMM1158;
1108498	+	TSS_010795	1000	7737	0	12	IP	within gene(s) PMM1157; 175nt upstream of gene PMM1158;
1108754	+	TSS_010799	1000	647	0	11	I	within gene(s) PMM1158;
1108805	+	TSS_010806	1000	4140	0	20	I	within gene(s) PMM1158;
1108856	+	TSS_010811	1000	133	0	6	I	within gene(s) PMM1158;
1108874	+	TSS_010813	1000	133	0	0	I	within gene(s) PMM1158;
1108911	-	TSS_029156	1000	116	0	0	Ai	antisense to gene(s) PMM1158;
1108924	-	TSS_029157	1000	146	0	0	Ai	antisense to gene(s) PMM1158;
1108925	+	TSS_010815	1000	117	0	3	I	within gene(s) PMM1158;
1108940	+	TSS_010817	1000	348	0	3	I	within gene(s) PMM1158;
1108982	+	TSS_010828	1000	997	0	31	I	within gene(s) PMM1158;
1109018	+	TSS_010837	1000	789	0	22	I	within gene(s) PMM1158;
1109030	+	TSS_010839	1000	210	0	0	I	within gene(s) PMM1158;
1109031	-	TSS_029159	1000	141	0	0	Ai	antisense to gene(s) PMM1158;
1109043	-	TSS_029161	1000	179	0	6	Ai	antisense to gene(s) PMM1158;
1109073	+	TSS_010851	1000	402	0	45	I	within gene(s) PMM1158;
1109084	-	TSS_029163	1000	619	0	0	Ai	antisense to gene(s) PMM1158;
1109099	+	TSS_010860	1000	513	0	17	I	within gene(s) PMM1158;
1109117	+	TSS_010863	1000	187	0	1	I	within gene(s) PMM1158;
1109125	+	TSS_010865	1000	146	5.90E-12	0	I	within gene(s) PMM1158;
1109147	+	TSS_010866	1000	318	0	1	I	within gene(s) PMM1158;
1109162	+	TSS_010868	1000	154	0	0	I	within gene(s) PMM1158;
1109165	-	TSS_029165	1000	225	0	1	Ai	antisense to gene(s) PMM1158;
1109196	-	TSS_029167	1000	711	0	3	Ai	antisense to gene(s) PMM1158;
1109204	+	TSS_010879	1000	689	0	42	I	within gene(s) PMM1158;
1109212	-	TSS_029171	1000	245	0	6	Ai	antisense to gene(s) PMM1158;
1109237	+	TSS_010889	1000	717	0	30	I	within gene(s) PMM1158;
1109279	+	TSS_010900	1000	336	0	9	I	within gene(s) PMM1158;
1109351	+	TSS_010904	1000	111	0	0	I	within gene(s) PMM1158;
1109360	+	TSS_010905	1000	139	0	0	I	within gene(s) PMM1158;
1109387	+	TSS_010911	1000	311	0	17	I	within gene(s) PMM1158;
1109399	+	TSS_010914	1000	332	0	16	I	within gene(s) PMM1158;
1109402	-	TSS_029174	1000	914	0	5	Ai	antisense to gene(s) PMM1158;
1109429	+	TSS_010923	1000	137	0	13	I	within gene(s) PMM1158;
1109461	-	TSS_029176	1000	330	0	0	Ai	antisense to gene(s) PMM1158;
1109462	+	TSS_010933	1000	385	0	24	I	within gene(s) PMM1158;
1109477	+	TSS_010937	1000	278	0	0	I	within gene(s) PMM1158;
1109489	+	TSS_010938	1000	111	0	0	I	within gene(s) PMM1158;
1109525	+	TSS_010945	1000	143	0	18	I	within gene(s) PMM1158;
1109540	+	TSS_010946	1000	616	0	35	I	within gene(s) PMM1158;

1109618	+	TSS_010964	1000	165	0	3	I	within gene(s) PMM1158;
1109648	+	TSS_010966	1000	135	0	0	I	within gene(s) PMM1158;
1109653	-	TSS_029182	1000	197	0	0	Ai	antisense to gene(s) PMM1158;
1109694	-	TSS_029183	1000	1107	0	1	Ai	antisense to gene(s) PMM1158;
1109705	-	TSS_029185	1000	131	0	1	Ai	antisense to gene(s) PMM1158;
1109792	-	TSS_029187	1000	401	0	0	Ai	antisense to gene(s) PMM1158;
1109834	+	TSS_010988	1000	294	0	0	I	within gene(s) PMM1158;
1109873	+	TSS_010989	1000	217	0	0	I	within gene(s) PMM1158;
1114472	+	TSS_011002	1000	101	0	6	P	107nt upstream of gene PMM1163;
1115277	+	TSS_011006	1000	190	0	0	O	-
1115437	+	TSS_011008	1000	273	0	0	O	-
1118179	-	TSS_029223	1000	444	0	1	P	33nt upstream of gene PMM1165;
1120049	+	TSS_011022	1000	2340	0	2	P	15nt upstream of gene PMM1169;
1121150	+	TSS_011028	1000	432	0	0	Ai	antisense to gene(s) PMM1171;
1121159	+	TSS_011029	1000	491	0	0	Ai	antisense to gene(s) PMM1171;
1121311	+	TSS_011031	1000	197	0	0	Ai	antisense to gene(s) PMM1171;
1121331	-	TSS_029245	1000	104	0	9	I	within gene(s) PMM1171;
1121343	-	TSS_029247	1000	165	0	15	I	within gene(s) PMM1171;
1121377	+	TSS_011033	1000	237	0	1	Ai	antisense to gene(s) PMM1171;
1121400	-	TSS_029256	1000	500	0	0	I	within gene(s) PMM1171;
1121448	-	TSS_029257	1000	172	0	3	I	within gene(s) PMM1171;
1121466	-	TSS_029261	1000	405	0	16	I	within gene(s) PMM1171;
1121508	-	TSS_029269	1000	369	0	12	I	within gene(s) PMM1171;
1121580	-	TSS_029274	1000	5743	0	3	P	27nt upstream of gene PMM1171;
1122779	+	TSS_011035	1000	513	0	3	P	17nt upstream of gene PMM1174;
1123430	-	TSS_029281	1000	641	0	6	Ai	antisense to gene(s) PMM1175;
1124119	+	TSS_011044	1000	262	0	0	I	within gene(s) PMM1176;
1125577	+	TSS_011048	1000	125	0	0	Ai	antisense to gene(s) PMM1177;
1126503	-	TSS_029289	1000	127	0	2	I	within gene(s) PMM1178;
1127018	+	TSS_011054	1000	533	0	0	Ai	antisense to gene(s) PMM1178;
1127288	-	TSS_029294	1000	125	0	1	P	26nt upstream of gene PMM1178;
1127626	+	TSS_011060	1000	3700	0	2	Ai	antisense to gene(s) PMM1179;
1127928	+	TSS_011064	1000	145	0	0	Ai	antisense to gene(s) PMM1179;
1128259	+	TSS_011066	1000	1084	0	1	P	28nt upstream of gene PMM1180;
1128268	+	TSS_011068	1000	521	0	3	P	19nt upstream of gene PMM1180;
1128474	-	TSS_029306	1000	1688	0	3	PAi	241nt upstream of gene PMM1179; antisense to gene(s) PMM1180;
1128570	-	TSS_029308	1000	862	0	1	Ai	antisense to gene(s) PMM1180;
1130036	+	TSS_011120	1000	116	0	15	IP	within gene(s) PMM1182; 123nt upstream of gene PMM1183;
1130146	+	TSS_011127	1000	11813	0	4	P	13nt upstream of gene PMM1183;
1130398	-	TSS_029319	1000	479	0	0	Ai	antisense to gene(s) PMM1184;
1130619	+	TSS_011129	1000	200	0	1	IP	within gene(s) PMM1184; 74nt upstream of gene PMM1185;
1130649	+	TSS_011132	1000	1611	0	1	IP	within gene(s) PMM1184; 44nt upstream of gene PMM1185;
1131120	+	TSS_011137	1000	332	0	2	P	59nt upstream of gene PMM1186;
1131150	+	TSS_011139	1000	6836	0	4	P	29nt upstream of gene PMM1186;
1131452	+	TSS_011155	1000	225	0	18	I	within gene(s) PMM1186;
1131719	+	TSS_011167	1000	815	0	12	I	within gene(s) PMM1186;
1131752	+	TSS_011172	1000	157	0	1	I	within gene(s) PMM1186;
1131989	+	TSS_011184	1000	304	0	4	I	within gene(s) PMM1186;
1133995	+	TSS_011199	1000	679	0	5	I	within gene(s) PMM1188;
1135227	-	TSS_029330	1000	105	0	1	Ai	antisense to gene(s) PMM1189;
1136341	+	TSS_011212	1000	172	0	0	P	18nt upstream of gene PMM1190;
1136852	+	TSS_011222	1000	1257	0	1	P	0nt upstream of gene PMM1191;
1136930	+	TSS_011226	1000	108	0	15	I	within gene(s) PMM1191;
1136957	+	TSS_011230	1000	215	0	9	I	within gene(s) PMM1191;
1137017	+	TSS_011235	1000	261	0	19	I	within gene(s) PMM1191;
1137053	+	TSS_011243	1000	635	0	7	I	within gene(s) PMM1191;
1137073	-	TSS_029341	1000	269	0	26	Ai	antisense to gene(s) PMM1191;
1137086	+	TSS_011246	1000	215	0	3	I	within gene(s) PMM1191;
1137131	+	TSS_011251	1000	117	0	3	I	within gene(s) PMM1191;
1137146	+	TSS_011255	1000	400	0	18	I	within gene(s) PMM1191;
1137185	+	TSS_011264	1000	109	0	0	I	within gene(s) PMM1191;
1137200	+	TSS_011266	1000	449	0	6	I	within gene(s) PMM1191;
1137233	+	TSS_011271	1000	157	0	0	I	within gene(s) PMM1191;
1137285	-	TSS_029345	1000	323	0	0	Ai	antisense to gene(s) PMM1191;
1137321	-	TSS_029346	1000	329	0	0	Ai	antisense to gene(s) PMM1191;
1137338	+	TSS_011281	1000	166	0	0	I	within gene(s) PMM1191;
1137382	-	TSS_029348	1000	1731	0	1	Ai	antisense to gene(s) PMM1191;
1137394	-	TSS_029349	1000	355	0	0	Ai	antisense to gene(s) PMM1191;
1137445	-	TSS_029352	1000	110	0	0	Ai	antisense to gene(s) PMM1191;
1137842	+	TSS_011323	1000	167	0	12	I	within gene(s) PMM1191;
1137863	+	TSS_011328	1000	218	0	6	I	within gene(s) PMM1191;
1137881	+	TSS_011330	1000	183	0	4	I	within gene(s) PMM1191;
1137941	+	TSS_011342	1000	180	0	15	I	within gene(s) PMM1191;
1137992	+	TSS_011349	1000	111	0	3	I	within gene(s) PMM1191;
1138019	+	TSS_011353	1000	133	0	18	I	within gene(s) PMM1191;
1138050	-	TSS_029358	1000	215	0	0	Ai	antisense to gene(s) PMM1191;
1138064	+	TSS_011358	1000	113	0	0	I	within gene(s) PMM1191;

1138100	+	TSS_011361	1000	128	0	10 I	within gene(s) PMM1191;
1138172	+	TSS_011383	1000	608	0	63 I	within gene(s) PMM1191;
1138187	+	TSS_011387	1000	165	0	3 I	within gene(s) PMM1191;
1138235	+	TSS_011394	1000	373	0	9 I	within gene(s) PMM1191;
1138244	+	TSS_011395	1000	206	0	3 I	within gene(s) PMM1191;
1138412	+	TSS_011408	1000	1239	0	15 I	within gene(s) PMM1191;
1138442	+	TSS_011414	1000	133	0	6 I	within gene(s) PMM1191;
1138460	+	TSS_011416	1000	123	0	9 I	within gene(s) PMM1191;
1138493	+	TSS_011422	1000	203	0	15 I	within gene(s) PMM1191;
1138502	+	TSS_011423	1000	135	0	12 I	within gene(s) PMM1191;
1138535	+	TSS_011431	1000	150	0	21 I	within gene(s) PMM1191;
1138553	+	TSS_011435	1000	257	0	1 I	within gene(s) PMM1191;
1138574	+	TSS_011438	1000	123	0	3 I	within gene(s) PMM1191;
1138651	-	TSS_029368	1000	163	0	0 Ai	antisense to gene(s) PMM1191;
1138670	+	TSS_011447	1000	151	0	12 I	within gene(s) PMM1191;
1138742	+	TSS_011465	1000	218	0	87 I	within gene(s) PMM1191;
1138799	+	TSS_011480	1000	103	0	24 I	within gene(s) PMM1191;
1138805	-	TSS_029371	1000	170	0	1 Ai	antisense to gene(s) PMM1191;
1138862	-	TSS_029373	1000	813	0	0 Ai	antisense to gene(s) PMM1191;
1138882	-	TSS_029376	1000	250	0	5 Ai	antisense to gene(s) PMM1191;
1138904	-	TSS_029378	1000	175	0	0 Ai	antisense to gene(s) PMM1191;
1138932	-	TSS_029379	1000	140	0	0 Ai	antisense to gene(s) PMM1191;
1139305	-	TSS_029382	1000	103	0	0 I	within gene(s) PMM1192;
1139830	+	TSS_011485	1000	319	0	0 PAi	95nt upstream of gene PMM1193; antisense to gene(s) PMM1192;
1142062	+	TSS_011489	1000	121	0	9 I	within gene(s) PMM1196;
1147383	-	TSS_029389	1000	125	0	0 I	within gene(s) PMM1199;
1150601	-	TSS_029399	1000	103	0	0 I	within gene(s) PMM1202;
1151357	+	TSS_011519	1000	130	0	5 Ai	antisense to gene(s) PMM1203;
1151532	-	TSS_029403	1000	101	0	5 I	within gene(s) PMM1203;
1152750	-	TSS_029408	1000	262	0	0 I	within gene(s) PMM1204;
1152998	-	TSS_029409	1000	358	0	1 IP	within gene(s) PMM1205; 88nt upstream of gene PMM1204;
1154175	-	TSS_029418	1000	168	0	4 P	18nt upstream of gene PMM1205;
1157183	+	TSS_011529	1000	101	0	0 Ai	antisense to gene(s) PMM1208;
1157220	+	TSS_011530	1000	620	0	0 Ai	antisense to gene(s) PMM1208;
1158156	-	TSS_029428	1000	145	0	0 P	14nt upstream of gene PMM1208;
1158392	+	TSS_011532	1000	103	0	0 Ai	antisense to gene(s) PMM1209;
1164607	-	TSS_029444	1000	108	0	2 Ai	antisense to gene(s) PMM1215;
1168987	-	TSS_029453	1000	108	0	0 Ai	antisense to gene(s) PMM1219;
1175722	+	TSS_011582	1000	133	0	1 I	within gene(s) PMM1225;
1181121	+	TSS_011589	1000	140	0	0 P	27nt upstream of gene PMM1229;
1181350	+	TSS_011590	1000	151	0	0 I	within gene(s) PMM1229;
1181446	-	TSS_029472	1000	341	0	2 Ai	antisense to gene(s) PMM1229;
1183984	+	TSS_011594	1000	653	0	6 I	within gene(s) PMM1232;
1186173	+	TSS_011598	1000	571	0	14 I	within gene(s) PMM1234;
1186359	-	TSS_029484	1000	448	0	2 Ai	antisense to gene(s) PMM1234;
1186648	-	TSS_029486	1000	115	0	4 Ai	antisense to gene(s) PMM1234;
1186823	+	TSS_011606	1000	3760	0	2 I	within gene(s) PMM1234;
1187310	+	TSS_011610	1000	135	0	0 I	within gene(s) PMM1235;
1187583	+	TSS_011614	1000	105	0	0 I	within gene(s) PMM1235;
1187821	+	TSS_011617	1000	413	0	3 IP	within gene(s) PMM1235; 110nt upstream of gene PMM1236;
1192382	+	TSS_011627	1000	234	0	8 IP	within gene(s) PMM1239; 39nt upstream of gene PMM1240;
1192406	+	TSS_011630	1000	523	0	3 IP	within gene(s) PMM1239; 15nt upstream of gene PMM1240;
1199524	+	TSS_011647	1000	102	0	0 Ai	antisense to gene(s) PMM1247;
1201748	-	TSS_029527	1000	189	0	1 I	within gene(s) PMM1249;
1202453	-	TSS_029535	1000	189	0	1 I	within gene(s) PMM1250;
1203802	+	TSS_011652	1000	514	0	1 Ai	antisense to gene(s) PMM1251;
1204621	-	TSS_029554	1000	395	0	0 P	82nt upstream of gene PMM1251;
1206887	+	TSS_011663	1000	179	0	0 Ai	antisense to gene(s) PMM1254;
1208693	-	TSS_029568	1000	107	0	0 P	157nt upstream of gene PMM1255;
1209541	-	TSS_029570	1000	1411	0	3 I	within gene(s) PMM1256;
1211786	+	TSS_011675	1000	220	0	0 I	within gene(s) PMM1258;
1212300	-	TSS_029583	1000	125	0	0 Ai	antisense to gene(s) PMM1258;
1212532	+	TSS_011678	1000	197	0	1 IP	within gene(s) PMM1258; 148nt upstream of gene PMM1259;
1212721	-	TSS_029585	1000	182	0	2 Ai	antisense to gene(s) PMM1258 PMM1259;
1212906	+	TSS_011680	1000	415	0	0 I	within gene(s) PMM1259;
1213317	+	TSS_011691	1000	856	0	4 I	within gene(s) PMM1259;
1214272	+	TSS_011695	1000	128	0	0 I	within gene(s) PMM1260;
1214339	+	TSS_011696	1000	125	0	0 I	within gene(s) PMM1260;
1214724	+	TSS_011699	1000	956	0	0 IP	within gene(s) PMM1260; 140nt upstream of gene PMM1261;
1214740	+	TSS_011700	1000	111	0	0 IP	within gene(s) PMM1260; 124nt upstream of gene PMM1261;
1214815	+	TSS_011701	1000	125	0	0 IP	within gene(s) PMM1260; 49nt upstream of gene PMM1261;
1215731	-	TSS_029592	1000	111	0	2 Ai	antisense to gene(s) PMM1261;
1216376	-	TSS_029597	1000	148	0	3 I	within gene(s) PMM1262;
1216548	+	TSS_011712	1000	122	0	0 Ai	antisense to gene(s) PMM1262;
1216690	+	TSS_011713	1000	125	0	0 Ai	antisense to gene(s) PMM1262;
1218027	+	TSS_011715	1000	119	0	0 Ai	antisense to gene(s) PMM1264;
1218040	+	TSS_011716	1000	119	0	0 Ai	antisense to gene(s) PMM1264;

1218110	+	TSS_011720	1000	131	0	1	Ai	antisense to gene(s) PMM1264;
1218175	-	TSS_029618	1000	420	0	21	I	within gene(s) PMM1264;
1218209	+	TSS_011722	1000	110	0	0	Ai	antisense to gene(s) PMM1264;
1218256	-	TSS_029631	1000	120	0	33	I	within gene(s) PMM1264;
1218313	-	TSS_029643	1000	204	0	15	I	within gene(s) PMM1264;
1218427	-	TSS_029659	1000	102	0	1	I	within gene(s) PMM1264;
1218456	+	TSS_011728	1000	105	0	0	Ai	antisense to gene(s) PMM1264;
1218465	-	TSS_029667	1000	335	0	27	I	within gene(s) PMM1264;
1218505	-	TSS_029671	1000	144	0	0	I	within gene(s) PMM1264;
1218556	-	TSS_029676	1000	215	0	3	I	within gene(s) PMM1264;
1218563	+	TSS_011730	1000	245	0	4	Ai	antisense to gene(s) PMM1264;
1218616	-	TSS_029681	1000	119	0	3	I	within gene(s) PMM1264;
1218623	+	TSS_011733	1000	1255	0	1	Ai	antisense to gene(s) PMM1264;
1218649	-	TSS_029684	1000	1457	0	24	I	within gene(s) PMM1264;
1218735	+	TSS_011734	1000	132	0	1	Ai	antisense to gene(s) PMM1264;
1218757	-	TSS_029701	1000	236	0	0	I	within gene(s) PMM1264;
1218787	-	TSS_029704	1000	101	0	0	I	within gene(s) PMM1264;
1218832	-	TSS_029705	1000	233	0	0	I	within gene(s) PMM1264;
1218868	-	TSS_029707	1000	165	0	6	I	within gene(s) PMM1264;
1218883	-	TSS_029711	1000	110	0	6	I	within gene(s) PMM1264;
1218904	-	TSS_029716	1000	279	0	12	I	within gene(s) PMM1264;
1218939	+	TSS_011737	1000	357	0	0	Ai	antisense to gene(s) PMM1264;
1218943	-	TSS_029724	1000	366	0	48	I	within gene(s) PMM1264;
1219000	-	TSS_029735	1000	104	0	27	I	within gene(s) PMM1264;
1219090	-	TSS_029742	1000	174	0	0	I	within gene(s) PMM1264;
1219102	-	TSS_029743	1000	101	0	6	I	within gene(s) PMM1264;
1219126	-	TSS_029746	1000	230	0	15	I	within gene(s) PMM1264;
1219168	-	TSS_029757	1000	362	0	50	I	within gene(s) PMM1264;
1219255	+	TSS_011741	1000	204	0	1	Ai	antisense to gene(s) PMM1264;
1219300	-	TSS_029777	1000	120	0	21	I	within gene(s) PMM1264;
1219348	-	TSS_029786	1000	133	0	27	I	within gene(s) PMM1264;
1219417	-	TSS_029792	1000	545	0	30	I	within gene(s) PMM1264;
1219468	-	TSS_029804	1000	197	0	15	I	within gene(s) PMM1264;
1219522	-	TSS_029813	1000	192	0	6	I	within gene(s) PMM1264;
1219666	-	TSS_029825	1000	109	0	10	I	within gene(s) PMM1264;
1219705	-	TSS_029831	1000	173	0	1	I	within gene(s) PMM1264;
1219762	-	TSS_029832	1000	406	0	0	I	within gene(s) PMM1264;
1219801	-	TSS_029833	1000	334	0	1	P	24nt upstream of gene PMM1264;
1219908	-	TSS_029835	1000	315	0	0	IP	within gene(s) PMM1265; 131nt upstream of gene PMM1264;
1220160	+	TSS_011747	1000	179	0	5	Ai	antisense to gene(s) PMM1265;
1221551	-	TSS_029847	1000	130	0	2	IP	within gene(s) PMM1267; 57nt upstream of gene PMM1266;
1221738	-	TSS_029851	1000	109	0	3	IP	within gene(s) PMM1267; 244nt upstream of gene PMM1266;
1223096	+	TSS_011758	1000	133	0	0	Ai	antisense to gene(s) PMM1269;
1223575	-	TSS_029861	1000	166	0	3	I	within gene(s) PMM1269;
1224017	-	TSS_029869	1000	5801	0	10	P	16nt upstream of gene PMM1269;
1224107	+	TSS_011763	1000	568	0	2	Ai	antisense to gene(s) PMM1270;
1224306	-	TSS_029878	1000	480	0	6	I	within gene(s) PMM1270;
1224483	+	TSS_011767	1000	1891	0	1	Ai	antisense to gene(s) PMM1270;
1224903	-	TSS_029922	1000	113	0	38	I	within gene(s) PMM1270;
1225097	+	TSS_011769	1000	497	0	6	P	51nt upstream of gene PMM1271;
1225100	-	TSS_029932	1000	931	0	2	P	20nt upstream of gene PMM1270;
1225179	+	TSS_011771	1000	111	0	0	I	within gene(s) PMM1271;
1226226	+	TSS_011774	1000	178	0	0	Ai	antisense to gene(s) PMM1272;
1226290	+	TSS_011775	1000	112	0	0	PAi	248nt upstream of gene PMM1273; antisense to gene(s) PMM1272;
1226399	+	TSS_011776	1000	2758	0	2	PAi	139nt upstream of gene PMM1273; antisense to gene(s) PMM1272;
1226493	-	TSS_029939	1000	7114	0	2	P	23nt upstream of gene PMM1272;
1228410	-	TSS_029943	1000	104	0	0	Ai	antisense to gene(s) PMM1274;
1228488	-	TSS_029944	1000	106	0	0	Ai	antisense to gene(s) PMM1274;
1228824	+	TSS_011784	1000	1010	0	3	P	13nt upstream of gene PMM1276;
1229056	+	TSS_011785	1000	173	0	0	P	15nt upstream of gene PMM1277;
1229316	+	TSS_011788	1000	796	0	6	Ai	antisense to gene(s) PMM1278;
1229883	-	TSS_029952	1000	123	0	1	IP	within gene(s) PMM1279; 18nt upstream of gene PMM1278;
1230756	-	TSS_029968	1000	166	0	0	I	within gene(s) PMM1280;
1230808	-	TSS_029969	1000	462	0	2	I	within gene(s) PMM1280;
1231095	+	TSS_011796	1000	107	0	0	Ai	antisense to gene(s) PMM1280;
1231722	-	TSS_029976	1000	797	0	2	IP	within gene(s) PMM1281; 194nt upstream of gene PMM1280;
1233089	+	TSS_011805	1000	2218	0	1	Ai	antisense to gene(s) PMM1283;
1233102	+	TSS_011806	1000	217	0	1	Ai	antisense to gene(s) PMM1283;
1233159	+	TSS_011808	1000	135	0	0	Ai	antisense to gene(s) PMM1283;
1233255	+	TSS_011810	1000	179	0	1	Ai	antisense to gene(s) PMM1283;
1233266	-	TSS_029989	1000	1116	0	39	I	within gene(s) PMM1283;
1233285	+	TSS_011812	1000	152	0	0	Ai	antisense to gene(s) PMM1283;
1233300	+	TSS_011813	1000	415	0	0	Ai	antisense to gene(s) PMM1283;
1233302	-	TSS_029999	1000	1092	0	12	I	within gene(s) PMM1283;
1233311	+	TSS_011815	1000	717	0	1	Ai	antisense to gene(s) PMM1283;
1233320	-	TSS_030001	1000	543	0	0	I	within gene(s) PMM1283;
1233322	+	TSS_011817	1000	347	0	1	Ai	antisense to gene(s) PMM1283;

1233332	-	TSS_030002	1000	343	0	0	I	within gene(s) PMM1283;
1233365	-	TSS_030010	1000	1466	0	66	I	within gene(s) PMM1283;
1233366	+	TSS_011818	1000	133	0	0	Ai	antisense to gene(s) PMM1283;
1233384	+	TSS_011820	1000	210	0	1	Ai	antisense to gene(s) PMM1283;
1233399	+	TSS_011821	1000	595	0	1	Ai	antisense to gene(s) PMM1283;
1233419	-	TSS_030023	1000	450	0	0	I	within gene(s) PMM1283;
1233428	-	TSS_030024	1000	359	0	0	I	within gene(s) PMM1283;
1233437	-	TSS_030025	1000	310	0	0	I	within gene(s) PMM1283;
1233465	+	TSS_011826	1000	280	0	0	Ai	antisense to gene(s) PMM1283;
1233473	+	TSS_011827	1000	364	0	4	Ai	antisense to gene(s) PMM1283;
1233533	-	TSS_030055	1000	1491	0	141	I	within gene(s) PMM1283;
1233611	-	TSS_030074	1000	618	0	30	I	within gene(s) PMM1283;
1233652	-	TSS_030085	1000	670	0	3	I	within gene(s) PMM1283;
1233669	+	TSS_011829	1000	102	0	0	Ai	antisense to gene(s) PMM1283;
1233677	-	TSS_030089	1000	964	0	6	I	within gene(s) PMM1283;
1233686	-	TSS_030090	1000	343	0	0	I	within gene(s) PMM1283;
1233701	-	TSS_030092	1000	254	0	3	I	within gene(s) PMM1283;
1233722	-	TSS_030098	1000	1087	0	39	I	within gene(s) PMM1283;
1233764	-	TSS_030111	1000	685	0	9	I	within gene(s) PMM1283;
1233817	-	TSS_030114	1000	44852	0	6	P	17nt upstream of gene PMM1283;
1235226	-	TSS_030137	1000	11729	0	4	P	16nt upstream of gene PMM1285;
1235758	+	TSS_011842	1000	200	0	0	Ai	antisense to gene(s) PMM1286;
1235915	-	TSS_030155	1000	122	0	14	I	within gene(s) PMM1286;
1236134	-	TSS_030164	1000	163	0	24	I	within gene(s) PMM1286;
1236677	-	TSS_030188	1000	135	0	3	I	within gene(s) PMM1286;
1236777	-	TSS_030193	1000	5371	0	7	P	16nt upstream of gene PMM1286;
1236798	+	TSS_011847	1000	110	0	0	Ad	antisense to gene(s) PMM1287 (14nt downstream);
1238666	-	TSS_030212	1000	284	0	0	I	within gene(s) PMM1287;
1239039	+	TSS_011850	1000	498	0	0	P	30nt upstream of gene PMM1288;
1239358	-	TSS_030214	1000	225	0	1	Ai	antisense to gene(s) PMM1288;
1239381	+	TSS_011868	1000	104	0	12	I	within gene(s) PMM1288;
1239426	+	TSS_011874	1000	102	0	7	I	within gene(s) PMM1288;
1239678	+	TSS_011885	1000	139	0	3	I	within gene(s) PMM1288;
1239822	+	TSS_011899	1000	293	0	37	I	within gene(s) PMM1288;
1239949	-	TSS_030221	1000	392	0	0	Ai	antisense to gene(s) PMM1288;
1240190	+	TSS_011907	1000	961	0	1	Ai	antisense to gene(s) PMM1289;
1240340	+	TSS_011916	1000	215	0	3	Ai	antisense to gene(s) PMM1289;
1240449	-	TSS_030242	1000	112	0	9	I	within gene(s) PMM1289;
1240490	-	TSS_030246	1000	735	0	7	I	within gene(s) PMM1289;
1240521	-	TSS_030250	1000	221	0	12	I	within gene(s) PMM1289;
1240674	-	TSS_030265	1000	115	0	13	I	within gene(s) PMM1289;
1240780	+	TSS_011921	1000	188	0	1	Ai	antisense to gene(s) PMM1289;
1240796	+	TSS_011922	1000	127	0	0	Ai	antisense to gene(s) PMM1289;
1240970	-	TSS_030279	1000	217	0	1	I	within gene(s) PMM1289;
1241031	-	TSS_030283	1000	109	0	1	I	within gene(s) PMM1289;
1241067	-	TSS_030286	1000	11084	0	2	P	18nt upstream of gene PMM1289;
1241165	+	TSS_011928	1000	162	0	3	Ad	antisense to gene(s) PMM1290 (26nt downstream);
1241345	+	TSS_011931	1000	140	0	1	Ai	antisense to gene(s) PMM1290;
1241474	-	TSS_030293	1000	104	0	1	I	within gene(s) PMM1290;
1242168	+	TSS_011938	1000	306	0	0	Ai	antisense to gene(s) PMM1290;
1242445	+	TSS_011939	1000	1042	0	0	Ai	antisense to gene(s) PMM1290;
1242497	+	TSS_011940	1000	257	0	0	Ai	antisense to gene(s) PMM1290;
1243042	-	TSS_030304	1000	117	0	6	Ai	antisense to gene(s) PMM1291;
1243222	+	TSS_011948	1000	153	0	8	I	within gene(s) PMM1291;
1243570	-	TSS_030306	1000	142	0	0	Ai	antisense to gene(s) PMM1291;
1244009	+	TSS_011958	1000	539	0	1	Ai	antisense to gene(s) PMM1292;
1245509	+	TSS_011966	1000	2870	0	1	P	17nt upstream of gene PMM1293;
1245566	+	TSS_011968	1000	374	0	5	I	within gene(s) PMM1293;
1245584	+	TSS_011971	1000	329	0	4	I	within gene(s) PMM1293;
1245700	+	TSS_011979	1000	690	0	2	I	within gene(s) PMM1293;
1245742	+	TSS_011981	1000	101	0	6	I	within gene(s) PMM1293;
1245769	+	TSS_011985	1000	201	0	27	I	within gene(s) PMM1293;
1245796	+	TSS_011989	1000	1764	0	6	I	within gene(s) PMM1293;
1245811	+	TSS_011991	1000	709	0	3	I	within gene(s) PMM1293;
1245841	+	TSS_011999	1000	236	0	16	I	within gene(s) PMM1293;
1245861	+	TSS_012002	1000	105	0	0	I	within gene(s) PMM1293;
1246003	+	TSS_012009	1000	175	0	0	IP	within gene(s) PMM1293; 170nt upstream of gene PMM1294;
1246188	+	TSS_012010	1000	175	0	0	I	within gene(s) PMM1294;
1246197	+	TSS_012011	1000	149	0	0	I	within gene(s) PMM1294;
1246245	+	TSS_012021	1000	204	0	39	I	within gene(s) PMM1294;
1246263	+	TSS_012025	1000	161	0	6	I	within gene(s) PMM1294;
1246320	+	TSS_012031	1000	126	0	0	I	within gene(s) PMM1294;
1246440	-	TSS_030316	1000	117	0	0	Ai	antisense to gene(s) PMM1294;
1246482	+	TSS_012047	1000	166	0	10	IP	within gene(s) PMM1294; 248nt upstream of gene PMM1295;
1247681	-	TSS_030323	1000	290	0	2	P	20nt upstream of gene PMM1296;
1250117	+	TSS_012059	1000	138	0	0	Ai	antisense to gene(s) PMM1299;
1250130	-	TSS_030377	1000	2545	0	6	IP	within gene(s) PMM1299; 75nt upstream of gene PMM1298;

1250468	-	TSS_030384	1000	474	0	5	I	within gene(s) PMM1299;
1251088	+	TSS_012062	1000	265	0	0	P	15nt upstream of gene PMM1300;
1252018	+	TSS_012065	1000	120	0	0	I	within gene(s) PMM1300;
1252148	+	TSS_012068	1000	137	0	2	I	within gene(s) PMM1300;
1252609	-	TSS_030401	1000	142	0	16	O	-
1252727	-	TSS_030416	1000	4117	0	5	O	-
1252970	+	TSS_012079	1000	1085	0	5	P	32nt upstream of gene PMM1301;
1253102	+	TSS_012081	1000	280	0	0	I	within gene(s) PMM1301;
1256224	+	TSS_012096	1000	144	0	1	Ai	antisense to gene(s) PMM1303;
1258142	-	TSS_030434	1000	244	0	12	P	16nt upstream of gene PMM1304;
1258258	+	TSS_012102	1000	1041	0	6	P	151nt upstream of gene PMM1305;
1259122	-	TSS_030445	1000	225	0	1	Ai	antisense to gene(s) PMM1305;
1261120	+	TSS_012130	1000	157	0	0	IP	within gene(s) PMM1307; 179nt upstream of gene PMM1308;
1262176	+	TSS_012136	1000	1701	0	2	P	26nt upstream of gene PMM1309;
1262799	+	TSS_012174	1000	158	0	22	I	within gene(s) PMM1309;
1262859	+	TSS_012183	1000	169	0	9	I	within gene(s) PMM1309;
1262883	+	TSS_012187	1000	134	0	0	I	within gene(s) PMM1309;
1262904	+	TSS_012188	1000	105	0	0	I	within gene(s) PMM1309;
1263674	+	TSS_012210	1000	1136	0	1	I	within gene(s) PMM1310;
1264789	-	TSS_030468	1000	105	0	0	I	within gene(s) PMM1311;
1264868	+	TSS_012221	1000	192	0	4	Ai	antisense to gene(s) PMM1311;
1265561	+	TSS_012225	1000	19854	0	4	I	within gene(s) PMM1312;
1265689	+	TSS_012229	1000	156	0	0	I	within gene(s) PMM1312;
1266112	+	TSS_012249	1000	121	0	12	I	within gene(s) PMM1312;
1266213	-	TSS_030474	1000	213	0	1	Ai	antisense to gene(s) PMM1312;
1266220	+	TSS_012256	1000	101	0	10	I	within gene(s) PMM1312;
1266690	+	TSS_012276	1000	19857	0	2	P	20nt upstream of gene PMM1313;
1266702	+	TSS_012278	1000	465	0	0	P	8nt upstream of gene PMM1313;
1266713	+	TSS_012279	1000	251	0	0	I	within gene(s) PMM1313;
1266750	+	TSS_012286	1000	222	0	17	I	within gene(s) PMM1313;
1266761	+	TSS_012288	1000	110	0	27	I	within gene(s) PMM1313;
1266866	+	TSS_012299	1000	186	0	12	I	within gene(s) PMM1313;
1266890	+	TSS_012302	1000	148	0	33	I	within gene(s) PMM1313;
1267025	+	TSS_012329	1000	158	0	18	I	within gene(s) PMM1313;
1267067	+	TSS_012334	1000	221	0	45	I	within gene(s) PMM1313;
1267140	-	TSS_030482	1000	164	0	1	Ai	antisense to gene(s) PMM1313;
1267176	-	TSS_030484	1000	157	0	0	Ai	antisense to gene(s) PMM1313;
1267212	-	TSS_030485	1000	196	0	0	Ai	antisense to gene(s) PMM1313;
1267250	+	TSS_012358	1000	136	0	36	IP	within gene(s) PMM1313; 156nt upstream of gene PMM1314;
1267775	+	TSS_012422	1000	138	0	57	I	within gene(s) PMM1314;
1268104	+	TSS_012434	1000	3560	0	2	P	24nt upstream of gene PMM1315;
1268156	-	TSS_030495	1000	178	0	0	Ai	antisense to gene(s) PMM1315;
1268473	+	TSS_012467	1000	142	0	36	I	within gene(s) PMM1315;
1270161	+	TSS_012504	1000	358	0	7	P	15nt upstream of gene PMM1317;
1270182	+	TSS_012506	1000	101	0	0	I	within gene(s) PMM1317;
1270931	-	TSS_030509	1000	202	0	9	I	within gene(s) PMM1318;
1272067	-	TSS_030516	1000	102307	0	8	P	65nt upstream of gene PMM1321;
1273518	-	TSS_030523	1000	2327	0	3	Ai	antisense to gene(s) PMM1322;
1274764	+	TSS_012534	1000	751	0	1	P	86nt upstream of gene PMM1323;
1275301	-	TSS_030533	1000	193	0	0	Ai	antisense to gene(s) PMM1323;
1275591	+	TSS_012543	1000	145	0	0	I	within gene(s) PMM1323;
1276011	-	TSS_030536	1000	835	0	2	Ai	antisense to gene(s) PMM1323;
1276210	+	TSS_012547	1000	326	0	0	I	within gene(s) PMM1324;
1276620	-	TSS_030541	1000	357	0	0	Ai	antisense to gene(s) PMM1324;
1276627	-	TSS_030542	1000	204	0	0	Ai	antisense to gene(s) PMM1324;
1276641	+	TSS_012552	1000	499	0	0	I	within gene(s) PMM1324;
1278318	+	TSS_012563	1000	578	0	2	IP	within gene(s) PMM1326; 106nt upstream of gene PMM1327;
1281106	-	TSS_030551	1000	524	0	1	IP	within gene(s) PMM1330; 132nt upstream of gene PMM1329;
1281349	-	TSS_030556	1000	147	0	1	P	30nt upstream of gene PMM1330;
1282915	+	TSS_012595	1000	103	0	1	I	within gene(s) PMM1332;
1284193	-	TSS_030568	1000	201	0	0	I	within gene(s) PMM1333;
1284754	+	TSS_012608	1000	186	0	1	Ai	antisense to gene(s) PMM1334;
1285667	-	TSS_030572	1000	183	0	3	I	within gene(s) PMM1335;
1285747	-	TSS_030575	1000	127	0	0	I	within gene(s) PMM1335;
1286762	+	TSS_012620	1000	394	0	6	Ai	antisense to gene(s) PMM1337;
1286766	-	TSS_030582	1000	157	0	1	IP	within gene(s) PMM1337; 87nt upstream of gene PMM1336;
1286829	-	TSS_030585	1000	121	0	2	IP	within gene(s) PMM1337; 150nt upstream of gene PMM1336;
1287550	-	TSS_030588	1000	167	0	0	IP	within gene(s) PMM1338; 17nt upstream of gene PMM1337;
1287887	-	TSS_030606	1000	440	0	10	I	within gene(s) PMM1338;
1287977	-	TSS_030608	1000	284	0	0	I	within gene(s) PMM1338;
1288162	+	TSS_012630	1000	207	0	6	Ai	antisense to gene(s) PMM1338;
1288706	-	TSS_030623	1000	106	0	2	I	within gene(s) PMM1338;
1288975	-	TSS_030631	1000	223	0	2	I	within gene(s) PMM1338;
1289984	-	TSS_030645	1000	101	0	2	I	within gene(s) PMM1339;
1291245	-	TSS_030653	1000	174	0	0	Ai	antisense to gene(s) PMM1340;
1291318	-	TSS_030654	1000	273	0	0	Ai	antisense to gene(s) PMM1340;
1291478	+	TSS_012654	1000	1552	0	2	I	within gene(s) PMM1340;

1292169	-	TSS_030660	1000	143	0	2	Ai	antisense to gene(s) PMM1341;
1293363	+	TSS_012679	1000	131	0	0	I	within gene(s) PMM1341;
1293438	+	TSS_012684	1000	396	0	7	I	within gene(s) PMM1341;
1293604	+	TSS_012689	1000	114	0	0	I	within gene(s) PMM1341;
1293828	+	TSS_012691	1000	212	0	2	I	within gene(s) PMM1341;
1294828	-	TSS_030688	1000	223	0	3	I	within gene(s) PMM1342;
1295467	-	TSS_030717	1000	182	0	6	I	within gene(s) PMM1342;
1295522	-	TSS_030721	1000	158	0	0	P	13nt upstream of gene PMM1342;
1295547	-	TSS_030722	1000	170	0	3	P	38nt upstream of gene PMM1342;
1295943	-	TSS_030726	1000	114	0	0	P	48nt upstream of gene PMM1343;
1295976	+	TSS_012701	1000	1194	0	10	P	65nt upstream of gene PMM1344;
1296395	+	TSS_012721	1000	8987	0	7	IP	within gene(s) PMM1344; 96nt upstream of gene PMM1345;
1296404	+	TSS_012724	1000	142	0	4	IP	within gene(s) PMM1344; 87nt upstream of gene PMM1345;
1296628	-	TSS_030733	1000	290	0	0	Ai	antisense to gene(s) PMM1345;
1297508	-	TSS_030736	1000	486	0	6	P	89nt upstream of gene PMM1346;
1299151	+	TSS_012745	1000	612	0	0	Ai	antisense to gene(s) PMM1348;
1299740	+	TSS_012746	1000	372	0	0	P	25nt upstream of gene PMM1349;
1299756	+	TSS_012747	1000	148	0	2	P	9nt upstream of gene PMM1349;
1300005	+	TSS_012749	1000	177	0	5	I	within gene(s) PMM1349;
1300564	-	TSS_030748	1000	134	0	0	Ai	antisense to gene(s) PMM1349;
1300739	+	TSS_012757	1000	419	0	0	P	24nt upstream of gene PMM1350;
1300764	+	TSS_012760	1000	13106	0	2	I	within gene(s) PMM1350;
1300820	+	TSS_012764	1000	257	0	0	I	within gene(s) PMM1350;
1300829	+	TSS_012765	1000	114	0	0	I	within gene(s) PMM1350;
1300862	+	TSS_012771	1000	247	0	21	I	within gene(s) PMM1350;
1300907	+	TSS_012782	1000	290	0	39	I	within gene(s) PMM1350;
1300946	+	TSS_012792	1000	184	0	15	I	within gene(s) PMM1350;
1300973	+	TSS_012795	1000	237	0	3	I	within gene(s) PMM1350;
1301021	+	TSS_012814	1000	427	0	39	I	within gene(s) PMM1350;
1301120	+	TSS_012831	1000	329	0	33	I	within gene(s) PMM1350;
1301738	-	TSS_030758	1000	313	0	1	P	22nt upstream of gene PMM1351;
1302007	-	TSS_030766	1000	343	0	22	I	within gene(s) PMM1352;
1302034	-	TSS_030771	1000	585	0	30	I	within gene(s) PMM1352;
1302064	-	TSS_030780	1000	339	0	4	I	within gene(s) PMM1352;
1302079	-	TSS_030784	1000	210	0	7	I	within gene(s) PMM1352;
1302130	-	TSS_030787	1000	21809	0	2	P	33nt upstream of gene PMM1352;
1302279	-	TSS_030791	1000	212	0	4	IP	within gene(s) PMM1353; 182nt upstream of gene PMM1352;
1304163	+	TSS_012843	1000	755	0	0	Ai	antisense to gene(s) PMM1354;
1304348	+	TSS_012845	1000	104	0	1	Ai	antisense to gene(s) PMM1354;
1304765	-	TSS_030839	1000	1411	0	0	P	19nt upstream of gene PMM1354;
1304776	-	TSS_030840	1000	435	0	4	P	30nt upstream of gene PMM1354;
1307622	+	TSS_012863	1000	703	0	3	O	-
1309146	-	TSS_030851	1000	163	0	0	I	within gene(s) PMM1360;
1309784	+	TSS_012869	1000	139	0	2	P	56nt upstream of gene PMM1361;
1313108	+	TSS_012881	1000	1118	0	3	P	16nt upstream of gene PMM1365;
1313370	+	TSS_012905	1000	139	0	42	I	within gene(s) PMM1365;
1313421	+	TSS_012914	1000	209	0	1	IAd	within gene(s) PMM1365; antisense to gene(s) PMM1366 (22nt downstream);
1314413	-	TSS_030862	1000	114	0	1	P	240nt upstream of gene PMM1366;
1315687	-	TSS_030870	1000	141	0	0	IP	within gene(s) PMM1369; 193nt upstream of gene PMM1368;
1316344	-	TSS_030876	1000	901	0	5	P	10nt upstream of gene PMM1369;
1317691	-	TSS_030884	1000	128	0	0	P	27nt upstream of gene PMM1370;
1317860	+	TSS_012926	1000	123	0	2	O	-
1318282	-	TSS_030885	1000	379	0	0	O	-
1319591	+	TSS_012931	1000	4345	0	2	O	-
1319627	+	TSS_012937	1000	124	0	8	O	-
1319858	-	TSS_030887	1000	123	0	0	O	-
1319966	+	TSS_012944	1000	691	0	2	P	13nt upstream of gene PMM1372;
1319980	+	TSS_012945	1000	2558	0	5	I	within gene(s) PMM1372;
1320260	-	TSS_030890	1000	285	0	1	Ad	antisense to gene(s) PMM1372 (4nt downstream);
1321010	+	TSS_012949	1000	5885	0	2	O	-
1322462	-	TSS_030899	1000	575	0	1	O	-
1322954	-	TSS_030901	1000	109	0	0	P	15nt upstream of gene PMM1374;
1323803	+	TSS_012959	1000	103	0	6	P	38nt upstream of gene PMM1376;
1324248	+	TSS_012967	1000	530	0	0	P	19nt upstream of gene PMM1377;
1326668	-	TSS_030911	1000	15045	0	2	O	-
1328576	+	TSS_012984	1000	120	0	0	O	-
1328956	-	TSS_030922	1000	4964	0	2	O	-
1329106	+	TSS_012987	1000	560	0	3	O	-
1330723	-	TSS_030926	1000	130	0	0	Ai	antisense to gene(s) PMM1381;
1332432	+	TSS_013000	1000	2552	0	2	O	-
1332606	-	TSS_030934	1000	112	0	4	O	-
1332932	-	TSS_030935	1000	153	0	2	I	within gene(s) PMM1383;
1333562	-	TSS_030940	1000	165	0	2	P	188nt upstream of gene PMM1383;
1334143	+	TSS_013005	1000	825	0	2	O	-
1334317	+	TSS_013007	1000	124	0	0	O	-
1334431	+	TSS_013013	1000	104	0	12	P	182nt upstream of gene PMM1384;
1335359	+	TSS_013015	1000	15494	0	2	O	-

1336436	+	TSS_013023	1000	800	0	1	O	-
1338319	-	TSS_030954	1000	255	0	5	Ai	antisense to gene(s) PMM1386;
1338981	+	TSS_013031	1000	230	0	0	P	17nt upstream of gene PMM1387;
1339834	-	TSS_030959	1000	428	0	0	P	32nt upstream of gene PMM1388;
1341205	-	TSS_030964	1000	402	0	0	P	20nt upstream of gene PMM1390;
1341449	+	TSS_013039	1000	179	0	0	P	15nt upstream of gene PMM1391;
1343374	+	TSS_013043	1000	162	0	0	I	within gene(s) PMM1394;
1343856	-	TSS_030971	1000	3349	0	7	O	-
1344024	-	TSS_030976	1000	219	0	3	O	-
1345064	-	TSS_030986	1000	457	0	2	I	within gene(s) PMM1396;
1345151	-	TSS_030993	1000	121	0	12	IP	within gene(s) PMM1397; 84nt upstream of gene PMM1396;
1345196	-	TSS_031006	1000	391	0	45	IP	within gene(s) PMM1397; 129nt upstream of gene PMM1396;
1345256	-	TSS_031019	1000	596	0	3	IP	within gene(s) PMM1397; 189nt upstream of gene PMM1396;
1345471	-	TSS_031032	1000	186	0	1	IP	within gene(s) PMM1398; 113nt upstream of gene PMM1397;
1345537	-	TSS_031040	1000	192	0	2	IP	within gene(s) PMM1398; 179nt upstream of gene PMM1397;
1345584	-	TSS_031044	1000	127	0	11	IP	within gene(s) PMM1399; 17nt upstream of gene PMM1398;
1345641	-	TSS_031050	1000	112	0	0	IP	within gene(s) PMM1399; 74nt upstream of gene PMM1398;
1345650	-	TSS_031051	1000	111	0	4	IP	within gene(s) PMM1399; 83nt upstream of gene PMM1398;
1345665	-	TSS_031056	1000	517	0	4	IP	within gene(s) PMM1399; 98nt upstream of gene PMM1398;
1346141	+	TSS_013055	1000	149	0	1	Ai	antisense to gene(s) PMM1400;
1346194	-	TSS_031066	1000	110	0	3	I	within gene(s) PMM1400;
1346272	-	TSS_031073	1000	112	0	2	I	within gene(s) PMM1400;
1346284	-	TSS_031077	1000	127	0	12	I	within gene(s) PMM1400;
1346308	-	TSS_031080	1000	209	0	6	I	within gene(s) PMM1400;
1346341	-	TSS_031084	1000	123	0	9	I	within gene(s) PMM1400;
1346356	-	TSS_031088	1000	373	0	15	I	within gene(s) PMM1400;
1346398	-	TSS_031098	1000	952	0	15	I	within gene(s) PMM1400;
1346431	-	TSS_031107	1000	1580	0	27	I	within gene(s) PMM1400;
1346455	-	TSS_031112	1000	366	0	3	I	within gene(s) PMM1400;
1346470	-	TSS_031115	1000	919	0	6	I	within gene(s) PMM1400;
1346485	-	TSS_031117	1000	386	0	0	I	within gene(s) PMM1400;
1346500	-	TSS_031120	1000	813	0	6	I	within gene(s) PMM1400;
1346515	-	TSS_031124	1000	6319	0	12	I	within gene(s) PMM1400;
1346536	-	TSS_031129	1000	328	0	0	I	within gene(s) PMM1400;
1346593	-	TSS_031135	1000	590	0	19	I	within gene(s) PMM1400;
1346608	-	TSS_031140	1000	535	0	1	I	within gene(s) PMM1400;
1346717	-	TSS_031144	1000	389	0	37	P	91nt upstream of gene PMM1400;
1346985	+	TSS_013062	1000	1898	0	0	P	146nt upstream of gene PMM1401;
1347056	+	TSS_013064	1000	230	0	0	P	75nt upstream of gene PMM1401;
1347808	+	TSS_013066	1000	139	0	0	P	21nt upstream of gene PMM1402;
1348509	+	TSS_013077	1000	123	0	0	O	-
1350449	-	TSS_031165	1000	449	0	3	P	81nt upstream of gene PMM1404;
1351013	-	TSS_031169	1000	1102	0	4	P	26nt upstream of gene PMM1405;
1352055	-	TSS_031177	1000	503	0	0	P	24nt upstream of gene PMM1408;
1352429	-	TSS_031183	1000	1538	0	3	P	18nt upstream of gene PMM1409;
1353625	-	TSS_031197	1000	296	0	7	O	-
1353716	-	TSS_031201	1000	1292	0	1	O	-
1354742	+	TSS_013089	1000	216	0	18	I	within gene(s) PMM1412;
1354808	+	TSS_013103	1000	160	0	27	I	within gene(s) PMM1412;
1354859	+	TSS_013113	1000	207	0	39	I	within gene(s) PMM1412;
1355165	+	TSS_013123	1000	1532	0	3	P	17nt upstream of gene PMM1413;
1355538	-	TSS_031211	1000	111	0	0	O	-
1355578	+	TSS_013126	1000	131	0	6	P	14nt upstream of gene PMM1414;
1355765	+	TSS_013130	1000	196	0	2	IP	within gene(s) PMM1414; 230nt upstream of gene PMM1415;
1356437	-	TSS_031213	1000	301	0	5	Ai	antisense to gene(s) PMM1415;
1356695	+	TSS_013136	1000	141	0	1	I	within gene(s) PMM1415;
1357783	+	TSS_013144	1000	129	0	0	I	within gene(s) PMM1416;
1358056	+	TSS_013149	1000	152	0	0	I	within gene(s) PMM1416;
1358219	+	TSS_013154	1000	183	0	2	I	within gene(s) PMM1416;
1358579	+	TSS_013157	1000	113	0	0	I	within gene(s) PMM1416;
1358652	+	TSS_013162	1000	1004	0	2	I	within gene(s) PMM1416;
1358898	-	TSS_031229	1000	513	0	0	Ai	antisense to gene(s) PMM1416;
1359128	-	TSS_031231	1000	120	0	0	Ai	antisense to gene(s) PMM1416;
1359810	-	TSS_031236	1000	386	0	1	Ai	antisense to gene(s) PMM1416;
1359899	-	TSS_031245	1000	337	0	9	O	-
1360152	+	TSS_013174	1000	112	0	0	O	-
1360185	-	TSS_031250	1000	21127	0	3	O	-
1362875	+	TSS_013182	1000	210	0	0	Ai	antisense to gene(s) PMM1421 PMM1422;
1363264	-	TSS_031266	1000	353	0	14	I	within gene(s) PMM1422;
1363417	-	TSS_031268	1000	319	0	0	P	27nt upstream of gene PMM1422;
1364219	-	TSS_031270	1000	289	0	12	P	69nt upstream of gene PMM1424;
1364352	-	TSS_031278	1000	2664	0	0	P	202nt upstream of gene PMM1424;
1364483	+	TSS_013187	1000	157	0	0	O	-
1365171	+	TSS_013191	1000	116	0	4	P	15nt upstream of gene PMM1425;
1366150	-	TSS_031282	1000	232	0	2	Ai	antisense to gene(s) PMM1425;
1366222	-	TSS_031284	1000	305	0	0	Ai	antisense to gene(s) PMM1425;
1367120	+	TSS_013196	1000	186	0	0	P	6nt upstream of gene PMM1427;

1367587	+	TSS_013200	1000	365	0	2	P	92nt upstream of gene PMM1428;
1367686	+	TSS_013203	1000	123	0	0	I	within gene(s) PMM1428;
1367737	+	TSS_013204	1000	120	0	0	I	within gene(s) PMM1428;
1369350	-	TSS_031289	1000	162	0	2	P	19nt upstream of gene PMM1431;
1369959	+	TSS_013211	1000	218	0	1	I	within gene(s) PMM1432;
1370154	-	TSS_031293	1000	102	0	0	Ai	antisense to gene(s) PMM1432;
1371618	+	TSS_013219	1000	122	0	0	P	59nt upstream of gene PMM1434;
1371875	+	TSS_013228	1000	205	0	25	I	within gene(s) PMM1434;
1372149	-	TSS_031302	1000	1890	0	2	Ai	antisense to gene(s) PMM1434;
1372730	+	TSS_013239	1000	146	0	6	I	within gene(s) PMM1434;
1372986	+	TSS_013248	1000	191	0	5	I	within gene(s) PMM1434;
1373308	+	TSS_013253	1000	1934	0	4	P	17nt upstream of gene PMM1435;
1373532	+	TSS_013258	1000	426	0	1	I	within gene(s) PMM1435;
1373670	+	TSS_013261	1000	223	0	2	Ai	antisense to gene(s) PMM1436;
1373679	+	TSS_013262	1000	1316	0	0	Ai	antisense to gene(s) PMM1436;
1373756	-	TSS_031311	1000	104	0	3	I	within gene(s) PMM1436;
1373844	+	TSS_013264	1000	548	0	13	Ai	antisense to gene(s) PMM1436;
1373858	-	TSS_031323	1000	226	0	33	I	within gene(s) PMM1436;
1373890	-	TSS_031330	1000	192	0	12	I	within gene(s) PMM1436;
1373898	+	TSS_013267	1000	210	0	0	Ai	antisense to gene(s) PMM1436;
1373900	-	TSS_031332	1000	479	0	30	I	within gene(s) PMM1436;
1373925	+	TSS_013268	1000	104	0	0	Ai	antisense to gene(s) PMM1436;
1373945	-	TSS_031345	1000	174	0	3	I	within gene(s) PMM1436;
1373993	-	TSS_031349	1000	121	0	9	I	within gene(s) PMM1436;
1374014	-	TSS_031353	1000	354	0	14	I	within gene(s) PMM1436;
1374056	-	TSS_031363	1000	554	0	45	I	within gene(s) PMM1436;
1374098	-	TSS_031372	1000	113	1.30E-08	0	I	within gene(s) PMM1436;
1374122	-	TSS_031375	1000	221	0	12	I	within gene(s) PMM1436;
1374134	-	TSS_031377	1000	315	0	0	I	within gene(s) PMM1436;
1374185	-	TSS_031390	1000	700	0	39	I	within gene(s) PMM1436;
1374206	-	TSS_031393	1000	193	0	3	I	within gene(s) PMM1436;
1374224	-	TSS_031398	1000	631	0	12	I	within gene(s) PMM1436;
1374229	+	TSS_013272	1000	1106	0	0	Ai	antisense to gene(s) PMM1436;
1374236	+	TSS_013273	1000	127	0	0	Ai	antisense to gene(s) PMM1436;
1374251	-	TSS_031405	1000	537	0	24	I	within gene(s) PMM1436;
1374253	+	TSS_013275	1000	531	0	1	Ai	antisense to gene(s) PMM1436;
1374273	+	TSS_013276	1000	186	0	0	Ai	antisense to gene(s) PMM1436;
1374296	-	TSS_031416	1000	1421	0	87	I	within gene(s) PMM1436;
1374386	-	TSS_031444	1000	388	0	15	I	within gene(s) PMM1436;
1374404	-	TSS_031448	1000	1029	0	18	I	within gene(s) PMM1436;
1374422	-	TSS_031452	1000	211	0	0	I	within gene(s) PMM1436;
1374444	+	TSS_013282	1000	418	0	5	Ai	antisense to gene(s) PMM1436;
1374445	-	TSS_031457	1000	897	0	108	I	within gene(s) PMM1436;
1374468	+	TSS_013284	1000	145	0	0	Ai	antisense to gene(s) PMM1436;
1374560	-	TSS_031494	1000	354	0	24	I	within gene(s) PMM1436;
1374596	-	TSS_031503	1000	270	0	30	I	within gene(s) PMM1436;
1374629	-	TSS_031508	1000	182	0	0	I	within gene(s) PMM1436;
1374653	-	TSS_031514	1000	443	0	39	I	within gene(s) PMM1436;
1374692	-	TSS_031524	1000	637	0	24	I	within gene(s) PMM1436;
1374706	+	TSS_013291	1000	143	0	0	Ai	antisense to gene(s) PMM1436;
1374722	-	TSS_031532	1000	266	0	10	I	within gene(s) PMM1436;
1374752	-	TSS_031536	1000	1255	0	25	I	within gene(s) PMM1436;
1374806	-	TSS_031549	1000	3135	0	33	I	within gene(s) PMM1436;
1374833	-	TSS_031555	1000	218	0	0	I	within gene(s) PMM1436;
1374851	-	TSS_031557	1000	837	0	18	I	within gene(s) PMM1436;
1374887	+	TSS_013292	1000	367	0	0	Ai	antisense to gene(s) PMM1436;
1374890	-	TSS_031568	1000	672	0	57	I	within gene(s) PMM1436;
1374908	+	TSS_013293	1000	103	0	0	Ai	antisense to gene(s) PMM1436;
1374959	-	TSS_031587	1000	308	0	21	I	within gene(s) PMM1436;
1374974	-	TSS_031590	1000	214	0	24	I	within gene(s) PMM1436;
1375013	-	TSS_031597	1000	152	0	0	I	within gene(s) PMM1436;
1375038	+	TSS_013294	1000	495	0	0	Ai	antisense to gene(s) PMM1436;
1375058	-	TSS_031610	1000	455	0	54	I	within gene(s) PMM1436;
1375097	-	TSS_031617	1000	120	0	9	I	within gene(s) PMM1436;
1375139	-	TSS_031620	1000	328	0	24	I	within gene(s) PMM1436;
1375205	-	TSS_031629	1000	514	0	12	I	within gene(s) PMM1436;
1375244	-	TSS_031638	1000	202	0	18	I	within gene(s) PMM1436;
1375257	+	TSS_013297	1000	3397	0	1	Ai	antisense to gene(s) PMM1436;
1375264	-	TSS_031642	1000	134	0	4	I	within gene(s) PMM1436;
1375284	-	TSS_031644	1000	176	0	2	P	10nt upstream of gene PMM1436;
1375334	-	TSS_031646	1000	416	0	0	IP	within gene(s) PMM1437; 60nt upstream of gene PMM1436;
1375343	-	TSS_031648	1000	1026	0	2	IP	within gene(s) PMM1437; 69nt upstream of gene PMM1436;
1375361	-	TSS_031650	1000	143	0	0	IP	within gene(s) PMM1437; 87nt upstream of gene PMM1436;
1375373	-	TSS_031651	1000	423	0	20	IP	within gene(s) PMM1437; 99nt upstream of gene PMM1436;
1375391	+	TSS_013298	1000	195	0	1	Ai	antisense to gene(s) PMM1437;
1375404	+	TSS_013300	1000	161	0	0	Ai	antisense to gene(s) PMM1437;
1375412	-	TSS_031662	1000	531	0	14	IP	within gene(s) PMM1437; 138nt upstream of gene PMM1436;

1375436	-	TSS_031667	1000	297	0	26	IP	within gene(s) PMM1437; 162nt upstream of gene PMM1436;
1375472	-	TSS_031672	1000	268	0	0	IP	within gene(s) PMM1437; 198nt upstream of gene PMM1436;
1375502	-	TSS_031681	1000	560	0	21	IP	within gene(s) PMM1437; 228nt upstream of gene PMM1436;
1375511	-	TSS_031682	1000	137	7.90E-14	0	IP	within gene(s) PMM1437; 237nt upstream of gene PMM1436;
1375520	-	TSS_031683	1000	120	4.50E-09	0	IP	within gene(s) PMM1437; 246nt upstream of gene PMM1436;
1375529	-	TSS_031684	1000	162	0	0	I	within gene(s) PMM1437;
1375544	-	TSS_031686	1000	377	0	7	I	within gene(s) PMM1437;
1375586	-	TSS_031694	1000	729	0	21	I	within gene(s) PMM1437;
1375598	-	TSS_031695	1000	132	1.70E-07	0	I	within gene(s) PMM1437;
1375613	-	TSS_031697	1000	546	0	24	I	within gene(s) PMM1437;
1375640	-	TSS_031702	1000	315	0	0	P	3nt upstream of gene PMM1437;
1375676	-	TSS_031703	1000	422	0	0	P	39nt upstream of gene PMM1437;
1375697	-	TSS_031704	1000	504	0	0	P	60nt upstream of gene PMM1437;
1375706	-	TSS_031707	1000	418221	0	7	P	69nt upstream of gene PMM1437;
1375837	+	TSS_013305	1000	1121	0	1	P	21nt upstream of gene PMM1438;
1376113	+	TSS_013324	1000	183	0	10	I	within gene(s) PMM1438;
1376221	+	TSS_013334	1000	153	0	1	I	within gene(s) PMM1438;
1376281	+	TSS_013342	1000	102	0	0	I	within gene(s) PMM1438;
1376400	-	TSS_031719	1000	167	0	1	Ai	antisense to gene(s) PMM1438;
1376407	+	TSS_013359	1000	257	0	36	I	within gene(s) PMM1438;
1376440	-	TSS_031722	1000	266	0	0	Ai	antisense to gene(s) PMM1438;
1376471	+	TSS_013368	1000	149	0	9	I	within gene(s) PMM1438;
1376500	+	TSS_013372	1000	198	0	6	I	within gene(s) PMM1438;
1376542	+	TSS_013377	1000	289	0	15	I	within gene(s) PMM1438;
1376547	-	TSS_031724	1000	272	0	0	Ai	antisense to gene(s) PMM1438;
1376572	+	TSS_013383	1000	218	0	0	I	within gene(s) PMM1438;
1376665	+	TSS_013388	1000	213	0	39	I	within gene(s) PMM1438;
1376719	+	TSS_013403	1000	139	0	12	I	within gene(s) PMM1438;
1376761	+	TSS_013408	1000	134	0	0	I	within gene(s) PMM1438;
1376776	+	TSS_013410	1000	259	0	1	I	within gene(s) PMM1438;
1376893	+	TSS_013414	1000	142	0	18	I	within gene(s) PMM1438;
1377151	+	TSS_013442	1000	123	0	12	IP	within gene(s) PMM1438; 216nt upstream of gene PMM1439;
1377943	+	TSS_013471	1000	150	0	1	Ai	antisense to gene(s) PMM1440;
1378047	-	TSS_031757	1000	137	0	12	I	within gene(s) PMM1440;
1378081	+	TSS_013475	1000	183	0	0	Ai	antisense to gene(s) PMM1440;
1378110	-	TSS_031767	1000	212	0	9	I	within gene(s) PMM1440;
1378250	-	TSS_031773	1000	381	0	0	I	within gene(s) PMM1440;
1378535	-	TSS_031774	1000	160	0	0	P	17nt upstream of gene PMM1441;
1378674	+	TSS_013479	1000	279	0	2	Ai	antisense to gene(s) PMM1442;
1379084	-	TSS_031785	1000	117	0	0	I	within gene(s) PMM1442;
1379477	+	TSS_013484	1000	7309	0	9	Ai	antisense to gene(s) PMM1442;
1379514	+	TSS_013486	1000	103	0	0	Ai	antisense to gene(s) PMM1442;
1379556	+	TSS_013487	1000	943	0	0	Ai	antisense to gene(s) PMM1442;
1379949	+	TSS_013489	1000	216	0	0	P	27nt upstream of gene PMM1443;
1380019	+	TSS_013493	1000	374	0	6	I	within gene(s) PMM1443;
1380027	+	TSS_013494	1000	378	0	0	I	within gene(s) PMM1443;
1380876	+	TSS_013509	1000	1923	0	2	IP	within gene(s) PMM1443; 80nt upstream of gene PMM1444;
1382749	+	TSS_013522	1000	144	0	0	IP	within gene(s) PMM1445; 31nt upstream of gene PMM1446;
1385792	+	TSS_013531	1000	836	0	3	Ai	antisense to gene(s) PMM1449;
1385823	+	TSS_013533	1000	148	0	0	Ai	antisense to gene(s) PMM1449;
1386056	-	TSS_031809	1000	112	0	0	I	within gene(s) PMM1449;
1386095	+	TSS_013535	1000	164	0	1	Ai	antisense to gene(s) PMM1450;
1386275	-	TSS_031819	1000	117	0	0	IP	within gene(s) PMM1450; 197nt upstream of gene PMM1449;
1386617	-	TSS_031836	1000	139	0	18	I	within gene(s) PMM1450;
1386631	-	TSS_031839	1000	216	0	6	I	within gene(s) PMM1450;
1386653	-	TSS_031845	1000	230	0	19	I	within gene(s) PMM1450;
1386686	-	TSS_031852	1000	111	0	6	I	within gene(s) PMM1450;
1386700	+	TSS_013540	1000	441	0	0	Ai	antisense to gene(s) PMM1450;
1386710	-	TSS_031855	1000	173	0	30	I	within gene(s) PMM1450;
1386887	-	TSS_031877	1000	223	0	19	I	within gene(s) PMM1450;
1386905	-	TSS_031881	1000	105	0	18	I	within gene(s) PMM1450;
1386965	-	TSS_031890	1000	104	0	6	I	within gene(s) PMM1450;
1387001	-	TSS_031893	1000	114	0	9	I	within gene(s) PMM1450;
1387066	+	TSS_013542	1000	177	0	1	Ai	antisense to gene(s) PMM1451;
1387078	+	TSS_013544	1000	133	0	0	Ai	antisense to gene(s) PMM1451;
1387081	-	TSS_031899	1000	115	0	10	IP	within gene(s) PMM1451; 38nt upstream of gene PMM1450;
1387102	-	TSS_031905	1000	176	0	18	IP	within gene(s) PMM1451; 59nt upstream of gene PMM1450;
1387285	-	TSS_031922	1000	111	0	18	IP	within gene(s) PMM1451; 242nt upstream of gene PMM1450;
1387309	-	TSS_031926	1000	164	0	9	I	within gene(s) PMM1451;
1387330	-	TSS_031933	1000	118	0	9	I	within gene(s) PMM1451;
1387384	-	TSS_031939	1000	129	0	24	I	within gene(s) PMM1451;
1387408	-	TSS_031943	1000	153	0	3	I	within gene(s) PMM1451;
1387420	-	TSS_031945	1000	117	0	2	I	within gene(s) PMM1451;
1387543	-	TSS_031961	1000	101	0	9	I	within gene(s) PMM1451;
1387588	-	TSS_031966	1000	183	0	6	I	within gene(s) PMM1451;
1387618	-	TSS_031971	1000	204	0	21	I	within gene(s) PMM1451;
1387660	-	TSS_031980	1000	258	0	6	I	within gene(s) PMM1451;

1387661	+	TSS_013548	1000	166	0	2	Ai	antisense to gene(s) PMM1451;
1387681	-	TSS_031983	1000	136	0	0	I	within gene(s) PMM1451;
1387751	-	TSS_031988	1000	198	0	13	I	within gene(s) PMM1451;
1387783	-	TSS_031996	1000	107	0	12	I	within gene(s) PMM1451;
1387869	-	TSS_032006	1000	299	0	8	I	within gene(s) PMM1451;
1387900	-	TSS_032011	1000	413	0	54	I	within gene(s) PMM1451;
1387990	-	TSS_032024	1000	241	0	3	I	within gene(s) PMM1451;
1388011	-	TSS_032028	1000	353	0	18	I	within gene(s) PMM1451;
1388047	-	TSS_032035	1000	133	0	9	I	within gene(s) PMM1451;
1388119	-	TSS_032040	1000	208	0	0	I	within gene(s) PMM1451;
1388140	-	TSS_032043	1000	416	0	12	I	within gene(s) PMM1451;
1388180	+	TSS_013561	1000	417	0	7	Ai	antisense to gene(s) PMM1451;
1388188	-	TSS_032056	1000	609	0	72	I	within gene(s) PMM1451;
1388242	-	TSS_032068	1000	117	0	0	I	within gene(s) PMM1451;
1388252	-	TSS_032070	1000	230	0	11	I	within gene(s) PMM1451;
1388308	-	TSS_032083	1000	283	0	45	I	within gene(s) PMM1451;
1388326	-	TSS_032086	1000	188	0	12	I	within gene(s) PMM1451;
1388346	+	TSS_013566	1000	135	0	1	Ai	antisense to gene(s) PMM1451;
1388383	-	TSS_032093	1000	182	0	15	I	within gene(s) PMM1451;
1388455	-	TSS_032098	1000	153	0	3	I	within gene(s) PMM1451;
1388530	-	TSS_032106	1000	221	0	9	I	within gene(s) PMM1451;
1388669	-	TSS_032117	1000	4726	0	8	IP	within gene(s) PMM1452; 88nt upstream of gene PMM1451;
1388708	-	TSS_032120	1000	112	0	15	IP	within gene(s) PMM1452; 127nt upstream of gene PMM1451;
1388726	-	TSS_032124	1000	191	0	9	IP	within gene(s) PMM1452; 145nt upstream of gene PMM1451;
1388825	-	TSS_032128	1000	349	0	4	IP	within gene(s) PMM1452; 244nt upstream of gene PMM1451;
1389346	-	TSS_032144	1000	911	0	10	IP	within gene(s) PMM1453; 191nt upstream of gene PMM1452;
1389819	-	TSS_032158	1000	144	0	1	IP	within gene(s) PMM1454; 152nt upstream of gene PMM1453;
1390499	-	TSS_032174	1000	112	0	0	P	60nt upstream of gene PMM1455;
1390725	-	TSS_032177	1000	195	0	1	I	within gene(s) PMM1456;
1390773	-	TSS_032185	1000	117	0	7	I	within gene(s) PMM1456;
1390806	-	TSS_032188	1000	120	0	10	I	within gene(s) PMM1456;
1391446	-	TSS_032213	1000	217	0	0	IP	within gene(s) PMM1457; 115nt upstream of gene PMM1456;
1391614	-	TSS_032221	1000	101	0	2	I	within gene(s) PMM1457;
1391900	-	TSS_032222	1000	301	0	1	P	44nt upstream of gene PMM1457;
1392904	-	TSS_032225	1000	164	0	4	Ai	antisense to gene(s) PMM1458;
1393119	+	TSS_013585	1000	106	0	0	I	within gene(s) PMM1458;
1393186	+	TSS_013586	1000	558	0	0	I	within gene(s) PMM1458;
1393368	+	TSS_013590	1000	1583	0	2	P	69nt upstream of gene PMM1459;
1393733	+	TSS_013596	1000	123	0	2	I	within gene(s) PMM1459;
1395953	+	TSS_013600	1000	1733	0	6	P	14nt upstream of gene PMM1462;
1396223	+	TSS_013611	1000	3844	0	5	P	14nt upstream of gene PMM1463;
1396297	+	TSS_013620	1000	105	0	16	I	within gene(s) PMM1463;
1396318	+	TSS_013625	1000	112	0	12	I	within gene(s) PMM1463;
1397666	+	TSS_013638	1000	3082	0	4	P	16nt upstream of gene PMM1465;
1398489	+	TSS_013681	1000	245	0	45	I	within gene(s) PMM1465;
1398534	+	TSS_013688	1000	116	0	3	I	within gene(s) PMM1465;
1398965	+	TSS_013698	1000	124	0	5	IP	within gene(s) PMM1465; 53nt upstream of gene PMM1466;
1401712	-	TSS_032253	1000	179	0	0	I	within gene(s) PMM1467;
1403187	-	TSS_032263	1000	1397	0	1	P	46nt upstream of gene PMM1467;
1410409	-	TSS_032277	1000	106	0	0	I	within gene(s) PMM1476;
1411270	-	TSS_032306	1000	105	0	3	I	within gene(s) PMM1478;
1411576	-	TSS_032319	1000	11591	0	3	P	17nt upstream of gene PMM1479;
1412048	+	TSS_013727	1000	261	0	0	Ai	antisense to gene(s) PMM1480;
1412609	-	TSS_032325	1000	747	0	5	P	161nt upstream of gene PMM1480;
1413912	+	TSS_013732	1000	703	0	0	O	-
1414077	-	TSS_032340	1000	106	0	1	IP	within gene(s) PMM1483; 184nt upstream of gene PMM1482;
1414293	-	TSS_032345	1000	171	0	7	I	within gene(s) PMM1483;
1414314	-	TSS_032349	1000	105	0	7	I	within gene(s) PMM1483;
1414329	-	TSS_032351	1000	184	0	23	I	within gene(s) PMM1483;
1414389	-	TSS_032362	1000	148	0	18	I	within gene(s) PMM1483;
1414401	-	TSS_032364	1000	158	0	3	I	within gene(s) PMM1483;
1414422	-	TSS_032367	1000	106	0	9	I	within gene(s) PMM1483;
1414437	-	TSS_032369	1000	286	0	6	I	within gene(s) PMM1483;
1414482	-	TSS_032375	1000	197	0	15	I	within gene(s) PMM1483;
1414653	-	TSS_032392	1000	294	0	33	I	within gene(s) PMM1483;
1414716	-	TSS_032407	1000	110	0	21	I	within gene(s) PMM1483;
1414814	-	TSS_032416	1000	115	0	11	I	within gene(s) PMM1483;
1414908	-	TSS_032424	1000	135	0	15	I	within gene(s) PMM1483;
1414936	+	TSS_013746	1000	102	0	0	Ai	antisense to gene(s) PMM1483;
1414968	-	TSS_032435	1000	1018	0	13	I	within gene(s) PMM1483;
1415010	-	TSS_032445	1000	162	0	12	I	within gene(s) PMM1483;
1415040	-	TSS_032447	1000	233	0	0	I	within gene(s) PMM1483;
1415079	-	TSS_032449	1000	180	0	3	I	within gene(s) PMM1483;
1415133	-	TSS_032456	1000	446	0	81	I	within gene(s) PMM1483;
1415214	-	TSS_032474	1000	160	0	36	I	within gene(s) PMM1483;
1415226	+	TSS_013752	1000	122	0	0	Ai	antisense to gene(s) PMM1483;
1415400	-	TSS_032494	1000	548	0	18	I	within gene(s) PMM1483;

1415436	-	TSS_032502	1000	130	0	12	I	within gene(s) PMM1483;
1415466	-	TSS_032507	1000	184	0	18	I	within gene(s) PMM1483;
1415715	+	TSS_013757	1000	225	0	0	Ai	antisense to gene(s) PMM1483;
1415907	+	TSS_013759	1000	291	0	1	Ai	antisense to gene(s) PMM1483;
1415910	-	TSS_032543	1000	189	0	30	I	within gene(s) PMM1483;
1415943	-	TSS_032548	1000	211	0	11	I	within gene(s) PMM1483;
1415981	-	TSS_032554	1000	165	0	8	I	within gene(s) PMM1483;
1416012	-	TSS_032558	1000	471	0	18	I	within gene(s) PMM1483;
1416066	-	TSS_032573	1000	525	0	75	I	within gene(s) PMM1483;
1416114	-	TSS_032585	1000	104	0	0	I	within gene(s) PMM1483;
1416123	-	TSS_032586	1000	103	0	0	I	within gene(s) PMM1483;
1416189	-	TSS_032591	1000	135	0	12	I	within gene(s) PMM1483;
1416227	-	TSS_032595	1000	348	0	2	I	within gene(s) PMM1483;
1416255	-	TSS_032598	1000	198	0	30	I	within gene(s) PMM1483;
1416272	+	TSS_013764	1000	119	0	0	Ai	antisense to gene(s) PMM1483;
1416288	-	TSS_032607	1000	146	0	0	I	within gene(s) PMM1483;
1416333	-	TSS_032614	1000	128	0	9	I	within gene(s) PMM1483;
1416401	+	TSS_013767	1000	296	0	0	Ai	antisense to gene(s) PMM1483;
1416423	-	TSS_032623	1000	133	0	15	I	within gene(s) PMM1483;
1416430	+	TSS_013769	1000	546	0	6	Ai	antisense to gene(s) PMM1483;
1416483	-	TSS_032629	1000	117	0	15	I	within gene(s) PMM1483;
1416518	+	TSS_013772	1000	153	0	0	Ai	antisense to gene(s) PMM1483;
1416528	-	TSS_032638	1000	142	0	15	I	within gene(s) PMM1483;
1416963	-	TSS_032688	1000	117	0	6	I	within gene(s) PMM1483;
1416999	-	TSS_032691	1000	2329	0	16	I	within gene(s) PMM1483;
1417029	-	TSS_032699	1000	120	0	6	I	within gene(s) PMM1483;
1417062	-	TSS_032707	1000	305	0	24	I	within gene(s) PMM1483;
1417098	-	TSS_032714	1000	292	0	30	I	within gene(s) PMM1483;
1417128	-	TSS_032719	1000	303	0	3	I	within gene(s) PMM1483;
1417146	-	TSS_032723	1000	104	0	6	I	within gene(s) PMM1483;
1417253	+	TSS_013782	1000	107	0	0	Ai	antisense to gene(s) PMM1483;
1417302	-	TSS_032739	1000	139	0	12	I	within gene(s) PMM1483;
1417319	+	TSS_013785	1000	163	0	7	Ai	antisense to gene(s) PMM1483;
1417332	+	TSS_013787	1000	122	0	0	Ai	antisense to gene(s) PMM1483;
1417434	-	TSS_032763	1000	159	0	27	I	within gene(s) PMM1483;
1417470	-	TSS_032769	1000	103	0	3	I	within gene(s) PMM1483;
1417512	-	TSS_032778	1000	162	0	27	I	within gene(s) PMM1483;
1417554	-	TSS_032786	1000	107	0	18	I	within gene(s) PMM1483;
1417602	-	TSS_032794	1000	101	0	12	I	within gene(s) PMM1483;
1417887	-	TSS_032813	1000	449	0	9	I	within gene(s) PMM1483;
1418150	+	TSS_013792	1000	1222	0	1	Ai	antisense to gene(s) PMM1484;
1418301	+	TSS_013799	1000	125	0	0	Ai	antisense to gene(s) PMM1484;
1418469	-	TSS_032859	1000	210	0	18	I	within gene(s) PMM1484;
1418489	+	TSS_013800	1000	1287	0	4	Ai	antisense to gene(s) PMM1484;
1418507	+	TSS_013802	1000	2999	0	6	Ai	antisense to gene(s) PMM1484;
1418673	-	TSS_032875	1000	132	0	20	I	within gene(s) PMM1484;
1418723	-	TSS_032888	1000	302	0	23	I	within gene(s) PMM1484;
1418742	-	TSS_032892	1000	177	0	15	I	within gene(s) PMM1484;
1418763	-	TSS_032896	1000	165	0	6	I	within gene(s) PMM1484;
1418780	+	TSS_013807	1000	524	0	3	Ai	antisense to gene(s) PMM1484;
1418831	+	TSS_013810	1000	153	0	1	Ai	antisense to gene(s) PMM1484;
1418919	-	TSS_032912	1000	125	0	21	I	within gene(s) PMM1484;
1418948	-	TSS_032915	1000	308	0	1	I	within gene(s) PMM1484;
1418976	-	TSS_032919	1000	123	0	3	I	within gene(s) PMM1484;
1418994	-	TSS_032923	1000	254	0	21	I	within gene(s) PMM1484;
1419034	+	TSS_013816	1000	229	0	9	Ai	antisense to gene(s) PMM1484;
1419062	+	TSS_013818	1000	108	0	0	Ai	antisense to gene(s) PMM1484;
1419177	-	TSS_032937	1000	238	0	12	I	within gene(s) PMM1484;
1419207	-	TSS_032943	1000	139	0	15	I	within gene(s) PMM1484;
1419214	+	TSS_013820	1000	465	0	1	Ai	antisense to gene(s) PMM1484;
1419222	-	TSS_032946	1000	215	0	0	I	within gene(s) PMM1484;
1419267	-	TSS_032954	1000	731	0	9	I	within gene(s) PMM1484;
1419422	-	TSS_032968	1000	105	0	0	I	within gene(s) PMM1484;
1419537	-	TSS_032977	1000	112	0	9	I	within gene(s) PMM1484;
1419648	+	TSS_013825	1000	573	0	0	Ai	antisense to gene(s) PMM1484;
1419651	-	TSS_032983	1000	385	0	7	I	within gene(s) PMM1484;
1419666	-	TSS_032988	1000	291	0	25	I	within gene(s) PMM1484;
1419730	+	TSS_013827	1000	183	0	0	Ai	antisense to gene(s) PMM1484;
1419792	-	TSS_033007	1000	172	0	12	I	within gene(s) PMM1484;
1419819	-	TSS_033012	1000	191	0	9	I	within gene(s) PMM1484;
1419883	+	TSS_013829	1000	292	0	0	Ai	antisense to gene(s) PMM1484;
1419954	-	TSS_033023	1000	133	0	0	I	within gene(s) PMM1484;
1420037	-	TSS_033026	1000	328	0	0	IP	within gene(s) PMM1485; 56nt upstream of gene PMM1484;
1420171	-	TSS_033042	1000	272	0	10	IP	within gene(s) PMM1485; 190nt upstream of gene PMM1484;
1420202	-	TSS_033048	1000	130	0	3	IP	within gene(s) PMM1485; 221nt upstream of gene PMM1484;
1420412	-	TSS_033058	1000	210	0	7	I	within gene(s) PMM1485;
1420518	+	TSS_013831	1000	450	0	2	Ai	antisense to gene(s) PMM1485;

1420706	-	TSS_033082	1000	540	0	0	I	within gene(s) PMM1485;
1420720	+	TSS_013835	1000	215	0	4	Ai	antisense to gene(s) PMM1485;
1420736	-	TSS_033084	1000	174	0	3	I	within gene(s) PMM1485;
1420978	-	TSS_033107	1000	204	0	0	I	within gene(s) PMM1485;
1421015	+	TSS_013842	1000	104	0	3	Ai	antisense to gene(s) PMM1485;
1421293	-	TSS_033127	1000	116	0	1	I	within gene(s) PMM1485;
1421475	+	TSS_013845	1000	134	0	1	Ai	antisense to gene(s) PMM1485;
1421567	+	TSS_013849	1000	137	0	0	Ai	antisense to gene(s) PMM1485;
1421612	-	TSS_033143	1000	111	0	0	I	within gene(s) PMM1485;
1421688	+	TSS_013851	1000	207	0	1	Ai	antisense to gene(s) PMM1485;
1421690	-	TSS_033151	1000	137	0	0	I	within gene(s) PMM1485;
1422142	+	TSS_013856	1000	482	0	0	Ai	antisense to gene(s) PMM1485;
1422371	-	TSS_033185	1000	122	0	3	I	within gene(s) PMM1485;
1422752	-	TSS_033201	1000	195	0	15	I	within gene(s) PMM1485;
1422856	-	TSS_033206	1000	826	0	0	I	within gene(s) PMM1485;
1423046	-	TSS_033213	1000	285	0	0	I	within gene(s) PMM1485;
1423961	+	TSS_013868	1000	466	0	1	Ai	antisense to gene(s) PMM1486;
1424666	-	TSS_033229	1000	389	0	6	P	20nt upstream of gene PMM1487;
1426785	-	TSS_033254	1000	838	0	8	P	38nt upstream of gene PMM1489;
1427278	+	TSS_013888	1000	253	0	2	Ai	antisense to gene(s) PMM1490;
1428507	+	TSS_013893	1000	561	0	0	IP	within gene(s) PMM1491; 98nt upstream of gene PMM1492;
1428755	+	TSS_013900	1000	113	0	4	I	within gene(s) PMM1492;
1428986	+	TSS_013908	1000	184	0	0	I	within gene(s) PMM1492;
1429041	-	TSS_033281	1000	151	0	0	Ai	antisense to gene(s) PMM1492;
1429376	+	TSS_013930	1000	140	0	3	I	within gene(s) PMM1492;
1429529	+	TSS_013934	1000	115	0	8	I	within gene(s) PMM1492;
1429590	+	TSS_013942	1000	336	0	12	I	within gene(s) PMM1492;
1429682	+	TSS_013950	1000	229	0	12	I	within gene(s) PMM1492;
1429712	+	TSS_013955	1000	225	0	7	I	within gene(s) PMM1492;
1429727	+	TSS_013958	1000	452	0	4	I	within gene(s) PMM1492;
1429742	+	TSS_013962	1000	244	0	18	I	within gene(s) PMM1492;
1429871	+	TSS_013978	1000	101	0	3	IP	within gene(s) PMM1492; 134nt upstream of gene PMM1493;
1431083	+	TSS_013991	1000	146	0	0	I	within gene(s) PMM1494;
1431142	+	TSS_013995	1000	658	0	7	I	within gene(s) PMM1494;
1431241	+	TSS_014004	1000	689	0	15	I	within gene(s) PMM1494;
1431340	+	TSS_014012	1000	127	0	6	I	within gene(s) PMM1494;
1431352	+	TSS_014014	1000	129	0	1	I	within gene(s) PMM1494;
1431379	+	TSS_014016	1000	140	0	0	I	within gene(s) PMM1494;
1431397	+	TSS_014019	1000	115	0	6	I	within gene(s) PMM1494;
1431469	+	TSS_014025	1000	364	0	6	I	within gene(s) PMM1494;
1431481	+	TSS_014028	1000	323	0	3	I	within gene(s) PMM1494;
1431490	+	TSS_014029	1000	109	0	0	I	within gene(s) PMM1494;
1431508	+	TSS_014032	1000	169	0	3	I	within gene(s) PMM1494;
1431529	+	TSS_014035	1000	267	0	12	I	within gene(s) PMM1494;
1431553	+	TSS_014037	1000	125	0	0	I	within gene(s) PMM1494;
1431808	+	TSS_014047	1000	113	0	6	I	within gene(s) PMM1494;
1431838	+	TSS_014048	1000	172	0	30	I	within gene(s) PMM1494;
1431913	+	TSS_014062	1000	145	0	9	I	within gene(s) PMM1494;
1432697	-	TSS_033307	1000	104	0	0	Ai	antisense to gene(s) PMM1494;
1432963	+	TSS_014101	1000	215	0	13	I	within gene(s) PMM1494;
1432991	+	TSS_014107	1000	555	0	6	I	within gene(s) PMM1494;
1433023	+	TSS_014111	1000	834	0	7	I	within gene(s) PMM1494;
1433164	+	TSS_014116	1000	120	0	6	I	within gene(s) PMM1494;
1433296	+	TSS_014124	1000	501	0	1	I	within gene(s) PMM1494;
1435445	+	TSS_014154	1000	228	0	0	I	within gene(s) PMM1496;
1436999	+	TSS_014163	1000	1232	0	1	P	24nt upstream of gene PMM1498;
1437356	-	TSS_033320	1000	646	0	1	Ai	antisense to gene(s) PMM1498;
1437515	+	TSS_014186	1000	108	0	21	I	within gene(s) PMM1498;
1438103	+	TSS_014213	1000	148	0	7	I	within gene(s) PMM1498;
1439023	-	TSS_033332	1000	784	0	0	I	within gene(s) PMM1499;
1439138	+	TSS_014227	1000	146	0	0	Ai	antisense to gene(s) PMM1500;
1439379	-	TSS_033353	1000	161	0	4	I	within gene(s) PMM1500;
1439399	+	TSS_014229	1000	184	0	0	Ai	antisense to gene(s) PMM1500;
1439422	-	TSS_033357	1000	263	0	0	I	within gene(s) PMM1500;
1439578	-	TSS_033362	1000	172	0	1	I	within gene(s) PMM1500;
1440282	-	TSS_033398	1000	1658	0	2	P	23nt upstream of gene PMM1500;
1440561	+	TSS_014237	1000	246	0	0	I	within gene(s) PMM1501;
1443305	+	TSS_014248	1000	1785	0	2	PAi	201nt upstream of gene PMM1504; antisense to gene(s) PMM1503;
1443593	-	TSS_033410	1000	724	0	0	PAi	140nt upstream of gene PMM1503; antisense to gene(s) PMM1504;
1444017	-	TSS_033414	1000	125	0	2	Ai	antisense to gene(s) PMM1504;
1446052	+	TSS_014256	1000	164	0	0	Ai	antisense to gene(s) PMM1507;
1446314	-	TSS_033439	1000	102	0	0	I	within gene(s) PMM1507;
1446371	-	TSS_033441	1000	133	0	1	P	51nt upstream of gene PMM1507;
1446380	-	TSS_033444	1000	3568	0	8	P	60nt upstream of gene PMM1507;
1446507	+	TSS_014263	1000	125	0	2	Ai	antisense to gene(s) PMM1508;
1446537	-	TSS_033457	1000	285	0	6	IP	within gene(s) PMM1508; 217nt upstream of gene PMM1507;
1446580	+	TSS_014267	1000	197	0	0	Ai	antisense to gene(s) PMM1508;

1446684 -	TSS_033468	1000	175	0	12	I	within gene(s) PMM1508;
1446705 -	TSS_033472	1000	529	0	37	I	within gene(s) PMM1508;
1446753 -	TSS_033484	1000	339	0	0	I	within gene(s) PMM1508;
1446762 -	TSS_033485	1000	349	0	30	I	within gene(s) PMM1508;
1446807 -	TSS_033497	1000	162	0	6	I	within gene(s) PMM1508;
1446816 -	TSS_033498	1000	206	0	0	I	within gene(s) PMM1508;
1446820 +	TSS_014273	1000	477	0	17	Ai	antisense to gene(s) PMM1508;
1446831 -	TSS_033499	1000	148	0	12	I	within gene(s) PMM1508;
1446855 -	TSS_033502	1000	234	0	6	I	within gene(s) PMM1508;
1446870 -	TSS_033504	1000	134	0	0	I	within gene(s) PMM1508;
1446879 -	TSS_033505	1000	161	0	0	I	within gene(s) PMM1508;
1446894 -	TSS_033506	1000	230	0	0	I	within gene(s) PMM1508;
1446903 -	TSS_033507	1000	198	0	9	I	within gene(s) PMM1508;
1446939 -	TSS_033512	1000	158	0	30	I	within gene(s) PMM1508;
1447023 -	TSS_033532	1000	802	0	42	I	within gene(s) PMM1508;
1447047 +	TSS_014277	1000	108	0	0	Ai	antisense to gene(s) PMM1508;
1447050 -	TSS_033538	1000	510	0	9	I	within gene(s) PMM1508;
1447058 +	TSS_014279	1000	1766	0	1	Ai	antisense to gene(s) PMM1508;
1447059 -	TSS_033539	1000	180	0	0	I	within gene(s) PMM1508;
1447079 +	TSS_014280	1000	327	0	0	Ai	antisense to gene(s) PMM1508;
1447086 -	TSS_033540	1000	1538	0	0	I	within gene(s) PMM1508;
1447098 -	TSS_033541	1000	1046	0	3	I	within gene(s) PMM1508;
1447116 -	TSS_033545	1000	163	0	7	I	within gene(s) PMM1508;
1447167 -	TSS_033559	1000	500	0	63	I	within gene(s) PMM1508;
1447197 -	TSS_033567	1000	145	0	6	I	within gene(s) PMM1508;
1447224 +	TSS_014281	1000	243	0	0	Ai	antisense to gene(s) PMM1508;
1447254 -	TSS_033577	1000	104	0	54	I	within gene(s) PMM1508;
1447287 -	TSS_033583	1000	101	0	0	I	within gene(s) PMM1508;
1447398 -	TSS_033592	1000	117	0	18	I	within gene(s) PMM1508;
1447433 -	TSS_033595	1000	300	0	0	I	within gene(s) PMM1508;
1447455 -	TSS_033596	1000	129	0	0	I	within gene(s) PMM1508;
1447488 -	TSS_033600	1000	103	0	3	I	within gene(s) PMM1508;
1447500 -	TSS_033602	1000	136	0	1	I	within gene(s) PMM1508;
1447577 -	TSS_033611	1000	196	0	14	I	within gene(s) PMM1508;
1447608 -	TSS_033616	1000	144	0	1	I	within gene(s) PMM1508;
1447617 -	TSS_033617	1000	144	0	3	I	within gene(s) PMM1508;
1447634 +	TSS_014289	1000	819	0	1	O	-
1447711 -	TSS_033619	1000	345	0	0	IP	within gene(s) PMM1509; 82nt upstream of gene PMM1508;
1447723 -	TSS_033620	1000	135	0	0	IP	within gene(s) PMM1509; 94nt upstream of gene PMM1508;
1447735 -	TSS_033622	1000	378	0	11	IP	within gene(s) PMM1509; 106nt upstream of gene PMM1508;
1447786 -	TSS_033626	1000	304	0	3	IP	within gene(s) PMM1509; 157nt upstream of gene PMM1508;
1447798 -	TSS_033628	1000	143	0	3	IP	within gene(s) PMM1509; 169nt upstream of gene PMM1508;
1447813 -	TSS_033629	1000	484	0	0	IP	within gene(s) PMM1509; 184nt upstream of gene PMM1508;
1447831 -	TSS_033630	1000	522	0	0	IP	within gene(s) PMM1509; 202nt upstream of gene PMM1508;
1447864 -	TSS_033633	1000	597	0	10	IP	within gene(s) PMM1509; 235nt upstream of gene PMM1508;
1447894 -	TSS_033636	1000	223	0	5	I	within gene(s) PMM1509;
1448059 -	TSS_033640	1000	154	0	12	I	within gene(s) PMM1509;
1448107 -	TSS_033642	1000	122	0	0	I	within gene(s) PMM1509;
1448269 -	TSS_033654	1000	106	0	12	I	within gene(s) PMM1509;
1448311 -	TSS_033659	1000	170	0	6	I	within gene(s) PMM1509;
1448332 -	TSS_033660	1000	209	0	12	I	within gene(s) PMM1509;
1448377 -	TSS_033667	1000	147	0	15	I	within gene(s) PMM1509;
1448434 -	TSS_033676	1000	304	0	24	I	within gene(s) PMM1509;
1448485 -	TSS_033684	1000	1442	0	51	I	within gene(s) PMM1509;
1448583 -	TSS_033704	1000	122	0	0	I	within gene(s) PMM1509;
1448767 -	TSS_033723	1000	147	0	12	I	within gene(s) PMM1509;
1448800 -	TSS_033730	1000	113	0	30	I	within gene(s) PMM1509;
1448818 -	TSS_033733	1000	244	0	30	I	within gene(s) PMM1509;
1448863 -	TSS_033744	1000	218	0	0	I	within gene(s) PMM1509;
1449152 +	TSS_014303	1000	134	0	1	Ai	antisense to gene(s) PMM1509;
1449943 +	TSS_014311	1000	150	0	0	Ai	antisense to gene(s) PMM1510;
1450004 +	TSS_014312	1000	185	0	4	Ai	antisense to gene(s) PMM1510;
1450080 -	TSS_033814	1000	194	0	24	I	within gene(s) PMM1510;
1450188 -	TSS_033836	1000	406	0	15	I	within gene(s) PMM1510;
1450209 -	TSS_033840	1000	145	0	6	I	within gene(s) PMM1510;
1450230 -	TSS_033843	1000	107	0	0	I	within gene(s) PMM1510;
1450367 -	TSS_033850	1000	123	0	0	IP	within gene(s) PMM1511; 44nt upstream of gene PMM1510;
1450376 -	TSS_033851	1000	141	0	0	IP	within gene(s) PMM1511; 53nt upstream of gene PMM1510;
1450406 -	TSS_033852	1000	204	0	12	IP	within gene(s) PMM1511; 83nt upstream of gene PMM1510;
1450505 -	TSS_033860	1000	116	0	21	IP	within gene(s) PMM1511; 182nt upstream of gene PMM1510;
1450507 +	TSS_014316	1000	115	0	0	Ai	antisense to gene(s) PMM1511;
1450571 -	TSS_033868	1000	144	0	8	IP	within gene(s) PMM1511; 248nt upstream of gene PMM1510;
1450700 -	TSS_033873	1000	108	0	0	I	within gene(s) PMM1511;
1450891 +	TSS_014318	1000	166	0	4	P	31nt upstream of gene PMM1512;
1450919 +	TSS_014320	1000	140	0	0	P	3nt upstream of gene PMM1512;
1451660 +	TSS_014325	1000	140	0	0	I	within gene(s) PMM1512;
1452589 -	TSS_033879	1000	125	0	1	Ai	antisense to gene(s) PMM1512;

1454166	+	TSS_014370	1000	191	0	2	I	within gene(s) PMM1512;
1454321	+	TSS_014377	1000	172	0	0	I	within gene(s) PMM1512;
1454543	+	TSS_014384	1000	343	0	3	I	within gene(s) PMM1512;
1454745	+	TSS_014386	1000	115	0	1	I	within gene(s) PMM1512;
1456713	-	TSS_033900	1000	3678	0	4	P	20nt upstream of gene PMM1514;
1457923	+	TSS_014405	1000	104	0	0	Ai	antisense to gene(s) PMM1517;
1459395	-	TSS_033910	1000	146	0	0	Ai	antisense to gene(s) PMM1518;
1459609	+	TSS_014409	1000	147	0	0	Ai	antisense to gene(s) PMM1519;
1459813	+	TSS_014412	1000	127	0	0	Ai	antisense to gene(s) PMM1519;
1459836	-	TSS_033923	1000	241	0	10	I	within gene(s) PMM1519;
1459857	-	TSS_033927	1000	118	0	10	I	within gene(s) PMM1519;
1459955	-	TSS_033945	1000	107	0	9	I	within gene(s) PMM1519;
1460021	-	TSS_033951	1000	193	0	1	I	within gene(s) PMM1519;
1460127	-	TSS_033959	1000	102	0	0	I	within gene(s) PMM1519;
1462056	+	TSS_014418	1000	400	0	0	Ai	antisense to gene(s) PMM1523;
1462103	-	TSS_033972	1000	263	0	19	I	within gene(s) PMM1523;
1462135	-	TSS_033978	1000	302	0	15	I	within gene(s) PMM1523;
1462153	-	TSS_033982	1000	772	0	24	I	within gene(s) PMM1523;
1462225	-	TSS_034005	1000	4923	0	44	I	within gene(s) PMM1523;
1462260	-	TSS_034014	1000	1285	0	6	I	within gene(s) PMM1523;
1462266	+	TSS_014424	1000	240	0	0	Ai	antisense to gene(s) PMM1523;
1462291	-	TSS_034024	1000	4931	0	33	I	within gene(s) PMM1523;
1462312	-	TSS_034030	1000	991	0	36	I	within gene(s) PMM1523;
1462366	-	TSS_034039	1000	503	0	3	I	within gene(s) PMM1523;
1462387	-	TSS_034041	1000	394	0	0	I	within gene(s) PMM1523;
1462416	-	TSS_034044	1000	6956	0	57	I	within gene(s) PMM1523;
1462477	-	TSS_034062	1000	348	0	0	I	within gene(s) PMM1523;
1462519	-	TSS_034070	1000	922	0	30	I	within gene(s) PMM1523;
1462557	-	TSS_034075	1000	578	0	12	I	within gene(s) PMM1523;
1462573	-	TSS_034079	1000	1001	0	12	I	within gene(s) PMM1523;
1462606	-	TSS_034084	1000	358	0	9	I	within gene(s) PMM1523;
1462630	-	TSS_034085	1000	238	0	0	I	within gene(s) PMM1523;
1462639	-	TSS_034086	1000	526	0	2	I	within gene(s) PMM1523;
1462651	-	TSS_034088	1000	971	0	33	I	within gene(s) PMM1523;
1462696	-	TSS_034103	1000	467	0	6	I	within gene(s) PMM1523;
1462714	-	TSS_034106	1000	898	0	6	I	within gene(s) PMM1523;
1462771	-	TSS_034122	1000	2502	0	69	I	within gene(s) PMM1523;
1462817	+	TSS_014431	1000	134	0	0	Ai	antisense to gene(s) PMM1523;
1462819	-	TSS_034131	1000	478	0	0	I	within gene(s) PMM1523;
1462831	-	TSS_034132	1000	298	0	0	I	within gene(s) PMM1523;
1462833	+	TSS_014433	1000	175	0	1	Ai	antisense to gene(s) PMM1523;
1462846	-	TSS_034136	1000	4960	0	6	I	within gene(s) PMM1523;
1462857	+	TSS_014434	1000	375	0	1	Ai	antisense to gene(s) PMM1523;
1462858	-	TSS_034138	1000	1373	0	31	I	within gene(s) PMM1523;
1462930	-	TSS_034155	1000	493	0	39	I	within gene(s) PMM1523;
1462948	-	TSS_034159	1000	1377	0	18	I	within gene(s) PMM1523;
1462981	-	TSS_034169	1000	1197	0	28	I	within gene(s) PMM1523;
1463010	-	TSS_034177	1000	292	0	7	I	within gene(s) PMM1523;
1463026	-	TSS_034180	1000	229	0	0	I	within gene(s) PMM1523;
1463062	-	TSS_034182	1000	682	0	12	I	within gene(s) PMM1523;
1463103	-	TSS_034191	1000	2146	0	66	I	within gene(s) PMM1523;
1463197	-	TSS_034213	1000	1074	0	54	I	within gene(s) PMM1523;
1463257	-	TSS_034227	1000	445	0	34	I	within gene(s) PMM1523;
1463347	-	TSS_034235	1000	109	0	0	I	within gene(s) PMM1523;
1463356	-	TSS_034237	1000	155	0	1	I	within gene(s) PMM1523;
1463392	-	TSS_034239	1000	110	0	0	I	within gene(s) PMM1523;
1463410	-	TSS_034241	1000	438	0	3	I	within gene(s) PMM1523;
1463421	+	TSS_014441	1000	102	0	0	Ai	antisense to gene(s) PMM1523;
1463446	-	TSS_034250	1000	321	0	33	I	within gene(s) PMM1523;
1463467	-	TSS_034255	1000	314	0	21	I	within gene(s) PMM1523;
1463536	-	TSS_034270	1000	698	0	72	I	within gene(s) PMM1523;
1463537	+	TSS_014442	1000	106	0	1	Ai	antisense to gene(s) PMM1523;
1463593	-	TSS_034285	1000	319	0	30	I	within gene(s) PMM1523;
1463632	-	TSS_034296	1000	344	0	0	I	within gene(s) PMM1523;
1463659	-	TSS_034301	1000	266	0	17	I	within gene(s) PMM1523;
1463677	-	TSS_034304	1000	600	0	15	I	within gene(s) PMM1523;
1463698	-	TSS_034308	1000	266	0	9	I	within gene(s) PMM1523;
1463722	-	TSS_034312	1000	215	0	9	I	within gene(s) PMM1523;
1463734	-	TSS_034314	1000	164	0	3	I	within gene(s) PMM1523;
1463744	+	TSS_014444	1000	150	0	0	Ai	antisense to gene(s) PMM1523;
1463746	-	TSS_034317	1000	632	0	10	I	within gene(s) PMM1523;
1463763	-	TSS_034322	1000	175	0	0	I	within gene(s) PMM1523;
1463779	-	TSS_034323	1000	177	0	2	I	within gene(s) PMM1523;
1463812	-	TSS_034325	1000	237	0	9	I	within gene(s) PMM1523;
1463829	+	TSS_014448	1000	442	0	1	Ai	antisense to gene(s) PMM1523;
1463857	-	TSS_034331	1000	652	0	6	I	within gene(s) PMM1523;
1463878	-	TSS_034336	1000	491	0	18	I	within gene(s) PMM1523;

1463899	-	TSS_034342	1000	1011	0	7	I	within gene(s) PMM1523;
1463923	-	TSS_034345	1000	551	0	9	I	within gene(s) PMM1523;
1463951	+	TSS_014450	1000	152	0	1	Ai	antisense to gene(s) PMM1523;
1463962	-	TSS_034353	1000	447	0	36	I	within gene(s) PMM1523;
1463989	-	TSS_034356	1000	140	3.30E-06	0	I	within gene(s) PMM1523;
1464007	-	TSS_034357	1000	176	0	0	I	within gene(s) PMM1523;
1464022	-	TSS_034358	1000	143	4.70E-06	0	I	within gene(s) PMM1523;
1464049	-	TSS_034360	1000	1365	0	9	I	within gene(s) PMM1523;
1464106	-	TSS_034368	1000	2549	0	4	I	within gene(s) PMM1523;
1464129	-	TSS_034369	1000	540	0	1	I	within gene(s) PMM1523;
1464145	-	TSS_034371	1000	375	0	1	P	12nt upstream of gene PMM1523;
1464180	-	TSS_034373	1000	687	0	0	IP	within gene(s) PMM1524; 47nt upstream of gene PMM1523;
1464188	-	TSS_034374	1000	539	0	0	IP	within gene(s) PMM1524; 55nt upstream of gene PMM1523;
1464199	-	TSS_034375	1000	501	0	0	IP	within gene(s) PMM1524; 66nt upstream of gene PMM1523;
1464233	-	TSS_034376	1000	1931	0	0	IP	within gene(s) PMM1524; 100nt upstream of gene PMM1523;
1464257	-	TSS_034378	1000	1052	0	6	IP	within gene(s) PMM1524; 124nt upstream of gene PMM1523;
1464278	-	TSS_034385	1000	5877	0	11	IP	within gene(s) PMM1524; 145nt upstream of gene PMM1523;
1464296	-	TSS_034390	1000	1067	0	9	IP	within gene(s) PMM1524; 163nt upstream of gene PMM1523;
1464314	-	TSS_034393	1000	2406	0	12	IP	within gene(s) PMM1524; 181nt upstream of gene PMM1523;
1464338	-	TSS_034401	1000	1278	0	30	IP	within gene(s) PMM1524; 205nt upstream of gene PMM1523;
1464383	-	TSS_034409	1000	424	0	0	IP	within gene(s) PMM1524; 250nt upstream of gene PMM1523;
1464401	-	TSS_034413	1000	519	0	9	I	within gene(s) PMM1524;
1464424	-	TSS_034414	1000	365	0	1	I	within gene(s) PMM1524;
1464434	-	TSS_034416	1000	540	0	0	I	within gene(s) PMM1524;
1464441	-	TSS_034417	1000	233	3.50E-06	0	I	within gene(s) PMM1524;
1464464	-	TSS_034424	1000	1177	0	14	I	within gene(s) PMM1524;
1464474	-	TSS_034427	1000	344	0	1	I	within gene(s) PMM1524;
1464485	-	TSS_034428	1000	371	0	3	I	within gene(s) PMM1524;
1464512	-	TSS_034434	1000	2332	0	18	I	within gene(s) PMM1524;
1464527	-	TSS_034439	1000	936	0	9	I	within gene(s) PMM1524;
1464545	-	TSS_034442	1000	282	0	0	I	within gene(s) PMM1524;
1464569	+	TSS_014462	1000	110	0	1	Ai	antisense to gene(s) PMM1524;
1464575	-	TSS_034450	1000	3052	0	27	I	within gene(s) PMM1524;
1464596	-	TSS_034453	1000	397	0	0	I	within gene(s) PMM1524;
1464612	+	TSS_014463	1000	182	0	0	Ai	antisense to gene(s) PMM1524;
1464632	-	TSS_034454	1000	301	1.30E-07	0	I	within gene(s) PMM1524;
1464704	-	TSS_034479	1000	3027	0	132	I	within gene(s) PMM1524;
1464785	-	TSS_034506	1000	274	0	0	I	within gene(s) PMM1524;
1464806	-	TSS_034507	1000	755	0	0	I	within gene(s) PMM1524;
1464824	-	TSS_034508	1000	246	0	0	I	within gene(s) PMM1524;
1464839	-	TSS_034510	1000	1135	0	12	I	within gene(s) PMM1524;
1464869	-	TSS_034513	1000	226	0	0	I	within gene(s) PMM1524;
1464876	+	TSS_014465	1000	392	0	4	Ai	antisense to gene(s) PMM1524;
1464887	+	TSS_014468	1000	442	0	0	Ai	antisense to gene(s) PMM1524;
1464887	-	TSS_034514	1000	178	2.10E-07	9	I	within gene(s) PMM1524;
1464905	-	TSS_034517	1000	242	0	6	I	within gene(s) PMM1524;
1464927	-	TSS_034522	1000	1218	0	18	I	within gene(s) PMM1524;
1464947	-	TSS_034530	1000	346	0	3	I	within gene(s) PMM1524;
1464959	+	TSS_014469	1000	845	0	0	Ai	antisense to gene(s) PMM1524;
1464968	-	TSS_034535	1000	1905	0	21	I	within gene(s) PMM1524;
1465007	-	TSS_034542	1000	384	0	6	I	within gene(s) PMM1524;
1465017	+	TSS_014470	1000	124	0	0	Ai	antisense to gene(s) PMM1524;
1465031	-	TSS_034543	1000	332	0	6	I	within gene(s) PMM1524;
1465060	-	TSS_034548	1000	180	0	5	I	within gene(s) PMM1524;
1465069	-	TSS_034549	1000	687	0	25	I	within gene(s) PMM1524;
1465112	-	TSS_034566	1000	624	0	12	I	within gene(s) PMM1524;
1465113	+	TSS_014472	1000	794	0	8	Ai	antisense to gene(s) PMM1524;
1465127	+	TSS_014476	1000	215	0	0	Ai	antisense to gene(s) PMM1524;
1465139	-	TSS_034571	1000	220	0	15	I	within gene(s) PMM1524;
1465140	+	TSS_014477	1000	107	0	0	Ai	antisense to gene(s) PMM1524;
1465157	-	TSS_034574	1000	534	0	15	I	within gene(s) PMM1524;
1465175	-	TSS_034577	1000	129	4.60E-09	0	I	within gene(s) PMM1524;
1465185	+	TSS_014482	1000	204	0	9	Ai	antisense to gene(s) PMM1524;
1465196	-	TSS_034581	1000	460	0	15	I	within gene(s) PMM1524;
1465197	+	TSS_014483	1000	249	0	0	Ai	antisense to gene(s) PMM1524;
1465298	-	TSS_034591	1000	609	0	36	I	within gene(s) PMM1524;
1465322	-	TSS_034596	1000	214	0	6	I	within gene(s) PMM1524;
1465361	-	TSS_034606	1000	1088	0	64	I	within gene(s) PMM1524;
1465412	-	TSS_034622	1000	494	0	3	I	within gene(s) PMM1524;
1465442	-	TSS_034630	1000	811	0	15	I	within gene(s) PMM1524;
1465453	-	TSS_034631	1000	321	0	0	I	within gene(s) PMM1524;
1465475	-	TSS_034632	1000	778	0	3	I	within gene(s) PMM1524;
1465524	+	TSS_014491	1000	121	0	5	Ai	antisense to gene(s) PMM1524;
1465603	-	TSS_034650	1000	850	0	84	I	within gene(s) PMM1524;
1465640	-	TSS_034660	1000	683	0	3	I	within gene(s) PMM1524;
1465661	-	TSS_034665	1000	7712	0	51	I	within gene(s) PMM1524;
1465736	-	TSS_034680	1000	168	0	0	I	within gene(s) PMM1524;

1465769	-	TSS_034681	1000	140	0	0 I	within gene(s) PMM1524;
1465793	-	TSS_034684	1000	301	0	12 I	within gene(s) PMM1524;
1465805	-	TSS_034686	1000	149	0	15 I	within gene(s) PMM1524;
1465843	-	TSS_034690	1000	289	0	34 I	within gene(s) PMM1524;
1465885	-	TSS_034699	1000	265	0	1 I	within gene(s) PMM1524;
1465907	-	TSS_034704	1000	346	0	15 I	within gene(s) PMM1524;
1465934	-	TSS_034706	1000	159	0	0 I	within gene(s) PMM1524;
1465964	-	TSS_034710	1000	822	0	21 I	within gene(s) PMM1524;
1465998	+	TSS_014494	1000	291	0	0 Ai	antisense to gene(s) PMM1524;
1466014	-	TSS_034716	1000	214	0	2 I	within gene(s) PMM1524;
1466027	-	TSS_034720	1000	834	0	12 I	within gene(s) PMM1524;
1466034	+	TSS_014495	1000	131	0	0 Ai	antisense to gene(s) PMM1524;
1466051	-	TSS_034724	1000	217	0	0 I	within gene(s) PMM1524;
1466114	-	TSS_034725	1000	2041	0	3 I	within gene(s) PMM1524;
1466147	-	TSS_034728	1000	274	0	5 I	within gene(s) PMM1524;
1466180	+	TSS_014498	1000	1821	0	2 Ai	antisense to gene(s) PMM1524;
1466189	-	TSS_034735	1000	1089	0	27 I	within gene(s) PMM1524;
1466213	-	TSS_034742	1000	213	0	9 I	within gene(s) PMM1524;
1466231	-	TSS_034746	1000	280	0	10 I	within gene(s) PMM1524;
1466264	-	TSS_034753	1000	411	0	30 I	within gene(s) PMM1524;
1466300	-	TSS_034757	1000	400	0	0 I	within gene(s) PMM1524;
1466318	-	TSS_034759	1000	127	0	0 I	within gene(s) PMM1524;
1466330	-	TSS_034760	1000	244	0	12 I	within gene(s) PMM1524;
1466354	-	TSS_034765	1000	379	0	1 I	within gene(s) PMM1524;
1466366	-	TSS_034768	1000	616	0	3 I	within gene(s) PMM1524;
1466429	-	TSS_034771	1000	420	0	7 I	within gene(s) PMM1524;
1466463	-	TSS_034774	1000	3142	0	2 I	within gene(s) PMM1524;
1466519	-	TSS_034775	1000	8337	0	0 P	54nt upstream of gene PMM1524;
1466585	-	TSS_034776	1000	1279	0	0 P	120nt upstream of gene PMM1524;
1468500	-	TSS_034778	1000	176	0	0 Ai	antisense to gene(s) PMM1525;
1468533	-	TSS_034780	1000	323	0	0 Ai	antisense to gene(s) PMM1525;
1468704	+	TSS_014509	1000	101	0	7 P	21nt upstream of gene PMM1526;
1469172	+	TSS_014511	1000	293	0	0 I	within gene(s) PMM1526;
1469928	-	TSS_034786	1000	117	0	0 I	within gene(s) PMM1527;
1470923	-	TSS_034789	1000	143	0	0 P	35nt upstream of gene PMM1527;
1470954	+	TSS_014517	1000	775	0	2 P	15nt upstream of gene PMM1528;
1471756	+	TSS_014522	1000	393	0	0 Ai	antisense to gene(s) PMM1529;
1473040	-	TSS_034824	1000	408	0	18 IP	within gene(s) PMM1531; 169nt upstream of gene PMM1530;
1473343	-	TSS_034835	1000	165	0	6 IP	within gene(s) PMM1532; 48nt upstream of gene PMM1531;
1473409	-	TSS_034837	1000	339	0	39 IP	within gene(s) PMM1532; 114nt upstream of gene PMM1531;
1473466	-	TSS_034850	1000	110	0	0 IP	within gene(s) PMM1532; 171nt upstream of gene PMM1531;
1473735	-	TSS_034870	1000	157	0	2 I	within gene(s) PMM1532;
1473750	-	TSS_034871	1000	114	0	0 P	14nt upstream of gene PMM1532;
1473764	-	TSS_034872	1000	3641	0	4 P	28nt upstream of gene PMM1532;
1474126	-	TSS_034877	1000	197	0	0 I	within gene(s) PMM1533;
1474891	-	TSS_034886	1000	175	0	12 IP	within gene(s) PMM1534; 232nt upstream of gene PMM1533;
1474906	-	TSS_034889	1000	241	0	7 IP	within gene(s) PMM1534; 247nt upstream of gene PMM1533;
1474933	-	TSS_034894	1000	164	0	6 I	within gene(s) PMM1534;
1474954	-	TSS_034896	1000	160	0	8 I	within gene(s) PMM1534;
1474993	-	TSS_034902	1000	324	0	6 I	within gene(s) PMM1534;
1475170	-	TSS_034908	1000	153	0	0 IP	within gene(s) PMM1535; 123nt upstream of gene PMM1534;
1475257	-	TSS_034911	1000	320	0	0 IP	within gene(s) PMM1535; 210nt upstream of gene PMM1534;
1475305	-	TSS_034914	1000	987	0	0 I	within gene(s) PMM1535;
1475407	-	TSS_034917	1000	141	0	4 I	within gene(s) PMM1535;
1475507	-	TSS_034922	1000	307	0	0 I	within gene(s) PMM1535;
1476176	-	TSS_034965	1000	479	0	17 IP	within gene(s) PMM1536; 175nt upstream of gene PMM1535;
1476251	-	TSS_034975	1000	251	0	15 IP	within gene(s) PMM1536; 250nt upstream of gene PMM1535;
1476308	-	TSS_034984	1000	167	0	6 I	within gene(s) PMM1536;
1476326	-	TSS_034989	1000	439	0	24 I	within gene(s) PMM1536;
1476356	-	TSS_034995	1000	149	0	0 I	within gene(s) PMM1536;
1476546	+	TSS_014545	1000	135	0	7 Ai	antisense to gene(s) PMM1537;
1476569	-	TSS_035015	1000	157	0	0 IP	within gene(s) PMM1537; 129nt upstream of gene PMM1536;
1476583	+	TSS_014549	1000	120	0	0 Ai	antisense to gene(s) PMM1537;
1476679	+	TSS_014552	1000	306	0	0 Ai	antisense to gene(s) PMM1537;
1476725	-	TSS_035030	1000	165	0	0 I	within gene(s) PMM1537;
1476737	-	TSS_035031	1000	198	0	18 I	within gene(s) PMM1537;
1476767	-	TSS_035039	1000	163	0	3 I	within gene(s) PMM1537;
1476782	-	TSS_035042	1000	157	0	18 I	within gene(s) PMM1537;
1478505	-	TSS_035092	1000	320	0	0 I	within gene(s) PMM1540;
1478682	+	TSS_014562	1000	234	0	0 Ai	antisense to gene(s) PMM1540;
1479634	-	TSS_035149	1000	172	0	9 IP	within gene(s) PMM1542; 217nt upstream of gene PMM1541;
1479671	+	TSS_014572	1000	117	0	0 Ai	antisense to gene(s) PMM1542;
1479684	+	TSS_014573	1000	691	0	0 Ai	antisense to gene(s) PMM1542;
1479695	+	TSS_014574	1000	163	0	1 Ai	antisense to gene(s) PMM1542;
1479751	-	TSS_035161	1000	115	0	15 I	within gene(s) PMM1542;
1479787	-	TSS_035165	1000	108	0	21 I	within gene(s) PMM1542;
1479809	+	TSS_014579	1000	274	0	3 Ai	antisense to gene(s) PMM1542;

1479894	-	TSS_035181	1000	101	0	0	I	within gene(s) PMM1542;
1479903	+	TSS_014583	1000	133	0	2	Ai	antisense to gene(s) PMM1542;
1479910	-	TSS_035184	1000	154	0	6	I	within gene(s) PMM1542;
1479946	-	TSS_035188	1000	116	0	0	I	within gene(s) PMM1542;
1479953	+	TSS_014586	1000	210	0	1	Ai	antisense to gene(s) PMM1542;
1479980	+	TSS_014587	1000	112	0	0	Ai	antisense to gene(s) PMM1542;
1480021	-	TSS_035190	1000	113	0	9	I	within gene(s) PMM1542;
1480042	-	TSS_035194	1000	214	0	3	P	0nt upstream of gene PMM1542;
1480111	-	TSS_035196	1000	108	0	0	IP	within gene(s) PMM1543; 69nt upstream of gene PMM1542;
1480219	-	TSS_035205	1000	148	0	15	IP	within gene(s) PMM1543; 177nt upstream of gene PMM1542;
1480246	-	TSS_035210	1000	288	0	12	IP	within gene(s) PMM1543; 204nt upstream of gene PMM1542;
1480267	-	TSS_035212	1000	191	0	30	IP	within gene(s) PMM1543; 225nt upstream of gene PMM1542;
1480470	-	TSS_035237	1000	186	0	12	IP	within gene(s) PMM1544; 44nt upstream of gene PMM1543;
1480500	-	TSS_035241	1000	259	0	7	IP	within gene(s) PMM1544; 74nt upstream of gene PMM1543;
1480526	-	TSS_035246	1000	409	0	48	IP	within gene(s) PMM1544; 100nt upstream of gene PMM1543;
1480601	+	TSS_014592	1000	104	0	0	Ai	antisense to gene(s) PMM1544;
1480604	-	TSS_035261	1000	8785	0	12	IP	within gene(s) PMM1544; 178nt upstream of gene PMM1543;
1480623	-	TSS_035265	1000	124	0	12	IP	within gene(s) PMM1544; 197nt upstream of gene PMM1543;
1480632	+	TSS_014595	1000	180	0	3	Ai	antisense to gene(s) PMM1544;
1480662	-	TSS_035268	1000	335	0	3	IP	within gene(s) PMM1544; 236nt upstream of gene PMM1543;
1480779	-	TSS_035274	1000	142	0	19	I	within gene(s) PMM1544;
1480830	-	TSS_035289	1000	348	0	46	I	within gene(s) PMM1544;
1480875	-	TSS_035299	1000	158	0	33	I	within gene(s) PMM1544;
1480959	-	TSS_035319	1000	193	0	24	I	within gene(s) PMM1544;
1480992	-	TSS_035325	1000	513	0	1	P	12nt upstream of gene PMM1544;
1481060	+	TSS_014603	1000	331	0	4	Ai	antisense to gene(s) PMM1545;
1481067	-	TSS_035329	1000	580	0	1	IP	within gene(s) PMM1545; 87nt upstream of gene PMM1544;
1481088	-	TSS_035330	1000	218	0	3	IP	within gene(s) PMM1545; 108nt upstream of gene PMM1544;
1481118	-	TSS_035335	1000	186	0	13	IP	within gene(s) PMM1545; 138nt upstream of gene PMM1544;
1481181	-	TSS_035345	1000	129	0	3	IP	within gene(s) PMM1545; 201nt upstream of gene PMM1544;
1481361	-	TSS_035355	1000	138	0	3	I	within gene(s) PMM1545;
1481443	-	TSS_035359	1000	345	0	27	IP	within gene(s) PMM1546; 49nt upstream of gene PMM1545;
1481503	-	TSS_035371	1000	101	0	9	IP	within gene(s) PMM1546; 109nt upstream of gene PMM1545;
1481518	-	TSS_035374	1000	207	0	2	IP	within gene(s) PMM1546; 124nt upstream of gene PMM1545;
1481554	-	TSS_035382	1000	153	0	9	IP	within gene(s) PMM1546; 160nt upstream of gene PMM1545;
1481701	-	TSS_035391	1000	174	0	6	I	within gene(s) PMM1546;
1481752	-	TSS_035401	1000	250	0	4	I	within gene(s) PMM1546;
1481803	-	TSS_035403	1000	345	0	1	I	within gene(s) PMM1546;
1481827	-	TSS_035407	1000	190	0	21	I	within gene(s) PMM1546;
1481890	-	TSS_035415	1000	148	0	5	I	within gene(s) PMM1546;
1481953	-	TSS_035422	1000	106	0	0	P	12nt upstream of gene PMM1546;
1482034	-	TSS_035426	1000	727	0	1	IP	within gene(s) PMM1547; 93nt upstream of gene PMM1546;
1482391	-	TSS_035443	1000	332	0	18	IP	within gene(s) PMM1548; 18nt upstream of gene PMM1547;
1482512	+	TSS_014616	1000	303	0	0	Ai	antisense to gene(s) PMM1548;
1482526	+	TSS_014617	1000	129	0	1	Ai	antisense to gene(s) PMM1548;
1482634	-	TSS_035464	1000	146	0	0	I	within gene(s) PMM1548;
1482655	-	TSS_035470	1000	144	0	12	I	within gene(s) PMM1548;
1482682	-	TSS_035474	1000	172	0	33	I	within gene(s) PMM1548;
1482715	-	TSS_035481	1000	215	0	6	I	within gene(s) PMM1548;
1482753	-	TSS_035485	1000	923	0	9	IP	within gene(s) PMM1549; 14nt upstream of gene PMM1548;
1482821	-	TSS_035491	1000	409	0	5	IP	within gene(s) PMM1549; 82nt upstream of gene PMM1548;
1482876	-	TSS_035498	1000	806	0	27	IP	within gene(s) PMM1549; 137nt upstream of gene PMM1548;
1483028	-	TSS_035504	1000	193	0	0	IP	within gene(s) PMM1550; 26nt upstream of gene PMM1549;
1483243	-	TSS_035531	1000	311	0	26	IP	within gene(s) PMM1551; 14nt upstream of gene PMM1550;
1483479	-	TSS_035549	1000	111	0	15	IP	within gene(s) PMM1551; 250nt upstream of gene PMM1550;
1483513	-	TSS_035558	1000	139	0	15	I	within gene(s) PMM1551;
1483528	-	TSS_035561	1000	182	0	9	I	within gene(s) PMM1551;
1483606	-	TSS_035569	1000	131	0	18	I	within gene(s) PMM1551;
1483672	-	TSS_035577	1000	160	0	19	I	within gene(s) PMM1551;
1483696	-	TSS_035581	1000	109	0	3	I	within gene(s) PMM1551;
1483720	-	TSS_035582	1000	227	0	1	P	12nt upstream of gene PMM1551;
1483738	+	TSS_014636	1000	152	0	0	Ai	antisense to gene(s) PMM1552;
1483758	-	TSS_035591	1000	202	0	9	IP	within gene(s) PMM1552; 50nt upstream of gene PMM1551;
1483845	-	TSS_035602	1000	132	0	33	IP	within gene(s) PMM1552; 137nt upstream of gene PMM1551;
1483902	-	TSS_035607	1000	140	0	1	IP	within gene(s) PMM1552; 194nt upstream of gene PMM1551;
1483975	+	TSS_014640	1000	1715	0	1	Ai	antisense to gene(s) PMM1552;
1483989	-	TSS_035615	1000	172	0	3	I	within gene(s) PMM1552;
1484028	-	TSS_035625	1000	126	0	27	I	within gene(s) PMM1552;
1484058	-	TSS_035628	1000	129	0	2	I	within gene(s) PMM1552;
1484143	+	TSS_014644	1000	141	0	0	Ai	antisense to gene(s) PMM1552;
1484217	+	TSS_014647	1000	156	0	1	Ai	antisense to gene(s) PMM1552;
1484304	-	TSS_035650	1000	218	0	15	I	within gene(s) PMM1552;
1484358	-	TSS_035662	1000	106	0	12	I	within gene(s) PMM1552;
1484379	+	TSS_014650	1000	254	0	0	Ai	antisense to gene(s) PMM1552;
1484436	-	TSS_035667	1000	157	0	0	I	within gene(s) PMM1552;
1484468	-	TSS_035668	1000	104	0	0	IP	within gene(s) PMM1553; 17nt upstream of gene PMM1552;
1484481	-	TSS_035669	1000	125	0	2	IP	within gene(s) PMM1553; 30nt upstream of gene PMM1552;

1484546	-	TSS_035672	1000	101	0	9 IP	within gene(s) PMM1553; 95nt upstream of gene PMM1552;
1484597	-	TSS_035677	1000	107	0	9 IP	within gene(s) PMM1553; 146nt upstream of gene PMM1552;
1484630	-	TSS_035684	1000	461	0	36 IP	within gene(s) PMM1553; 179nt upstream of gene PMM1552;
1484681	-	TSS_035696	1000	132	0	21 IP	within gene(s) PMM1553; 230nt upstream of gene PMM1552;
1484792	-	TSS_035712	1000	149	0	6 I	within gene(s) PMM1553;
1484971	-	TSS_035725	1000	948	0	57 IP	within gene(s) PMM1554; 134nt upstream of gene PMM1553;
1485026	-	TSS_035738	1000	141	0	0 IP	within gene(s) PMM1554; 189nt upstream of gene PMM1553;
1485041	-	TSS_035739	1000	156	0	9 IP	within gene(s) PMM1554; 204nt upstream of gene PMM1553;
1485191	+	TSS_014654	1000	195	0	2 Ai	antisense to gene(s) PMM1555;
1485223	+	TSS_014656	1000	186	0	0 Ai	antisense to gene(s) PMM1555;
1485334	-	TSS_035765	1000	743	0	69 IP	within gene(s) PMM1555; 221nt upstream of gene PMM1554;
1485370	-	TSS_035776	1000	2546	0	10 I	within gene(s) PMM1555;
1485403	-	TSS_035779	1000	133	0	0 I	within gene(s) PMM1555;
1485407	+	TSS_014660	1000	233	0	9 Ai	antisense to gene(s) PMM1555;
1485418	-	TSS_035781	1000	324	0	9 I	within gene(s) PMM1555;
1485445	-	TSS_035784	1000	209	0	18 I	within gene(s) PMM1555;
1485484	-	TSS_035792	1000	255	0	21 I	within gene(s) PMM1555;
1485505	-	TSS_035797	1000	291	0	24 I	within gene(s) PMM1555;
1485534	+	TSS_014665	1000	310	0	1 Ai	antisense to gene(s) PMM1555;
1485538	-	TSS_035806	1000	120	0	18 I	within gene(s) PMM1555;
1485568	+	TSS_014667	1000	125	0	1 Ai	antisense to gene(s) PMM1555;
1485574	-	TSS_035814	1000	153	0	6 I	within gene(s) PMM1555;
1485667	-	TSS_035825	1000	298	0	42 I	within gene(s) PMM1555;
1485685	-	TSS_035828	1000	113	0	0 I	within gene(s) PMM1555;
1485699	-	TSS_035829	1000	114	0	13 I	within gene(s) PMM1555;
1485727	-	TSS_035834	1000	103	0	4 I	within gene(s) PMM1555;
1485748	+	TSS_014668	1000	299	0	0 Ai	antisense to gene(s) PMM1555;
1485766	-	TSS_035842	1000	165	0	24 I	within gene(s) PMM1555;
1485778	-	TSS_035846	1000	139	0	0 I	within gene(s) PMM1555;
1485826	-	TSS_035850	1000	171	0	3 I	within gene(s) PMM1555;
1485868	-	TSS_035852	1000	156	0	9 I	within gene(s) PMM1555;
1485958	-	TSS_035860	1000	147	0	6 I	within gene(s) PMM1555;
1486033	-	TSS_035872	1000	106	0	1 P	12nt upstream of gene PMM1555;
1486047	+	TSS_014673	1000	129	0	1 Ai	antisense to gene(s) PMM1556;
1486054	-	TSS_035874	1000	203	0	3 IP	within gene(s) PMM1556; 33nt upstream of gene PMM1555;
1486078	-	TSS_035880	1000	179	0	15 IP	within gene(s) PMM1556; 57nt upstream of gene PMM1555;
1486129	-	TSS_035884	1000	348	0	1 IP	within gene(s) PMM1556; 108nt upstream of gene PMM1555;
1486161	-	TSS_035890	1000	779	0	12 IP	within gene(s) PMM1556; 140nt upstream of gene PMM1555;
1486258	-	TSS_035897	1000	110	0	6 IP	within gene(s) PMM1556; 237nt upstream of gene PMM1555;
1486353	-	TSS_035905	1000	159	0	21 IP	within gene(s) PMM1557; 17nt upstream of gene PMM1556;
1486398	-	TSS_035911	1000	177	0	6 IP	within gene(s) PMM1557; 62nt upstream of gene PMM1556;
1486430	-	TSS_035914	1000	775	0	4 IP	within gene(s) PMM1557; 94nt upstream of gene PMM1556;
1486728	-	TSS_035928	1000	114	0	12 I	within gene(s) PMM1557;
1486752	-	TSS_035931	1000	162	0	16 I	within gene(s) PMM1557;
1486788	-	TSS_035938	1000	112	0	39 I	within gene(s) PMM1557;
1486899	-	TSS_035952	1000	539	0	18 I	within gene(s) PMM1557;
1486929	-	TSS_035957	1000	204	0	12 I	within gene(s) PMM1557;
1486979	-	TSS_035964	1000	326	0	14 IP	within gene(s) PMM1558; 14nt upstream of gene PMM1557;
1487021	+	TSS_014685	1000	256	0	0 Ai	antisense to gene(s) PMM1558;
1487036	-	TSS_035973	1000	219	0	1 IP	within gene(s) PMM1558; 71nt upstream of gene PMM1557;
1487162	-	TSS_035981	1000	139	0	0 IP	within gene(s) PMM1558; 197nt upstream of gene PMM1557;
1487198	-	TSS_035985	1000	157	0	0 IP	within gene(s) PMM1558; 233nt upstream of gene PMM1557;
1487222	-	TSS_035987	1000	432	0	27 I	within gene(s) PMM1558;
1487282	-	TSS_036003	1000	207	0	1 I	within gene(s) PMM1558;
1487366	-	TSS_036009	1000	155	0	2 I	within gene(s) PMM1558;
1487886	+	TSS_014690	1000	104	0	8 P	22nt upstream of gene PMM1559;
1487895	+	TSS_014692	1000	501	0	6 P	13nt upstream of gene PMM1559;
1489480	+	TSS_014703	1000	370	0	6 P	92nt upstream of gene PMM1561;
1490373	+	TSS_014708	1000	6580	0	2 P	85nt upstream of gene PMM1562;
1490482	+	TSS_014712	1000	171	0	0 I	within gene(s) PMM1562;
1490500	+	TSS_014714	1000	103	0	3 I	within gene(s) PMM1562;
1490509	+	TSS_014715	1000	177	0	9 I	within gene(s) PMM1562;
1490540	+	TSS_014723	1000	124	0	10 I	within gene(s) PMM1562;
1490563	+	TSS_014729	1000	305	0	15 I	within gene(s) PMM1562;
1490578	+	TSS_014736	1000	392	0	17 I	within gene(s) PMM1562;
1490614	+	TSS_014744	1000	101	0	0 I	within gene(s) PMM1562;
1490629	+	TSS_014745	1000	134	0	0 I	within gene(s) PMM1562;
1490650	+	TSS_014748	1000	822	0	15 I	within gene(s) PMM1562;
1490668	+	TSS_014750	1000	241	0	9 I	within gene(s) PMM1562;
1490692	+	TSS_014756	1000	335	0	13 I	within gene(s) PMM1562;
1490716	+	TSS_014761	1000	199	0	6 I	within gene(s) PMM1562;
1490726	-	TSS_036025	1000	107	0	0 Ai	antisense to gene(s) PMM1562;
1490734	+	TSS_014763	1000	245	0	0 I	within gene(s) PMM1562;
1490743	+	TSS_014764	1000	271	0	9 I	within gene(s) PMM1562;
1490788	-	TSS_036026	1000	155	0	2 Ai	antisense to gene(s) PMM1562;
1490803	+	TSS_014780	1000	560	0	68 I	within gene(s) PMM1562;
1490881	+	TSS_014798	1000	609	0	51 I	within gene(s) PMM1562;

1490923	+	TSS_014807	1000	104	0	9	I	within gene(s) PMM1562;
1490944	+	TSS_014808	1000	104	0	6	I	within gene(s) PMM1562;
1490956	-	TSS_036029	1000	164	0	3	Ai	antisense to gene(s) PMM1562;
1490959	+	TSS_014810	1000	141	0	15	I	within gene(s) PMM1562;
1490986	+	TSS_014815	1000	127	0	0	I	within gene(s) PMM1562;
1491010	+	TSS_014818	1000	139	0	9	I	within gene(s) PMM1562;
1491011	-	TSS_036032	1000	203	0	0	Ai	antisense to gene(s) PMM1562;
1491028	+	TSS_014819	1000	179	0	3	I	within gene(s) PMM1562;
1491067	+	TSS_014828	1000	496	0	30	I	within gene(s) PMM1562;
1491070	-	TSS_036033	1000	175	0	1	Ai	antisense to gene(s) PMM1562;
1491092	+	TSS_014833	1000	214	0	13	I	within gene(s) PMM1562;
1491099	-	TSS_036035	1000	102	0	0	Ai	antisense to gene(s) PMM1562;
1491112	+	TSS_014836	1000	422	0	24	I	within gene(s) PMM1562;
1491139	+	TSS_014844	1000	265	0	2	I	within gene(s) PMM1562;
1491148	+	TSS_014845	1000	143	0	12	I	within gene(s) PMM1562;
1491169	+	TSS_014850	1000	179	0	12	I	within gene(s) PMM1562;
1491193	+	TSS_014856	1000	161	0	63	I	within gene(s) PMM1562;
1491265	+	TSS_014873	1000	114	0	0	I	within gene(s) PMM1562;
1491376	+	TSS_014880	1000	112	0	6	I	within gene(s) PMM1562;
1491389	+	TSS_014882	1000	107	0	8	I	within gene(s) PMM1562;
1491478	+	TSS_014897	1000	379	0	96	I	within gene(s) PMM1562;
1491629	-	TSS_036043	1000	149	0	0	I	within gene(s) PMM1563;
1491832	-	TSS_036046	1000	169	0	2	P	14nt upstream of gene PMM1563;
1493560	-	TSS_036049	1000	113	0	0	Ai	antisense to gene(s) PMM1565;
1494281	+	TSS_014926	1000	287	0	0	Ai	antisense to gene(s) PMM1566;
1494828	+	TSS_014929	1000	131	0	8	Ai	antisense to gene(s) PMM1566;
1495329	+	TSS_014931	1000	1822	0	5	Ai	antisense to gene(s) PMM1566;
1495758	-	TSS_036061	1000	1052	0	2	PAi	19nt upstream of gene PMM1566; antisense to gene(s) PMM1567;
1495801	-	TSS_036063	1000	246	0	0	PAi	62nt upstream of gene PMM1566; antisense to gene(s) PMM1567;
1496620	+	TSS_014936	1000	977	0	0	IAd	within gene(s) PMM1567; antisense to gene(s) PMM1568 (11nt downstream);
1496633	+	TSS_014937	1000	103	0	0	Ai	antisense to gene(s) PMM1568;
1496755	-	TSS_036068	1000	166	0	4	I	within gene(s) PMM1568;
1496904	-	TSS_036078	1000	152	0	2	I	within gene(s) PMM1568;
1497016	-	TSS_036084	1000	11613	0	10	P	21nt upstream of gene PMM1568;
1497103	-	TSS_036088	1000	159	0	0	P	108nt upstream of gene PMM1568;
1499500	-	TSS_036100	1000	521	0	2	I	within gene(s) PMM1570;
1499641	-	TSS_036102	1000	263	0	0	P	35nt upstream of gene PMM1570;
1499646	+	TSS_014958	1000	372	0	11	P	71nt upstream of gene PMM1571;
1499680	-	TSS_036103	1000	172	0	0	P	74nt upstream of gene PMM1570;
1499710	+	TSS_014963	1000	276	0	0	P	7nt upstream of gene PMM1571;
1501198	+	TSS_014969	1000	159	0	0	Ai	antisense to gene(s) PMM1573;
1502167	+	TSS_014971	1000	118	0	3	O	-
1502320	-	TSS_036110	1000	177	0	0	I	within gene(s) PMM1574;
1502938	-	TSS_036121	1000	161	0	3	IP	within gene(s) PMM1575; 204nt upstream of gene PMM1574;
1503407	+	TSS_014977	1000	217	0	1	Ai	antisense to gene(s) PMM1575;
1503685	-	TSS_036142	1000	180	0	3	I	within gene(s) PMM1575;
1503721	-	TSS_036150	1000	134	0	33	I	within gene(s) PMM1575;
1504039	-	TSS_036161	1000	208	0	3	I	within gene(s) PMM1575;
1504888	-	TSS_036179	1000	198	0	0	I	within gene(s) PMM1575;
1504964	-	TSS_036182	1000	183	0	0	I	within gene(s) PMM1575;
1505392	-	TSS_036192	1000	155	0	10	I	within gene(s) PMM1575;
1505673	-	TSS_036200	1000	127	0	0	I	within gene(s) PMM1575;
1505706	-	TSS_036201	1000	724	0	0	I	within gene(s) PMM1575;
1507214	-	TSS_036207	1000	102	0	0	I	within gene(s) PMM1577;
1508411	-	TSS_036216	1000	478	0	1	P	11nt upstream of gene PMM1577;
1508562	+	TSS_014991	1000	334	0	1	Ai	antisense to gene(s) PMM1578;
1508766	+	TSS_014993	1000	177	0	0	Ai	antisense to gene(s) PMM1578;
1508792	-	TSS_036241	1000	316	0	4	I	within gene(s) PMM1578;
1508804	-	TSS_036243	1000	153	0	0	I	within gene(s) PMM1578;
1508834	-	TSS_036245	1000	304	0	10	I	within gene(s) PMM1578;
1508855	-	TSS_036249	1000	442	0	10	I	within gene(s) PMM1578;
1508882	-	TSS_036253	1000	120	0	3	P	0nt upstream of gene PMM1578;
1510763	-	TSS_036259	1000	126	0	0	I	within gene(s) PMM1580;
1511027	+	TSS_015001	1000	874	0	1	Ai	antisense to gene(s) PMM1580;
1511176	+	TSS_015002	1000	126	0	0	Ai	antisense to gene(s) PMM1580;
1512795	-	TSS_036268	1000	720	0	2	P	92nt upstream of gene PMM1581;
1513258	-	TSS_036272	1000	123	0	0	Ai	antisense to gene(s) PMM1582;
1513707	-	TSS_036273	1000	623	0	0	Ai	antisense to gene(s) PMM1582;
1513724	-	TSS_036274	1000	716	0	0	Ai	antisense to gene(s) PMM1582;
1514262	-	TSS_036276	1000	132	0	2	I	within gene(s) PMM1583;
1515544	-	TSS_036282	1000	519	0	0	I	within gene(s) PMM1585;
1518815	-	TSS_036309	1000	286	0	3	I	within gene(s) PMM1589;
1519102	-	TSS_036320	1000	161	0	1	I	within gene(s) PMM1589;
1519244	-	TSS_036323	1000	949	0	1	P	24nt upstream of gene PMM1589;
1520122	+	TSS_015033	1000	108	0	0	I	within gene(s) PMM1590;
1523398	-	TSS_036340	1000	109	0	0	I	within gene(s) PMM1594;
1523570	-	TSS_036343	1000	1254	0	1	P	23nt upstream of gene PMM1594;

1524215	+	TSS_015045	1000	223	0	2	P	40nt upstream of gene PMM1596;
1525299	+	TSS_015068	1000	107	0	9	I	within gene(s) PMM1596;
1528712	-	TSS_036372	1000	149	0	5	I	within gene(s) PMM1599;
1528738	-	TSS_036376	1000	101	0	0	I	within gene(s) PMM1599;
1528833	-	TSS_036379	1000	316	0	2	P	16nt upstream of gene PMM1599;
1529108	-	TSS_036384	1000	116	0	3	I	within gene(s) PMM1600;
1529156	-	TSS_036388	1000	265	0	3	I	within gene(s) PMM1600;
1529429	-	TSS_036404	1000	140	0	18	I	within gene(s) PMM1600;
1529501	-	TSS_036410	1000	274	0	0	I	within gene(s) PMM1600;
1529522	-	TSS_036412	1000	189	0	6	I	within gene(s) PMM1600;
1529573	-	TSS_036417	1000	146	0	9	I	within gene(s) PMM1600;
1529888	-	TSS_036436	1000	175	0	9	I	within gene(s) PMM1600;
1530109	+	TSS_015094	1000	464	0	0	Ai	antisense to gene(s) PMM1600;
1530254	-	TSS_036450	1000	4533	0	1	P	27nt upstream of gene PMM1600;
1530284	-	TSS_036455	1000	274	0	10	P	57nt upstream of gene PMM1600;
1530378	+	TSS_015099	1000	10961	0	3	P	3nt upstream of gene PMM1601;
1530948	+	TSS_015128	1000	177	0	6	I	within gene(s) PMM1601;
1531203	-	TSS_036458	1000	104	0	1	Ai	antisense to gene(s) PMM1601;
1531302	+	TSS_015166	1000	101	0	3	I	within gene(s) PMM1601;
1531400	-	TSS_036459	1000	372	0	0	Ai	antisense to gene(s) PMM1601;
1532253	+	TSS_015222	1000	132	0	3	I	within gene(s) PMM1601;
1533530	+	TSS_015238	1000	138	0	0	Ai	antisense to gene(s) PMM1602;
1533586	+	TSS_015240	1000	118	0	0	Ai	antisense to gene(s) PMM1602;
1533752	-	TSS_036476	1000	16470	0	8	P	14nt upstream of gene PMM1602;
1533794	-	TSS_036480	1000	1643	0	1	P	56nt upstream of gene PMM1602;
1533863	-	TSS_036481	1000	154	0	0	P	125nt upstream of gene PMM1602;
1534044	-	TSS_036483	1000	154	0	4	O	-
1534092	-	TSS_036485	1000	541	0	0	O	-
1534100	-	TSS_036486	1000	170	0	1	O	-
1534301	-	TSS_036493	1000	258	0	2	O	-
1535304	+	TSS_015385	1000	189	0	3	I	within gene(s) PMM1605;
1535492	-	TSS_036499	1000	201	0	0	PAi	224nt upstream of gene PMM1604; antisense to gene(s) PMM1605;
1537533	-	TSS_036518	1000	673	0	1	I	within gene(s) PMM1606;
1537744	+	TSS_015393	1000	338	0	0	Ai	antisense to gene(s) PMM1606;
1537998	-	TSS_036535	1000	248	0	18	I	within gene(s) PMM1607;
1538040	-	TSS_036543	1000	137	0	7	I	within gene(s) PMM1607;
1538103	-	TSS_036546	1000	201	0	0	P	30nt upstream of gene PMM1607;
1538226	+	TSS_015403	1000	132	0	0	IP	within gene(s) PMM1608; 231nt upstream of gene PMM1609;
1538247	+	TSS_015407	1000	102	0	11	IP	within gene(s) PMM1608; 210nt upstream of gene PMM1609;
1538256	+	TSS_015408	1000	110	0	0	IP	within gene(s) PMM1608; 201nt upstream of gene PMM1609;
1538307	+	TSS_015413	1000	172	0	0	IP	within gene(s) PMM1608; 150nt upstream of gene PMM1609;
1539467	+	TSS_015433	1000	2654	0	2	I	within gene(s) PMM1609;
1539568	+	TSS_015436	1000	842	0	2	IP	within gene(s) PMM1609; 176nt upstream of gene PMM1610;
1539716	-	TSS_036553	1000	139	0	0	Ad	antisense to gene(s) PMM1609 (16nt downstream);
1539778	-	TSS_036555	1000	1338	0	4	Ai	antisense to gene(s) PMM1610;
1539930	+	TSS_015451	1000	252	0	30	I	within gene(s) PMM1610;
1539957	+	TSS_015453	1000	114	0	15	I	within gene(s) PMM1610;
1540059	+	TSS_015463	1000	140	0	9	I	within gene(s) PMM1610;
1540202	-	TSS_036559	1000	156	0	0	Ai	antisense to gene(s) PMM1610;
1540236	+	TSS_015471	1000	123	0	1	I	within gene(s) PMM1610;
1540533	+	TSS_015500	1000	277	0	9	I	within gene(s) PMM1610;
1540599	+	TSS_015506	1000	222	0	4	I	within gene(s) PMM1610;
1540634	-	TSS_036566	1000	114	0	0	Ai	antisense to gene(s) PMM1610;
1540845	+	TSS_015517	1000	109	0	6	I	within gene(s) PMM1610;
1541023	-	TSS_036569	1000	173	0	0	Ai	antisense to gene(s) PMM1610;
1541079	+	TSS_015538	1000	111	0	0	I	within gene(s) PMM1610;
1541091	+	TSS_015539	1000	227	0	0	I	within gene(s) PMM1610;
1541109	+	TSS_015543	1000	281	0	18	I	within gene(s) PMM1610;
1541313	+	TSS_015551	1000	124	0	8	I	within gene(s) PMM1610;
1542021	+	TSS_015571	1000	148	0	4	Ai	antisense to gene(s) PMM1611;
1542045	-	TSS_036589	1000	523	0	18	I	within gene(s) PMM1611;
1542093	-	TSS_036592	1000	239	0	2	I	within gene(s) PMM1611;
1542102	-	TSS_036594	1000	121	0	18	I	within gene(s) PMM1611;
1542168	-	TSS_036607	1000	121	0	14	I	within gene(s) PMM1611;
1542204	-	TSS_036613	1000	113	0	0	I	within gene(s) PMM1611;
1542336	-	TSS_036630	1000	288	0	9	I	within gene(s) PMM1611;
1542348	-	TSS_036633	1000	129	0	15	I	within gene(s) PMM1611;
1542411	-	TSS_036644	1000	129	0	6	I	within gene(s) PMM1611;
1542516	-	TSS_036656	1000	139	0	15	I	within gene(s) PMM1611;
1542549	-	TSS_036662	1000	126	0	6	I	within gene(s) PMM1611;
1542633	-	TSS_036670	1000	150	0	9	I	within gene(s) PMM1611;
1542738	-	TSS_036678	1000	194	0	15	I	within gene(s) PMM1611;
1542801	-	TSS_036686	1000	194	0	2	I	within gene(s) PMM1611;
1542810	-	TSS_036688	1000	129	0	9	I	within gene(s) PMM1611;
1542933	-	TSS_036700	1000	134	0	33	I	within gene(s) PMM1611;
1543254	-	TSS_036717	1000	3666	0	3	P	126nt upstream of gene PMM1611;
1543584	+	TSS_015584	1000	608	0	0	Ai	antisense to gene(s) PMM1613;

1543786	+	TSS_015585	1000	337	0	1	Ai	antisense to gene(s) PMM1613;
1544476	+	TSS_015594	1000	123	0	0	Ai	antisense to gene(s) PMM1613;
1544650	-	TSS_036726	1000	113	0	0	I	within gene(s) PMM1613;
1546541	+	TSS_015602	1000	151	0	4	PAi	32nt upstream of gene PMM1616; antisense to gene(s) PMM1615;
1546552	-	TSS_036739	1000	764	0	1	I	within gene(s) PMM1615;
1547526	+	TSS_015607	1000	259	0	0	Ai	antisense to gene(s) PMM1618;
1548968	-	TSS_036755	1000	154	0	0	P	25nt upstream of gene PMM1618;
1549170	-	TSS_036759	1000	136	0	10	IP	within gene(s) PMM1619; 227nt upstream of gene PMM1618;
1549210	-	TSS_036767	1000	172	0	16	I	within gene(s) PMM1619;
1549235	-	TSS_036769	1000	1102	0	5	I	within gene(s) PMM1619;
1549341	-	TSS_036778	1000	109	0	15	I	within gene(s) PMM1619;
1549500	-	TSS_036792	1000	246	0	12	I	within gene(s) PMM1619;
1549539	-	TSS_036794	1000	245	0	1	I	within gene(s) PMM1619;
1550719	-	TSS_036800	1000	117	0	0	I	within gene(s) PMM1621;
1551244	+	TSS_015622	1000	152	0	2	Ai	antisense to gene(s) PMM1622;
1551373	+	TSS_015624	1000	141	0	1	Ai	antisense to gene(s) PMM1622;
1551407	-	TSS_036807	1000	175	0	7	IP	within gene(s) PMM1622; 205nt upstream of gene PMM1621;
1551449	-	TSS_036811	1000	170	0	1	IP	within gene(s) PMM1622; 247nt upstream of gene PMM1621;
1551872	-	TSS_036825	1000	111	0	18	I	within gene(s) PMM1622;
1552273	-	TSS_036846	1000	2972	0	4	P	14nt upstream of gene PMM1622;
1552380	+	TSS_015630	1000	526	0	1	P	12nt upstream of gene PMM1623;
1552488	+	TSS_015634	1000	164	0	3	I	within gene(s) PMM1623;
1552921	+	TSS_015642	1000	185	0	0	Ai	antisense to gene(s) PMM1624;
1553709	-	TSS_036864	1000	195	0	36	I	within gene(s) PMM1625;
1553802	-	TSS_036875	1000	285	0	12	I	within gene(s) PMM1625;
1553859	-	TSS_036884	1000	258	0	5	I	within gene(s) PMM1625;
1553970	-	TSS_036893	1000	414	0	46	I	within gene(s) PMM1625;
1554015	-	TSS_036903	1000	391	0	45	I	within gene(s) PMM1625;
1554065	+	TSS_015655	1000	360	0	4	Ai	antisense to gene(s) PMM1625;
1554078	-	TSS_036918	1000	188	0	18	I	within gene(s) PMM1625;
1554099	-	TSS_036923	1000	274	0	12	I	within gene(s) PMM1625;
1554135	-	TSS_036934	1000	427	0	141	I	within gene(s) PMM1625;
1554261	-	TSS_036970	1000	151	0	0	I	within gene(s) PMM1625;
1554282	-	TSS_036971	1000	120	0	0	I	within gene(s) PMM1625;
1554300	-	TSS_036974	1000	255	0	21	I	within gene(s) PMM1625;
1554327	-	TSS_036981	1000	105	0	2	I	within gene(s) PMM1625;
1554372	-	TSS_036985	1000	136	0	3	I	within gene(s) PMM1625;
1554381	-	TSS_036986	1000	180	0	18	I	within gene(s) PMM1625;
1554450	-	TSS_036999	1000	433	0	33	I	within gene(s) PMM1625;
1554465	-	TSS_037003	1000	503	0	4	I	within gene(s) PMM1625;
1554498	-	TSS_037011	1000	1455	0	51	I	within gene(s) PMM1625;
1554543	-	TSS_037023	1000	490	0	42	I	within gene(s) PMM1625;
1554603	-	TSS_037039	1000	324	0	30	I	within gene(s) PMM1625;
1554627	-	TSS_037045	1000	232	0	0	I	within gene(s) PMM1625;
1554636	-	TSS_037046	1000	121	2.00E-09	0	I	within gene(s) PMM1625;
1554651	-	TSS_037048	1000	143	0	6	I	within gene(s) PMM1625;
1554666	-	TSS_037050	1000	316	0	3	I	within gene(s) PMM1625;
1554678	-	TSS_037052	1000	168	0	6	I	within gene(s) PMM1625;
1554690	-	TSS_037054	1000	235	0	0	I	within gene(s) PMM1625;
1554708	-	TSS_037060	1000	755	0	15	I	within gene(s) PMM1625;
1554732	-	TSS_037068	1000	424	0	31	I	within gene(s) PMM1625;
1554777	-	TSS_037076	1000	117	0	12	I	within gene(s) PMM1625;
1554819	-	TSS_037079	1000	101	0	0	I	within gene(s) PMM1625;
1554856	-	TSS_037083	1000	7457	0	4	P	16nt upstream of gene PMM1625;
1554868	+	TSS_015666	1000	215	0	0	P	22nt upstream of gene PMM1626;
1557432	+	TSS_015682	1000	276	0	1	Ai	antisense to gene(s) PMM1629;
1557493	-	TSS_037105	1000	120	0	34	I	within gene(s) PMM1629;
1557562	-	TSS_037121	1000	109	0	12	I	within gene(s) PMM1629;
1557604	-	TSS_037124	1000	190	0	4	I	within gene(s) PMM1629;
1557658	-	TSS_037129	1000	183	0	0	I	within gene(s) PMM1629;
1557709	-	TSS_037134	1000	204	0	3	I	within gene(s) PMM1629;
1557730	-	TSS_037140	1000	145	0	24	I	within gene(s) PMM1629;
1557910	-	TSS_037163	1000	120	0	12	I	within gene(s) PMM1629;
1557988	-	TSS_037169	1000	204	0	21	I	within gene(s) PMM1629;
1558291	-	TSS_037185	1000	775	0	3	P	18nt upstream of gene PMM1629;
1558425	+	TSS_015689	1000	240	0	0	Ai	antisense to gene(s) PMM1630;
1558456	+	TSS_015690	1000	243	0	0	Ai	antisense to gene(s) PMM1630;
1558619	-	TSS_037189	1000	101	0	11	I	within gene(s) PMM1630;
1558709	-	TSS_037197	1000	245	0	3	I	within gene(s) PMM1630;
1558934	-	TSS_037212	1000	102	0	0	I	within gene(s) PMM1630;
1558976	-	TSS_037217	1000	102	0	15	I	within gene(s) PMM1630;
1559126	-	TSS_037240	1000	241	0	18	I	within gene(s) PMM1630;
1559168	-	TSS_037247	1000	148	0	3	I	within gene(s) PMM1630;
1559240	+	TSS_015699	1000	1458	0	9	Ai	antisense to gene(s) PMM1630;
1559402	-	TSS_037259	1000	155	0	10	I	within gene(s) PMM1630;
1559420	-	TSS_037263	1000	175	0	18	I	within gene(s) PMM1630;
1559495	-	TSS_037277	1000	141	0	30	I	within gene(s) PMM1630;

1559546	-	TSS_037286	1000	145	0	21	I	within gene(s) PMM1630;
1559756	-	TSS_037298	1000	7710	0	9	I	within gene(s) PMM1630;
1559776	-	TSS_037303	1000	339	0	4	I	within gene(s) PMM1630;
1559806	-	TSS_037308	1000	3831	0	3	P	23nt upstream of gene PMM1630;
1561061	-	TSS_037320	1000	10626	0	16	IP	within gene(s) PMM1634; 205nt upstream of gene PMM1633;
1561536	-	TSS_037333	1000	106	0	2	I	within gene(s) PMM1634;
1561575	-	TSS_037341	1000	131	0	18	I	within gene(s) PMM1634;
1561608	-	TSS_037346	1000	144	0	9	I	within gene(s) PMM1634;
1561614	+	TSS_015709	1000	143	0	1	Ai	antisense to gene(s) PMM1634;
1561620	-	TSS_037348	1000	122	0	21	I	within gene(s) PMM1634;
1561701	+	TSS_015713	1000	257	0	2	Ai	antisense to gene(s) PMM1634;
1562815	-	TSS_037418	1000	298	0	0	I	within gene(s) PMM1634;
1563062	-	TSS_037420	1000	296	0	4	PAi	242nt upstream of gene PMM1634; antisense to gene(s) PMM1635;
1563883	+	TSS_015727	1000	173	0	5	P	41nt upstream of gene PMM1636;
1563968	-	TSS_037425	1000	392	0	0	Ai	antisense to gene(s) PMM1636;
1564062	+	TSS_015730	1000	524	0	0	I	within gene(s) PMM1636;
1564123	-	TSS_037426	1000	888	0	0	Ai	antisense to gene(s) PMM1636;
1565022	-	TSS_037429	1000	181	0	0	Ai	antisense to gene(s) PMM1637;
1565055	-	TSS_037430	1000	306	0	0	Ai	antisense to gene(s) PMM1637;
1566011	+	TSS_015754	1000	117	0	0	I	within gene(s) PMM1638;
1566190	-	TSS_037438	1000	159	0	0	Ai	antisense to gene(s) PMM1638;
1566861	+	TSS_015756	1000	114	0	0	Ai	antisense to gene(s) PMM1639;
1567106	-	TSS_037458	1000	108	0	12	I	within gene(s) PMM1639;
1567157	+	TSS_015762	1000	119	0	7	Ai	antisense to gene(s) PMM1639;
1567187	-	TSS_037474	1000	170	0	33	I	within gene(s) PMM1639;
1567265	-	TSS_037481	1000	184	0	0	I	within gene(s) PMM1639;
1567303	-	TSS_037482	1000	110	0	3	I	within gene(s) PMM1639;
1567388	+	TSS_015764	1000	110	0	0	Ai	antisense to gene(s) PMM1639;
1567784	-	TSS_037502	1000	145	0	9	I	within gene(s) PMM1639;
1567848	+	TSS_015768	1000	102	0	1	Ai	antisense to gene(s) PMM1639;
1567886	-	TSS_037511	1000	188	0	12	I	within gene(s) PMM1639;
1568020	-	TSS_037526	1000	357	0	23	I	within gene(s) PMM1639;
1568056	-	TSS_037532	1000	101	0	0	I	within gene(s) PMM1639;
1568329	+	TSS_015770	1000	249	0	0	Ai	antisense to gene(s) PMM1639;
1569177	-	TSS_037601	1000	396	0	0	P	31nt upstream of gene PMM1639;
1569383	+	TSS_015778	1000	205	0	1	I	within gene(s) PMM1640;
1570481	-	TSS_037608	1000	151	0	0	IP	within gene(s) PMM1642; 248nt upstream of gene PMM1641;
1570531	-	TSS_037612	1000	3093	0	1	I	within gene(s) PMM1642;
1570615	-	TSS_037615	1000	380	0	0	I	within gene(s) PMM1642;
1571379	-	TSS_037627	1000	169	0	0	P	16nt upstream of gene PMM1643;
1571400	+	TSS_015784	1000	120	0	0	Ad	antisense to gene(s) PMM1644 (13nt downstream);
1571519	-	TSS_037633	1000	122	0	0	IP	within gene(s) PMM1644; 156nt upstream of gene PMM1643;
1571555	-	TSS_037637	1000	235	0	21	IP	within gene(s) PMM1644; 192nt upstream of gene PMM1643;
1571578	-	TSS_037642	1000	173	0	0	IP	within gene(s) PMM1644; 215nt upstream of gene PMM1643;
1571590	-	TSS_037643	1000	124	0	0	IP	within gene(s) PMM1644; 227nt upstream of gene PMM1643;
1571608	-	TSS_037644	1000	194	0	0	IP	within gene(s) PMM1644; 245nt upstream of gene PMM1643;
1571739	+	TSS_015786	1000	239	0	0	P	14nt upstream of gene PMM1645;
1571777	+	TSS_015788	1000	332	0	1	I	within gene(s) PMM1645;
1573488	-	TSS_037647	1000	135	0	0	Ai	antisense to gene(s) PMM1645;
1576258	+	TSS_015797	1000	640	0	0	I	within gene(s) PMM1648;
1576619	-	TSS_037660	1000	161	0	0	Ai	antisense to gene(s) PMM1648;
1576861	+	TSS_015807	1000	687	0	2	I	within gene(s) PMM1648;
1578502	-	TSS_037670	1000	150	0	0	I	within gene(s) PMM1649;
1579875	+	TSS_015824	1000	106	0	0	Ai	antisense to gene(s) PMM1649;
1580087	-	TSS_037712	1000	332	0	3	P	22nt upstream of gene PMM1649;
1580887	-	TSS_037734	1000	1848	0	3	I	within gene(s) PMM1650;
1582153	+	TSS_015832	1000	157	0	0	Ai	antisense to gene(s) PMM1652;
1582233	-	TSS_037740	1000	237	0	18	I	within gene(s) PMM1652;
1582254	-	TSS_037745	1000	129	0	18	I	within gene(s) PMM1652;
1582484	+	TSS_015834	1000	235	0	0	Ai	antisense to gene(s) PMM1652;
1582766	-	TSS_037784	1000	416	0	1	I	within gene(s) PMM1652;
1582966	+	TSS_015841	1000	164	0	0	Ai	antisense to gene(s) PMM1652;
1583497	-	TSS_037843	1000	351	0	7	I	within gene(s) PMM1652;
1583628	-	TSS_037848	1000	131	0	2	I	within gene(s) PMM1652;
1584367	-	TSS_037863	1000	152	0	0	I	within gene(s) PMM1653;
1584409	-	TSS_037866	1000	5274	0	7	I	within gene(s) PMM1653;
1584725	+	TSS_015856	1000	208	0	1	Ai	antisense to gene(s) PMM1653;
1585023	-	TSS_037876	1000	1541	0	5	IP	within gene(s) PMM1654; 71nt upstream of gene PMM1653;
1586076	+	TSS_015864	1000	175	0	8	P	32nt upstream of gene PMM1655;
1587579	+	TSS_015881	1000	659	0	3	P	16nt upstream of gene PMM1656;
1587964	+	TSS_015903	1000	150	0	3	I	within gene(s) PMM1656;
1588301	+	TSS_015911	1000	2374	0	5	P	45nt upstream of gene PMM1657;
1588808	+	TSS_015927	1000	180	0	12	I	within gene(s) PMM1657;
1588838	+	TSS_015932	1000	239	0	21	I	within gene(s) PMM1657;
1588868	+	TSS_015939	1000	217	0	15	I	within gene(s) PMM1657;
1588910	+	TSS_015950	1000	178	0	24	I	within gene(s) PMM1657;
1588940	+	TSS_015957	1000	122	0	0	I	within gene(s) PMM1657;

1588997	+	TSS_015966	1000	200	0	18 I	within gene(s) PMM1657;
1589012	+	TSS_015968	1000	131	0	1 I	within gene(s) PMM1657;
1589048	+	TSS_015975	1000	954	0	12 I	within gene(s) PMM1657;
1589072	+	TSS_015978	1000	113	0	3 I	within gene(s) PMM1657;
1589264	+	TSS_015992	1000	124	0	0 I	within gene(s) PMM1657;
1590631	-	TSS_037895	1000	165	0	0 Ai	antisense to gene(s) PMM1658;
1594453	+	TSS_016011	1000	2320	0	2 P	19nt upstream of gene PMM1661;
1594701	+	TSS_016015	1000	448	0	0 I	within gene(s) PMM1662;
1596903	+	TSS_016033	1000	286	0	16 I	within gene(s) PMM1665;
1597086	+	TSS_016052	1000	114	0	15 I	within gene(s) PMM1665;
1597137	+	TSS_016062	1000	304	0	12 I	within gene(s) PMM1665;
1597536	+	TSS_016075	1000	134	0	6 I	within gene(s) PMM1665;
1599230	-	TSS_037935	1000	118	0	0 P	3nt upstream of gene PMM1667;
1599320	-	TSS_037939	1000	858	0	0 P	93nt upstream of gene PMM1667;
1599747	-	TSS_037944	1000	101	0	5 I	within gene(s) PMM1668;
1600936	+	TSS_016107	1000	344	0	0 Ai	antisense to gene(s) PMM1668;
1602622	-	TSS_038032	1000	123	0	4 I	within gene(s) PMM1669;
1604240	-	TSS_038043	1000	459	0	1 IP	within gene(s) PMM1671; 236nt upstream of gene PMM1670;
1604740	-	TSS_038050	1000	475	0	2 P	24nt upstream of gene PMM1671;
1604757	+	TSS_016120	1000	468	0	12 I	within gene(s) PMM1672;
1604952	+	TSS_016129	1000	118	0	3 I	within gene(s) PMM1672;
1604976	+	TSS_016131	1000	106	0	9 I	within gene(s) PMM1672;
1605078	+	TSS_016145	1000	102	0	1 I	within gene(s) PMM1672;
1605372	+	TSS_016157	1000	173	0	3 I	within gene(s) PMM1672;
1605445	+	TSS_016166	1000	213	0	15 I	within gene(s) PMM1672;
1605722	-	TSS_038054	1000	147	0	0 Ai	antisense to gene(s) PMM1672;
1605770	+	TSS_016173	1000	108	0	0 I	within gene(s) PMM1673;
1605791	+	TSS_016176	1000	124	0	9 I	within gene(s) PMM1673;
1605839	+	TSS_016177	1000	154	0	1 I	within gene(s) PMM1673;
1605878	+	TSS_016184	1000	154	0	30 I	within gene(s) PMM1673;
1605911	+	TSS_016191	1000	119	0	7 I	within gene(s) PMM1673;
1606013	+	TSS_016203	1000	136	0	30 I	within gene(s) PMM1673;
1606251	+	TSS_016219	1000	398	0	2 P	15nt upstream of gene PMM1674;
1606665	+	TSS_016223	1000	107	0	2 I	within gene(s) PMM1674;
1607611	+	TSS_016232	1000	948	0	2 IP	within gene(s) PMM1674; 49nt upstream of gene PMM1675;
1607768	-	TSS_038062	1000	622	0	0 Ai	antisense to gene(s) PMM1675;
1607789	+	TSS_016235	1000	105	0	0 I	within gene(s) PMM1675;
1609627	+	TSS_016243	1000	586	0	2 P	4nt upstream of gene PMM1676;
1610827	-	TSS_038077	1000	228	0	0 O	-
1611598	+	TSS_016249	1000	112	0	6 I	within gene(s) PMM1678;
1611841	+	TSS_016260	1000	112	0	19 IP	within gene(s) PMM1678; 92nt upstream of gene PMM1679;
1612164	+	TSS_016263	1000	107	0	0 I	within gene(s) PMM1679;
1616290	+	TSS_016278	1000	422	0	0 Ai	antisense to gene(s) PMM1682;
1616799	-	TSS_038121	1000	105	0	15 I	within gene(s) PMM1682;
1616930	-	TSS_038126	1000	884	0	6 P	26nt upstream of gene PMM1682;
1617422	-	TSS_038135	1000	624	0	9 P	93nt upstream of gene PMM1683;
1618516	+	TSS_016291	1000	285	0	0 I	within gene(s) PMM1685;
1618595	+	TSS_016293	1000	189	0	3 I	within gene(s) PMM1685;
1619698	-	TSS_038141	1000	282	0	0 Ai	antisense to gene(s) PMM1686;
1619840	-	TSS_038142	1000	178	0	0 Ai	antisense to gene(s) PMM1686;
1620131	+	TSS_016299	1000	614	0	4 P	24nt upstream of gene PMM1687;
1621301	+	TSS_016304	1000	459	0	4 P	25nt upstream of gene PMM1688;
1621782	+	TSS_016305	1000	121	0	1 I	within gene(s) PMM1688;
1622476	-	TSS_038147	1000	930	0	6 Ai	antisense to gene(s) PMM1688;
1623293	+	TSS_016319	1000	410	0	2 I	within gene(s) PMM1689;
1624058	+	TSS_016338	1000	128	0	21 I	within gene(s) PMM1689;
1624375	+	TSS_016354	1000	214	0	8 I	within gene(s) PMM1689;
1624911	-	TSS_038154	1000	129	0	0 Ai	antisense to gene(s) PMM1690;
1625074	-	TSS_038155	1000	273	0	0 Ai	antisense to gene(s) PMM1690;
1628185	-	TSS_038160	1000	224	0	5 Ai	antisense to gene(s) PMM1693;
1628843	-	TSS_038163	1000	108	0	1 I	within gene(s) PMM1694;
1628980	+	TSS_016374	1000	506	0	2 PAi	173nt upstream of gene PMM1695; antisense to gene(s) PMM1694;
1629107	-	TSS_038166	1000	221	0	0 P	0nt upstream of gene PMM1694;
1629235	+	TSS_016376	1000	180	0	1 I	within gene(s) PMM1695;
1630728	+	TSS_016382	1000	199	0	1 I	within gene(s) PMM1696;
1632609	+	TSS_016386	1000	215	0	1 P	98nt upstream of gene PMM1697;
1632963	-	TSS_038178	1000	131	0	1 Ai	antisense to gene(s) PMM1697;
1633015	+	TSS_016398	1000	121	0	4 I	within gene(s) PMM1697;
1633040	+	TSS_016401	1000	192	0	0 I	within gene(s) PMM1697;
1633252	-	TSS_038182	1000	322	0	1 Ai	antisense to gene(s) PMM1697;
1633316	+	TSS_016418	1000	253	0	12 I	within gene(s) PMM1697;
1633397	+	TSS_016424	1000	101	0	24 I	within gene(s) PMM1697;
1633425	-	TSS_038186	1000	163	0	1 Ai	antisense to gene(s) PMM1697;
1634577	+	TSS_016440	1000	300	0	0 Ai	antisense to gene(s) PMM1699;
1634772	+	TSS_016442	1000	218	0	0 Ai	antisense to gene(s) PMM1699;
1635344	+	TSS_016443	1000	215	0	2 PAi	199nt upstream of gene PMM1700; antisense to gene(s) PMM1699;
1635663	+	TSS_016449	1000	116	0	27 I	within gene(s) PMM1700;

1635705	+	TSS_016456	1000	120	0	8	I	within gene(s) PMM1700;
1636128	+	TSS_016489	1000	140	0	21	I	within gene(s) PMM1700;
1636519	+	TSS_016527	1000	113	0	15	I	within gene(s) PMM1700;
1636725	+	TSS_016549	1000	235	0	21	I	within gene(s) PMM1700;
1636950	+	TSS_016570	1000	134	0	45	I	within gene(s) PMM1700;
1637052	+	TSS_016594	1000	135	0	6	I	within gene(s) PMM1700;
1639698	-	TSS_038209	1000	125	0	0	I	within gene(s) PMM1702;
1640380	-	TSS_038211	1000	343	0	0	P	32nt upstream of gene PMM1702;
1640445	+	TSS_016645	1000	1216	0	1	I	within gene(s) PMM1703;
1641448	+	TSS_016649	1000	383	0	0	IAd	within gene(s) PMM1703; antisense to gene(s) PMM1704 (29nt downstream);
1641526	-	TSS_038216	1000	116	0	3	I	within gene(s) PMM1704;
1641562	-	TSS_038219	1000	136	0	21	I	within gene(s) PMM1704;
1641604	-	TSS_038230	1000	409	0	63	I	within gene(s) PMM1704;
1641664	-	TSS_038246	1000	160	0	12	I	within gene(s) PMM1704;
1641724	-	TSS_038266	1000	1013	0	69	I	within gene(s) PMM1704;
1641750	-	TSS_038273	1000	121	0	0	I	within gene(s) PMM1704;
1641787	-	TSS_038275	1000	160	0	6	I	within gene(s) PMM1704;
1641808	-	TSS_038277	1000	208	0	0	I	within gene(s) PMM1704;
1641841	-	TSS_038282	1000	269	0	27	I	within gene(s) PMM1704;
1641889	-	TSS_038291	1000	154	0	24	I	within gene(s) PMM1704;
1641937	-	TSS_038300	1000	507	0	49	I	within gene(s) PMM1704;
1642006	-	TSS_038314	1000	426	0	21	I	within gene(s) PMM1704;
1642036	-	TSS_038322	1000	131	0	3	I	within gene(s) PMM1704;
1642045	-	TSS_038323	1000	239	0	15	I	within gene(s) PMM1704;
1642050	+	TSS_016654	1000	325	0	0	Ai	antisense to gene(s) PMM1704;
1642075	-	TSS_038330	1000	270	0	24	I	within gene(s) PMM1704;
1642108	-	TSS_038336	1000	281	0	30	I	within gene(s) PMM1704;
1642147	-	TSS_038346	1000	109	5.10E-14	0	I	within gene(s) PMM1704;
1642183	-	TSS_038347	1000	133	0	0	I	within gene(s) PMM1704;
1642205	-	TSS_038352	1000	300	0	33	I	within gene(s) PMM1704;
1642229	+	TSS_016659	1000	270	0	2	Ai	antisense to gene(s) PMM1704;
1642258	-	TSS_038363	1000	265	0	24	I	within gene(s) PMM1704;
1642285	-	TSS_038368	1000	857	0	66	I	within gene(s) PMM1704;
1642301	+	TSS_016661	1000	110	0	0	Ai	antisense to gene(s) PMM1704;
1642348	-	TSS_038388	1000	429	0	0	I	within gene(s) PMM1704;
1642357	-	TSS_038389	1000	109	3.90E-08	0	I	within gene(s) PMM1704;
1642378	-	TSS_038391	1000	262	0	3	I	within gene(s) PMM1704;
1642387	-	TSS_038392	1000	155	0	0	I	within gene(s) PMM1704;
1642402	-	TSS_038393	1000	196	0	0	I	within gene(s) PMM1704;
1642411	-	TSS_038394	1000	113	7.20E-10	6	I	within gene(s) PMM1704;
1642444	-	TSS_038403	1000	931	0	37	I	within gene(s) PMM1704;
1642477	-	TSS_038412	1000	539	0	27	I	within gene(s) PMM1704;
1642522	-	TSS_038425	1000	154	0	12	I	within gene(s) PMM1704;
1642564	-	TSS_038429	1000	134	0	6	I	within gene(s) PMM1704;
1642606	-	TSS_038433	1000	863	0	16	I	within gene(s) PMM1704;
1642639	-	TSS_038442	1000	269	0	18	I	within gene(s) PMM1704;
1642666	-	TSS_038447	1000	130	0	9	I	within gene(s) PMM1704;
1642678	-	TSS_038449	1000	115	0	6	I	within gene(s) PMM1704;
1642717	-	TSS_038451	1000	114	0	0	I	within gene(s) PMM1704;
1642759	-	TSS_038461	1000	369	0	33	I	within gene(s) PMM1704;
1642771	-	TSS_038462	1000	471	0	63	I	within gene(s) PMM1704;
1642810	+	TSS_016670	1000	205	0	0	Ai	antisense to gene(s) PMM1704;
1642846	-	TSS_038484	1000	281	0	36	I	within gene(s) PMM1704;
1642900	-	TSS_038495	1000	679	0	45	I	within gene(s) PMM1704;
1642957	-	TSS_038510	1000	296	0	3	I	within gene(s) PMM1704;
1642975	-	TSS_038512	1000	170	0	18	I	within gene(s) PMM1704;
1643011	-	TSS_038517	1000	217	0	6	I	within gene(s) PMM1704;
1643053	-	TSS_038525	1000	191	0	24	I	within gene(s) PMM1704;
1643137	-	TSS_038529	1000	377	0	0	I	within gene(s) PMM1704;
1643144	+	TSS_016673	1000	450	0	5	Ai	antisense to gene(s) PMM1704;
1643146	-	TSS_038530	1000	117	0	0	I	within gene(s) PMM1704;
1643153	+	TSS_016675	1000	444	0	1	Ai	antisense to gene(s) PMM1704;
1643179	-	TSS_038542	1000	384	0	25	I	within gene(s) PMM1704;
1643197	-	TSS_038547	1000	289	0	9	I	within gene(s) PMM1704;
1643215	-	TSS_038548	1000	148	0	6	I	within gene(s) PMM1704;
1643239	-	TSS_038553	1000	431	0	18	I	within gene(s) PMM1704;
1643311	-	TSS_038557	1000	254	0	9	I	within gene(s) PMM1704;
1643316	+	TSS_016677	1000	193	0	0	PAi	182nt upstream of gene PMM1705; antisense to gene(s) PMM1704;
1643335	-	TSS_038560	1000	111	0	0	I	within gene(s) PMM1704;
1643350	-	TSS_038562	1000	111	0	6	I	within gene(s) PMM1704;
1643359	-	TSS_038563	1000	162	0	0	I	within gene(s) PMM1704;
1643399	-	TSS_038567	1000	14277	0	7	P	19nt upstream of gene PMM1704;
1644415	+	TSS_016679	1000	1741	0	1	P	50nt upstream of gene PMM1706;
1644804	+	TSS_016696	1000	133	0	1	I	within gene(s) PMM1706;
1645357	-	TSS_038579	1000	234	0	2	I	within gene(s) PMM1707;
1645691	-	TSS_038594	1000	173	0	6	I	within gene(s) PMM1707;
1646195	-	TSS_038610	1000	1080	0	6	P	48nt upstream of gene PMM1707;

1646216	-	TSS_038613	1000	3722	0	0	P	69nt upstream of gene PMM1707;
1646481	+	TSS_016716	1000	138	0	0	IP	within gene(s) PMM1708; 217nt upstream of gene PMM1709;
1646490	+	TSS_016717	1000	209	0	0	IP	within gene(s) PMM1708; 208nt upstream of gene PMM1709;
1646517	+	TSS_016722	1000	186	0	10	IP	within gene(s) PMM1708; 181nt upstream of gene PMM1709;
1646542	+	TSS_016727	1000	166	0	9	IP	within gene(s) PMM1708; 156nt upstream of gene PMM1709;
1647512	-	TSS_038618	1000	148	0	0	Ai	antisense to gene(s) PMM1709;
1647767	+	TSS_016737	1000	123	0	3	IP	within gene(s) PMM1709; 21nt upstream of gene PMM1710;
1654524	+	TSS_016764	1000	273	0	4	I	within gene(s) PMM1714;
1656926	+	TSS_016775	1000	192	0	6	I	within gene(s) PMM1716;
1657573	-	TSS_038666	1000	101	0	0	Ai	antisense to gene(s) PMM1716;

**Class represents the classification of the start site. I represents internal start sites, Ai represents antisense start sites, P represents primary start sites,
IP represents internal or primary start sites, PAi represents primary or Antisense start sites, Ad represnts Antisense or downstream
IAd represents internal Antisense or downstream and O represents orphan.
