# Supplementary material for: Nitrogen cost minimization is promoted by structural changes in the transcriptome of N-deprived Prochlorococcus cells
Source: ISME J. 2017 Jun 6;11(10):2267–78. doi: 10.1038/ismej.2017.88 (PMC5607370; doi:10.1038/ismej.2017.88)
Supplement: Supplementary Table 12 [file ismej201788x19.pdf]

Table S12.Internal Start Site Comparison of This Study to Voigt et al. (2014). Internal TSSs identified by TSSAR were directly compared to Voigt et al. to determine presence or absence in their study. Genes in bold did not have an internal TSS in their study.

Identity	Internal TSS Identified by Voigt
PMM0003	Yes
PMM0004	Yes
**PMM0007**	**No**
PMM0010	Yes
**PMM0012**	**No**
PMM0022	Yes
PMM0024	Yes
PMM0025	Yes
PMM0026	Yes
**PMM0027**	**No**
**PMM0030**	**No**
PMM0032	Yes
**PMM0034**	**No**
**PMM0035**	**No**
PMM0036	Yes
PMM0037	Yes
PMM0038	Yes
**PMM0042**	**No**
PMM0044	Yes
**PMM0045**	**No**
**PMM0047**	**No**
**PMM0051**	**No**
**PMM0055**	**No**
**PMM0058**	**No**
PMM0060	Yes
**PMM0063**	**No**
PMM0065	Yes
**PMM0073**	**No**
**PMM0075**	**No**
**PMM0077**	**No**
**PMM0081**	**No**
PMM0083	Yes
PMM0084	Yes
**PMM0085**	**No**
**PMM0086**	**No**
**PMM0087**	**No**
PMM0089	Yes
**PMM0091**	**No**
PMM0095	Yes
**PMM0099**	**No**
PMM0100	Yes
PMM0101	Yes
**PMM0103**	**No**
**PMM0117**	**No**
PMM0121	Yes
**PMM0123**	**No**
**PMM0126**	**No**
**PMM0128**	**No**
**PMM0129**	**No**
**PMM0131**	**No**
PMM0133	Yes
**PMM0134**	**No**
**PMM0135**	**No**
PMM0138	Yes
**PMM0143**	**No**
**PMM0144**	**No**
**PMM0147**	**No**
**PMM0148**	**No**
**PMM0149**	**No**
**PMM0150**	**No**
**PMM0152**	**No**
**PMM0153**	**No**
PMM0156	Yes
**PMM0159**	**No**
**PMM0160**	**No**
PMM0163	Yes
**PMM0164**	**No**
**PMM0169**	**No**
PMM0170	Yes
**PMM0172**	**No**
**PMM0185**	**No**
PMM0187	Yes
**PMM0191**	**No**
PMM0192	Yes
PMM0195	Yes
**PMM0201**	**No**
**PMM0202**	**No**
**PMM0203**	**No**
**PMM0205**	**No**
PMM0207	Yes
PMM0209	Yes
PMM0211	Yes
PMM0214	Yes
PMM0217	Yes
**PMM0222**	**No**
**PMM0223**	**No**
**PMM0224**	**No**
PMM0226	Yes
PMM0227	Yes
**PMM0228**	**No**
**PMM0233**	**No**
**PMM0235**	**No**
**PMM0238**	**No**
**PMM0244**	**No**
PMM0256	Yes
PMM0258	No
PMM0263	Yes
**PMM0264**	**No**
**PMM0265**	**No**
**PMM0268**	**No**
PMM0276	Yes
PMM0279	Yes
PMM0284	Yes
PMM0285	Yes
**PMM0293**	**No**
PMM0296	Yes
PMM0304	Yes

**PMM0312**	**No**
**PMM0315**	**No**
PMM0320	Yes
PMM0321	No
PMM0324	Yes
**PMM0325**	**No**
**PMM0326**	**No**
PMM0333	Yes
**PMM0335**	**No**
PMM0339	Yes
**PMM0346**	**No**
**PMM0356**	**No**
PMM0358	Yes
**PMM0366**	**No**
**PMM0368**	**No**
**PMM0370**	**No**
PMM0386	Yes
**PMM0390**	**No**
**PMM0397**	**No**
PMM0398	Yes
**PMM0405**	**No**
**PMM0406**	**No**
**PMM0407**	**No**
**PMM0420**	**No**
PMM0425	Yes
**PMM0426**	**No**
PMM0430	Yes
PMM0434	Yes
PMM0438	Yes
**PMM0445**	**No**
**PMM0446**	**No**
**PMM0448**	**No**
PMM0451	Yes
PMM0452	Yes
PMM0454	Yes
**PMM0461**	**No**
**PMM0462**	**No**
**PMM0472**	**No**
**PMM0473**	**No**
**PMM0477**	**No**
**PMM0482**	**No**
**PMM0483**	**No**
PMM0493	Yes
PMM0494	Yes
PMM0495	Yes
PMM0500	Yes
PMM0503	Yes
**PMM0513**	**No**
PMM0516	Yes
**PMM0518**	**No**
**PMM0519**	**No**
PMM0520	Yes
**PMM0521**	**No**
**PMM0522**	**No**
PMM0525	Yes
PMM0526	Yes
PMM0533	Yes
**PMM0534**	**No**
**PMM0537**	**No**
PMM0544	Yes
PMM0545	Yes
PMM0546	Yes
**PMM0550**	**No**
PMM0552	Yes
PMM0560	Yes
PMM0561	Yes
**PMM0565**	**No**
**PMM0577**	**No**
PMM0580	Yes
PMM0582	Yes
PMM0585	Yes
**PMM0590**	**No**
PMM0593	Yes
**PMM0594**	**No**
PMM0596	Yes
PMM0601	Yes
PMM0602	Yes
**PMM0604**	**No**
**PMM0608**	**No**
PMM0609	Yes
**PMM0611**	**No**
PMM0613	Yes
PMM0615	Yes
**PMM0618**	**No**
**PMM0619**	**No**
**PMM0622**	**No**
PMM0623	Yes
PMM0633	Yes
PMM0640	Yes
**PMM0642**	**No**
**PMM0644**	**No**
PMM0658	Yes
**PMM0659**	**No**
**PMM0660**	**No**
**PMM0661**	**No**
**PMM0676**	**No**
PMM0681	Yes
**PMM0683**	**No**
PMM0684	Yes
PMM0687	Yes
PMM0693	Yes
**PMM0694**	**No**
**PMM0698**	**No**
PMM0704	Yes
**PMM0707**	**No**
PMM0708	Yes
**PMM0709**	**No**
**PMM0710**	**No**
**PMM0725**	**No**

PMM0742	Yes
**PMM0743**	**No**
**PMM0749**	**No**
PMM0750	Yes
**PMM0753**	**No**
PMM0754	Yes
PMM0756	Yes
PMM0757	Yes
**PMM0758**	**No**
**PMM0760**	**No**
**PMM0766**	**No**
**PMM0767**	**No**
**PMM0770**	**No**
**PMM0774**	**No**
**PMM0780**	**No**
**PMM0784**	**No**
**PMM0785**	**No**
PMM0787	Yes
PMM0796	Yes
**PMM0804**	**No**
**PMM0815**	**No**
**PMM0819**	**No**
PMM0821	Yes
PMM0825	Yes
**PMM0829**	**No**
**PMM0831**	**No**
PMM0832	Yes
**PMM0842**	**No**
**PMM0844**	**No**
**PMM0853**	**No**
**PMM0858**	**No**
**PMM0863**	**No**
PMM0872	Yes
PMM0877	Yes
PMM0880	Yes
PMM0881	Yes
PMM0893	Yes
PMM0894	Yes
**PMM0896**	**No**
**PMM0897**	**No**
PMM0899	Yes
PMM0901	Yes
**PMM0906**	**No**
**PMM0907**	**No**
**PMM0908**	**No**
**PMM0912**	**No**
**PMM0913**	**No**
PMM0916	Yes
**PMM0941**	**No**
PMM0945	Yes
PMM0946	Yes
PMM0954	Yes
**PMM0955**	**No**
**PMM0961**	**No**
**PMM0965**	**No**
PMM0966	Yes
**PMM0970**	**No**
**PMM0974**	**No**
**PMM0975**	**No**
**PMM1009**	**No**
**PMM1022**	**No**
PMM1033	Yes
**PMM1033**	**No**
PMM1038	Yes
PMM1039	Yes
**PMM1040**	**No**
**PMM1041**	**No**
PMM1042	Yes
PMM1055	Yes
**PMM1057**	**No**
PMM1062	Yes
PMM1064	Yes
PMM1066	Yes
PMM1067	Yes
PMM1075	Yes
PMM1077	Yes
PMM1080	Yes
**PMM1081**	**No**
**PMM1088**	**No**
PMM1090	Yes
**PMM1091**	**No**
PMM1092	Yes
**PMM1093**	**No**
PMM1096	Yes
**PMM1113**	**No**
PMM1119	Yes
PMM1121	Yes
**PMM1123**	**No**
PMM1124	Yes
**PMM1127**	**No**
**PMM1132**	**No**
**PMM1139**	**No**
**PMM1140**	**No**
**PMM1142**	**No**
PMM1145	Yes
**PMM1157**	**No**
**PMM1158**	**No**
PMM1174	Yes
PMM1176	Yes
**PMM1178**	**No**
**PMM1186**	**No**
PMM1188	Yes
**PMM1191**	**No**
PMM1192	Yes
**PMM1196**	**No**
PMM1199	Yes
**PMM1202**	**No**
**PMM1204**	**No**
**PMM1205**	**No**

**PMM1225**	**No**
**PMM1232**	**No**
PMM1234	Yes
PMM1235	Yes
PMM1249	Yes
PMM1250	Yes
PMM1256	Yes
PMM1258	Yes
**PMM1259**	**No**
PMM1260	Yes
PMM1261	Yes
**PMM1264**	**No**
**PMM1269**	**No**
**PMM1270**	**No**
**PMM1280**	**No**
PMM1287	Yes
**PMM1288**	**No**
PMM1289	Yes
PMM1290	Yes
**PMM1293**	**No**
PMM1299	Yes
**PMM1300**	**No**
PMM1301	Yes
PMM1306	Yes
PMM1310	Yes
PMM1311	Yes
PMM1312	Yes
PMM1318	Yes
**PMM1322**	**No**
PMM1323	Yes
**PMM1324**	**No**
**PMM1332**	**No**
PMM1333	Yes
**PMM1335**	**No**
**PMM1338**	**No**
PMM1339	Yes
PMM1340	Yes
PMM1341	Yes
**PMM1342**	**No**
PMM1349	Yes
**PMM1350**	**No**
**PMM1360**	**No**
**PMM1383**	**No**
PMM1394	Yes
**PMM1396**	**No**
PMM1400	Yes
PMM1415	Yes
PMM1416	Yes
**PMM1422**	**No**
**PMM1428**	**No**
PMM1432	Yes
**PMM1434**	**No**
**PMM1435**	**No**
**PMM1436**	**No**
**PMM1443**	**No**
PMM1449	Yes
PMM1458	Yes
**PMM1465**	**No**
PMM1467	Yes
**PMM1476**	**No**
**PMM1483**	**No**
PMM1484	Yes
PMM1485	Yes
PMM1492	Yes
PMM1494	Yes
PMM1496	Yes
PMM1499	Yes
PMM1500	Yes
PMM1501	Yes
PMM1508	Yes
PMM1509	Yes
**PMM1512**	**No**
**PMM1519**	**No**
**PMM1523**	**No**
**PMM1524**	**No**
PMM1526	Yes
PMM1527	Yes
PMM1533	Yes
**PMM1535**	**No**
**PMM1536**	**No**
PMM1540	Yes
**PMM1542**	**No**
**PMM1555**	**No**
PMM1562	Yes
**PMM1566**	**No**
**PMM1570**	**No**
**PMM1574**	**No**
**PMM1575**	**No**
PMM1577	Yes
PMM1580	Yes
PMM1583	Yes
PMM1585	Yes
PMM1589	Yes
**PMM1594**	**No**
**PMM1595**	**No**
PMM1596	Yes
**PMM1600**	**No**
PMM1605	Yes
**PMM1606**	**No**
PMM1609	Yes
**PMM1615**	**No**
**PMM1619**	**No**
PMM1621	Yes
**PMM1625**	**No**
**PMM1629**	**No**
**PMM1630**	**No**
**PMM1636**	**No**
**PMM1638**	**No**
PMM1639	Yes

PMM1640	Yes
PMM1642	Yes
**PMM1648**	**No**
PMM1650	Yes
PMM1652	Yes
PMM1653	Yes
**PMM1657**	**No**
PMM1660	Yes
**PMM1662**	**No**
PMM1664	Yes
**PMM1665**	**No**
**PMM1667**	**No**
PMM1668	Yes
PMM1669	Yes
PMM1672	Yes
**PMM1674**	**No**
PMM1675	Yes
PMM1676	Yes
**PMM1679**	**No**
PMM1684	Yes
PMM1685	Yes
PMM1688	Yes
**PMM1689**	**No**
**PMM1694**	**No**
PMM1695	Yes
PMM1696	Yes
PMM1697	Yes
PMM1700	Yes
PMM1702	Yes
PMM1703	Yes
**PMM1704**	**No**
**PMM1706**	**No**
**PMM1707**	**No**
**PMM1711**	**No**
PMM1712	Yes
**PMM1714**	**No**
**PMM1716**	**No**
