# Supplementary material for: Nitrogen cost minimization is promoted by structural changes in the transcriptome of N-deprived Prochlorococcus cells
Source: ISME J. 2017 Jun 6;11(10):2267–78. doi: 10.1038/ismej.2017.88 (PMC5607370; doi:10.1038/ismej.2017.88)
Supplement: Supplementary Table 13 [file ismej201788x20.pdf]

Table S13. Ratio of internal to primary start site utilization based on read mappings and TSSAR. All results are for TSSAR identified internal start sites with a p value of 0. ID column matches to results found in Tables S8-S9

Position	Strand	ID	Identity	Primary Position	Ratio of Internal To Primary Reads	Type
1291478	+	TSS_007213	PMM1340	1290486	12.93	Control
730435	+	TSS_004333	PMM0767	730331	11.79	Control
1068999	-	TSS_016277	PMM1121	1069447	6.82	Control
1380019	+	TSS_007743	PMM1443	1379949	5.57	Control
1448025	-	TSS_019253	PMM1509	1449812	5.51	Control
860406	-	TSS_015192	PMM0897	860982	4.84	Control
32374	+	TSS_000209	PMM0034	32317	4.35	Control
387042	-	TSS_012107	PMM0407	387181	3.93	Control
140081	-	TSS_010302	PMM0143	140446	3.64	Control
324964	+	TSS_002154	PMM0335	324585	3.59	Control
1633040	+	TSS_009535	PMM1697	1632609	3.48	Control
1219165	-	TSS_016932	PMM1264	1219801	3.09	Control
98015	-	TSS_010136	PMM0095	98676	2.87	Control
763063	+	TSS_004496	PMM0804	762992	2.49	Control
590072	+	TSS_003690	PMM0619	589671	2.47	Control
467553	+	TSS_002893	PMM0494	467409	2.25	Control
468497	-	TSS_012700	PMM0495	469000	2.13	Control
1218465	-	TSS_016879	PMM1264	1219801	1.96	Control
258478	-	TSS_011103	PMM0268	258613	1.89	Control
223706	-	TSS_010918	PMM0228	223847	1.85	Control
582602	-	TSS_013632	PMM0611	582767	1.80	Control
1024480	+	TSS_005505	PMM1081	1023698	1.71	Control
1568018	-	TSS_021542	PMM1639	1569177	1.68	Control
154964	-	TSS_010404	PMM0159	155061	1.65	Control
467736	+	TSS_002903	PMM0494	467409	1.48	Control
466192	+	TSS_002884	PMM0493	465840	1.47	Control
558490	+	TSS_003542	PMM0590	558189	1.38	Control
328681	-	TSS_011822	PMM0339	329348	1.35	Control
1000995	-	TSS_015707	PMM1062	1002034	1.33	Control
1557486	-	TSS_021342	PMM1629	1558291	1.31	Control
1363264	-	TSS_017821	PMM1422	1363417	1.24	Control
121450	-	TSS_010237	PMM0123	121764	1.21	Control
123779	+	TSS_000612	PMM0126	123251	1.18	Control
1001916	-	TSS_015710	PMM1062	1002034	1.15	Control
1252148	+	TSS_006906	PMM1300	1251088	1.15	Control
150290	+	TSS_000819	PMM0152	149427	1.10	Control
1454543	+	TSS_008337	PMM1512	1450891	1.08	Control
66518	+	TSS_000351	PMM0060	65752	1.04	Control
1469172	+	TSS_008462	PMM1526	1468704	1.00	Control
730737	+	TSS_004340	PMM0767	730331	0.92	Control
223299	-	TSS_010889	PMM0228	223847	0.92	Control
1525299	+	TSS_008863	PMM1596	1524215	0.85	Control
900053	+	TSS_005025	PMM0941	899977	0.85	Control
1245796	+	TSS_006866	PMM1293	1245509	0.84	Control
1469928	-	TSS_019961	PMM1527	1470923	0.84	Control
66151	+	TSS_000349	PMM0060	65752	0.81	Control
151351	-	TSS_010369	PMM0153	151546	0.78	Control
151109	-	TSS_010365	PMM0153	151546	0.78	Control
614082	-	TSS_013745	PMM0644	614781	0.76	Control
704459	-	TSS_014101	PMM0743	705815	0.75	Control
143488	-	TSS_010323	PMM0147	143883	0.71	Control
1454166	+	TSS_008330	PMM1512	1450891	0.71	Control
1036076	+	TSS_005570	PMM1090	1035252	0.68	Control
1019222	-	TSS_015819	PMM1075	1019352	0.66	Control
1454745	+	TSS_008339	PMM1512	1450891	0.65	Control
1567306	-	TSS_021525	PMM1639	1569177	0.64	Control
105437	-	TSS_010164	PMM0101	105946	0.63	Control
78158	-	TSS_010048	PMM0073	79154	0.62	Control
1018655	-	TSS_015770	PMM1075	1019352	0.62	Control
730925	+	TSS_004346	PMM0767	730331	0.61	Control
223578	-	TSS_010913	PMM0228	223847	0.60	Control
628505	-	TSS_013812	PMM0660	628656	0.60	Control
499089	+	TSS_003081	PMM0526	497550	0.60	Control
1300005	+	TSS_007266	PMM1349	1299756	0.59	Control
784835	+	TSS_004587	PMM0829	784601	0.56	Control
1332932	-	TSS_017653	PMM1383	1333562	0.56	Control
309678	-	TSS_011589	PMM0324	309757	0.55	Control
348397	+	TSS_002235	PMM0366	348206	0.54	Control
1499500	-	TSS_020778	PMM1570	1499641	0.54	Control
1059858	+	TSS_005663	PMM1113	1059299	0.52	Control
746288	-	TSS_014510	PMM0784	746584	0.51	Control
66815	+	TSS_000353	PMM0060	65752	0.51	Control
12361	+	TSS_000061	PMM0010	11776	0.50	Control
433897	+	TSS_002683	PMM0454	433437	0.47	Control
63461	+	TSS_000341	PMM0058	63116	0.46	Control
1639698	-	TSS_021915	PMM1702	1640380	0.45	Control
1490650	+	TSS_008712	PMM1562	1490373	0.44	Control
189007	+	TSS_000955	PMM0195	188865	0.44	Control
1252018	+	TSS_006904	PMM1300	1251088	0.43	Control
1589048	+	TSS_009308	PMM1657	1588301	0.42	Control
1554498	-	TSS_021285	PMM1625	1554856	0.41	Control
723763	-	TSS_014249	PMM0760	724578	0.40	Control
1245700	+	TSS_006865	PMM1293	1245509	0.40	Control
614001	-	TSS_013743	PMM0644	614781	0.40	Control
1518815	-	TSS_020898	PMM1589	1519244	0.39	Control
78218	-	TSS_010050	PMM0073	79154	0.39	Control
705461	-	TSS_014138	PMM0743	705815	0.39	Control
15347	+	TSS_000080	PMM0012	14568	0.39	Control
1597137	+	TSS_009358	PMM1665	1596577	0.38	Control
504905	+	TSS_003122	PMM0533	504428	0.38	Control
27367	+	TSS_000180	PMM0026	27273	0.37	Control

644591	+	TSS_003974	PMM0676	643669	0.37	Control
197771	-	TSS_010596	PMM0205	198069	0.37	Control
197876	-	TSS_010601	PMM0205	198069	0.36	Control
129278	-	TSS_010273	PMM0131	130071	0.33	Control
1623293	+	TSS_009503	PMM1689	1623168	0.32	Control
729981	-	TSS_014324	PMM0766	730261	0.31	Control
78481	-	TSS_010056	PMM0073	79154	0.31	Control
493996	-	TSS_012831	PMM0522	494177	0.31	Control
1224306	-	TSS_017006	PMM1270	1225100	0.29	Control
105798	-	TSS_010171	PMM0101	105946	0.28	Control
1095730	+	TSS_005822	PMM1142	1094754	0.27	Control
588874	+	TSS_003680	PMM0618	588622	0.26	Control
466610	+	TSS_002887	PMM0493	465840	0.25	Control
78295	-	TSS_010052	PMM0073	79154	0.24	Control
189333	+	TSS_000960	PMM0195	188865	0.24	Control
738964	-	TSS_014383	PMM0774	739095	0.24	Control
144965	+	TSS_000739	PMM0149	144701	0.23	Control
737785	-	TSS_014366	PMM0774	739095	0.22	Control
441185	-	TSS_012492	PMM0462	441340	0.22	Control
105240	-	TSS_010162	PMM0101	105946	0.22	Control
349634	-	TSS_011872	PMM0368	349709	0.21	Control
145715	+	TSS_000765	PMM0149	144701	0.21	Control
398719	+	TSS_002519	PMM0420	398147	0.21	Control
1373532	+	TSS_007576	PMM1435	1373308	0.21	Control
145511	+	TSS_000754	PMM0149	144701	0.20	Control
703511	+	TSS_004164	PMM0742	703275	0.20	Control
738854	-	TSS_014379	PMM0774	739095	0.20	Control
218599	+	TSS_001138	PMM0224	217978	0.19	Control
1275591	+	TSS_007141	PMM1323	1274764	0.19	Control
145211	+	TSS_000745	PMM0149	144701	0.17	Control
864270	+	TSS_004816	PMM0901	863809	0.17	Control
1238666	-	TSS_017213	PMM1287	1238943	0.16	Control
1549235	-	TSS_021156	PMM1619	1549798	0.16	Control
1422856	-	TSS_019005	PMM1485	1423524	0.16	Control
1644804	+	TSS_009619	PMM1706	1644415	0.16	Control
218695	+	TSS_001143	PMM0224	217978	0.16	Control
143021	-	TSS_010320	PMM0147	143883	0.15	Control
1624375	+	TSS_009510	PMM1689	1623168	0.15	Control
125778	+	TSS_000632	PMM0128	125323	0.15	Control
1131719	+	TSS_006311	PMM1186	1131150	0.14	Control
1401712	-	TSS_018480	PMM1467	1403187	0.14	Control
220578	-	TSS_010821	PMM0226	221632	0.14	Control
863967	+	TSS_004806	PMM0901	863809	0.14	Control
1422752	-	TSS_019004	PMM1485	1423524	0.13	Control
611411	+	TSS_003813	PMM0642	610941	0.13	Control
498247	+	TSS_003041	PMM0526	497550	0.13	Control
1423046	-	TSS_019006	PMM1485	1423524	0.13	Control
253836	+	TSS_001391	PMM0263	252586	0.13	Control
498643	+	TSS_003048	PMM0526	497550	0.12	Control
160552	-	TSS_010442	PMM0164	160684	0.12	Control
497254	+	TSS_003020	PMM0525	496211	0.12	Control
803506	-	TSS_014887	PMM0844	804298	0.12	Control
1253102	+	TSS_006915	PMM1301	1252970	0.12	Control
132594	+	TSS_000675	PMM0134	132109	0.11	Control
1034423	-	TSS_016144	PMM1088	1034569	0.11	Control
205032	+	TSS_001030	PMM0211	204910	0.11	Control
1372986	+	TSS_007570	PMM1434	1371646	0.11	Control
1529501	-	TSS_020964	PMM1600	1530254	0.11	Control
31393	-	TSS_009806	PMM0032	31536	0.10	Control
1022762	-	TSS_015837	PMM1080	1023493	0.10	Control
1240490	-	TSS_017232	PMM1289	1241067	0.10	Control
747387	-	TSS_014538	PMM0785	747664	0.10	Control
384162	+	TSS_002423	PMM0405	383585	0.10	Control
1346497	-	TSS_017730	PMM1400	1346865	0.09	Control
145139	+	TSS_000744	PMM0149	144701	0.09	Control
1464704	-	TSS_019746	PMM1524	1466574	0.09	Control
447456	+	TSS_002741	PMM0472	447381	0.09	Control
27552	+	TSS_000183	PMM0026	27273	0.09	Control
92216	-	TSS_010120	PMM0086	92293	0.08	Control
724438	-	TSS_014296	PMM0760	724578	0.08	Control
857485	-	TSS_015161	PMM0894	857549	0.08	Control
1008963	+	TSS_005417	PMM1066	1007850	0.07	Control
221154	-	TSS_010854	PMM0226	221632	0.07	Control
299950	-	TSS_011379	PMM0312	300325	0.07	Control
1642444	-	TSS_022043	PMM1704	1643399	0.06	Control
208811	-	TSS_010711	PMM0214	208944	0.06	Control
384720	+	TSS_002453	PMM0405	383585	0.06	Control
384519	+	TSS_002440	PMM0405	383585	0.06	Control
1645357	-	TSS_022164	PMM1707	1646216	0.06	Control
1465661	-	TSS_019863	PMM1524	1466574	0.06	Control
457755	-	TSS_012661	PMM0483	457975	0.06	Control
299836	-	TSS_011375	PMM0312	300325	0.06	Control
119905	+	TSS_000600	PMM0121	119798	0.05	Control
1223572	-	TSS_016991	PMM1269	1224017	0.05	Control
208529	-	TSS_010696	PMM0214	208944	0.05	Control
221070	-	TSS_010850	PMM0226	221632	0.05	Control
166516	+	TSS_000889	PMM0172	165910	0.05	Control
207728	-	TSS_010664	PMM0214	208944	0.04	Control
310880	+	TSS_001841	PMM0326	310480	0.04	Control
1642285	-	TSS_022020	PMM1704	1643399	0.04	Control
147332	+	TSS_000792	PMM0150	146794	0.04	Control
1083716	+	TSS_005789	PMM1132	1082218	0.04	Control
1240970	-	TSS_017248	PMM1289	1241067	0.04	Control

1549500	-	TSS_021169	PMM1619	1549798	0.04	Control
431182	-	TSS_012320	PMM0452	432317	0.04	Control
432210	-	TSS_012388	PMM0452	432317	0.04	Control
147807	+	TSS_000799	PMM0150	146794	0.04	Control
310023	+	TSS_001792	PMM0325	309786	0.03	Control
868162	-	TSS_015227	PMM0907	869447	0.03	Control
1642606	-	TSS_022059	PMM1704	1643399	0.03	Control
1131989	+	TSS_006317	PMM1186	1131150	0.03	Control
195809	-	TSS_010551	PMM0202	196137	0.03	Control
676283	+	TSS_004102	PMM0710	675828	0.03	Control
927406	+	TSS_005145	PMM0970	926558	0.03	Control
927259	+	TSS_005142	PMM0970	926558	0.02	Control
926646	+	TSS_005123	PMM0970	926558	0.02	Control
1138412	+	TSS_006439	PMM1191	1136852	0.01	Control
1068972	-	TSS_022850	PMM1121	1069447	13.01	Experimental
1557493	-	TSS_028288	PMM1629	1558291	10.41	Experimental
900041	+	TSS_007669	PMM0941	899977	10.31	Experimental
468497	-	TSS_018015	PMM0495	469000	9.52	Experimental
1633014	+	TSS_013585	PMM1697	1632609	7.42	Experimental
491260	+	TSS_005334	PMM0519	490706	6.82	Experimental
80289	+	TSS_000660	PMM0075	79823	6.46	Experimental
223706	-	TSS_015742	PMM0228	223847	5.20	Experimental
140081	-	TSS_014893	PMM0143	140446	4.75	Experimental
123779	+	TSS_000953	PMM0126	123251	4.16	Experimental
704397	-	TSS_019669	PMM0743	705815	4.02	Experimental
258478	-	TSS_016059	PMM0268	258616	3.99	Experimental
763062	+	TSS_006882	PMM0804	762992	3.74	Experimental
1568020	-	TSS_028672	PMM1639	1569177	3.61	Experimental
354391	+	TSS_004218	PMM0370	352957	3.31	Experimental
781041	+	TSS_007009	PMM0825	779053	3.08	Experimental
1380027	+	TSS_011134	PMM1443	1379949	2.96	Experimental
353381	+	TSS_004069	PMM0370	352957	2.69	Experimental
12129	+	TSS_000105	PMM0010	11772	2.54	Experimental
1447936	-	TSS_026113	PMM1509	1449812	2.46	Experimental
197771	-	TSS_015365	PMM0205	198069	2.42	Experimental
1018655	-	TSS_021747	PMM1075	1019366	2.41	Experimental
1024480	+	TSS_008664	PMM1081	1023698	2.35	Experimental
176661	+	TSS_001491	PMM0185	176333	2.26	Experimental
1567303	-	TSS_028641	PMM1639	1569177	2.22	Experimental
197876	-	TSS_015373	PMM0205	198069	2.21	Experimental
747387	-	TSS_020214	PMM0785	747664	2.15	Experimental
860406	-	TSS_020949	PMM0897	860980	2.15	Experimental
1589048	+	TSS_013226	PMM1657	1588301	2.07	Experimental
96530	+	TSS_000795	PMM0091	96428	1.94	Experimental
1454166	+	TSS_011834	PMM1512	1450891	1.93	Experimental
154809	-	TSS_015069	PMM0159	155061	1.81	Experimental
154860	-	TSS_015079	PMM0159	155061	1.77	Experimental
981600	+	TSS_008375	PMM1038	981473	1.76	Experimental
1420706	-	TSS_025705	PMM1485	1423524	1.67	Experimental
1363264	-	TSS_024893	PMM1422	1363417	1.57	Experimental
1422856	-	TSS_025770	PMM1485	1423524	1.54	Experimental
1019222	-	TSS_021849	PMM1075	1019366	1.50	Experimental
1454543	+	TSS_011846	PMM1512	1450891	1.49	Experimental
1469172	+	TSS_011941	PMM1526	1468704	1.42	Experimental
628505	-	TSS_019027	PMM0660	628656	1.36	Experimental
1036076	+	TSS_008730	PMM1090	1035252	1.34	Experimental
150290	+	TSS_001294	PMM0152	149428	1.31	Experimental
1126503	-	TSS_023437	PMM1178	1127288	1.29	Experimental
129278	-	TSS_014851	PMM0131	130071	1.27	Experimental
189007	+	TSS_001518	PMM0195	188865	1.27	Experimental
746286	-	TSS_020205	PMM0784	746584	1.26	Experimental
121450	-	TSS_014816	PMM0123	121764	1.16	Experimental
1448485	-	TSS_026157	PMM1509	1449812	1.15	Experimental
1490794	+	TSS_012124	PMM1562	1490373	1.12	Experimental
1294828	-	TSS_024392	PMM1342	1295547	1.09	Experimental
349673	-	TSS_017086	PMM0368	349709	1.07	Experimental
1245700	+	TSS_009975	PMM1293	1245509	1.05	Experimental
774713	-	TSS_020416	PMM0819	774773	1.03	Experimental
433897	+	TSS_004880	PMM0454	433437	1.01	Experimental
143488	-	TSS_014969	PMM0147	143883	1.00	Experimental
63461	+	TSS_000483	PMM0058	63116	0.94	Experimental
105437	-	TSS_014669	PMM0101	105946	0.93	Experimental
128699	-	TSS_014848	PMM0131	130071	0.93	Experimental
1420978	-	TSS_025720	PMM1485	1423524	0.89	Experimental
155927	-	TSS_015116	PMM0160	156249	0.88	Experimental
499089	+	TSS_005510	PMM0526	497550	0.87	Experimental
98015	-	TSS_014647	PMM0095	98676	0.84	Experimental
1518815	-	TSS_027523	PMM1589	1519244	0.84	Experimental
644589	+	TSS_006296	PMM0676	643669	0.84	Experimental
1554498	-	TSS_028202	PMM1625	1554856	0.83	Experimental
1059858	+	TSS_008821	PMM1113	1059299	0.80	Experimental
105223	-	TSS_014665	PMM0101	105946	0.78	Experimental
1122905	+	TSS_009400	PMM1174	1122779	0.78	Experimental
1606665	+	TSS_013382	PMM1674	1606251	0.76	Experimental
1448025	-	TSS_026120	PMM1509	1449812	0.76	Experimental
1628843	-	TSS_029215	PMM1694	1629107	0.74	Experimental
131956	-	TSS_014865	PMM0133	132089	0.72	Experimental
1332932	-	TSS_024612	PMM1383	1333562	0.72	Experimental
644399	+	TSS_006291	PMM0676	643669	0.71	Experimental
723763	-	TSS_019917	PMM0760	724578	0.69	Experimental
497254	+	TSS_005402	PMM0525	496211	0.69	Experimental
1295467	-	TSS_024423	PMM1342	1295547	0.68	Experimental
466192	+	TSS_005194	PMM0493	465840	0.65	Experimental

27367	+	TSS_000244	PMM0026	27273	0.64	Experimental
1599097	-	TSS_028992	PMM1667	1599261	0.63	Experimental
125778	+	TSS_001001	PMM0128	125323	0.62	Experimental
498873	+	TSS_005478	PMM0526	497550	0.62	Experimental
144023	+	TSS_001117	PMM0148	143931	0.60	Experimental
207728	-	TSS_015450	PMM0214	208944	0.60	Experimental
532769	+	TSS_005766	PMM0565	531701	0.59	Experimental
145715	+	TSS_001168	PMM0149	144701	0.59	Experimental
1439422	-	TSS_025879	PMM1500	1440282	0.58	Experimental
1423046	-	TSS_025773	PMM1485	1423524	0.56	Experimental
1380210	+	TSS_011135	PMM1443	1379949	0.54	Experimental
398719	+	TSS_004637	PMM0420	398147	0.53	Experimental
582602	-	TSS_018857	PMM0611	582767	0.52	Experimental
163563	-	TSS_015219	PMM0169	163802	0.51	Experimental
1398489	+	TSS_011316	PMM1465	1397666	0.50	Experimental
145511	+	TSS_001155	PMM0149	144701	0.49	Experimental
493996	-	TSS_018181	PMM0522	494177	0.48	Experimental
78295	-	TSS_014555	PMM0073	79154	0.48	Experimental
144965	+	TSS_001131	PMM0149	144701	0.47	Experimental
220578	-	TSS_015598	PMM0226	221632	0.47	Experimental
141294	-	TSS_014931	PMM0144	141905	0.46	Experimental
1224306	-	TSS_023777	PMM1270	1225100	0.46	Experimental
1367686	+	TSS_011008	PMM1428	1367587	0.45	Experimental
1252379	+	TSS_010051	PMM1300	1251088	0.45	Experimental
1491478	+	TSS_012250	PMM1562	1490373	0.45	Experimental
803506	-	TSS_020584	PMM0844	804298	0.45	Experimental
145211	+	TSS_001137	PMM0149	144701	0.44	Experimental
154964	-	TSS_015097	PMM0159	155061	0.44	Experimental
78158	-	TSS_014552	PMM0073	79154	0.44	Experimental
457755	-	TSS_017991	PMM0483	457975	0.44	Experimental
299836	-	TSS_016360	PMM0312	300325	0.43	Experimental
322674	+	TSS_003860	PMM0333	322362	0.43	Experimental
1421293	-	TSS_025730	PMM1485	1423524	0.42	Experimental
27552	+	TSS_000247	PMM0026	27273	0.42	Experimental
757372	-	TSS_020252	PMM0796	757822	0.41	Experimental
299950	-	TSS_016361	PMM0312	300325	0.39	Experimental
205343	+	TSS_001635	PMM0211	204910	0.39	Experimental
218599	+	TSS_002225	PMM0224	217978	0.38	Experimental
611411	+	TSS_006094	PMM0642	610941	0.38	Experimental
15146	+	TSS_000146	PMM0012	14568	0.37	Experimental
1300005	+	TSS_010667	PMM1349	1299740	0.37	Experimental
498249	+	TSS_005443	PMM0526	497550	0.35	Experimental
1218465	-	TSS_023642	PMM1264	1219792	0.34	Experimental
1440154	-	TSS_025922	PMM1500	1440282	0.34	Experimental
78218	-	TSS_014553	PMM0073	79154	0.34	Experimental
92441	+	TSS_000752	PMM0087	92350	0.33	Experimental
1034417	-	TSS_022375	PMM1088	1034569	0.33	Experimental
228037	+	TSS_002293	PMM0235	227959	0.32	Experimental
1645357	-	TSS_029444	PMM1707	1646216	0.31	Experimental
588874	+	TSS_005927	PMM0618	588622	0.31	Experimental
1033430	-	TSS_022195	PMM1088	1034569	0.29	Experimental
143021	-	TSS_014962	PMM0147	143883	0.29	Experimental
160552	-	TSS_015186	PMM0164	160684	0.28	Experimental
1253102	+	TSS_010066	PMM1301	1252970	0.28	Experimental
155334	-	TSS_015103	PMM0160	156249	0.28	Experimental
1371875	+	TSS_011034	PMM1434	1371646	0.27	Experimental
1499500	-	TSS_027366	PMM1570	1499639	0.26	Experimental
737882	-	TSS_020050	PMM0774	739095	0.25	Experimental
1549235	-	TSS_027936	PMM1619	1549798	0.25	Experimental
737771	-	TSS_020038	PMM0774	739095	0.24	Experimental
349634	-	TSS_017084	PMM0368	349709	0.23	Experimental
1609831	+	TSS_013402	PMM1676	1609627	0.23	Experimental
145139	+	TSS_001136	PMM0149	144701	0.23	Experimental
205032	+	TSS_001621	PMM0211	204910	0.22	Experimental
31393	-	TSS_014141	PMM0032	31536	0.22	Experimental
1131452	+	TSS_009499	PMM1186	1131150	0.22	Experimental
1240490	-	TSS_024057	PMM1289	1241067	0.21	Experimental
160039	-	TSS_015153	PMM0164	160684	0.21	Experimental
1008963	+	TSS_008515	PMM1066	1007850	0.20	Experimental
1507214	-	TSS_027453	PMM1577	1508411	0.20	Experimental
724438	-	TSS_019979	PMM0760	724578	0.20	Experimental
977946	+	TSS_008346	PMM1033	977043	0.20	Experimental
1131719	+	TSS_009506	PMM1186	1131150	0.19	Experimental
119905	+	TSS_000937	PMM0121	119798	0.19	Experimental
384144	+	TSS_004505	PMM0405	383585	0.19	Experimental
386764	-	TSS_017359	PMM0407	387174	0.18	Experimental
738857	-	TSS_020089	PMM0774	739095	0.18	Experimental
1239822	+	TSS_009912	PMM1288	1239042	0.16	Experimental
384720	+	TSS_004566	PMM0405	383585	0.16	Experimental
1523398	-	TSS_027547	PMM1594	1523570	0.15	Experimental
328681	-	TSS_016997	PMM0339	329348	0.14	Experimental
1636527	+	TSS_013754	PMM1700	1635526	0.13	Experimental
306125	-	TSS_016557	PMM0321	306386	0.13	Experimental
1401712	-	TSS_025249	PMM1467	1403187	0.13	Experimental
901817	-	TSS_021079	PMM0945	905299	0.13	Experimental
384519	+	TSS_004541	PMM0405	383585	0.13	Experimental
217197	+	TSS_001906	PMM0223	216745	0.12	Experimental
1022762	-	TSS_021876	PMM1080	1023493	0.12	Experimental
310880	+	TSS_002942	PMM0326	310480	0.11	Experimental
1465661	-	TSS_019863	PMM1524	1466574	0.11	Experimental
32608	-	TSS_014167	PMM0035	33713	0.11	Experimental
383961	+	TSS_004480	PMM0405	383585	0.11	Experimental
1008779	+	TSS_008503	PMM1066	1007850	0.10	Experimental

431182	-	TSS_017698	PMM0452	432317	0.09	Experimental
32866	-	TSS_014189	PMM0035	33713	0.09	Experimental
310023	+	TSS_002878	PMM0325	309786	0.09	Experimental
1131989	+	TSS_009521	PMM1186	1131150	0.08	Experimental
1083716	+	TSS_008966	PMM1132	1082218	0.08	Experimental
1238666	-	TSS_024013	PMM1287	1238943	0.08	Experimental
166513	+	TSS_001406	PMM0172	165910	0.08	Experimental
1549500	-	TSS_027954	PMM1619	1549798	0.07	Experimental
92216	-	TSS_014618	PMM0086	92293	0.07	Experimental
166228	+	TSS_001376	PMM0172	165910	0.07	Experimental
734570	+	TSS_006758	PMM0770	734269	0.06	Experimental
927441	+	TSS_007993	PMM0970	926558	0.06	Experimental
1223575	-	TSS_023765	PMM1269	1224017	0.05	Experimental
926661	+	TSS_007807	PMM0970	926558	0.05	Experimental
1009058	+	TSS_008517	PMM1066	1007850	0.04	Experimental
147473	+	TSS_001238	PMM0150	146794	0.03	Experimental
147440	+	TSS_001236	PMM0150	146794	0.03	Experimental
147116	+	TSS_001224	PMM0150	146794	0.03	Experimental
1138412	+	TSS_009588	PMM1191	1136852	0.03	Experimental
253072	+	TSS_002460	PMM0263	252586	0.03	Experimental
252875	+	TSS_002439	PMM0263	252586	0.03	Experimental
147332	+	TSS_001231	PMM0150	146794	0.02	Experimental
147971	+	TSS_001271	PMM0150	146794	0.02	Experimental
