# Supplementary material for: Nitrogen cost minimization is promoted by structural changes in the transcriptome of N-deprived Prochlorococcus cells
Source: ISME J. 2017 Jun 6;11(10):2267–78. doi: 10.1038/ismej.2017.88 (PMC5607370; doi:10.1038/ismej.2017.88)
Supplement: Supplementary Table 14 [file ismej201788x21.pdf]

Table S14. Sequence IDs and protein threading results from transcripts with significant internal transcriptional starts sites in MED4 under N-limitation. The sequences highlighted in bold contain internal start sites with an NtcA binding site during transcription and a pyrimidine binding site for protein S1 binding during translation. The genes in this table combine for more than 90% of the gene expression during the treatment.

Sequence ID	Structure with best match	Protein	Model Confidence	Residues aligned	Amino Acids in Sequence	Difference Between Full length and Internal Protein Length	CDD Domain Affected by Internal TSS	Accession Number of CDD Domain Affected	Result	NtcA Binding	Pyrimidine Rich	Theoretical N-Savings (mol N/mol protein)
**PMM0211**	**c1hyuA_**	**Alkyl hydroperoxide reductase subunit**	**100**	**301**	**318**							
**PMM0211 Int**	**c1hyuA_**		**100**	**241**	**251**	**67**	**None**	**N/A**	**Protein aligns by MSA to major structural elements of PDB structures**	**Yes**	**Yes**	**79**
**PMM0214**	**c5da0A_**	**Sulfate transporter**	**100**	**460**	**554**							
**PMM0214 Int**	**c3mglA_**		**99**	**110**	**156**	**398**	**Possible specific transcription of HCO3_cotransp super family domain**	**cl23746**	**Protein has specific structural element or subunit transcribed**	**Yes**	**Yes**	**474**
**PMM0370**	**c2i4cA_**	**putative cyanate ABC transporter, substrate binding protein**	**100**	**361**	**561**							
**PMM0370 Int**	**c2i4cA_**		**100**	**365**	**406**	**155**	**None**	**N/A**	**Protein aligns by MSA to major structural elements of PDB structures**	**Yes**	**Yes**	**210**
**PMM1075**	**c2b5oA**	**Ferredoxin-nadp reductase**	**100**	**288**	**373**							
**PMM1075 Int**	**c1fncA_**		**100**	**234**	**238**	**135**	**None**	**N/A**	**Protein aligns by MSA to major structural elements of PDB structures**	**Yes**	**Yes**	**179**
**PMM1485**	**c3lu0C_**	**RNA polymerase**	**100**	**1032**	**1100**							
**PMM1485 Int**	**c3lu0C_**		**100**	**936**	**988**	**112**	**Possible modification of RNA polymerase beta subunit as almost entire protein is single domain**	**cd00653**	**Protein aligns by MSA to major structural elements of PDB structures**	**Yes**	**Yes**	**155**
**PMM1512**	**c1lm1A_**	**Glutamate synthase [nadph] large chain**	**100**	**1461**	**1521**							
**PMM1512 Int**	**c2vdcF_**		**100**	**410**	**413**	**1108**	**Possible specific transcription of GXGXG superfamily domain.**	**cl00239**	**Protein has specific structural element or subunit transcribed**	**Yes**	**Yes**	**1501**
**PMM1697**	**c4igcX**	**Type II alternative RNA polymerase sigma factor, sigma-70 family**	**100**	**338**	**339**							
**PMM1697 Int**	**c4igcX**		**100**	**176**	**187**	**111**	**Likely loss of Sigma-70 factor, region 1.2 domain**	**pfam00140**	**Protein lacks structural element or structure is incomplete**	**Yes**	**Yes**	**380**
PMM0063	c2vv5D_	putative membrane transport protein	91	79	310							
PMM0063 Int	c4b4aA_		89	19	205	105	Possible modification of the Ycf66 protein N-terminus domain	pfam07444	Protein lacks structural element or structure is incomplete	No	No	136
PMM0087	c2kbcA_	putative hormone protein	11	13	112							
PMM0087 Int	c2kbcA_		14	13	95	17	No domains detected	N/A	Protein lacks structural element or structure is incomplete	No	No	28
PMM0164	c5kinD_	tryptophan synthase beta chain	100	393	417							
PMM0164 Int	d1v8za1		100	371	377	40	None	N/A	Protein aligns by MSA to major structural elements of PDB structures	No	No	59
PMM0263	c5aexB_	ammonium transporter	100	419	486							
PMM0263 Int	c5aexB_		100	403	405	81	None	N/A	Protein aligns by MSA to major structural elements of PDB structures	No	No	113
PMM0368	c3znul_	putative lyase protein	49	73	105							
PMM0368 Int	d1kp0a1		53	31	84	21	First 16 aas of a Domain of Unknown Function	pfam11746	Protein lacks structural element or structure is incomplete	No	Yes	29
PMM0445	c1m56G_	cytochrome c oxidase subunit I	100	512	544							
PMM0445 Int	c1m56G_		100	451	463	81	Possible modification of cytochrome c oxidase subunit I as almost entire protein is single domain	TIGR02891	Protein aligns by MSA to major structural elements of PDB structures	No	Yes	105
PMM0545	c3aerC_	light-independent protochlorophyllide reductase subunit n	100	407	421							
PMM0545 Int	c3aerC_		100	256	263	158	Possible modification of light-independent protochlorophyllide reductase subunit n as almost entire protein is single domain	PRK02842	Protein aligns by MSA to major structural elements of PDB structures	No	Yes	210
PMM0661	d1l1la_	B12-dependent (class II) ribonucleotide reductase	100	653	805							
PMM0661 Int	d1l1la_		100	599	706	99	None	N/A	Protein aligns by MSA to major structural elements of PDB structures	No	Yes	144
PMM0684	d1smpi_	putative metalloprotease inhibitor	52	23	117							
PMM0684 Int	d2v3za1		40	32	105	12	None	N/A	Protein lacks structural element or structure is incomplete	No	No	15
PMM0687	c3edsA_	putative ctp pyrophosphohydrolase	24	18	111							
PMM0687 Int	c3edsA_		40	27	89	22	No domains detected	N/A	Protein lacks structural element or structure is incomplete	Yes	Yes	36
PMM0804	c3a9qR_	ferritin	100	177	184							
PMM0804 Int	c3qz3A_		100	160	166	18	None	N/A	Protein aligns by MSA to major structural elements of PDB structures	No	No	24
PMM0872	c4a94D_	putative carboxypeptidase inhibitor	73	18	82		No domains detected					
PMM0872 Int	c4a94D_		73	18	81	1	No domains detected		Protein lacks structural element or structure is incomplete	No	Yes	1
PMM0970	d1qo0a_	urea ABC transporter, Periplasmic binding protein-like I	100	368	439							
PMM0970 Int	d1qo0a_		100	357	381	58	None	N/A	Protein aligns by MSA to major structural elements of PDB structures	No	Yes	72
PMM1077	c4ew8A_	adaptive-response sensory kinase protein divl	100	228	375							
PMM1077 Int	c4ew8A_		100	207	226	149	None	N/A	Protein has specific structural element or subunit transcribed	No	Yes	211
PMM1123	c4l6rA_	putatuve cytochrome b562 and glucagon receptor chimera	33	30	237							
PMM1123 Int	d1oz9a_		19	13	180	57	None	N/A	Protein lacks structural element or structure is incomplete	No	Yes	88
PMM1158	c2axtc_	photosystem ii cp43 protein	100	446	460							
PMM1158 Int	c2axtc_		100	416	416	44	Possible modification of the photosystem ii cp43 protein as almost entire protein is single domain	TIGR01153	Protein aligns by MSA to major structural elements of PDB structures	Yes	No	64
PMM1186	3wvfA_	membrane protein insertase yidc	100	183	379							
PMM1186 Int	c3wvfA_		100	177	288	91	Possible modification of the membrane protein insertase yidc protein as almost entire protein is single domain	PRK02654	Protein aligns by MSA to major structural elements of PDB structures	No	Yes	120
PMM1301	c3n5mD_	adenosylmethionine-8-amino-7-oxononanoate aminotransferase	100	392	417							
PMM1301 Int	c4ysnD_		100	243	245	225	Possible modification of the acetylornithine aminotransferase protein as almost entire protein is single domain	PRK02627	Protein aligns by MSA to major structural elements of PDB structures	No	No	229
PMM1312	c5f4hF_	nucleotide binding protein pinc	100	159	369							
PMM1312 int	c5f4hF_		100	159	368	1	Possible specific transcription of (PilT N terminus) domain	cd09877	Protein has specific structural element or subunit transcribed	No	Yes	1
PMM1434	c4my4A_	2,3-bisphosphoglycerate-independent phosphoglycerate	100	500	540							
PMM1434 Int	c4my4A_		100	449	474	66	Possible modification of the 2,3-bisphosphoglycerate-independent phosphoglycerate protein as almost entire protein is single domain	cd16010	Protein aligns by MSA to major structural elements of PDB structures	No	No	89
PMM1625	c3dhyC_	adenosylhomocysteinase	100	463	475							
PMM1625 Int	c3n58D_		100	339	339	136	loss of the beginning amino acids in the homotetramer interface on conserved domain SAHH	PRK05476	Protein aligns by MSA to major structural elements of PDB structures	No	No	178
PMM1636	c5lmtX_	translation initiation factor if-3	100	162	190							
PMM1636 Int	c5lmtX_		100	107	119	71	loss of part of translation initiation factor 3 N terminal domain	pfam05198	Protein aligns by MSA to major structural elements of PDB structures	No	Yes	113
PMM1642	d2gexa1	putative SnoaL-like polyketide cyclase	100	124	146							
PMM1642 Int	d2gexa1		100	94	111	35	loss of the beginning amino acids in the SnoaL-like domain	pfam12680	Protein aligns by MSA to major structural elements of PDB structures	No	Yes	111
