# Supplementary material for: Nitrogen cost minimization is promoted by structural changes in the transcriptome of N-deprived Prochlorococcus cells
Source: ISME J. 2017 Jun 6;11(10):2267–78. doi: 10.1038/ismej.2017.88 (PMC5607370; doi:10.1038/ismej.2017.88)
Supplement: Supplementary Table 15 [file ismej201788x22.pdf]

Table S15: Relative number of pyrimidine-rich regions and Possible NtcA binding sites

Pyrimidine Rich Regions		NtcA Binding Sites
10mer	12mer	
959	438	287

* Pyrmindine-rich regions were 80% pyrimidine within 100bp of annotated translational start site
** NtcA binding sites were discovered based on published motifs and were within 100 bp of annotated translation start site.
