# Supplementary material for: Nitrogen cost minimization is promoted by structural changes in the transcriptome of N-deprived Prochlorococcus cells
Source: ISME J. 2017 Jun 6;11(10):2267–78. doi: 10.1038/ismej.2017.88 (PMC5607370; doi:10.1038/ismej.2017.88)
Supplement: Supplementary Table 16 [file ismej201788x23.pdf]

Table S16. Autocorrelation of Expression Values for Three Datasets -- This study all data, This study top 50% expression values and Tolonen et al. (2006).

		3 Hours Post Starvation	12 Hours Post Starvation	24 Hours Post Starvation
This study	3 Hours Post Starvation			
	12 Hours Post Starvation	-0.019		
	24 Hours Post Starvation	-0.012	0.804	
This study (Top 50%)	3 Hours Post Starvation			
	12 Hours Post Starvation	-0.055		
	24 Hours Post Starvation	-0.069	0.892	
Tolonen et al (2006)	3 Hours Post Starvation			
	12 Hours Post Starvation	0.499		
	24 Hours Post Starvation	0.549	0.772	
