# Supplementary material for: Nitrogen cost minimization is promoted by structural changes in the transcriptome of N-deprived Prochlorococcus cells
Source: ISME J. 2017 Jun 6;11(10):2267–78. doi: 10.1038/ismej.2017.88 (PMC5607370; doi:10.1038/ismej.2017.88)
Supplement: Supplementary Table 17 [file ismej201788x24.pdf]

Table S17. Transcripts Described During 3 Hours Post Starvation

Name	Log2 Fold Change (3hr)	Standard Error (3hr)	p-value (3hr)	Log2 Fold Change (12hr)	Standard Error (12hr)	p-value (12hr)	Log2 Fold Change (24hr)	Standard Error (24hr)	p-value (24hr)	Category	MIT Annotation
PMM0373	3.49	1.77	7.58E-16	1.71	0.34	2.25E-01	-0.37	0.33	6.37E-01	Other	Cyanate Lyase
PMM1514	2.02	1.14	1.04E-01	-0.11	0.54	6.90E-01	0.74	1.23	8.70E-01	Other	LipA Lipoic Acid Synthetase
PMM0258	2.05	0.69	2.75E-02	1.89	0.68	3.00E-02	1.81	0.39	1.98E-02	Other	glyA Serine hydroxymethyltransferase (SHMT)
PMM0246	0.57	0.11	7.62E-02	1.75	1.1	2.62E-01	2.17	1.2	3.48E-03	Transport and binding proteins	ntcA Global N regulatory protein
PMM1294	2.39	0.17	1.33E-02	0.13	0.33	7.42E-01	0.6	0.23	1.00E+00	Hydrogenase	Nickel-containing superoxide dismutase precursor (NISOD)
PMM0550	2.93	0.5	4.41E-05	-1.48	0.14	5.18E-03	-1.28	0.16	1.09E-01	CO2 fixation	rbcL, cbbL Ribulose bisphosphate carboxylase, large chain
PMM0551	1.86	1.22	9.56E-07	-1.09	0.45	2.50E-01	-1.24	0.01	8.99E-02	CO2 fixation	rbcS, cbbS Ribulose bisphosphate carboxylase, Small chain
PMM0263	3.67	0.28	2.30E-02	2.27	0.07	1.00E+00	2.26	0.16	1.00E+00	Transport and binding proteins	amt1 Ammonium transporter family
PMM0970	0.22	0	1.00E+00	4.07	0.33	3.52E-20	2.8	0.19	8.94E-03	Transport and binding proteins	urtA putative urea ABC transporter
PMM0971	-0.57	0.51	1.00E+00	2.37	0.69	4.93E-03	1.39	0.27	7.82E-02	Transport and binding proteins	urtB putative urea ABC transporter
PMM0370	-1.98	0.65	8.85E-02	3.85	0.42	5.77E-36	3.84	0.21	8.01E-32	Transport and binding proteins	putative cyanate ABC transporter (CynA)
PMM0371	0.08	1.01	1.00E+00	0.52	0.47	6.90E-01	2.65	0.5	2.80E-07	Transport and binding proteins	putative cyanate ABC transporter (CynB)
PMM0471	-2.38	0.48	4.52E-04	-0.85	0.25	3.91E-01	-2.22	1.26	3.49E-04	Adaptations and atypical conditions	hli20 possible high light inducible protein
PMM1615	-2.79	0.07	2.95E-07	2.44	0.1	2.30E-04	2.15	0.75	1.34E-02	DNA replication recombination and repair	ruvB Holliday junction DNA helicase RuvB
