# Supplementary material for: Nitrogen cost minimization is promoted by structural changes in the transcriptome of N-deprived Prochlorococcus cells
Source: ISME J. 2017 Jun 6;11(10):2267–78. doi: 10.1038/ismej.2017.88 (PMC5607370; doi:10.1038/ismej.2017.88)
Supplement: Supplementary Table 18 [file ismej201788x25.pdf]

Table S18. Ribosomal Transcripts with Significant Regulation 12 and 24 Hours Post Starvation

Name	Log2 Fold Change (3hr)	Standard Error (3hr)	p-value (3hr)	Log2 Fold Change (12hr)	Standard Error (12hr)	p-value (12hr)	Log2 Fold Change (24hr)	Standard Error (24hr)	p-value (24hr)	Category	MIT Annotation
PMM1706	2.06	1.73	1.00E+00	-4.65	2.38	2.45E-280	-2.2	1.58	1.22E-20	Ribosomal proteins	30S ribosomal protein S6
PMM1558	-0.82	0.66	1.00E+00	-4.3	1.26	5.94E-111	-4.05	1.14	9.49E-112	Ribosomal proteins	50S ribosomal protein L3
PMM1537	-0.34	0.28	1.00E+00	-3.72	0.62	7.61E-78	-4.16	0.2	1.72E-169	Ribosomal proteins	30S ribosomal protein S13
PMM1538	-0.55	0.1	1.00E+00	-3.41	0.63	9.72E-69	-4.17	0.58	1.41E-210	Ribosomal proteins	50S Ribosomal protein L36
PMM1511	-0.34	0.05	1.00E+00	-3.43	0.56	7.88E-38	-3.29	0.07	6.80E-32	Ribosomal proteins	30S ribosomal protein S12
PMM1345	-0.08	0.57	1.00E+00	-3.13	0.22	1.27E-18	-2.84	0.36	8.55E-14	Ribosomal proteins	50S ribosomal protein L27
PMM0987	-2.06	0.41	1.63E-01	-2.96	0.14	7.03E-15	-3.56	0.56	5.71E-45	Ribosomal proteins	30S Ribosomal protein S21
PMM1536	0.51	0.06	1.00E+00	-2.8	0.09	7.22E-13	-3.01	0.11	1.01E-17	Ribosomal proteins	30S ribosomal protein S11
PMM1285	-0.28	0.03	1.00E+00	-2.85	0.17	8.81E-14	-3.29	0.85	4.88E-25	Ribosomal proteins	30S Ribosomal protein S16
PMM0202	0.89	0.24	1.00E+00	-2.54	0.41	8.49E-14	-2.46	0.25	1.68E-11	Ribosomal proteins	50S ribosomal protein L10
PMM0870	-1.44	0.61	1.00E+00	-2.68	0.77	1.77E-09	-3.22	0.12	9.39E-35	Ribosomal proteins	50S Ribosomal protein L33
PMM0203	-1.3	0.12	1.00E+00	-2.46	0.16	4.06E-12	-1.98	0.32	5.65E-04	Ribosomal proteins	50S ribosomal protein L1
PMM1555	-0.72	0.21	1.00E+00	-2.44	0.1	2.82E-11	-2.54	0.42	9.37E-13	Ribosomal proteins	50S ribosomal protein L2
PMM0201	1.25	0.04	1.00E+00	-2.45	0	6.75E-09	-2.3	0.04	5.06E-07	Ribosomal proteins	50S ribosomal protein L7/L12
PMM0312	-0.02	0.11	1.00E+00	-2.38	0	3.74E-06	-1.75	0.31	1.55E-03	Ribosomal proteins	30S ribosomal protein S1, homolog A
PMM1552	-0.45	0.31	1.00E+00	-2.26	0.28	5.74E-06	-2.56	0.04	7.26E-11	Ribosomal proteins	30S ribosomal protein S3
PMM1344	0.45	0.31	1.00E+00	-2.28	0.5	5.56E-06	-2.59	0.36	7.67E-09	Ribosomal proteins	50S ribosomal protein L21
PMM1553	-0.32	0.16	1.00E+00	-2.23	0.12	5.43E-05	-2.42	0.54	3.24E-10	Ribosomal proteins	50S ribosomal protein L22
PMM1532	0.58	0.32	1.00E+00	-2.19	0.28	1.98E-05	-2.98	0	2.89E-21	Ribosomal proteins	50S ribosomal protein L13
PMM0410	-0.72	0.58	1.00E+00	-2.04	0.49	4.05E-08	-1.68	0.48	3.87E-03	Ribosomal proteins	30S ribosomal protein S4
PMM1554	0.52	0.22	9.10E-01	-2.06	0.41	1.35E-04	-2.15	0.49	1.09E-05	Ribosomal proteins	30S Ribosomal protein S19
PMM1661	-1.53	0.23	7.80E-01	-2.05	0.94	2.78E-05	-2.81	0.82	1.57E-20	Ribosomal proteins	50S ribosomal protein L35
PMM1556	-0.73	0.13	1.00E+00	-1.9	0.6	3.55E-05	-2.11	0.02	1.76E-05	Ribosomal proteins	50S ribosomal protein L23
PMM1545	-0.48	0.45	8.22E-01	-2.04	0.08	1.02E-02	-1	0.3	2.06E-01	Ribosomal proteins	30S ribosomal protein S8
