# Supplementary material for: Nitrogen cost minimization is promoted by structural changes in the transcriptome of N-deprived Prochlorococcus cells
Source: ISME J. 2017 Jun 6;11(10):2267–78. doi: 10.1038/ismej.2017.88 (PMC5607370; doi:10.1038/ismej.2017.88)
Supplement: Supplementary Table 19 [file ismej201788x26.pdf]

Table S19. Predicted Operon Organization by Rockhopper

Start	Stop	Strand	Number of Genes	Genes
1412664	1423313	-	5	PMM1481, hli3, rpoC2, rpoC1, rpoB
1046989	1057039	-	6	recD, recB, PMM1104, recC, PMM1106, pdxJ
1163795	1173759	+	10	PMM1214, PMM1215, PMM1216, PMM1217, rfaE, PMM1219, PMM1220, PMM1221, PMM1222, PMM1223
1181148	1190461	+	10	PMM1229, PMM1230, PMM1231, PMM1232, rfbD, PMM1234, PMM1235, PMM1236, PMM1237, PMM1238
747739	756909	-	10	leuB, lpxD, proB, PMM0789, PMM0790, PMM0791, PMM0792, PMM0793, PMM0794, PMM0795
560476	569580	-	7	PMM0593, ndhD, thrB, PMM0596, thrS, trpS, PMM0599
1473853	1481941	-	14	truA, rplQ, rpoA, rps11, rpsM, rpmJ, adk, secY, rplO, rpsE, rplR, rplF, rpsH, rplE
1283592	1290442	-	7	PMM1333, lpxB, lpxA, fabZ, lpxC, PMM1338, purC
518725	525382	+	7	csoS1, rbcL, rbcS, csoS2, csoS3, PMM0554, PMM0555
689797	696364	-	6	PMM0727, PMM0728, PMM0729, PMM0730, PMM0731, PMM0732
585243	591682	+	6	PMM0614, PMM0615, PMM0616, murI, sds, acs
414484	420509	+	5	topA, PMM0437, PMM0438, PMM0439, PMM0440
1384483	1390439	-	9	PMM1447, PMM1448, petF, atpC, atpA, atpH,atpD, atpF, atpG, PMM1455
713478	719193	+	5	rpsB, tsf, PMM0755, recG, PMM0757
799312	804919	-	5	fmt, pmbA, tldD, PNIL34,AT103, PMM0845
1482017	1487615	-	12	rplX, rplN, rpsQ, rpl29, rplP, rpsC, rplV, rpsS, rplB, rplW, rplD, rplC
872358	877920	+	5	pykF, PMM0913, PMM0914, PMM0915, PMM0916
1035266	1040822	+	6	lysA, PMM1091, uppS, BioB, PMM1094, F2J10.13
1194486	1199985	-	6	PMM1242, PMM1243, PMM1244, PMM1245, PMM1246, PMM1247
1089194	1094685	+	3	PMM1139, polA, cysS
1599361	1604716	-	4	gcvP, gcsH, PMM1670, PMM1671
926568	931720	+	5	urtA, urtB, urtC, urtD, urtE
1623197	1628319	+	5	pyrG, PMM1690, PMM1691, PMM1692, PMM1693
569751	574840	+	5	PMM0600, PMM0601, PMM0602, PMM0603, PMM0604
1450922	1455790	+	2	glsF, PMM1513
74373	79121	-	4	PMM0070, PMM0071, PMM0072, PMM0073
477171	481864	-	4	PMM0505, purA,adeK, psb27, proS
708634	713327	+	6	pcyA, PMM0748, PMM0749, PMM0750, PMM0751, PMM0752
59496	64148	+	2	PMM0057, PMM0058
1649575	1654209	-	2	uvrA, recN
907197	911829	-	6	PMM0948, PMM0949, rsbV, carA, trpD, PMM0953
244685	249266	-	4	leuD, leuC, PMM0257, glyA
1403281	1407851	+	5	bioF, PMM1469, PMM1470, BioD, BioA
1461905	1466465	-	2	psaB, psaA
1030010	1034552	-	3	todF, gldA, clpC
1379976	1384477	+	4	PMM1443, PMM1444, nadD, PMM1446
832609	837108	-	2	metH, ilvE
790292	794787	+	7	dapB, PMM0833, ubiH, PMM0835, PMM0836, PMM0837, PMM0838
1558374	1562820	-	5	PMM1630, PMM1631, PMM1632, PMM1633, gyrB
638594	642922	-	4	PMM0671, PMM0672, PMM0673, aspC
1013873	1018110	-	3	cbiA,cobB, PMM1073, zwf
174	4383	+	3	dnaN, PMM0002, purL
1274850	1279020	+	5	PMM1323, PMM1324, PMM1325, PMM1326, PMM1327
10367	14526	+	4	PMM0008, nusB, ftsY, PMM0011
152080	156231	-	6	PMM0155, ppnK, ndhE, ndhG, ndhI, ndhA
40052	44190	-	3	PMM0041, PMM0042, PMM0043
210201	214315	+	4	PMM0216, mutS, obgE, PMM0219
191925	195968	-	5	hisC/cobC, pyrD, rnhA, rplL, rplJ
1606266	1610275	+	3	dnaB, PMM1675, PMM1676
1096353	1100323	-	4	PMM1144, pntB, pntA-2, pntA-1
88020	91964	+	3	PMM0083, PMM0084, PMM0085
1635543	1639484	+	2	acnB, PMM1701
993604	997537	-	5	thrA,hom, PMM1052, PMM1053, ruvC, chlI
975567	979494	+	3	PMM1032, PMM1033, PMM1034
126143	130037	-	3	holB, PMM0130, PMM0131
1124366	1128233	-	3	PMM1177, PMM1178, PMM1179
1043002	1046851	-	3	PMM1099, PMM1100, PMM1101
352975	356820	+	4	PMM0370, PMM0371, PMM0372, cynS
1002199	1006038	+	2	gyrA, crtL1
857094	860917	-	4	PMM0894, PMM0895, dnaJ2, dnaK
1149095	1152910	-	4	PMM1201, PMM1202, rfbG, ddhA
177069	180883	-	3	hisC, argS, nadC
1508995	1512703	-	3	PMM1579, rodA, PMM1581
863832	867515	+	6	htpG, rpmB, PMM0903, PMM0904, PMM0905, psaK
289627	293290	-	2	PMM0303, uvrD
173420	177072	+	6	prfB, PMM0181, PMM0182, dgkA, pabA, PMM0185
98735	102370	-	3	PMM0096, PMM0097, PMM0098
1258409	1262024	+	4	PMM1305, ddl, PMM1307, PMM1308
1060080	1063692	-	4	PMM1114, crtH, gidA, pbsY
581401	585009	-	3	glmU, PMM0612, aroA
676875	680425	-	3	PMM0711, PMM0712, gap1

1538208	1541750	+	3	acpP, fabF, tktA
1654279	1657817	+	3	PMM1714, PMM1715, thrC
916593	920110	-	3	PMM0959, PMM0960, PMM0961
422426	425917	-	4	PMM0443, ctaE, ctaD, ctaC
229639	233103	+	2	PMM0237, ileS
1502288	1505707	-	2	PMM1574, ppc
1290576	1293983	+	2	purD, nblS
318175	321573	-	4	FPG, psaE, PMM0330, PMM0331
888188	891578	-	3	secF, secD, pdhB
1613535	1616904	-	2	PMM1681, valS
1629153	1632509	+	2	PMM1695, ppk
1562973	1566307	+	4	miaA, infC, PMM1637, cysE
387242	390561	-	3	PMM0408, metB, rpsD
1446430	1449748	-	2	tuf, fusA
921384	924678	-	4	ureC, ureB, ureA, ureD
488453	491727	+	3	PMM0517, ftsI, tal
805514	808778	-	4	zam, PMM0848, PMM0849, PMM0850
981474	984734	+	3	PMM1038, PMM1039, PMM1040
306489	309735	-	3	minC, PMM0323, ctpA
823040	826283	-	4	metG, PMM0868, rpsR, rpmG
1229209	1232440	-	4	PMM1278, PMM1279, PMM1280, era
1543322	1546553	-	4	PMM1612, PMM1613, PMM1614, ruvB
1520852	1524069	-	5	PMM1591, pebB, pebA, ho1, PMM1595
370993	374209	+	4	engA, PMM0392, PMM0393, PMM0394
438164	441311	-	4	cobM, yvoC, petA, petC
1525676	1528817	-	3	PMM1597, PMM1598, PMM1599
379082	382186	+	3	lipB, fadD, PMM0403
734287	737385	+	4	gnd, PMM0771, PMM0772, PMM0773
167587	170668	-	3	menE, menC, menA
1247716	1250792	-	3	trpC, lpdA, spoU
18283	21295	+	4	grpE, dnaJ, PMM0018, PMM0019
426170	429153	+	3	PMM0447, ctaB, PMM0449
527242	530220	+	3	hisG, PMM0561, PMM0562
269602	272567	-	3	PMM0280, hisB, fabI
81171	84133	+	2	pgm, yrvN
406258	409210	-	3	PMM0427, chlG, PMM0429
205971	208916	-	3	PMM0212, sbtA, PMM0214
499746	502658	-	3	PMM0528, PMM0529, rps1b
725513	728423	+	3	typA, PMM0763, PMM0764
1233875	1236761	-	3	phoH,psiH, rpsP, ffh
1578026	1580904	-	2	uvrB, PMM1650
613198	616075	-	2	PMM0644, PMM0645
990673	993541	-	3	PMM1048, PMM1049, PMM1050
1591512	1594380	-	2	PMM1659, PMM1660
513553	516393	-	2	chlB, chlN
578565	581398	+	2	glgA, murF
441721	444553	-	3	PMM0464, PMM0465, PMM0466
1216960	1219777	-	2	PMM1263, ftsH3
1575216	1578017	+	2	PMM1647, lysC
143954	146728	+	2	PMM0148, ndhF
504457	507217	+	3	PMM0533, accA, PMM0535
108003	110754	-	3	PMM0104, ribG, PMM0106
1133930	1136661	+	3	serS, PMM1189, rpsN
899088	901786	+	5	PMM0940, PMM0941, ruvA, rpsO, PMM0944
21261	23953	-	3	PMM0020, murB, murC
671290	673975	-	2	PMM0707, PMM0708
846450	849130	-	3	PMM0886, nifS, dapF
1434244	1436918	+	2	PMM1496, PMM1497
685879	688537	+	3	pstC,phoW, pstA,phoT, PMM0725
575865	578519	+	2	menD, menB
462266	464918	-	2	futB,hitB, PMM0490
737403	740043	-	3	ilvD, PMM0775, PMM0776
1505761	1508400	-	2	PMM1576, trpE
1255497	1258126	-	2	PMM1303, codA
1131179	1133805	+	2	PMM1186, PMM1187
1547126	1549740	-	3	PMM1617, lysS, PMM1619
274511	277122	-	3	phr, PMM0286, folK
1617516	1620104	+	3	mazG, speE, PMM1686
484307	486891	-	2	PMM0514, cobC
933470	936042	+	3	PMM0976, PMM0977, PMM0978
810354	812916	+	2	ftsH4, PMM0855
68803	71353	-	3	hli2, PMM0065, PMM0066
134349	136892	+	3	fabH, fabD, plsC

219107	221616	-	2	PMM0225, ftsH2
472859	475364	-	3	PMM0498, argB, PMM0500
911909	914414	+	2	PMM0954, msrA
1302241	1304746	-	2	prmA, serA
383670	386168	+	2	pdhC, queA
1253002	1255500	+	2	argD, folC
296547	299024	-	2	PMM0310, metK
706117	708593	-	2	PMM0745, PMM0746
842214	844682	-	2	uvrC, PMM0883
1190636	1193092	+	2	hetA, PMM1240
776518	778961	-	4	PMM0821, K8K14.9, PMM0823, PMM0824
1107613	1110055	+	2	psbD, psbC
102557	104991	+	2	PMM0099, nadB
491753	494164	-	3	PMM0520, frr, pyrH
861281	863682	+	3	petF, PMM0899, hisZ
1440558	1442949	+	2	PMM1501, rnhB
744191	746566	-	3	PMM0782, PMM0783, accD
631192	633554	+	2	PMM0662, prfC
1596609	1598964	+	2	sqdB, sqdX
139518	141867	-	2	crtB,pys, pds,crtD
1213939	1216288	+	2	PMM1260, ugd
1549913	1552259	-	3	PMM1620, mreC, mreB
34925	37269	+	2	guaA, PMM0038
1011546	1013876	+	3	folD, crtE, PMM1071
730434	732754	+	2	glpX, hemA
1279010	1281319	-	3	pyrF, tyrS, PMM1330
368613	370920	+	4	aroD, PMM0388, cobI/cbiL, PMM0390
116503	118799	+	4	PMM0116, PMM0117, PMM0118, PMM0119
558202	560483	+	3	proA, folB, PMM0592
1211578	1213846	+	2	PMM1258, PMM1259
1027581	1029845	+	2	cad, PMM1085
1206280	1208536	-	2	PMM1254, PMM1255
1471482	1473736	-	4	prfA, rpmE, rpsI, rplM
1202291	1204539	-	2	PMM1250, PMM1251
610958	613191	+	2	cysD, metA
412094	414315	-	2	PMM0434, ndhB
1226538	1228724	+	3	ribF, thiE, PMM1275
242502	244681	+	2	psbI, PMM0254
460093	462269	+	2	PMM0487, PMM0488
606272	608444	-	2	PMM0638, stpA
1409390	1411559	-	4	PMM1476, PMM1477, PMM1478, PMM1479
1158248	1160415	-	2	galE, PMM1210
1428101	1430265	+	3	PMM1491, nusA, PMM1493
465856	468011	+	2	cxp, ppa
132148	134295	+	2	rpaB, plsX
894799	896939	+	3	PMM0936, umuC, PMM0938
891762	893874	-	3	PMM0931, ispE, ksgA
1281470	1283579	+	2	PMM1331, PMM1332
390657	392746	+	3	PMM0411, PMM0412, murE
1552760	1554840	-	2	PMM1624, ahcY
25173	27243	-	2	thiL, PMM0025
782376	784444	-	2	PMM0826, PMM0827
1156082	1158142	-	2	PMM1207, gmd
267567	269599	+	2	PMM0278, PMM0279
377005	379037	-	3	PMM0398, PMM0399, lrtA
546886	548918	-	2	PMM0582, hemE
393515	395523	-	2	PMM0415, PMM0416
600795	602798	-	2	PMM0632, crtL2
943361	945357	-	2	PMM0990, PMM0991
1268128	1270118	+	2	ilvC, cobD
1223093	1225080	-	2	ppnK, pheS
202752	204736	-	2	PMM0209, PMM0210
635907	637881	+	3	PMM0667, PMM0668, PMM0669
1594472	1596442	+	4	rpmI, rplT, PMM1663, thiG
1393437	1395380	+	2	ccdA, ycf44
148684	150625	+	2	PMM0151, PMM0152
1219892	1221831	-	3	ribD, PMM1266, PMM1267
1584044	1585980	-	2	dapA, asd
114476	116403	-	2	PMM0114, crtQ
28330	30250	-	2	pdxA, PMM0029
1375858	1377771	+	2	atpB, atpC
494294	496189	-	2	cobO, PMM0524
593891	595768	+	3	hisS, PMM0624, PMM0625

669400	671273	+	2	phoB, phoR
1371677	1373549	+	2	pgmI, secG
482478	484344	+	4	PMM0510, ppa, PMM0512, lepB
1161772	1163628	-	2	PMM1212, PMM1213
272674	274526	+	2	PMM0283, degT
551361	553213	-	2	PMM0585, PMM0586
186826	188657	-	2	rluD, rbgA
236686	238500	-	3	thyX, dcd, PMM0245
1057133	1058940	+	4	PMM1108, PMM1109, PMM1110, PMM1111
8416	10219	-	2	PMM0006, PMM0007
1110791	1112568	+	2	cobB, PMM1161
434126	435898	-	2	PMM0455, ubiA
1611496	1613264	+	2	PMM1678, lig
643157	644911	+	2	PMM0675, ispG
924726	926463	+	3	ureE, ureF, ureG
905364	907093	-	2	gatA, PMM0947
1491573	1493294	-	2	PMM1563, PMM1564
279339	281008	-	2	PMM0289, PMM0290
597856	599525	+	2	PMM0628, PMM0629
1101190	1102859	+	2	PMM1149, PMM1150
120073	121735	-	2	PMM0122, cysK2
165925	167573	+	2	ndhH, PMM0173
453269	454916	+	2	PMM0479, PMM0480
300941	302587	-	2	psbT, psbB
303280	304919	+	3	psbM, hemK, PMM0319
282495	284126	-	3	ndhJ, ndhK, ndhC
540814	542434	-	3	PMM0575, PMM0576, PMM0577
1646439	1648051	+	3	PMM1708, mraY, PMM1710
1515294	1516901	-	2	PMM1585, PMM1586
1444352	1445944	-	2	PMM1505, PMM1506
1438677	1440259	-	2	clpS, PMM1500
740106	741683	+	2	PMM0777, cobW
760044	761605	-	3	PMM0800, ycf39, PMM0802
999094	1000647	-	3	PMM1059, hisH, trxA
1129553	1131106	+	4	PMM1182, rpmH, rnpA, PMM1185
784615	786166	+	2	tpiA, folP
1104668	1106218	+	2	PMM1153, ilvH
1094783	1096332	+	2	dxr, PMM1143
634282	635821	-	2	hslO, PMM0666
226153	227666	-	2	pyrB, PMM0234
409270	410775	+	2	hisF, PMM0431
1040819	1042320	-	2	lipA,lip, recR
525728	527198	-	3	PMM0557, PMM0558, PMM0559
1487908	1489378	+	2	PMM1559, PMM1560
223922	225389	-	2	dfp, PMM0230
1604745	1606207	+	2	des9, rplI
284199	285650	+	2	rub, PMM0296
341600	343044	+	2	PMM0357, thiD
538345	539780	+	3	ndhL, PMM0571, trpA
955448	956880	-	2	purT, PMM1005
651412	652836	+	2	purK, SsrS
85259	86680	-	2	PMM0080, cysH
264658	266070	+	2	pyrE, PMM0276
886571	887982	+	2	psb28, PMM0927
239494	240903	+	2	PMM0247, PMM0248
113083	114483	+	3	PMM0111, rbfA, hemD
374367	375756	+	2	PMM0395, proC
820328	821686	-	3	PMM0862, cobU/cobP, PMM0864
1304849	1306207	+	2	PMM1355, PMM1356
429207	430551	+	2	PMM0450, PMM0451
882897	884227	+	2	PMM0921, PMM0922
1266710	1268011	+	2	clpP4, clpP
530222	531519	-	2	PMM0563, PMM0564
958893	960188	+	2	PMM1009, PMM1010
260796	262088	+	2	tgt, psbK
97375	98660	-	2	PMM0094, PMM0095
681401	682685	-	2	PMM0716, PMM0717
1351144	1352411	-	4	PMM1406, PMM1407, PMM1408, PMM1409
445994	447252	+	2	PMM0470, hli20
1390606	1391856	-	2	atpI, atp1
840919	842158	-	2	PMM0880, PMM0881
1120321	1121553	-	2	PMM1170, isiB
884216	885443	-	2	PMM0923, PMM0924

1128287	1129487	+	2	sppA, tyrA
196161	197360	-	2	rplA, rplK
172160	173343	-	2	gshB, PMM0179
309806	310987	+	2	petB, petD
1306399	1307560	+	2	PMM1357, PMM1358
997801	998954	-	2	PMM1056, cytM
961832	962973	+	2	PMM1013, PMM1014
305233	306371	-	2	minE, minD
476059	477191	+	2	PMM0503, PMM0504
1362259	1363390	-	3	PMM1420, PMM1421, PMM1422
940658	941780	+	3	PMM0985, PMM0986, rpsU
1103512	1104631	-	2	ycf39, petM
1245526	1246643	+	2	PMM1293, PMM1294
1139925	1141028	+	2	PMM1193, PMM1194
1423552	1424646	-	2	PMM1486, rpsT
808959	810036	+	2	PMM0851, PMM0852
27287	28353	+	2	efp, accB,fabE
1314462	1315494	-	2	PMM1367, PMM1368
1121694	1122700	+	2	PMM1172, PMM1173
31101	32091	-	2	PMM0032, PMM0033
160714	161695	+	2	PMM0165, cysC
574845	575813	-	2	PMM0605, lepB
71401	72368	-	2	PMM0067, def
141958	142919	+	2	PMM0145, PMM0146
357535	358490	-	3	PMM0376, PMM0377, PMM0378
121892	122846	+	2	PMM0124, PMM0125
1270652	1271598	-	3	PMM1318, PMM1319, PMM1320
969422	970361	+	3	PMM1023, PMM1024, PMM1025
742007	742924	+	2	PMM0779, purQ
136897	137805	-	2	PMM0139, ycf34
1456993	1457893	+	2	PMM1515, PMM1516
763548	764442	-	2	PMM0805, PMM0806
620091	620976	-	2	PMM0650, PMM0651
1449853	1450727	-	2	rps7, rpsL
344032	344904	+	2	PMM0360, PMM0361
240884	241753	-	2	PMM0249, pth
553268	554127	-	2	PMM0587, PMM0588
1569781	1570637	-	2	PMM1641, PMM1642
57789	58637	-	2	PMM0054, PMM0055
1354682	1355490	+	2	PMM1412, PMM1413
450648	451453	-	2	rplS, PMM0476
359690	360486	-	2	PMM0379, PMM0380
946932	947727	+	2	PMM0994, PMM0995
332317	333110	+	2	PMM0345, PMM0346
871171	871963	+	2	PMM0909, PMM0910
1517250	1518042	-	2	PMM1587, PMM1588
893891	894678	+	2	PMM0934, PMM0935
464964	465746	-	2	phhB, PMM0492
622839	623617	-	2	PMM0655, PMM0656
1118243	1118991	-	2	PMM1166, PMM1167
1301351	1302097	-	2	PMM1351, petF
1459606	1460348	-	2	psaL, psaI
285777	286512	+	4	psbE, psbF, psbL, psbJ
773065	773800	-	4	hli19, hli18, hli17, hli16
1344939	1345674	-	4	hli9, hli8, hli7, hli6
1570887	1571609	-	2	ribH, psbZ
1407861	1408582	-	2	PMM1473, PMM1474
445200	445919	-	2	psaJ, psaF
360886	361597	-	2	PMM0381, PMM0382
1296041	1296751	+	2	rplU, rpmA
294221	294926	-	2	cpeS, PMM0307
1363451	1364150	-	2	PMM1423, PMM1424
402171	402802	+	2	PMM0423, PMM0424
1368721	1369331	-	2	PMM1430, rpoZ
1395967	1396575	+	2	PMM1462, glnB
92390	92994	+	2	PMM0087, PMM0088
225590	226150	+	2	PMM0231, PMM0232
107394	107938	-	2	petL, PMM0103
249507	250049	+	2	PMM0259, PMM0260
510866	511407	+	2	psaM, PMM0541
950017	950551	+	2	PMM0999, PMM1000
980848	981347	-	2	PMM1036, PMM1037
665181	665674	+	2	PMM0699, PMM0700

936046	936539	-	2	PMM0979, PMM0980
663181	663662	-	2	PMM0696, PMM0697
658635	659028	-	2	hli22, hli21
1334613	1334860	+	2	hli12, hli11
